# Multifunctional bioactivity of *Portulaca oleracea* seed oil: GC-MS-based characterization and in vitro evaluation

**DOI:** 10.1038/s41598-026-55359-2

**Published:** 2026-06-13

**Authors:** Rushdy M. Ahmed, Hafsa Nour El-Din Abd El-Kader Ebrahim, Mahmoud A. Al-Saman, Mohamed Fawzy Ramadan, Rafaat M. Elsanhoty

**Affiliations:** 1 Special Food And Nutrition Department, Agriculture Research Center, Food Technology Research Institute, Giza, Egypt; 2 Food Technology Department, Faculty of Agriculture, Suez Canal University, Ismailia, Egypt; 3 Faculty of Biotechnology, University of Sadat City, 22857/79, Sadat City, Egypt; 4 Department of Clinical Nutrition, Faculty of Applied Medical Sciences, Umm Al-Qura University, Makkah, Kingdom of Saudi Arabia

**Keywords:** Antioxidant, Cytotoxicity, Cell lines, A549, HepG-2, MCF-7, Anti-inflammatory, Phenolics, Flavonoids, Biochemistry, Biotechnology, Cancer, Plant sciences

## Abstract

Purslane (*Portulaca oleracea* L.) is a widespread weed highly valued for its nutritional value, particularly its *omega*-3 fatty acid content. This study aimed to investigate the pharmacological properties and the phenolic and flavonoid contents of *P.* olec*racea* seed oil. *P. oleracea* seed oil was tested for antioxidant activity (DPPH· and ABTS), cell viability and cytotoxicity and anti-inflammatory activity. The contents of total phenolics, flavonoids, and constituents were characterized. The seed oil of *P.oleracea* had high flavonoid (4.53 mg/g) and phenolic (8.65 mg/g) content. The antioxidant activity of *P. oleracea* seed oil increased from 24.16% at a concentration of 0.5 µg/mL to 97.28% at a concentration of 1000 µg/mL. The MTT assay demonstrated that *P. oleracea* seed oil exhibited relatively weak to moderate cytotoxic activity against the A549, HepG-2, and MCF-7 cell lines. Lower concentrations of the seed oil extract exhibited anti-inflammatory activity, and these effects were compared with those of indomethacin as a positive control. GC-MS analysis showed that the seed oil of *P. oleracea* is rich in olean-12-ene-3,28-diol and 9-octadecenoic acid. It could be concluded that *P. oleracea* seed oil exhibits notable *in vitro* antioxidant and cytotoxic activities, which may be attributed to its content of flavonoids and phenolic compounds. However, further *in vivo* studies and toxicity evaluations are required to confirm its potential applications.

**Supplementary Information:**

The online version contains supplementary material available at https://doi.org/10.1038/s41598-026-55359-2.

## Introduction

*Portulaca oleracea* L., commonly referred to as purslane, is a weed that is widely distributed throughout the world, specifically in tropical and subtropical regions^1–7^. It is a member of the *Portulacacea* family. It is a perennial herbaceous plant that thrives in warm, humid climates and serves as a source of animal feed, medicine, and human consumption. Researchers have considered purslane “a weed with nutritive potential“^8^. The aerial parts of the plant (leaves and stems) are commonly used in vegetable dishes and as tea, especially in the Mediterranean region^9^. It is rich in many biologically active constituents, including alkaloids, flavonoids, coumarins, polysaccharides, *omega*-6 and *omega*-3 fatty acids, terpenoids, sterols, proteins, vitamins, and minerals^10–12^. Aerial plant parts contain high levels of α-linolenic acid (ALA), but the high levels of oxalic acid and nitrates hinder the widespread acceptance of the species in the human diet^13^. *P. oleracea* seeds are reddish-brown to black, oval, and tiny. It has slightly astringent, diuretic and carminative properties^8^. According to ethnobotanical studies, the seeds can be consumed by humans and animals and possess medicinal properties similar to those of the aerial parts^14^. Many plants are excellent suppliers of omega-3 fatty acids, some of which are now used in the food and pharmaceutical industries^15^. ALA is abundant in vegetable oils derived from species such as chia (*Salvia hispanica*) and flaxseed (*Linum usitatissimum*) at a rate of 52–55 and 61.3%, respectively, and 32.4% of ALA is found in purslane seeds^16^.

*P. oleracea* seed oil has highly nutritional value because it is rich in polyunsaturated fatty acids consisting mainly of linoleic acid, ALA and oleic acid. At the same time, it also contains phenolic compounds (protocatechuic and *p*-hydroxybenzoic acids) and phenolic lipids (alkylresorcinols)^17^. According to studies by Jalali et al.^18^, and Al-Sheddi et al.^19^,, the alcoholic seed extract exhibited antioxidant activity. It mitigated hydrogen peroxide-induced hepatocyte death in human cells. *P. oleracea* seeds improved lipid profiles in obese adolescents with dyslipidemia^20^. Moreover, *P. oleracea* seed oil can be used as a substitute for synthetic antioxidants in food preservation^21^. According to Farhadpour et al.^22^,, alcoholic seed extracts showed analgesic and cytotoxic effects on human hepatocellular carcinoma cells. As reported by Al-Sheddi et al.^23^,, the seed oil also exhibited cytotoxic effects on hepatocellular carcinoma and human lung cancer cell lines. Cytotoxic activity of the seed oil has been reported against cervical HeLa cells, esophageal Eca-109 cells, and breast MCF-7 cells. These effects were attributed to the suppression of MTA1, RhoA, Rac1/Cdc42, and MMP-2 expression, as well as inhibitory effects on MCF-7 cell proliferation and significant inhibition of HeLa and Eca-109 cell proliferation^24^. Oxidative stress, resulting from an imbalance between the production and neutralization of oxidants, is a leading cause of many diseases affecting modern societies. Free radicals produce oxidative stress when they bind to biological macromolecules, such as proteins, lipids, and DNA, in healthy human cells, attempting to stabilize them. This leads to damage to proteins and DNA, as well as lipid peroxidation^25^. These changes contribute to cancer, atherosclerosis, cardiovascular diseases, aging, and inflammatory diseases^26^. Although several studies have reported the antioxidant and pharmacological properties of *P. oleracea*, most have focused on crude extracts, with limited attention to seed oil. In addition, the relationship between chemical composition and biological activity remains insufficiently explored.

There is a strong correlation between inflammation, free radicals, and carcinogenesis. Anticancer drugs are highly valued when they exhibit anti-inflammatory and free radical scavenging properties. Cancer can be fatal due to several factors, including the lack of effective treatments, adverse drug reactions, and the high cost of chemotherapy drugs. To stop, delay, or even reverse the development of cancer, attempts are still being conducted to find powerful naturally occurring anti-cancer substances. Although studies have reported promising antioxidant and biological activities of *P. oleracea*, the reported effects vary considerably depending on the plant part, extraction method, solvent system, and analytical approach. In addition, limited information is available regarding the bioactivity of the seed oil, particularly its anti-inflammatory and cytotoxic properties. Therefore, the present study aimed to characterize the chemical composition and evaluate the antioxidant, anti-inflammatory, and* in vitro* cytotoxic activities of *P. oleracea* seed oil. It was hypothesized that the oil may exhibit biological activities associated with its bioactive constituents.

## Materials and methods

### Chemicals

All chemicals and standards used were of high analytical grade and purchased from Sigma-Aldrich (St. Louis, MO, USA).

### Sample collection

Plant collection and all related experimental procedures were conducted in accordance with relevant institutional, national, and international guidelines and legislation. Permissions for plant collection were obtained from the relevant authorities where required. *P. oleracea* is not considered an endangered or protected species.

The studied *P. oleracea* L. seed samples were collected from Waraq Island, Giza Governorate, Egypt and represent the Egyptian flora. The plant material was taxonomically authenticated by Prof. Safwat A. Azer (Flora and Phytotaxonomy Research Department, Horticultural Research Institute, Agricultural Research Center, Giza, Egypt)^27^. A voucher specimen was prepared and deposited in the Herbarium of the University of Sadat City, Egypt, under accession number No. PO-024-10 (*Portulaca oleracea* L.). The seeds were harvested from cultivated plants at maturity in October 2024, when the seed capsules turned dark brown to black and became dry and brittle, facilitating collection. The seeds were carefully cleaned to remove foreign matter (i.e., stones and small stalks), then dried under direct sunlight for 24 h.

### Oil extraction

Cold extraction was carried out using a ZX85 twin-screw vegetable oil expeller (Ruian Every Machinery, China)^28^. The oil temperature during extraction ranged from 20 to 22 °C. The nozzle diameter was 6 mm, while the resulting seed cake diameter ranged from 1.0 to 1.5 mm. Seeds were fed into the screw press at a rate of 2 kg/h with a rotational speed of 65 rpm. Oil samples were collected after 30 min of operation to ensure steady-state conditions. All extraction experiments were performed in triplicate. A jacketed cooling system was employed during extraction to prevent excessive heat generation due to compressive forces and to maintain the oil temperature within the specified range. The crude oil was centrifuged twice at 3500 × g for 10 min. After each centrifugation step, the supernatant was carefully transferred into clean dark glass vials to minimize light exposure. The oil extraction yield was recorded as 23% based on the initial seed weight. The obtained oil samples were stored in the dark at room temperature for no more than 72 h before analysis to minimize oxidative changes. No antioxidants or preservatives were added during storage.

### Total phenolic content (TPC)

TPC was determined using the method of Singleton et al.^29^,. Meanwhile, 3 mL of 10% Folin-Ciocalteau reagent was mixed with 50 µL of plant extract and 0.8 mL of 7.5% sodium bicarbonate solution. The reaction solution was incubated for 30 min at room temperature. The absorbance was measured at 765 nm using a Milton Roy spectrophotometer (Spectronic 1201).TPC was compared with a gallic acid standard curve and expressed as mg gallic acid equivalents (GAE)/g extract.

### Total flavonoid content (TFC)

TFC was quantified using the method of Chang et al.^30^. Briefly, 0.1 mL of the extract was mixed with 3.9 mL of distilled water and 0.3 mL of 5% sodium nitrite solution. The mixture was allowed to react for 5 min, then 0.3 mL of a 10% aluminum chloride solution was added. After 6 min, the mixture solution was treated with 2 mL of 1 mM sodium hydroxide. Finally, 2.4 mL of distilled water was added to all samples. Absorbance was read at 510 nm against a blank sample without reaction using a Milton Roy spectrophotometer (Spectronic 1201). TFC was expressed as mg quercetin equivalent (QE)/g extract.

### Antioxidant capacity

#### DPPH· assay

The radical scavenging activity was evaluated using the 2,2-diphenyl-1-picrylhydrazyl (DPPH·) assay according to Al-Saman et al.^31^,, with minor modifications. All measurements were performed in triplicate, and results were expressed as mean ± standard deviation (SD). The reaction mixture consisted of 3 mL of a methanolic solution of DPPH· (0.1 mM; 0.004%, w/v) and 40 µL of oil sample at different concentrations (0.5–1000 µg/mL), prepared by dilution in the extraction solvent. The mixture was vigorously shaken and incubated in the dark at 37 °C for 30 min. Absorbance was measured at 515 nm using a UV-visible spectrophotometer (Milton Roy, Spectronic 1201) with a 1 cm path length cuvette. A reagent blank (methanol without DPPH·) and a control (DPPH· solution without sample) were included, and absorbance values were corrected accordingly. Ascorbic acid was used as a positive control under the same experimental conditions. The DPPH· radical scavenging activity (%) was calculated using the following equation:

$$DPPH\cdot{\text{ }}scavenging{\text{ }}activity{\text{ }}\left( \% \right) = 100 \times (A_{0} - A_{1} )/A_{0}$$

where A₀ is the absorbance of the control, and A₁ is the absorbance of the sample.

% inhibition was calculated at different concentrations, while the IC₅₀ value (the concentration required to scavenge 50% of DPPH· radicals) was determined from the dose–response curve using GraphPad Prism (San Diego, CA, USA).

#### ABTS^+^ radical scavenging assay

Antioxidant activity was determined using the ABTS⁺ radical scavenging assay as described byLing et al.^32^. All experiments were performed in triplicate, and results were expressed as mean ± standard deviation (SD). The ABTS⁺ radical cation was generated by reacting ABTS stock solution (1.8 mM; Sigma, PN: A3219) with potassium persulfate (0.63 mM), and the mixture was allowed to stand in the dark at room temperature for 12–16 h. Before analysis, the solution was diluted with ethanol to obtain an absorbance of 0.700 ± 0.03 at 734 nm. Oil samples were diluted 1:10 (*v/v*) with 80% methanol. Subsequently, 190 µL of the ABTS⁺ working solution was mixed with 10 µL of the diluted sample in a 96-well microplate. Absorbance was measured at 734 nm using a microplate reader, and readings were recorded at 1 min intervals up to 13 min after initial mixing. A reagent blank (methanol without ABTS⁺) and a negative control (ABTS⁺ solution without sample) were included, and absorbance values were corrected accordingly. Ascorbic acid was used as a positive control under the same experimental conditions. The percentage of radical scavenging activity (%) was calculated using the following equation:

$$Free{\text{ }}radical{\text{ }}scavenging{\text{ }}activity{\text{ }}(\% ){\text{ }} = {\text{ }}[(An - As){\text{ }} \times {\text{ }}100]/An$$

where An is the absorbance of the negative control and As is the absorbance of the sample.

% inhibition was calculated at specific time points (13 min), while IC₅₀ values (the concentration required to inhibit 50% of ABTS⁺ radicals) were determined from dose–response curves using GraphPad Prism (San Diego, CA, USA).

### Cell viability and cytotoxic effects

#### Mammalian cell lines

The human lung cancer cell line (A549), human hepatocellular carcinoma cell line (HepG2), and human breast cancer cell line (MCF-7) were obtained from the American Type Culture Collection (ATCC, Rockville, MD, USA).

#### Cell lines propagation

Cells were cultured on RPMI-1640 medium supplemented with 50 µg/mL gentamycin and 10% inactivated fetal calf serum. Cells were subcultured 2 or 3 times per week and maintained at 37˚C in a humidified environment containing 5% CO_2_.

#### Cytotoxicity assay

Cancer cell lines were suspended in medium at a concentration of 5 × 10^4^ cells/well in Corning^®^ 96-well tissue culture plates and then incubated for 24 h. The tested concentrations of *P.oleracea* seed oil were added to plates (3 replicates). Vehicle controls were run with media or 0.5% dimethyl sulfoxide (DMSO) per 96-well plate as a control. After 24 h incubation, the number of viable cells was determined using the MTT assay. The media was removed from the 96-well plate and replaced with 100 µL of fresh RPMI 1640 medium without phenol red, followed by 10 µL of the 12 mM MTT stock solution (5 mg of MTT in 1 mL of PBS) per well, including untreated controls. The plates were incubated at 37^°^C and 5% CO_2_ for 4 h. An 85 µL aliquot of media was removed from the wells, 50 µL of DMSO was added to each well, the mixture was mixed with a pipette, and the wells were incubated at 37^°^C for 10 min. The optical density (OD) was measured at 590 nm using a microplate reader (SunRise, TECAN, USA) to determine the number of viable cells.

The percentage viability was calculated as [(OD_t_/OD_c_)]x100%, where OD_t_ is the mean OD of wells treated with the tested sample, and OD_c_ is the mean OD of untreated cells. The relationship between surviving cells and drug concentration was plotted to obtain a survival curve for each cancer cell line after treatment with the specified sample. Using Graphpad Prism software (San Diego, CA, USA), the graphic plots of the dose response curve for each concentration were used to determine the 50% inhibitory concentration (IC_50_), or the concentration required to cause toxic effects in 50% of intact cells^33^.

#### in vitro COX-1 inhibition assay

The in vitro inhibitory activity of *P. oleracea* seed oil against cyclooxygenase-1 (COX-1) was evaluated using a commercial COX-1 inhibitor screening assay kit (catalog no. K548, BioVision, USA), following the manufacturer’s instructions. Test samples were dissolved in DMSO and prepared at concentrations ranging from 0.25 to 500 µg/mL. A cofactor solution (50 µL) containing glutathione, hematin (1.0 mM), and N, N,N′,N′-tetramethyl-p-phenylenediamine (TMPD) in Tris-HCl buffer (pH 8.0) was added to the activated enzyme. The reaction mixtures, consisting of 60 µL of enzyme solution and 20 µL of test sample, were pre-incubated at room temperature for 5 min. The enzymatic reaction was initiated by adding 20 µL of arachidonic acid (substrate) and incubating for 5 min. Absorbance was measured at 570 nm using a spectrophotometer. Indomethacin was used as a positive control under the same experimental conditions. A control (without inhibitor) and appropriate blanks were included, and absorbance values were corrected accordingly. All experiments were performed in triplicate. The percentage inhibition of COX-1 activity was calculated relative to the control, and the IC₅₀ value was determined from the concentration–response curve using GraphPad Prism^34^.

### Determination of the main components in *P. oleracea* seed oil

#### Fatty acid methyl ester (FAME) preparation

Using boron trifluoride-methanol complex (14%, *w/v*) for 30 min at 50^°^C, acidic-catalyzed esterification was employed^35^. A 5.0 mL of water containing 0.2 mL of glacial acetic acid was added to stop the reaction. A 3.0 mL petroleum ether was used to extract esterified fatty acids (40–60^°^C).

#### GC/MS analysis

A Thermo Scientific TRACE 1310 Gas Chromatograph was used in conjunction with an ISQ LT (single quadrupole Mass Spectrometer) for the GC-MS analysis of FAMS of *P.oleracea* seed oil. DB5-MS, 30 m; 0.25 mm ID (J&W Scientific) was the column. The carrier gas was helium at a flow rate of 1.0 mL/min. Starting at 40^°^C and maintaining that temperature for 3 min; increasing to 280^°^C with a 5.0^°^C/min and maintaining that temperature for 5 min; and increasing to 290^°^C with a 7.5^°^C/min and maintaining that temperature for 1 min. Temperatures of 200^°^C for injection and 300^°^C for detection were used. Electron ionization (EI) was used to obtain mass spectra at 70 eV, with a m/z range of 40–450. WILEY & NIST MASS SPECTRAL DATA BASE identified the compounds. Authentic chemicals were also used for identification when available. The identities of some components were confirmed by mass spectral and retention data from authentic chemicals obtained under identical GC-MS conditions, and the concentrations of the selected compounds were calculated from standard calibration curves.

### Statistical analysis

All data were subjected to one-way analysis of variance (ANOVA). Before analysis, data were tested for normality and homogeneity of variances to ensure compliance with the assumptions of parametric tests. Biological replicates (*n* = 3 independent samples per treatment) were used, and all measurements were performed in triplicate (technical replicates). Results are expressed as mean ± standard deviation (SD). Significant differences among means were determined using Duncan’s multiple range test at a significance level of *p* ≤ 0.05. Statistical analyses were performed using SPSS (version 16, IBM, USA). IC₅₀ values were estimated by nonlinear regression analysis using GraphPad Prism (San Diego, CA, USA), where applicable^36^.

## Results and discussion

### TPC and TFC

Plants contain phenolic compounds in large quantities, and these compounds have drawn considerable interest due to their antioxidant properties and their capacity to scavenge free radicals. Plants used in food and medicine that contain phenolic compounds have been shown to reduce oxidative stress^37^.In this study, TPC was determined relative to standard gallic acid; results were expressed as mg of gallic acid equivalent (mg GAE/g oil), and TFC was expressed as mg of quercetin equivalent (mg QE/g seed oil). The TPC and TFC values of purslane (*Portulaca oleracea* L.) seed oil are given in Table 1. The amounts of TPC and TFC were 8.65 and 4.53 mg/g, respectively. Delfan-Hosseini et al.^38^, found TPC values for solvent-extracted purslane seed oils (0.19 mg GAE/g oil), while Sodeifian et al.^39^, reported the concentration of TPC in purslane seed oil obtained using supercritical fluid and solvent extraction (1.73 and 1.67 mg GAE/g oil, respectively). According to Petropoulos et al.^28^, the hot extraction method yielded a higher TPC than the cold extraction method. Amirul Alam et al.^40^, observed that TPC ranged from 0.96 to 9.12 mg/g DW and TFC from 0.13 to 1.44 mg/g DW in 13 collected purslane samples analyzed for TPC and TFC. These differences in literature reports could be attributed to different extraction parameters or genetic variations of the materials. The high content of TPC and TFC observed in the study may be attributed to differences in the extraction matrix and methodology. Notably, the values are expressed per g of extracted oil rather than per g of dry plant material, which limits the direct comparison with some literature reports. Oil-based extracts may concentrate lipophilic phenolic constituents, potentially contributing to higher apparent values. Therefore, comparisons should be interpreted with caution.

**Table 1:** Total phenolic compounds and flavonoid content of *Portulaca oleracea* seed oil.

	Concentration
Total phenolic content	8.65 ± 0.37*
Total flavonoid content	4.53 ± 0.29**

*mg GAE/g; **mg quercetin equivalent g^− 1^.

### Radical scavenging activity of *P. oleracea* seed oil

To learn more about the potential health benefits of *P.oleracea* seeds, we tested the antioxidant capacity of P. *oleracea* seed oil. Table 2 shows that all different concentrations of seed oil scavenged the DPPH· radical in a dose-dependent manner. It was observed that the scavenging effectiveness increased with increasing seed oil concentration. Antioxidant activity was measured using the DPPH· method with ascorbic acid as the standard. Ascorbic acid concentrations ranged from 1000 to 0.5 µg/mL. Ascorbic acid at 0.5 µg/mL showed a % inhibition of 9.34%, and at 1000 µg/mL, 98.65%.

**Table 2:** Radical scavenging activity of *P.oleracea* seed oil toward DPPH· and ABTS.

Conc. (µg/mL)	DPPH·	Standard	ABTS	Standard
1000	97.28 ± 0.64^*^	98.65 ± 0.10^**^	96.89 ± 0.37	95.46 ± 2.24
500	94.96 ± 0.82	96.34 ± 0.65	92.78 ± 0.16	95.30 ± 1.31
250	92.37 ± 0.91	94.25 ± 0.92	91.04 ± 0.32	93.05 ± 0.49
125	88.25 ± 0.73	90.89 ± 0.17	85.32 ± 0.46	91.29 ± 0.76
62.5	82.74 ± 1.28	86.42 ± 0.71	78.40 ± 0.62	85.22 ± 0.99
31.25	71.39 ± 1.75	79.35 ± 1.22	65.83 ± 0.95	72.28 ± 2.52
15.6	63.45 ± 1.31	65.05 ± 0.21	59.28 ± 1.06	62.36 ± 1.69
7.8	54.96 ± 0.82	43.01 ± 2.87	49.72 ± 0.84	42.39 ± 3.66
3.9	48.21 ± 1.23	31.04 ± 1.04	38.05 ± 0.69	33.10 ± 1.22
2	40.95 ± 0.69	22.18 ± 1.37	31.47 ± 1.21	24.98 ± 1.39
1	32.79 ± 0.37	14.48 ± 0.62	24.53 ± 0.38	18.13 ± 2.26
0.5	24.16 ± 0.42	09.34 ± 0.46	17.85 ± 0.91	10.87 ± 1.47

^*^Radical scavenging activity given as percentage inhibition.

^**^The percentage inhibition value of the standard compound, ascorbic acid.

According to statistical analysis, the highest scavenging percentage (97.28%) was observed at 1000 µg/mL, followed by 500 µg/mL (94.96%) and 250 µg/mL (92.37%). On the other hand, a concentration of 0.5 µg/mL showed less effective scavenging, with an average of 24.16%. The IC_50_ value of ascorbic acid was 10.21 ± 0.77 µg/mL, and the IC_50_ value for seed oil was 4.93 ± 0.43 µg/mL.

ABTS^+^ radical cation inhibition or hunting capacities of *P.oleracea* seed oil were investigated in comparison with standard ascorbic acid. ABTS^+^ free radicals were generated by mixing ABTS^+^with potassium persulfate. A spectrophotometer was used to measure at 734 nm under dark conditions. The higher ABTS^+^ radical-scavenging potential of seed oil was demonstrated at various concentrations. The highest ABTS^+^ radical scavenging activity was observed with standard ascorbic acid at 1000 µg/mL (95.46 ± 2.24%). The concentration of 1000 µg/mL of seed oil showed the maximum radical-scavenging potential of 96.89 ± 0.37%, as shown in Table 2. The IC_50_ value of ascorbic acid was 10.66 ± 0.89 µg/mL, and the IC_50_ value for seed oil was 8.02 ± 0.69 µg/mL. Although the seed oil exhibited a lower IC₅₀ value than ascorbic acid under the tested conditions, direct comparison should be interpreted cautiously due to differences in chemical composition, solubility behavior, and potential matrix effects between the complex oil sample and the pure reference compound. Therefore, the observed activity may reflect the combined action of multiple bioactive constituents present in the oil rather than the effect of a single antioxidant compound.

Increasing levels of *omega*−3 polyunsaturated fatty acids (PUFAs) have been linked to improved antioxidant activity^8,41^. Linolenic acid (an *omega*-3 fatty acid) appears to be a predominant component of *P. oleracea*seed oil. However, the observed antioxidant activity is likely influenced by a combination of bioactive constituents, and the specific contribution of linolenic acid cannot be confirmed without detailed compositional and correlation analyses. Tiveron^42^, reported that *P. oleracea* presents the lowest IC_50_ necessary to reduce 50% of DPPH· free radicals; moreover, the highest ascorbic acid content was found in the *P. oleracea* seed oil, followed by the leaf oil. This result may be attributed to the presence of multiple bioactive compounds acting synergistically in the oil. In summary, we observed antioxidant activity in *P. oleracea* seed oil, suggesting that it is a potential candidate as a food and pharmaceutical ingredient.

### Antiproliferative effects on HepG2, A-549, and MCF-7 cells

**Fig. 1:**
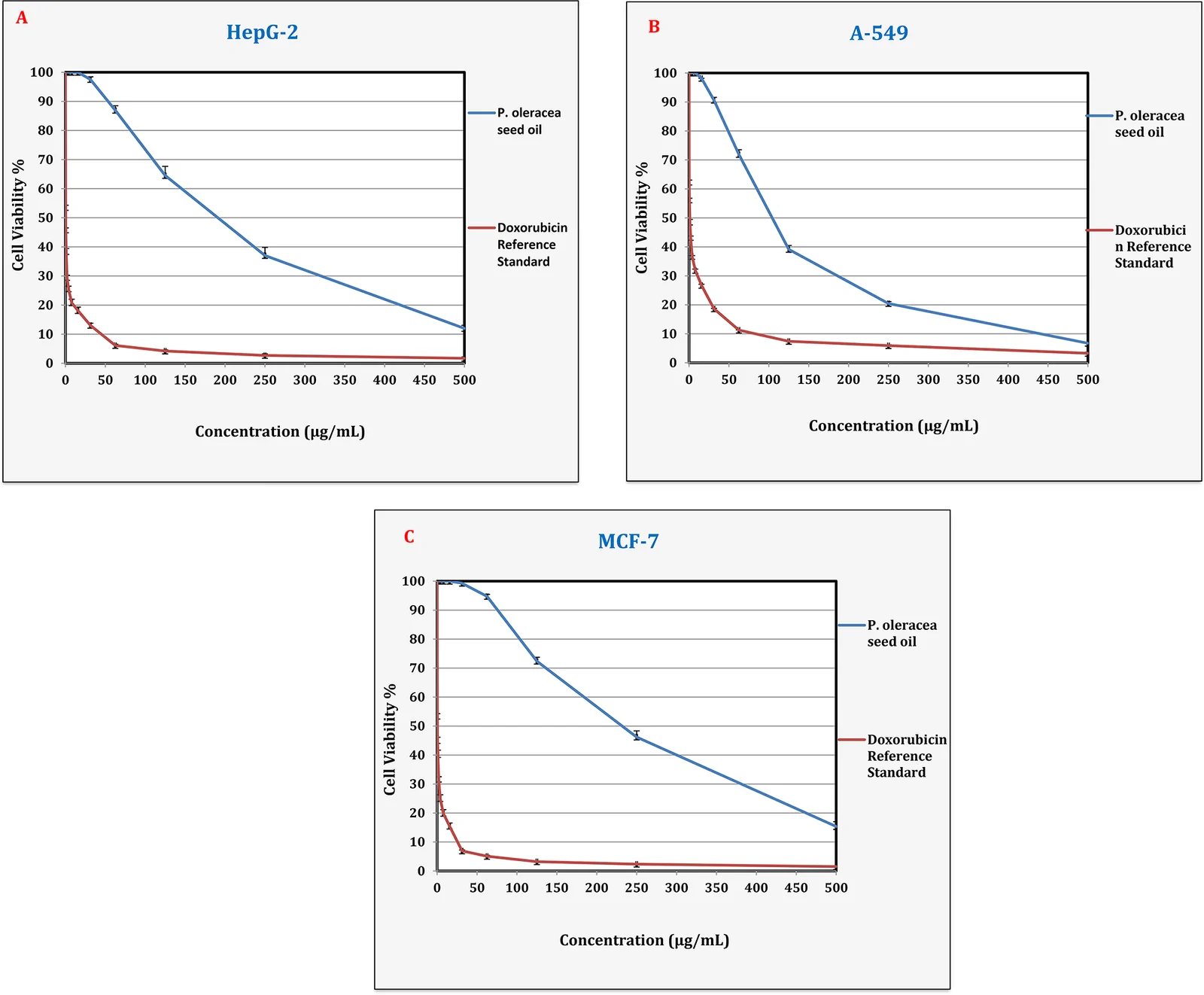
Toxicity effects of the *P.oleracea* seed oil against cancer cell lines: (A) HepG-2, (B) A-549, and (C) MCF-7 after 24 h of incubation.

Three cancer cell lines (HepG2, A549, and MCF-7) were used to assess the cytotoxicity of P. *oleracea* seed oil. The microculture tetrazolium (MTT) assay was used to assess the cytotoxicity of *P. oleracea* oil in cell lines. Compared with the control, cytotoxic activity was expressed as a percentage of cell viability in the HepG2, A549, and MCF-7 cell lines. Multiple concentrations of *P.oleracea* oil were used, and effective doses were calculated from a dose-response curve. A dose-response curve was used to calculate effective doses using several concentrations of *P.oleracea* oil. The results of the cytotoxicity test of *P. oleracea* oil against several cell lines are shown in Fig. 1. The cytotoxicity of *P. oleracea* oil was studied against the HepG2 cell line. Doxorubicin (reference standard) has an IC_50_ of 0.37 (0.09 µg/mL), while *P. oleracea* oil had an IC_50_ of 191.05 ± 4.59 µg/mL (Fig. 1A). The cytotoxicity of *P. oleracea* oil against the A549 cell line was investigated. The IC_50_ value of doxorubicin was 0.91 ± 0.13 µg/mL, while the IC_50_ value of *P. oleracea* oil was 104.26 ± 2.38 µg/mL (Fig. 1B).

On the other hand, the cytotoxicity of *P. oleracea* oil against the MCF-7 cell line was also evaluated. The IC_50_ value of doxorubicin was 0.35±0.07 µg/mL and the IC_50_ value of *P. oleracea* oil was 231.90 ± 5.14 µg/mL (Fig. 1C). Upon treatment with *P. oleracea* seed oil, A549 cells showed an increased rate of cell death compared to that of HepG-2 and MCF-7 cell lines at the same concentration of the oil used. A549 cells exhibited a higher rate of cell death after treatment with *P. oleracea s*eed oil compared to HepG-2, and MCF-7 cell lines at the same oil concentration.

Previous studies have found a relationship between antioxidant activity and the anticancer properties of plant extracts^43^, and there is evidence suggesting that phytochemicals in *P. oleracea* may have anticancer effects^44^. Alkaloids inhibit lung and breast cancer with moderate cytotoxic activities against A549, weak cytotoxic activities against K562, and low cytotoxic activity against MCF-7 and MDA-MB-435 cells. Another bioactivity of *P. oleracea* is portulacacerebrosie A, a cerebroside compound that suppresses the invasion and metastasis of HCCLM3 cells and is used to treat leucocytosis^45^. The bioactivity of *P. oleracea* and its potential to create new products from this underutilized plant in some regions warrant consideration, given its pharmacological qualities and potential value as a functional food. Several mechanisms underlying *P. oleracea’s* anticancer activity have been investigated. Cancer cell lines are cytotoxically affected by seed oil, hydroethanolic extracts, and aqueous extracts^46^.

#### *in vitro* anti-inflammatory activity

**Fig. 2:**
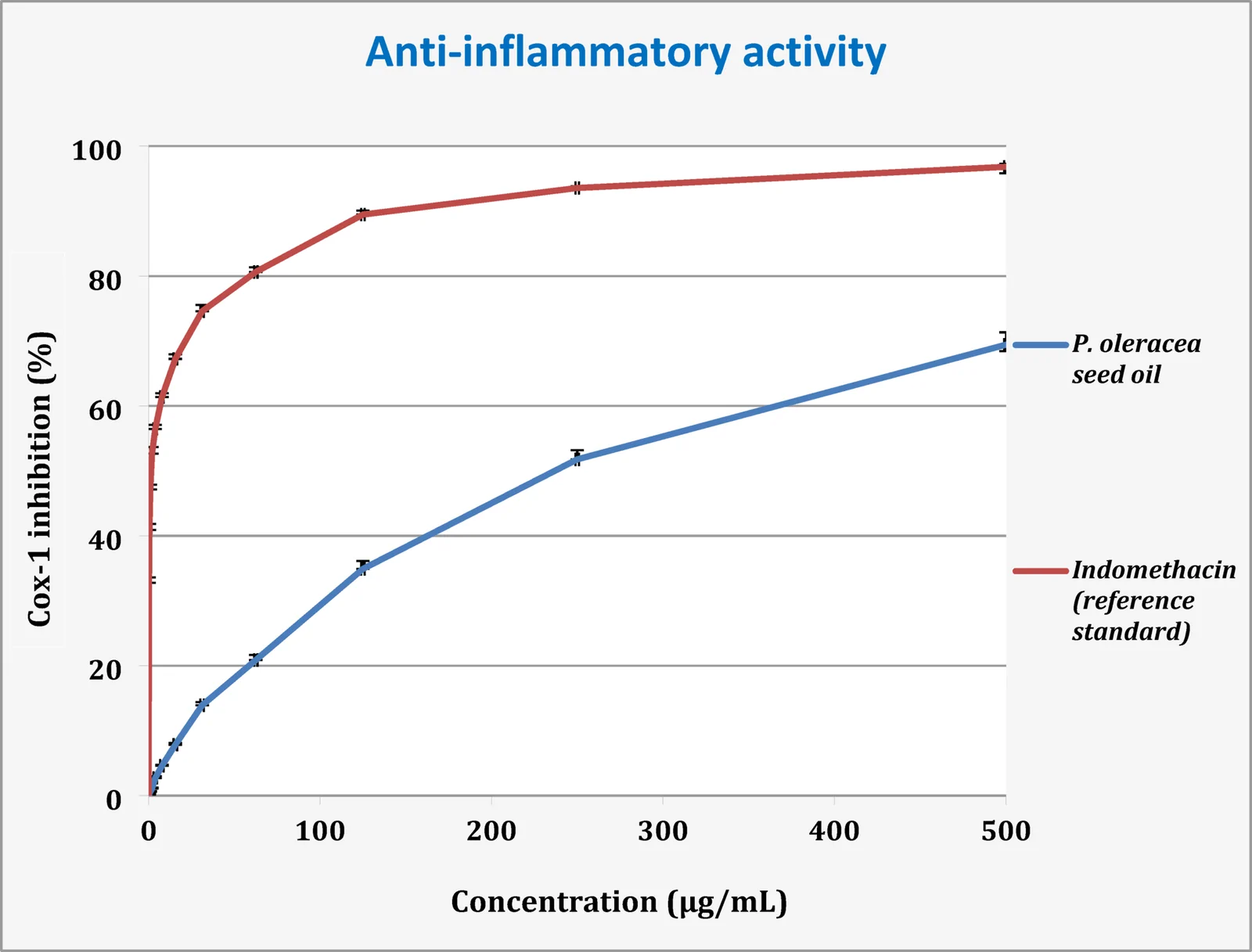
in vitro anti-inflammatory activity of *P.oleracea* seed oil.

*P.oleracea* seed oil showed inhibition of COX-1 isoenzymes at different concentrations (500, 250, 125, 62.5, 31.25, 15.63, 7.8, 3.9, 2, 1, 0.5 and 0.25 µg/mL). The protection percentage for *P.oleracea* seed oil was 69.43 at a concentration of 500 µg/mL. However, at low concentrations, low protection was observed. The results were shown in Fig. 2. The IC_50_ value for indomethacin was 1.54 ± 0.26 µg/mL; however, the IC_50_ value for *P.oleracea* oil was 236.81 ± 6.23 µg/mL. In the acute stage, inflammation is characterized by the accumulation of inflammatory cells (leukocytes) and mediators, as well as increased vascular permeability. Inflammation is the tissue’s defensive response to injury. Various cytokines, including interleukin (IL)−1 (α and β), tumor necrosis factor-alpha (TNF-α), IL-6, IL-8, and IL-11, are useful in acute inflammatory reactions^47^. Arachidonic acid metabolites, chemokines, cytokines, and free radicals are sources of inflammatory mediators that increase angiogenesis, mutagenesis, oncogene activation, and cell proliferation^48^. Medical herbs and their active ingredients are among the best candidates for new drugs and effective remedies, although non-steroidal and steroidal anti-inflammatory agents are currently used to treat acute and chronic inflammatory conditions such as rheumatoid arthritis. However, they showed significant side effects with long-term administration^49^. Ethanolic extract of *P. oleracea* reduced nitric oxide (NO) production and suppressed mRNA expression of inflammatory markers such as TNF-α and IL-1β. In addition, incubation of lipopolysaccharide (LPS)-stimulated human peripheral blood mononuclear cells with the hydroalcoholic extract from aerial parts of *P. oleracea* decreased the concentrations of TNF-α and IL-6^50^. Seed oil exhibited relatively low COX-1 inhibitory activity compared to indomethacin, as reflected by its higher IC₅₀ value.

#### GC-MS analysis of P. oleracea seed oil

**Fig. 3:**
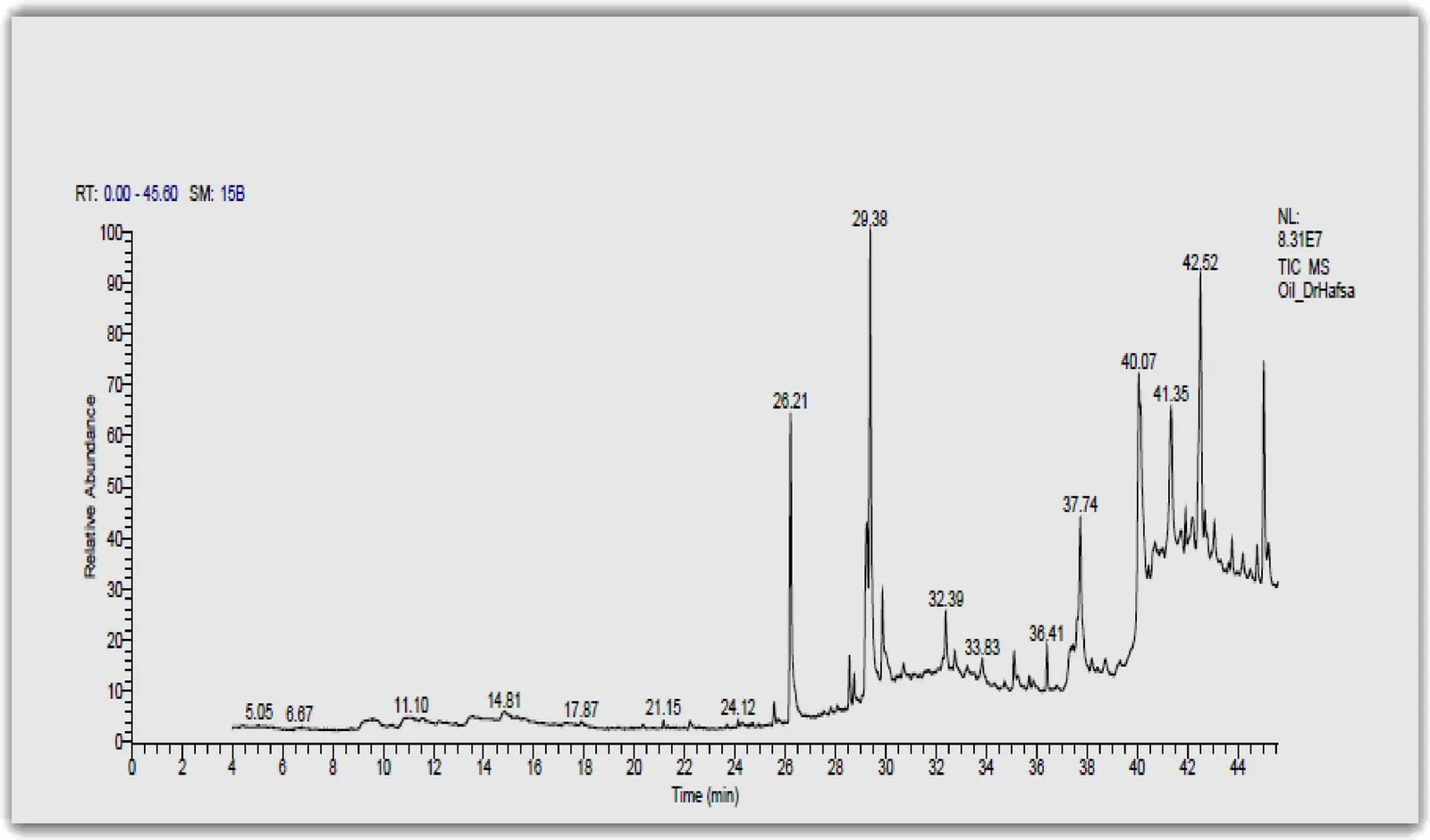
GC-MS chromatogram of purslane (*Portulaca oleracea* L.) seed oil.

The components of *P.oleracea* seed oil were determined using GC-MS based on their retention times and peak areas (Table 3; Fig. 3). Nine major bioactive substances were identified and categorized based on their chemical structures using GC-MS analysis. N-hexadecanoic acid (8.89), 9,12-octadecadienoic acid (Z, Z)- (4.96), 9-octadecenoic acid, (E)- (11.61), octadecanoic acid (3.53), oxiraneoctanoic acid, 3-octyl-, cis- (2.07%), 9-octadecenoic acid (z)-, 2-hydroxy-1-(hydroxyl methyl) ethyl ester (4.91%), betulin (8.64%), olean-12-ene-3,28-diol, (3á)- (12.17%), and á-sitosterol (6.94%) were the majoridentified components. Several additional peaks were observed in the chromatogram that could not be precisely identified. Therefore, the total explained peak area does not account for the entire oil composition. The presence of unidentified peaks suggests that other secondary constituents may also contribute to the overall biological activity. Compounds were tentatively identified by mass spectral matching against library databases, without retention index (RI) determination or authentic standard validation.

**Table 3:** Main components identified in*P.oleracea* seed oil.

Compound	Retention time (min)	Molecular formula	m/z fragments	Peak Area percentage^*^
n-Hexadecanoic acid (palmitic)	26.21	(C_16_H_32_O_2_) 256	43, 60, 73^#^, 129, 213, 256	8.89
9,12-Octadecadienoic acid (Z, Z)- (linoleic/omega-6)	29.21	(C_18_H_32_O_2_) 280	41, 55, 67^#^, 81, 95	4.96
**9-Octadecenoic acid**,** (E) - (Oleic)**	29.38	(C_18_H_34_O_2_) 282	55^#^, 69, 83, 97	11.61
Octadecanoic acid (stearic)	29.87	(C_18_H_36_O_2_) 284	29, 41, 43^#^, 55, 60, 73	3.53
Oxiraneoctanoic acid, 3-octyl-, cis-	32.39	(C_18_H_34_O_3_) 298	41, 55^#^, 69, 155	2.07
9-Octadecenoic acid (z)-, 2-hydroxy-1- (hydroxymethyl) ethyl ester	37.74	(C_21_H_40_O_4_) 356	41, 55^#^, 69, 81, 264	4.91
Betulin	40.06	(C_30_H_50_O_2_) 442	55, 81, 95,135, 189^#^	8.64
Olean-12-ene-3,28-diol, (3á)-	42.52	(C_30_H_50_O_2_) 442	203^#^, 204, 234	12.17
á-Sitosterol	45.04	(C_29_H_50_O) 414	41, 43, 55, 57, 81, 105	6.94

^*^The most intense ion is indicated by the base peak (tallest peak).

^#^(Peak area of individual compound/total peak areas of all compoundss) x100.

The results were consistent with those of Jilan et al.^51^, who suggested that oleamide, β-amyrin, ethyl palmitate, stigmasterol and β-sitosterol were the main fatty acids isolated from *P. oleracea*seed. The outcomes aligned with the findings of AynurGunenc et al.^17^, who suggested that linoleic acid (C18:2) was the most prevalent kind of linoleic acid found in purslane seed oil, followed by oleic acid (C18:1) and α-linolenic acid (C18:3); and Osama and Hussein^52^ who reported that 9,12,15-octadecatrienoic acid methyl ester and 9,12-octadecadienoic acid methyl ester represented 41.18% and 27.23%, respectively.GC–MS analysis revealed the presence of several bioactive compounds, including triterpenoids (e.g., betulin and olean-12-ene-3,28-diol) and phytosterols (e.g., α-sitosterol), which have been previously reported to exhibit antioxidant and anti-inflammatory activities. However, the relative peak area percentages obtained from GC–MS analysis are semi-quantitative and do not necessarily reflect the actual concentrations or bioavailability of these compounds. Therefore, it is difficult to attribute the observed biological activities to specific constituents directly. The observed bioactivities are more likely the result of synergistic interactions among multiple components rather than a single dominant compound.

## Conclusion

Several analyses in this study revealed that the *P.oleracea* seed oil is rich in bioactive compounds. *P. oleoracea* seed oil had the highest scavenging potency at 1000 µg/mL. The seed oil showed sufficient inhibition of COX-1 to be considered an anti-inflammatory agent. *in vitro* models showed that the seed oil exhibited moderate to weak cytotoxicity across several cancer cell lines, particularly compared with the reference drug doxorubicin.GC-MS analysis showed that olean-12-ene-3,28-diol, (3á)-was the most abundant component, with a value of approximately 12.17%, followed by 9-octadecenoic acid, (E)- (oleic) 11.61. Generally, this study revealed a protective effect of *P. oleracea* seed oil against several human cancer cell lines, which may be related to its antioxidant and anti-inflammatory activities. Although the tested oil exhibited cytotoxic effects against cancer cell lines, the lack of evaluation on normal cell lines limits conclusions regarding selectivity and biosafety, warranting further investigation. In summary, *P. oleracea* seed oil has the potential to serve as an easily accessible source of naturally occurring bioactive compounds with therapeutic applications. These findings may be of potential interest for pharmaceutical applications; however, further *in vivo* studies are required to validate the biological effects and assess safety. *P. oleracea* seed oil could be considered a promising natural source of bioactive compounds.

## Supplementary Information

Below is the link to the electronic supplementary material.


Supplementary Material 1


## Data Availability

The data presented in this study are available in this manuscript.
