# Supplementary material for: Multifunctional bioactivity of Portulaca oleracea seed oil: GC-MS-based characterization and in vitro evaluation
Source: Sci Rep. 2026 Jun 13;16:27063. doi: 10.1038/s41598-026-55359-2 (PMC13522612; doi:10.1038/s41598-026-55359-2)

My GC-MS Report

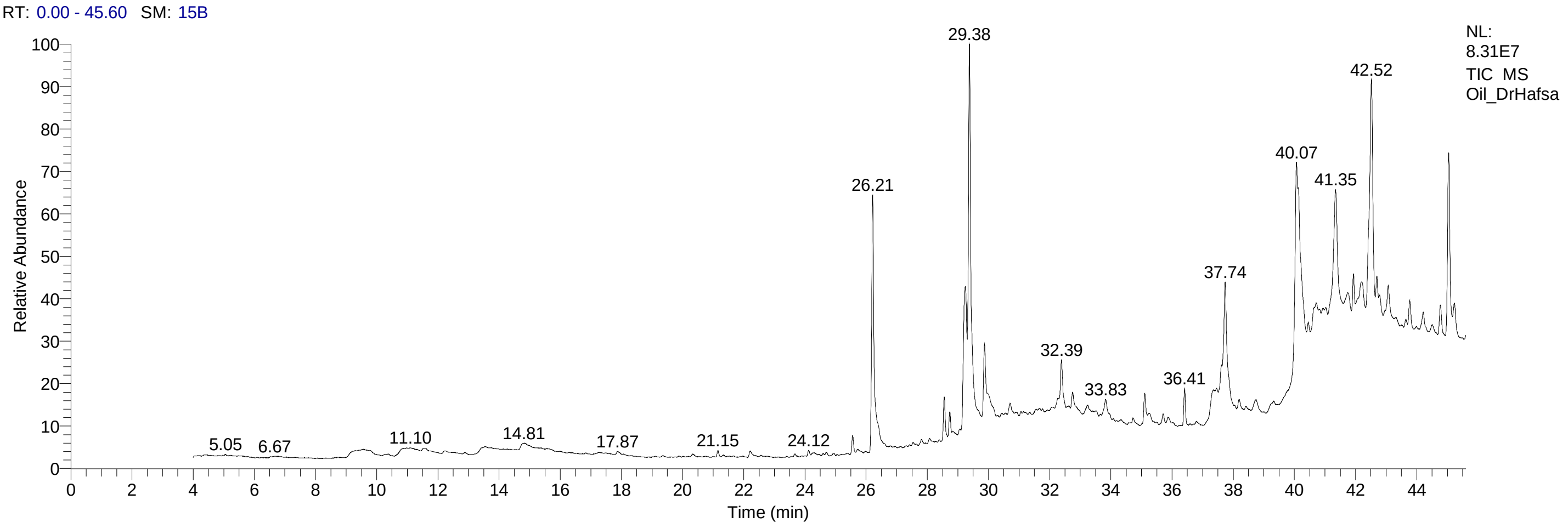

RT	Area %	Peak Area	Peak Height
10.82	0.23	5105236.15	887818.68
10.88	0.21	4691862.64	930288.09
10.98	0.26	5729138.23	915376.17
14.78	0.68	15239693.33	1009515.26
21.15	0.21	4780352.16	1400120.89
22.20	0.28	6168694.26	1187183.13
24.12	0.18	4058293.71	1208447.00
25.56	0.62	13979317.86	4102213.38
26.21	8.89	199356688.19	52082751.24
27.81	0.27	6141790.49	1234942.00
28.07	0.26	5840709.64	1010571.42
28.55	1.45	32606155.38	9618725.32
28.73	0.83	18697756.48	5636719.65
28.83	0.27	6018675.17	976228.86
29.05	0.12	2612445.08	854335.12
29.21	4.96	111188431.12	17809391.33
29.38	11.61	260320100.87	68983652.16
29.87	3.53	79114122.76	15060925.97
30.71	0.61	13625030.58	2323991.27
31.06	0.14	3089227.59	974716.06
31.54	0.23	5243847.33	934626.09
31.67	0.25	5656263.30	973959.94
32.27	0.36	8176969.90	1905843.54
32.39	2.07	46455503.97	10063343.48
32.75	0.78	17588056.84	3633739.50
32.87	0.28	6266657.87	983736.70
33.24	0.48	10827935.87	1642966.25
33.53	0.19	4245602.02	843926.81
33.83	1.04	23251642.33	3635707.00
33.96	0.13	2829957.76	971663.77
34.73	0.32	7139416.69	1465227.43
35.10	1.27	28418743.78	6504431.42
35.25	0.51	11334162.27	1686943.64
35.71	0.38	8612945.44	2112020.02
35.87	0.26	5841196.07	1146276.93
36.41	1.26	28335022.14	8242609.85
37.31	1.21	27233200.45	3177925.40
37.45	0.28	6371310.06	1778209.57
37.61	0.29	6425782.28	2607411.48
37.74	4.91	110163777.57	19970457.34
38.19	0.43	9587924.44	2119847.88
38.41	0.20	4532102.13	867208.93

My GC-MS Report

RT	Area %	Peak Area	Peak Height
38.74	0.58	12974837.85	1876042.09
39.23	0.17	3843028.03	904480.84
39.31	0.25	5551512.41	1001667.04
40.06	8.64	193743202.28	30054391.41
40.45	0.32	7134270.05	2455749.51
40.63	0.51	11361603.62	3339965.02
40.72	1.02	22892055.09	3650335.53
40.82	0.19	4287754.46	1549119.90
40.94	0.24	5379983.05	1339910.01
41.03	0.27	5978907.31	1382547.72
41.35	5.15	115347353.19	19099261.01
41.76	0.94	20990382.24	2861952.34
41.93	1.01	22730506.21	8029941.11
42.17	0.54	12215713.63	3952151.73
42.22	0.72	16082704.13	4201724.63
42.52	12.17	272709385.90	42649116.05
42.69	0.84	18858374.03	5916999.39
42.79	0.43	9712221.48	2647745.04
43.07	1.53	34363150.48	6160232.04
43.64	0.33	7324736.88	1681712.63
43.77	1.10	24599834.22	5739196.40
44.21	0.92	20529953.02	3767849.67
44.50	0.42	9315859.94	1512783.18
44.78	1.21	27022301.78	6236839.99
45.04	6.94	155547815.43	35825186.75
45.23	0.81	18071060.73	4342059.99

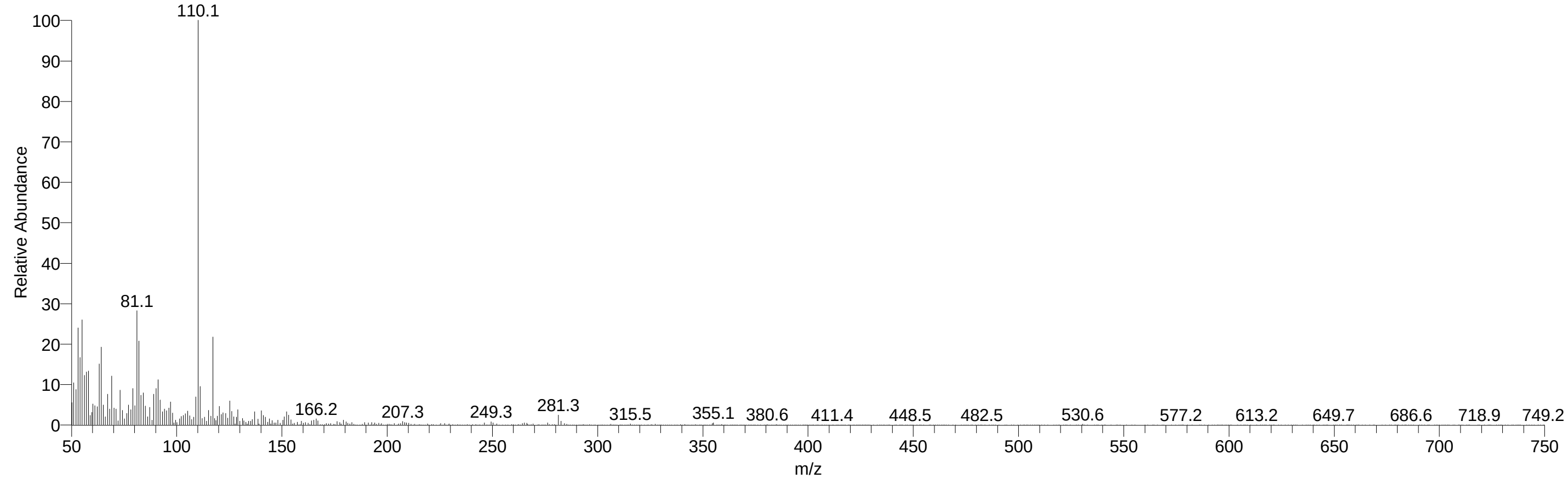

RT	Compound Name	Area %	MF	Molecular Formula	Molecular Weight	Cas #	Library
10.82	HYDROCHINON	0.23	902	C6H6O2	110	NA	WileyRegistry8e
10.82	1,2-15,16-Diepoxyhexadecane	0.23	621	C16H30O2	254	NA	mainlib
10.82	2-[12-(2-OXIRANYL)DODECYL]OXIRANE	0.23	621	C16H30O2	254	NA	WileyRegistry8e
10.82	Octahydrochromen-2-one	0.23	707	C9H14O2	154	NA	mainlib
10.82	OCTAHYDRO-2H-CHROMEN-2-ONE	0.23	707	C9H14O2	154	NA	WileyRegistry8e

My GC-MS Report

Compound Structure | Hit Spectrum

HYDROCHINON
Formula C6H6O2, MW 110, CAS# NA, Entry# 359374
HYDROQUINONE

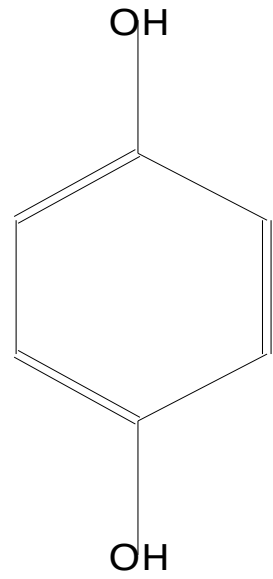

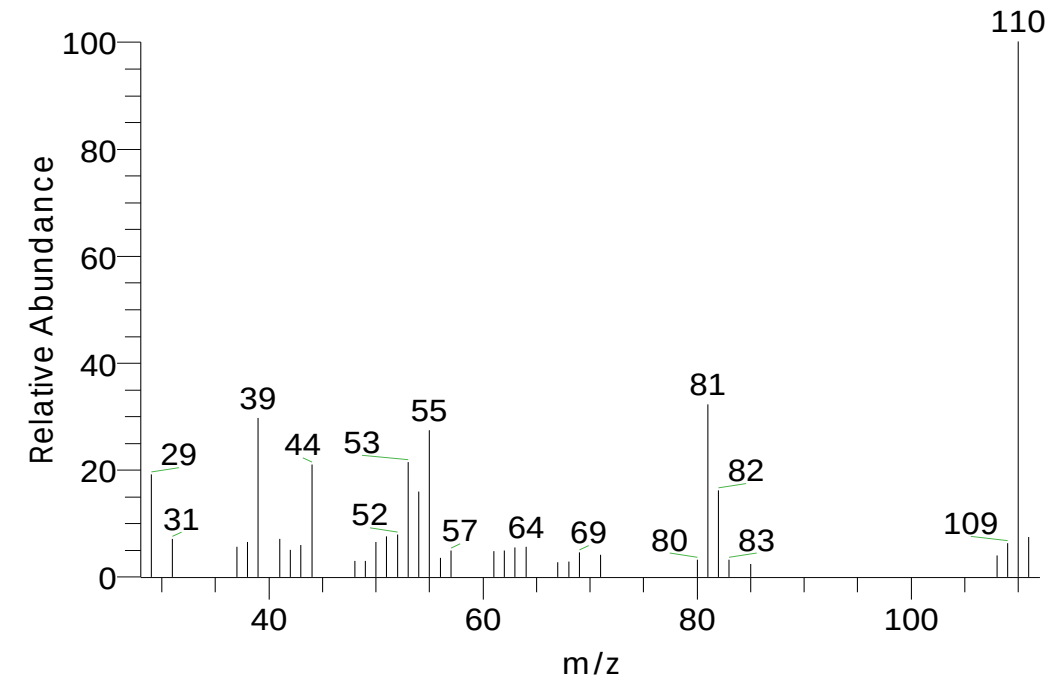

1,2-15,16-Diepoxyhexadecane
Formula C16H30O2, MW 254, CAS# NA, Entry# 19336
2-[12-(2-Oxiranyl)dodecyl]oxirane #

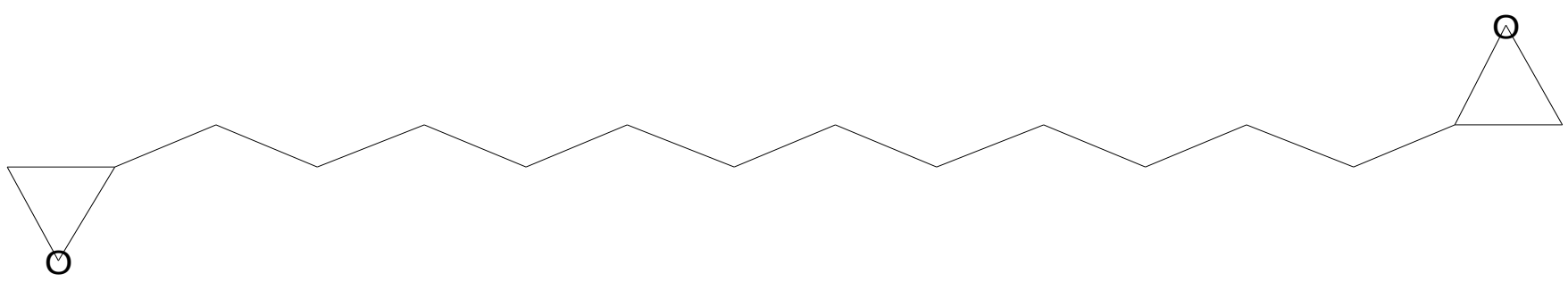

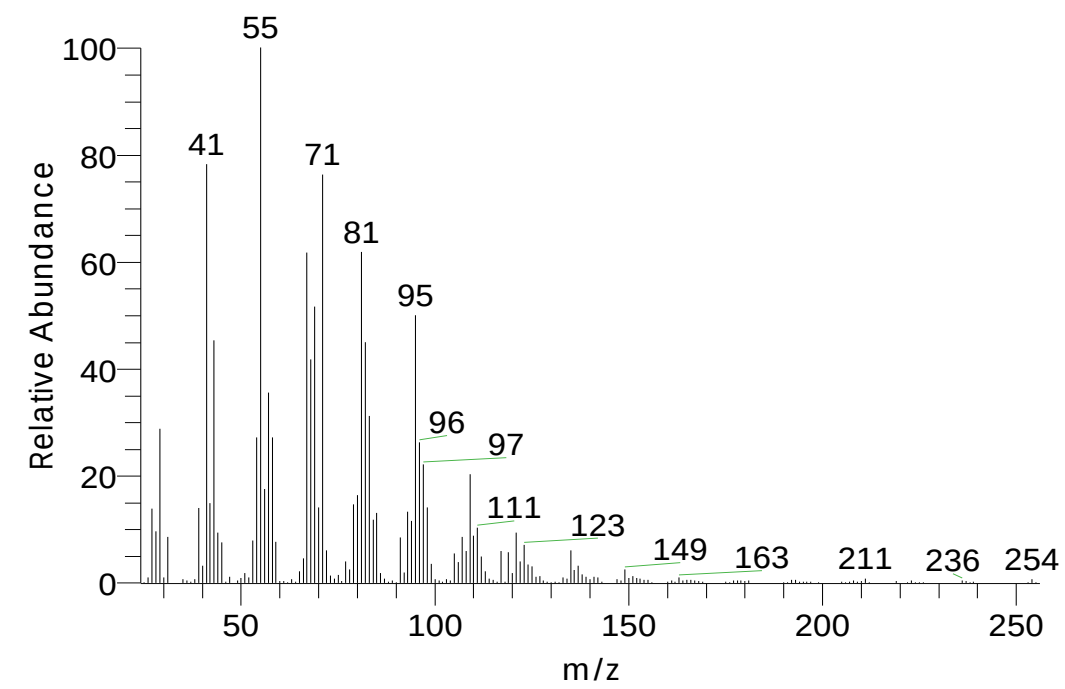

2-[12-(2-OXIRANYL)DODECYL]OXIRANE
Formula C16H30O2, MW 254, CAS# NA, Entry# 369137

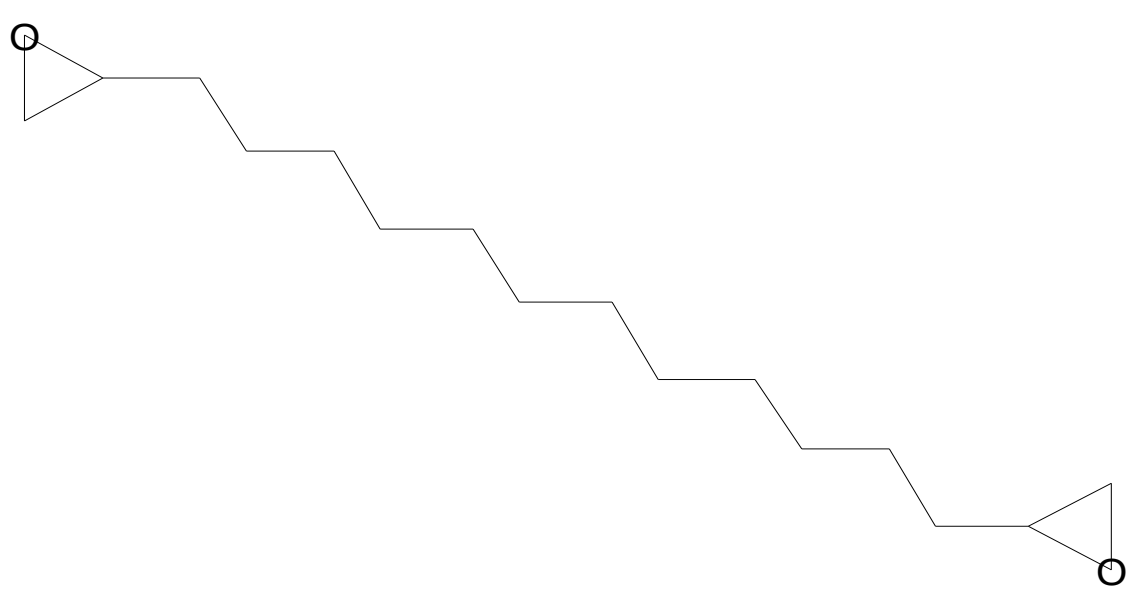

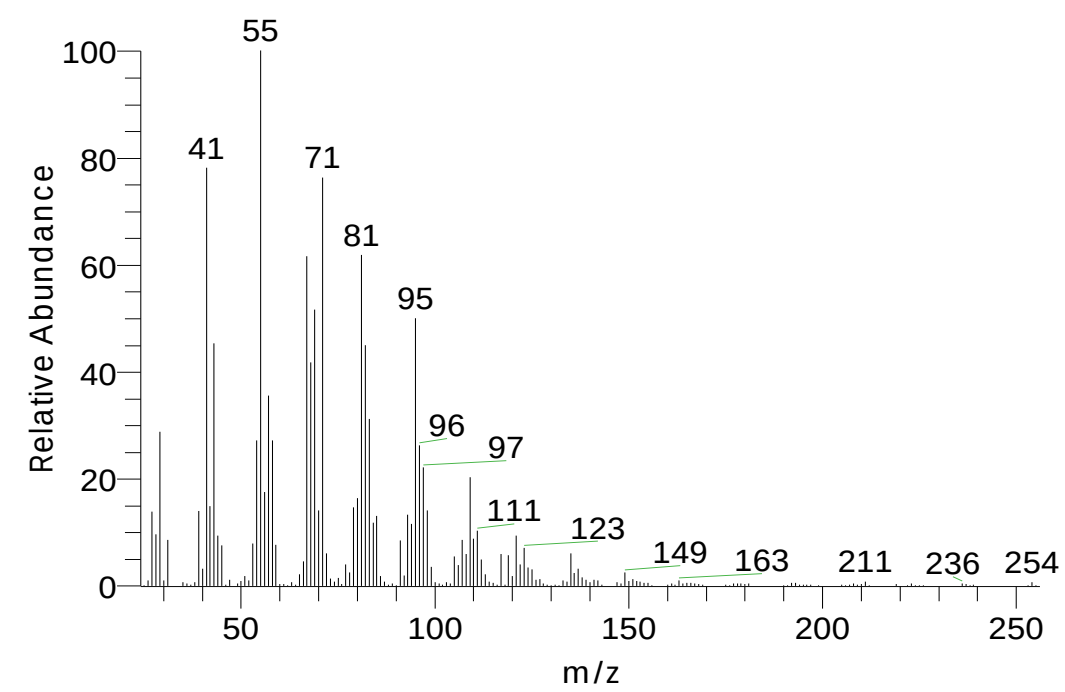

Octahydrochromen-2-one
Formula C9H14O2, MW 154, CAS# NA, Entry# 32958
Octahydro-2H-chromen-2-one #

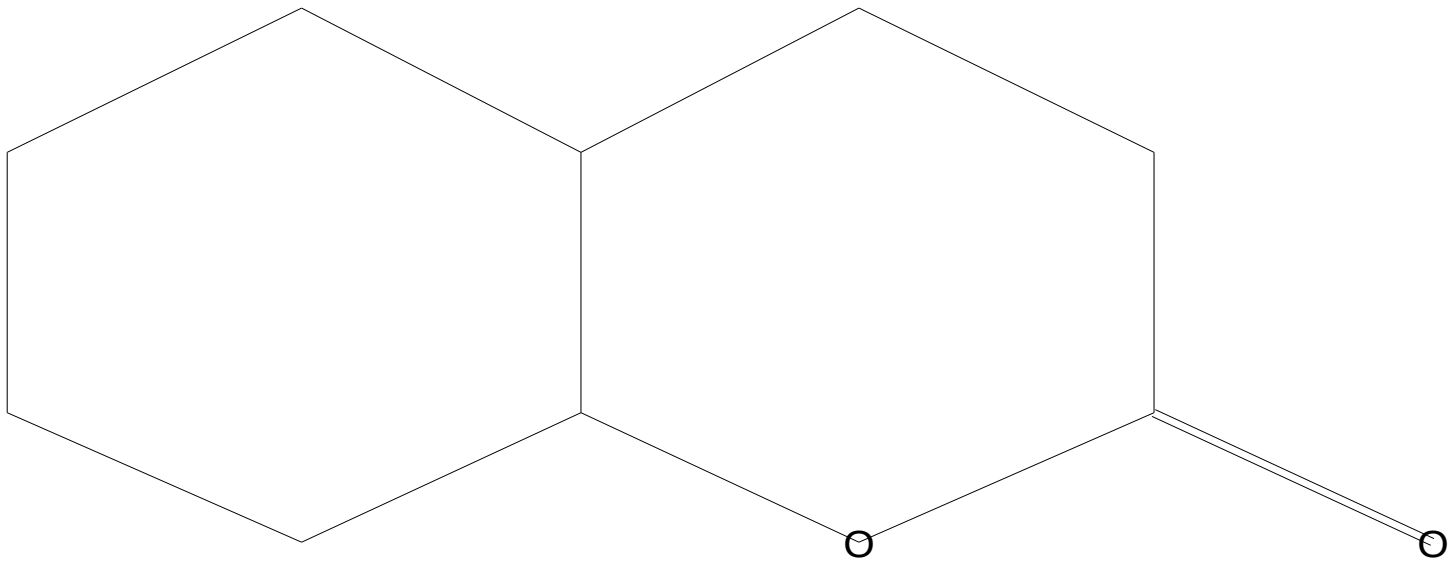

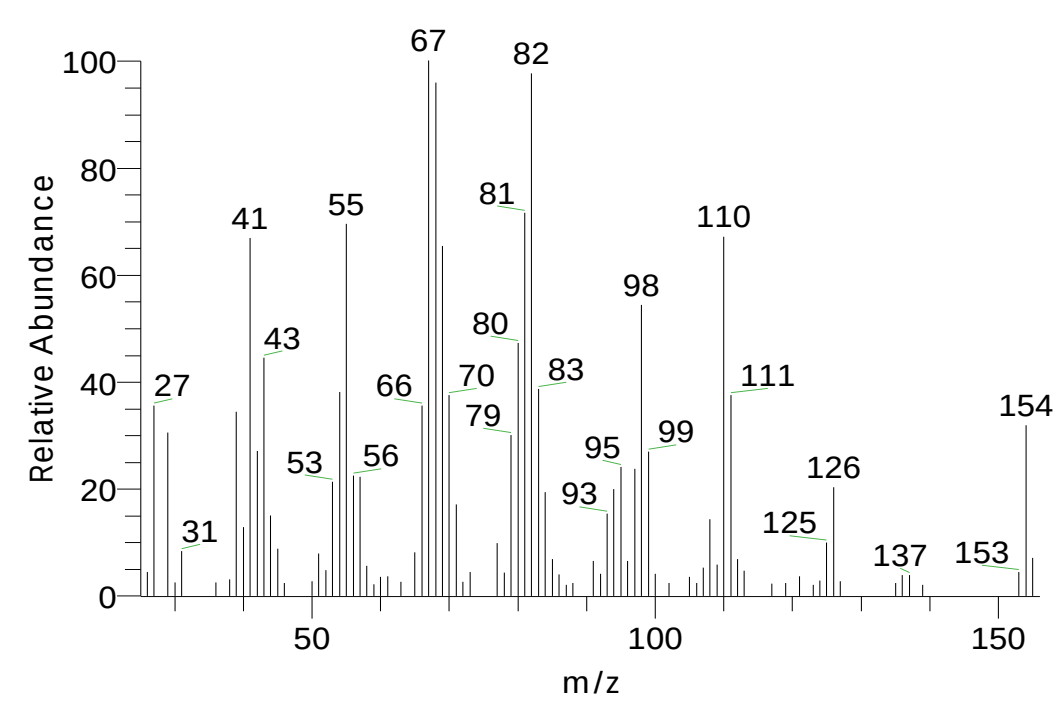

My GC-MS Report

Compound Structure | Hit Spectrum

OCTAHYDRO-2H-CHROMEN-2-ONE
Formula C9H14O2, MW 154, CAS# NA, Entry# 368330
OCTAHYDRO-CHROMEN-2-ONE

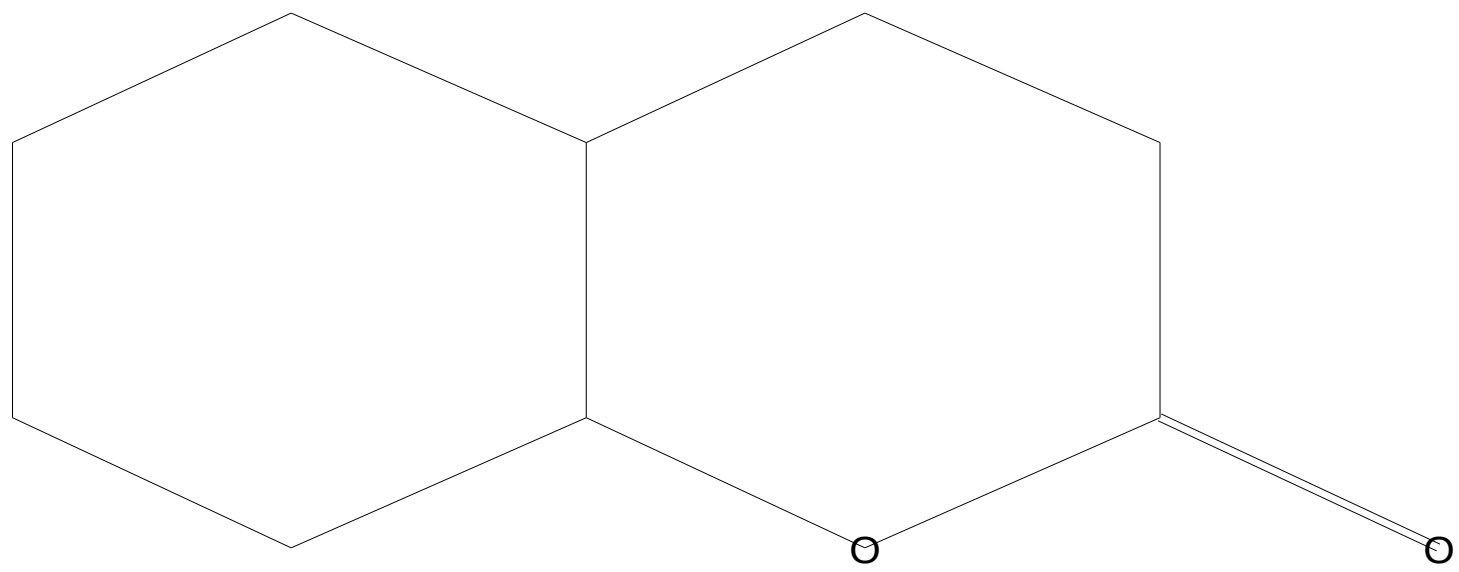

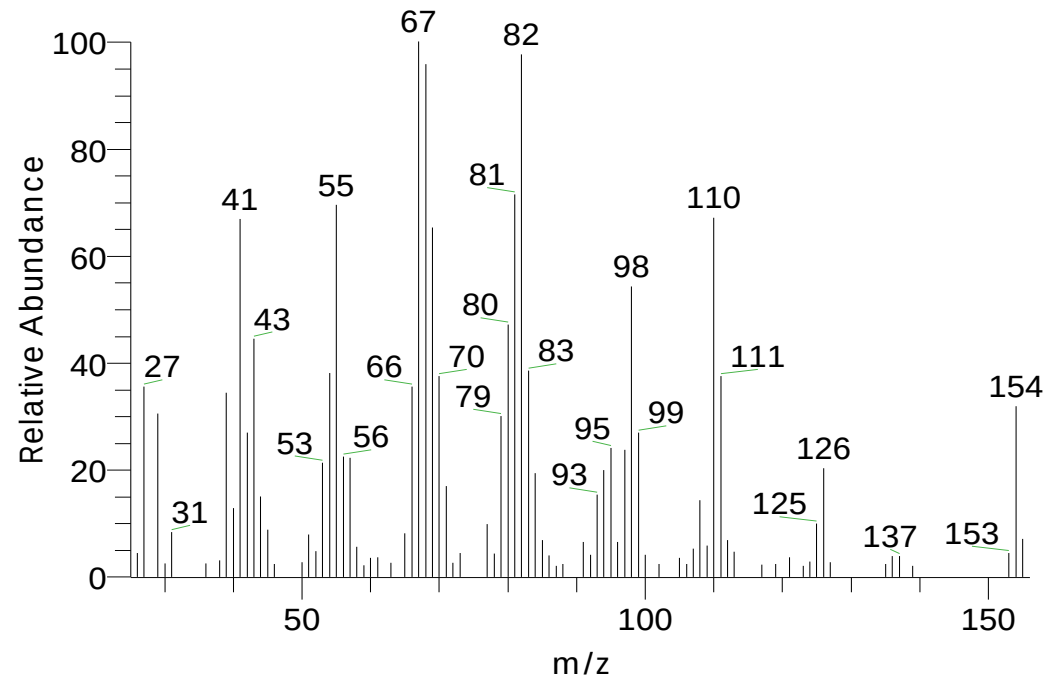

Oil_DrHafsa #2052 RT: 10.88 AV: 1 NL: 6.42E5
T: + c EI Full ms [50.000-750.000]

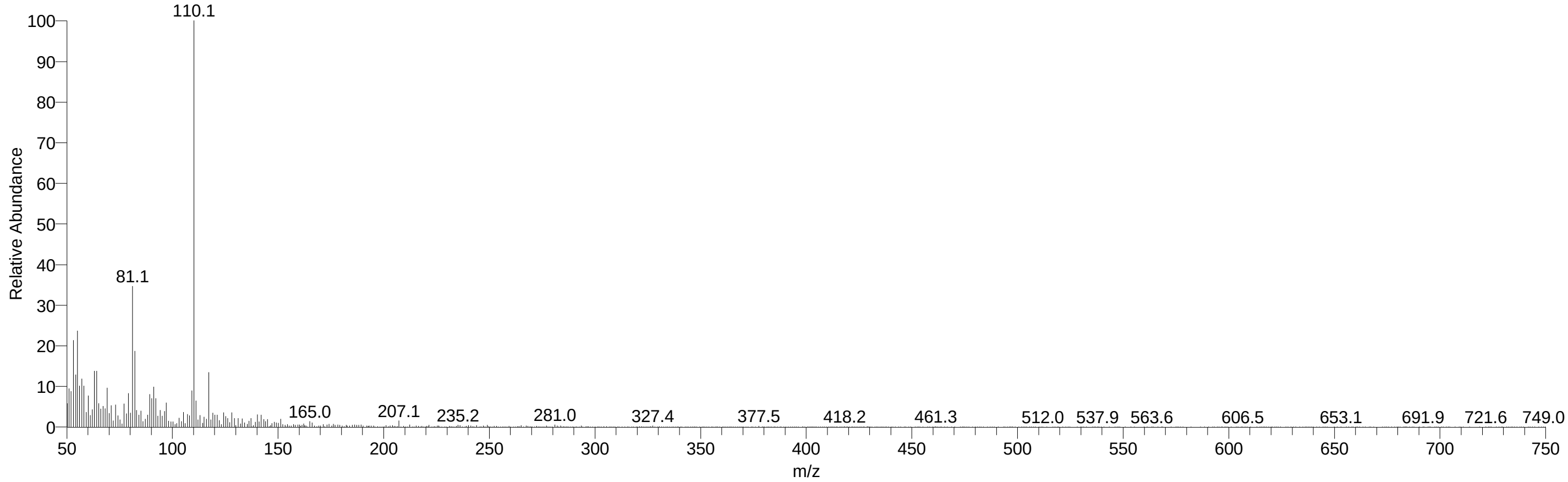

RT	Compound Name	Area %	MF	Molecular Formula	Molecular Weight	Cas #	Library
10.88	HYDROCHINON	0.21	932	C6H6O2	110	NA	WileyRegistry8e
10.88	1,2-BENZENEDIOL	0.21	753	C6H6O2	110	120-80-9	WileyRegistry8e
10.88	1,3-BENZENEDIOL, 4,4'-THIOBIS-	0.21	778	C12H10O4S	250	97-29-0	WileyRegistry8e
10.88	1,4-BENZENEDIOL	0.21	799	C6H6O2	110	123-31-9	WileyRegistry8e
10.88	Hydroquinone	0.21	817	C6H6O2	110	123-31-9	mainlib

Compound Structure | Hit Spectrum

HYDROCHINON
Formula C6H6O2, MW 110, CAS# NA, Entry# 359374
HYDROQUINONE

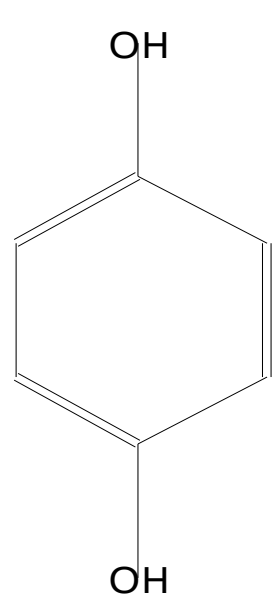

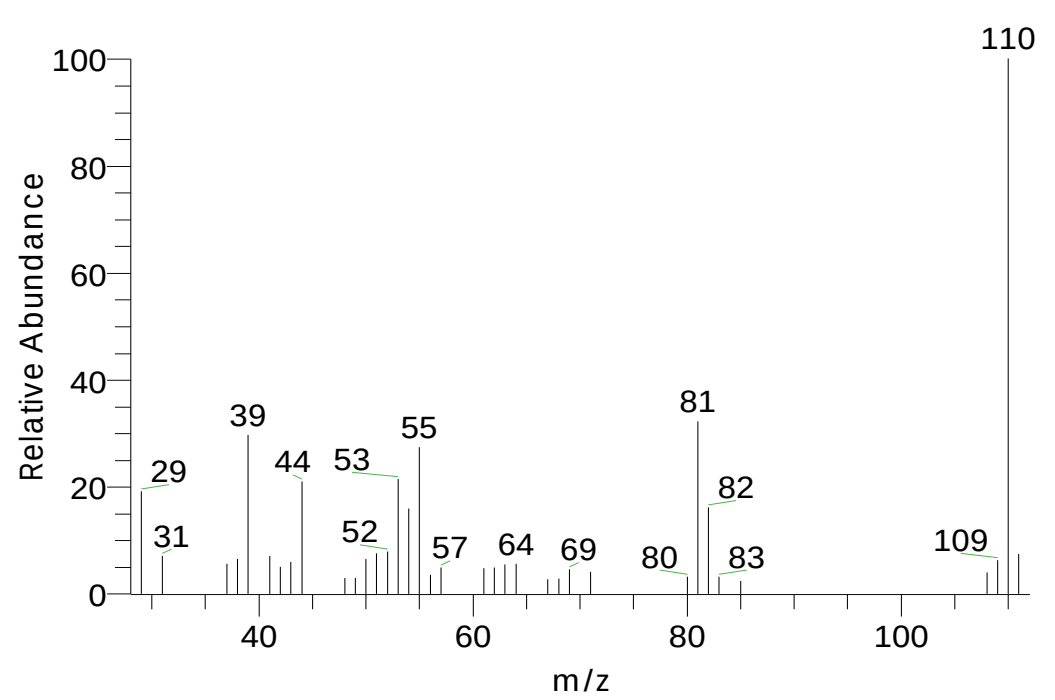

My GC-MS Report

Compound Structure | Hit Spectrum

1,2-BENZENEDIOL
Formula C6H6O2, MW 110, CAS# 120-80-9, Entry# 8910
BENZENE-1,2-DIOL

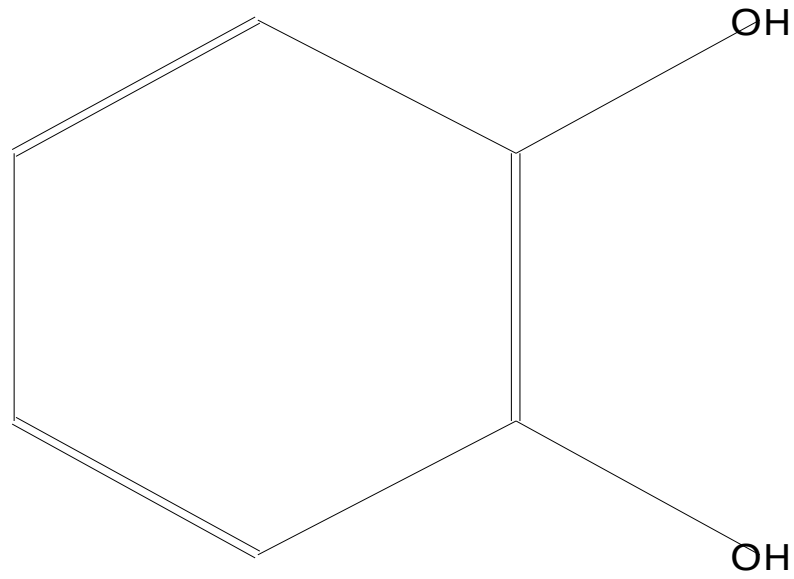

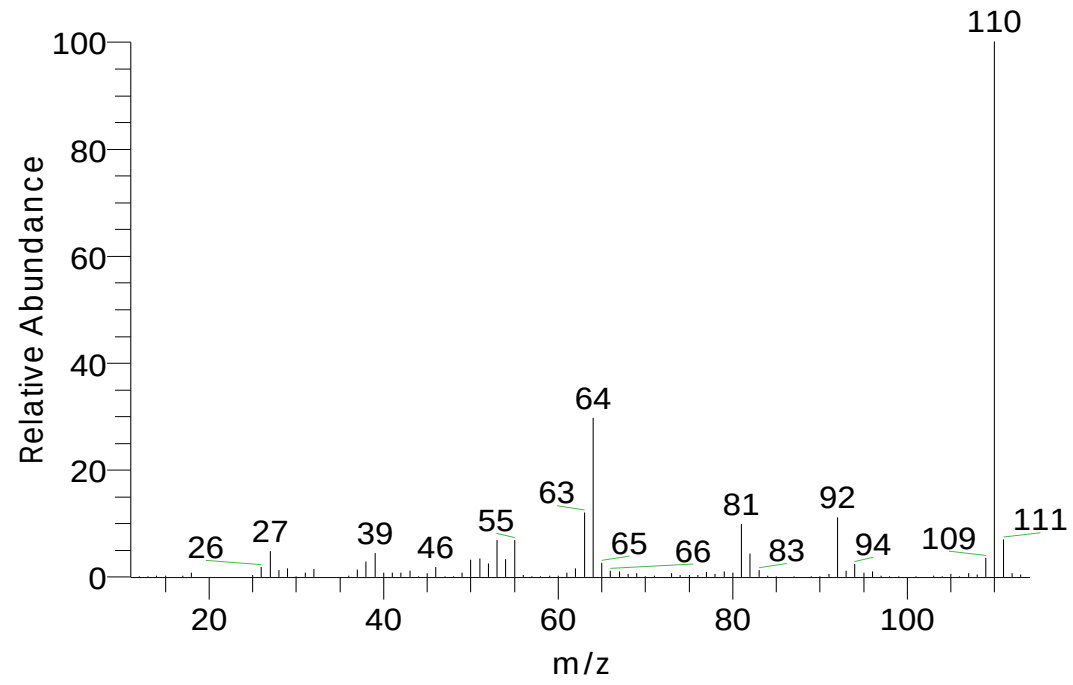

1,3-BENZENEDIOL, 4,4'-THIOBIS-
Formula C12H10O4S, MW 250, CAS# 97-29-0, Entry# 139461
2,2',4, 4'-TETRAHYDROXYDIPHENYL SULFIDE

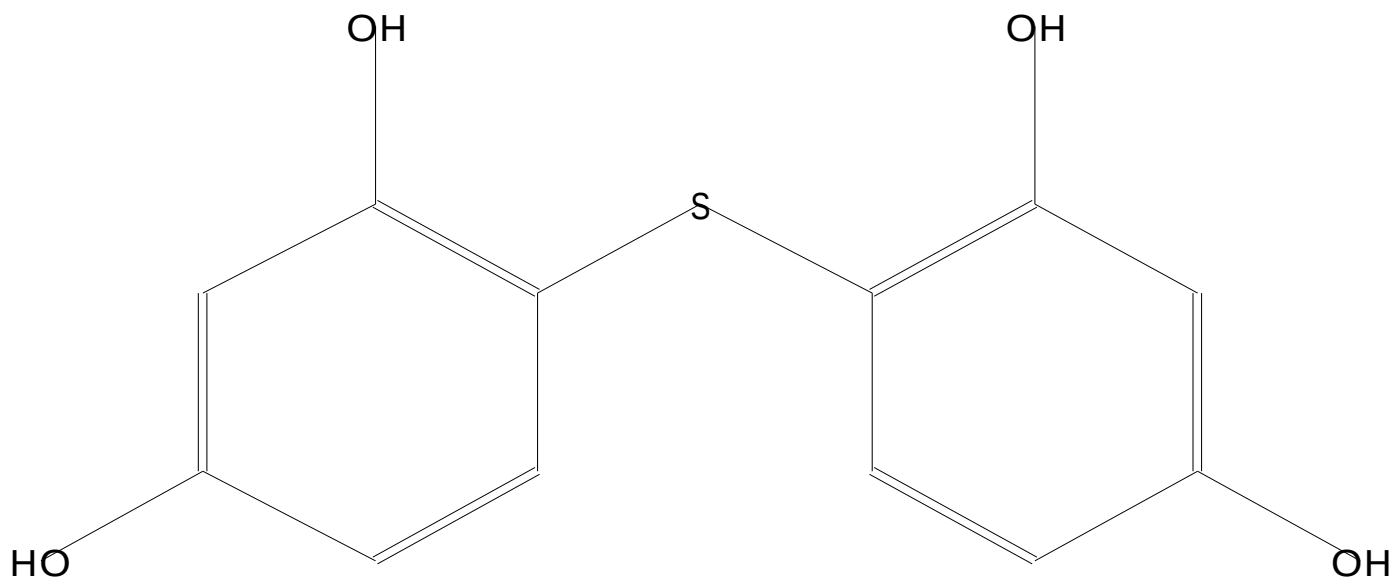

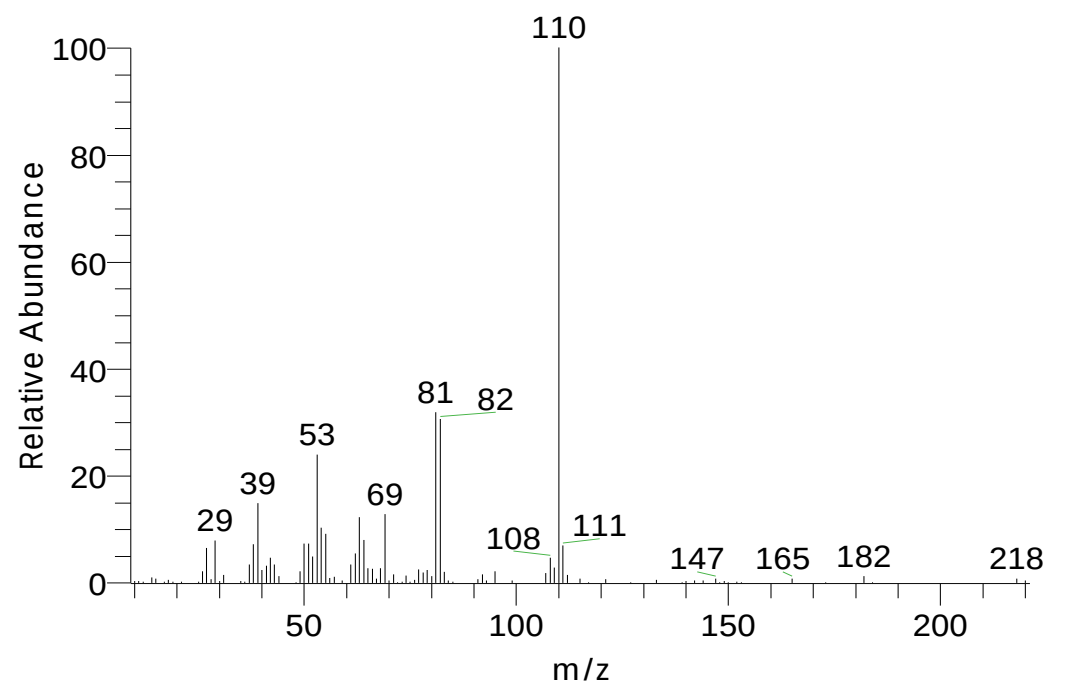

1,4-BENZENEDIOL
Formula C6H6O2, MW 110, CAS# 123-31-9, Entry# 8922
BENZENE-1,4-DIOL

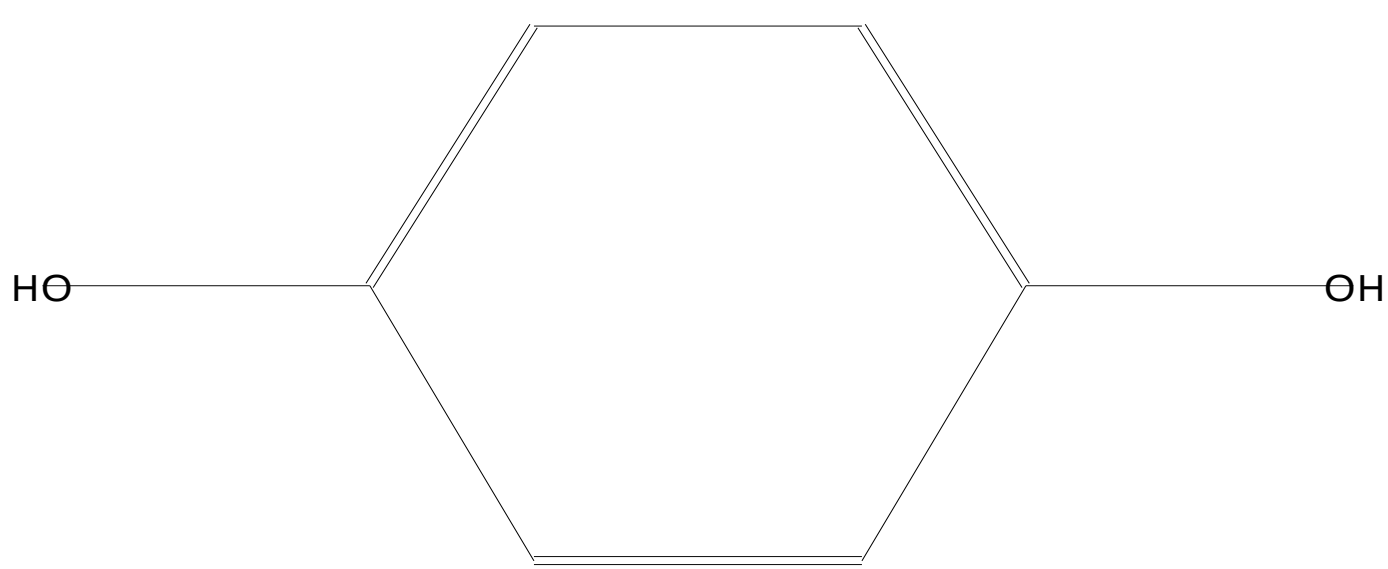

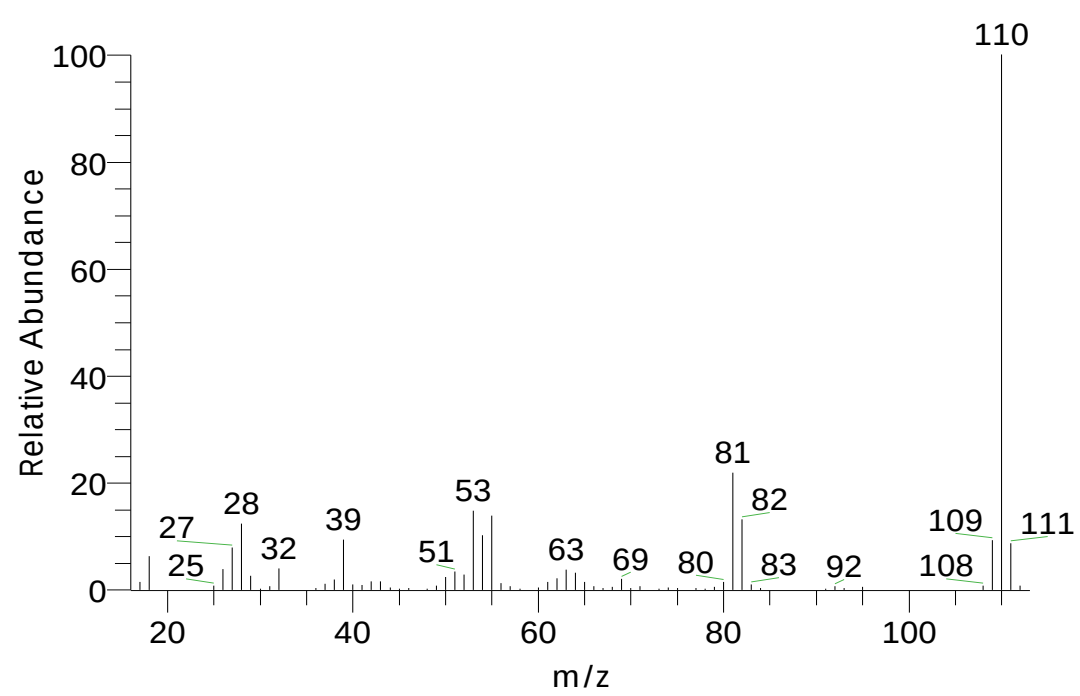

SI 614, RSI 817, mainlib, Entry# 87195, CAS# 123-31-9, Hydroquinone

Hydroquinone
Formula C6H6O2, MW 110, CAS# 123-31-9, Entry# 87195
1,4-Benzenediol

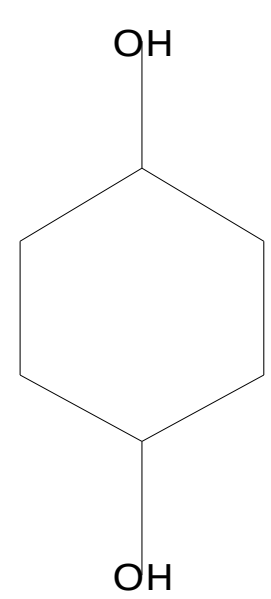

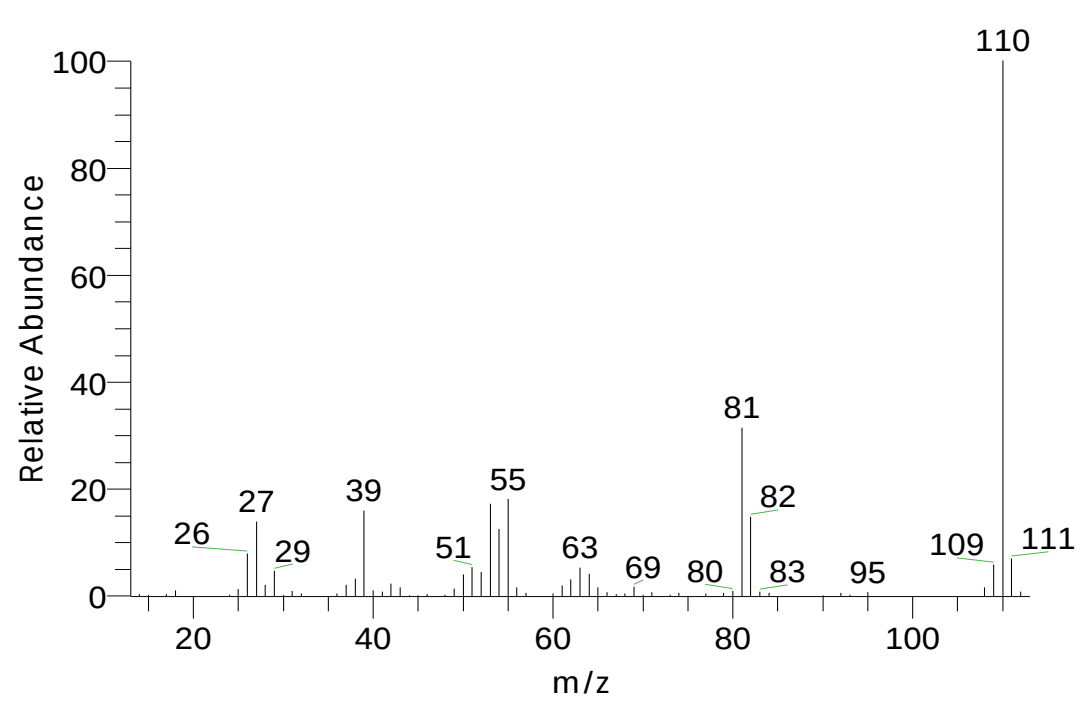

My GC-MS Report

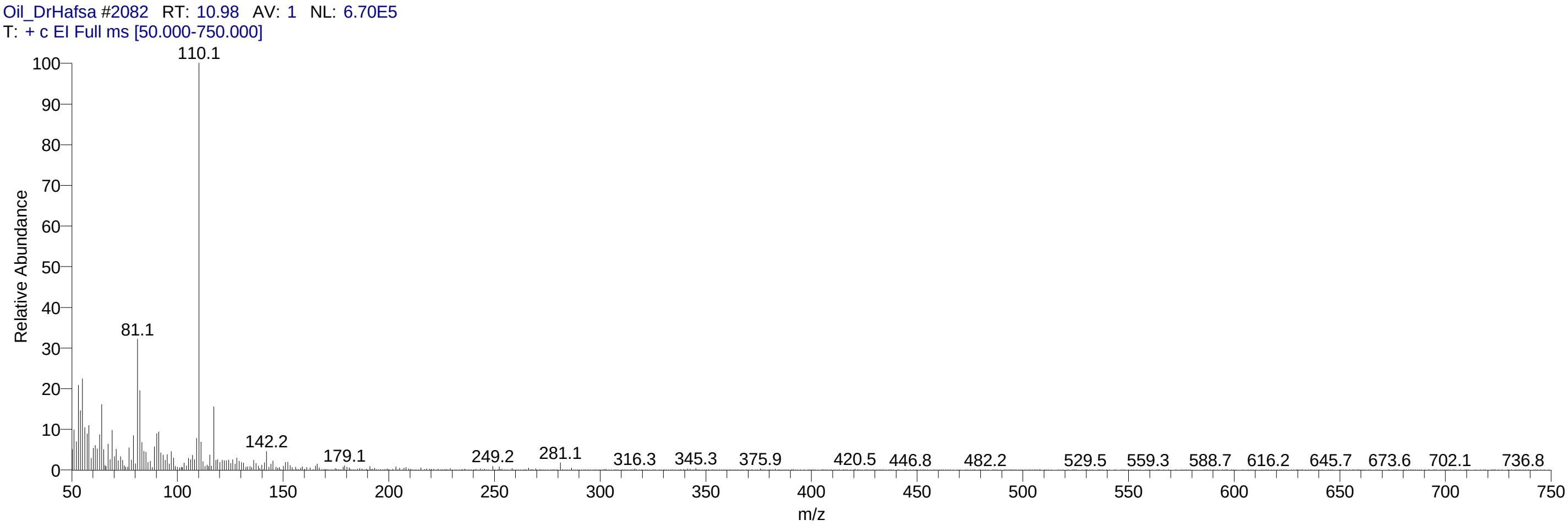

RT	Compound Name	Area %	MF	Molecular Formula	Molecular Weight	Cas #	Library
10.98	1,4-BENZENEDIOL	0.26	766	C6H6O2	110	123-31-9	WileyRegistry8e
10.98	HYDROCHINON	0.26	905	C6H6O2	110	NA	WileyRegistry8e
10.98	Hydroquinone	0.26	816	C6H6O2	110	123-31-9	mainlib
10.98	1,4-BENZENEDIOL	0.26	793	C6H6O2	110	123-31-9	WileyRegistry8e
10.98	1,3-BENZENEDIOL	0.26	733	C6H6O2	110	108-46-3	WileyRegistry8e

Compound Structure | Hit Spectrum

1,4-BENZENEDIOL
Formula C6H6O2, MW 110, CAS# 123-31-9, Entry# 8921
BENZENE-1,4-DIOL

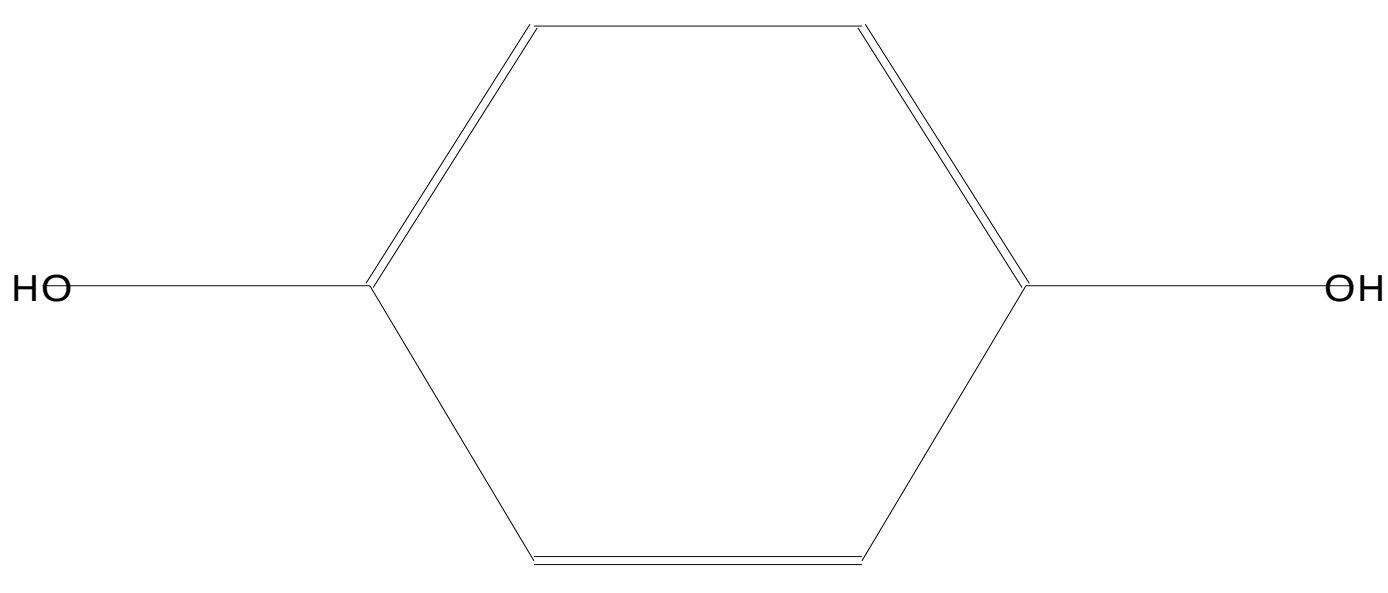

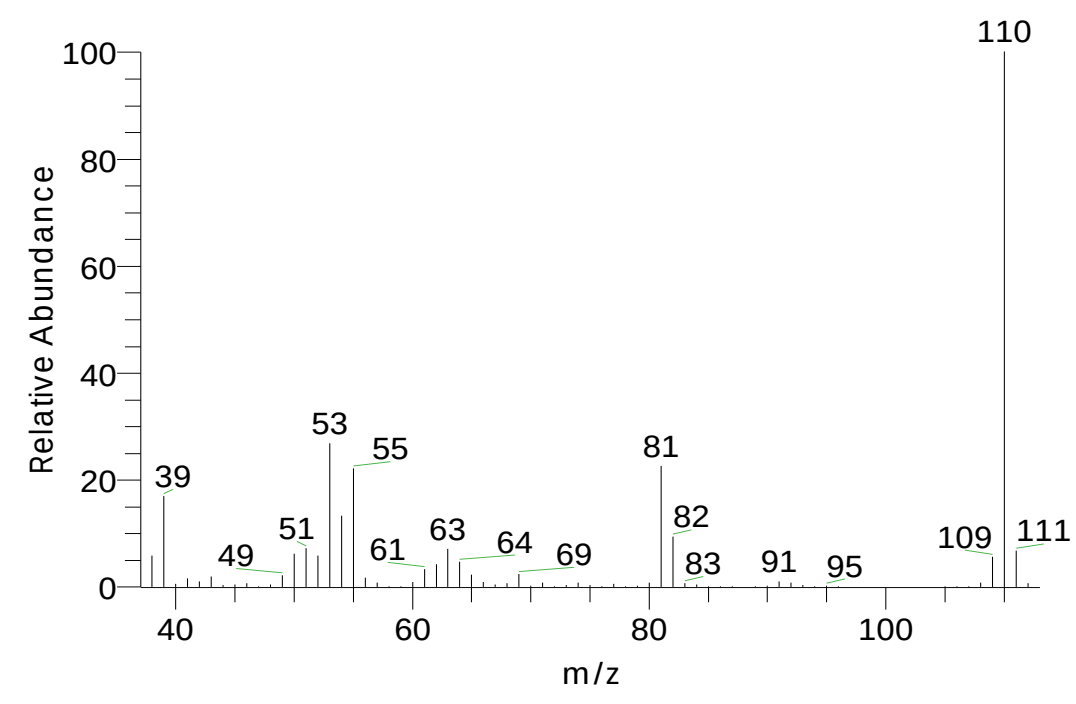

HYDROCHINON
Formula C6H6O2, MW 110, CAS# NA, Entry# 359374
HYDROQUINONE

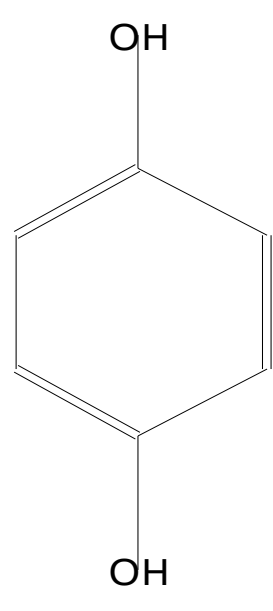

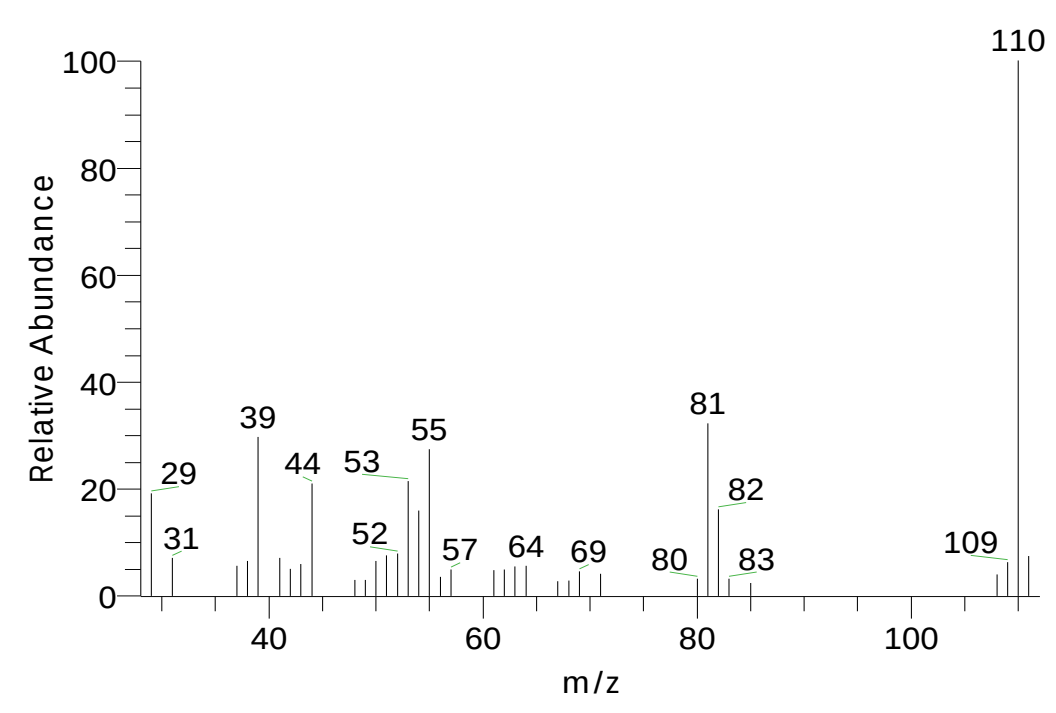

My GC-MS Report

Compound Structure | Hit Spectrum

Hydroquinone
Formula C6H6O2, MW 110, CAS# 123-31-9, Entry# 87195
1,4-Benzenediol

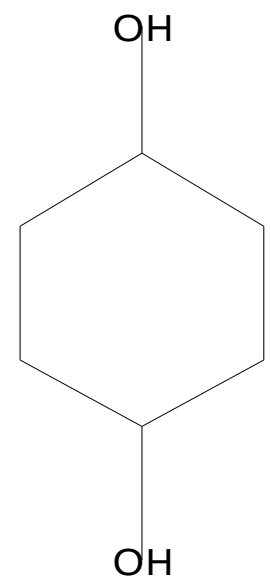

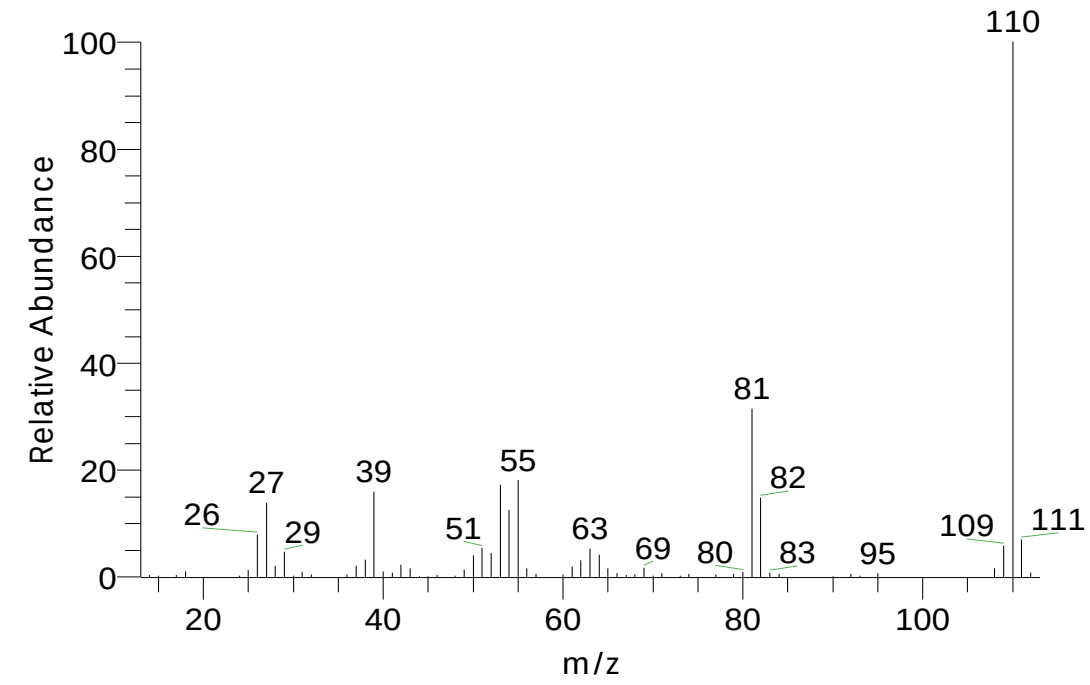

1,4-BENZENEDIOL
Formula C6H6O2, MW 110, CAS# 123-31-9, Entry# 8922
BENZENE-1,4-DIOL

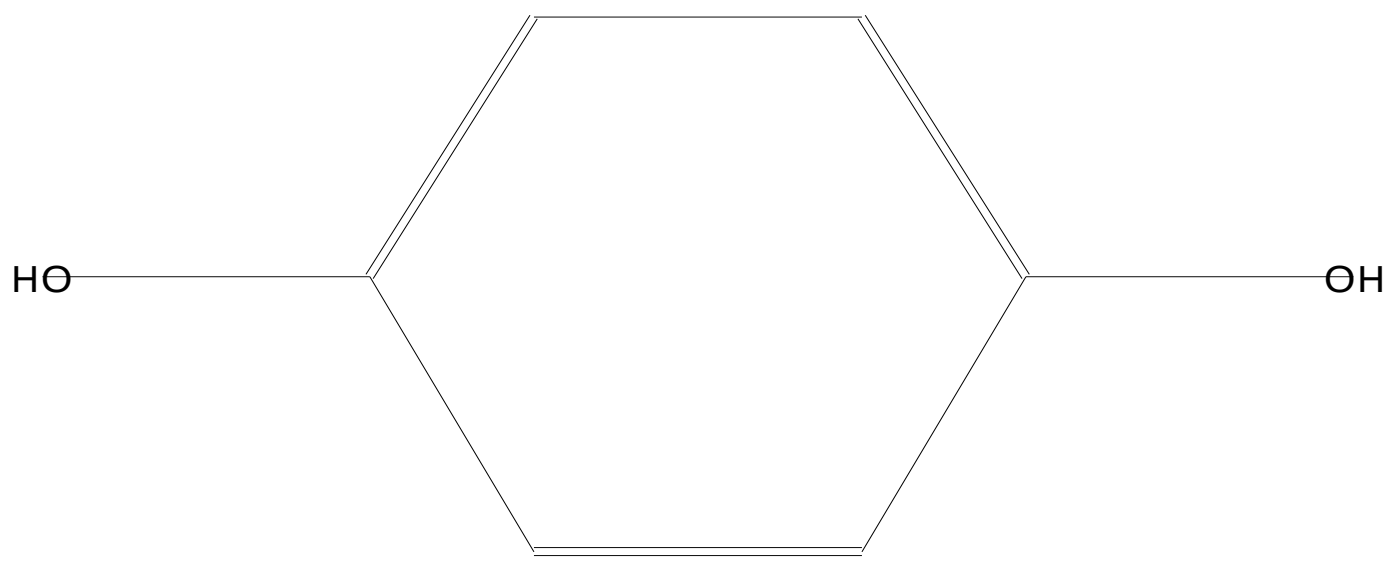

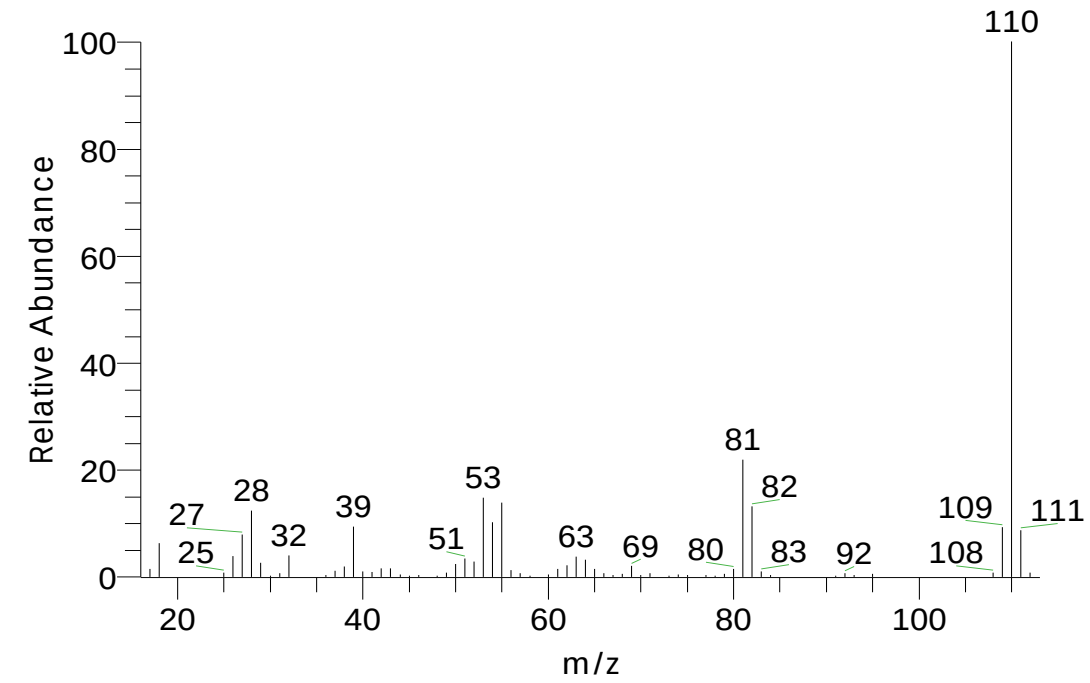

1,3-BENZENEDIOL
Formula C6H6O2, MW 110, CAS# 108-46-3, Entry# 8915
à-RESORCINOL

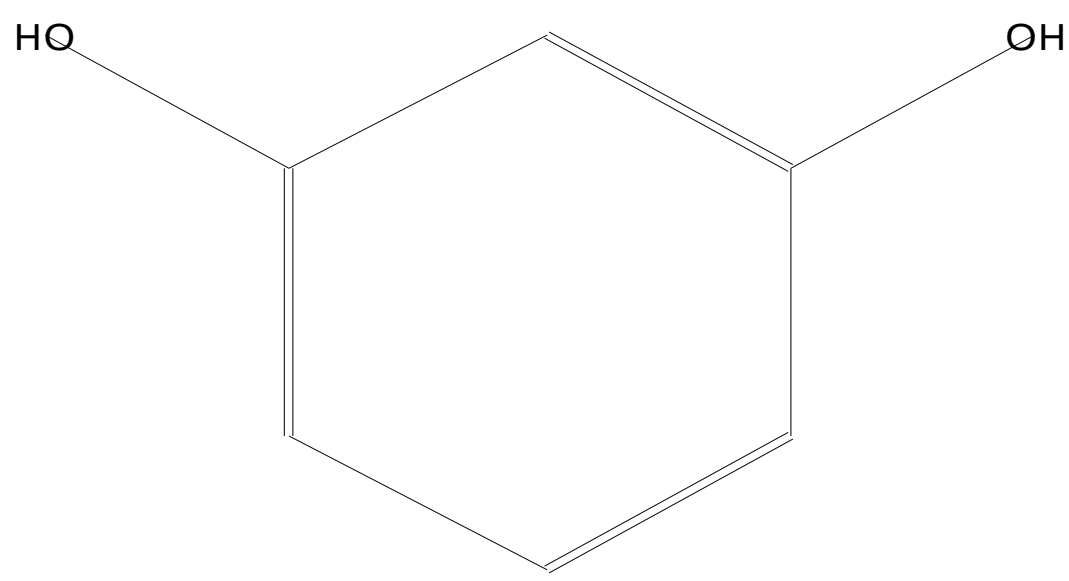

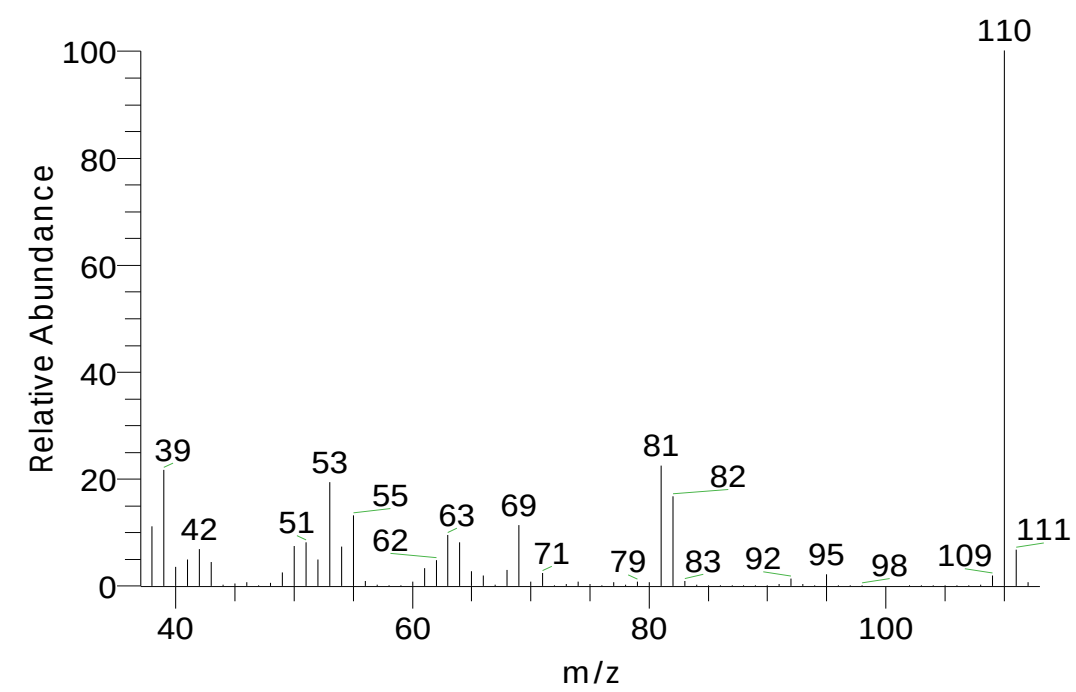

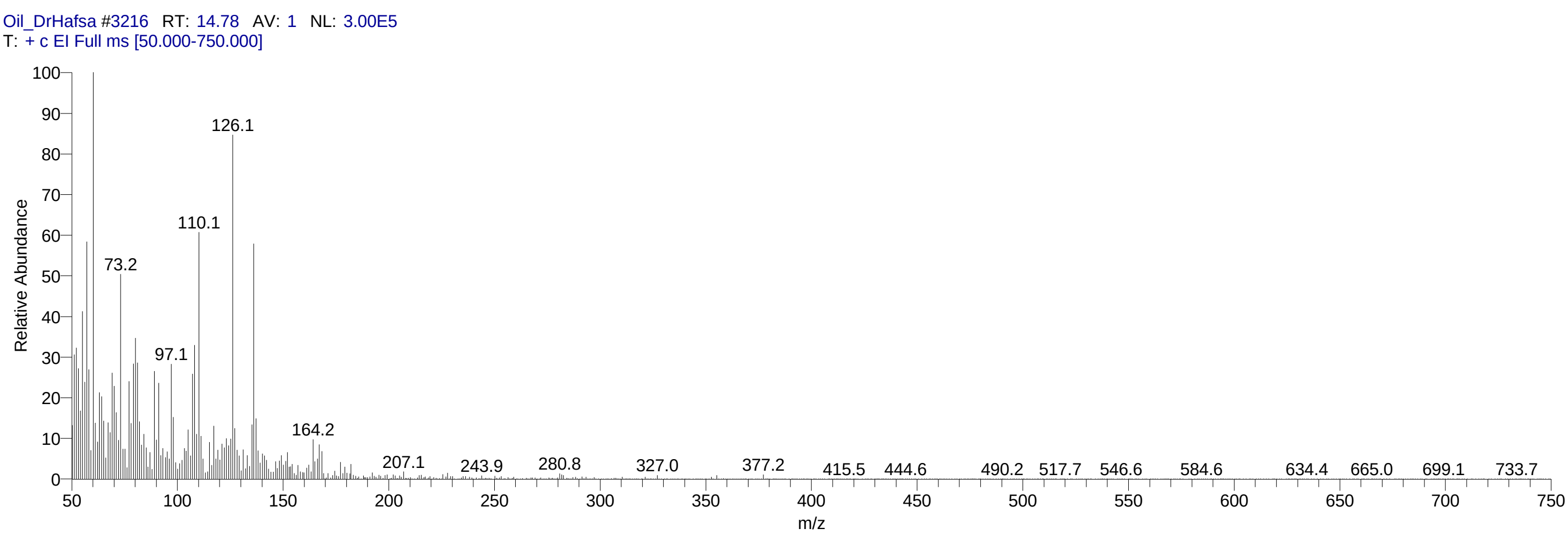

RT	Compound Name	Area %	MF	Molecular Formula	Molecular Weight	Cas #	Library
14.78	2-AMINOETHANETHIOL HYDROGEN SULFATE (ESTER)	0.68	717	C2H7NO3S2	157	2937-53-3	WileyRegistry8e
14.78	3-O-HEXOPYRANOSYLHEX-2-ULOFURANOSYL HEXOPYRANOSIDE #	0.68	651	C18H32O16	504	597-12-6	WileyRegistry8e

My GC-MS Report

RT	Compound Name	Area %	MF	Molecular Formula	Molecular Weight	Cas #	Library
14.78	Melezitose	0.68	650	C18H32O16	504	597-12-6	mainlib
14.78	Desulphosinigrin	0.68	653	C10H17NO6S	279	5115-81-1	mainlib
14.78	á-D-GLUCOPYRANOSE, 1-THIO-, 1-(N-HYDROXY-3-BUTENIMIDATE)	0.68	653	C10H17NO6S	279	5115-81-1	WileyRegistry8e

Compound Structure | Hit Spectrum

2-AMINOETHANETHIOL HYDROGEN SULFATE (ESTER)
Formula C2H7NO3S2, MW 157, CAS# 2937-53-3, Entry# 41029
2-AMINOETHANETHIOLSULFURIC ACID

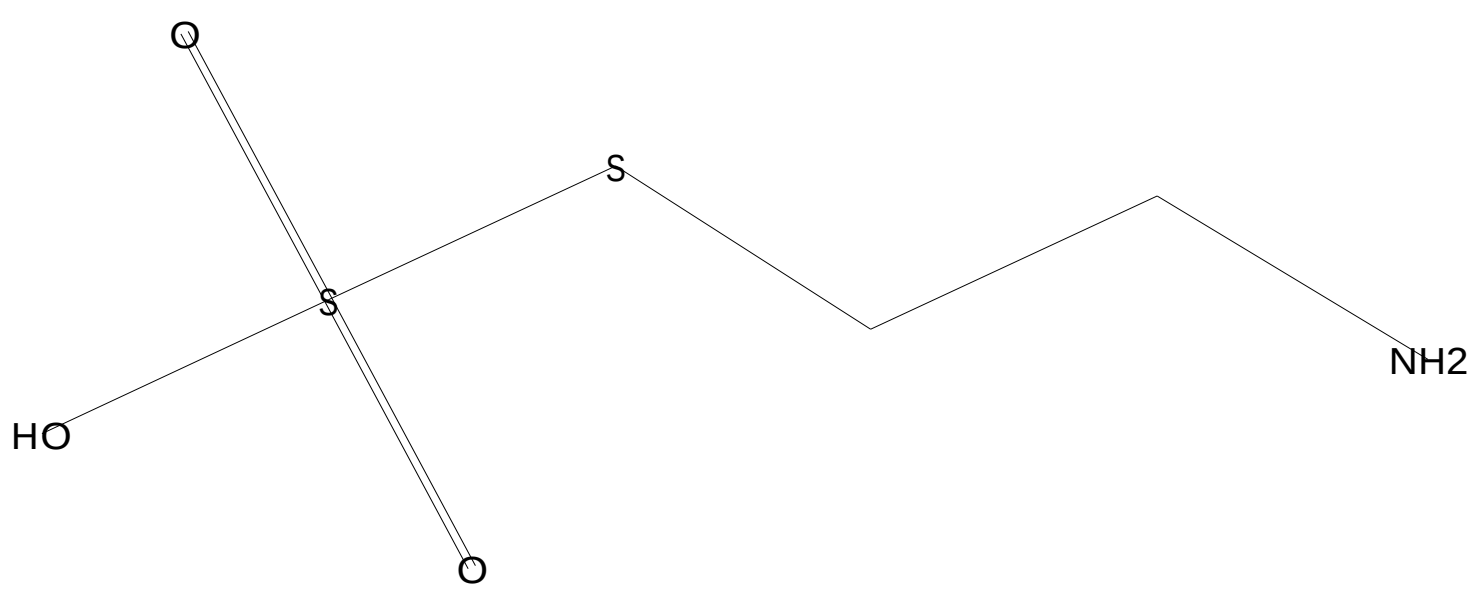

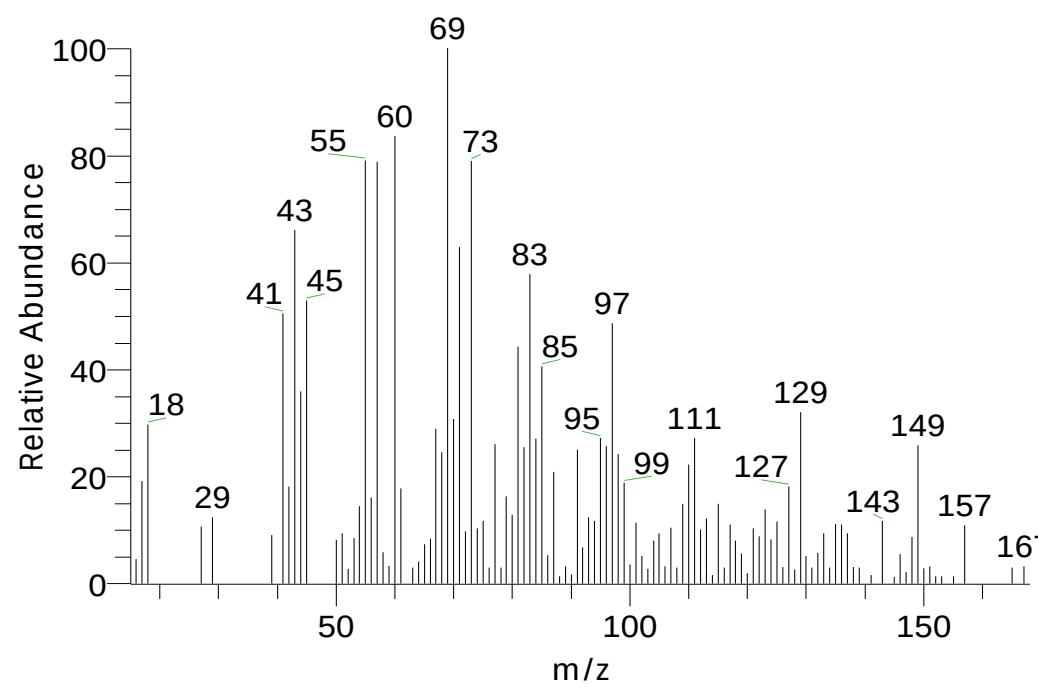

3-O-HEXOPYRANOSYLHEX-2-ULOFURANOSYL HEXOPYRANOSIDE #
Formula C18H32O16, MW 504, CAS# 597-12-6, Entry# 286042
à-D-GLUCOPYRANOSIDE, O-à-D-GLUCOPYRANOSYL-(1.->.3)-á-D-FRUCTOFURANOSYL

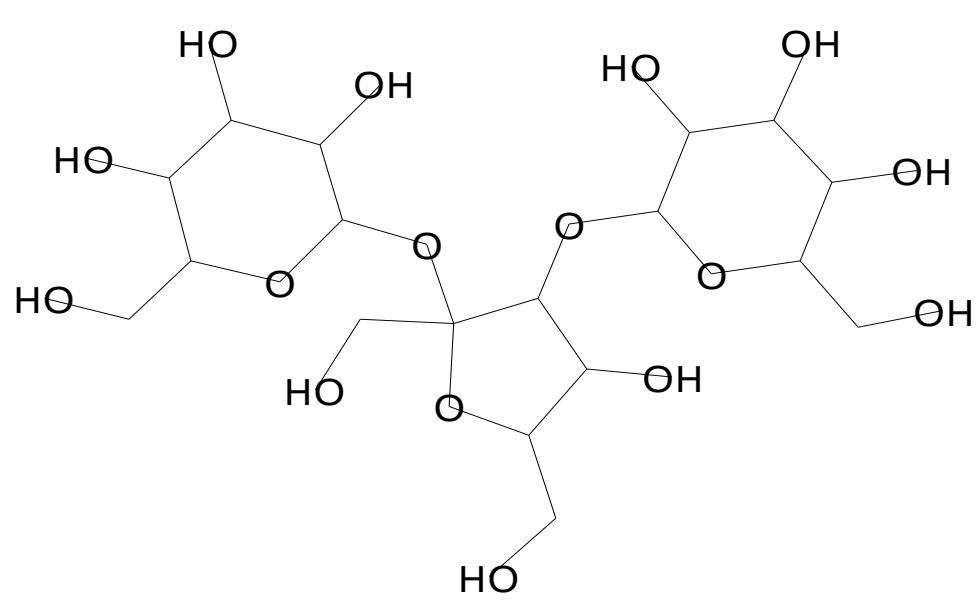

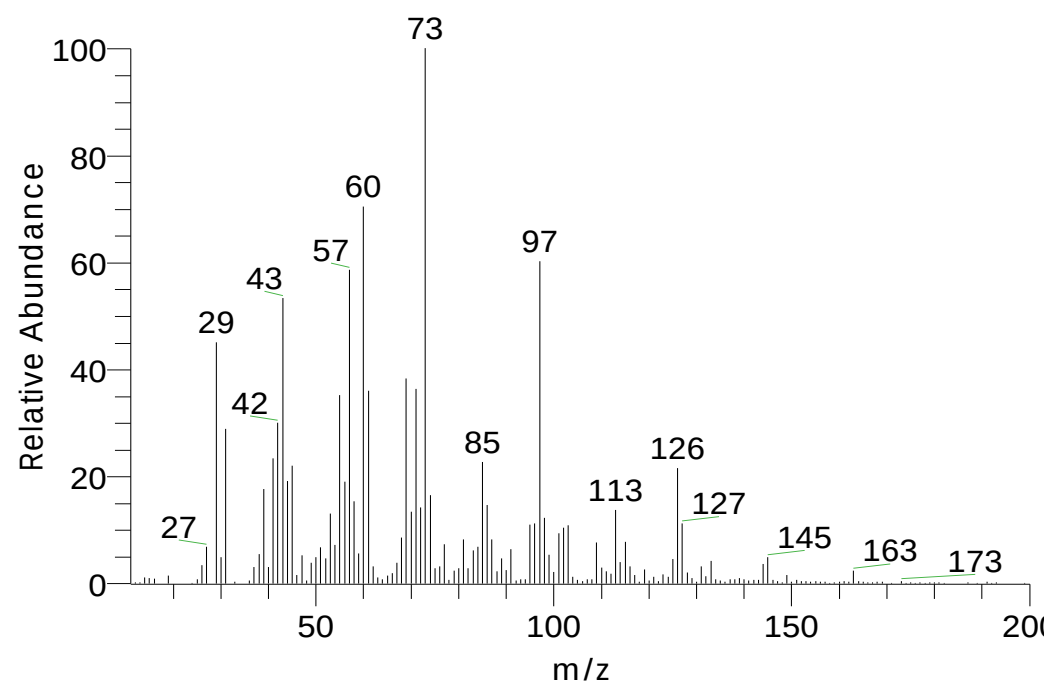

Melezitose
Formula C18H32O16, MW 504, CAS# 597-12-6, Entry# 41139
à-D-Glucopyranoside, O-à-D-glucopyranosyl-(1.fwdarw.3)-á-D-fructofuranosyl

SI 631, RSI 650, mainlib, Entry# 41139, CAS# 597-12-6, Melezitose

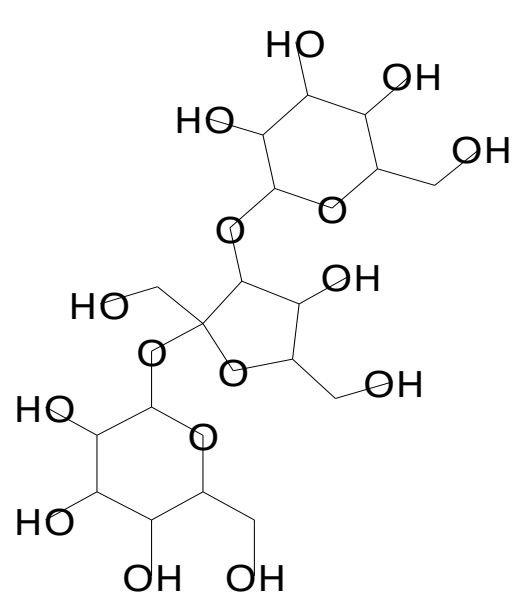

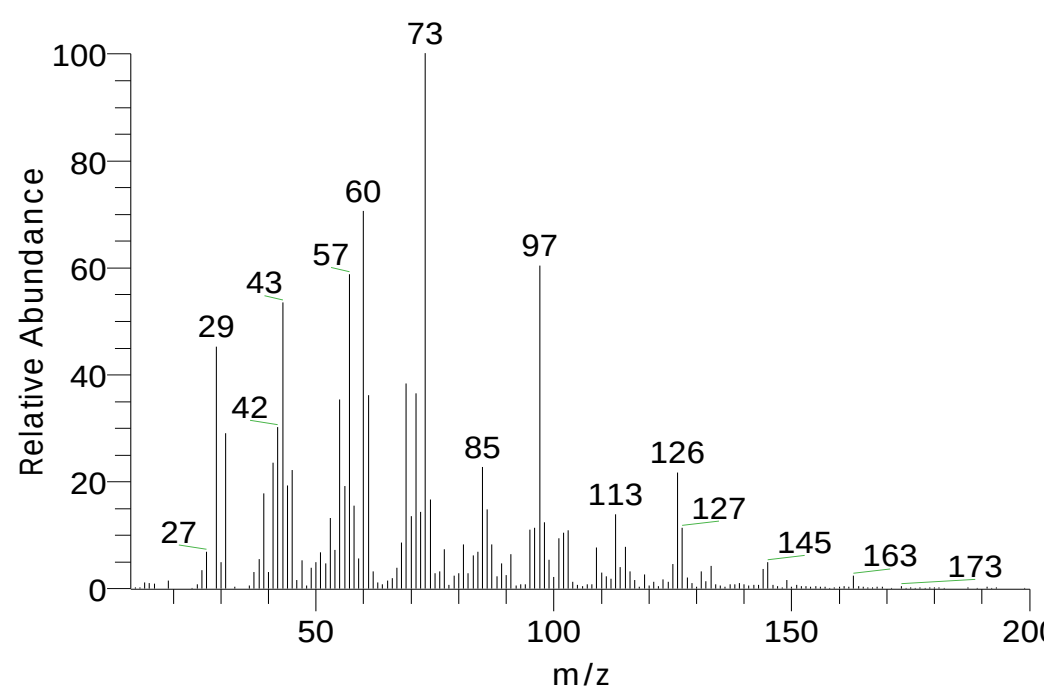

Desulphosinigrin
Formula C10H17NO6S, MW 279, CAS# 5115-81-1, Entry# 30961
1-S-[(1E)-N-Hydroxy-3-butenimidoyl]-1-thiohexopyranose #

SI 622, RSI 653, mainlib, Entry# 30961, CAS# 5115-81-1, Desulphosinigrin

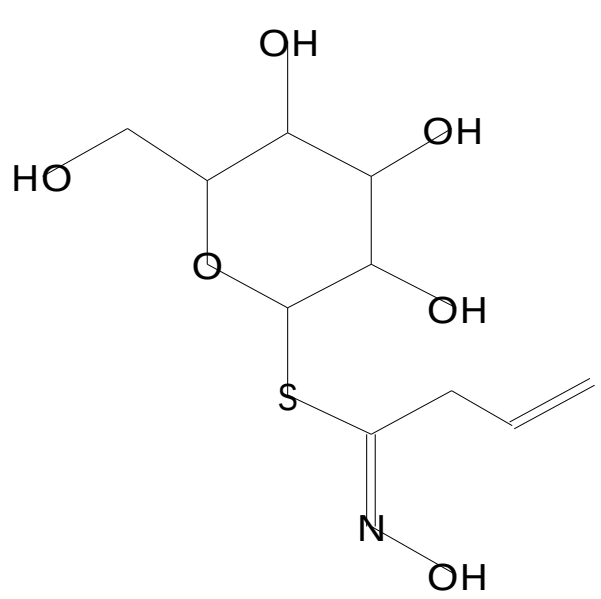

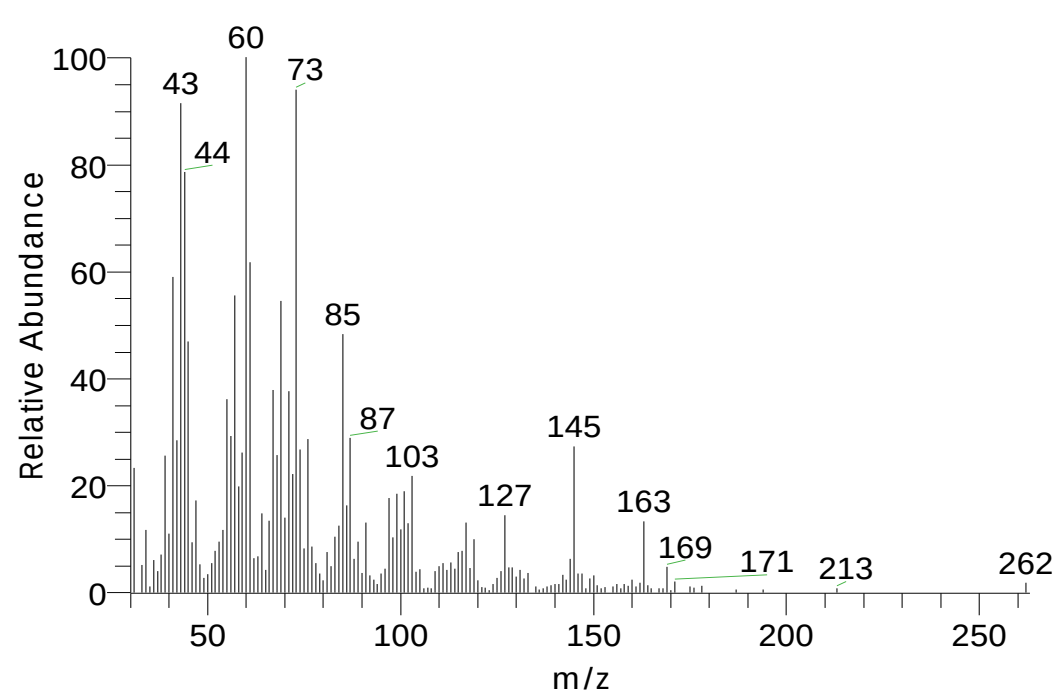

My GC-MS Report

Compound Structure | Hit Spectrum

á-D-GLUCOPYRANOSE, 1-THIO-, 1-(N-HYDROXY-3-BUTENIMIDATE)
Formula C10H17NO6S, MW 279, CAS# 5115-81-1, Entry# 169302
GLUCOPYRANOSE, 1-THIO-, 1-(3-BUTENOATE), OXIME

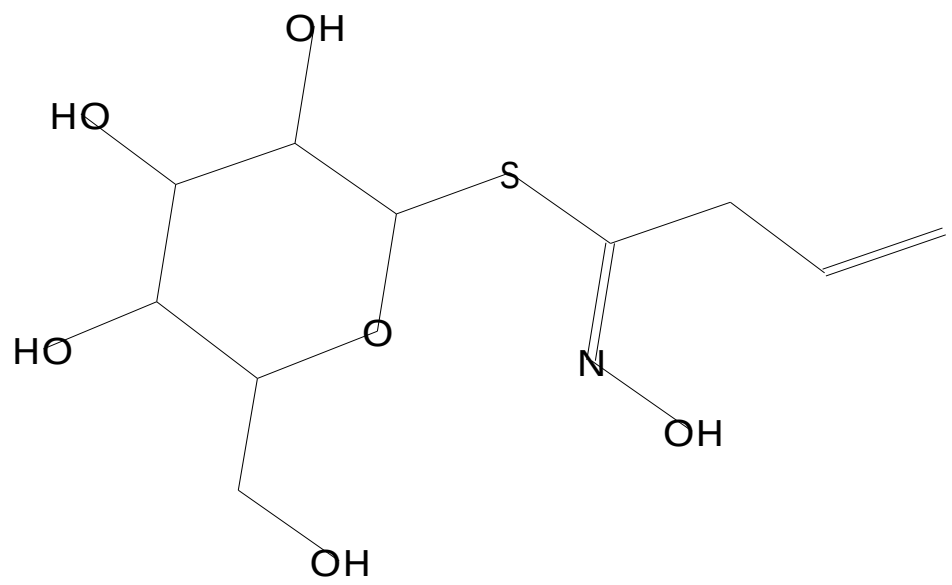

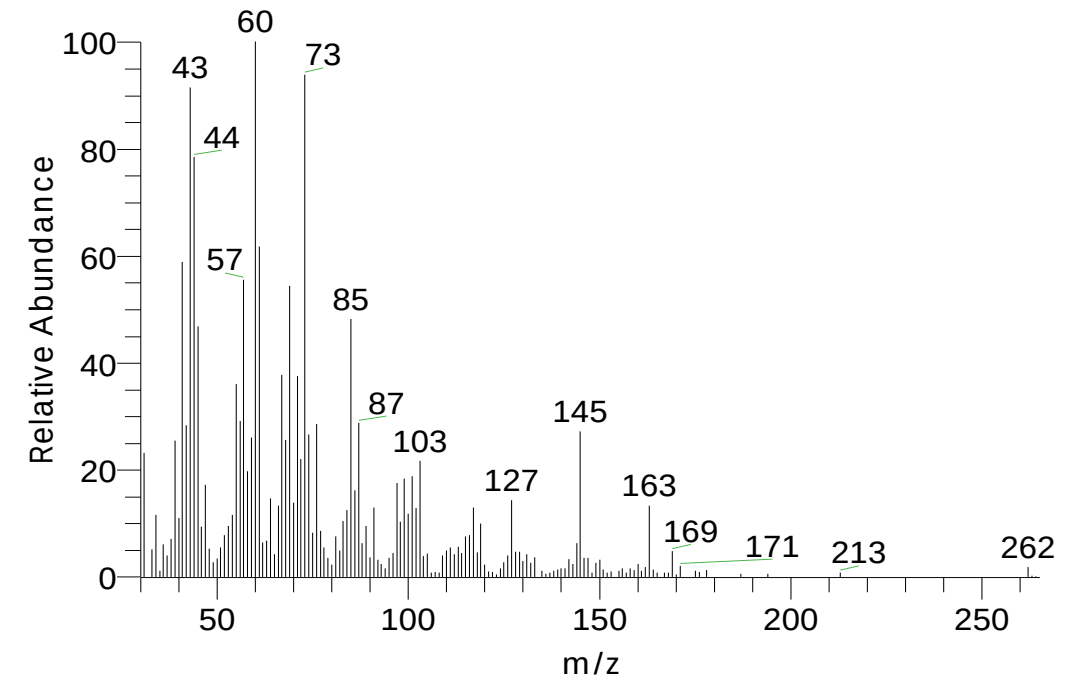

Oil_DrHafsa #5116 RT: 21.15 AV: 1 NL: 1.78E5
T: + c EI Full ms [50.000-750.000]

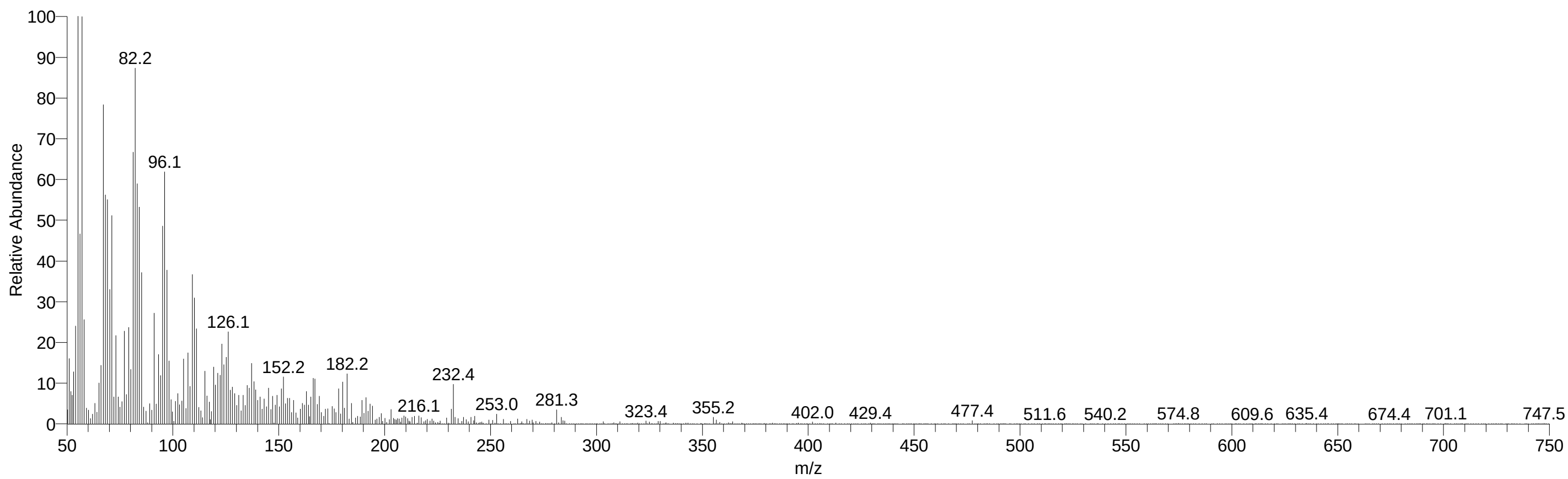

RT	Compound Name	Area %	MF	Molecular Formula	Molecular Weight	Cas #	Library
21.15	13-Heptadecyn-1-ol	0.21	767	C17H32O	252	56554-77-9	mainlib
21.15	13-HEPTADECYN-1-OL	0.21	767	C17H32O	252	56554-77-9	WileyRegistry8e
21.15	Z-(13,14-Epoxy)tetradec-11-en-1-ol acetate	0.21	757	C16H28O3	268	NA	mainlib
21.15	Ethanol, 2-(9-octadecenyloxy)-, (Z)-	0.21	737	C20H40O2	312	5353-25-3	mainlib
21.15	ETHANOL, 2-(9-OCTADECENYLOXY)-, (Z)-	0.21	736	C20H40O2	312	5353-25-3	WileyRegistry8e

Compound Structure | Hit Spectrum

13-Heptadecyn-1-ol
Formula C17H32O, MW 252, CAS# 56554-77-9, Entry# 51015
$:28DZJZIJZUKAATIV-UHFFFAOYSA-N

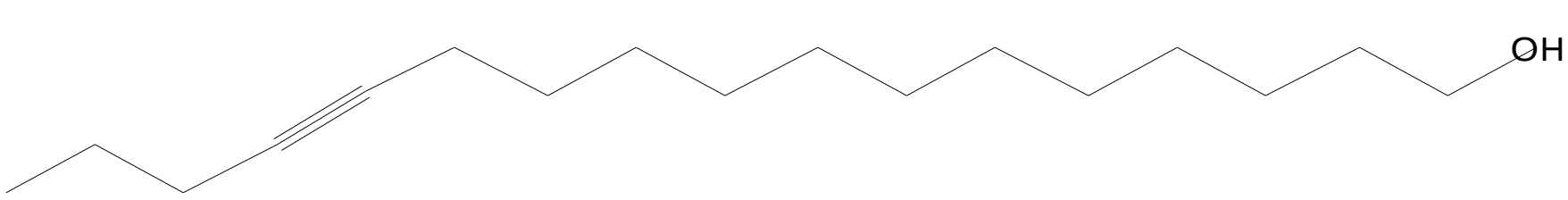

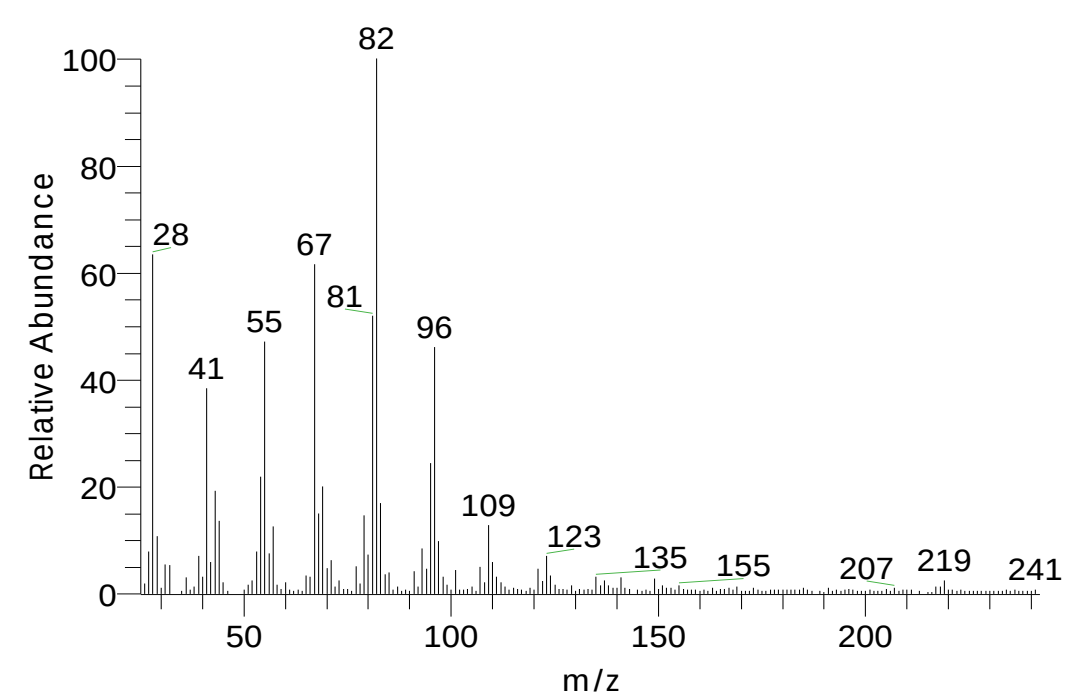

My GC-MS Report

Compound Structure | Hit Spectrum

13-HEPTADECYN-1-OL
Formula C17H32O, MW 252, CAS# 56554-77-9, Entry# 142927
1-HYDROXY HEPTADEC-13-YNE

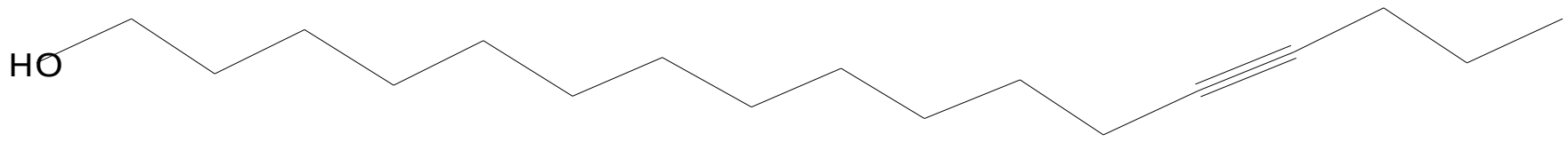

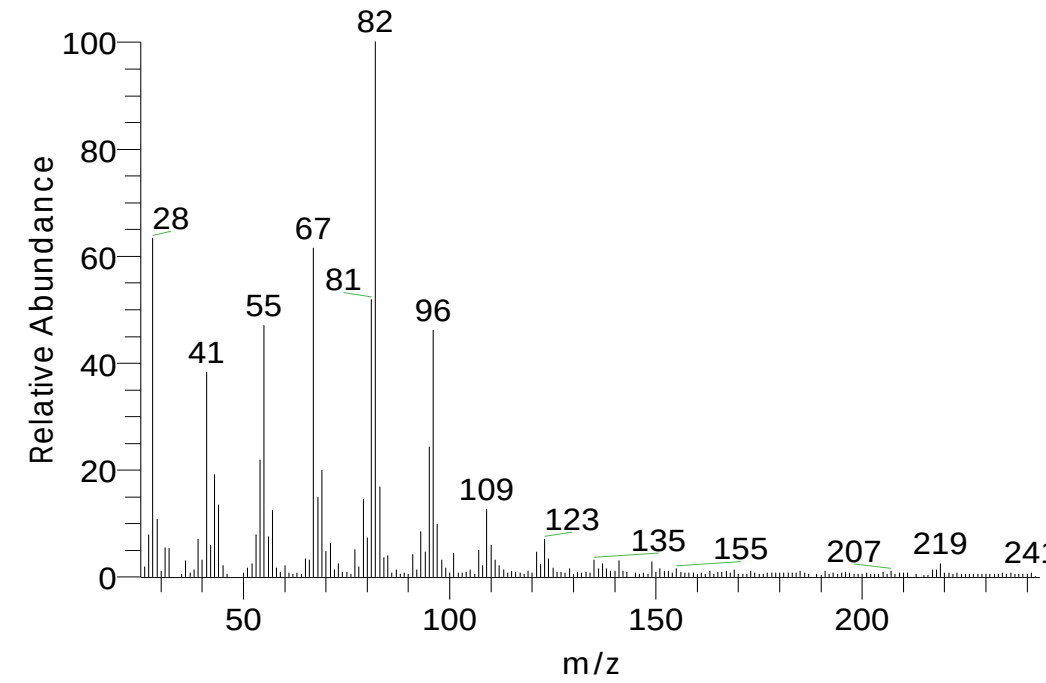

Z-(13,14-Epoxy)tetradec-11-en-1-ol acetate
Formula C16H28O3, MW 268, CAS# NA, Entry# 10459
(11Z)-12-(2-Oxiranyl)-11-dodecenyl acetate #

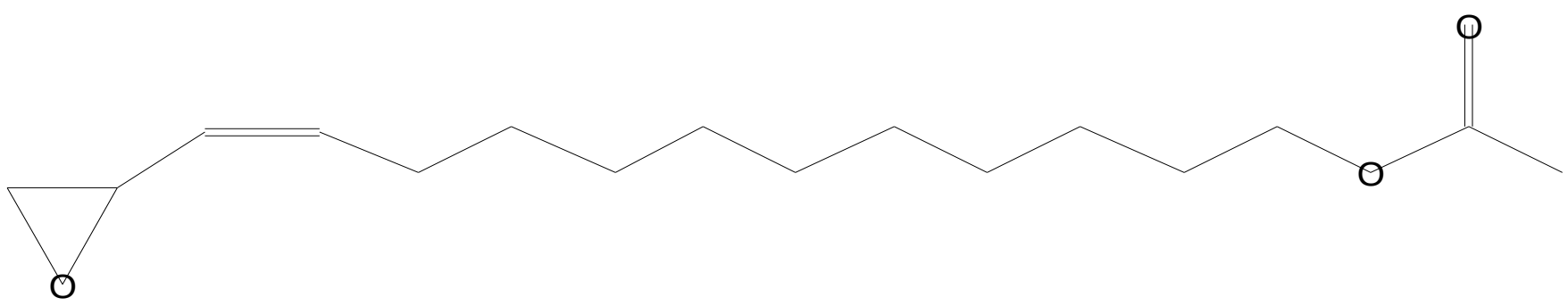

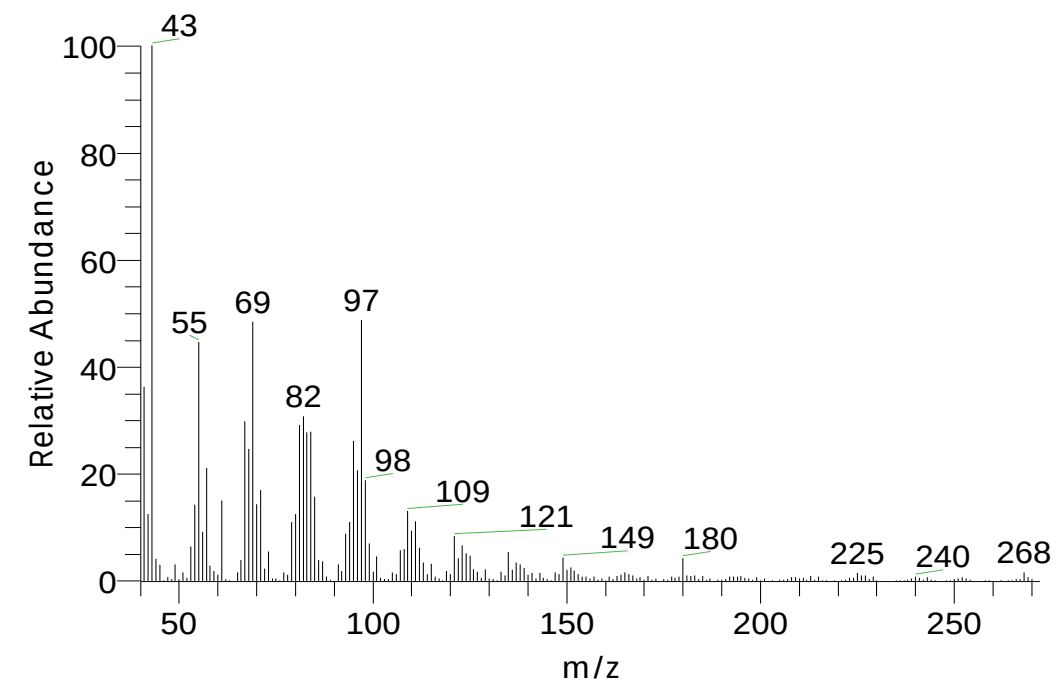

Ethanol, 2-(9-octadecenyloxy)-, (Z)-
Formula C20H40O2, MW 312, CAS# 5353-25-3, Entry# 20581
2-cis-9-Octadecenyloxyethanol

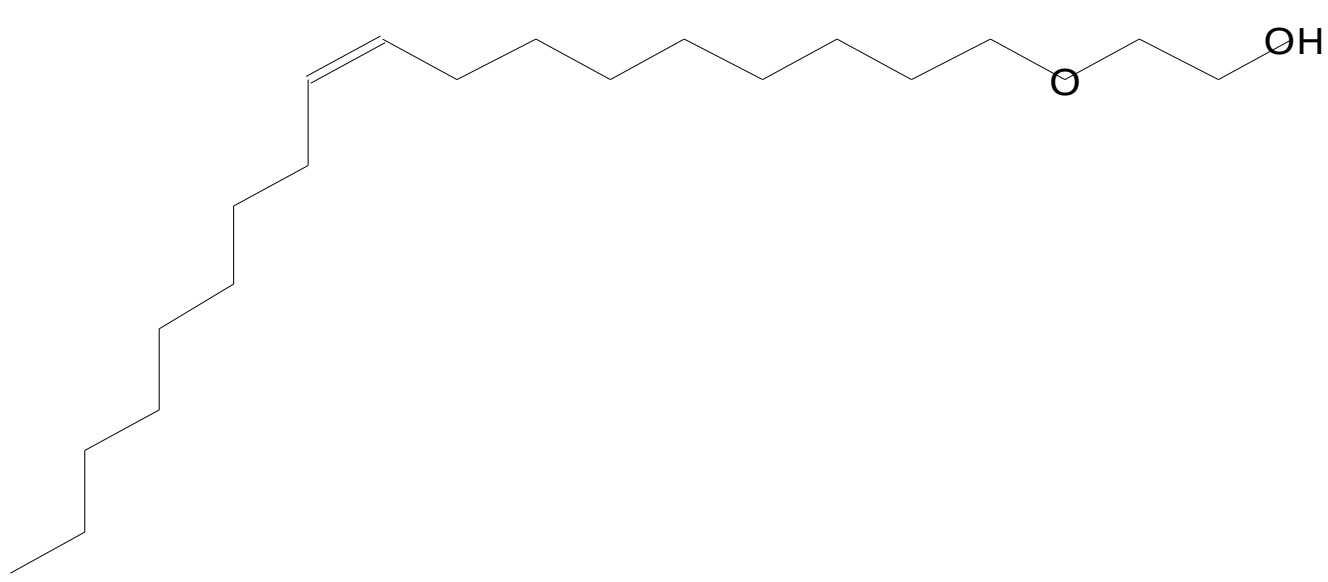

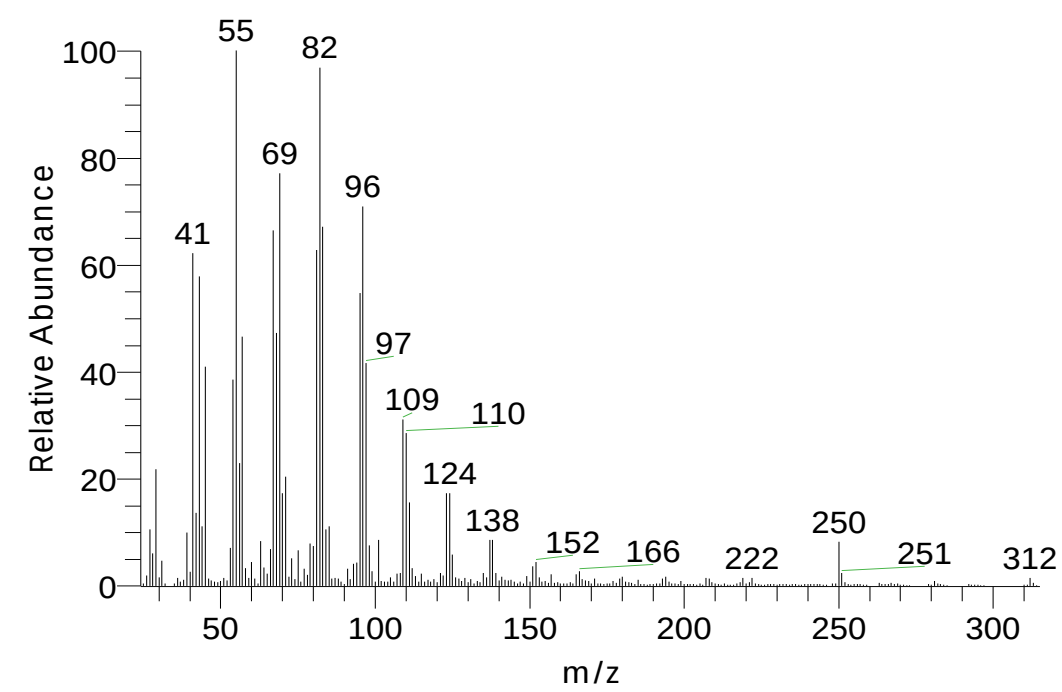

ETHANOL, 2-(9-OCTADECENYLOXY)-, (Z)-
Formula C20H40O2, MW 312, CAS# 5353-25-3, Entry# 200393
2-[(9Z)-9-OCTADECENYLOXY]ETHANOL #

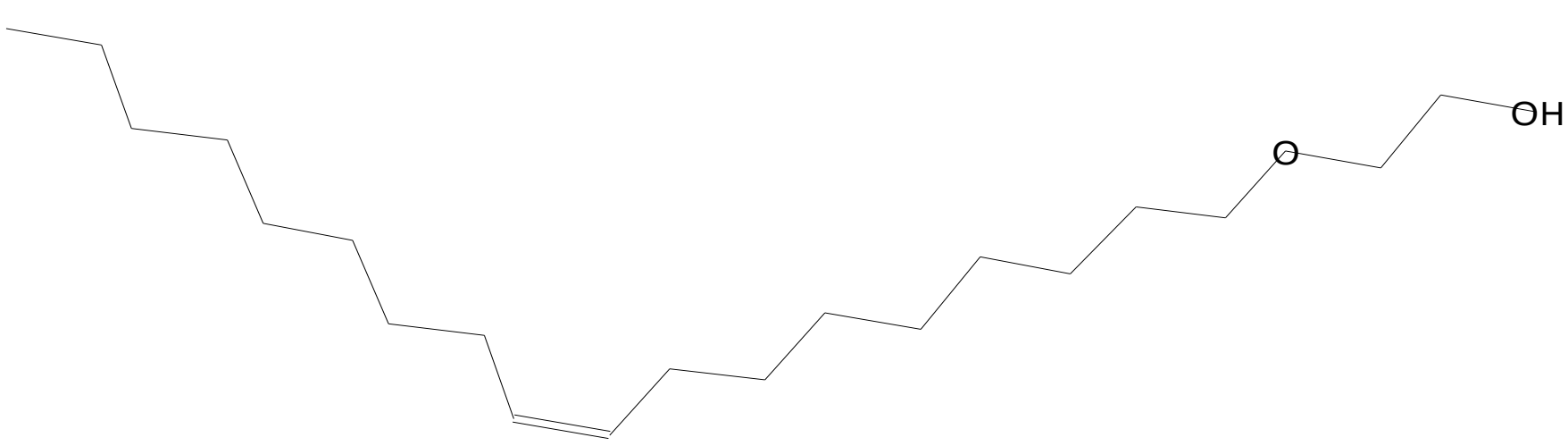

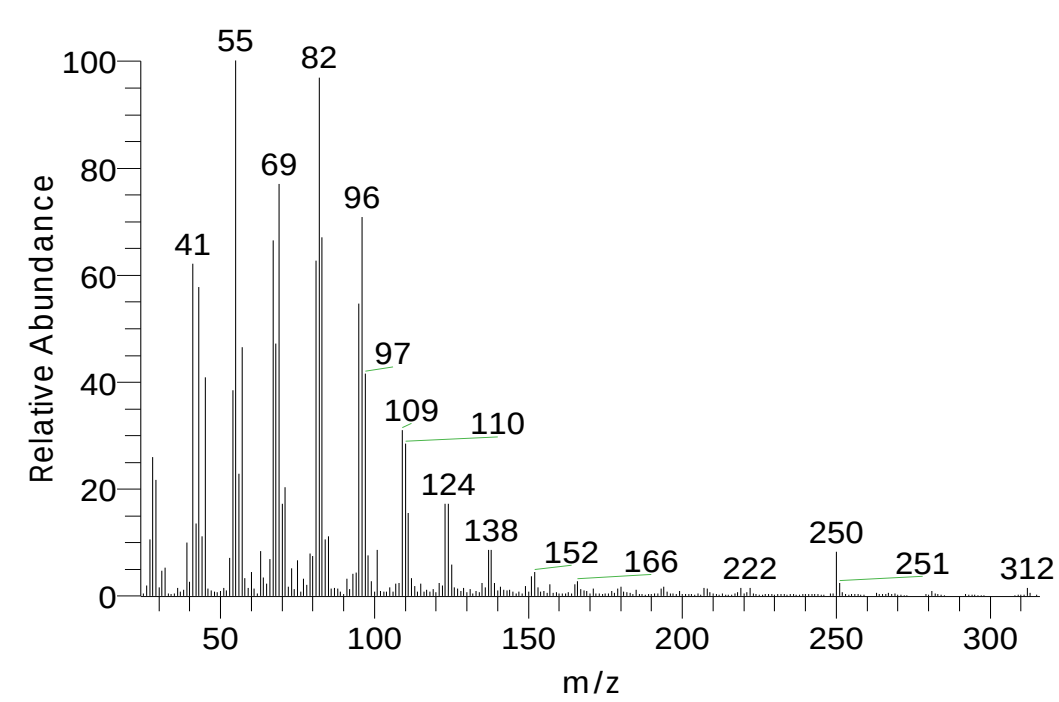

My GC-MS Report

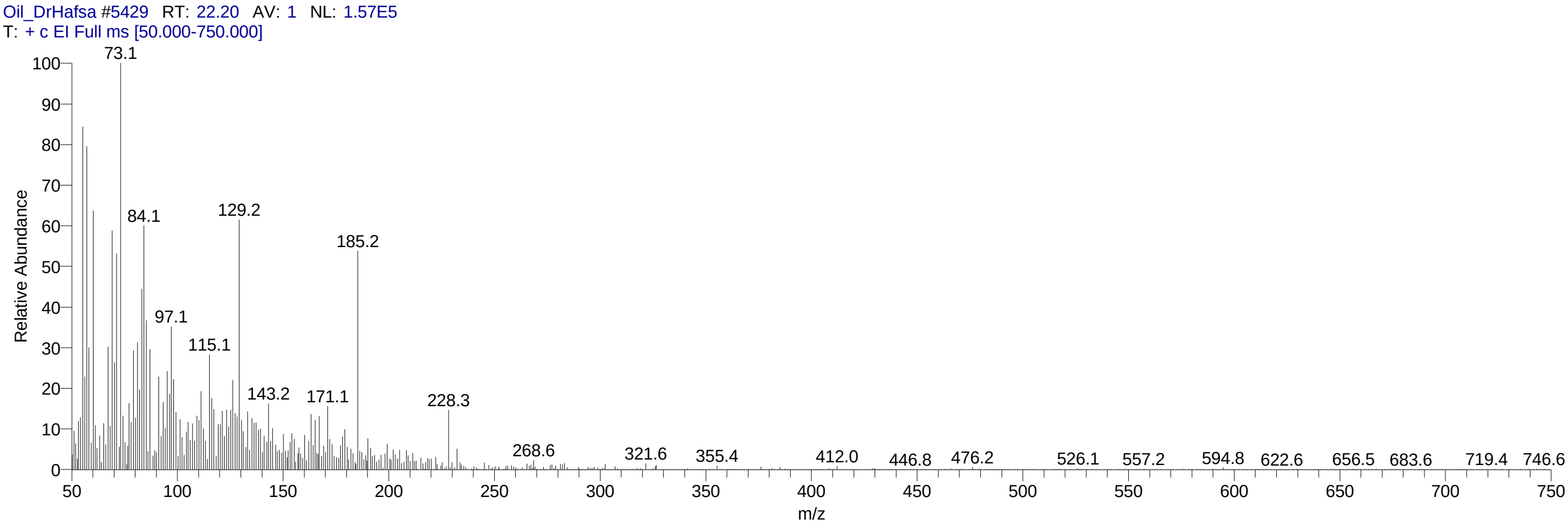

RT	Compound Name	Area %	MF	Molecular Formula	Molecular Weight	Cas #	Library
22.20	HEXADECANOIC ACID, 2,3-DIHYDROXYPROPYL ESTER	0.28	723	C19H38O4	330	542-44-9	WileyRegistry8e
22.20	TETRADECANOIC ACID	0.28	748	C14H28O2	228	544-63-8	WileyRegistry8e
22.20	PENTADECANOIC ACID	0.28	732	C15H30O2	242	1002-84-2	WileyRegistry8e
22.20	OCTADECANOIC ACID, 2,3-DIHYDROXYPROPYL ESTER	0.28	693	C21H42O4	358	123-94-4	WileyRegistry8e
22.20	HEXADECANOIC ACID, 2-HYDROXY-1,3-PROPANEDIYL ESTER	0.28	689	C35H68O5	568	502-52-3	WileyRegistry8e

Compound Structure	Hit Spectrum

HEXADECANOIC ACID, 2,3-DIHYDROXYPROPYL ESTER
Formula C19H38O4, MW 330, CAS# 542-44-9, Entry# 214589
2,3-DIHYDROXYPROPYL PALMITATE #

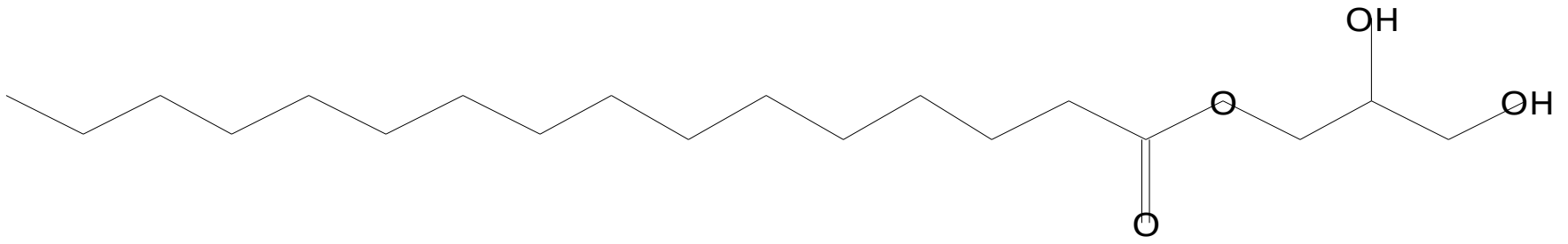

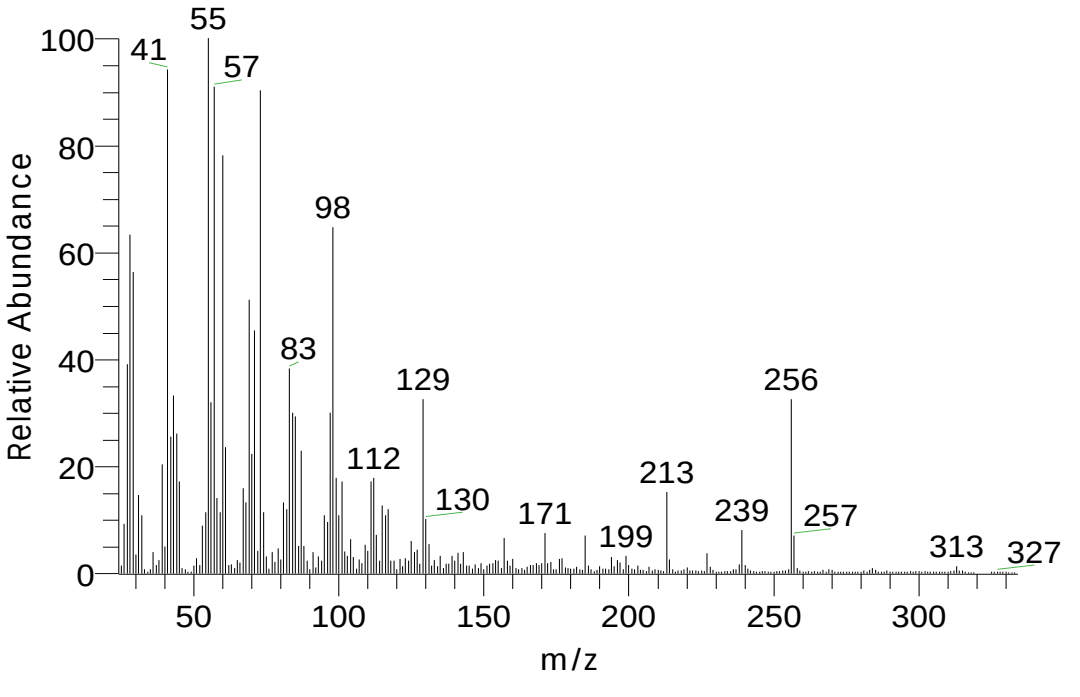

TETRADECANOIC ACID
Formula C14H28O2, MW 228, CAS# 544-63-8, Entry# 116584
METHYL TRIDECANOATE

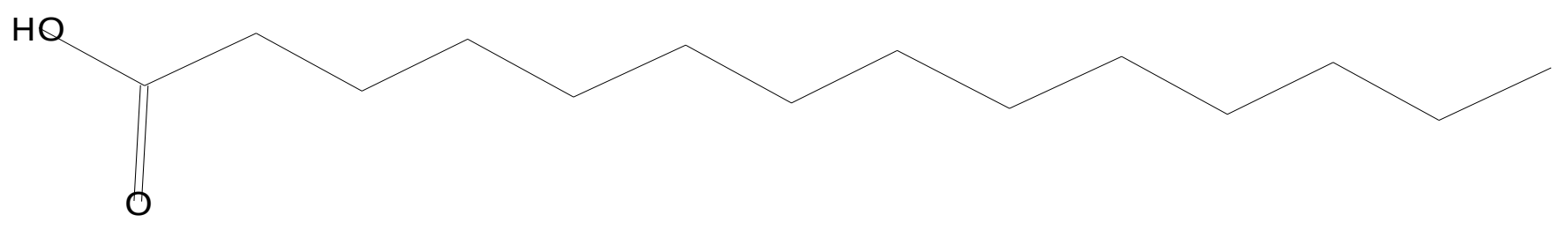

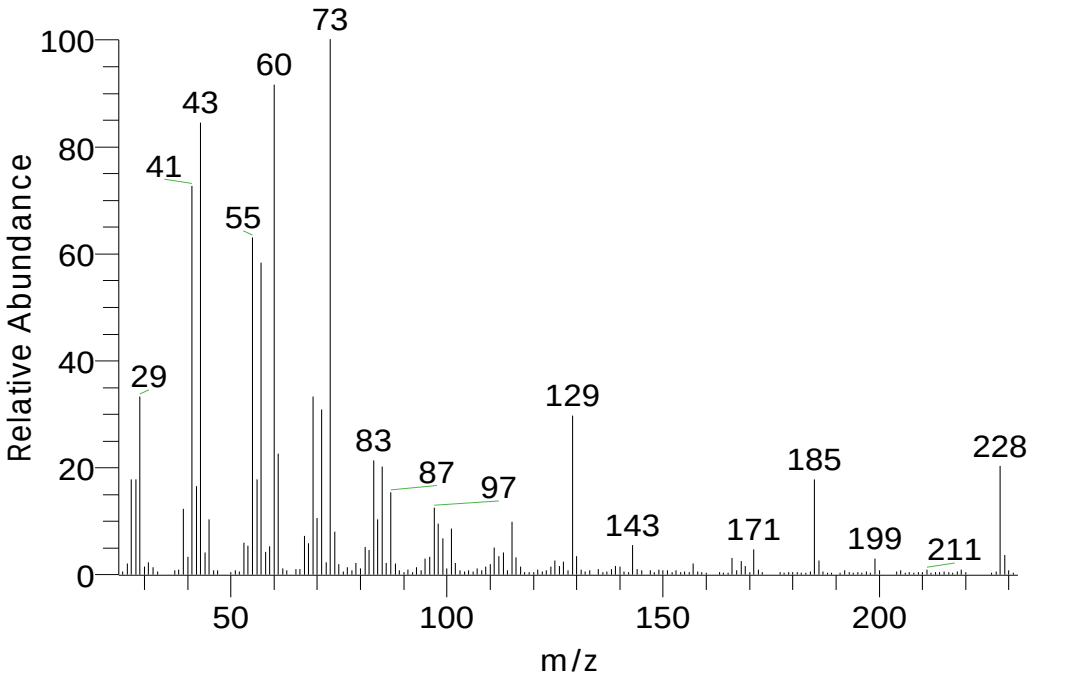

My GC-MS Report

Compound Structure	Hit Spectrum

PENTADECANOIC ACID
Formula C15H30O2, MW 242, CAS# 1002-84-2, Entry# 131990
14FA

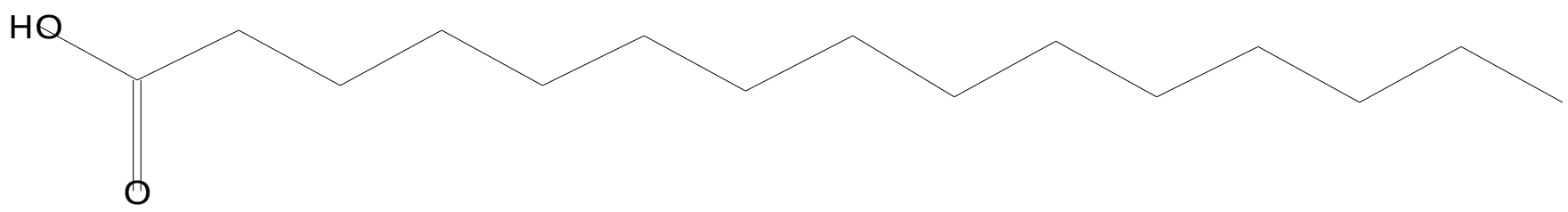

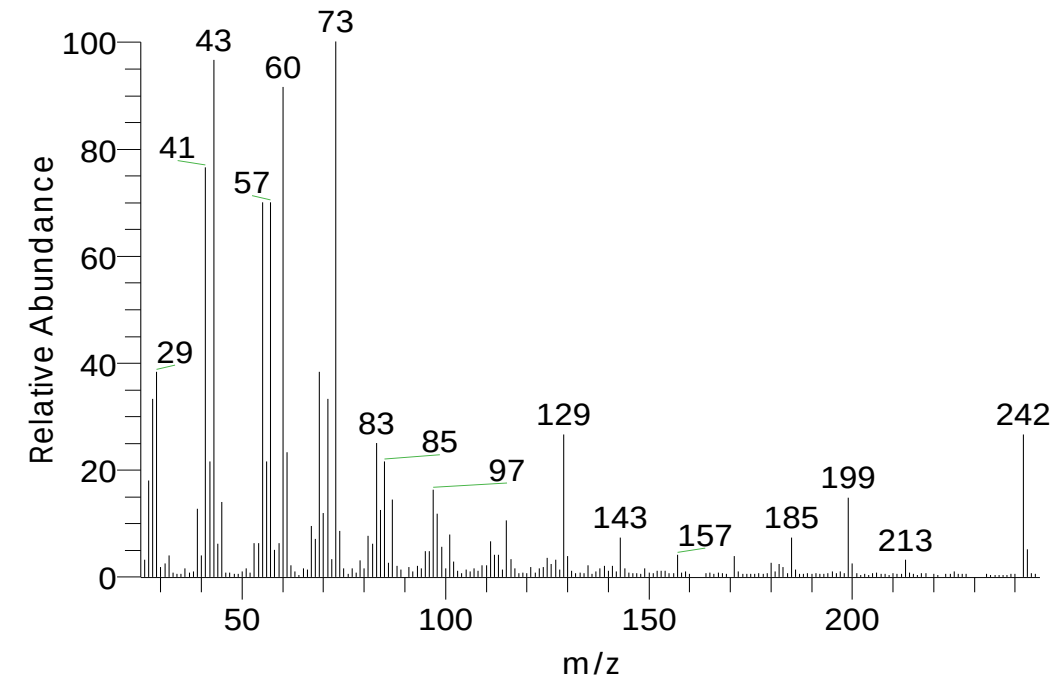

OCTADECANOIC ACID, 2,3-DIHYDROXYPROPYL ESTER
Formula C21H42O4, MW 358, CAS# 123-94-4, Entry# 233812
2,3-DIHYDROXYPROPYL STEARATE #

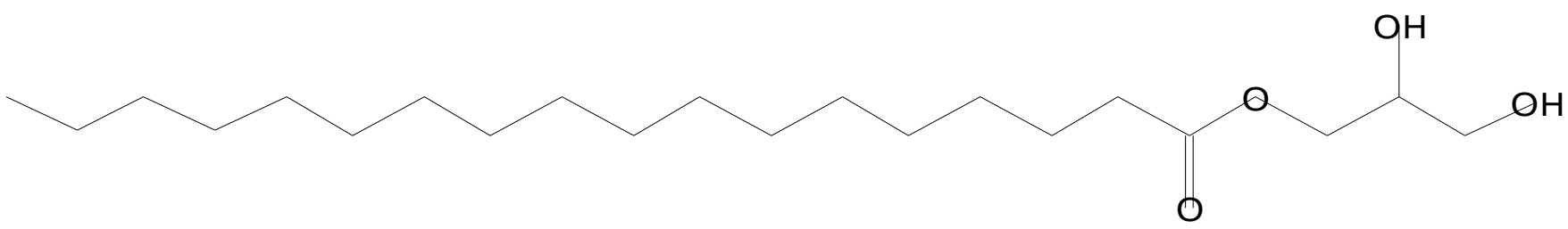

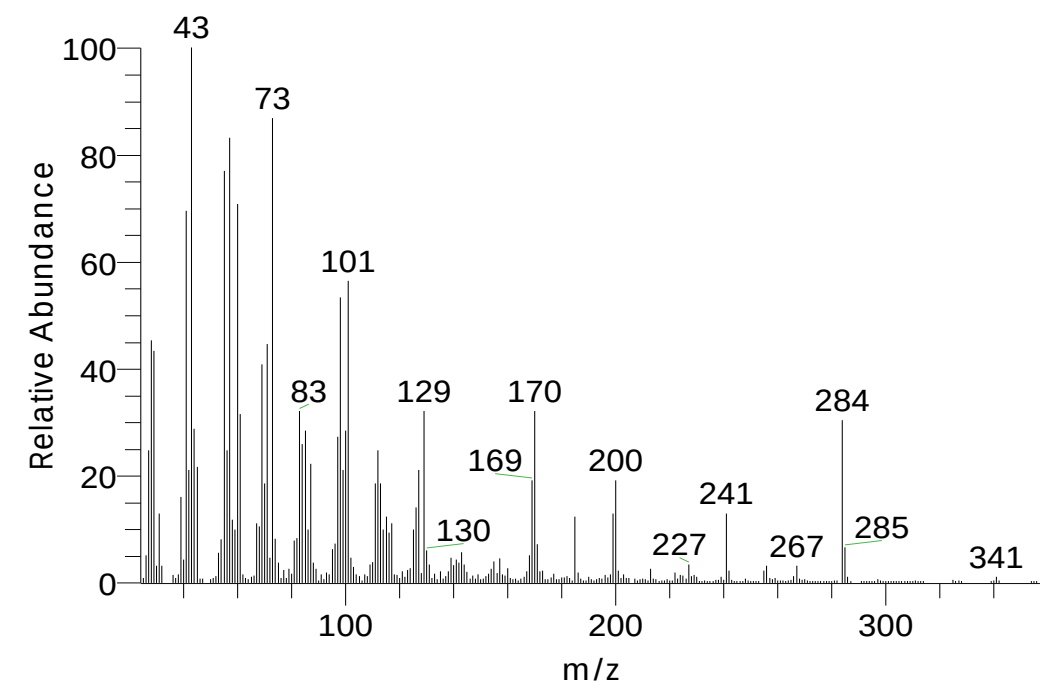

HEXADECANOIC ACID, 2-HYDROXY-1,3-PROPANEDIYL ESTER
Formula C35H68O5, MW 568, CAS# 502-52-3, Entry# 294143
2-HYDROXY-3-(PALMITOYLOXY)PROPYL PALMITATE #

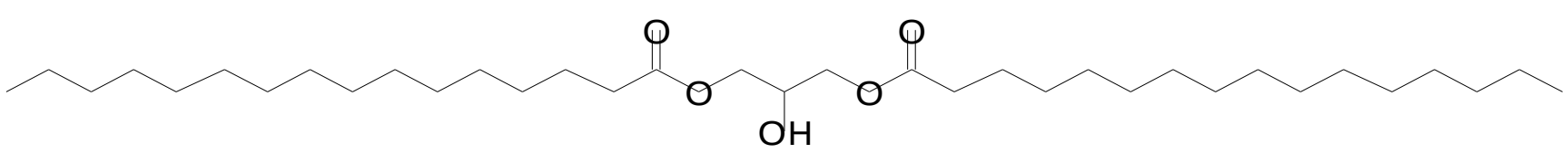

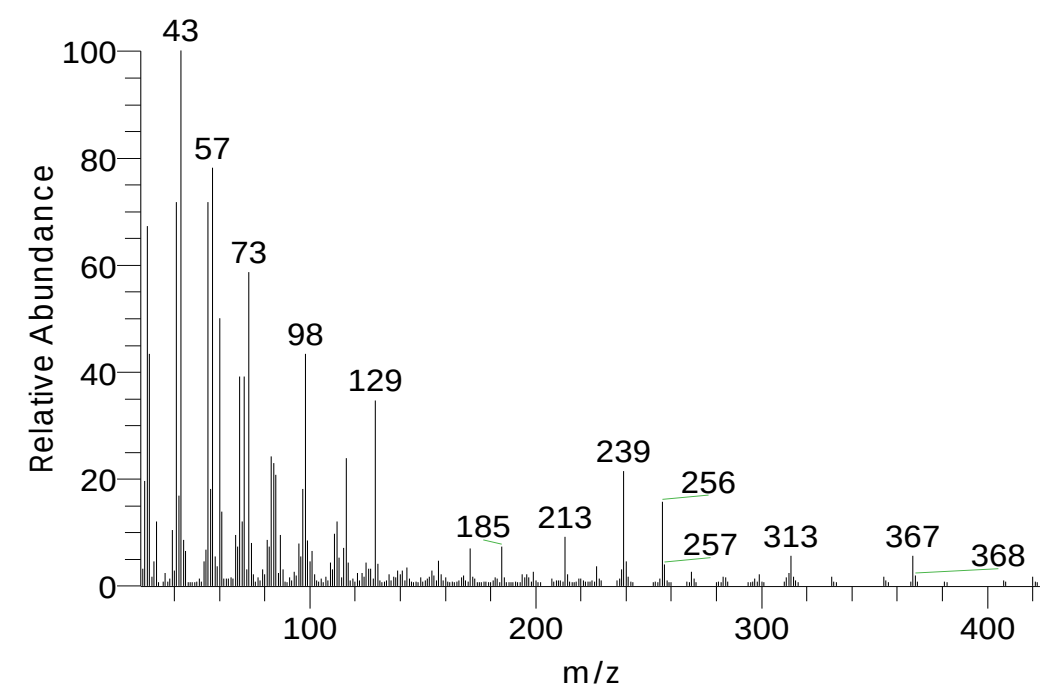

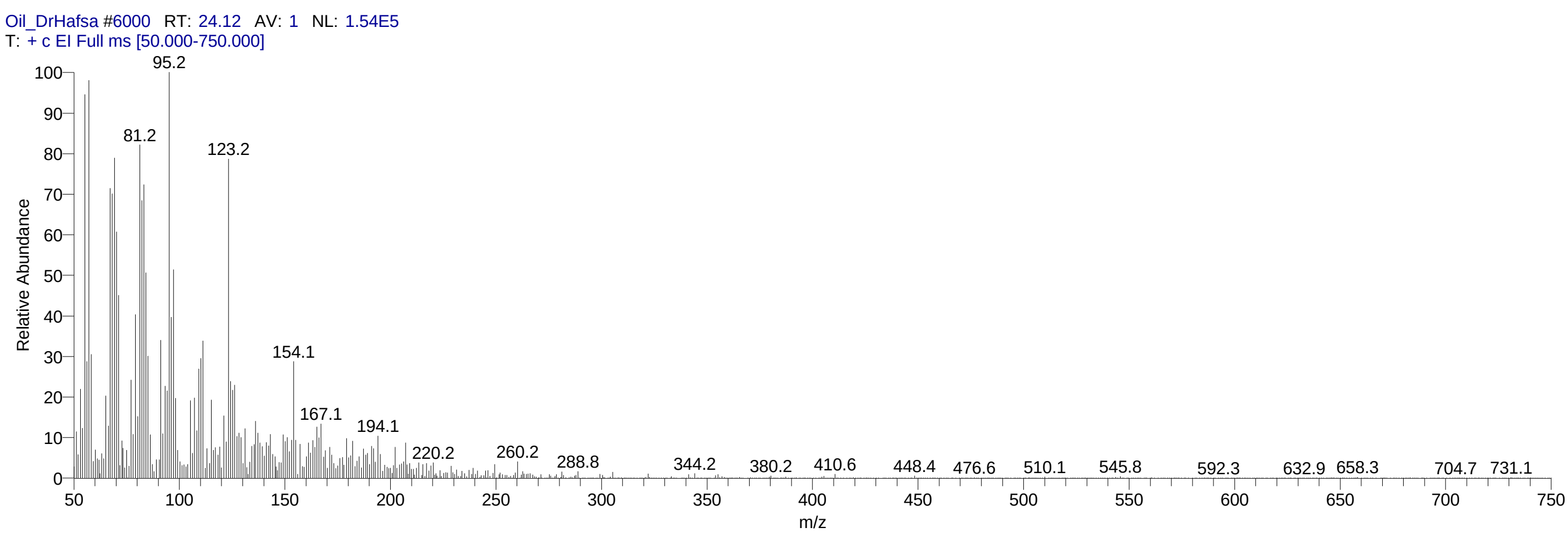

RT	Compound Name	Area %	MF	Molecular Formula	Molecular Weight	Cas #	Library
24.12	01297107001 TETRANEURIN - A - DIOL	0.18	753	C15H20O5	280	NA	WileyRegistry8e
24.12	Z-(13,14-Epoxy)tetradec-11-en-1-ol acetate	0.18	763	C16H28O3	268	NA	mainlib

My GC-MS Report

RT	Compound Name	Area %	MF	Molecular Formula	Molecular Weight	Cas #	Library
24.12	LACTAROPALLIDIN	0.18	754	C15H24O3	252	NA	WileyRegistry8e
24.12	1-Heptatriacotanol	0.18	748	C37H76O	536	105794-58-9	mainlib
24.12	E,E,Z-1,3,12-Nonadecatriene-5,14-diol	0.18	744	C19H34O2	294	NA	mainlib

Compound Structure | Hit Spectrum

01297107001 TETRANEURIN - A - DIOL
Formula C15H20O5, MW 280, CAS# NA, Entry# 170378

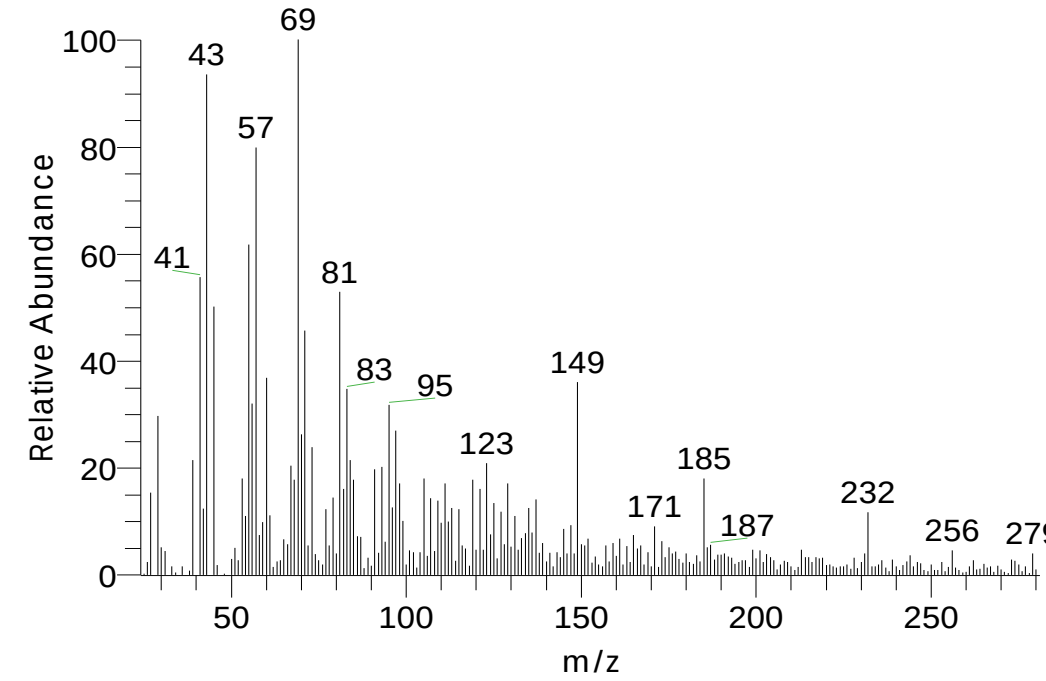

Z-(13,14-Epoxy)tetradec-11-en-1-ol acetate
Formula C16H28O3, MW 268, CAS# NA, Entry# 10459
(11Z)-12-(2-Oxiranyl)-11-dodecenyl acetate #

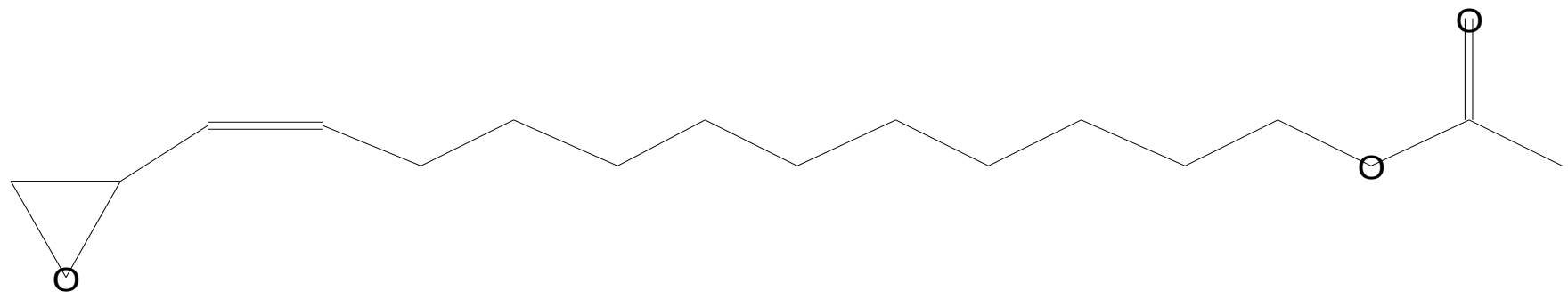

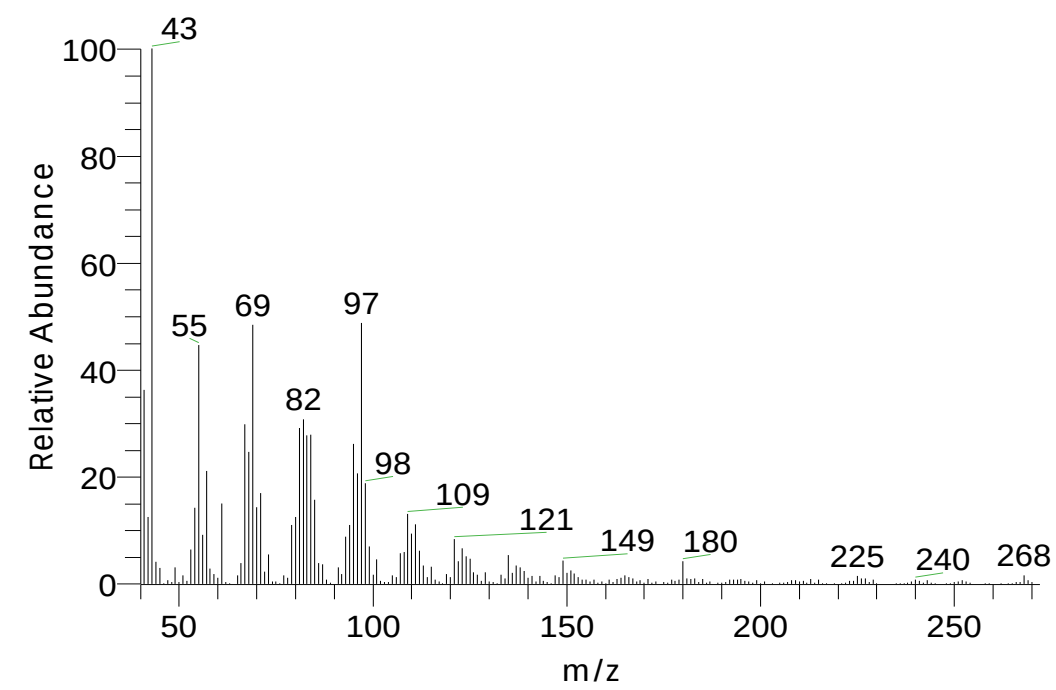

LACTAROPALLIDIN
Formula C15H24O3, MW 252, CAS# NA, Entry# 142433

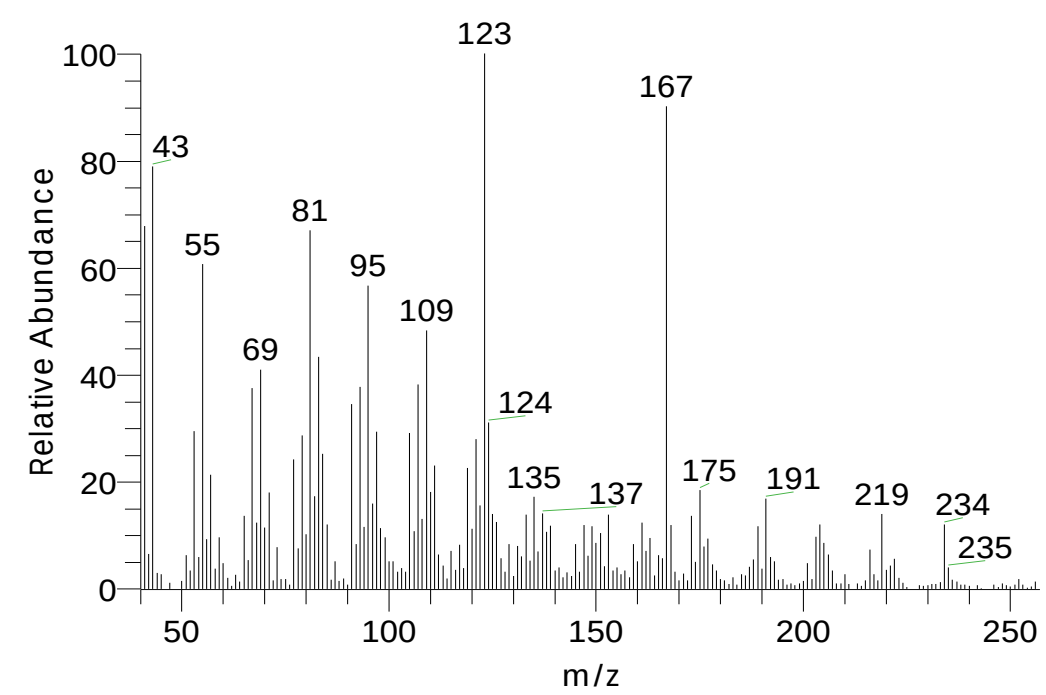

1-Heptatriacotanol
Formula C37H76O, MW 536, CAS# 105794-58-9, Entry# 7279
1-Heptatriacontanol #

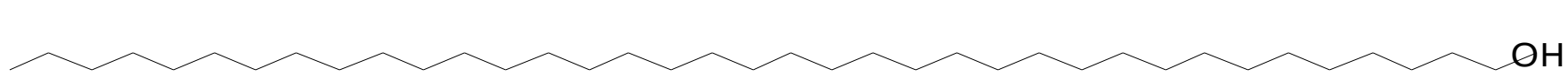

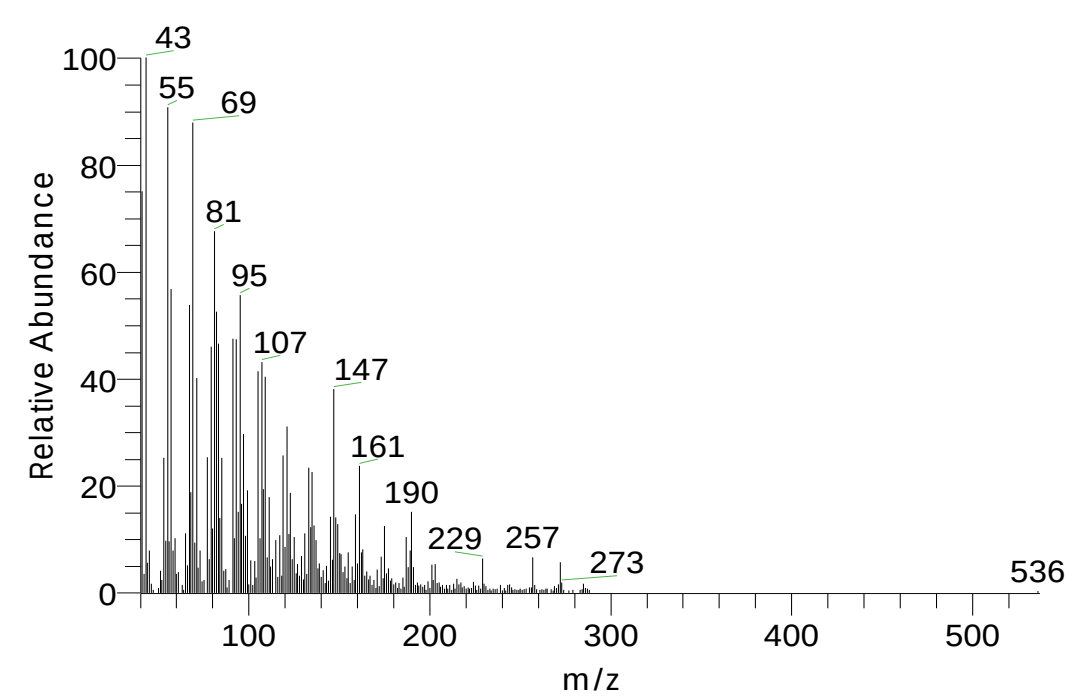

My GC-MS Report

Compound Structure | Hit Spectrum

E,E,Z-1,3,12-Nonadecatriene-5,14-diol
Formula C19H34O2, MW 294, CAS# NA, Entry# 21026
(3E,12Z)-1,3,12-Nonadecatriene-5,14-diol #

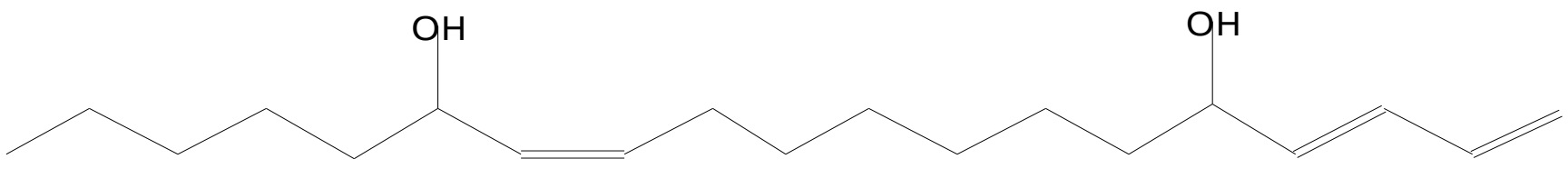

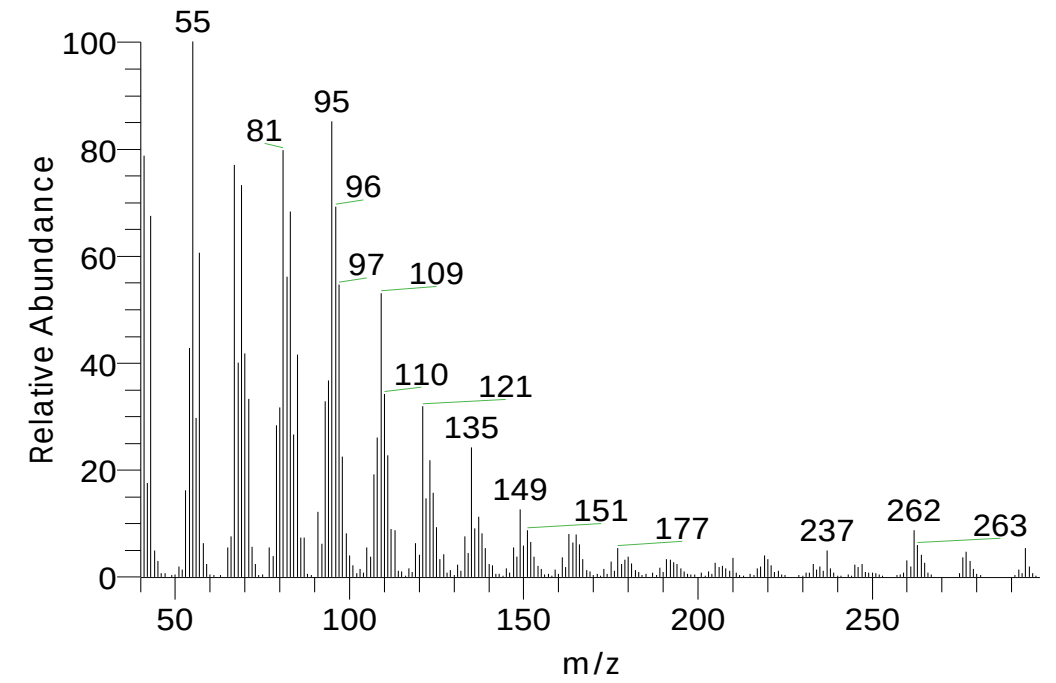

Oil_DrHafsa #6430 RT: 25.56 AV: 1 NL: 9.06E5
T: + c EI Full ms [50.000-750.000]

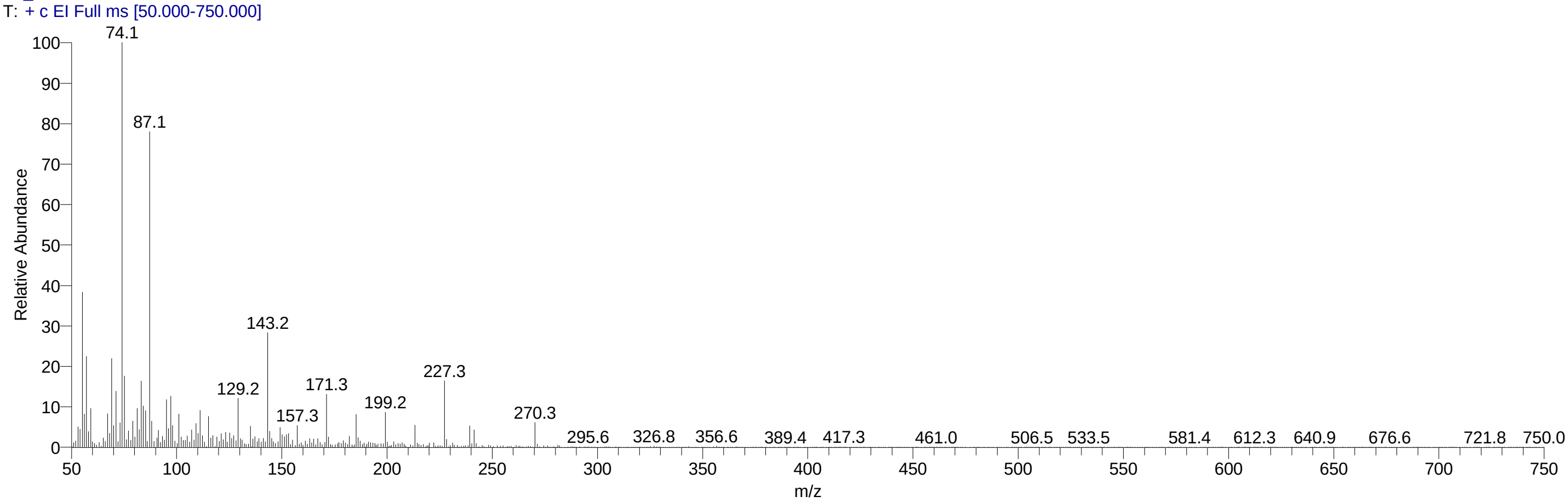

RT	Compound Name	Area %	MF	Molecular Formula	Molecular Weight	Cas #	Library
25.56	PENTADECANOIC ACID, 14-METHYL-, METHYL ESTER	0.62	807	C17H34O2	270	5129-60-2	WileyRegistry8e
25.56	Palmitic Acid methyl ester	0.62	800	C17H34O2	270	112-39-0	CaymanSpectralLibrary-NIST.HP
25.56	HEXADECANOIC ACID, METHYL ESTER	0.62	802	C17H34O2	270	112-39-0	WileyRegistry8e
25.56	HEXADECANOIC ACID, METHYL ESTER	0.62	829	C17H34O2	270	112-39-0	WileyRegistry8e
25.56	Hexadecanoic acid, methyl ester	0.62	817	C17H34O2	270	112-39-0	mainlib

Compound Structure | Hit Spectrum

PENTADECANOIC ACID, 14-METHYL-, METHYL ESTER
Formula C17H34O2, MW 270, CAS# 5129-60-2, Entry# 161312
METHYL 14-METHYLPENTADECANOATE

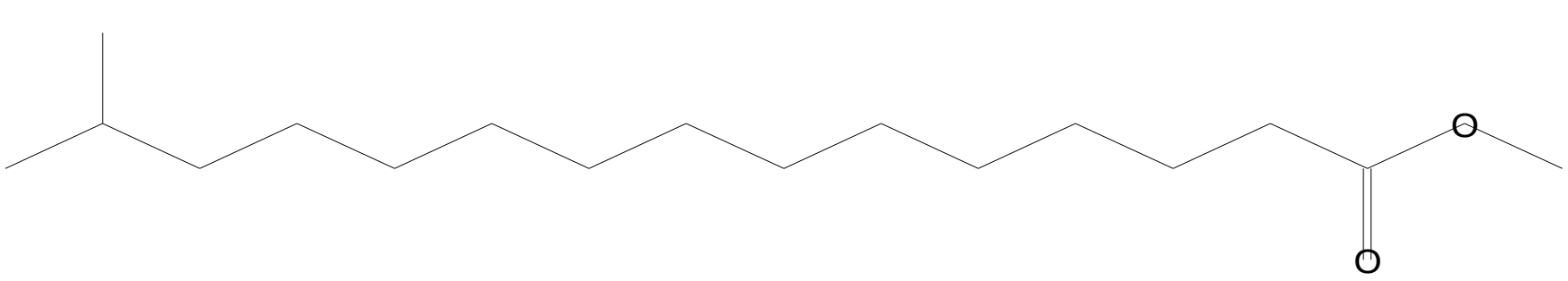

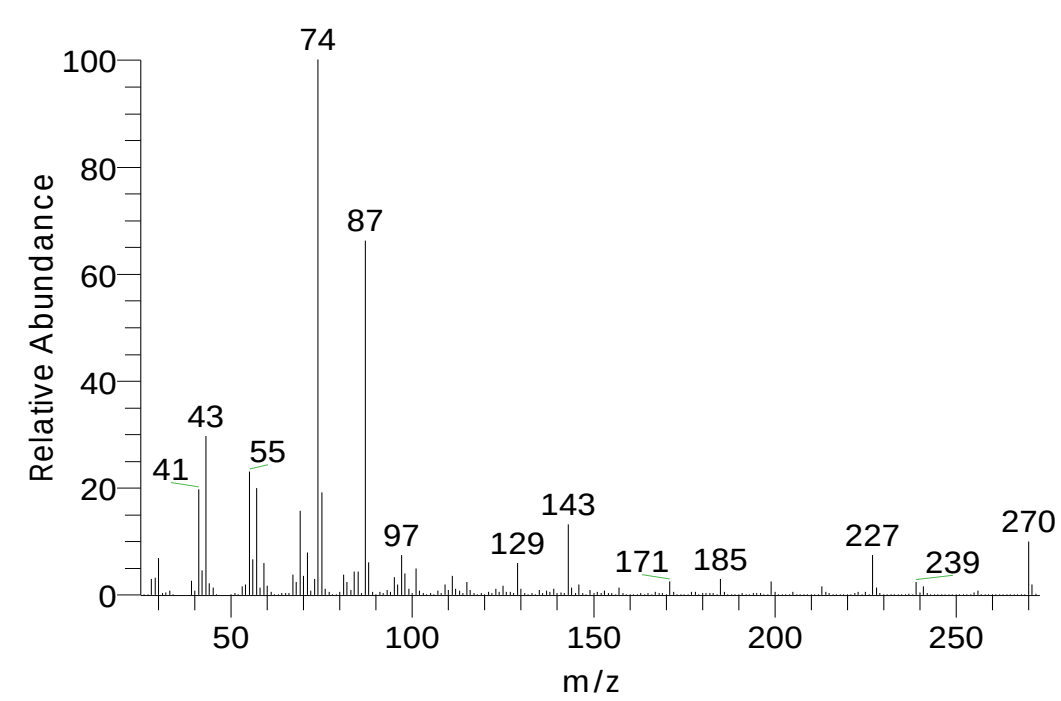

My GC-MS Report

Compound Structure | Hit Spectrum

Palmitic Acid methyl ester
Formula C17H34O2, MW 270, CAS# 112-39-0, Entry# 1088
Palmitic acid, methyl ester

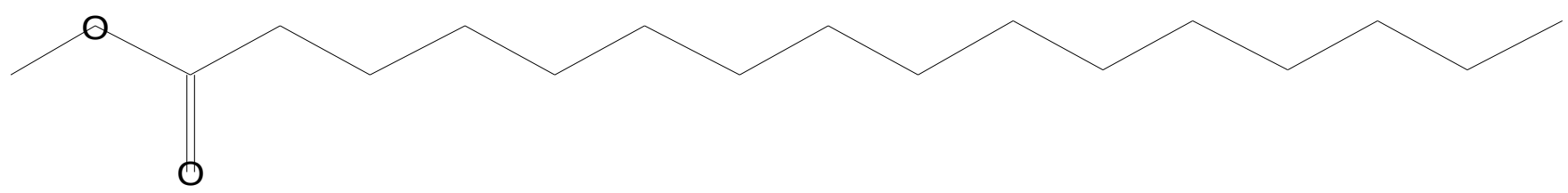

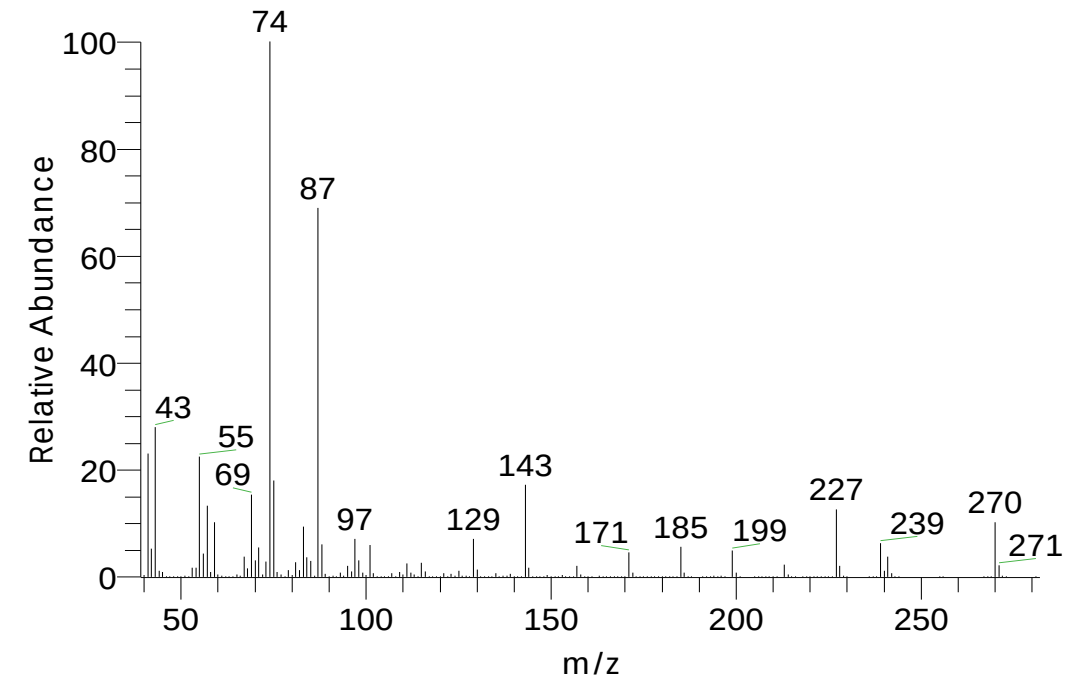

HEXADECANOIC ACID, METHYL ESTER
Formula C17H34O2, MW 270, CAS# 112-39-0, Entry# 161288
METHYL HEXADECANOATE

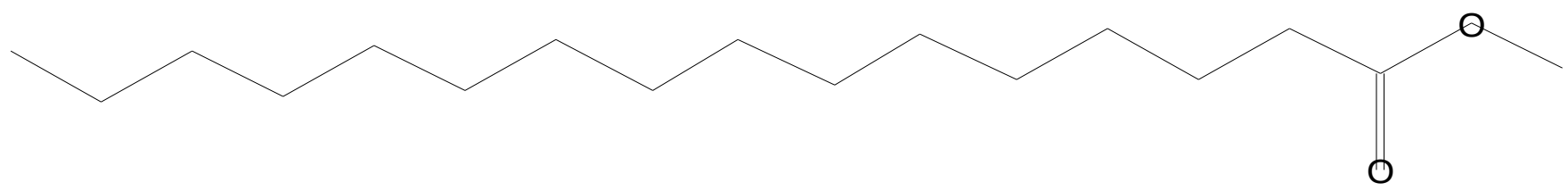

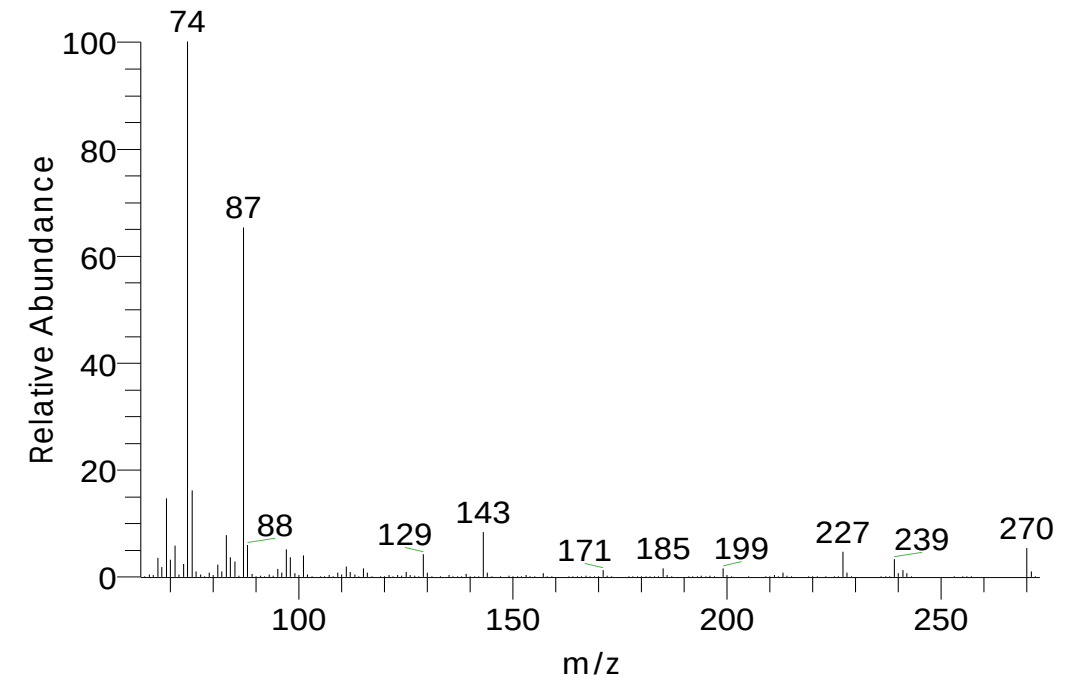

HEXADECANOIC ACID, METHYL ESTER
Formula C17H34O2, MW 270, CAS# 112-39-0, Entry# 161278
METHYL HEXADECANOATE

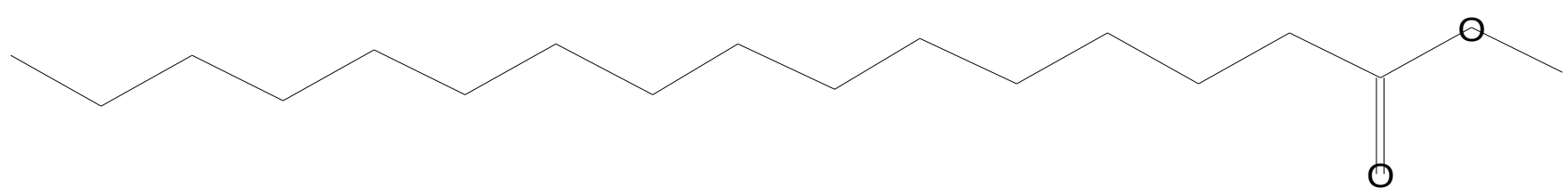

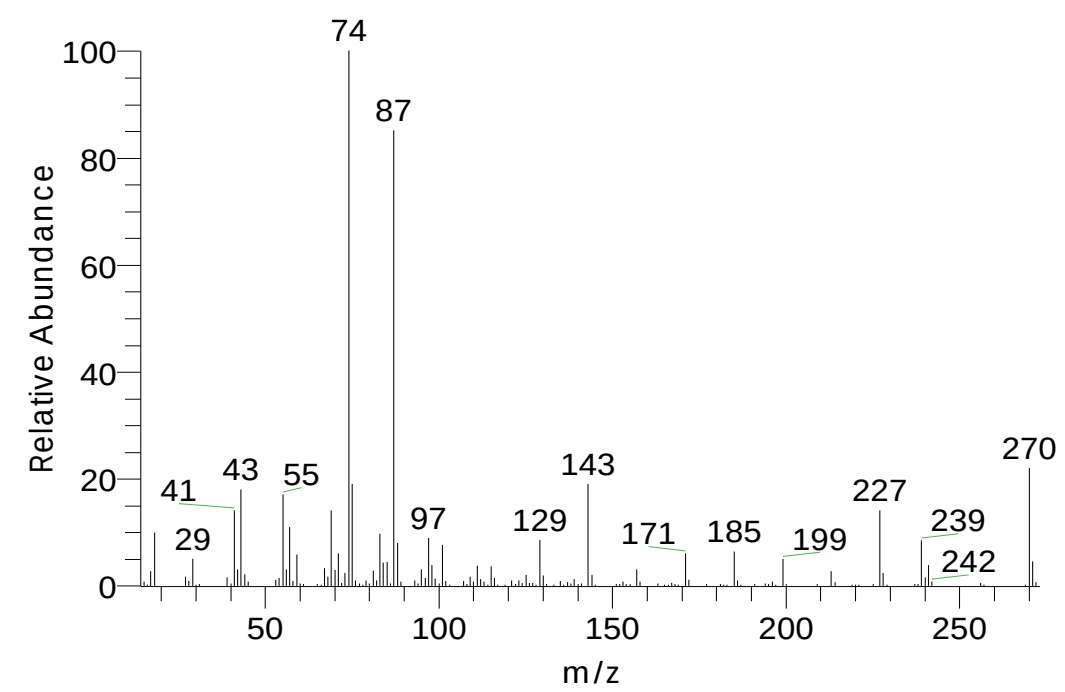

Hexadecanoic acid, methyl ester
Formula C17H34O2, MW 270, CAS# 112-39-0, Entry# 44729
Palmitic acid, methyl ester

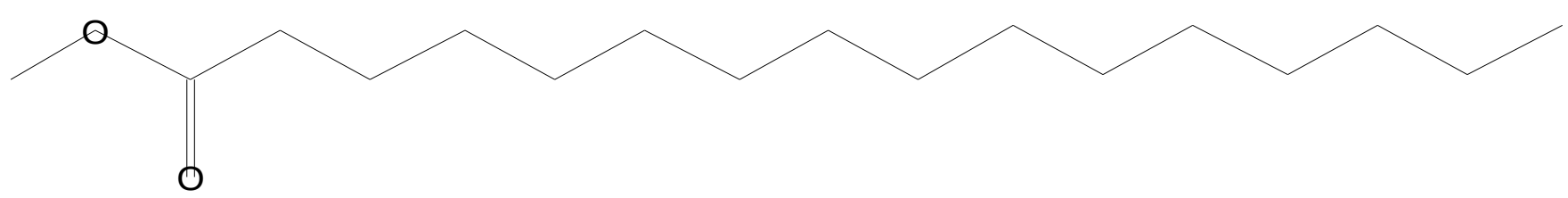

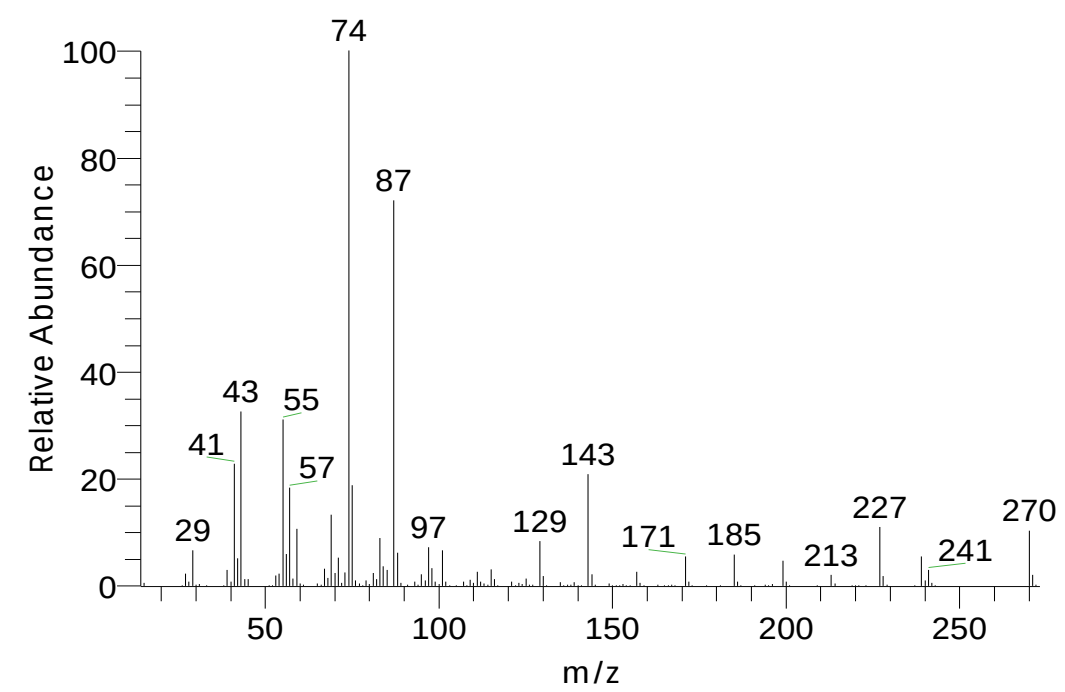

My GC-MS Report

Oil_DrHafsa #6624 RT: 26.21 AV: 1 NL: 5.35E6
T: + c EI Full ms [50.000-750.000]

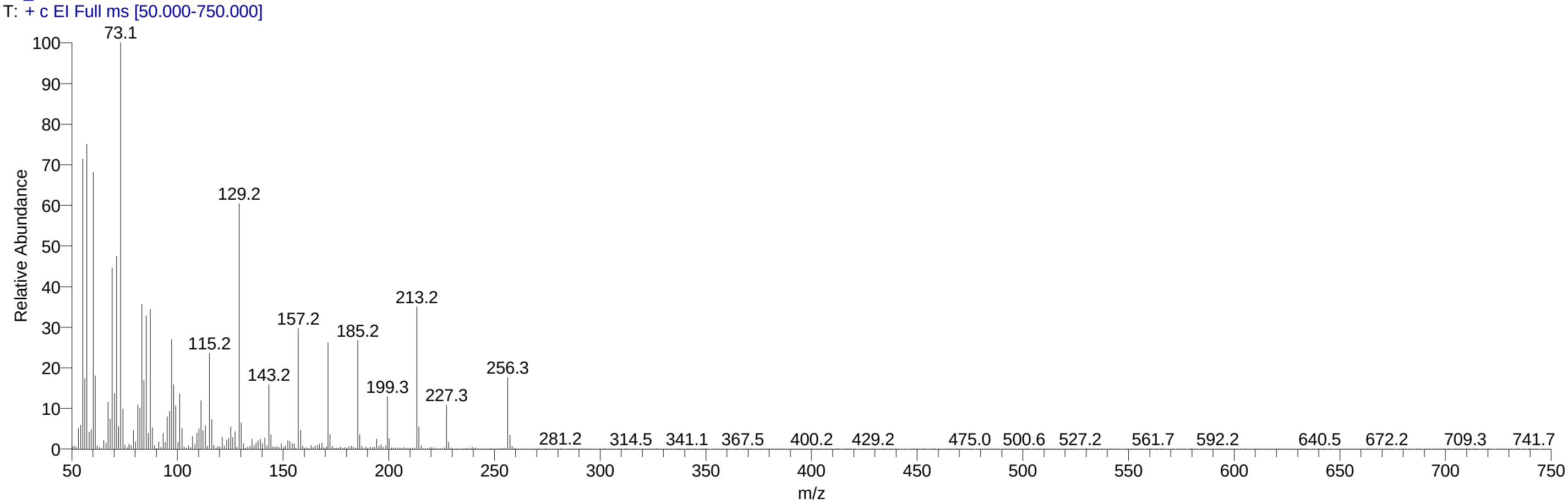

RT	Compound Name	Area %	MF	Molecular Formula	Molecular Weight	Cas #	Library
26.21	n-Hexadecanoic acid	8.89	924	C16H32O2	256	57-10-3	replib
26.21	HEXADECANOIC ACID	8.89	916	C16H32O2	256	57-10-3	WileyRegistry8e
26.21	HEXADECANOIC ACID	8.89	916	C16H32O2	256	57-10-3	WileyRegistry8e
26.21	n-Hexadecanoic acid	8.89	917	C16H32O2	256	57-10-3	replib
26.21	n-Hexadecanoic acid	8.89	867	C16H32O2	256	57-10-3	replib

Compound Structure | Hit Spectrum

n-Hexadecanoic acid
Formula C16H32O2, MW 256, CAS# 57-10-3, Entry# 9622
Hexadecanoic acid

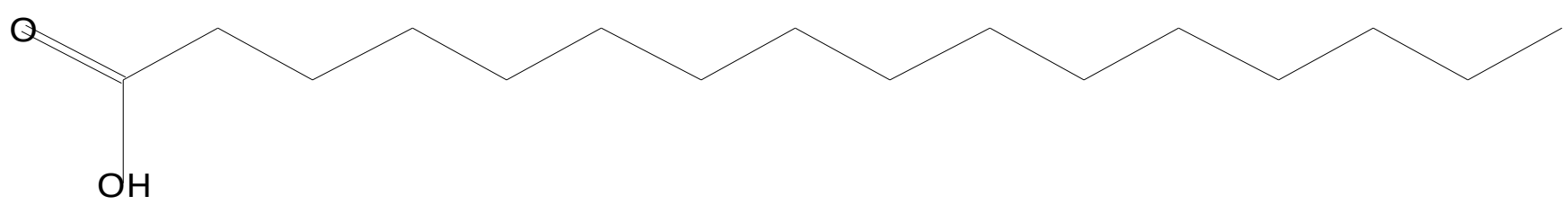

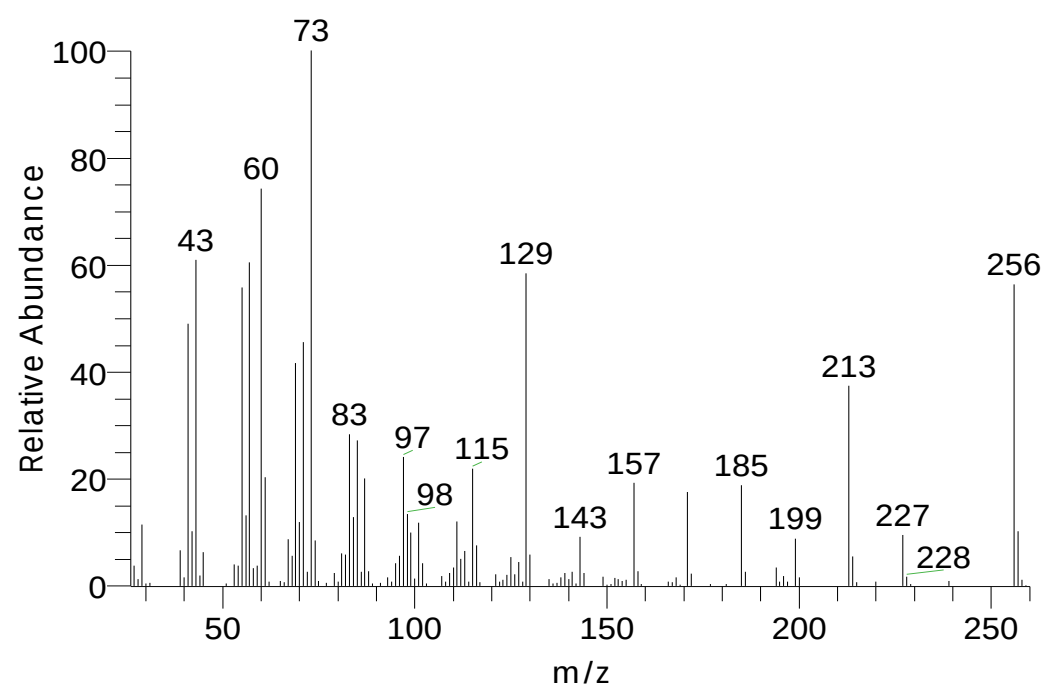

HEXADECANOIC ACID
Formula C16H32O2, MW 256, CAS# 57-10-3, Entry# 146744
HEXADECANOATE

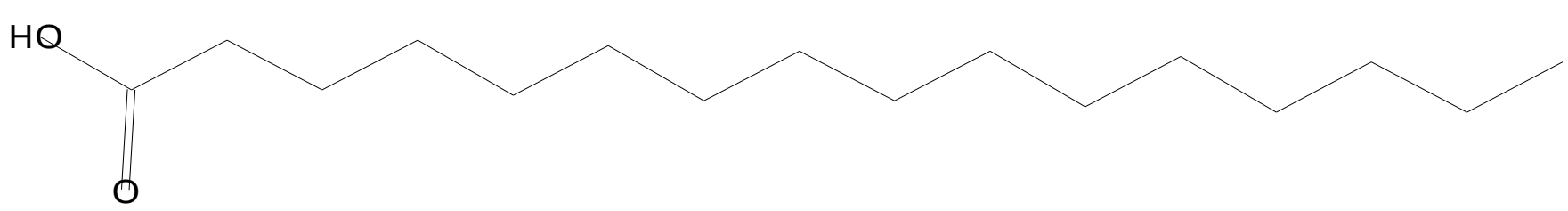

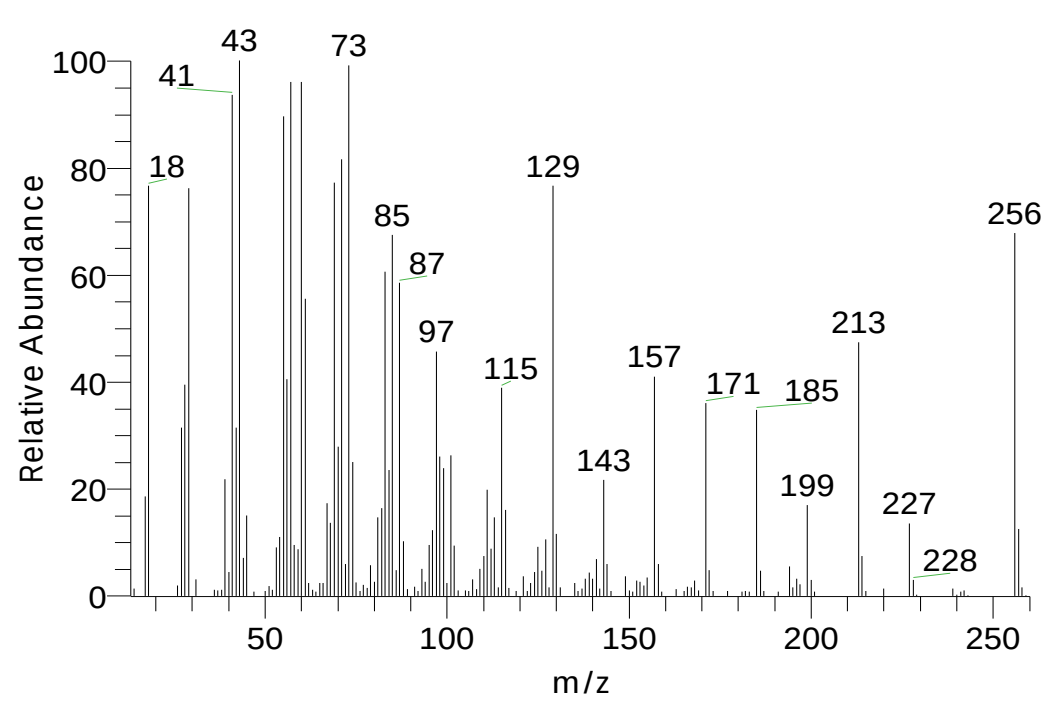

My GC-MS Report

Compound Structure | Hit Spectrum

HEXADECANOIC ACID
Formula C16H32O2, MW 256, CAS# 57-10-3, Entry# 397116
HEXADECANOATE

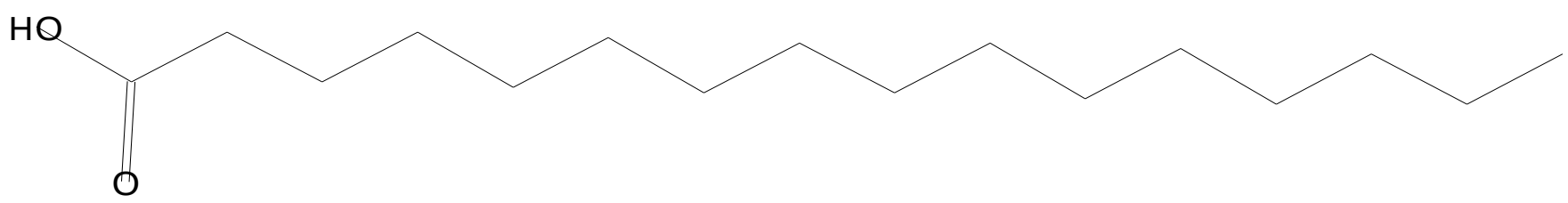

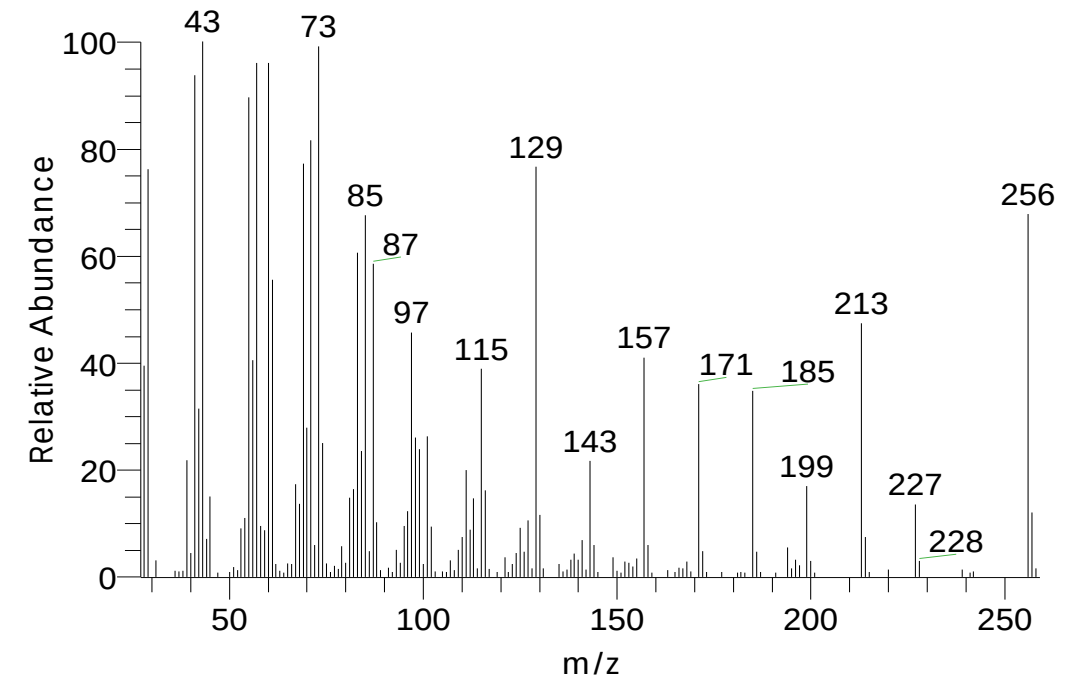

n-Hexadecanoic acid
Formula C16H32O2, MW 256, CAS# 57-10-3, Entry# 7566
Hexadecanoic acid

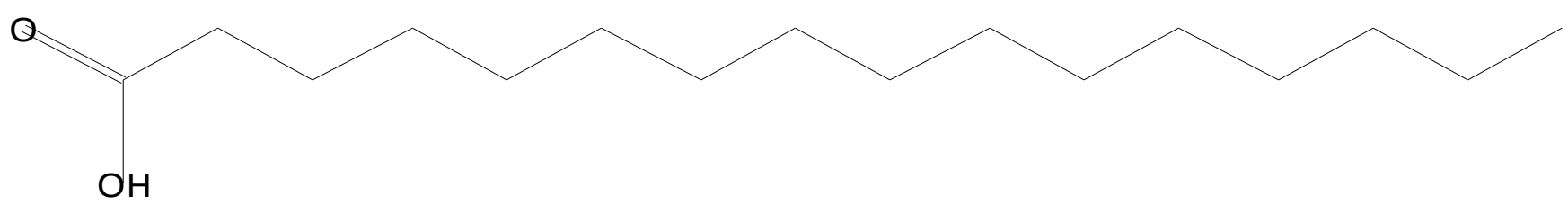

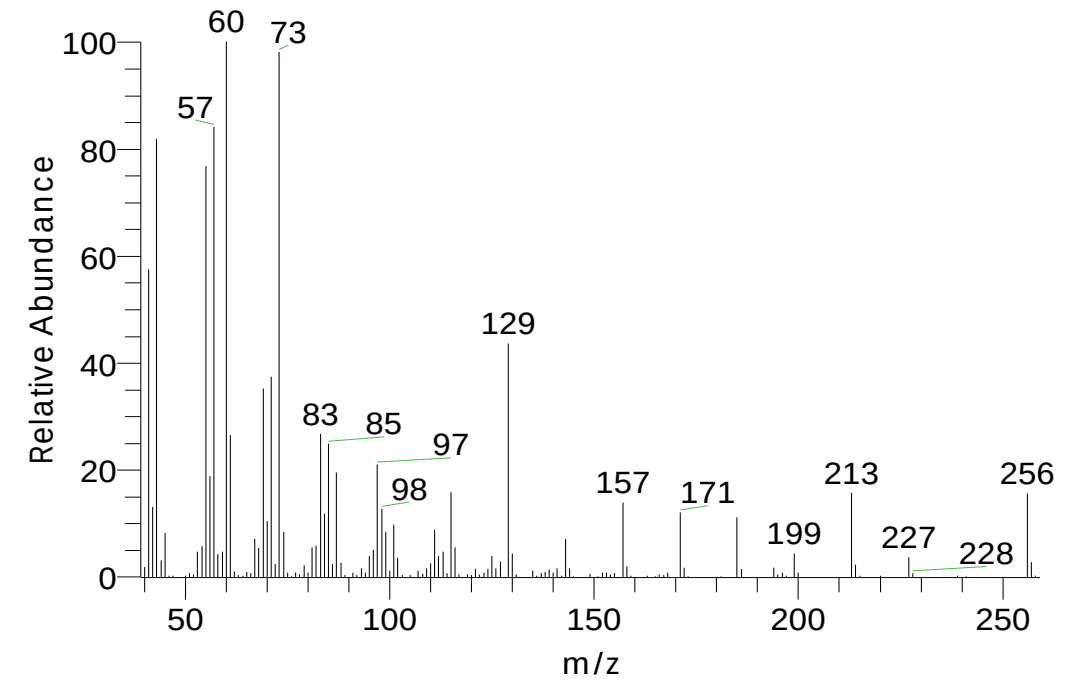

n-Hexadecanoic acid
Formula C16H32O2, MW 256, CAS# 57-10-3, Entry# 2779
Hexadecanoic acid

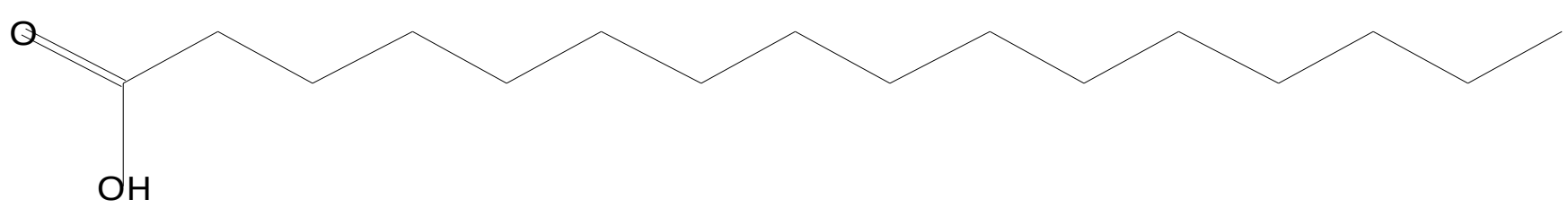

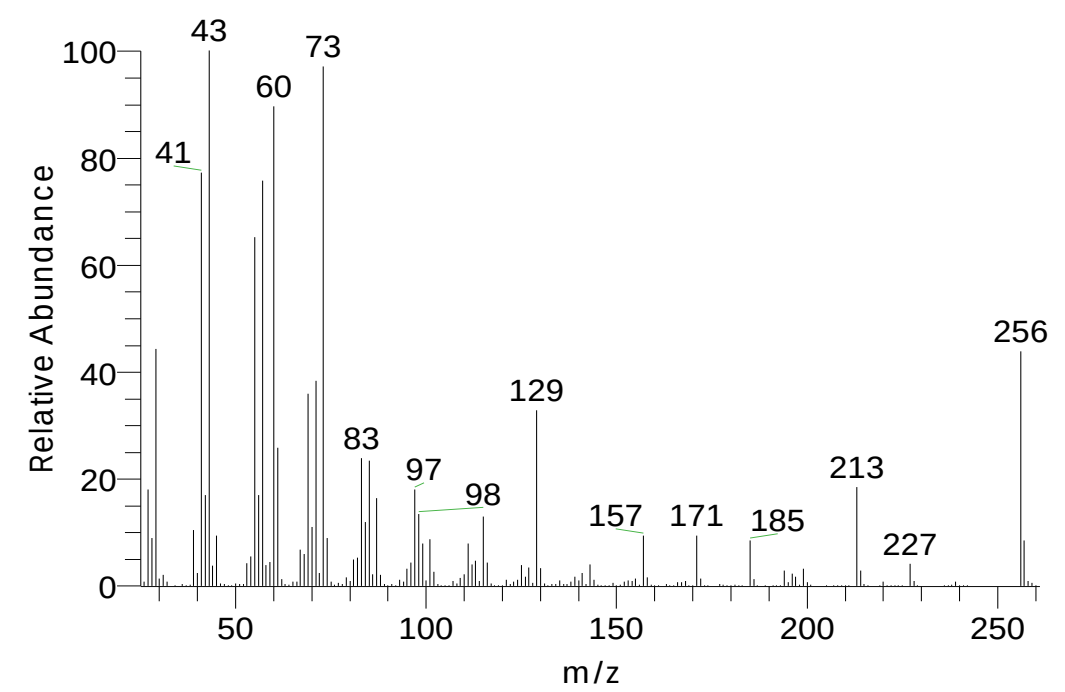

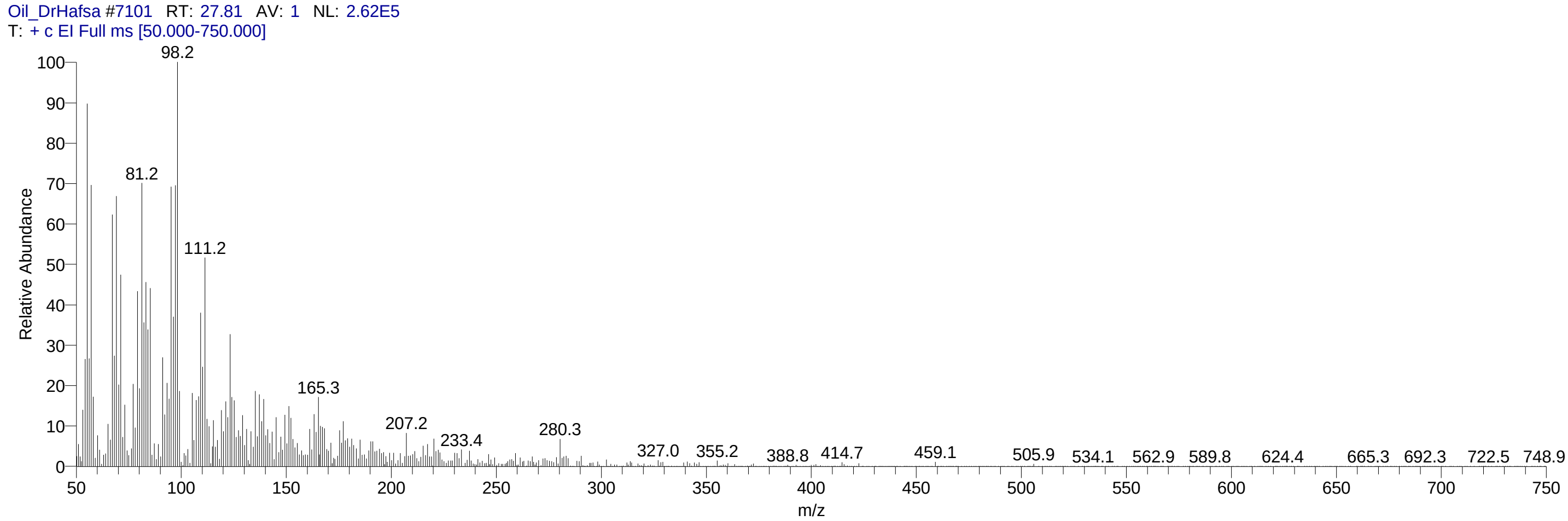

RT	Compound Name	Area %	MF	Molecular Formula	Molecular Weight	Cas #	Library
27.81	1-Heptatriacotanol	0.27	784	C37H76O	536	105794-58-9	mainlib
27.81	9-Octadecenoic acid, 1,2,3-propanetriyl ester, (E,E,E)-	0.27	740	C57H104O6	884	537-39-3	mainlib

My GC-MS Report

RT	Compound Name	Area %	MF	Molecular Formula	Molecular Weight	Cas #	Library
27.81	(E)-13-Docosenoic acid	0.27	742	C22H42O2	338	506-33-2	mainlib
27.81	HAHNFETT	0.27	726	N/A	0	NA	WileyRegistry8e
27.81	HAHNFETT	0.27	726	N/A	0	NA	WileyRegistry8e

Compound Structure | Hit Spectrum

1-Heptatriacotanol
Formula C37H76O, MW 536, CAS# 105794-58-9, Entry# 7279
1-Heptatriacontanol #

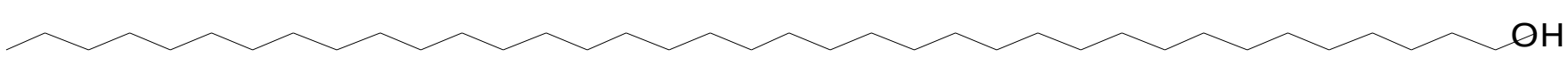

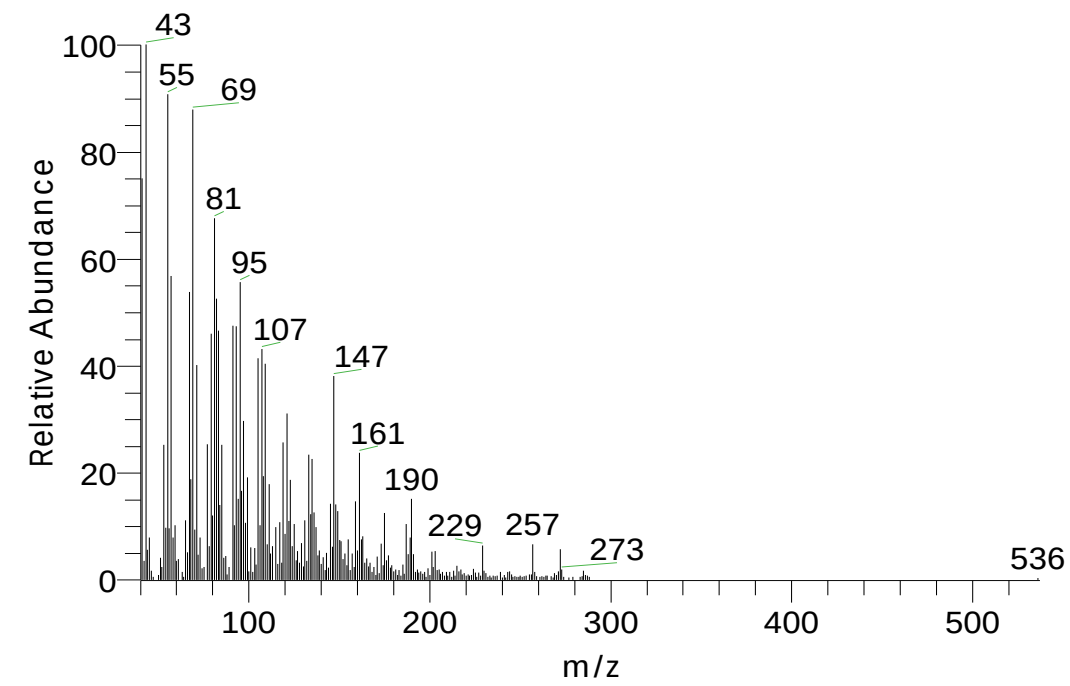

9-Octadecenoic acid, 1,2,3-propanetriyl ester, (E,E,E)-
Formula C57H104O6, MW 884, CAS# 537-39-3, Entry# 20268
2,3-Bis[(9E)-9-octadecenoyloxy]propyl (9E)-9-octadecenoate #

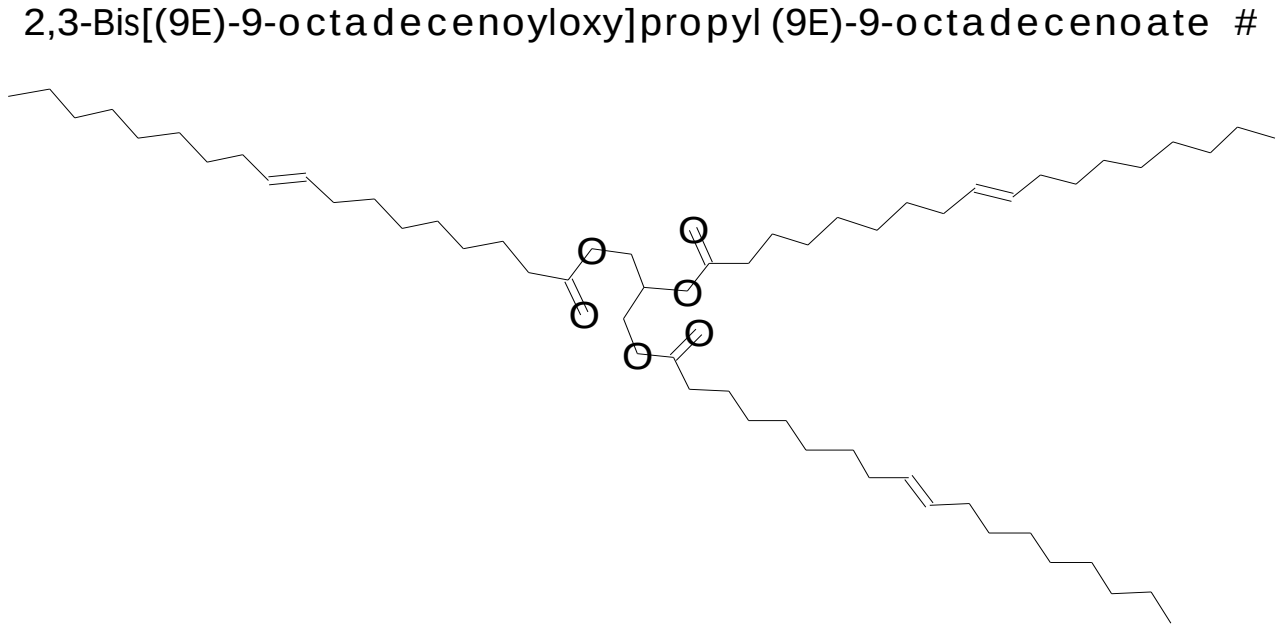

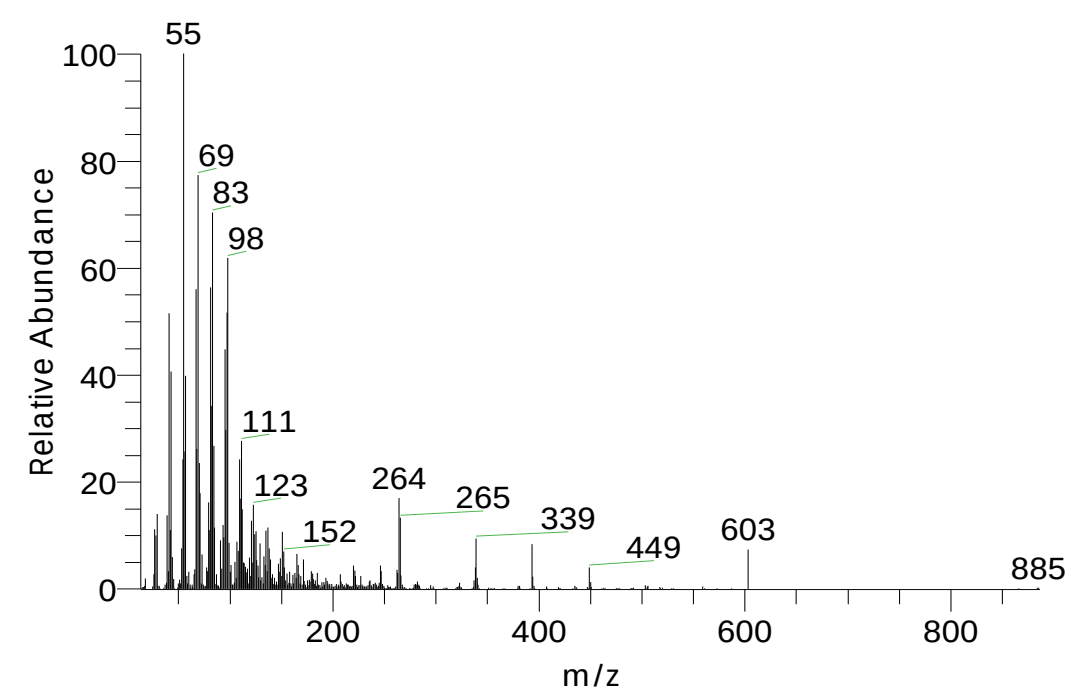

(E)-13-Docosenoic acid
Formula C22H42O2, MW 338, CAS# 506-33-2, Entry# 2721
13-Docosenoic acid, (E)-

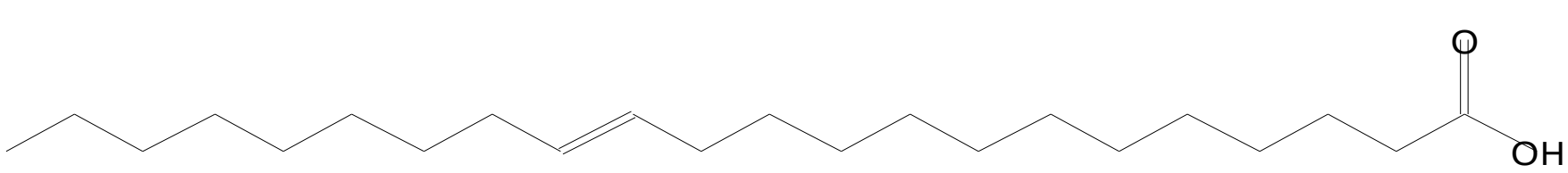

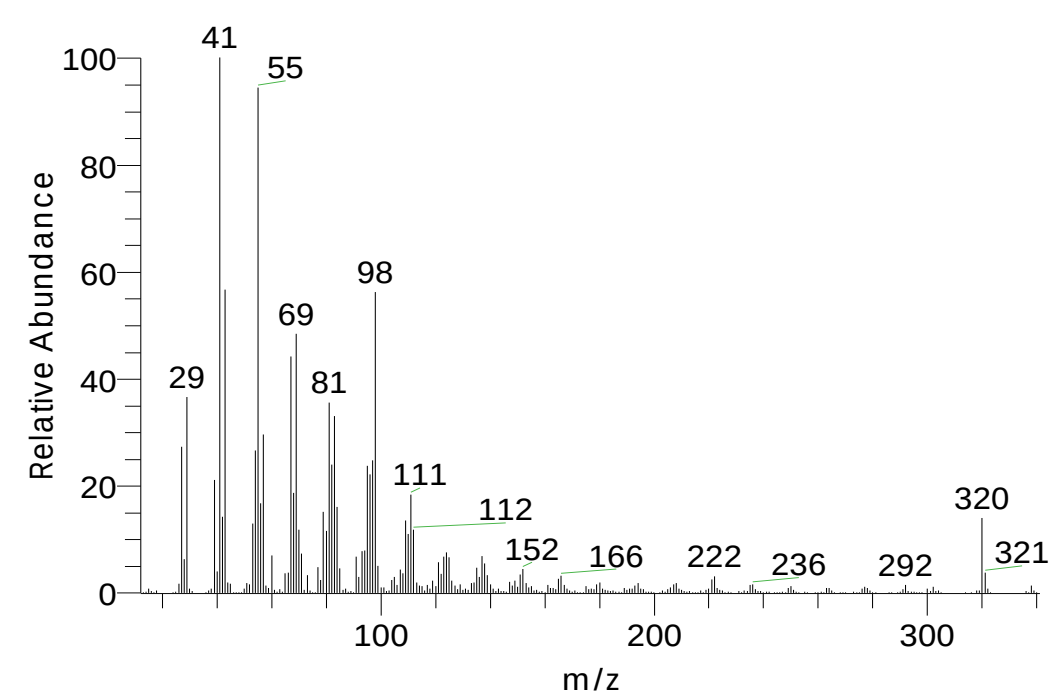

HAHNFETT
Formula , MW 0, CAS# NA, Entry# 305496

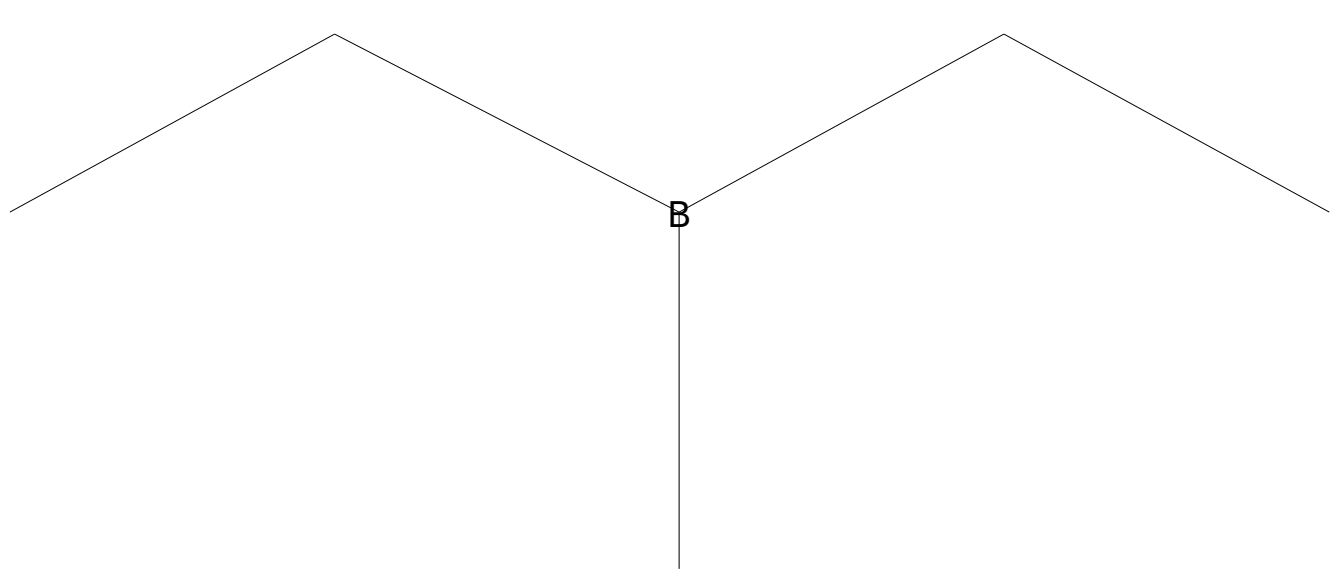

SI 717, RSI 726, WileyRegistry8e, Entry# 305496, CAS# NA, HAHNFETT

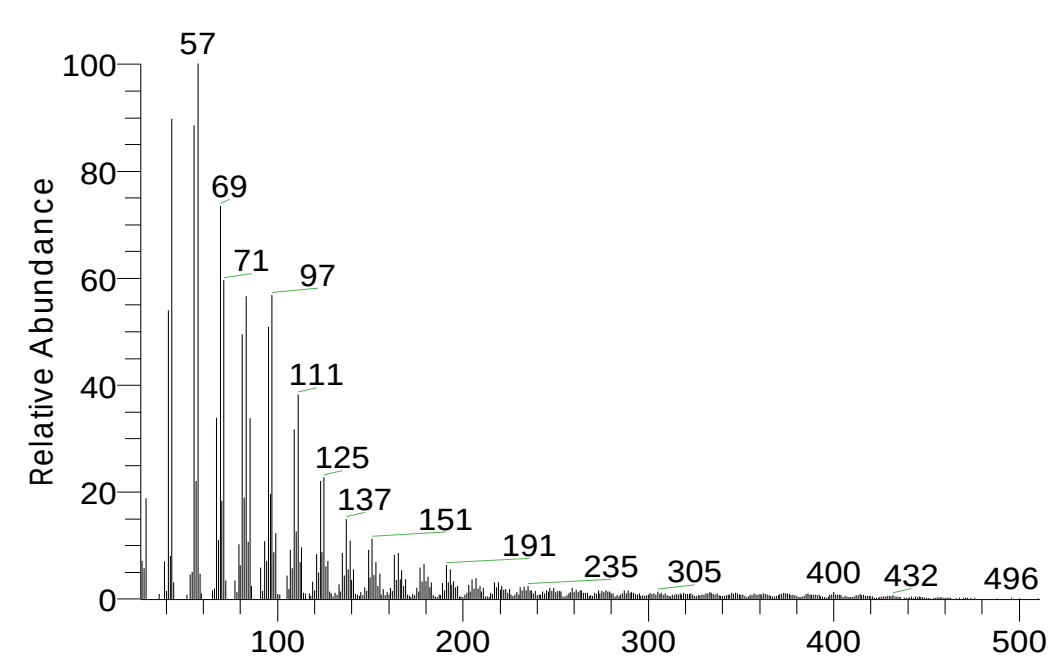

My GC-MS Report

Compound Structure | Hit Spectrum

HAHNFETT
Formula , MW 0, CAS# NA, Entry# 391160

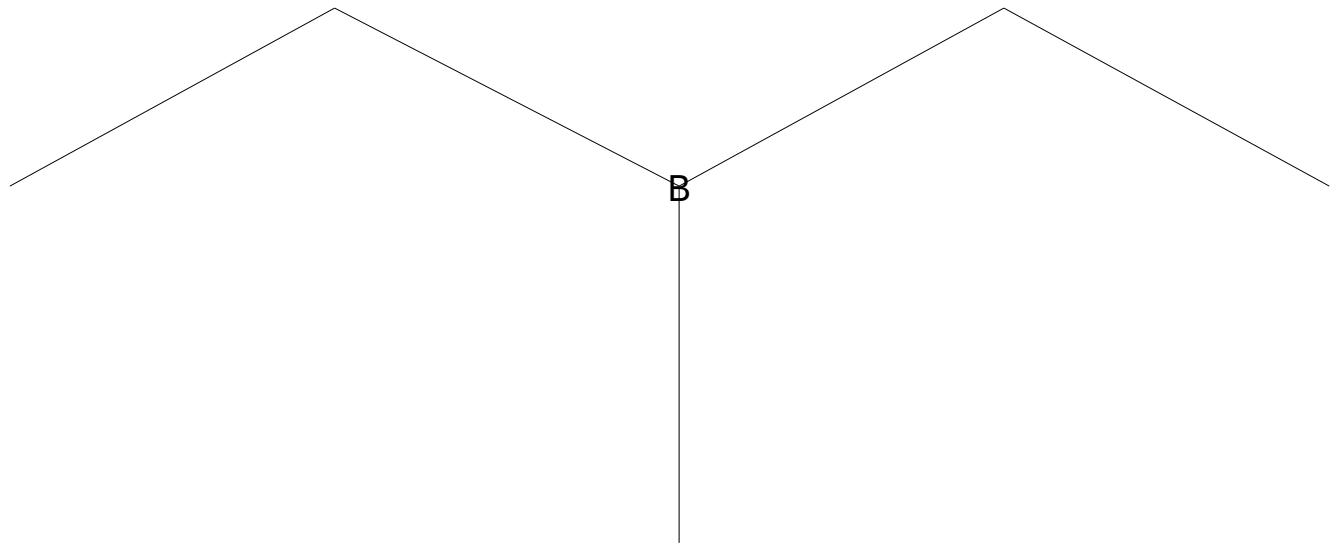

SI 717, RSI 726, WileyRegistry8e, Entry# 391160, CAS# NA, HAHNFETT

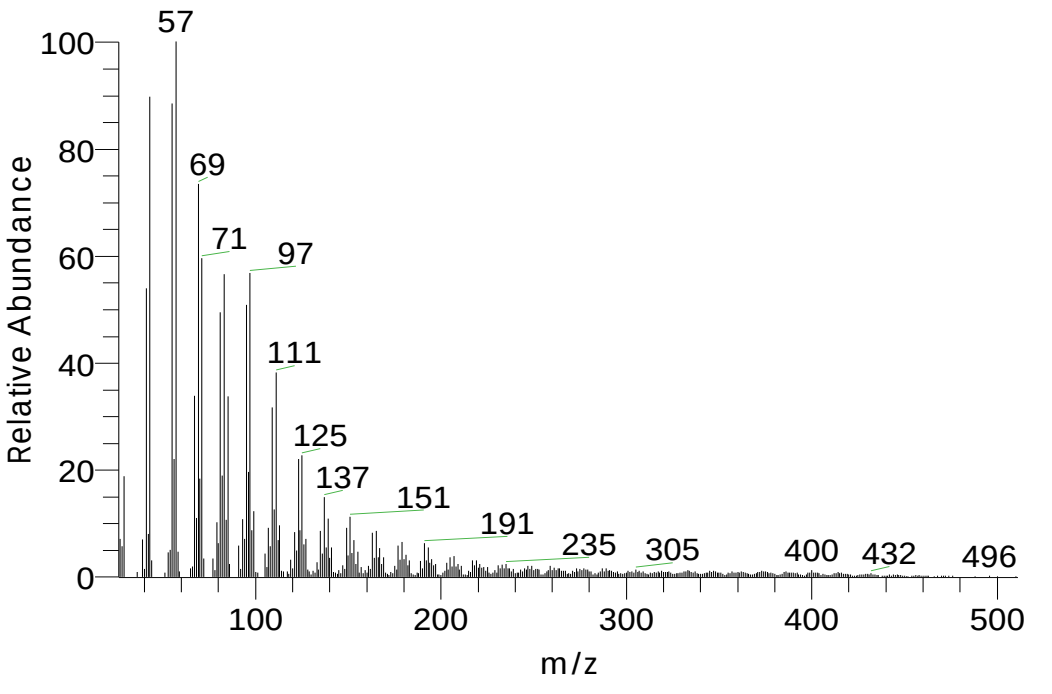

Oil_DrHafsa #7178 RT: 28.07 AV: 1 NL: 3.08E5
T: + c EI Full ms [50.000-750.000]

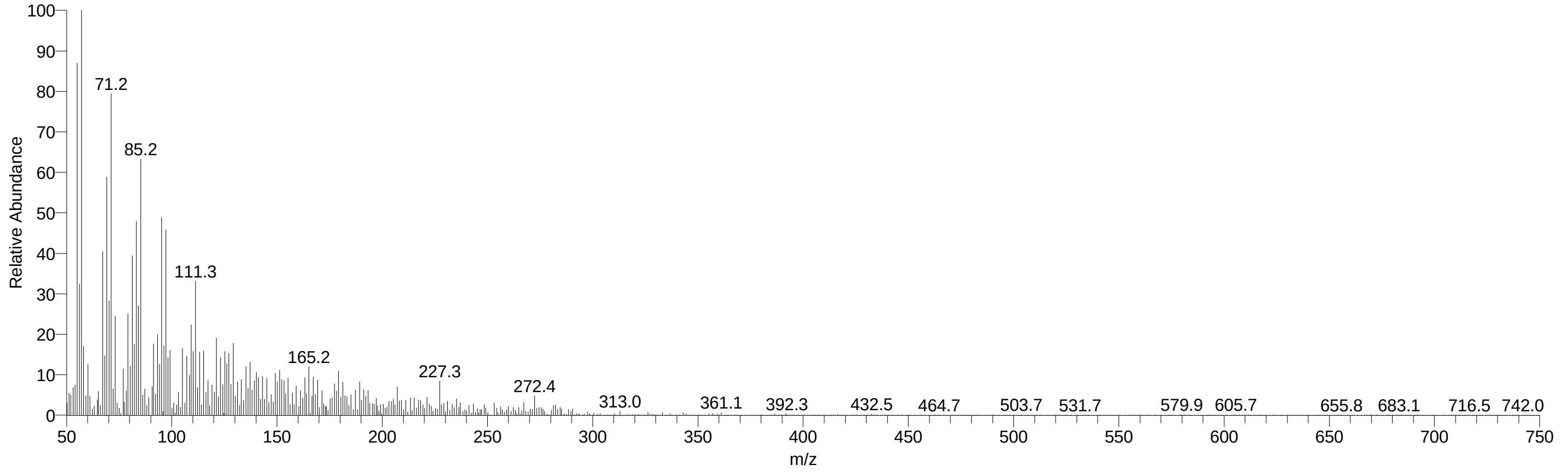

RT	Compound Name	Area %	MF	Molecular Formula	Molecular Weight	Cas #	Library
28.07	ISOCHIAPIN B	0.26	795	C19H22O6	346	NA	WileyRegistry8e
28.07	ISOCHIAPIN B %2<	0.26	795	C19H26O6	350	NA	WileyRegistry8e
28.07	1-Heptatriacotanol	0.26	795	C37H76O	536	105794-58-9	mainlib
28.07	DOTRIACONTANE	0.26	790	C32H66	450	544-85-4	WileyRegistry8e
28.07	2-ACETYL-3-(2-CINNAMIDO)ETHYL-7-METHOXYINDOLE	0.26	793	C22H22N2O3	362	NA	WileyRegistry8e

Compound Structure | Hit Spectrum

ISOCHIAPIN B
Formula C19H22O6, MW 346, CAS# NA, Entry# 225807

SI 793, RSI 795, WileyRegistry8e, Entry# 225807, CAS# NA, ISOCHIAPIN B

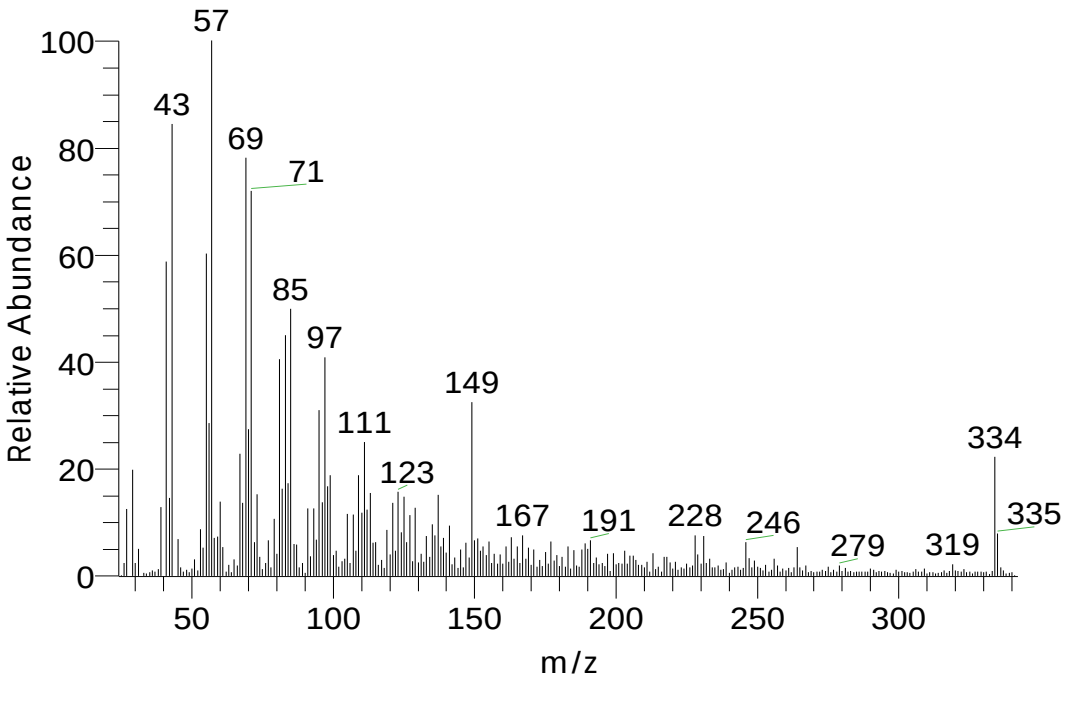

My GC-MS Report

Compound Structure | Hit Spectrum

ISOCHIAPIN B %2<
Formula C19H26O6, MW 350, CAS# NA, Entry# 228500

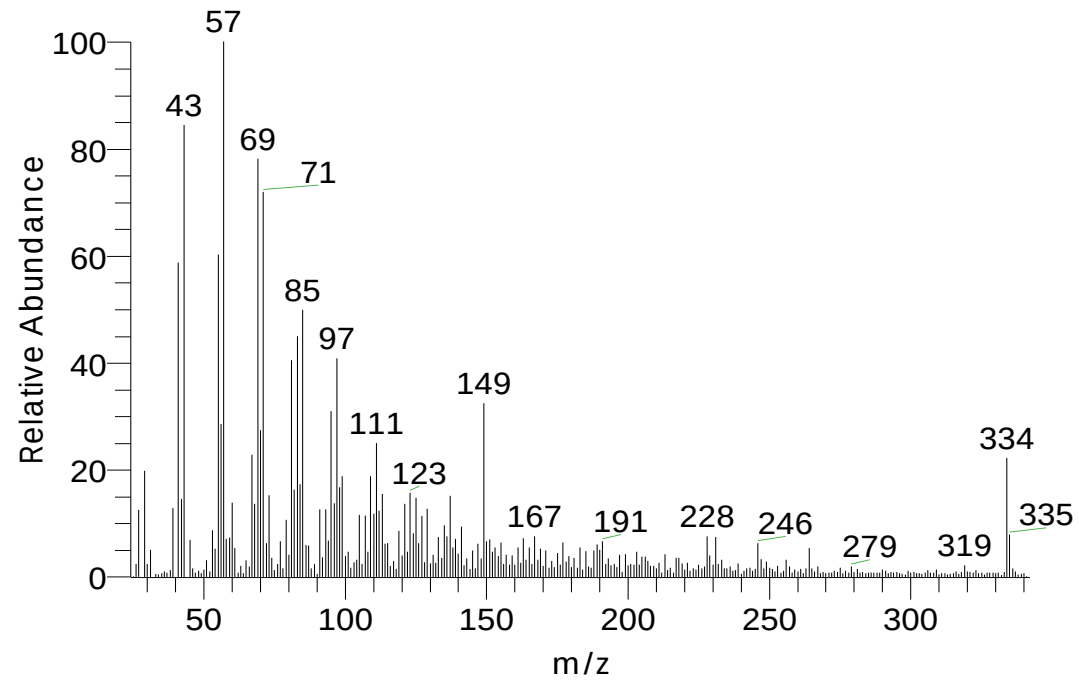

1-Heptatriacotanol
Formula C37H76O, MW 536, CAS# 105794-58-9, Entry# 7279
1-Heptatriacontanol #

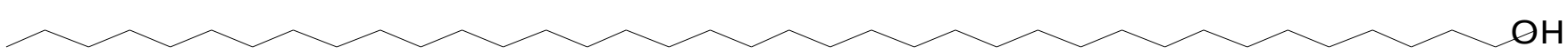

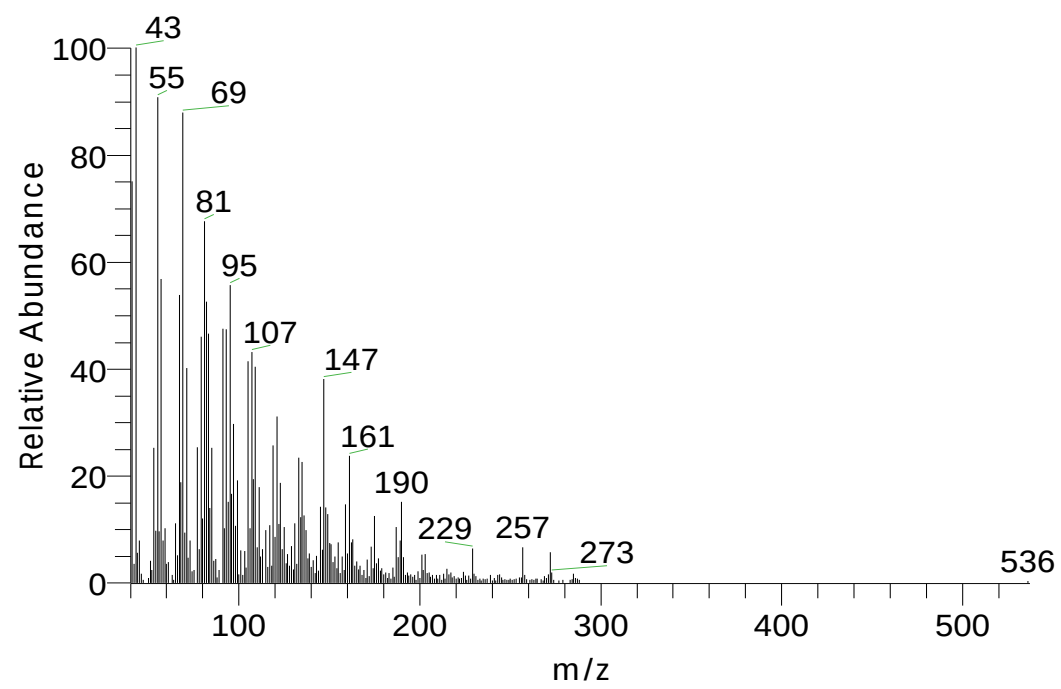

DOTRIACONTANE
Formula C32H66, MW 450, CAS# 544-85-4, Entry# 274478
AI3-52367

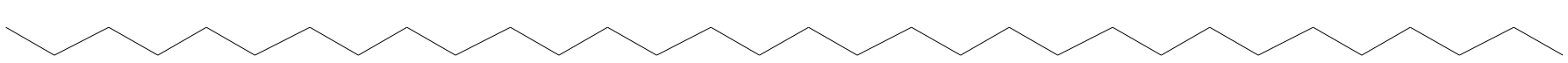

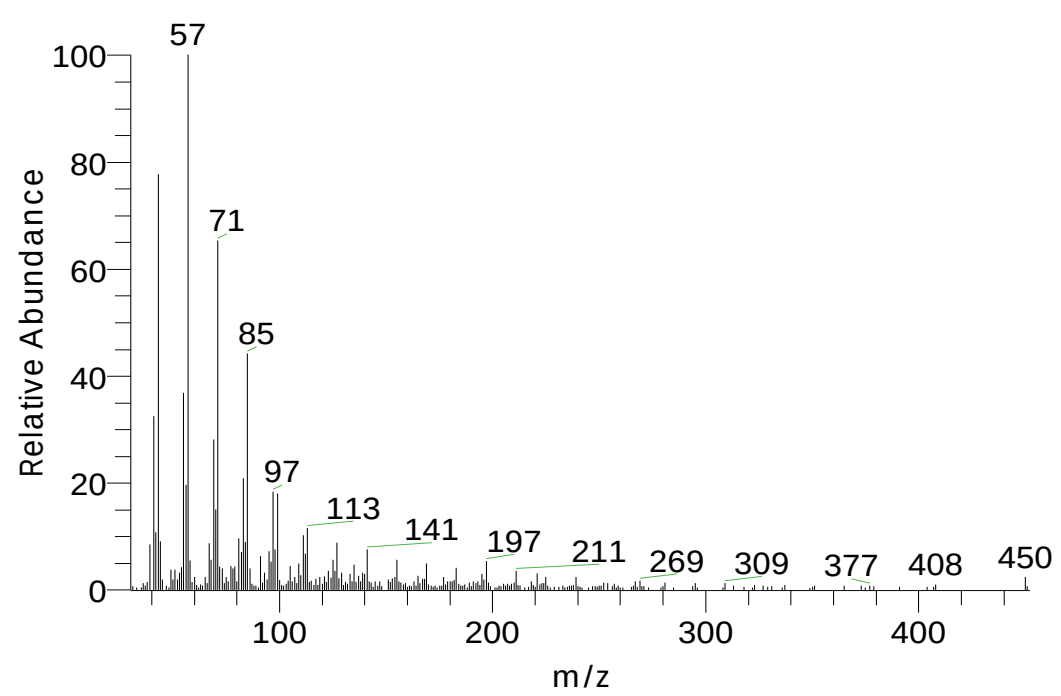

2-ACETYL-3-(2-CINNAMIDO)ETHYL-7-METHOXYINDOLE
Formula C22H22N2O3, MW 362, CAS# NA, Entry# 236392

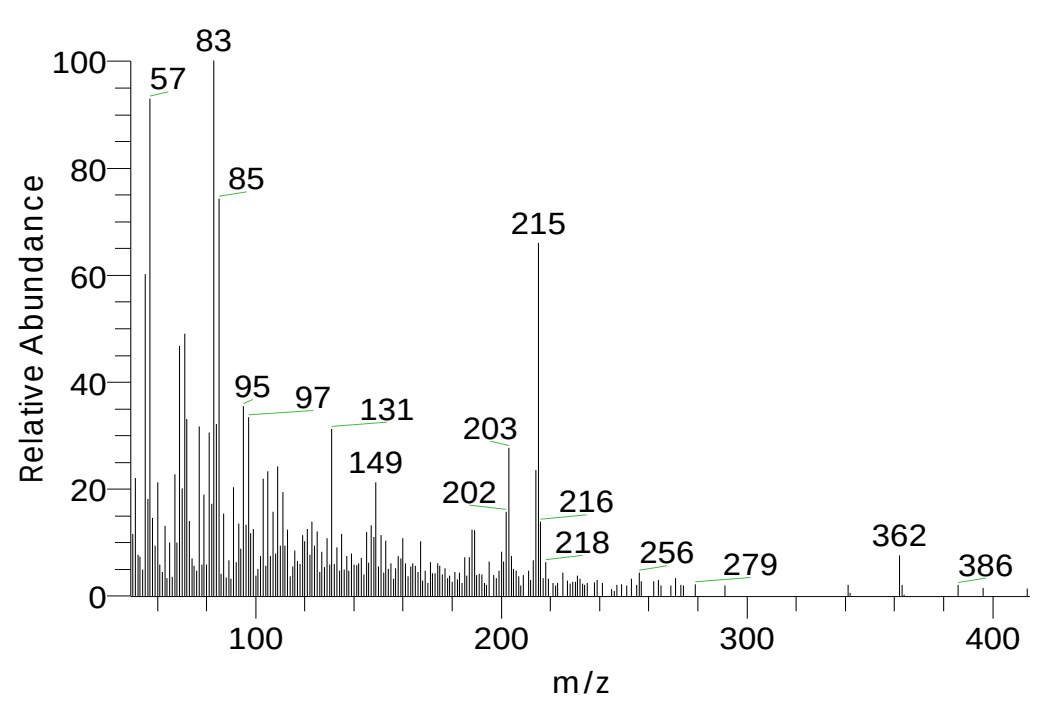

My GC-MS Report

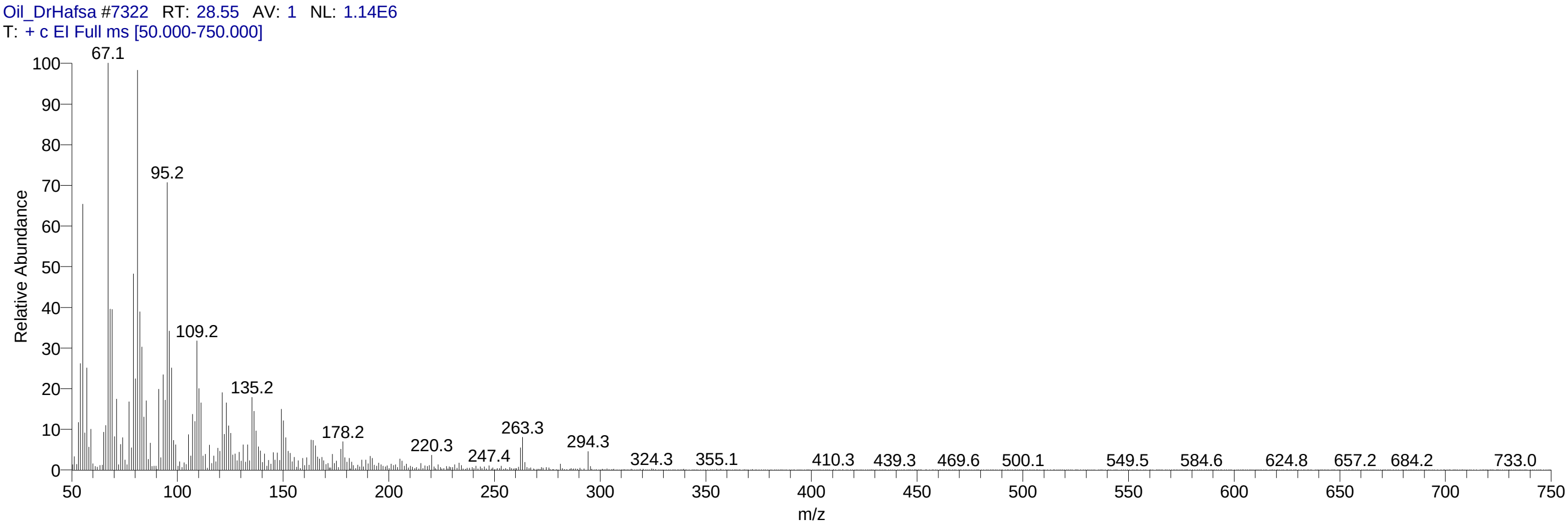

RT	Compound Name	Area %	MF	Molecular Formula	Molecular Weight	Cas #	Library
28.55	9,12-OCTADECADIENOIC ACID (Z,Z)-, METHYL ESTER	1.45	869	C19H34O2	294	112-63-0	WileyRegistry8e
28.55	7,10-Octadecadienoic acid, methyl ester	1.45	879	C19H34O2	294	56554-24-6	mainlib
28.55	8,11-Octadecadienoic acid, methyl ester	1.45	861	C19H34O2	294	56599-58-7	mainlib
28.55	8,11-OCTADECADIENOIC ACID, METHYL ESTER	1.45	861	C19H34O2	294	56599-58-7	WileyRegistry8e
28.55	7,10-OCTADECADIENOIC ACID, METHYL ESTER	1.45	877	C19H34O2	294	56554-24-6	WileyRegistry8e

Compound Structure | Hit Spectrum

9,12-OCTADECADIENOIC ACID (Z,Z)-, METHYL ESTER
Formula C19H34O2, MW 294, CAS# 112-63-0, Entry# 184280
METHYL (9Z,12Z)-9,12-OCTADECADIENOATE #

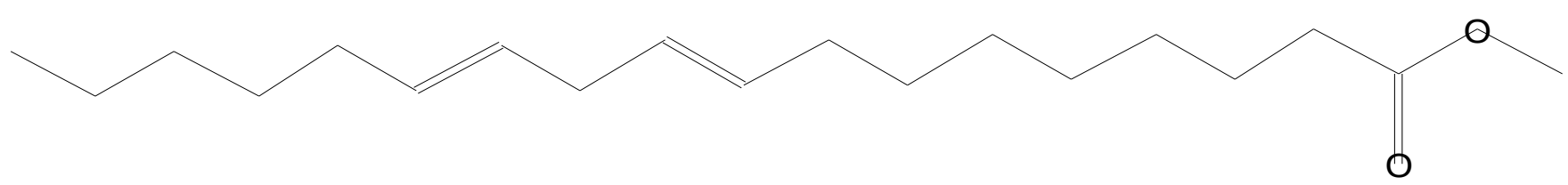

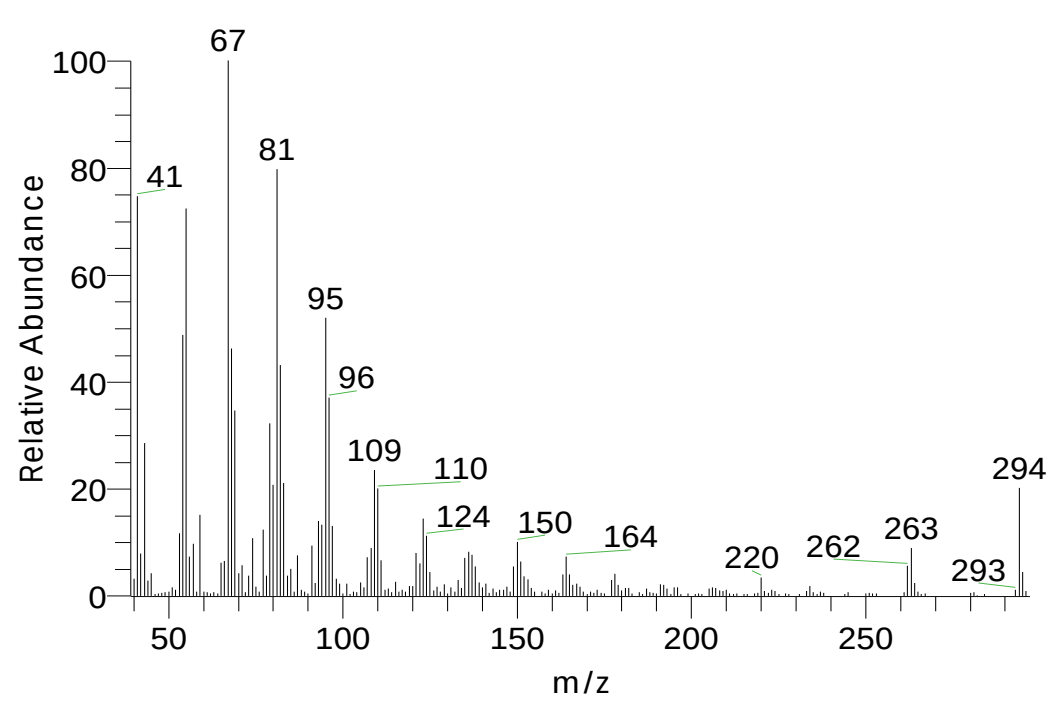

7,10-Octadecadienoic acid, methyl ester
Formula C19H34O2, MW 294, CAS# 56554-24-6, Entry# 32767
Methyl (7E,10E)-7,10-octadecadienoate #

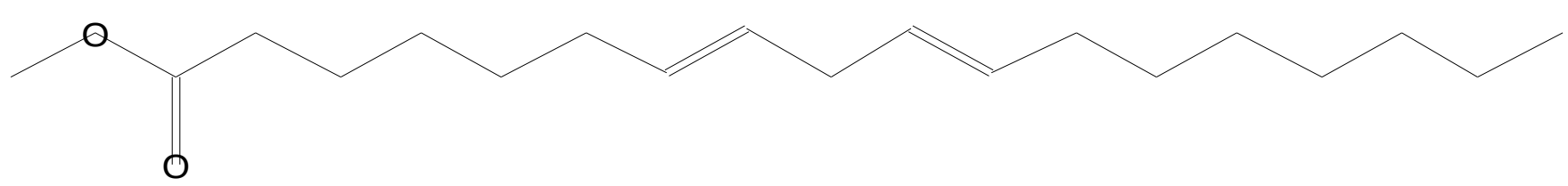

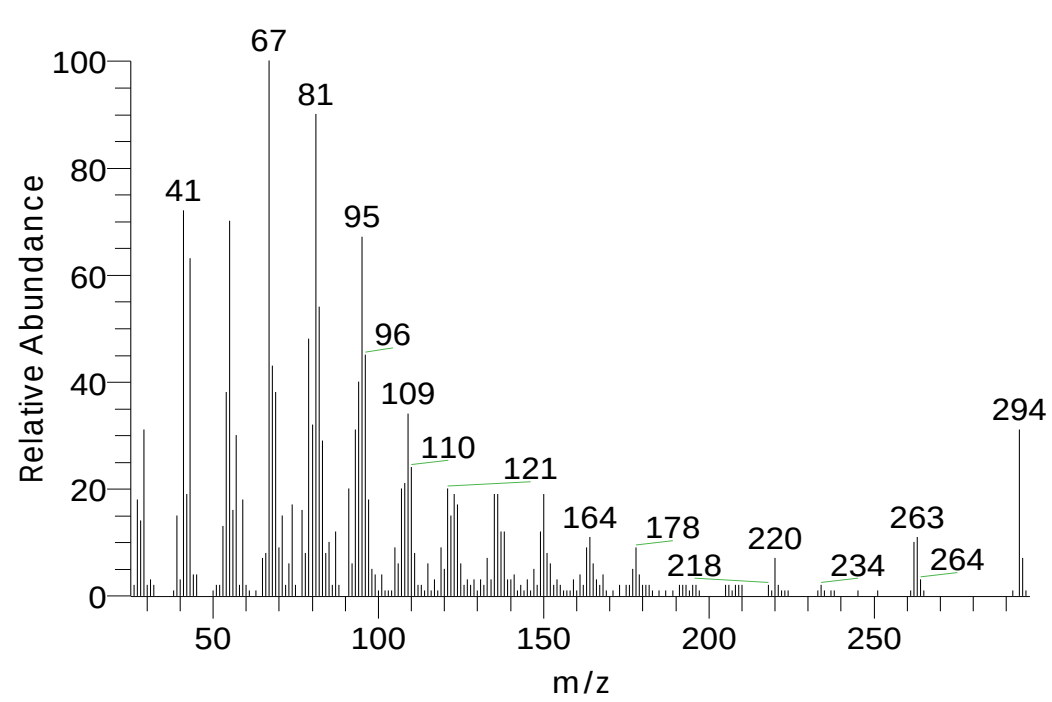

My GC-MS Report

Compound Structure | Hit Spectrum

8,11-Octadecadienoic acid, methyl ester
Formula C19H34O2, MW 294, CAS# 56599-58-7, Entry# 32771
Methyl (8E,11E)-8,11-octadecadienoate #

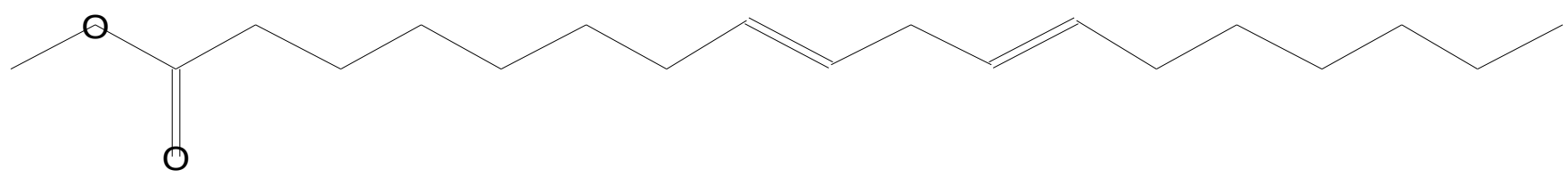

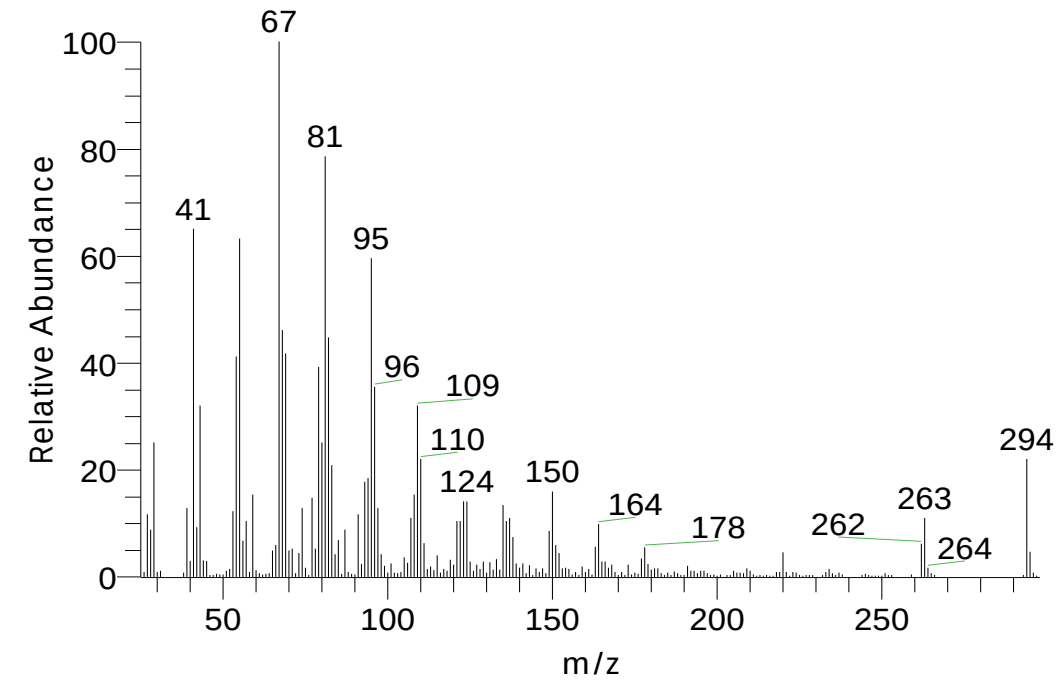

8,11-OCTADECADIENOIC ACID, METHYL ESTER
Formula C19H34O2, MW 294, CAS# 56599-58-7, Entry# 184248
METHYL (8E,11E)-8,11-OCTADECADIENOATE #

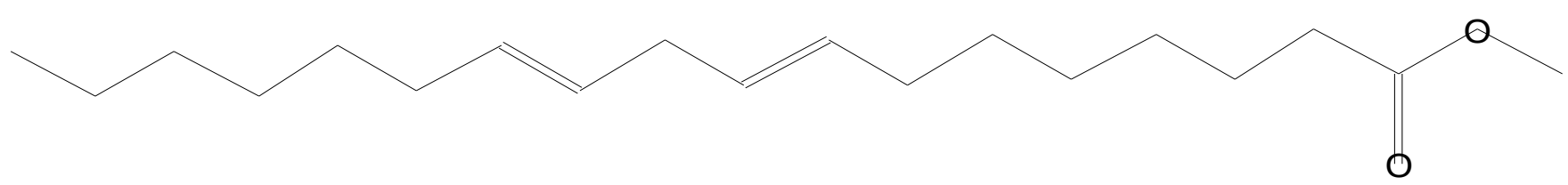

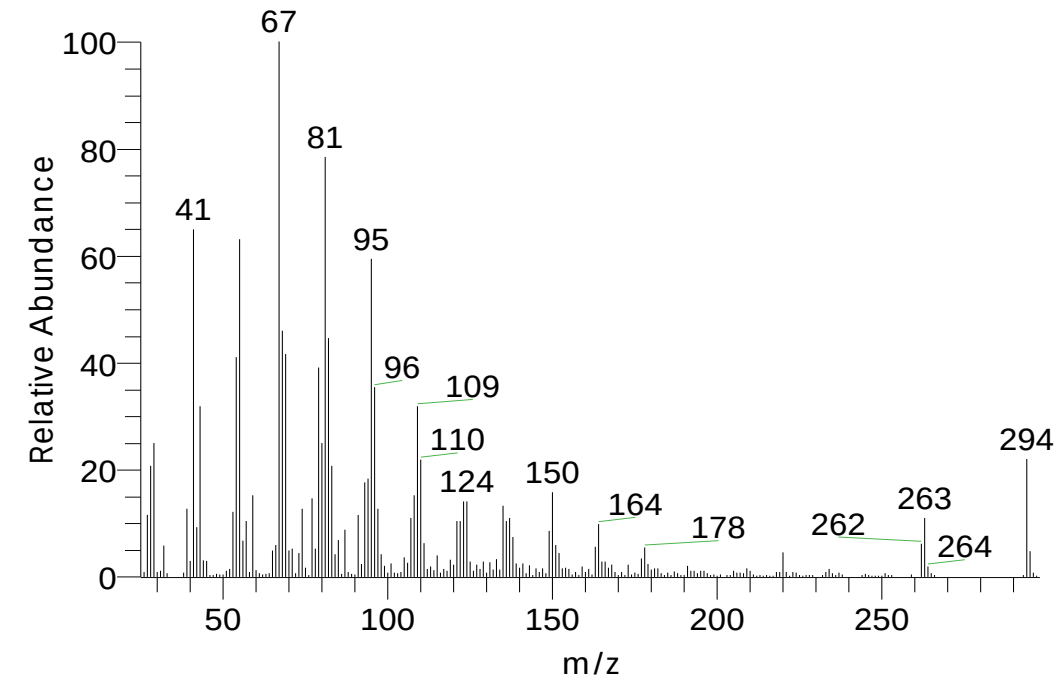

7,10-OCTADECADIENOIC ACID, METHYL ESTER
Formula C19H34O2, MW 294, CAS# 56554-24-6, Entry# 184246
METHYL (7E,10E)-7,10-OCTADECADIENOATE #

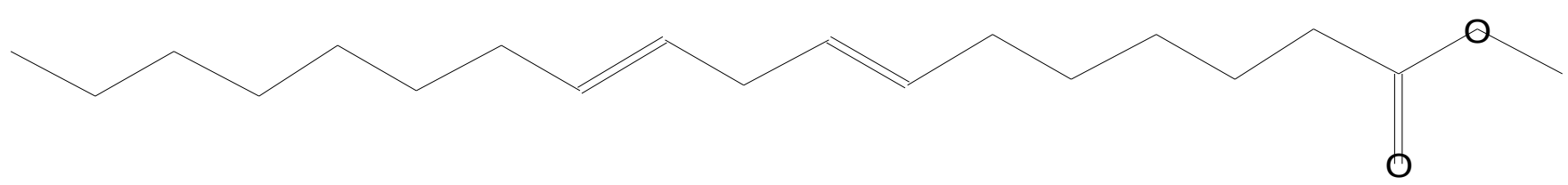

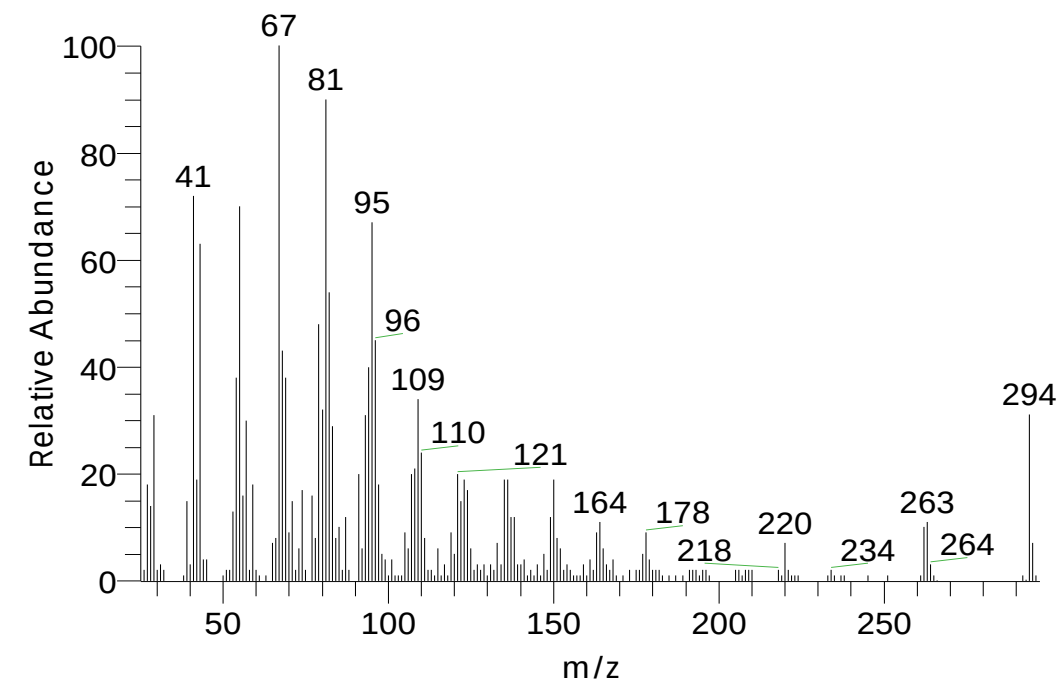

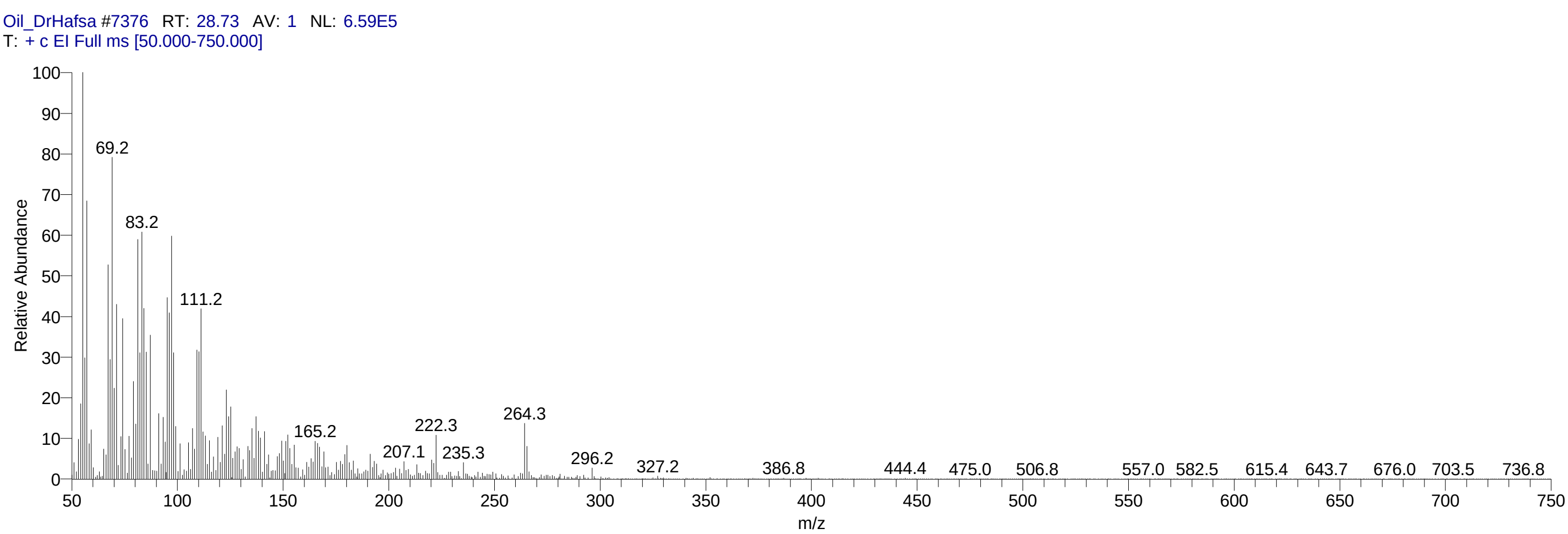

RT	Compound Name	Area %	MF	Molecular Formula	Molecular Weight	Cas #	Library
28.73	9-Octadecenoic acid, methyl ester, (E)-	0.83	833	C19H36O2	296	1937-62-8	replib
28.73	9-OCTADECENOIC ACID (Z)-, METHYL ESTER	0.83	854	C19H36O2	296	112-62-9	WileyRegistry8e

My GC-MS Report

RT	Compound Name	Area %	MF	Molecular Formula	Molecular Weight	Cas #	Library
28.73	4-Octadecenoic acid, methyl ester	0.83	818	C19H36O2	296	56555-10-3	mainlib
28.73	4-OCTADECENOIC ACID, METHYL ESTER	0.83	818	C19H36O2	296	56555-10-3	WileyRegistry8e
28.73	trans-13-Octadecenoic acid, methyl ester	0.83	833	C19H36O2	296	NA	mainlib

Compound Structure | Hit Spectrum

9-Octadecenoic acid, methyl ester, (E)-
Formula C19H36O2, MW 296, CAS# 1937-62-8, Entry# 4987
Elaidic acid, methyl ester

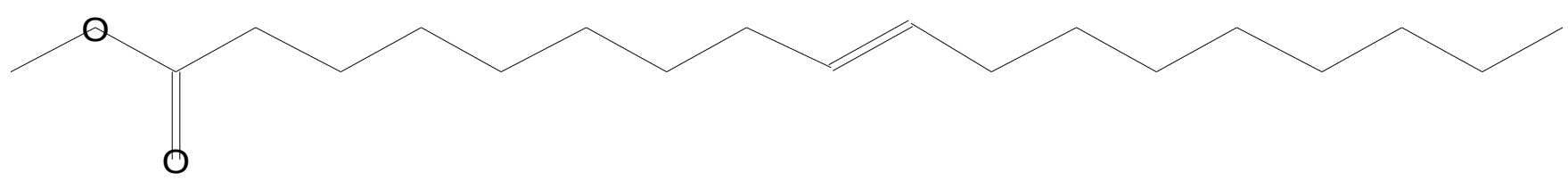

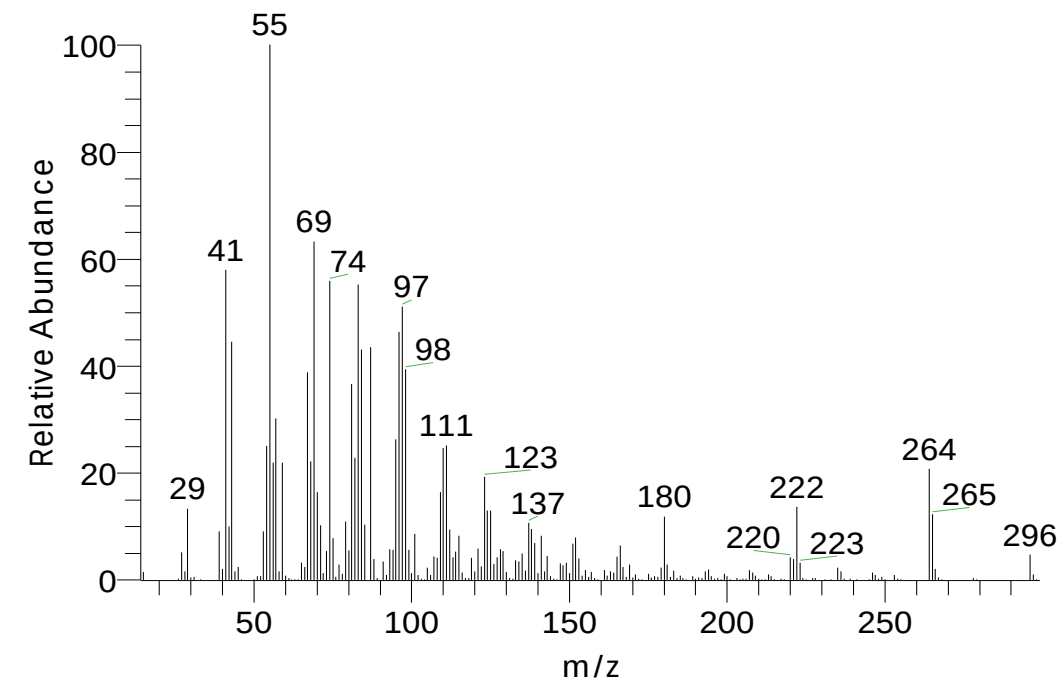

9-OCTADECENOIC ACID (Z)-, METHYL ESTER
Formula C19H36O2, MW 296, CAS# 112-62-9, Entry# 186150
9-OCTADECENOIC ACID, METHYL ESTER

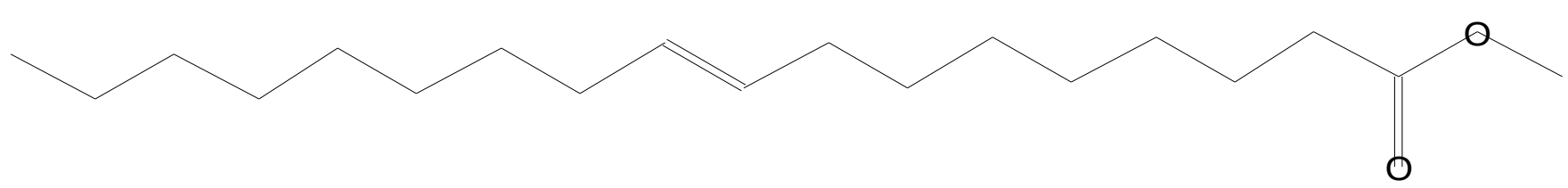

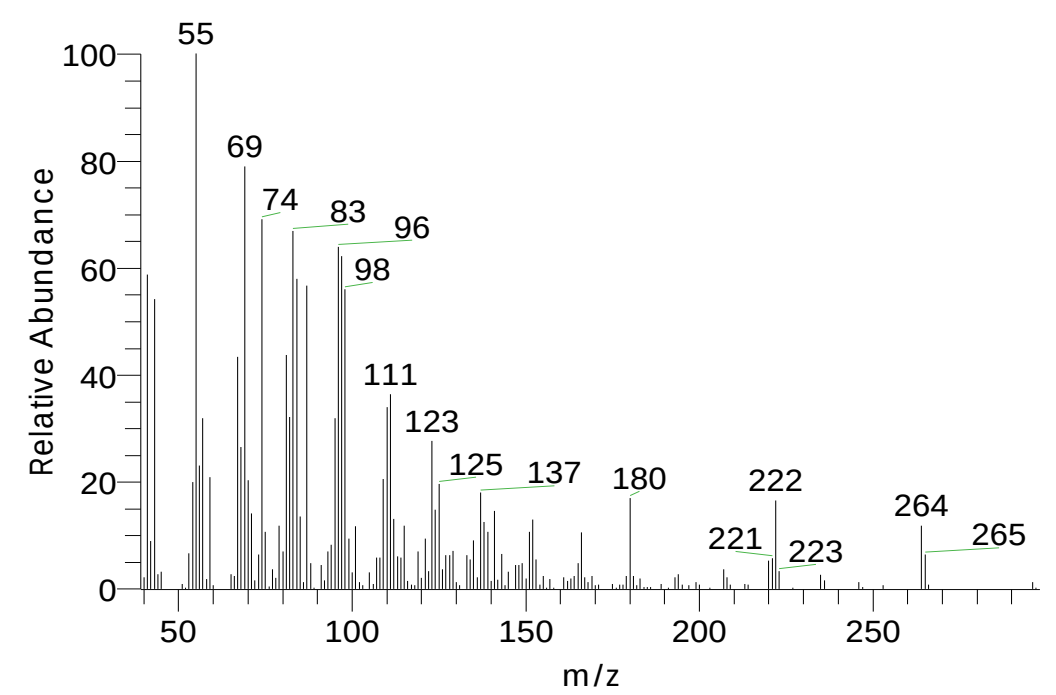

4-Octadecenoic acid, methyl ester
Formula C19H36O2, MW 296, CAS# 56555-10-3, Entry# 44543
Methyl (4E)-4-octadecenoate #

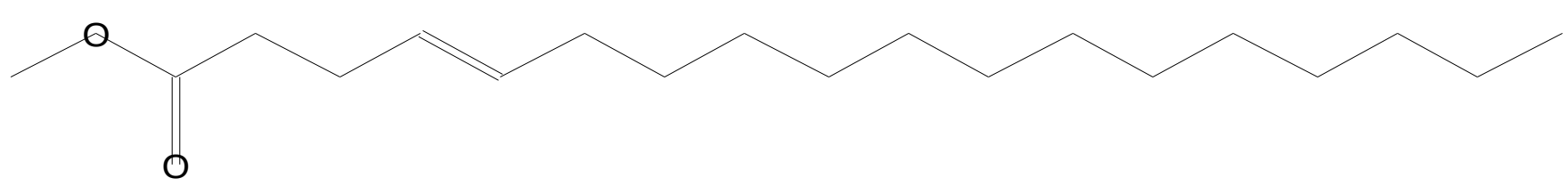

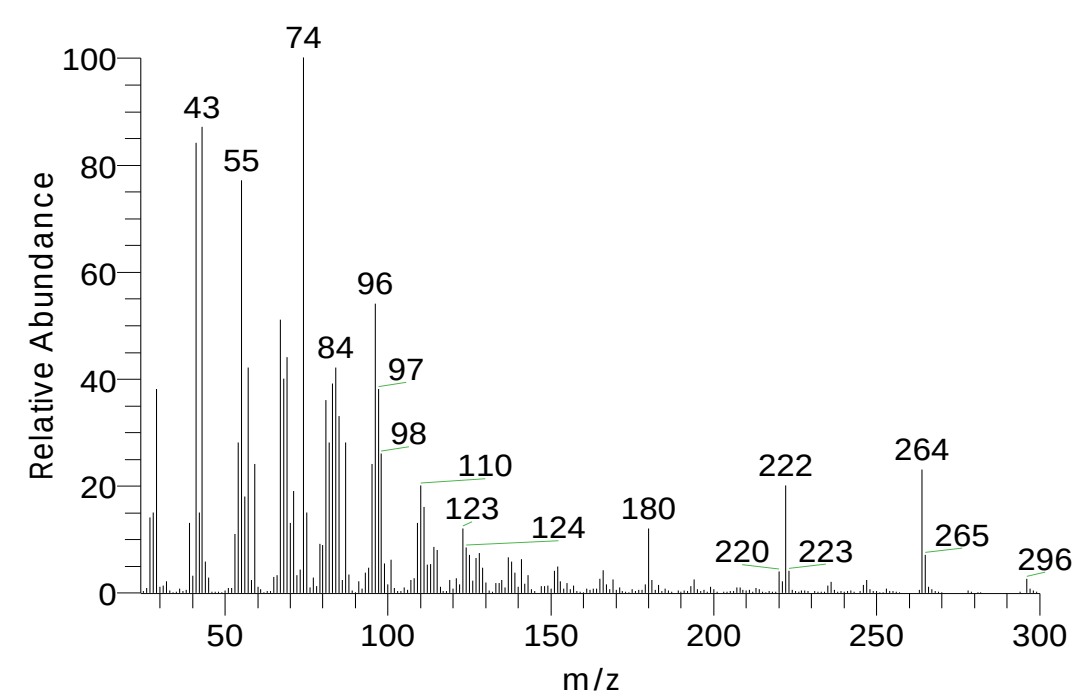

4-OCTADECENOIC ACID, METHYL ESTER
Formula C19H36O2, MW 296, CAS# 56555-10-3, Entry# 186129
METHYL OCTADEC-4-ENOATE

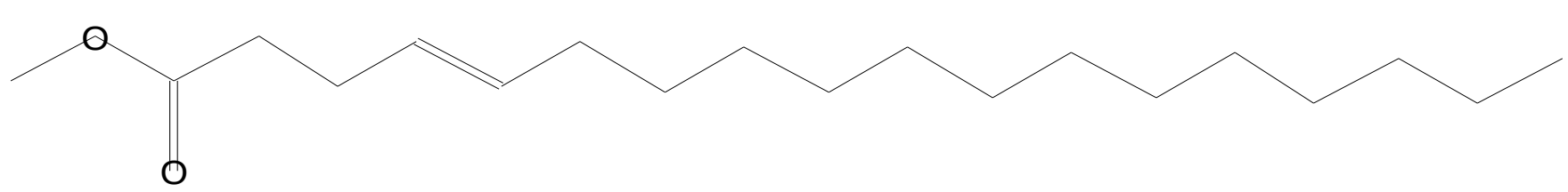

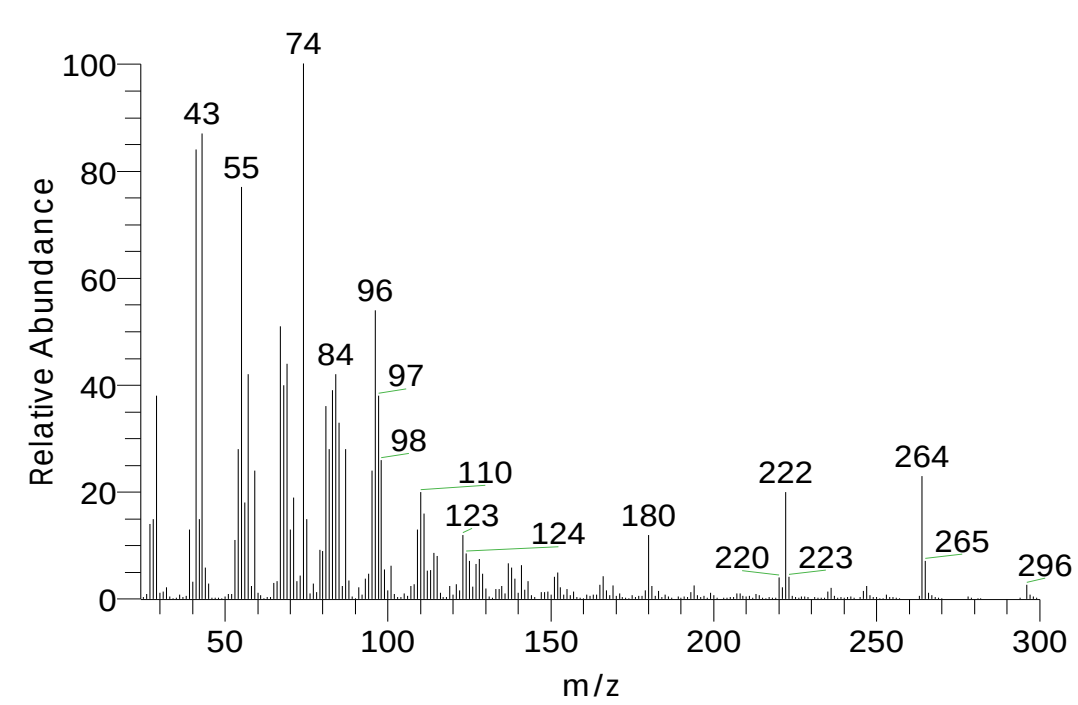

My GC-MS Report

Compound Structure | Hit Spectrum

trans-13-Octadecenoic acid, methyl ester
Formula C19H36O2, MW 296, CAS# NA, Entry# 20067
$:28OPLQDSJPOHPOSZ-VOTSOKGWSA-N

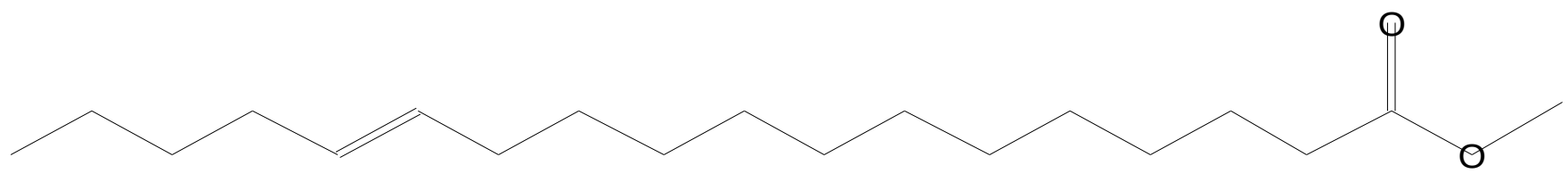

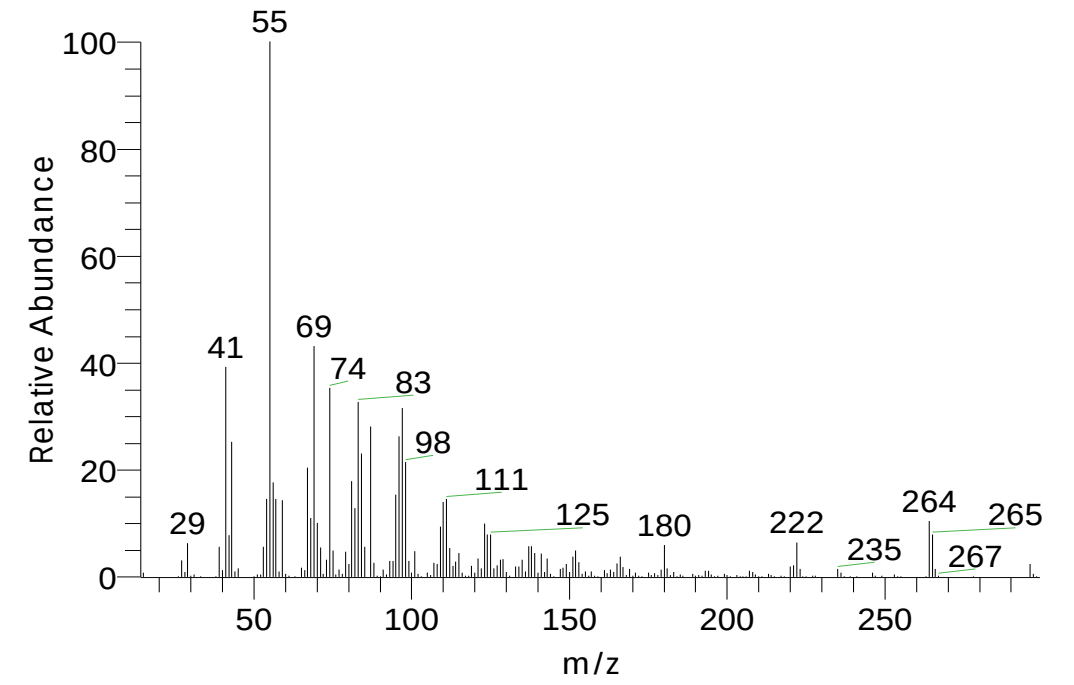

Oil_DrHafsa #7405 RT: 28.83 AV: 1 NL: 3.61E5
T: + c EI Full ms [50.000-750.000]

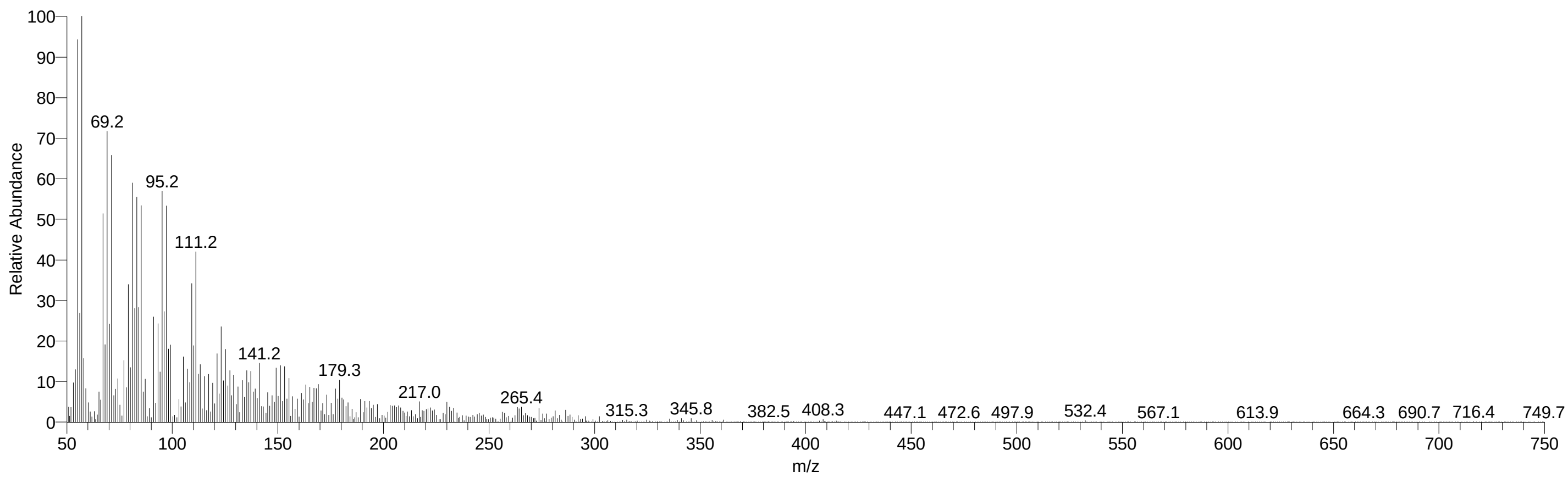

RT	Compound Name	Area %	MF	Molecular Formula	Molecular Weight	Cas #	Library
28.83	1-Heptatriacotanol	0.27	813	C37H76O	536	105794-58-9	mainlib
28.83	14-á-H-PREGNA	0.27	811	C21H36	288	NA	WileyRegistry8e
28.83	E,E,Z-1,3,12-Nonadecatriene-5,14-diol	0.27	784	C19H34O2	294	NA	mainlib
28.83	HAHNFETT	0.27	764	N/A	0	NA	WileyRegistry8e
28.83	HAHNFETT	0.27	764	N/A	0	NA	WileyRegistry8e

Compound Structure | Hit Spectrum

1-Heptatriacotanol
Formula C37H76O, MW 536, CAS# 105794-58-9, Entry# 7279
1-Heptatriacontanol #

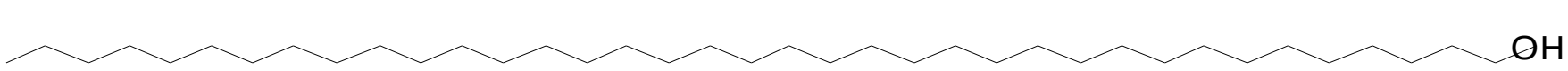

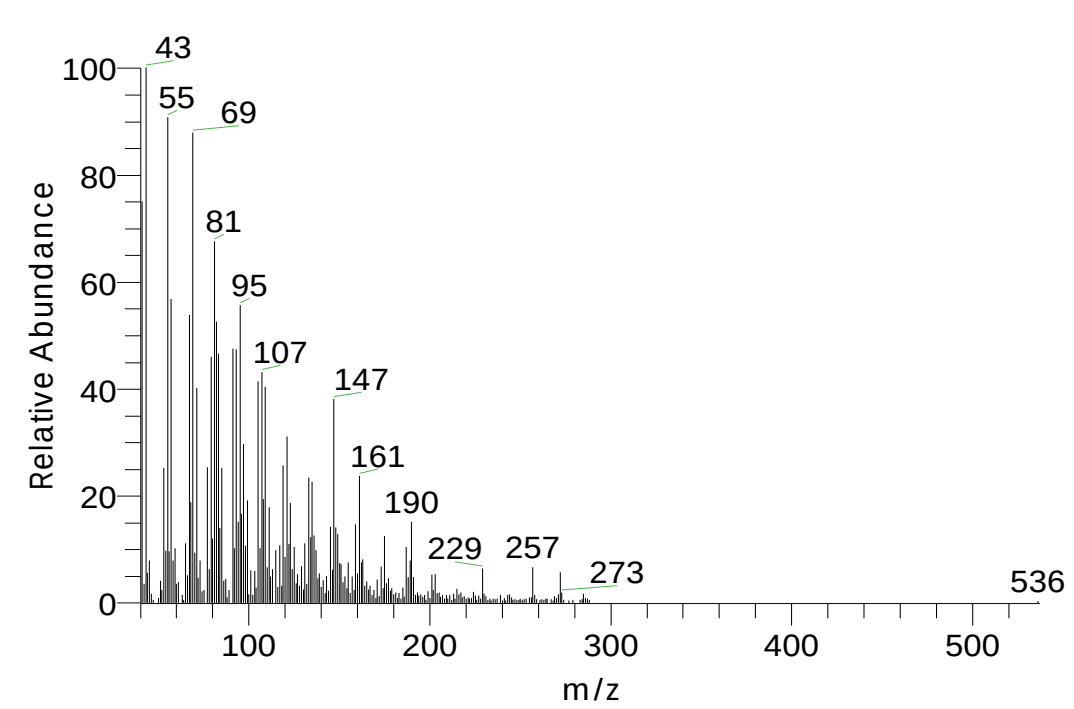

My GC-MS Report

Compound Structure | Hit Spectrum

14-á-H-PREGNA
Formula C21H36, MW 288, CAS# NA, Entry# 178939
14-á-PREGNA

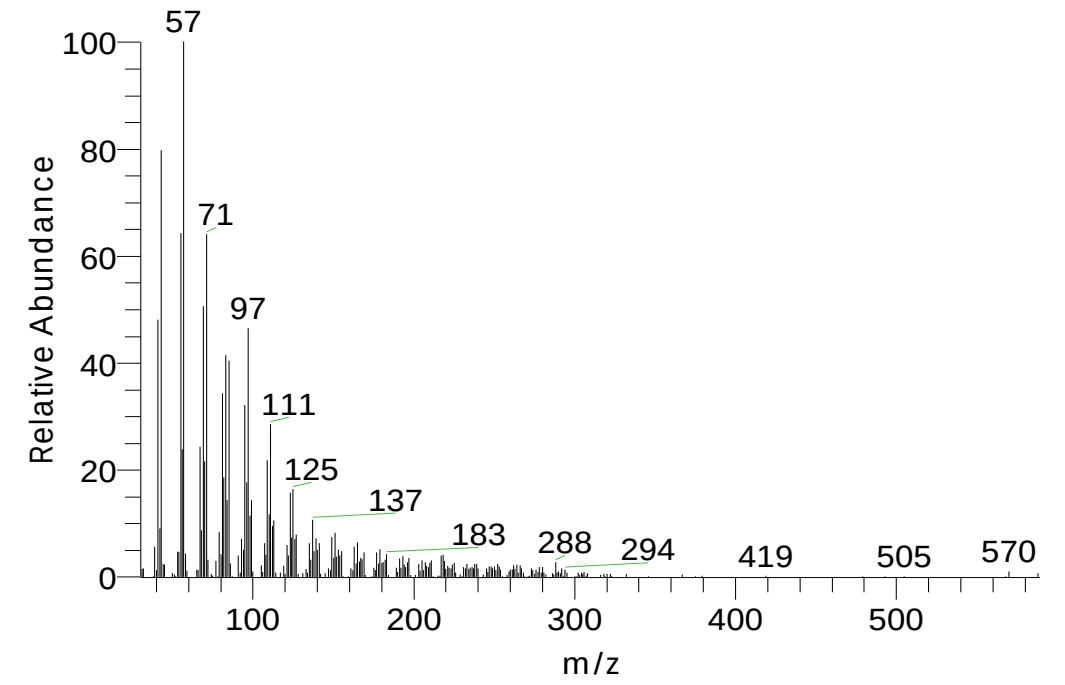

E,E,Z-1,3,12-Nonadecatriene-5,14-diol
Formula C19H34O2, MW 294, CAS# NA, Entry# 21026
(3E,12Z)-1,3,12-Nonadecatriene-5,14-diol #

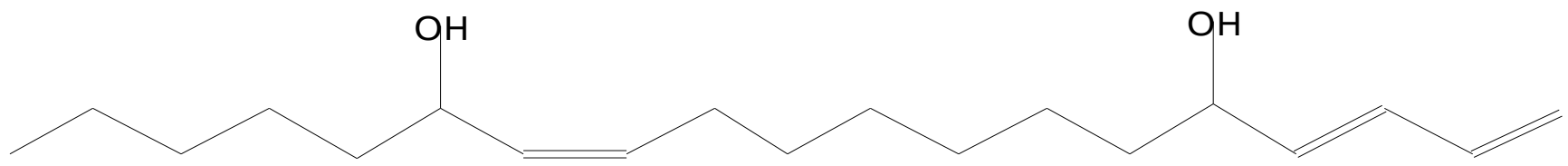

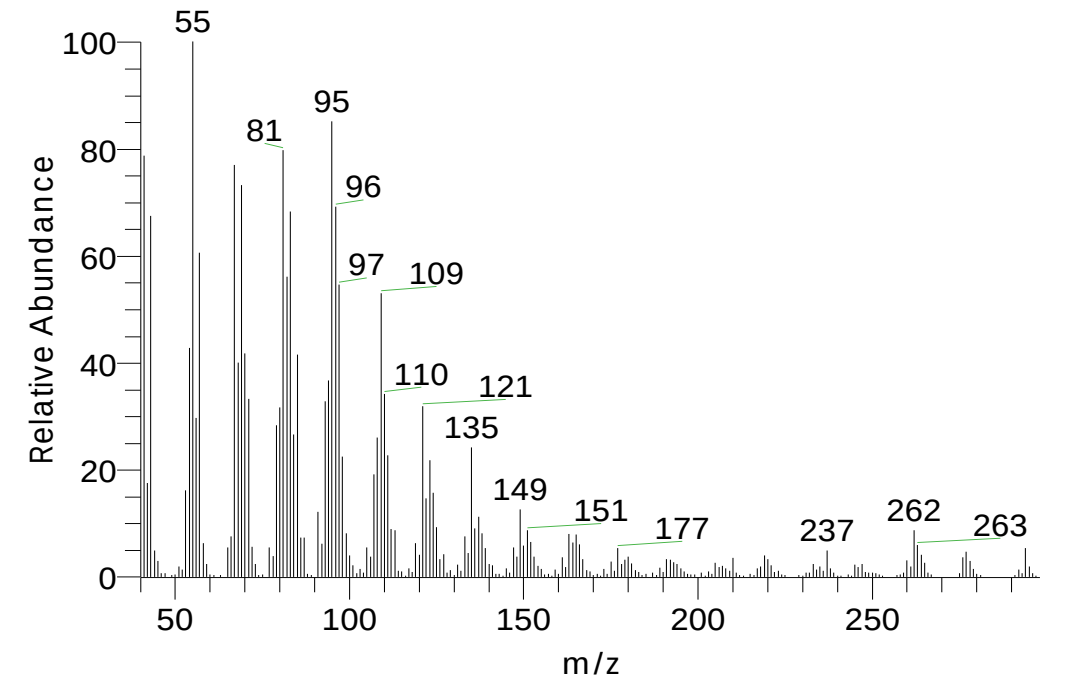

HAHNFETT
Formula , MW 0, CAS# NA, Entry# 305496

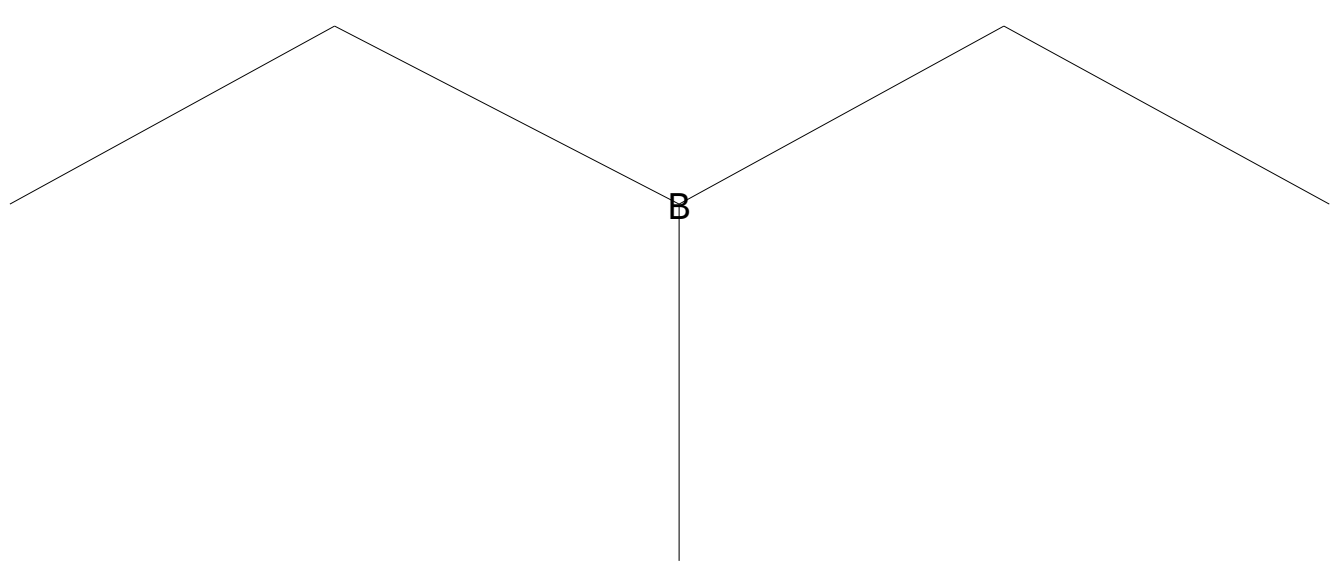

SI 754, RSI 764, WileyRegistry8e, Entry# 305496, CAS# NA, HAHNFETT

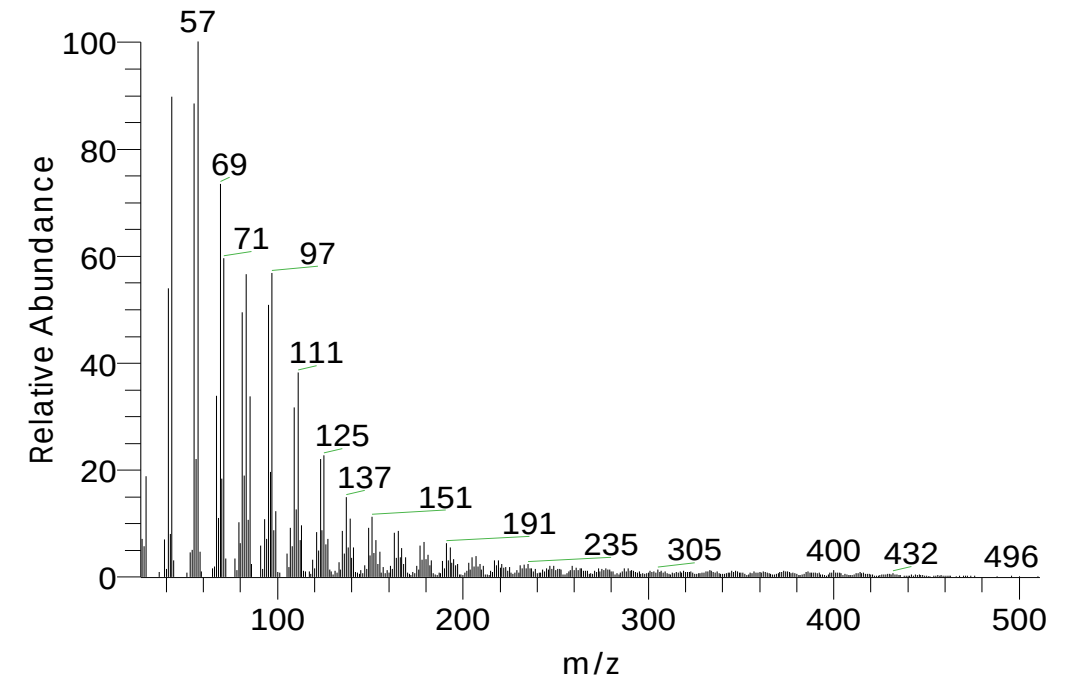

HAHNFETT
Formula , MW 0, CAS# NA, Entry# 391160

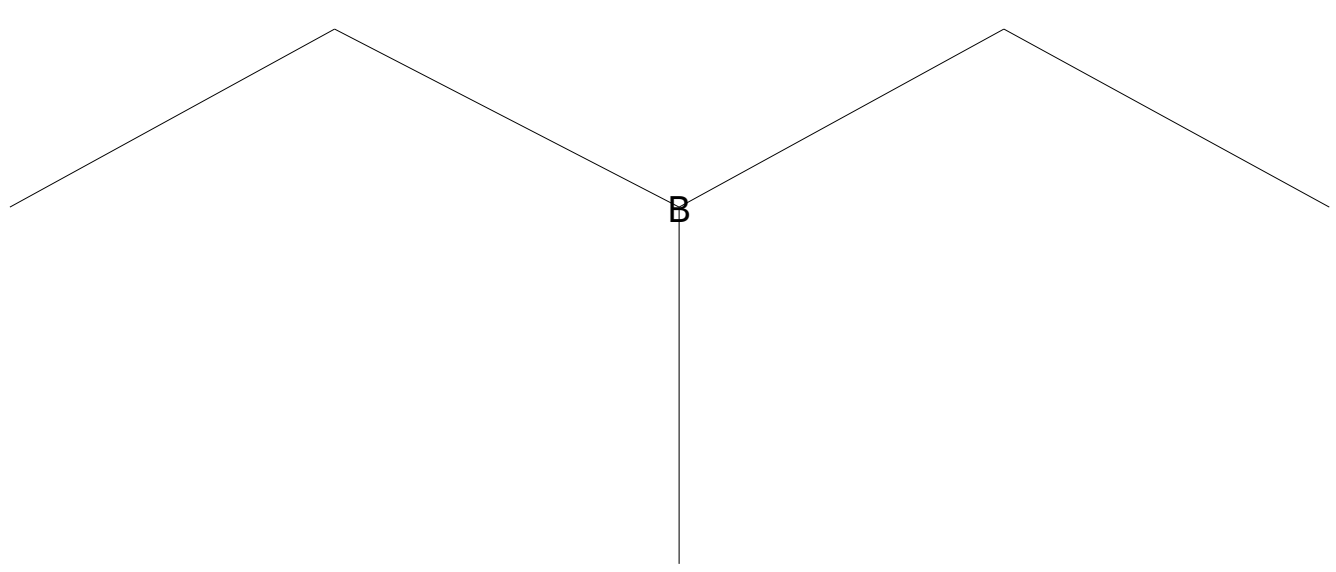

SI 754, RSI 764, WileyRegistry8e, Entry# 391160, CAS# NA, HAHNFETT

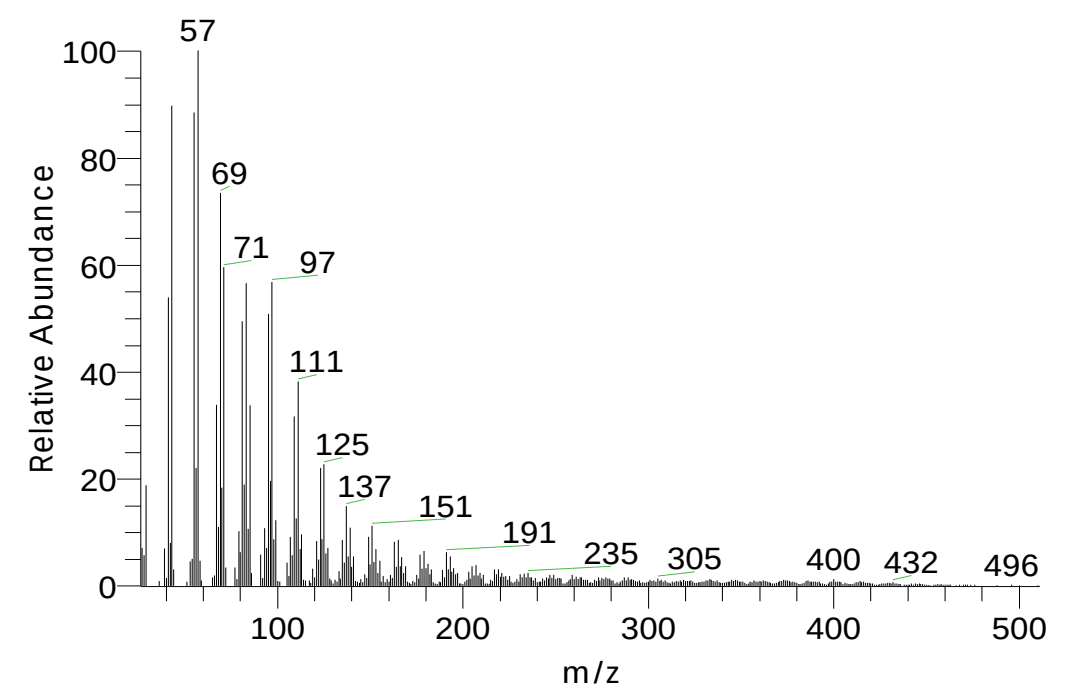

My GC-MS Report

Oil_DrHafsa #7471 RT: 29.05 AV: 1 NL: 4.51E5
T: + c EI Full ms [50.000-750.000]

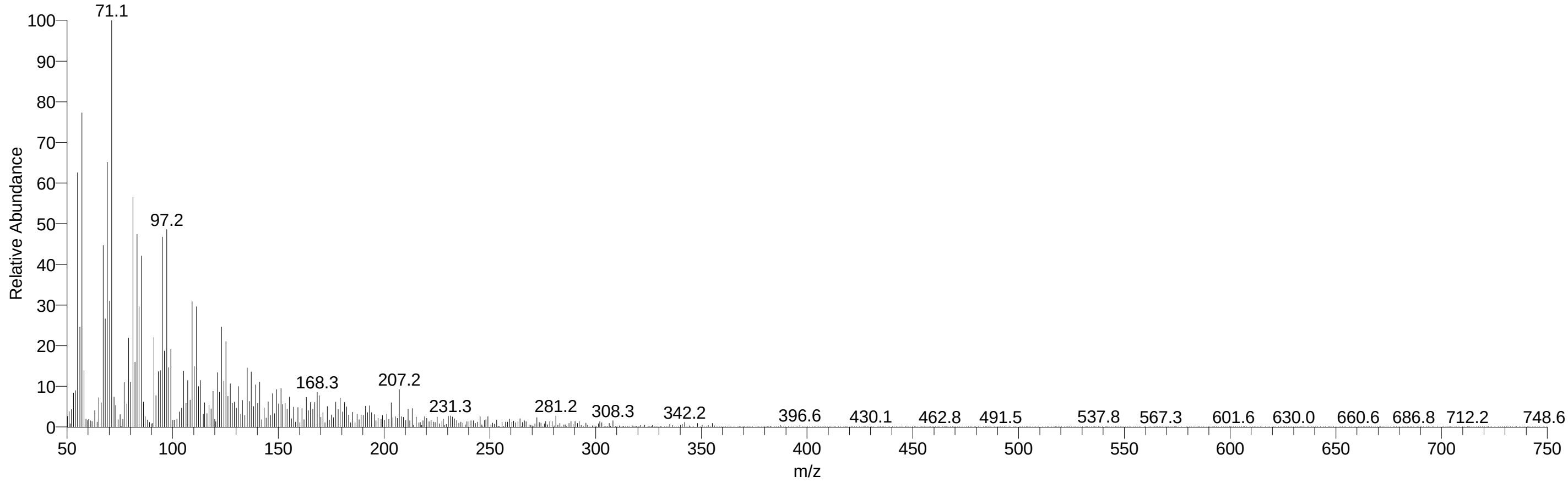

RT	Compound Name	Area %	MF	Molecular Formula	Molecular Weight	Cas #	Library
29.05	ISOCHIAPIN B	0.12	810	C19H22O6	346	NA	WileyRegistry8e
29.05	ISOCHIAPIN B %2<	0.12	810	C19H26O6	350	NA	WileyRegistry8e
29.05	01297107001 TETRANEURIN - A - DIOL	0.12	812	C15H20O5	280	NA	WileyRegistry8e
29.05	1-Heptatriacotanol	0.12	809	C37H76O	536	105794-58-9	mainlib
29.05	DOTRIACONTANE	0.12	806	C32H66	450	544-85-4	WileyRegistry8e

Compound Structure	Hit Spectrum
ISOCHIAPIN B<br>Formula C19H22O6, MW 346, CAS# NA, Entry# 225807	SI 807, RSI 810, WileyRegistry8e, Entry# 225807, CAS# NA, ISOCHIAPIN B

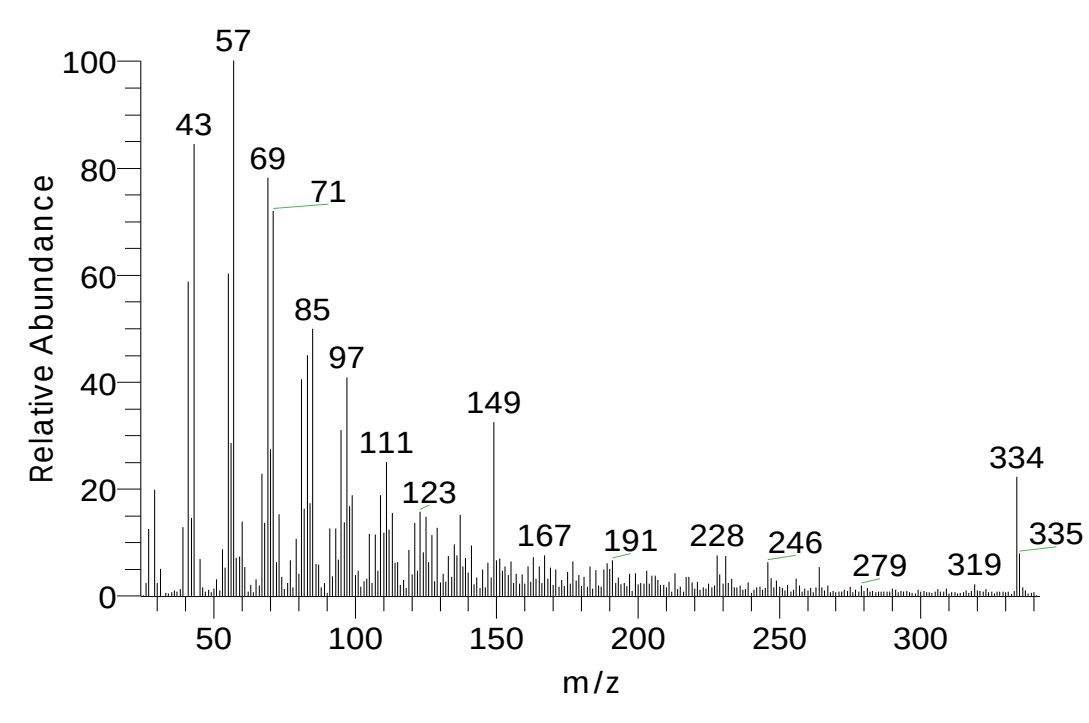

ISOCHIAPIN B %2<
Formula C19H26O6, MW 350, CAS# NA, Entry# 228500

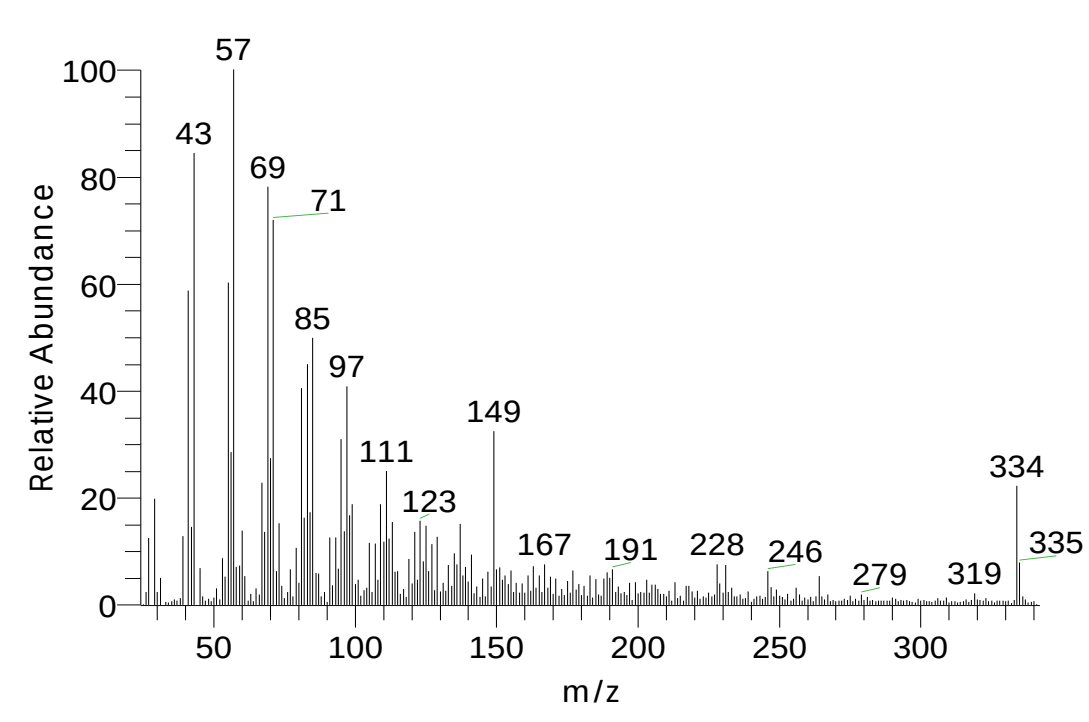

My GC-MS Report

Compound Structure	Hit Spectrum

01297107001 TETRANEURIN - A - DIOL
Formula C15H20O5, MW 280, CAS# NA, Entry# 170378

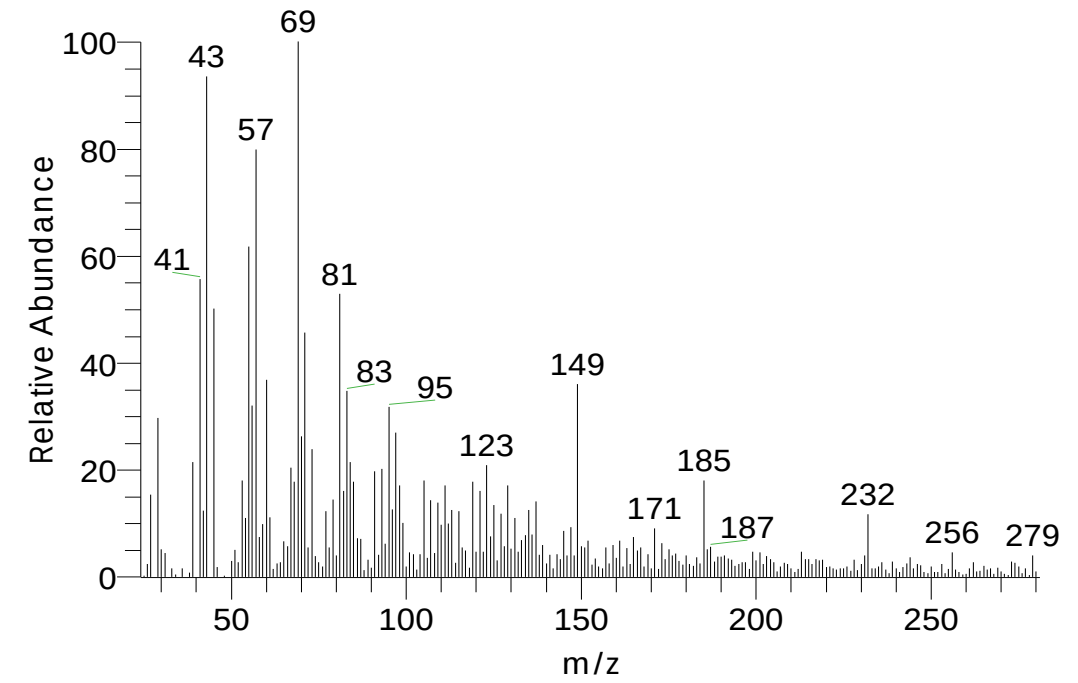

1-Heptatriacotanol
Formula C37H76O, MW 536, CAS# 105794-58-9, Entry# 7279
1-Heptatriacontanol #

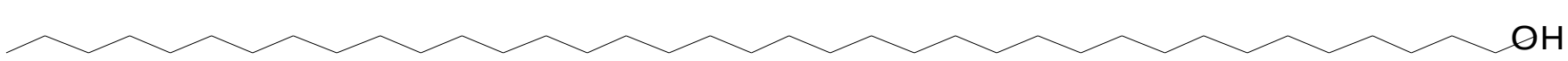

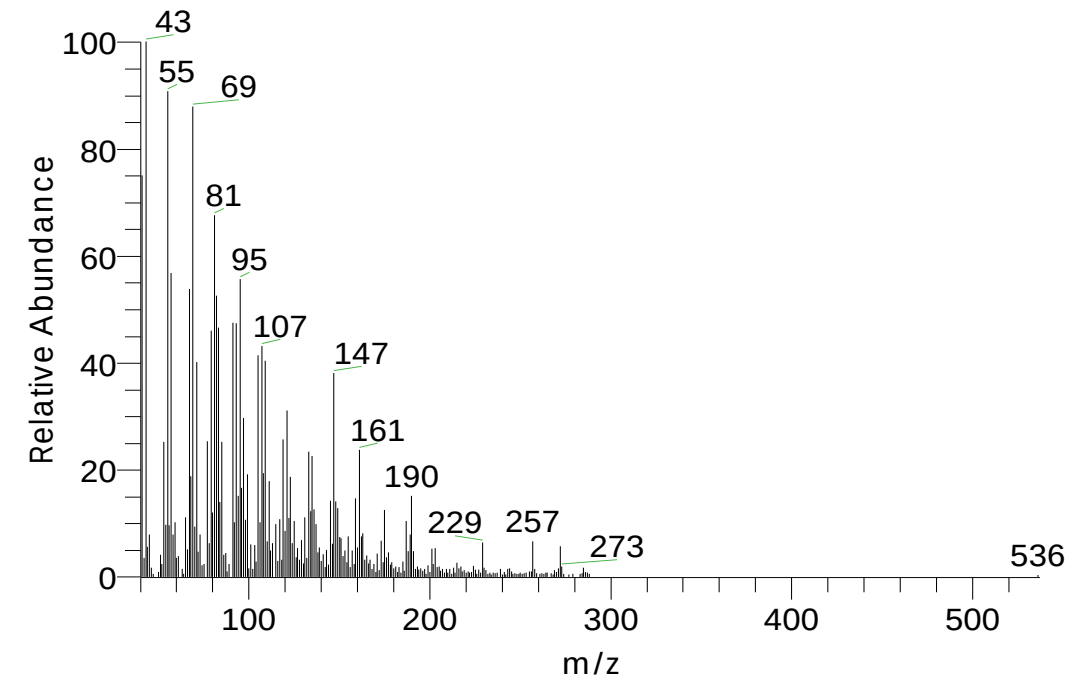

DOTRIACONTANE
Formula C32H66, MW 450, CAS# 544-85-4, Entry# 274478
AI3-52367

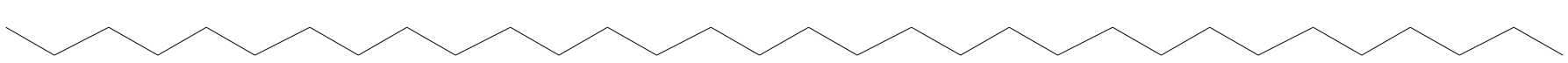

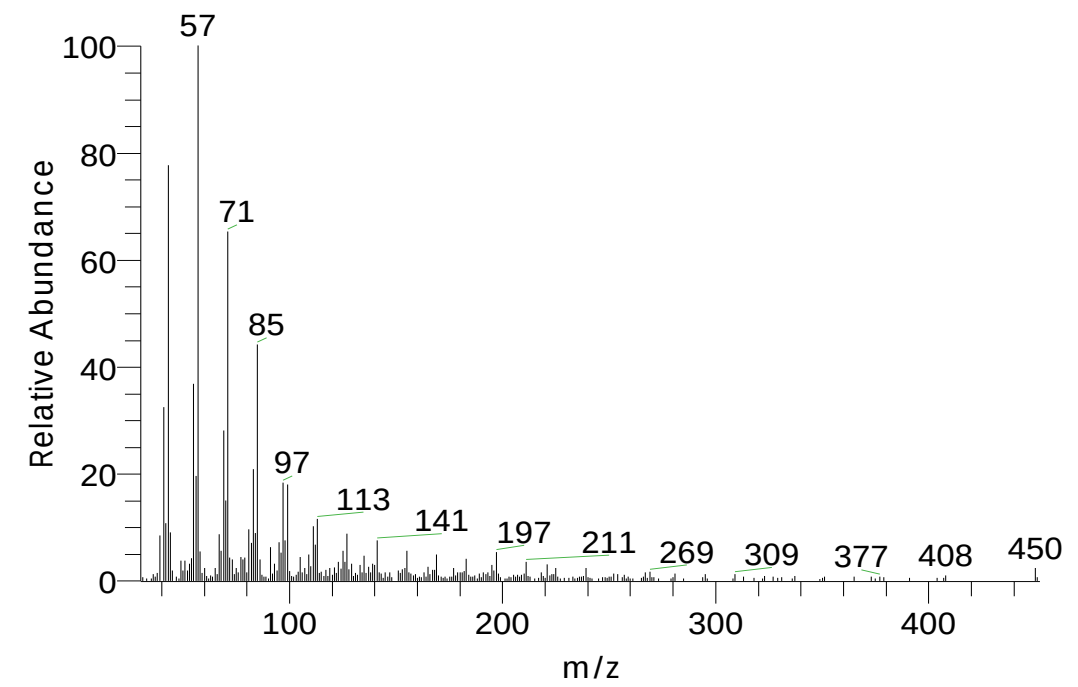

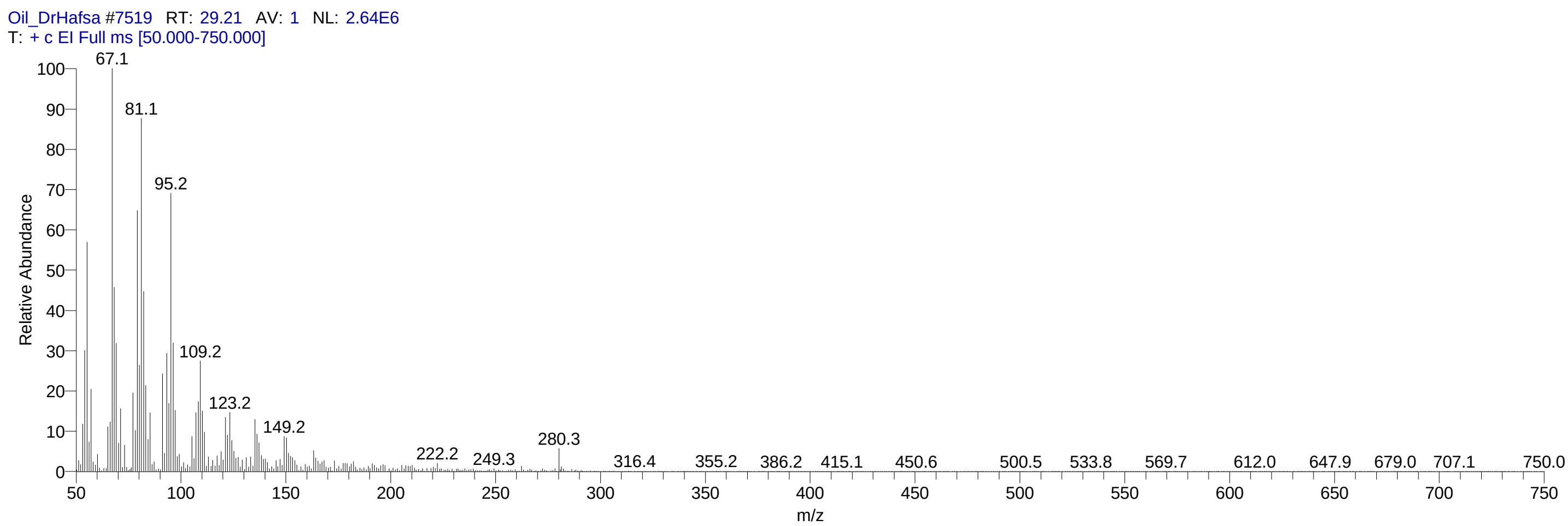

RT	Compound Name	Area %	MF	Molecular Formula	Molecular Weight	Cas #	Library
29.21	9,12-Octadecadienoic acid (Z,Z)-	4.96	870	C18H32O2	280	60-33-3	replib
29.21	9,12-Octadecadienoic acid (Z,Z)-	4.96	880	C18H32O2	280	60-33-3	replib
29.21	ETHYL (9Z,12Z)-9,12-OCTADECADIENOATE #	4.96	884	C20H36O2	308	544-35-4	WileyRegistry8e

My GC-MS Report

RT	Compound Name	Area %	MF	Molecular Formula	Molecular Weight	Cas #	Library
29.21	(Z)-18-Octadec-9-enolide	4.96	849	C18H32O2	280	80060-76-0	mainlib
29.21	HEXADECADIENOIC ACID, METHYL ESTER	4.96	834	C17H30O2	266	29961-54-4	WileyRegistry8e

Compound Structure | Hit Spectrum

9,12-Octadecadienoic acid (Z,Z)-
Formula C18H32O2, MW 280, CAS# 60-33-3, Entry# 8112
cis-9,cis-12-Octadecadienoic acid

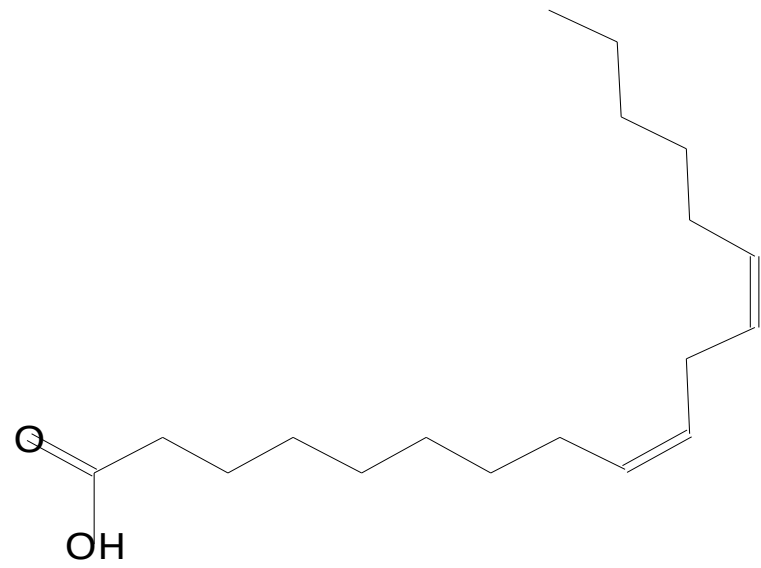

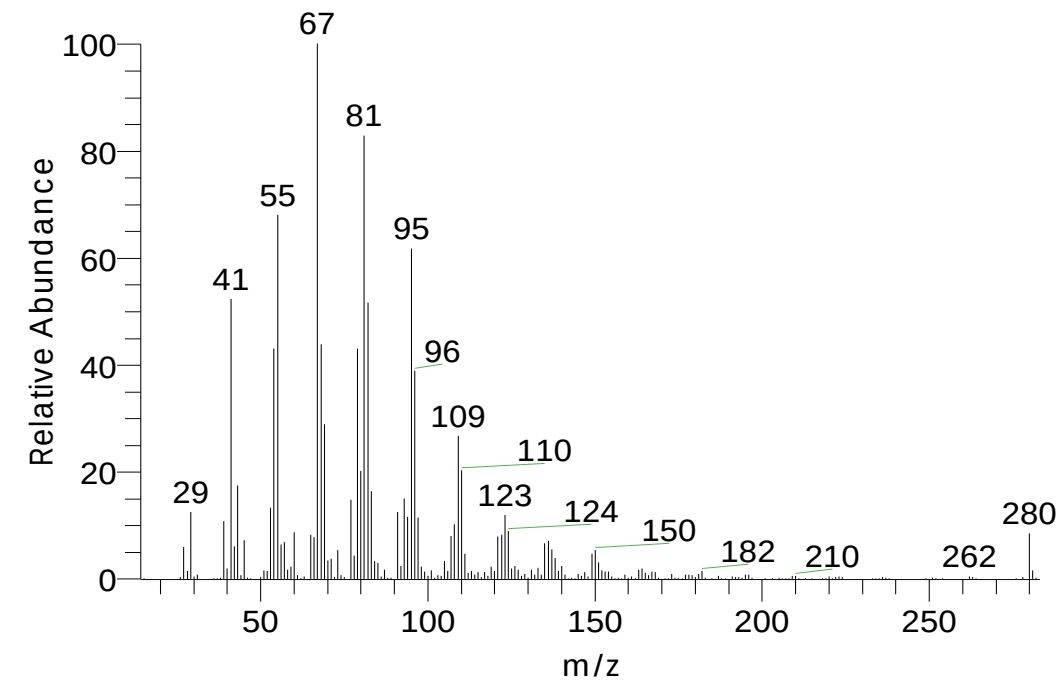

9,12-Octadecadienoic acid (Z,Z)-
Formula C18H32O2, MW 280, CAS# 60-33-3, Entry# 8129
cis-9,cis-12-Octadecadienoic acid

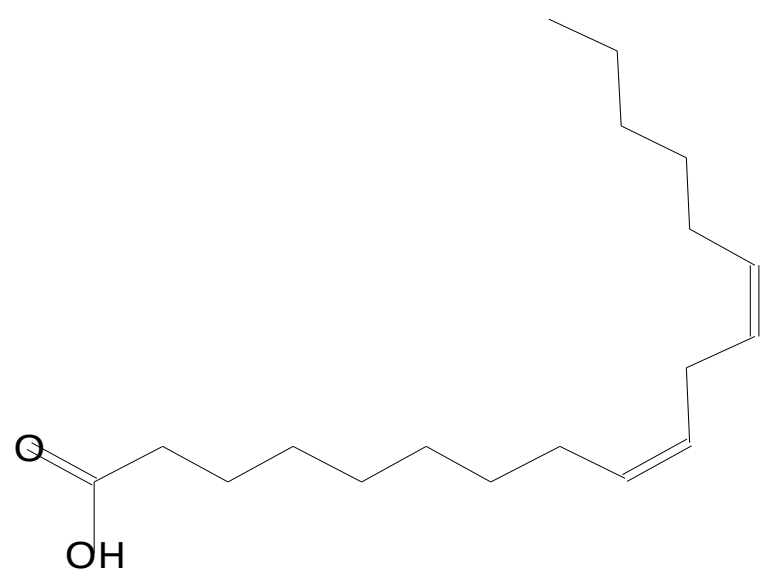

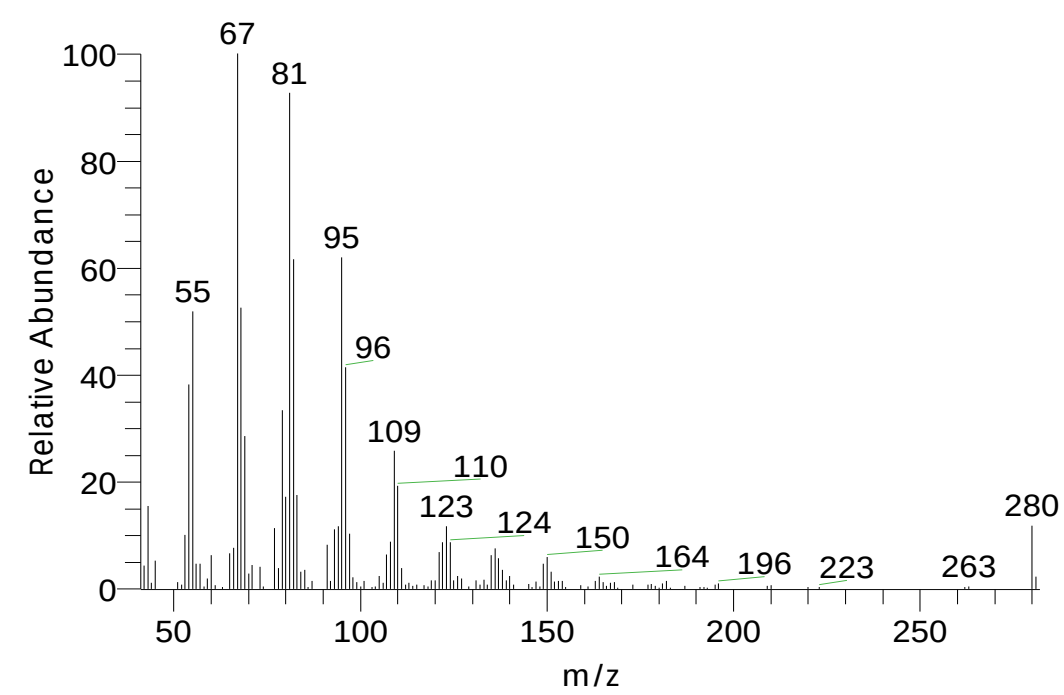

ETHYL (9Z,12Z)-9,12-OCTADECADIENOATE #
Formula C20H36O2, MW 308, CAS# 544-35-4, Entry# 196852
9,12-OCTADECADIENOIC ACID (9Z,12Z)-, ETHYL ESTER

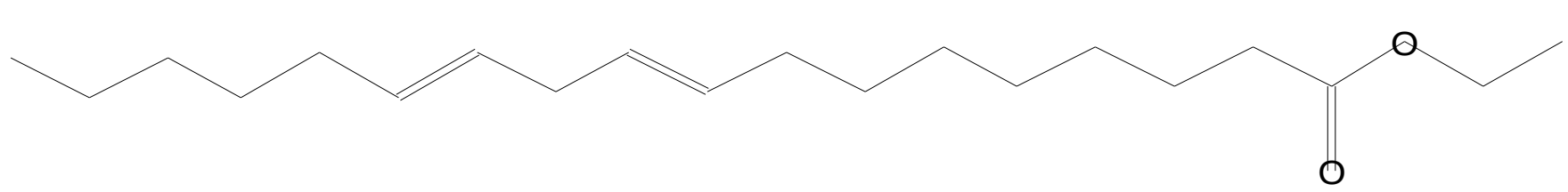

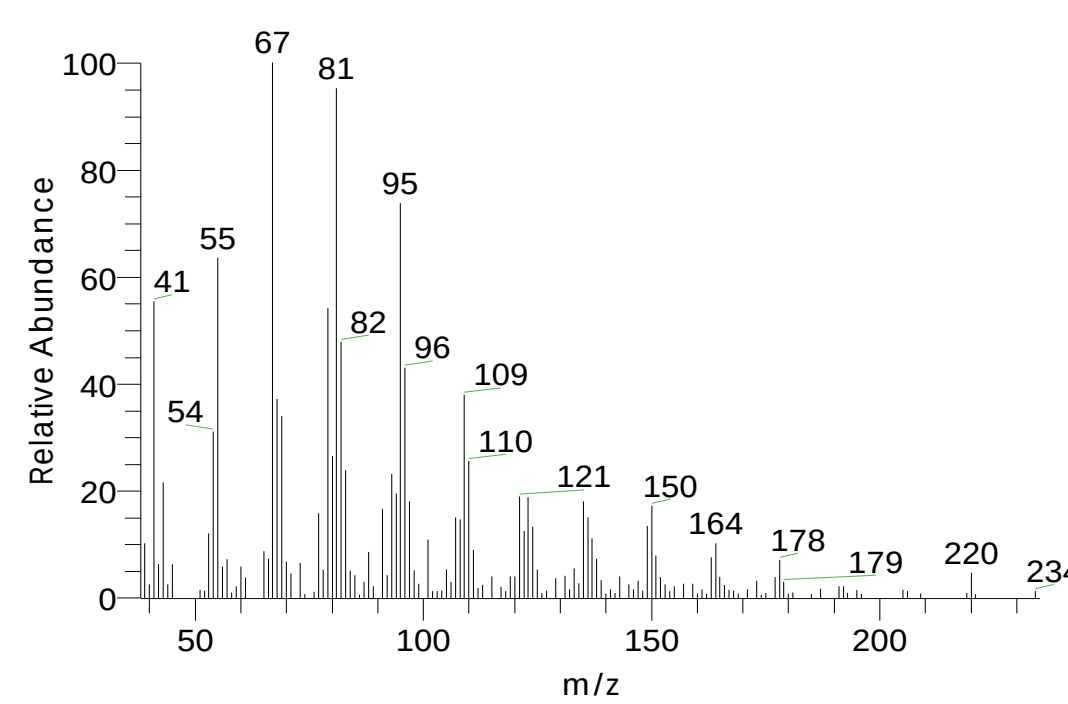

(Z)-18-Octadec-9-enolide
Formula C18H32O2, MW 280, CAS# 80060-76-0, Entry# 51421
Oxacyclononadec-10-en-2-one, (10Z)-

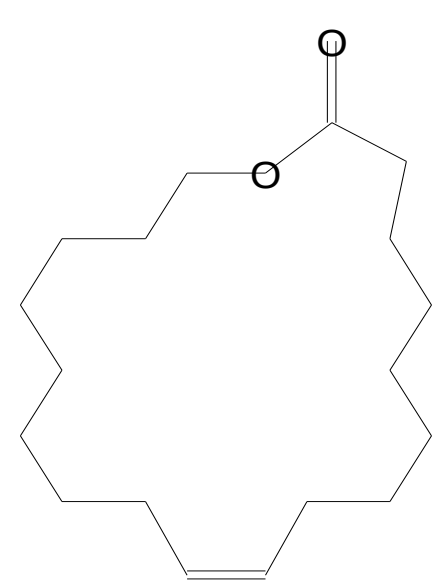

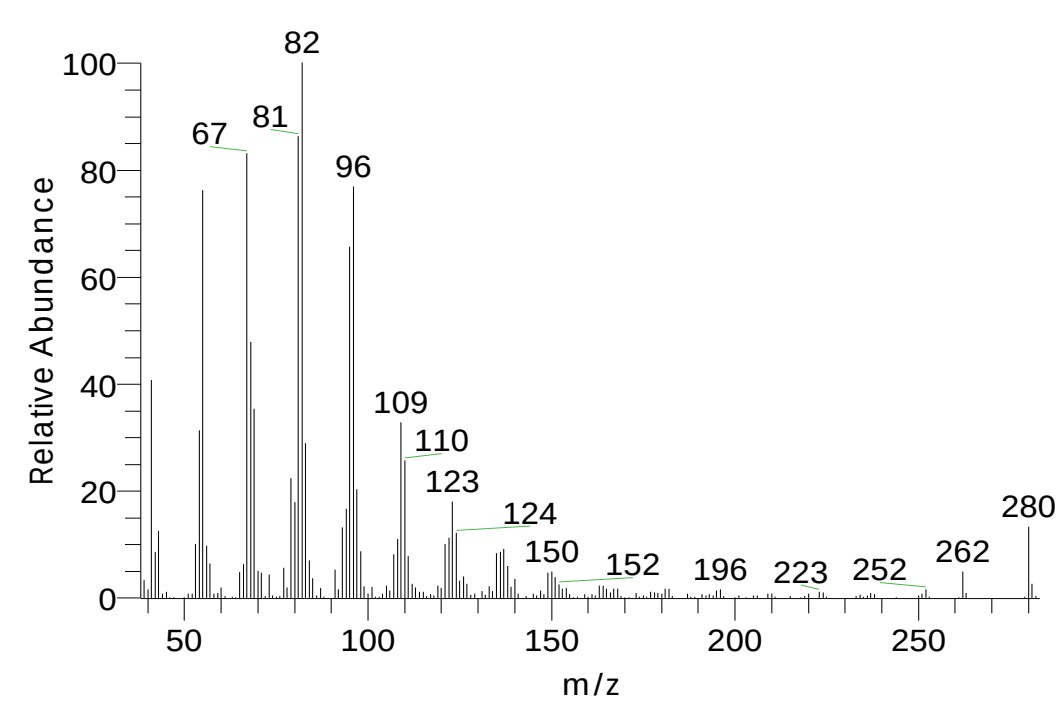

My GC-MS Report

Compound Structure

Hit Spectrum

HEXADECADIENOIC ACID, METHYL ESTER
Formula C17H30O2, MW 266, CAS# 29961-54-4, Entry# 157129
METHYL HEXADECADIENOATE

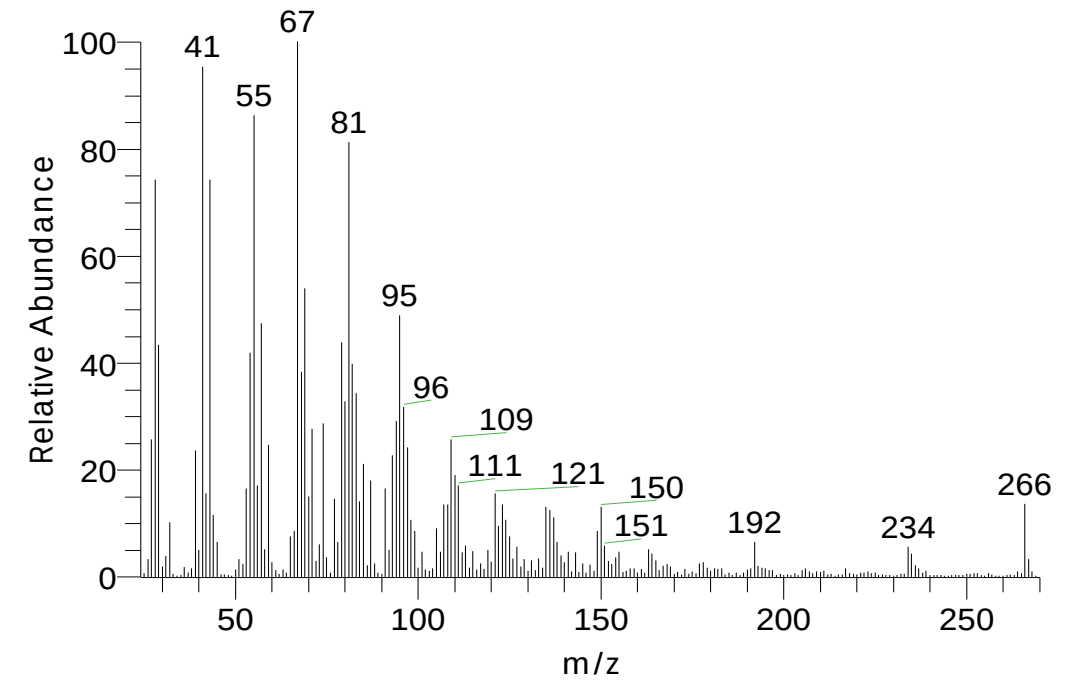

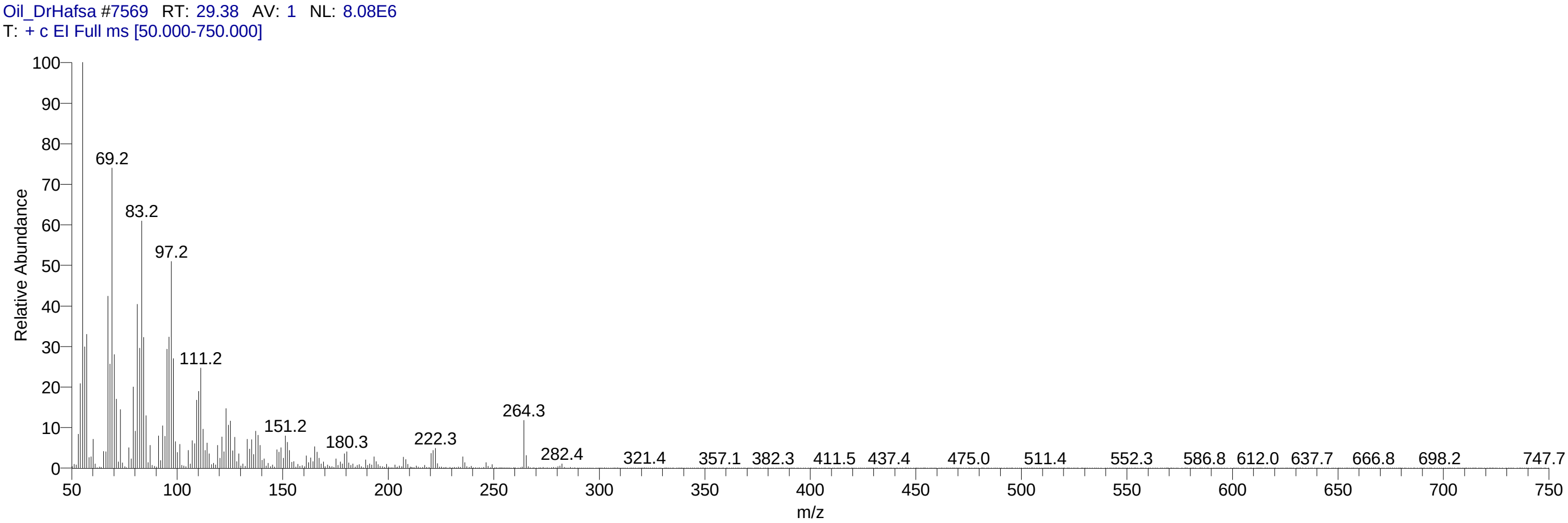

RT	Compound Name	Area %	MF	Molecular Formula	Molecular Weight	Cas #	Library
29.38	trans-13-Octadecenoic acid	11.61	920	C18H34O2	282	693-71-0	mainlib
29.38	cis-13-Octadecenoic acid	11.61	919	C18H34O2	282	13126-39-1	mainlib
29.38	cis-Vaccenic acid	11.61	918	C18H34O2	282	506-17-2	mainlib
29.38	9-Octadecenoic acid, (E)-	11.61	926	C18H34O2	282	112-79-8	replib
29.38	Oleic Acid	11.61	919	C18H34O2	282	112-80-1	replib

Compound Structure

Hit Spectrum

trans-13-Octadecenoic acid
Formula C18H34O2, MW 282, CAS# 693-71-0, Entry# 19306
$:28BDLLSHRIFPDGQB-AATRIKPKSA-N

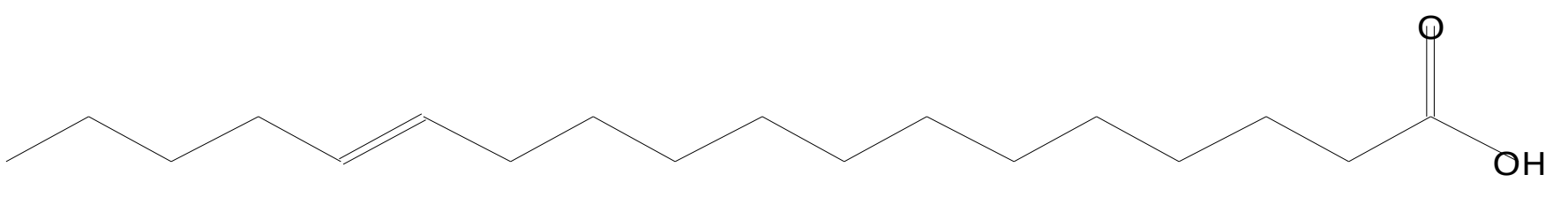

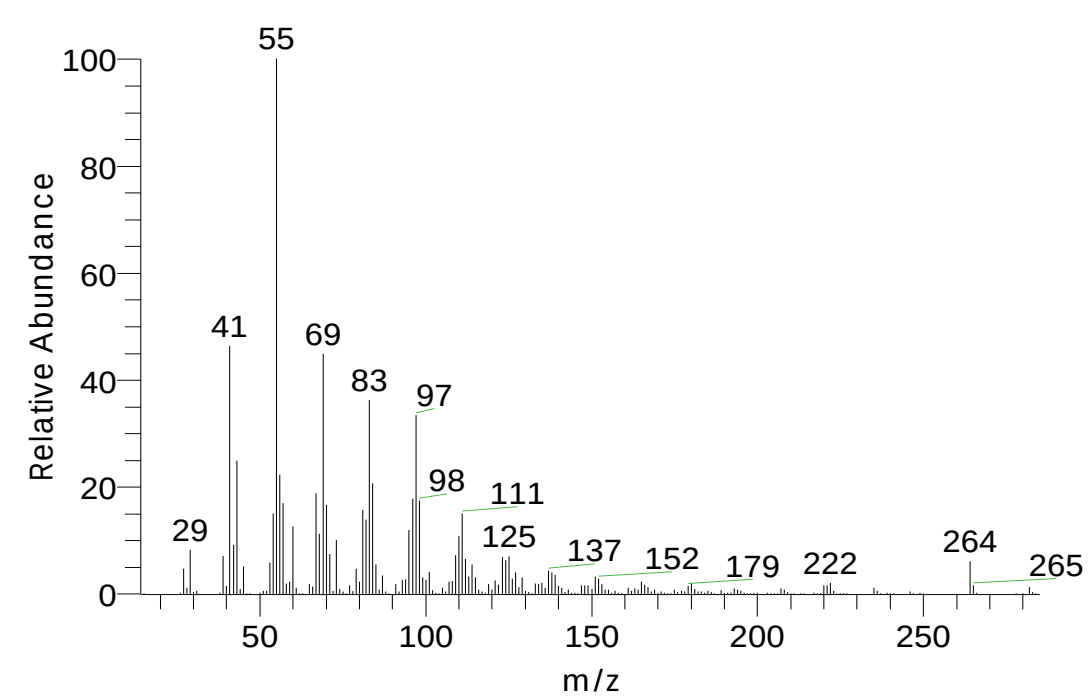

My GC-MS Report

Compound Structure | Hit Spectrum

cis-13-Octadecenoic acid
Formula C18H34O2, MW 282, CAS# 13126-39-1, Entry# 20126
$:28BDLLSHRIFPDGQB-WAYWQWQTSA-N

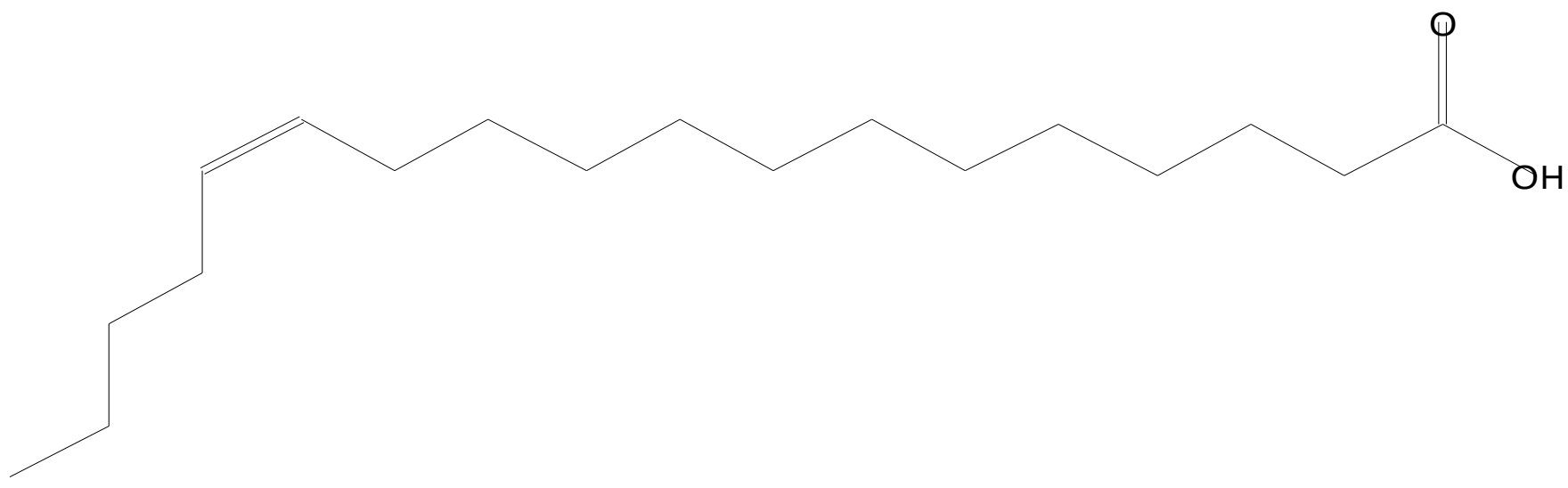

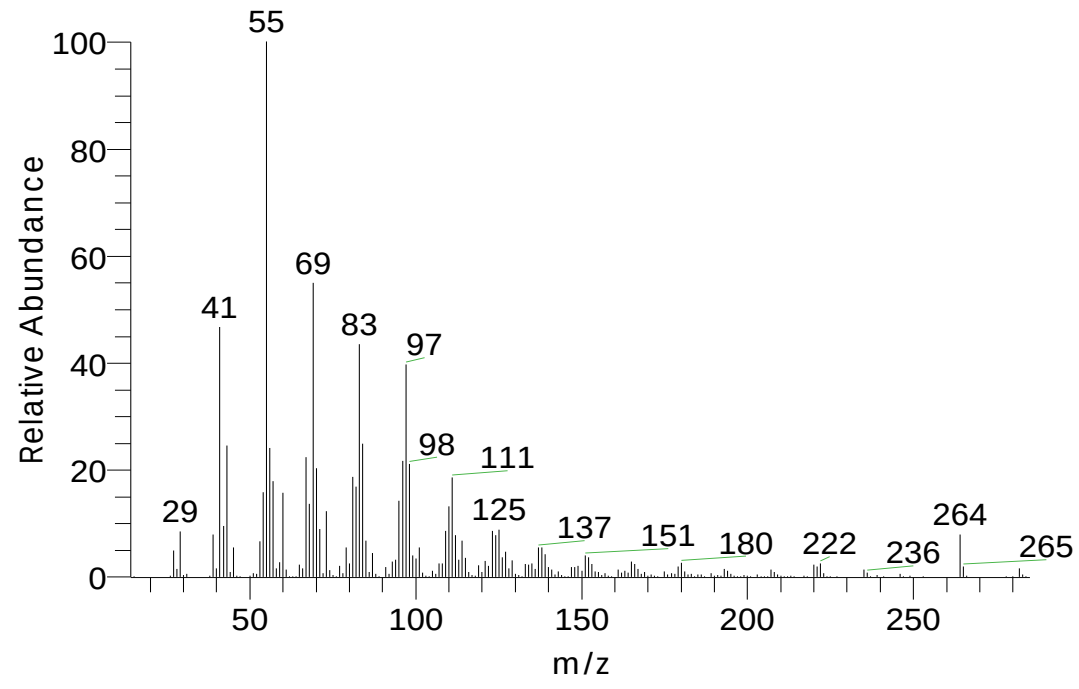

cis-Vaccenic acid
Formula C18H34O2, MW 282, CAS# 506-17-2, Entry# 20090
11-Octadecenoic acid, (Z)-

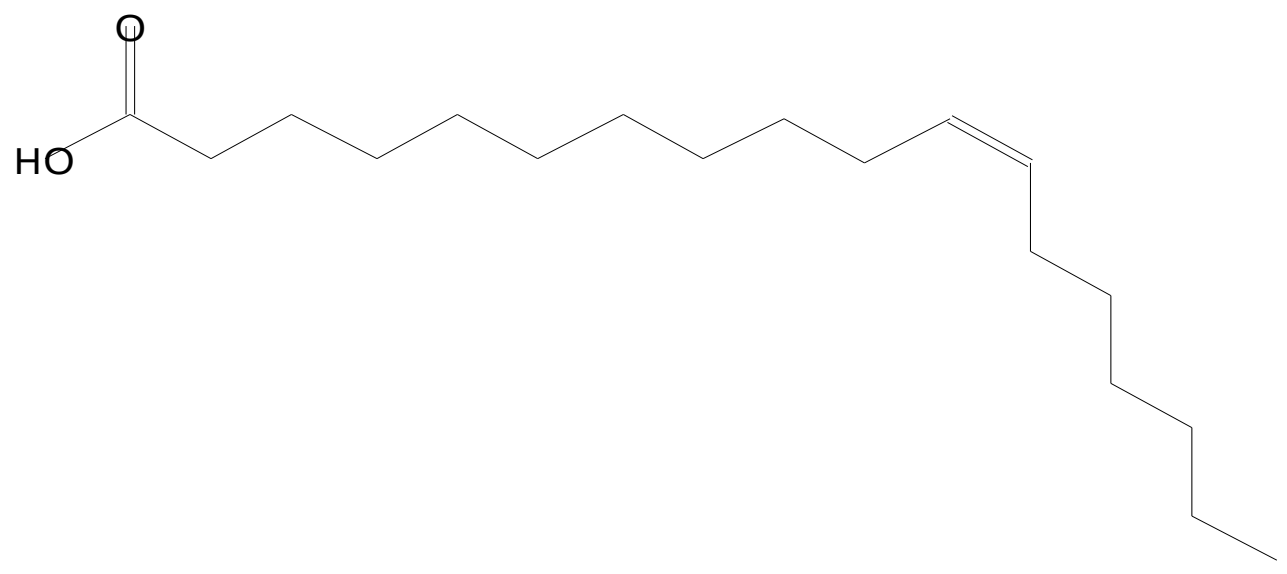

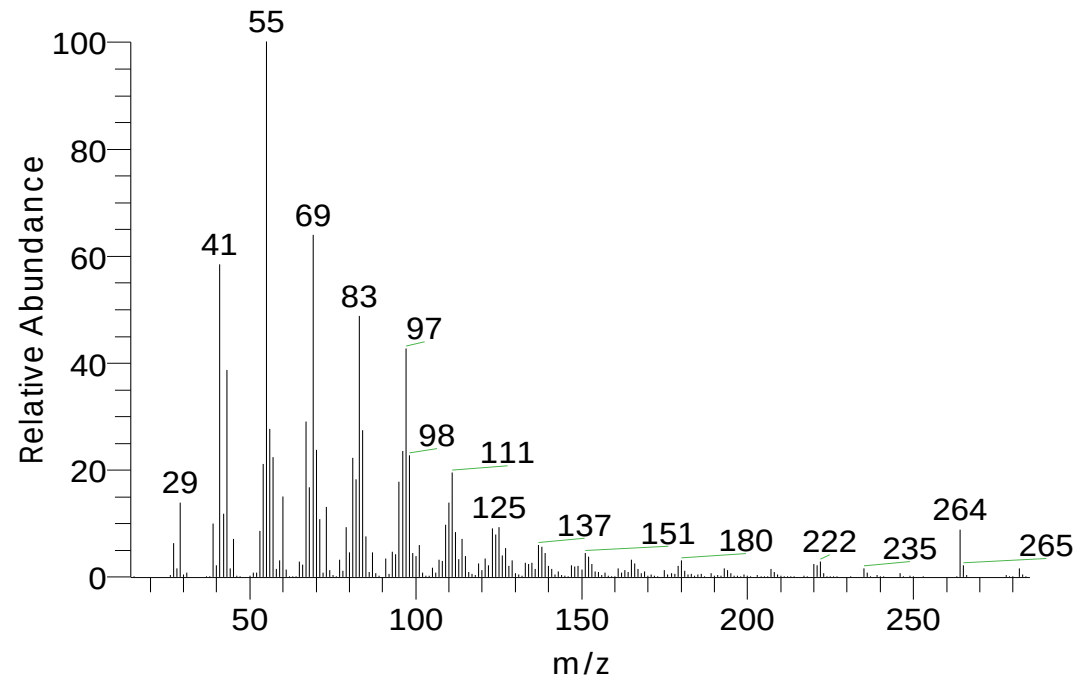

9-Octadecenoic acid, (E)-
Formula C18H34O2, MW 282, CAS# 112-79-8, Entry# 5015
trans-ë(sup 9)-Octadecenoic acid

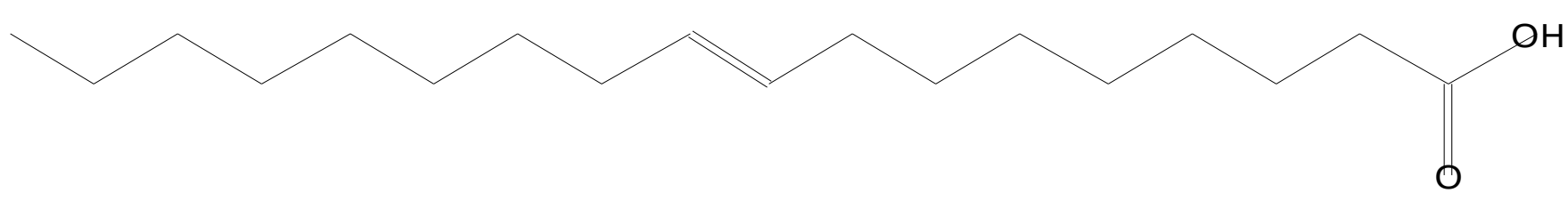

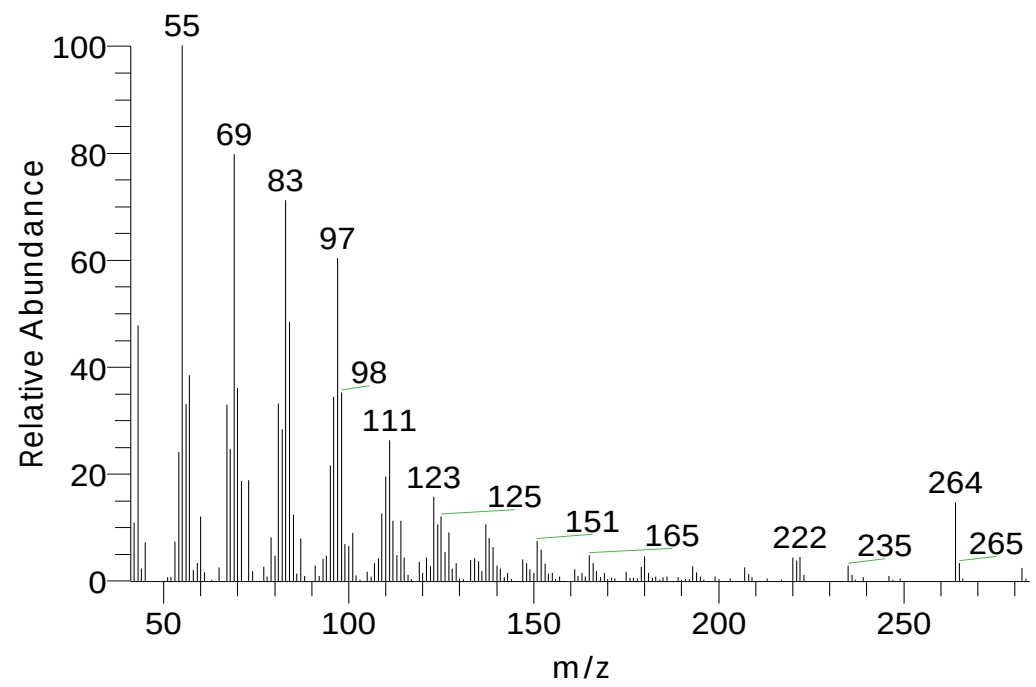

Oleic Acid
Formula C18H34O2, MW 282, CAS# 112-80-1, Entry# 5017
9-Octadecenoic acid (Z)-

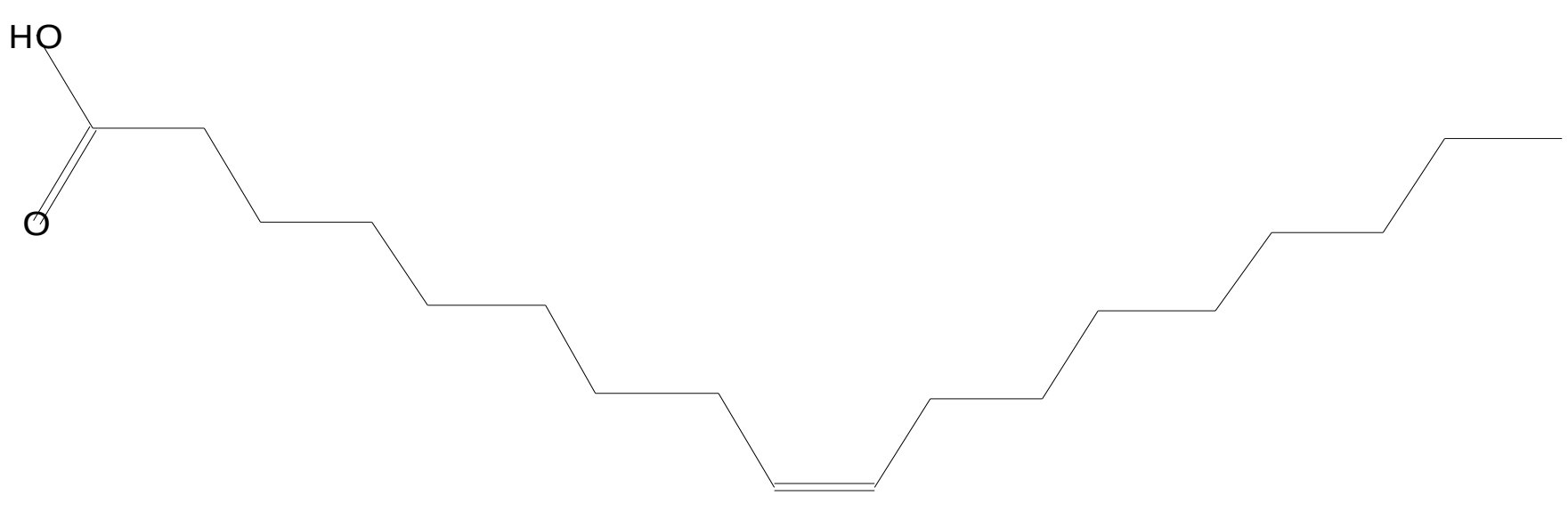

SI 908, RSI 919, replib, Entry# 5017, CAS# 112-80-1, Oleic Acid

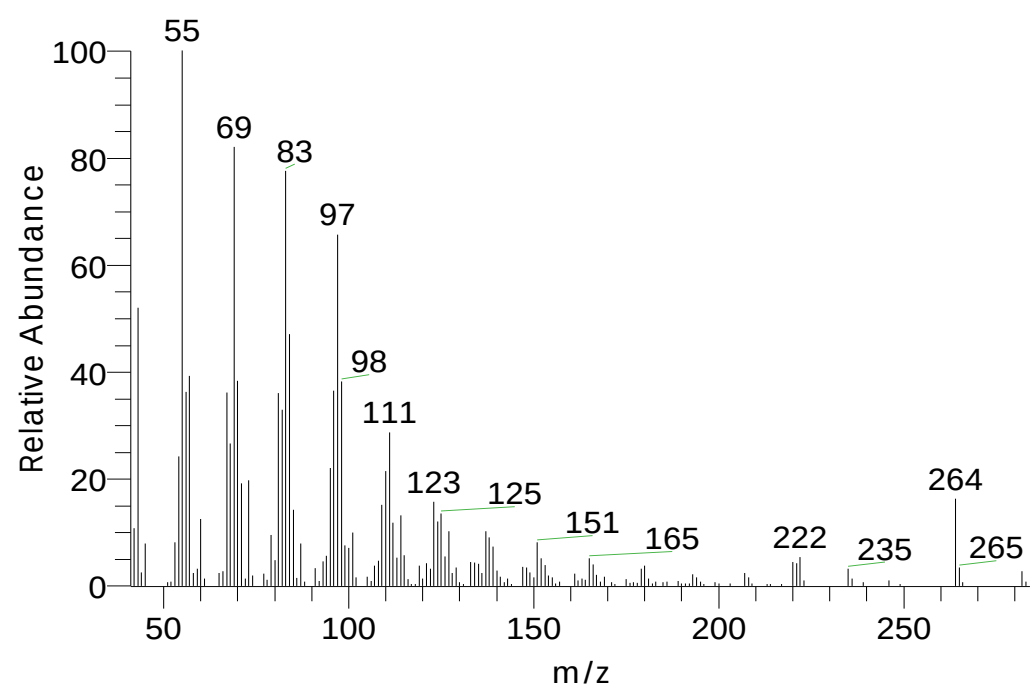

My GC-MS Report

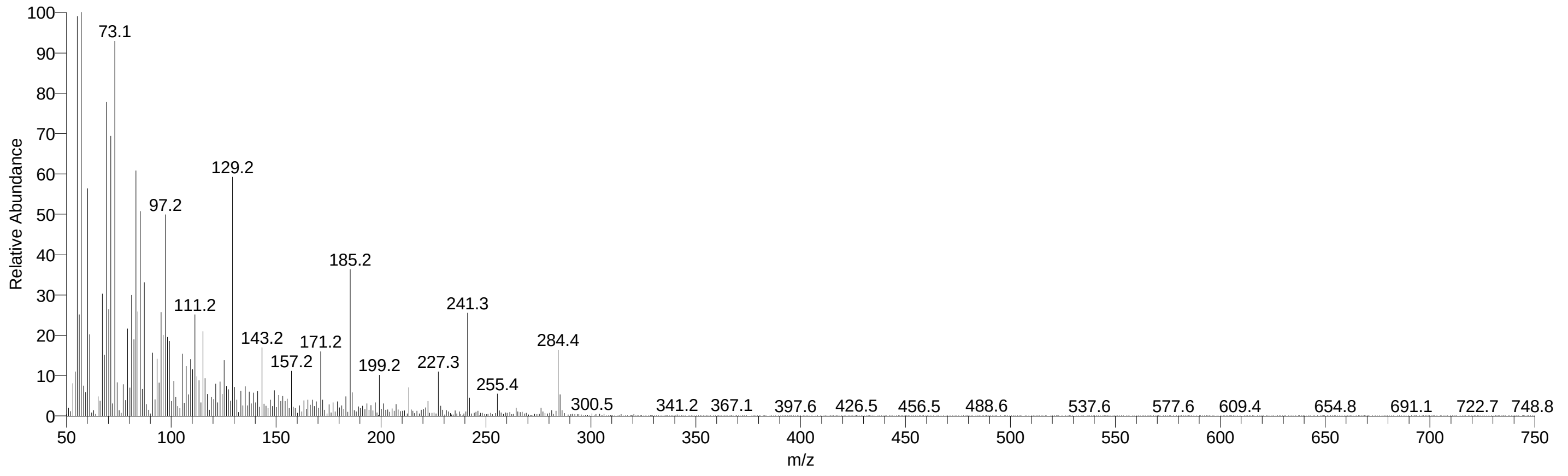

RT	Compound Name	Area %	MF	Molecular Formula	Molecular Weight	Cas #	Library
29.87	OCTADECANOIC ACID	3.53	838	C18H36O2	284	57-11-4	WileyRegistry8e
29.87	Octadecanoic acid	3.53	816	C18H36O2	284	57-11-4	mainlib
29.87	Octadecanoic acid	3.53	842	C18H36O2	284	57-11-4	replib
29.87	Octadecanoic acid	3.53	806	C18H36O2	284	57-11-4	replib
29.87	Octadecanoic acid, 2-hydroxy-1,3-propanediyl ester	3.53	765	C39H76O5	624	504-40-5	replib

Compound Structure | Hit Spectrum

OCTADECANOIC ACID
Formula C18H36O2, MW 284, CAS# 57-11-4, Entry# 174897
STEARATE

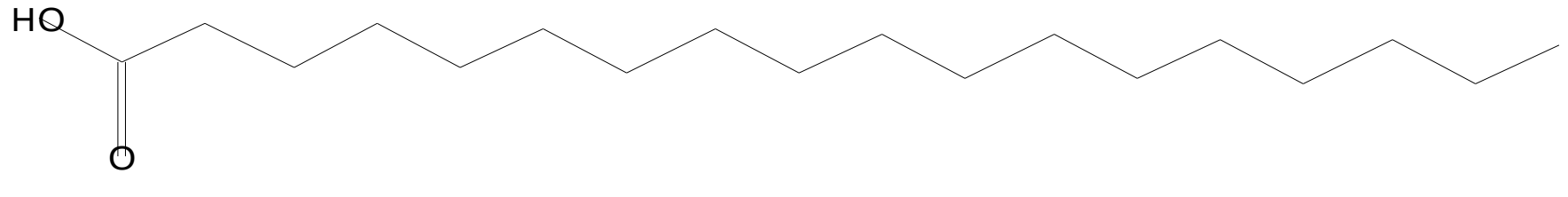

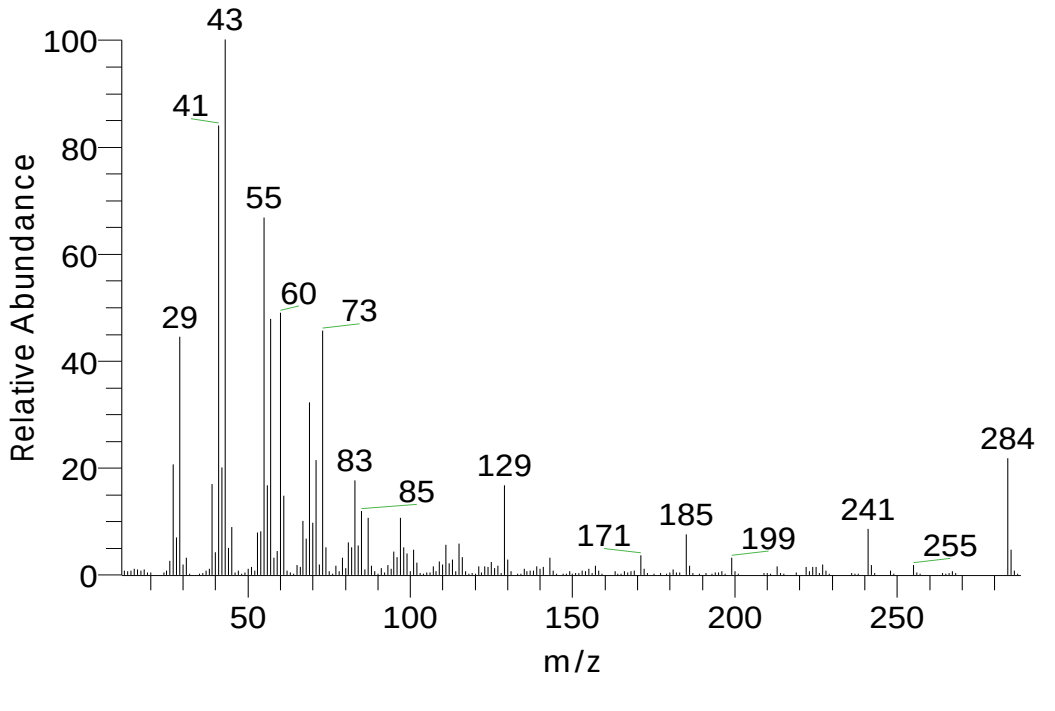

Octadecanoic acid
Formula C18H36O2, MW 284, CAS# 57-11-4, Entry# 9210
Stearic acid

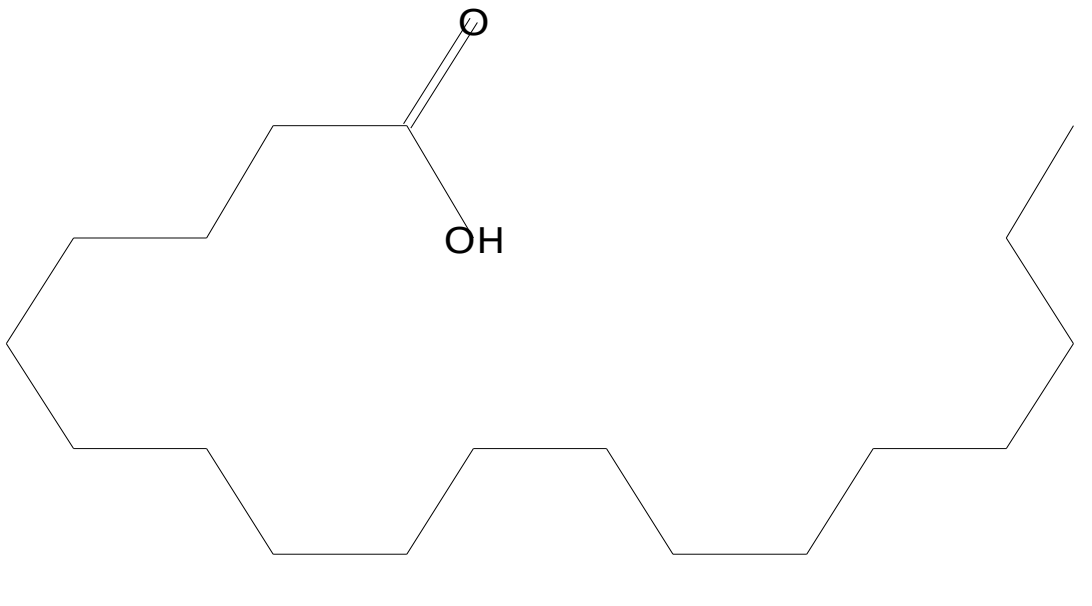

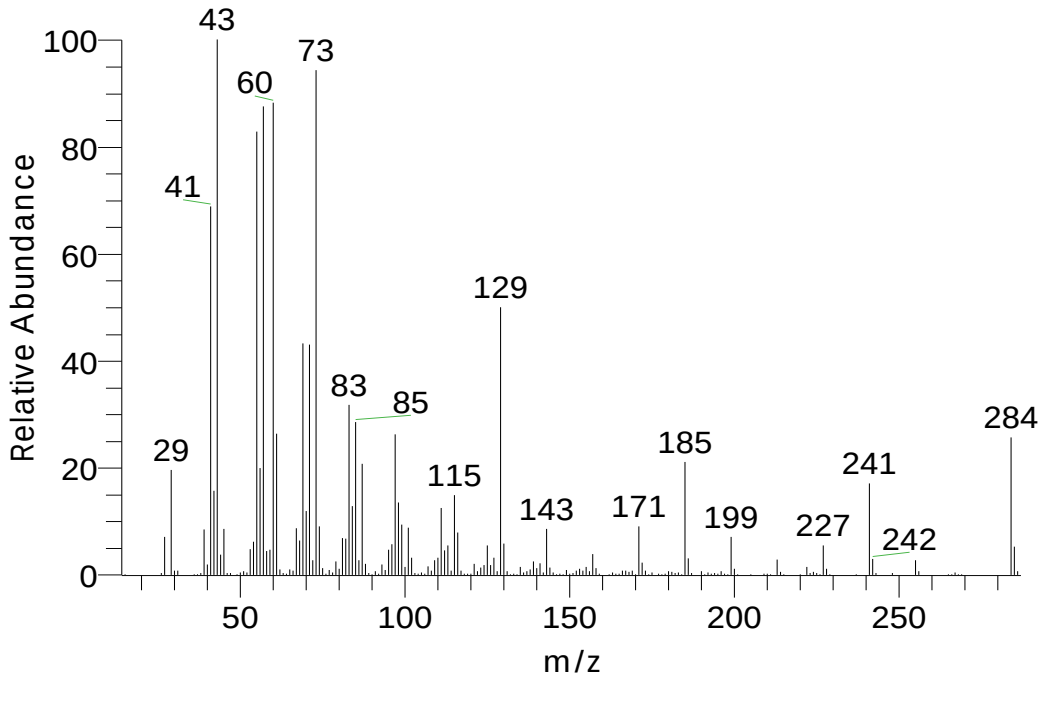

My GC-MS Report

Compound Structure | Hit Spectrum

Octadecanoic acid
Formula C18H36O2, MW 284, CAS# 57-11-4, Entry# 1866
Stearic acid

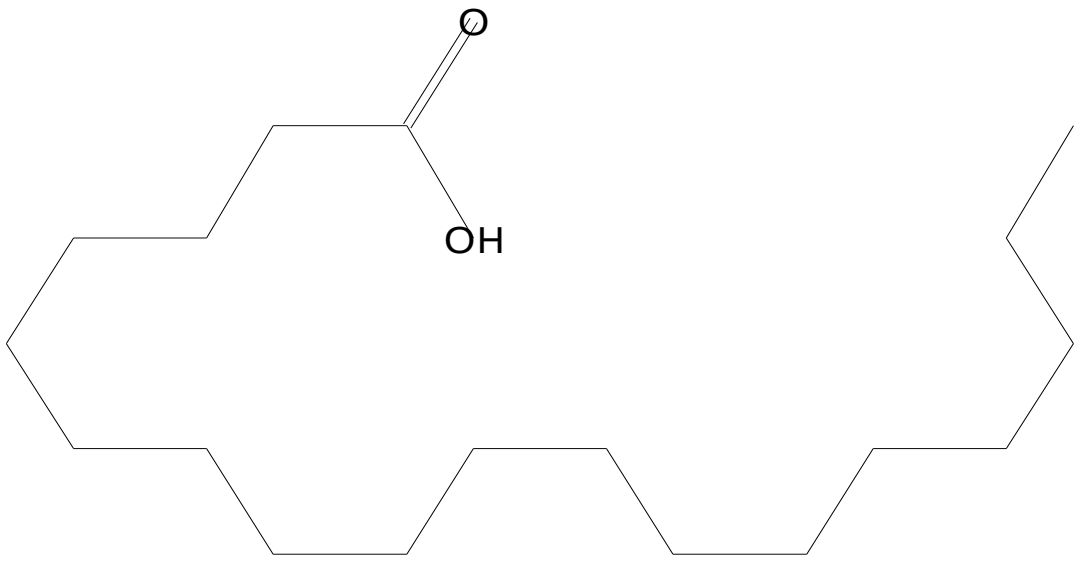

SI 775, RSI 842, replib, Entry# 1866, CAS# 57-11-4, Octadecanoic acid

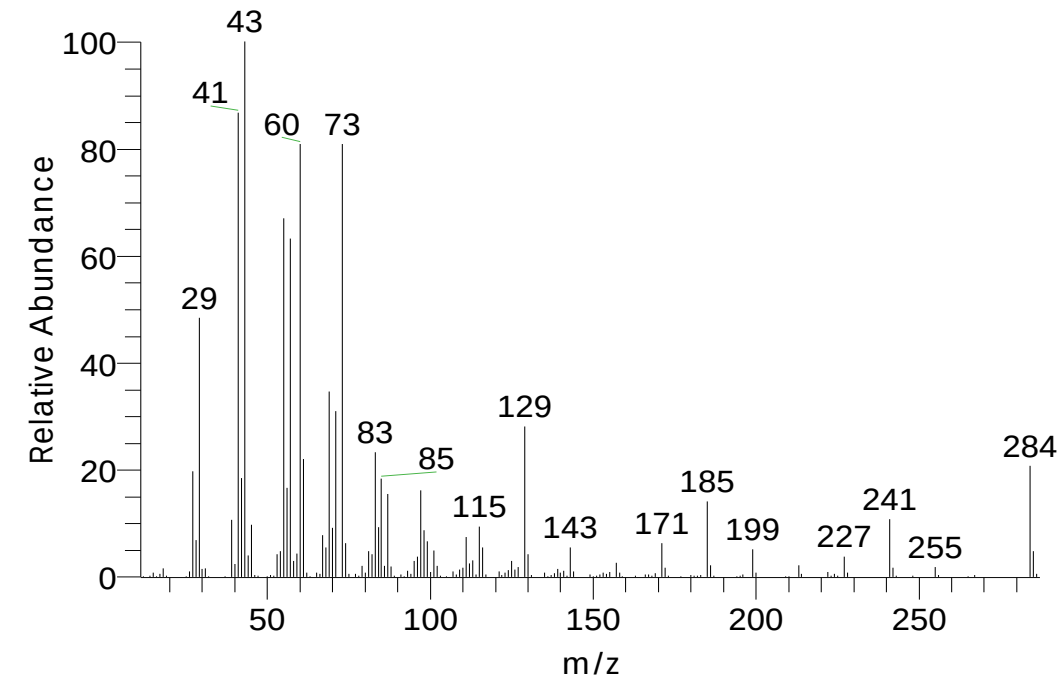

Octadecanoic acid
Formula C18H36O2, MW 284, CAS# 57-11-4, Entry# 9623
Stearic acid

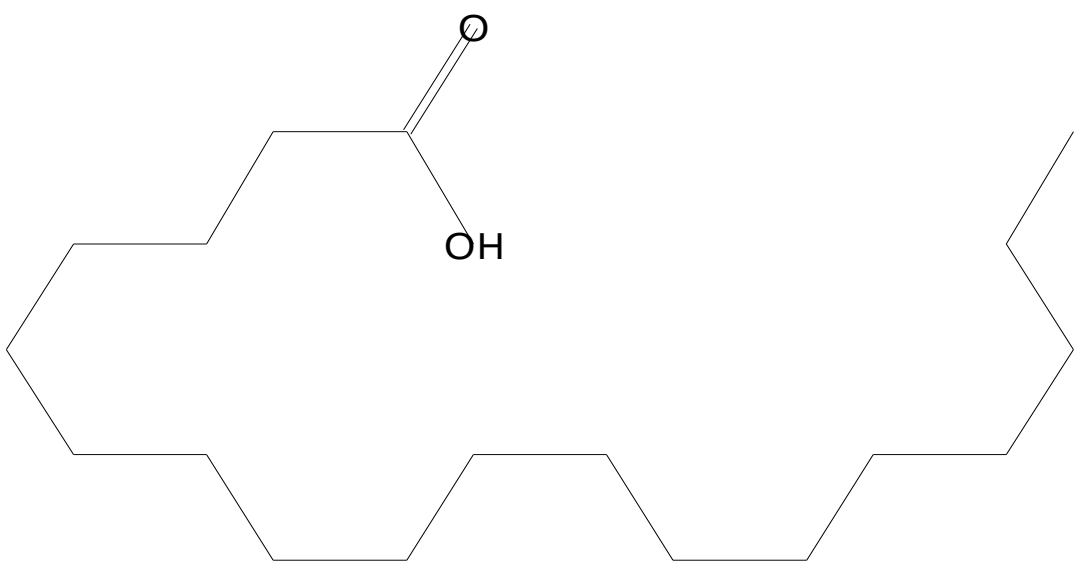

SI 765, RSI 806, replib, Entry# 9623, CAS# 57-11-4, Octadecanoic acid

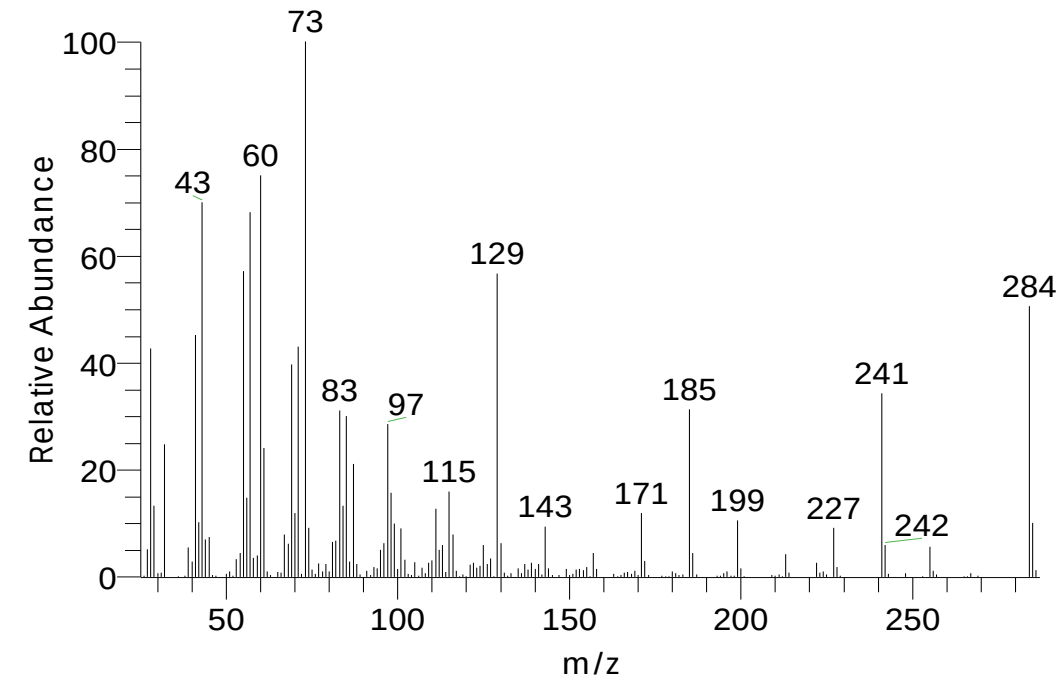

Octadecanoic acid, 2-hydroxy-1,3-propanediyl ester
Formula C39H76O5, MW 624, CAS# 504-40-5, Entry# 2272
Stearin, 1,3-di-

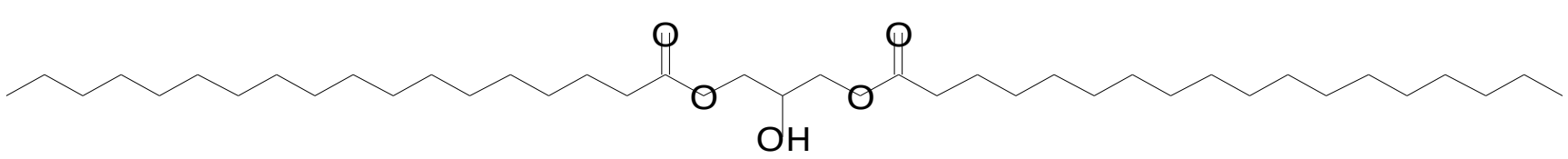

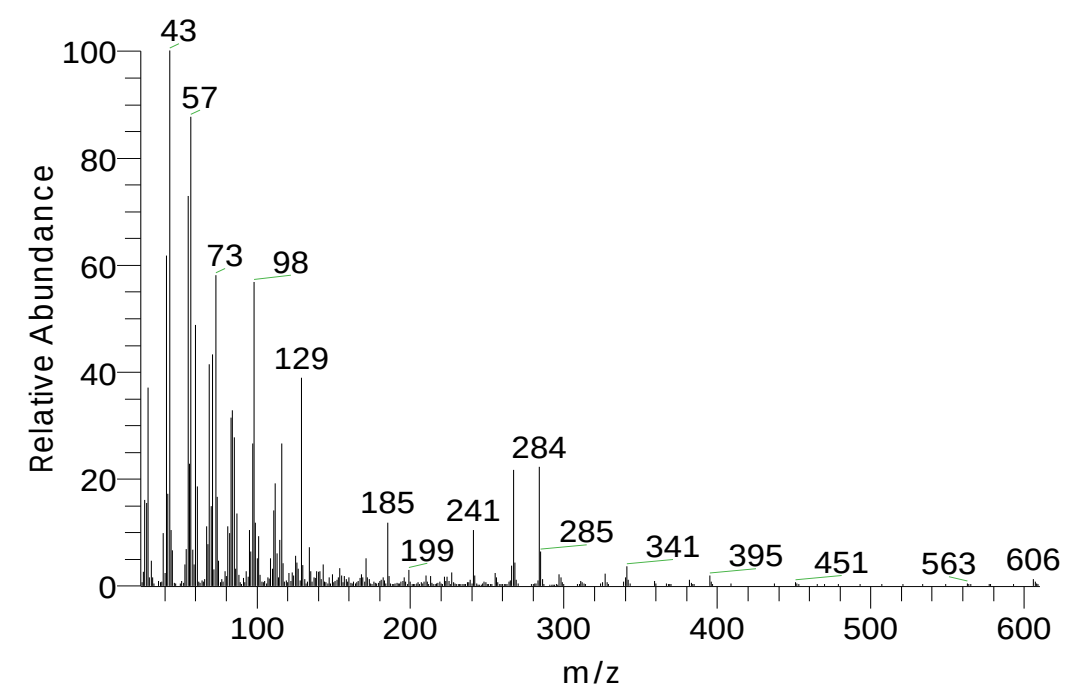

Oil_DrHafsa #7965 RT: 30.71 AV: 1 NL: 6.25E5
T: + c EI Full ms [50.000-750.000]

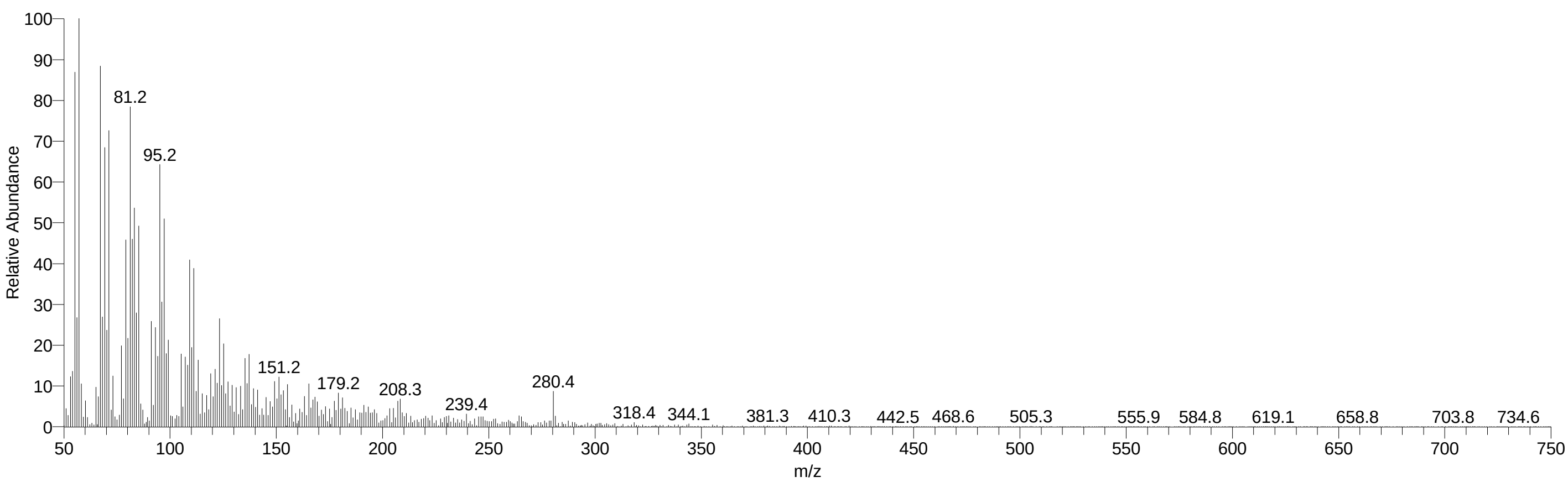

RT	Compound Name	Area %	MF	Molecular Formula	Molecular Weight	Cas #	Library
30.71	E,E,Z-1,3,12-Nonadecatriene-5,14-diol	0.61	802	C19H34O2	294	NA	mainlib
30.71	HAHNFETT	0.61	781	N/A	0	NA	WileyRegistry8e

My GC-MS Report

RT	Compound Name	Area %	MF	Molecular Formula	Molecular Weight	Cas #	Library
30.71	HAHNFETT	0.61	781	N/A	0	NA	WileyRegistry8e
30.71	12-Methyl-E,E-2,13-octadecadien-1-ol	0.61	848	C19H36O	280	NA	mainlib
30.71	14-á-H-PREGNA	0.61	815	C21H36	288	NA	WileyRegistry8e

Compound Structure | Hit Spectrum

E,E,Z-1,3,12-Nonadecatriene-5,14-diol
Formula C19H34O2, MW 294, CAS# NA, Entry# 21026
(3E,12Z)-1,3,12-Nonadecatriene-5,14-diol #

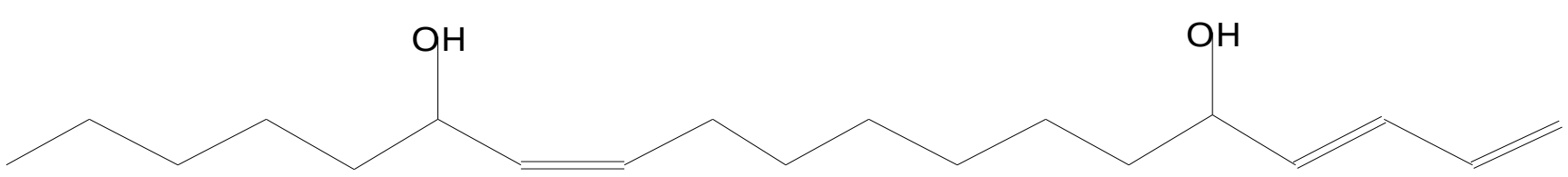

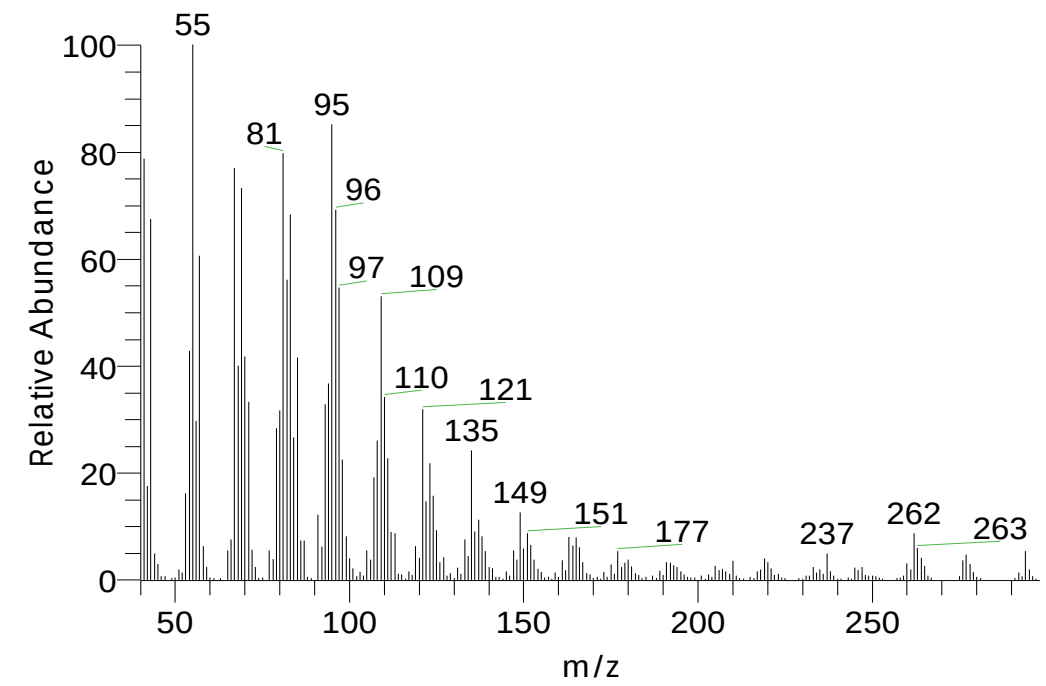

HAHNFETT
Formula , MW 0, CAS# NA, Entry# 305496

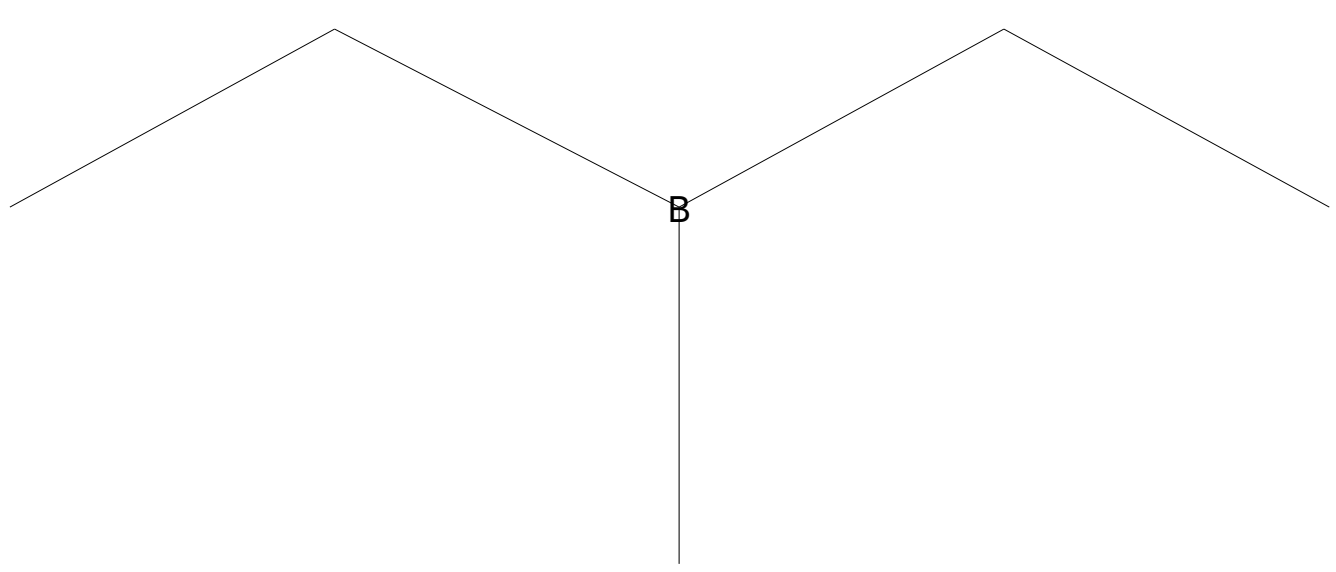

SI 774, RSI 781, WileyRegistry8e, Entry# 305496, CAS# NA, HAHNFETT

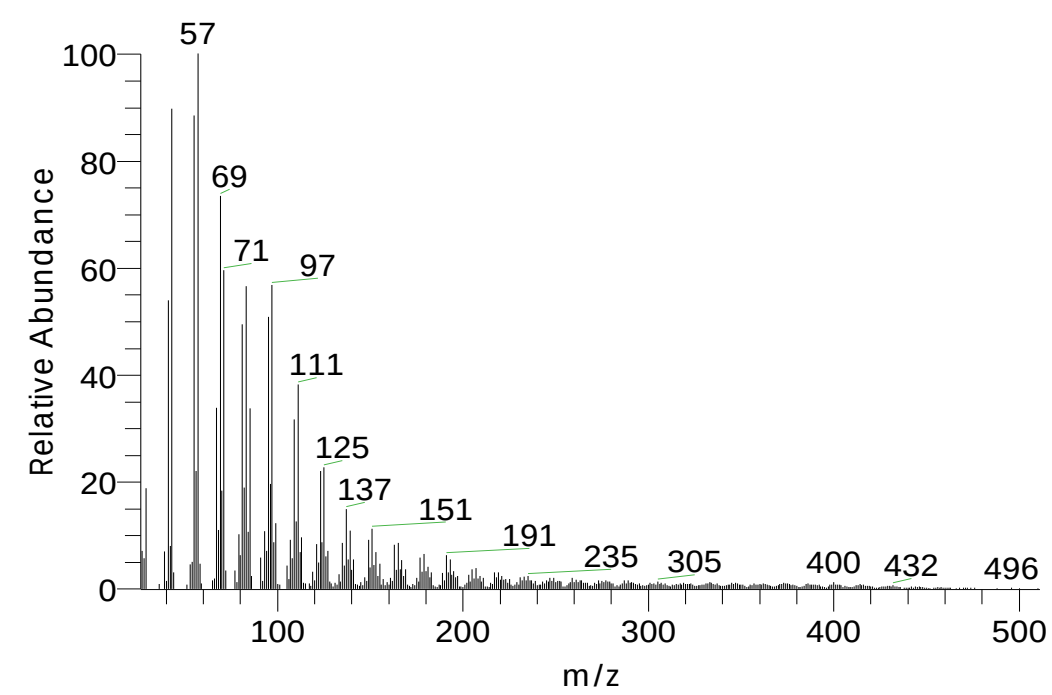

HAHNFETT
Formula , MW 0, CAS# NA, Entry# 391160

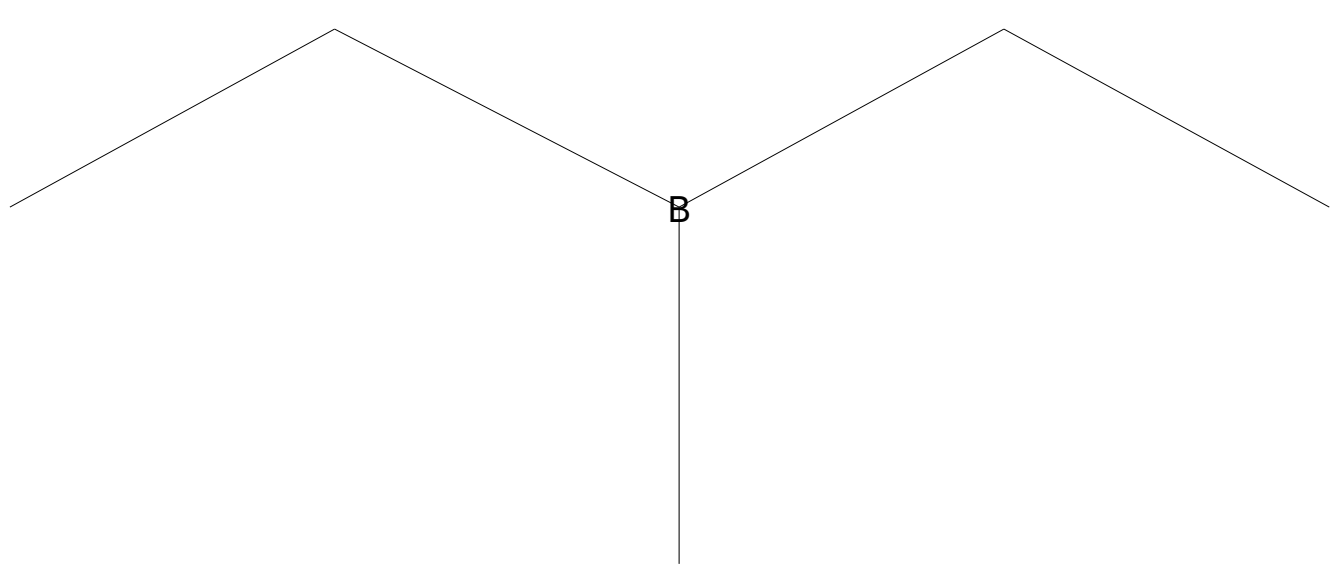

SI 774, RSI 781, WileyRegistry8e, Entry# 391160, CAS# NA, HAHNFETT

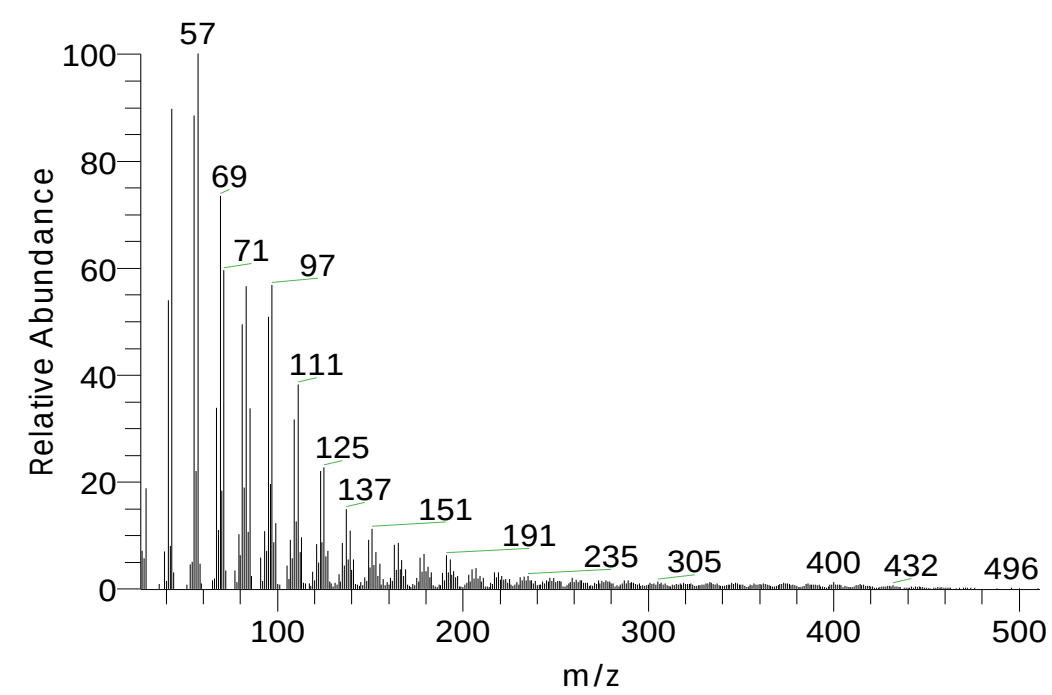

12-Methyl-E,E-2,13-octadecadien-1-ol
Formula C19H36O, MW 280, CAS# NA, Entry# 19016
(2E,15Z)-14-Methyl-2,15-octadecadien-1-ol #

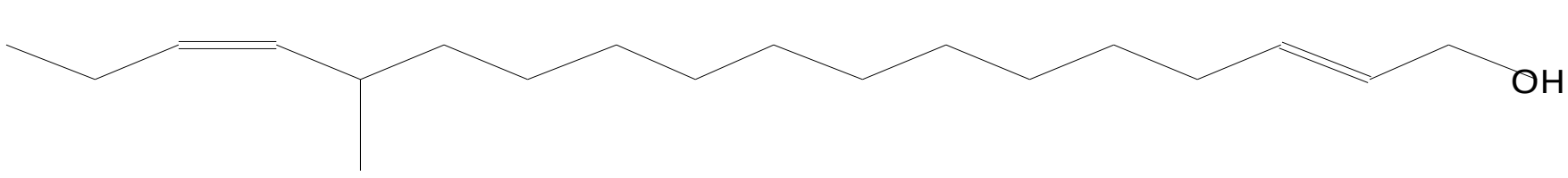

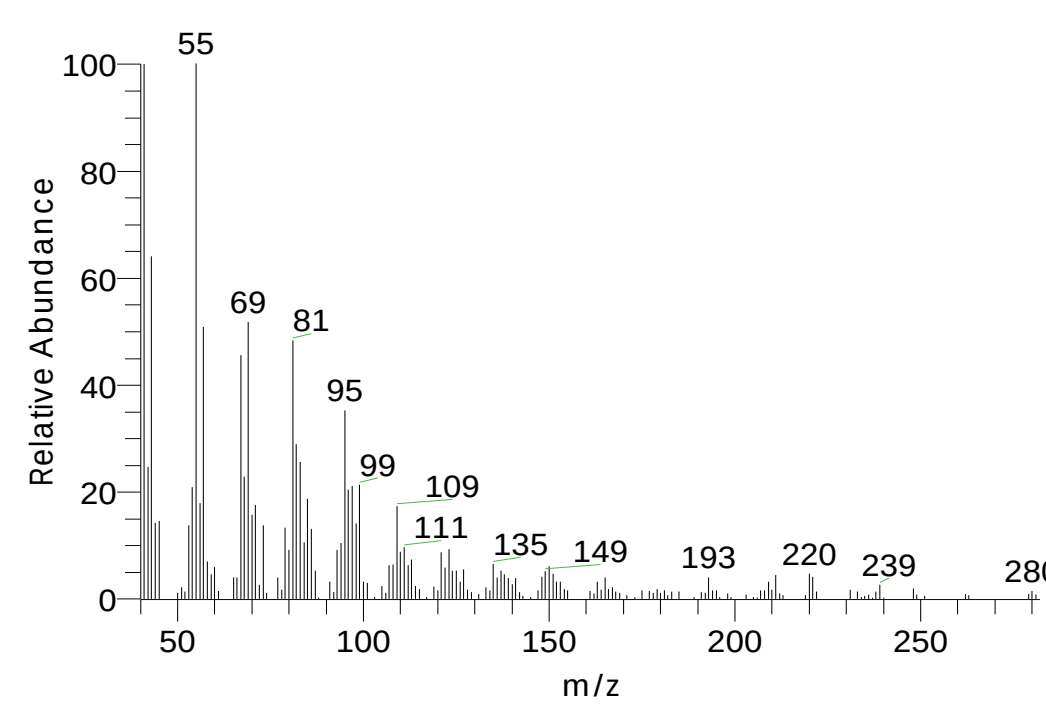

My GC-MS Report

Compound Structure | Hit Spectrum

14-á-H-PREGNA
Formula C21H36, MW 288, CAS# NA, Entry# 178939
14-á-PREGNA

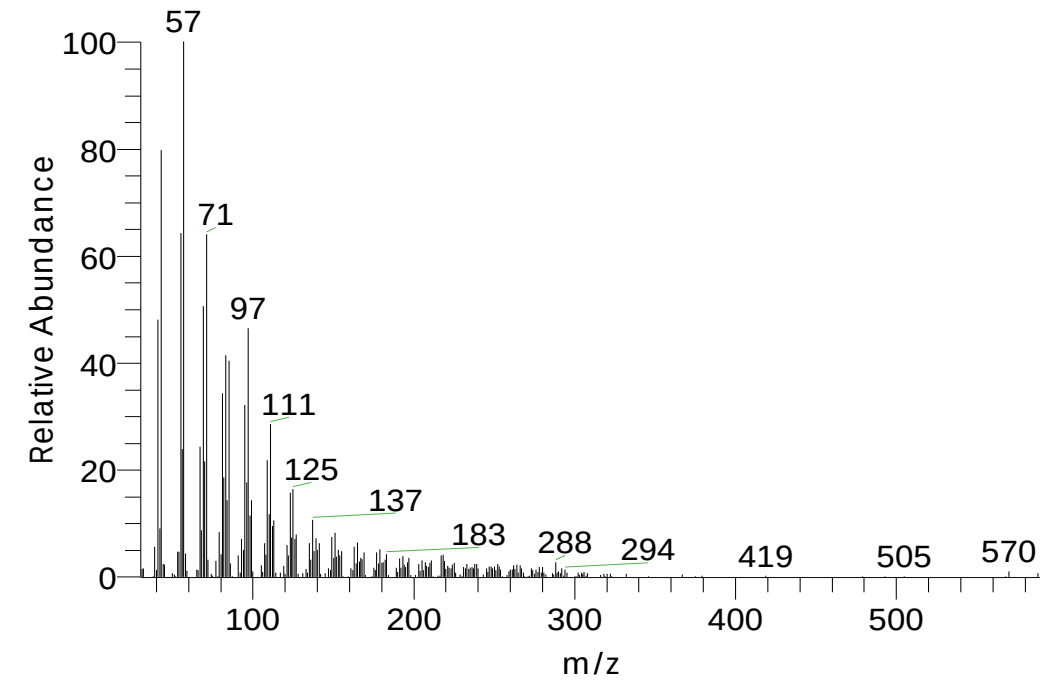

Oil_DrHafsa #8069 RT: 31.06 AV: 1 NL: 6.77E5
T: + c EI Full ms [50.000-750.000]

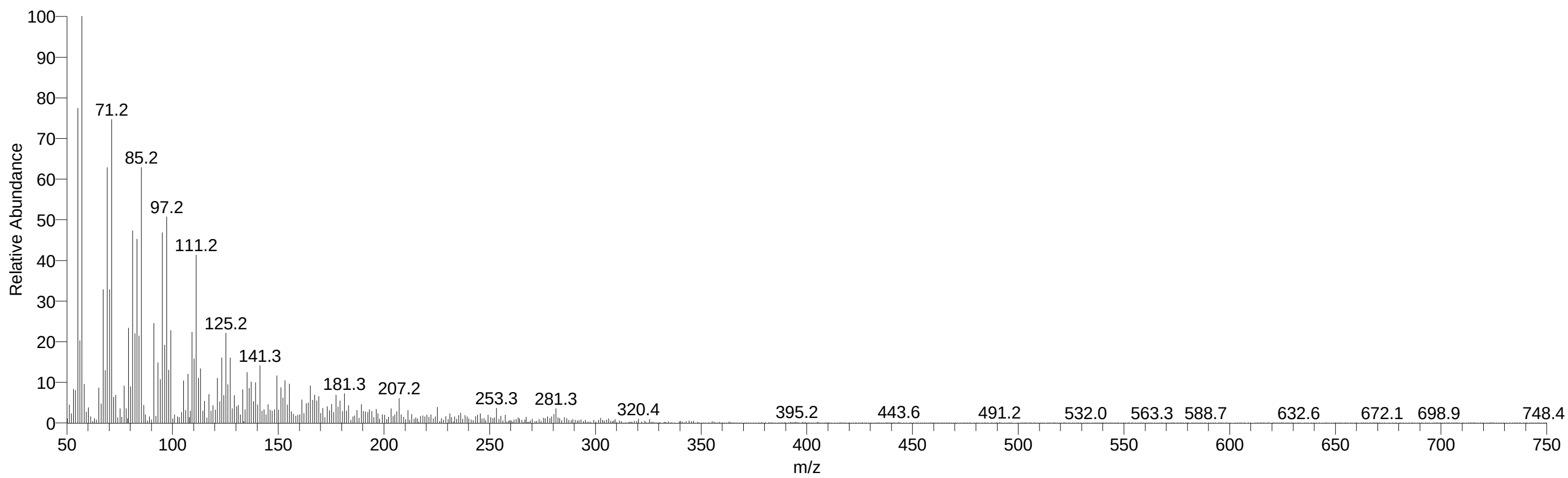

RT	Compound Name	Area %	MF	Molecular Formula	Molecular Weight	Cas #	Library
31.06	ISOCHIAPIN B	0.14	820	C19H22O6	346	NA	WileyRegistry8e
31.06	ISOCHIAPIN B %2<	0.14	820	C19H26O6	350	NA	WileyRegistry8e
31.06	HAHNFETT	0.14	786	N/A	0	NA	WileyRegistry8e
31.06	HAHNFETT	0.14	786	N/A	0	NA	WileyRegistry8e
31.06	DOTRIACONTANE	0.14	808	C32H66	450	544-85-4	WileyRegistry8e

Compound Structure | Hit Spectrum

ISOCHIAPIN B
Formula C19H22O6, MW 346, CAS# NA, Entry# 225807

SI 817, RSI 820, WileyRegistry8e, Entry# 225807, CAS# NA, ISOCHIAPIN B

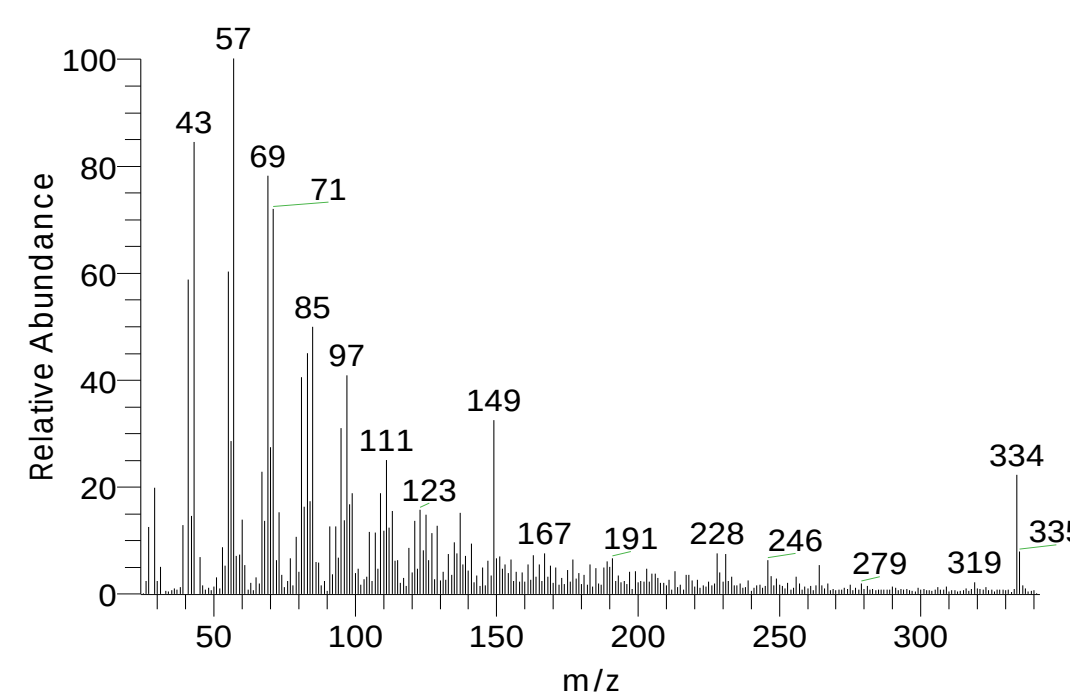

My GC-MS Report

Compound Structure | Hit Spectrum

ISOCHIAPIN B %2<
Formula C19H26O6, MW 350, CAS# NA, Entry# 228500

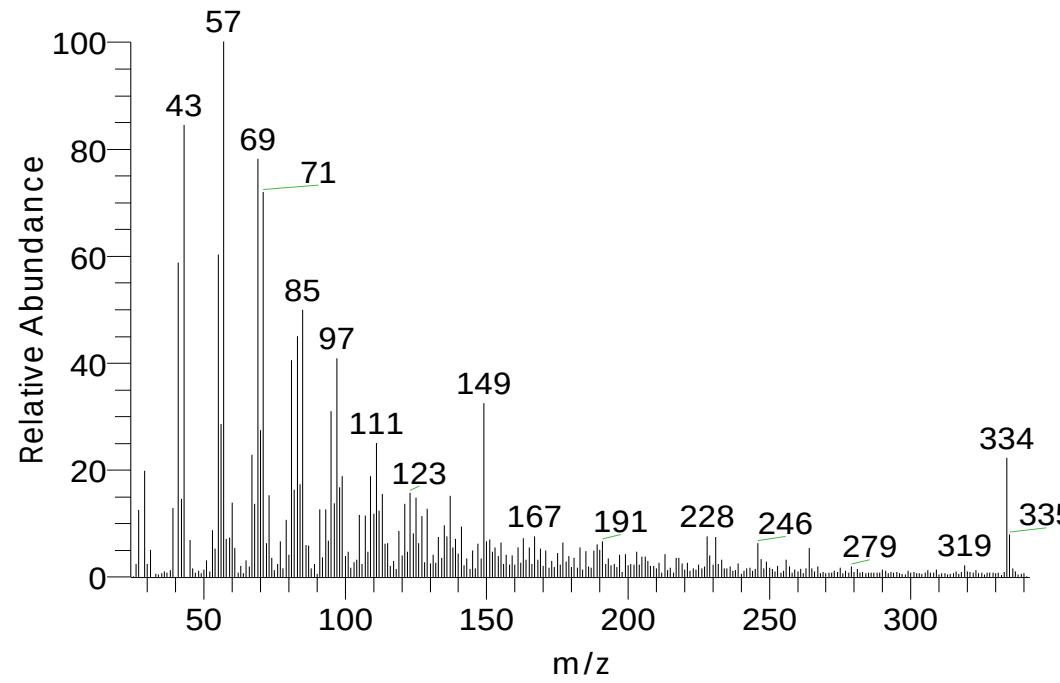

HAHNFETT
Formula , MW 0, CAS# NA, Entry# 305496

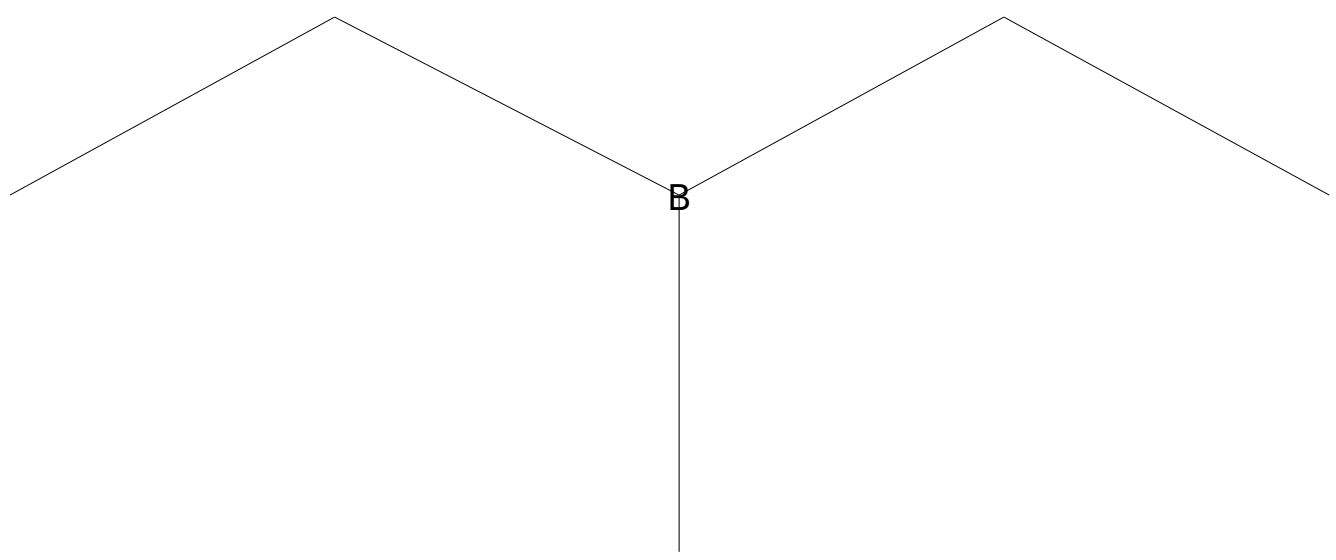

SI 780, RSI 786, WileyRegistry8e, Entry# 305496, CAS# NA, HAHNFETT

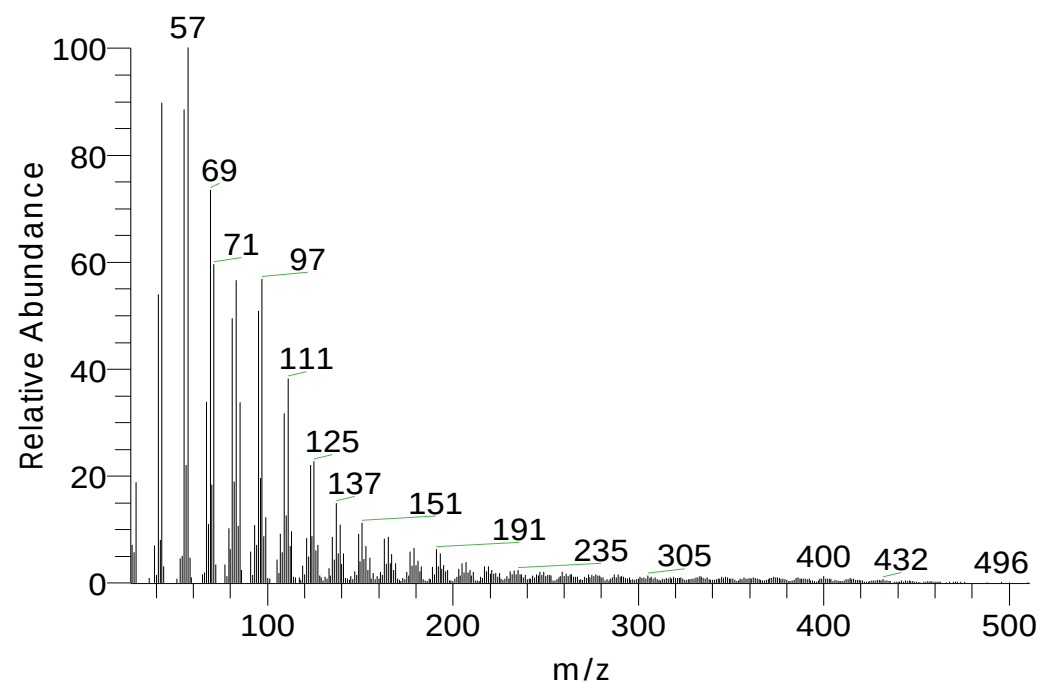

HAHNFETT
Formula , MW 0, CAS# NA, Entry# 391160

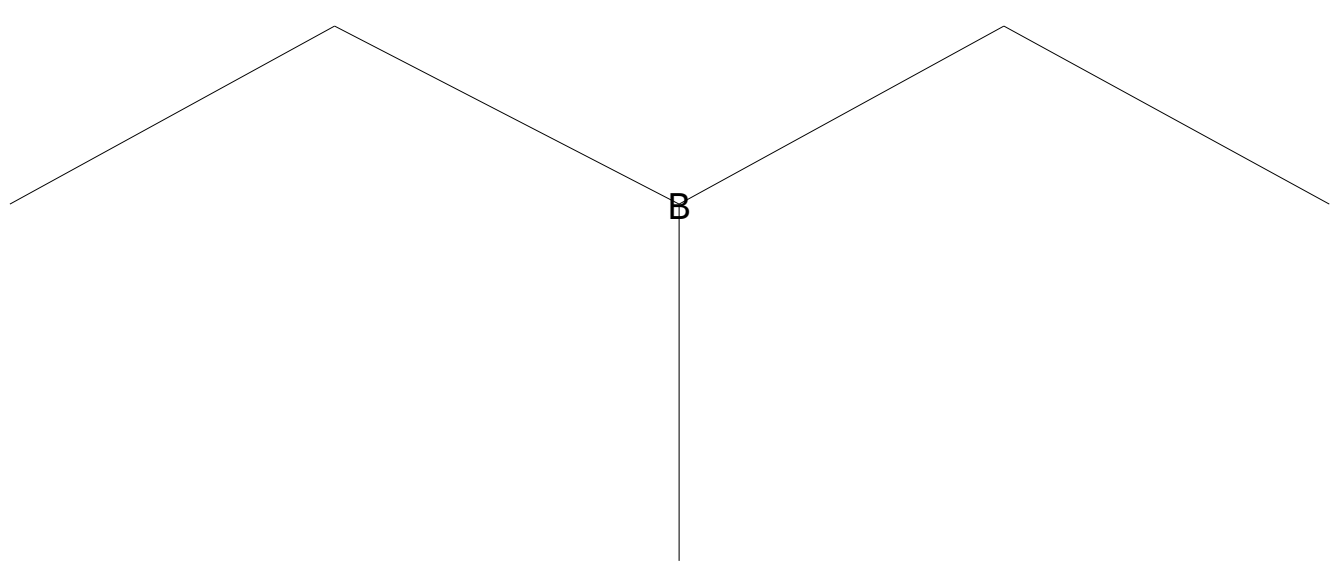

SI 780, RSI 786, WileyRegistry8e, Entry# 391160, CAS# NA, HAHNFETT

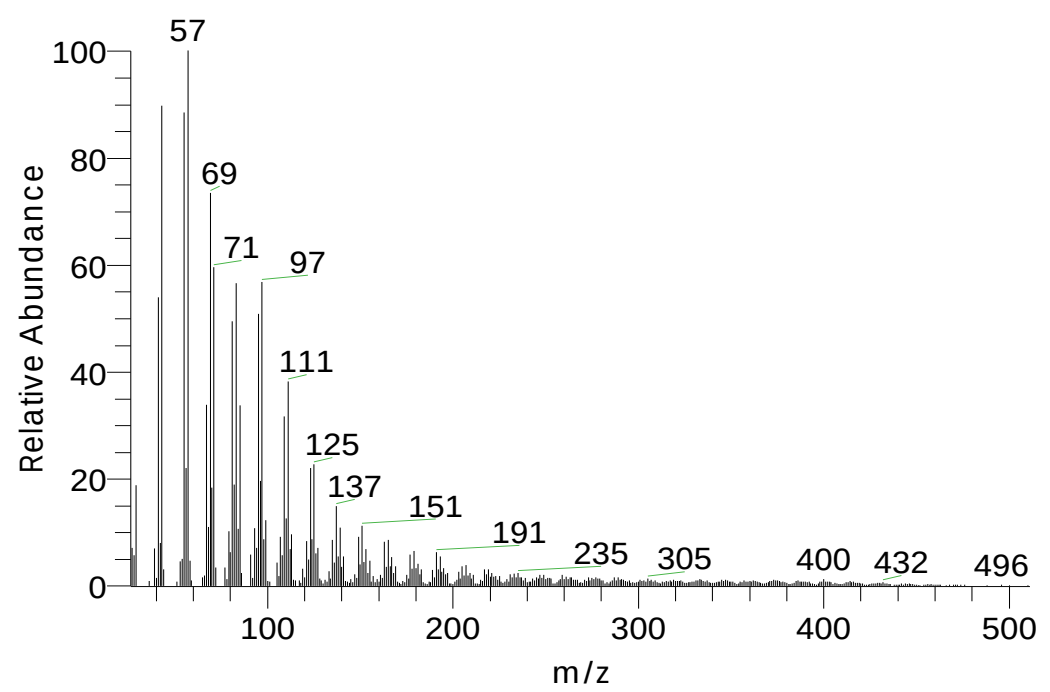

DOTRIACONTANE
Formula C32H66, MW 450, CAS# 544-85-4, Entry# 274478
AI3-52367

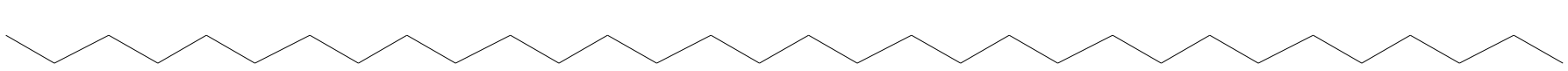

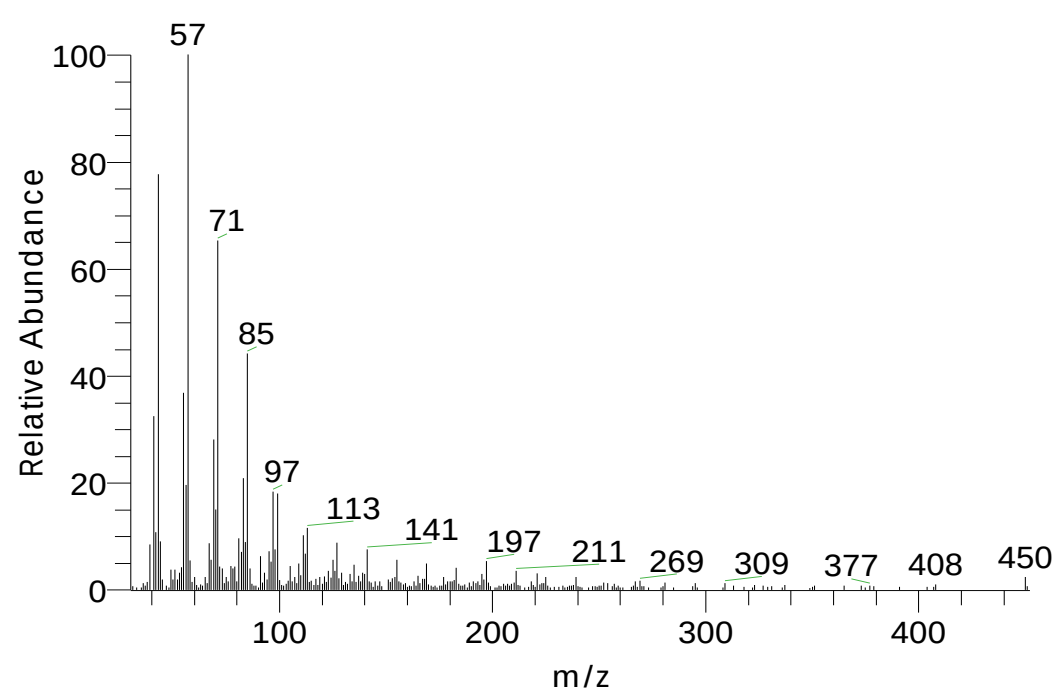

My GC-MS Report

Oil_DrHafsa #8214 RT: 31.54 AV: 1 NL: 6.85E5
T: + c EI Full ms [50.000-750.000]

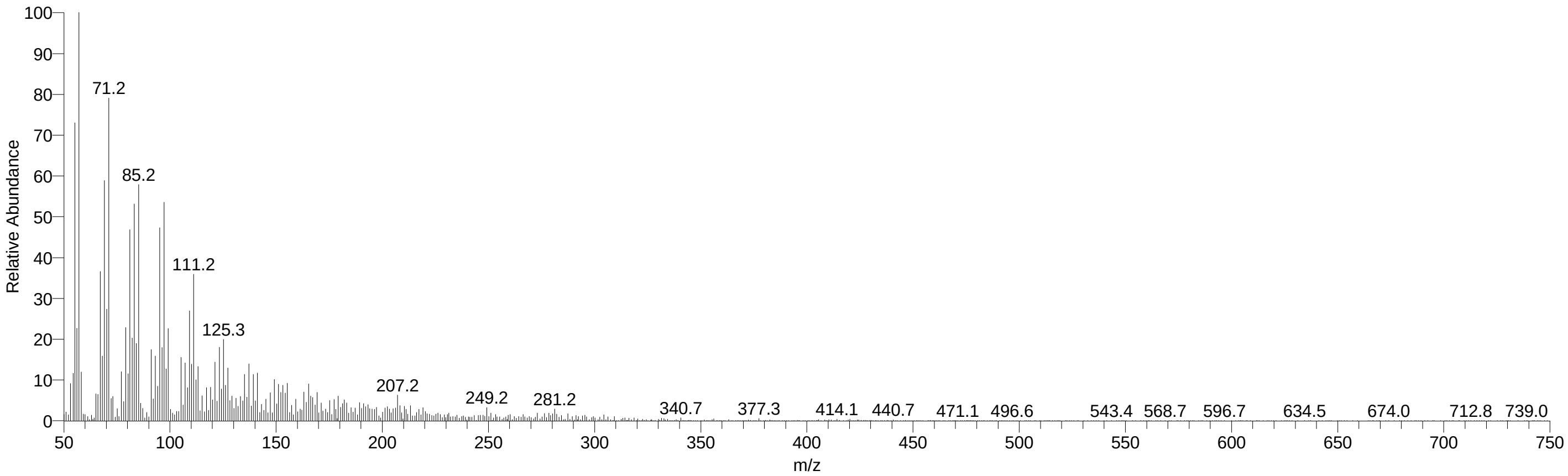

RT	Compound Name	Area %	MF	Molecular Formula	Molecular Weight	Cas #	Library
31.54	ISOCHIAPIN B	0.23	807	C19H22O6	346	NA	WileyRegistry8e
31.54	ISOCHIAPIN B %2<	0.23	807	C19H26O6	350	NA	WileyRegistry8e
31.54	DOTRIACONTANE	0.23	819	C32H66	450	544-85-4	WileyRegistry8e
31.54	HAHNFETT	0.23	776	N/A	0	NA	WileyRegistry8e
31.54	HAHNFETT	0.23	776	N/A	0	NA	WileyRegistry8e

Compound Structure	Hit Spectrum

ISOCHIAPIN B
Formula C19H22O6, MW 346, CAS# NA, Entry# 225807

SI 804, RSI 807, WileyRegistry8e, Entry# 225807, CAS# NA, ISOCHIAPIN B

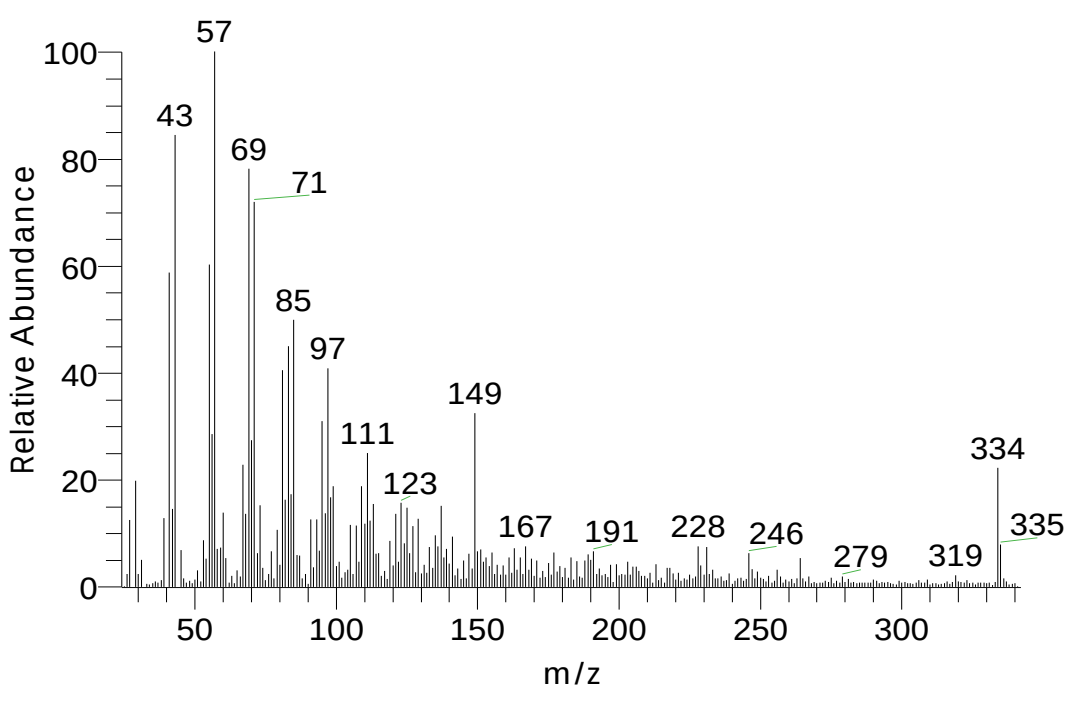

ISOCHIAPIN B %2<
Formula C19H26O6, MW 350, CAS# NA, Entry# 228500

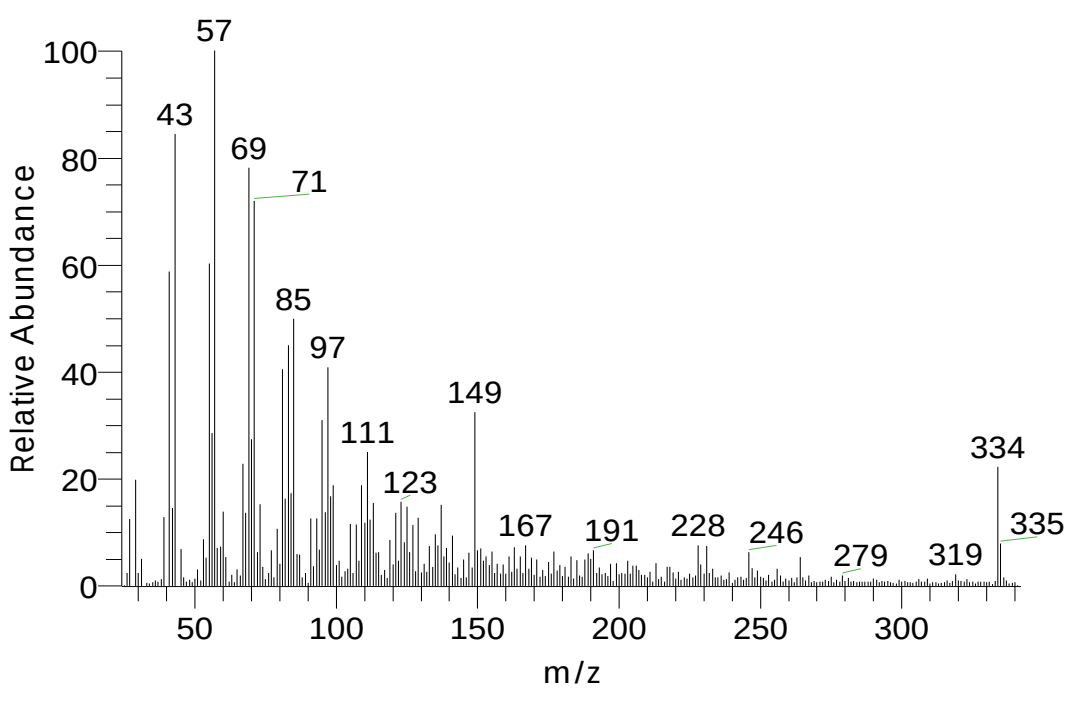

My GC-MS Report

Compound Structure | Hit Spectrum

DOTRIACONTANE
Formula C32H66, MW 450, CAS# 544-85-4, Entry# 274478
AI3-52367

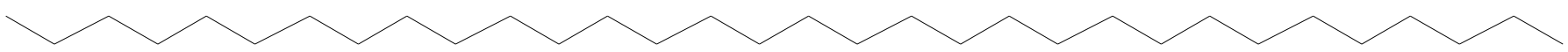

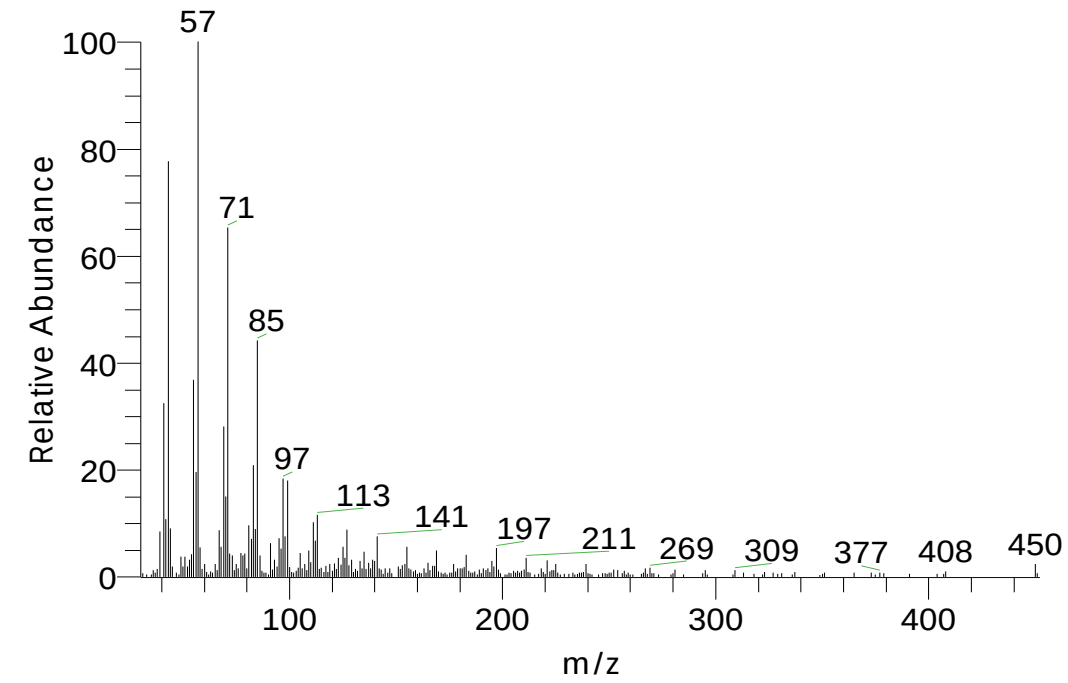

SI 770, RSI 776, WileyRegistry8e, Entry# 305496, CAS# NA, HAHNFETT

HAHNFETT
Formula , MW 0, CAS# NA, Entry# 305496

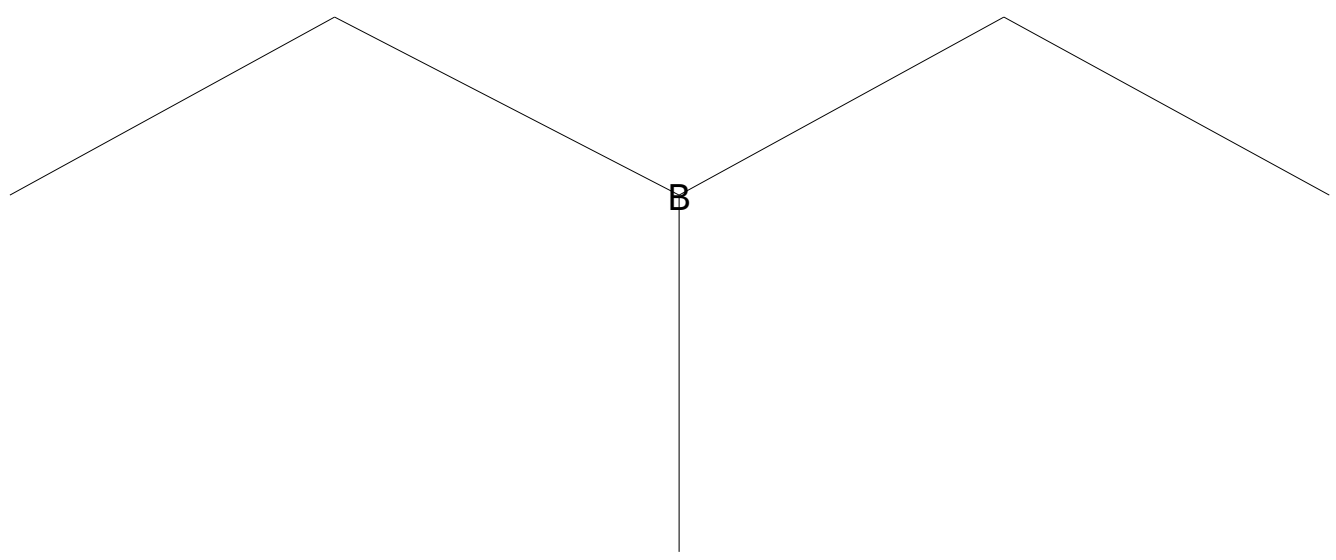

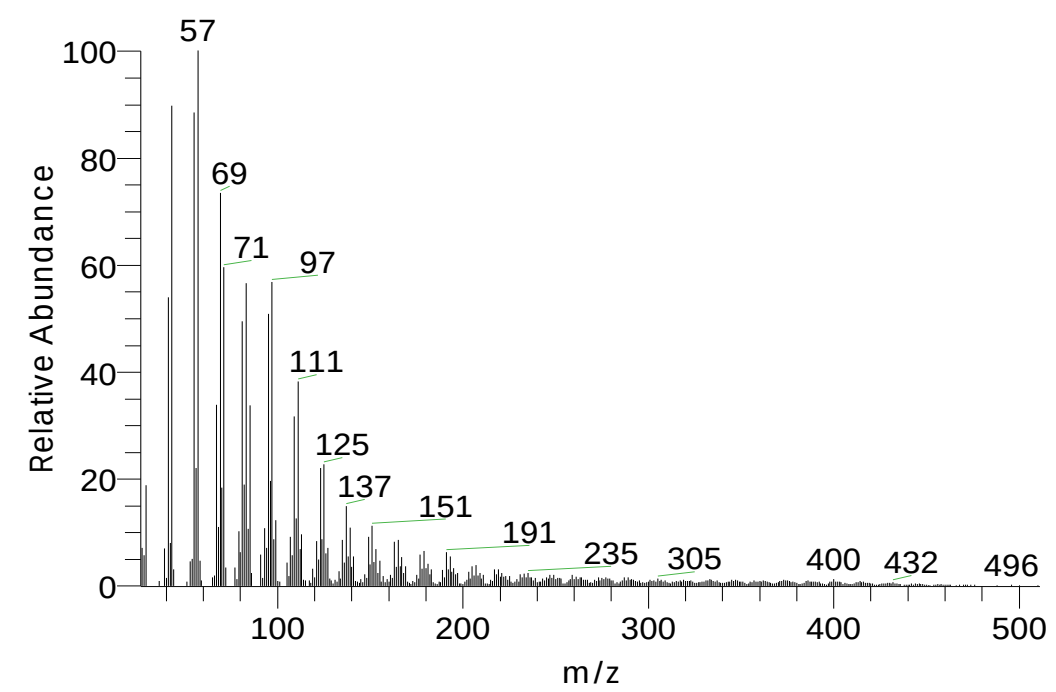

SI 770, RSI 776, WileyRegistry8e, Entry# 391160, CAS# NA, HAHNFETT

HAHNFETT
Formula , MW 0, CAS# NA, Entry# 391160

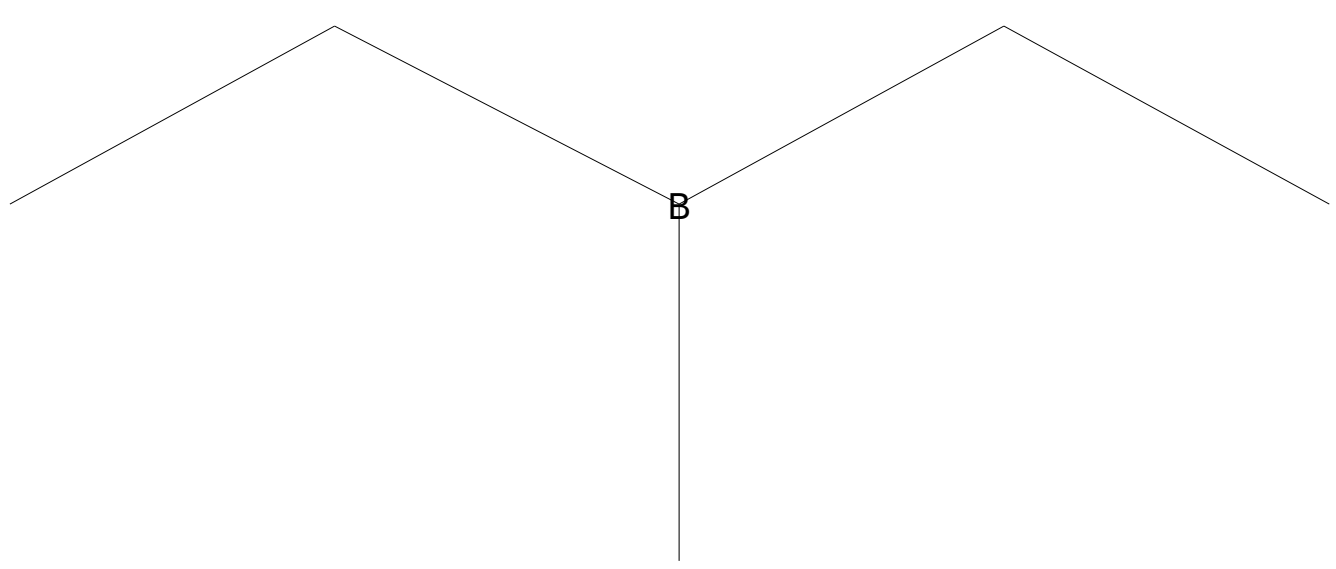

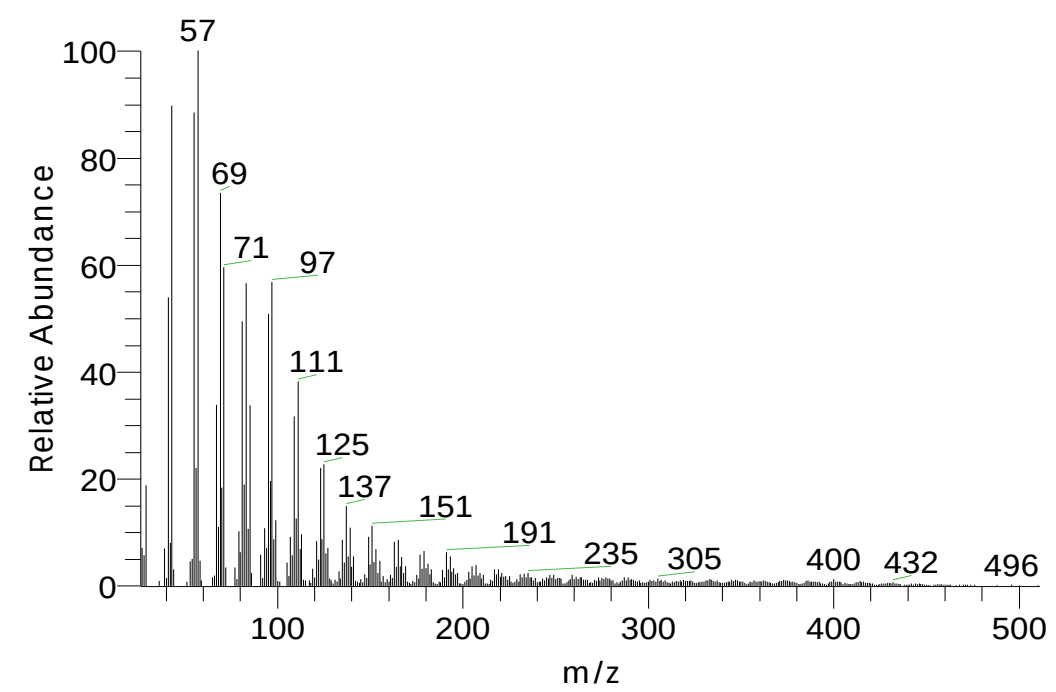

Oil_DrHafsa #8252 RT: 31.67 AV: 1 NL: 7.69E5
T: + c EI Full ms [50.000-750.000]

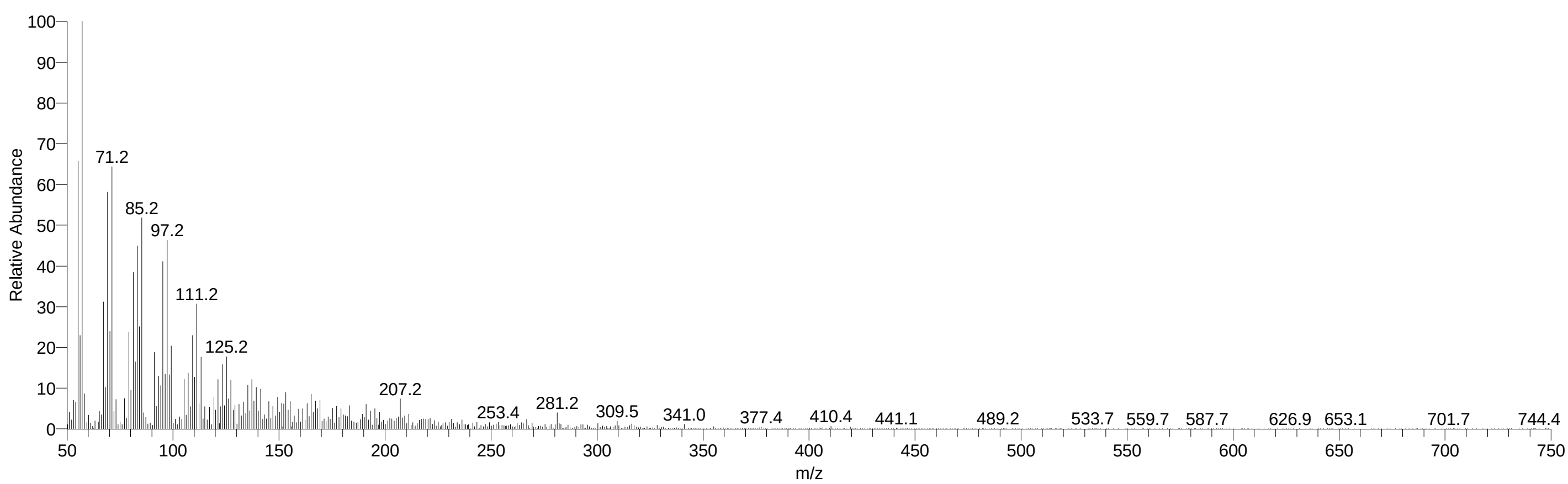

RT	Compound Name	Area %	MF	Molecular Formula	Molecular Weight	Cas #	Library
31.67	ISOCHIAPIN B	0.25	812	C19H22O6	346	NA	WileyRegistry8e
31.67	ISOCHIAPIN B %2<	0.25	812	C19H26O6	350	NA	WileyRegistry8e

My GC-MS Report

RT	Compound Name	Area %	MF	Molecular Formula	Molecular Weight	Cas #	Library
31.67	DOTRIACONTANE	0.25	832	C32H66	450	544-85-4	WileyRegistry8e
31.67	HAHNFETT	0.25	778	N/A	0	NA	WileyRegistry8e
31.67	HAHNFETT	0.25	778	N/A	0	NA	WileyRegistry8e

Compound Structure | Hit Spectrum

ISOCHIAPIN B
Formula C19H22O6, MW 346, CAS# NA, Entry# 225807

SI 808, RSI 812, WileyRegistry8e, Entry# 225807, CAS# NA, ISOCHIAPIN B

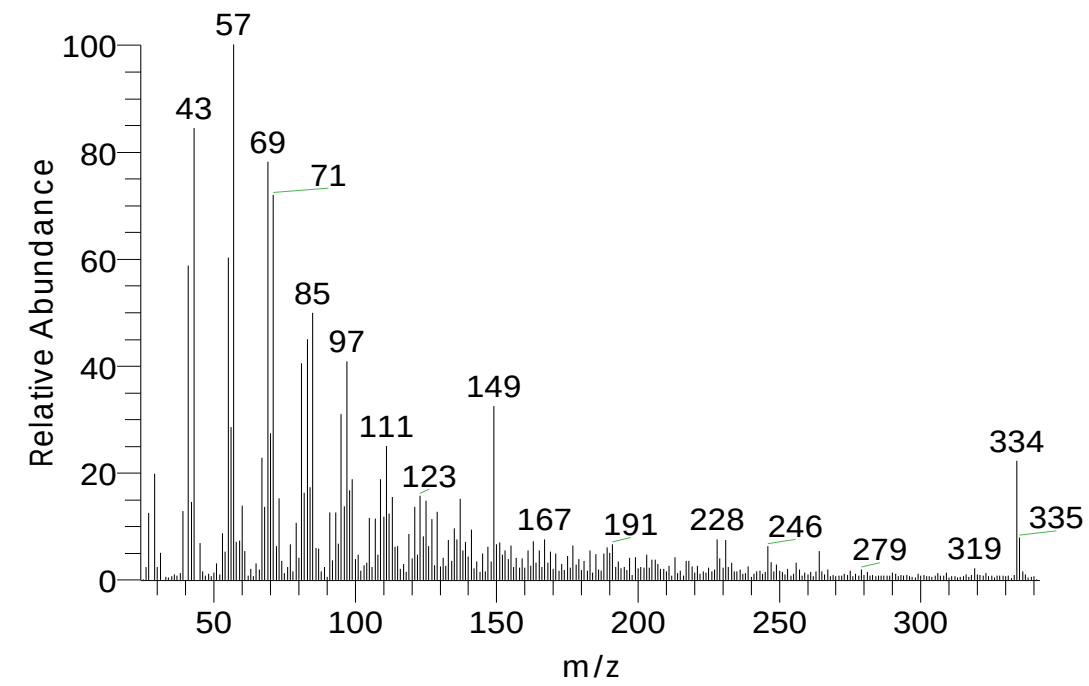

ISOCHIAPIN B %2<
Formula C19H26O6, MW 350, CAS# NA, Entry# 228500

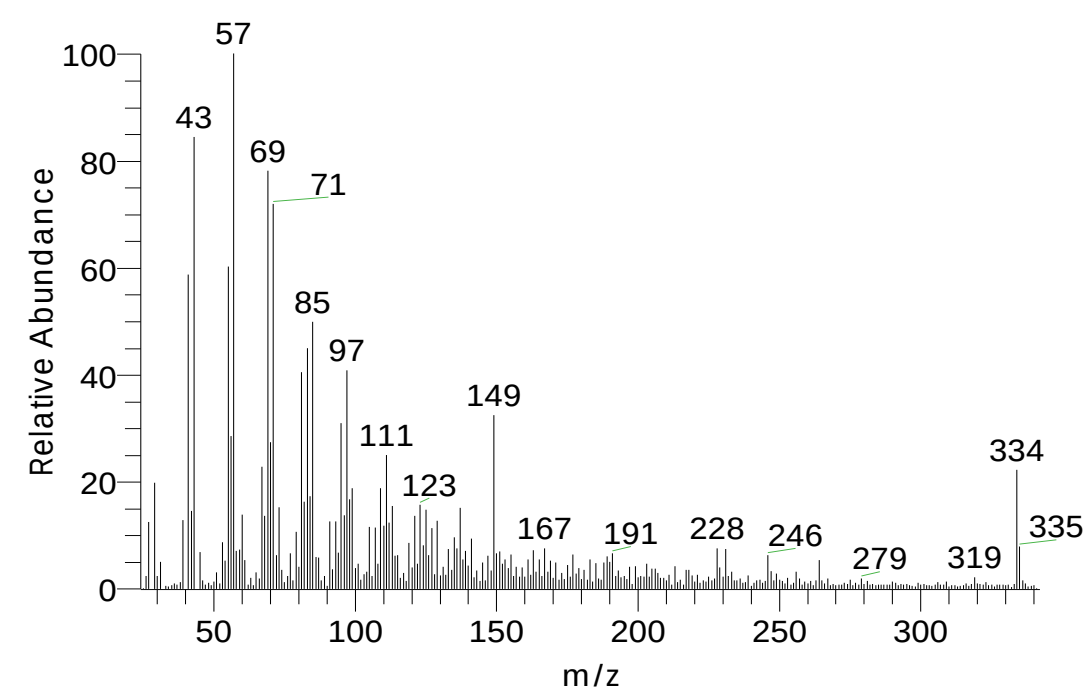

DOTRIACONTANE
Formula C32H66, MW 450, CAS# 544-85-4, Entry# 274478
AI3-52367

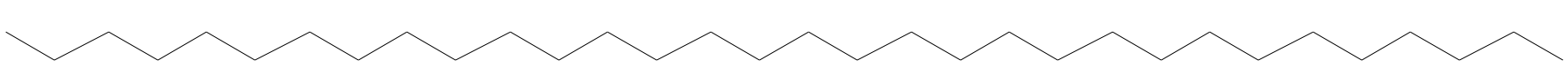

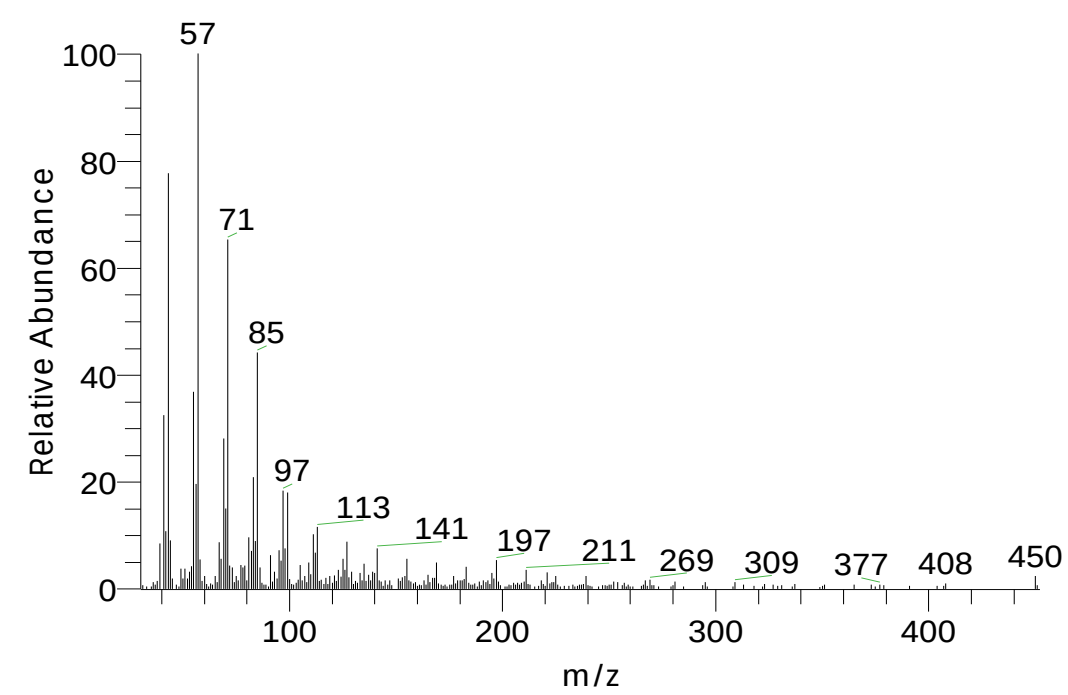

HAHNFETT
Formula , MW 0, CAS# NA, Entry# 305496

SI 771, RSI 778, WileyRegistry8e, Entry# 305496, CAS# NA, HAHNFETT

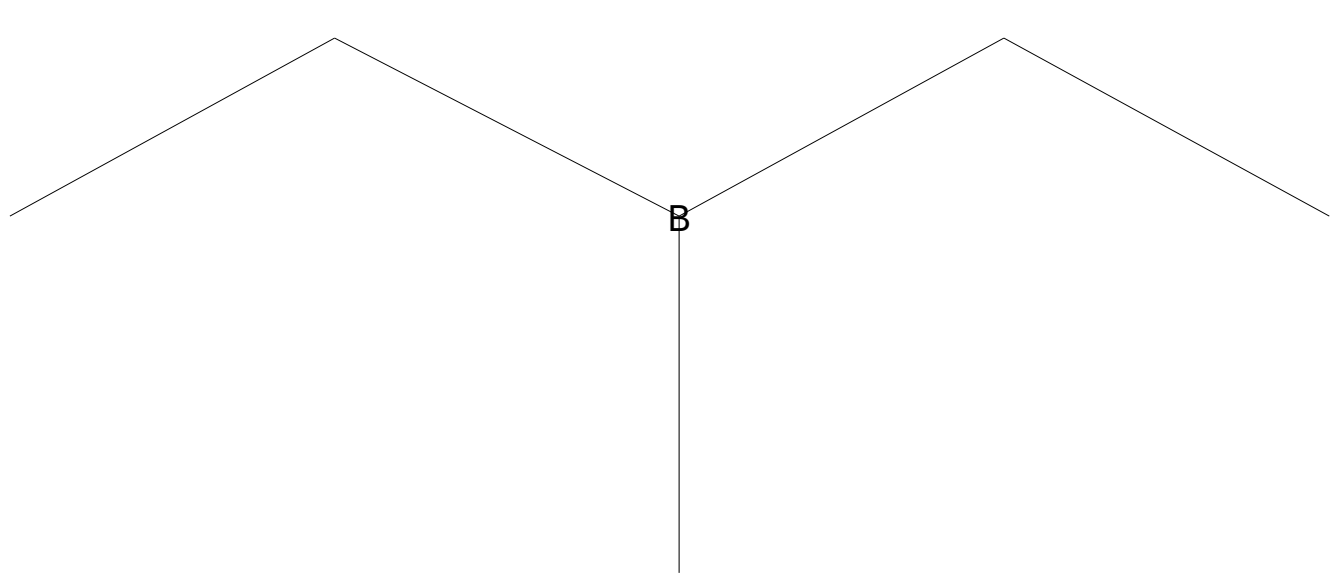

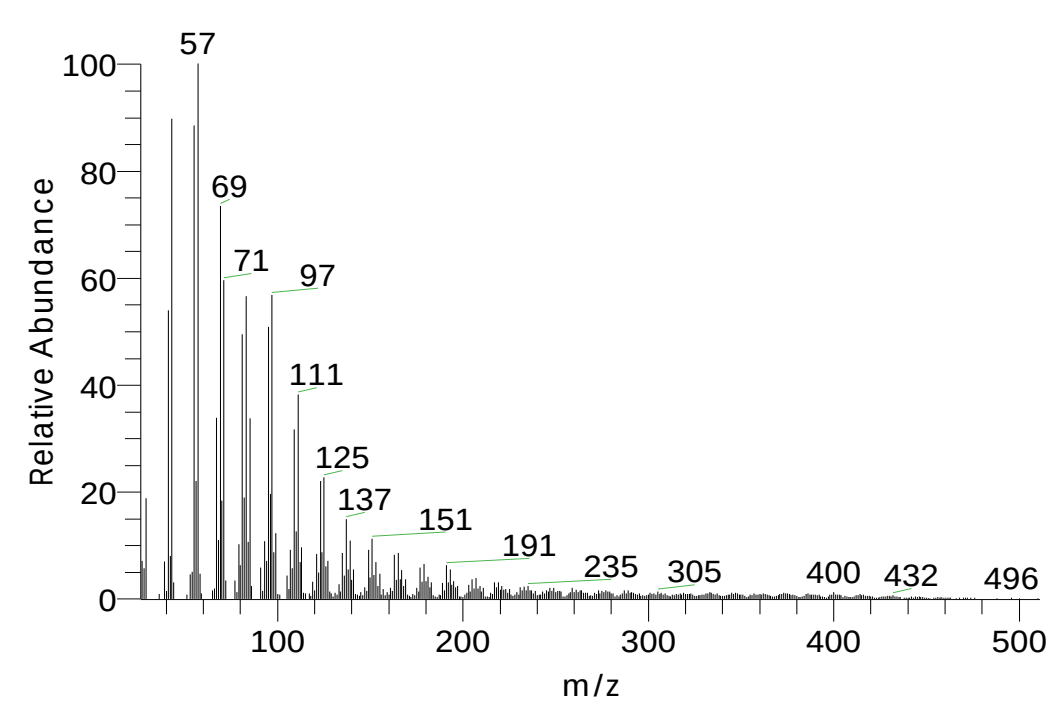

My GC-MS Report

Compound Structure | Hit Spectrum

HAHNFETT
Formula , MW 0, CAS# NA, Entry# 391160

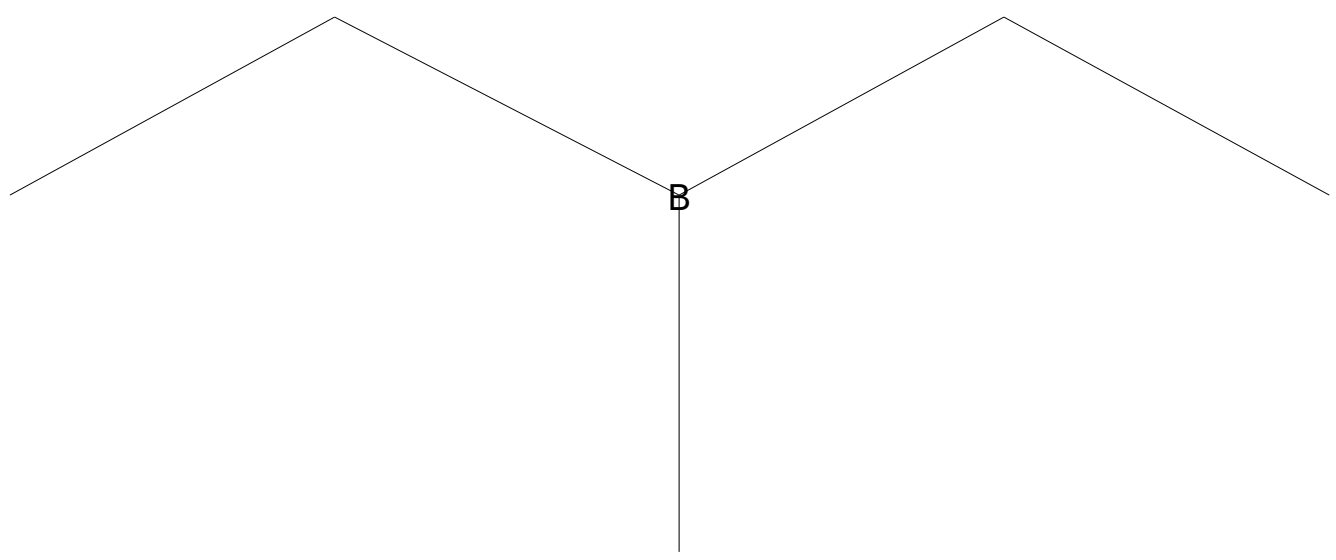

SI 771, RSI 778, WileyRegistry8e, Entry# 391160, CAS# NA, HAHNFETT

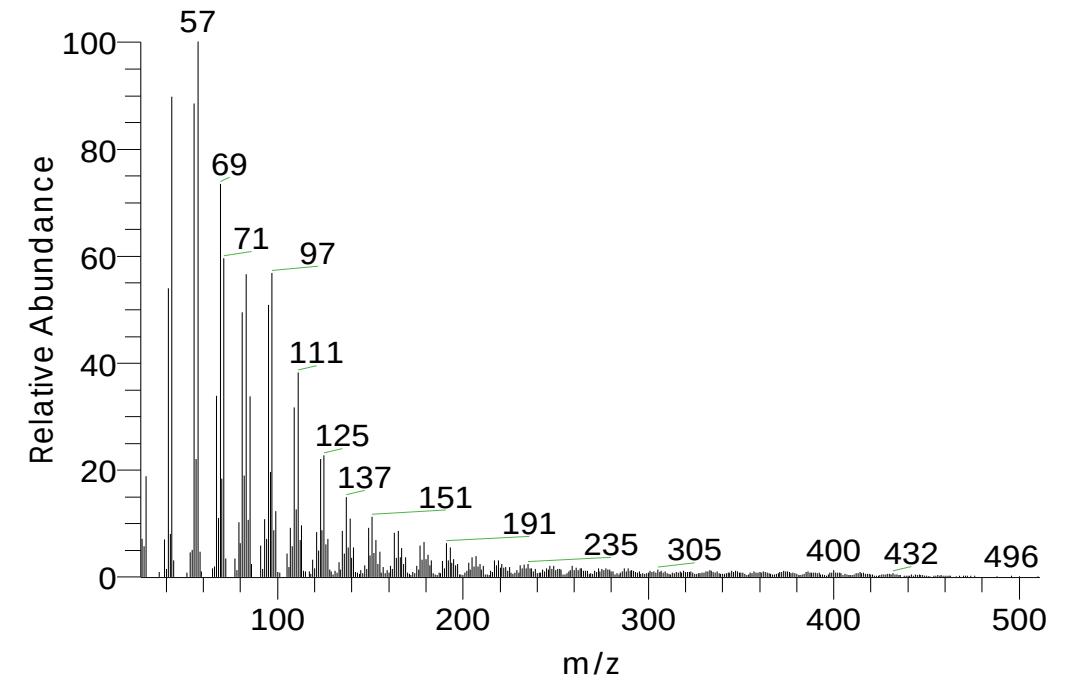

Oil_DrHafsa #8430 RT: 32.27 AV: 1 NL: 6.95E5
T: + c EI Full ms [50.000-750.000]

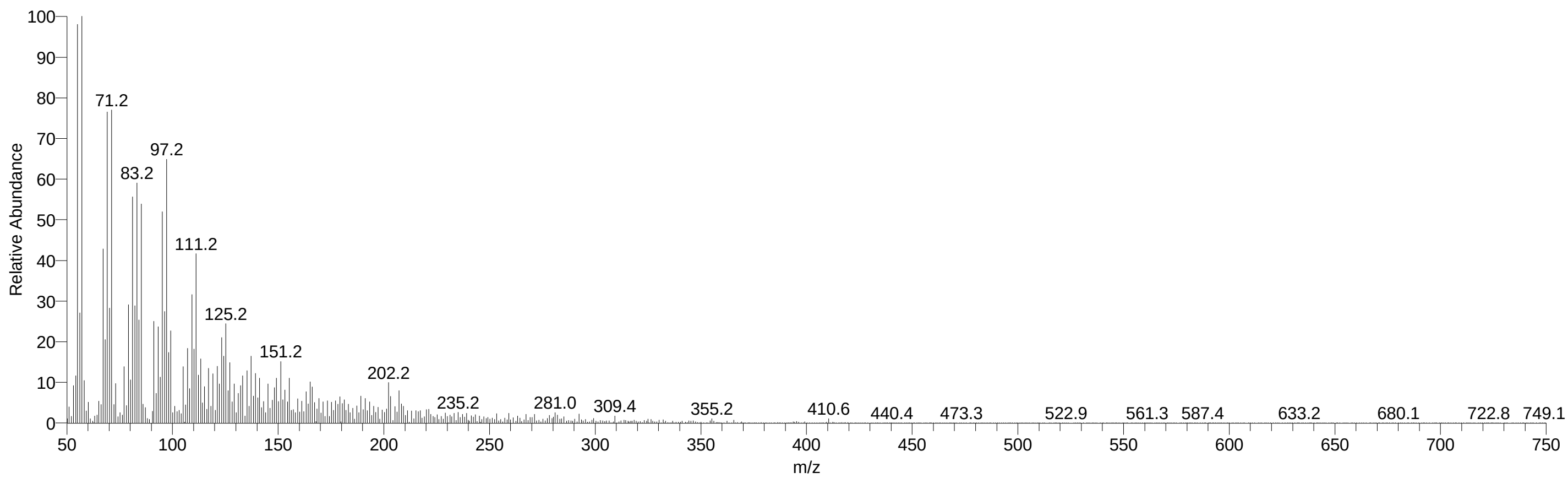

RT	Compound Name	Area %	MF	Molecular Formula	Molecular Weight	Cas #	Library
32.27	HAHNFETT	0.36	785	N/A	0	NA	WileyRegistry8e
32.27	HAHNFETT	0.36	785	N/A	0	NA	WileyRegistry8e
32.27	14-á-H-PREGNA	0.36	813	C21H36	288	NA	WileyRegistry8e
32.27	2-ACETYL-3-(2-CINNAMIDO)ETHYL-7-METHOXYINDOLE	0.36	787	C22H22N2O3	362	NA	WileyRegistry8e
32.27	14-á-H-PREGNA	0.36	772	C21H36	288	NA	WileyRegistry8e

Compound Structure | Hit Spectrum

HAHNFETT
Formula , MW 0, CAS# NA, Entry# 305496

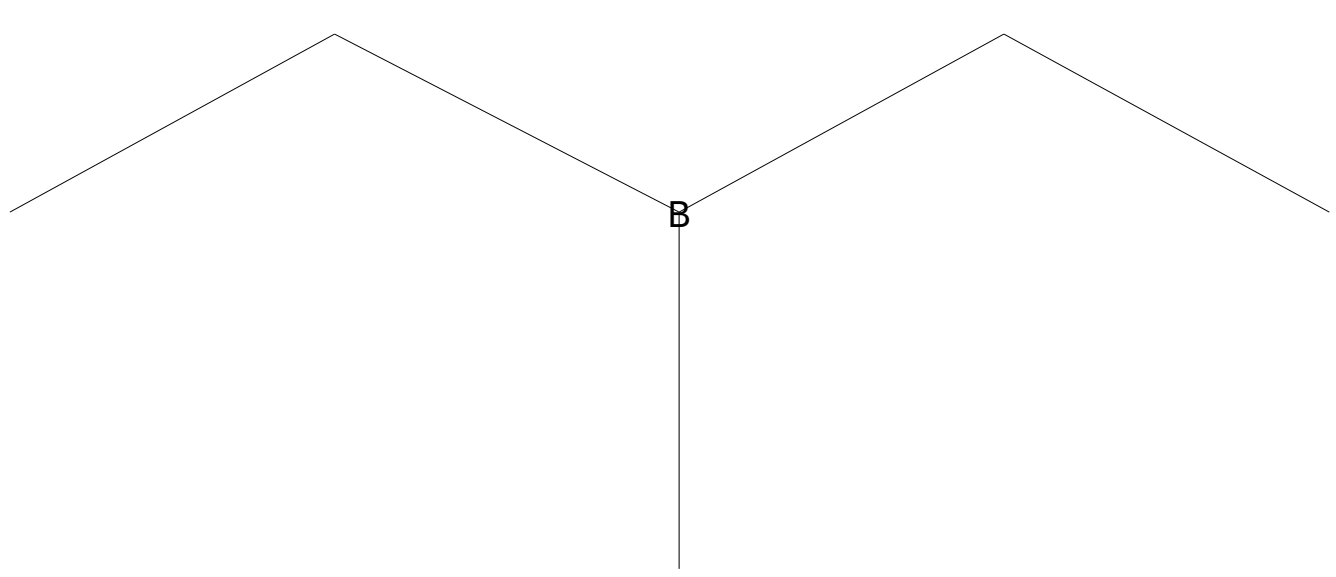

SI 778, RSI 785, WileyRegistry8e, Entry# 305496, CAS# NA, HAHNFETT

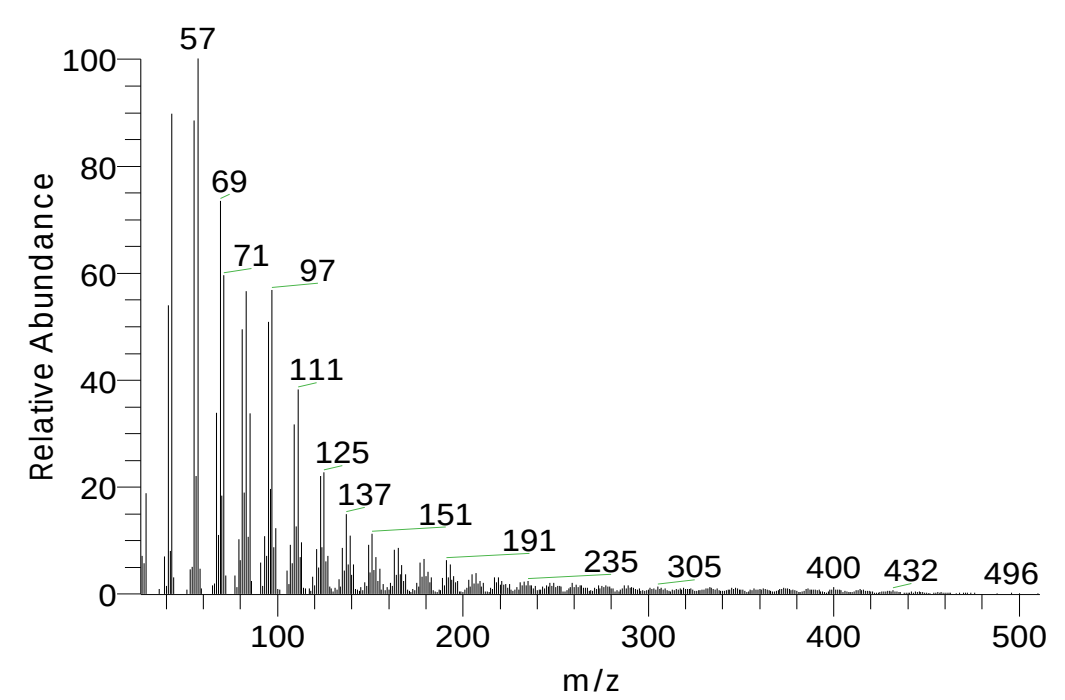

My GC-MS Report

Compound Structure | Hit Spectrum

HAHNFETT
Formula , MW 0, CAS# NA, Entry# 391160

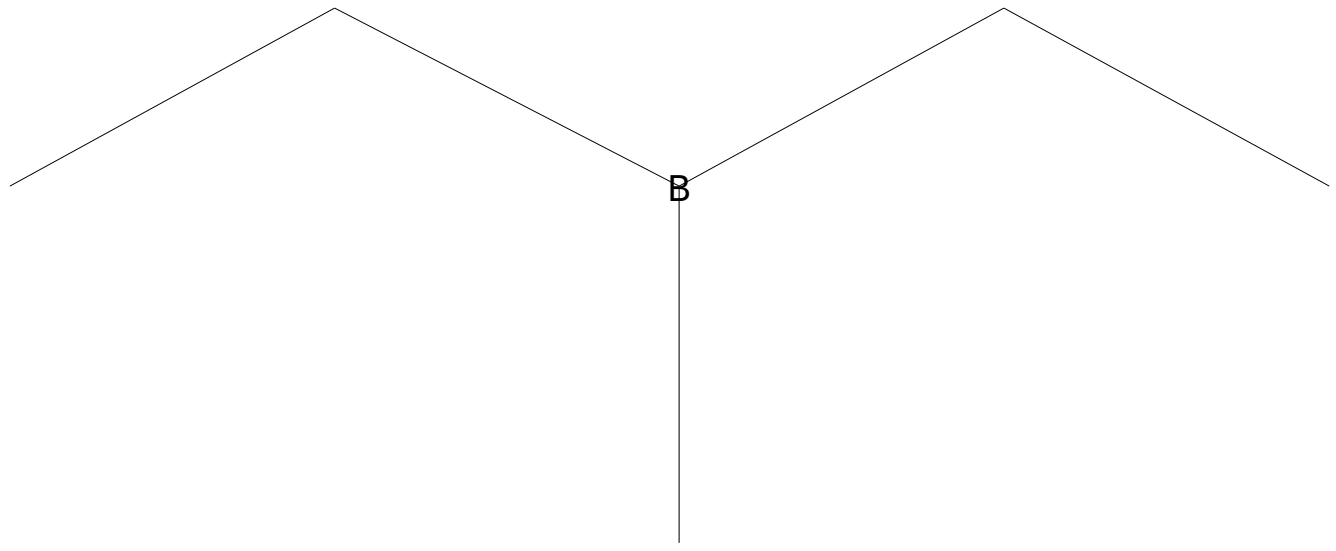

SI 778, RSI 785, WileyRegistry8e, Entry# 391160, CAS# NA, HAHNFETT

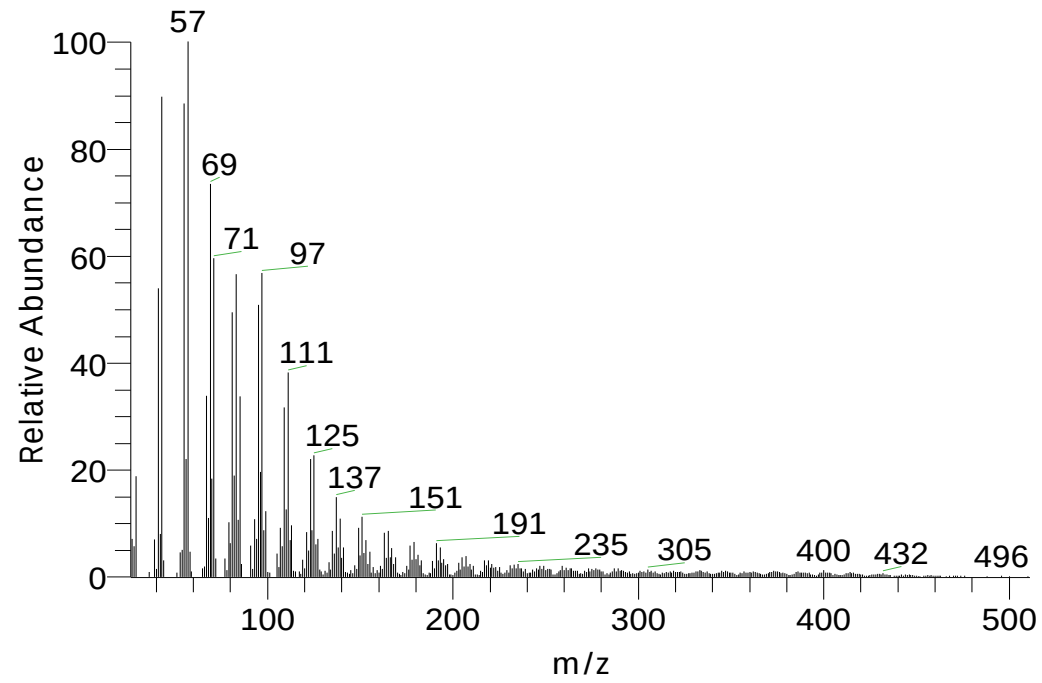

14-á-H-PREGNA
Formula C21H36, MW 288, CAS# NA, Entry# 178939
14-á-PREGNA

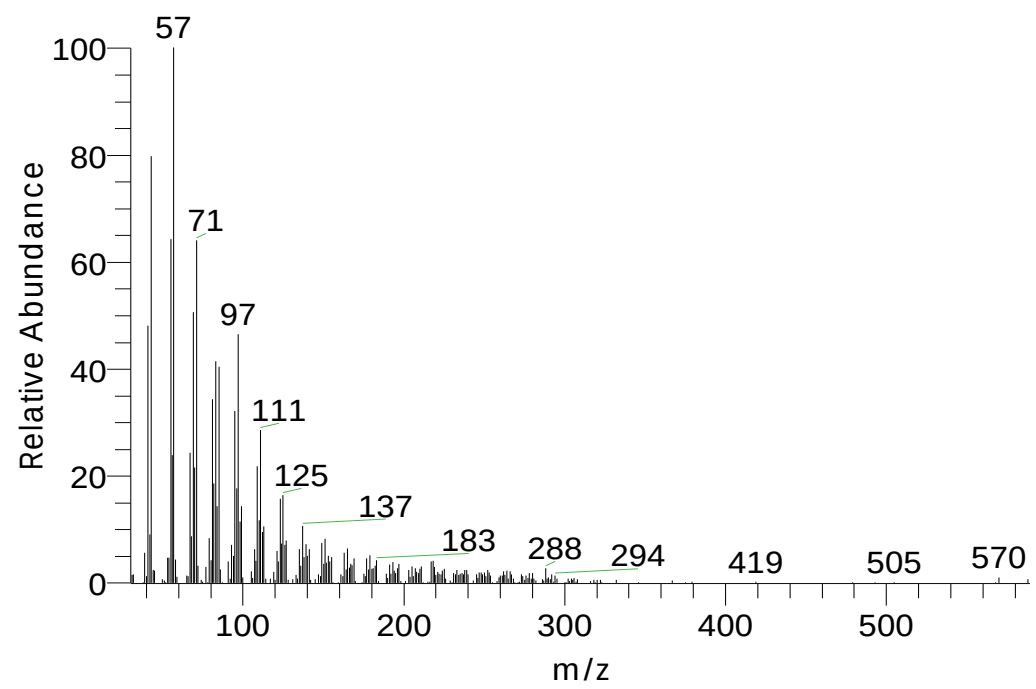

2-ACETYL-3-(2-CINNAMIDO)ETHYL-7-METHOXYINDOLE
Formula C22H22N2O3, MW 362, CAS# NA, Entry# 236392

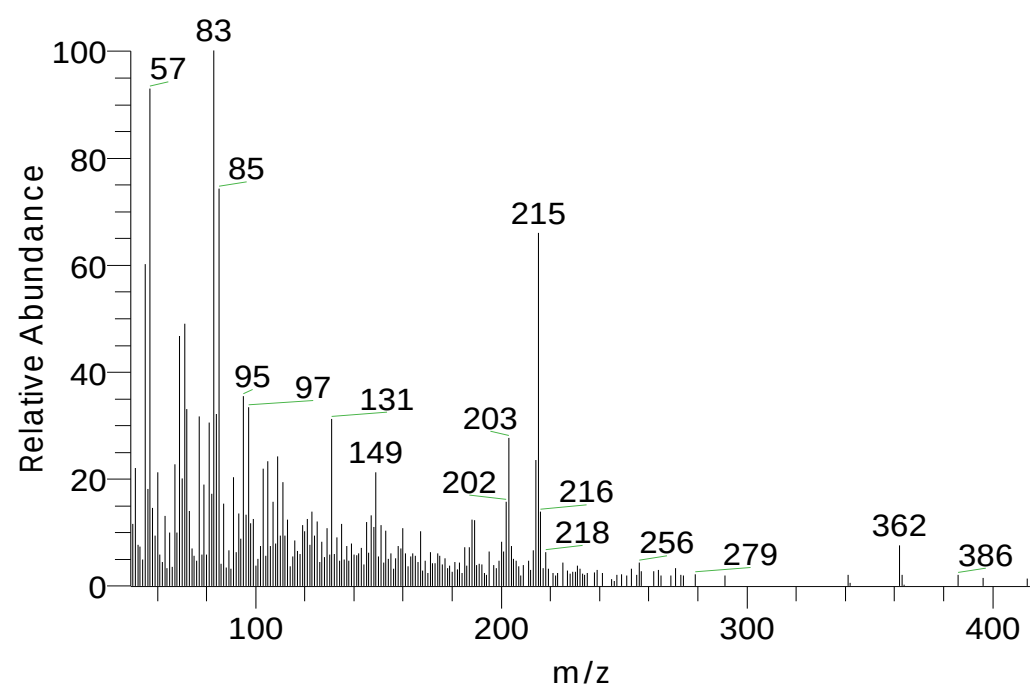

14-á-H-PREGNA
Formula C21H36, MW 288, CAS# NA, Entry# 178938
14-á-PREGNA

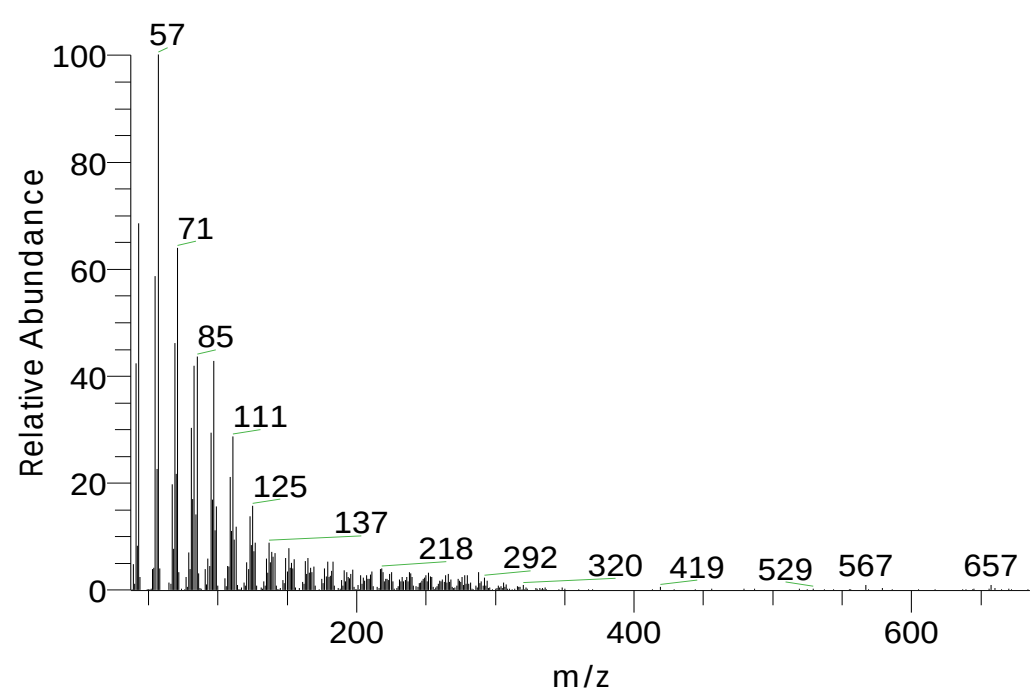

My GC-MS Report

Oil_DrHafsa #8465 RT: 32.39 AV: 1 NL: 1.74E6
T: + c EI Full ms [50.000-750.000]

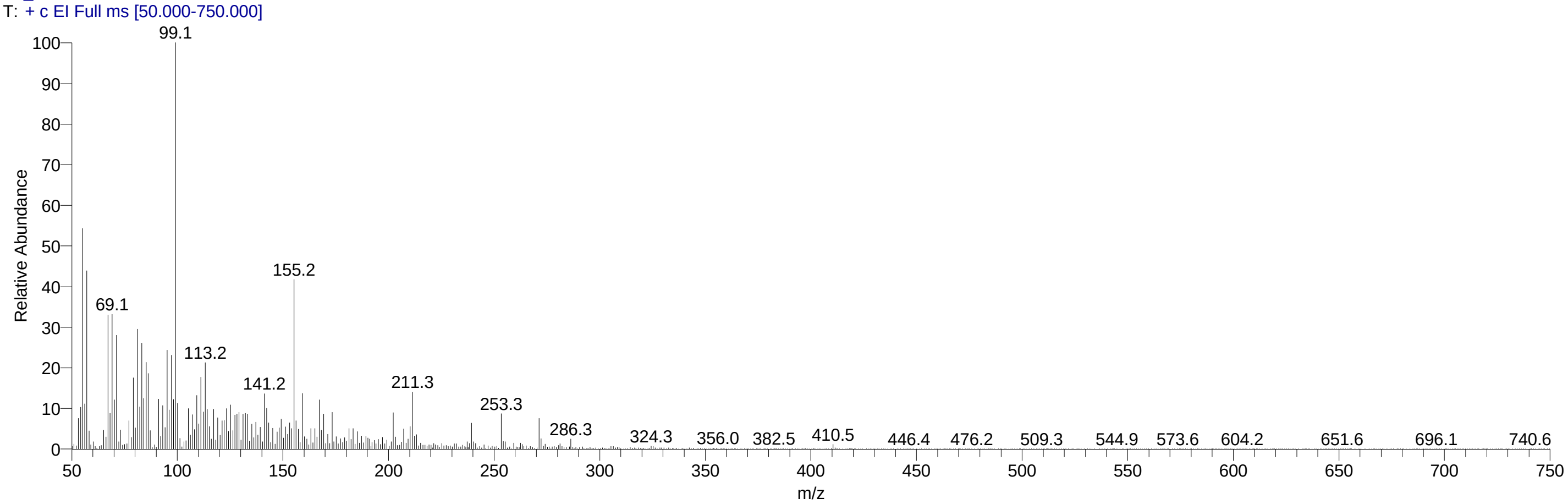

RT	Compound Name	Area %	MF	Molecular Formula	Molecular Weight	Cas #	Library
32.39	OXIRANEOCTANOIC ACID, 3-OCTYL-, CIS-	2.07	719	C18H34O3	298	24560-98-3	WileyRegistry8e
32.39	Oxiraneoctanoic acid, 3-octyl-, cis-	2.07	718	C18H34O3	298	24560-98-3	replib
32.39	CHOLESTAN-3-ONE, CYCLIC 1,2-ETHANEDIYL ACETAL, (5à)-	2.07	722	C29H50O2	430	1858-14-6	WileyRegistry8e
32.39	Cholestan-3-one, cyclic 1,2-ethanediyl aetal, (5á)-	2.07	712	C29H50O2	430	25328-53-4	mainlib
32.39	12-Methyl-E,E-2,13-octadecadien-1-ol	2.07	778	C19H36O	280	NA	mainlib

Compound Structure | Hit Spectrum

OXIRANEOCTANOIC ACID, 3-OCTYL-, CIS-
Formula C18H34O3, MW 298, CAS# 24560-98-3, Entry# 187808
8-(3-OCTYL-2-OXIRANYL)OCTANOIC ACID #

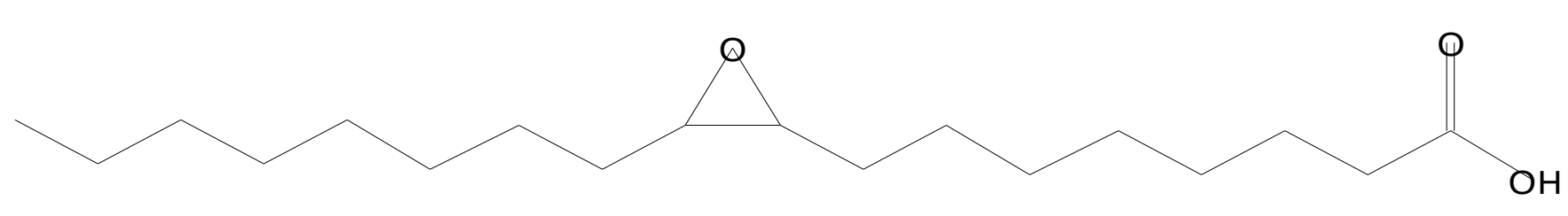

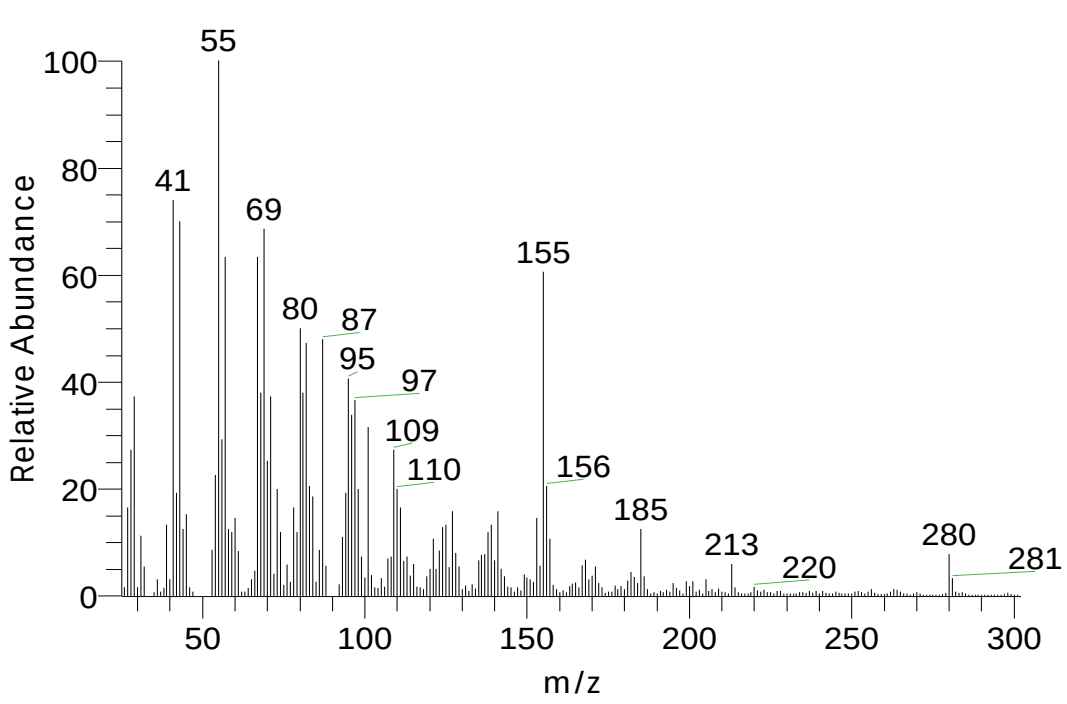

Oxiraneoctanoic acid, 3-octyl-, cis-
Formula C18H34O3, MW 298, CAS# 24560-98-3, Entry# 4626
Octadecanoic acid, 9,10-epoxy-, cis-

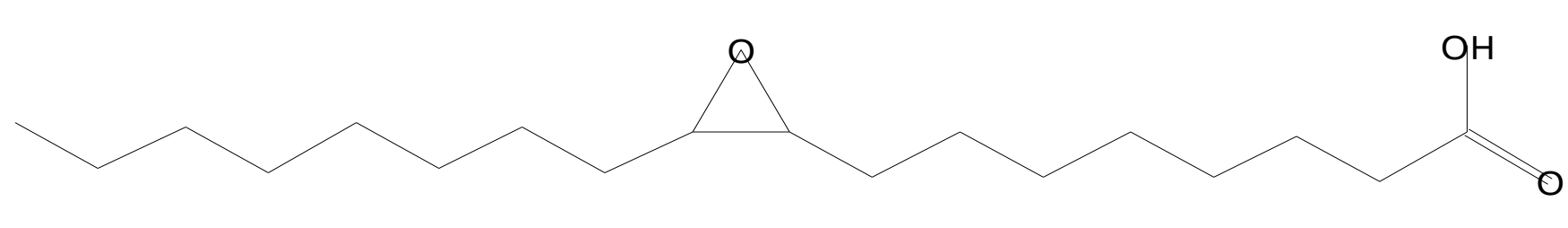

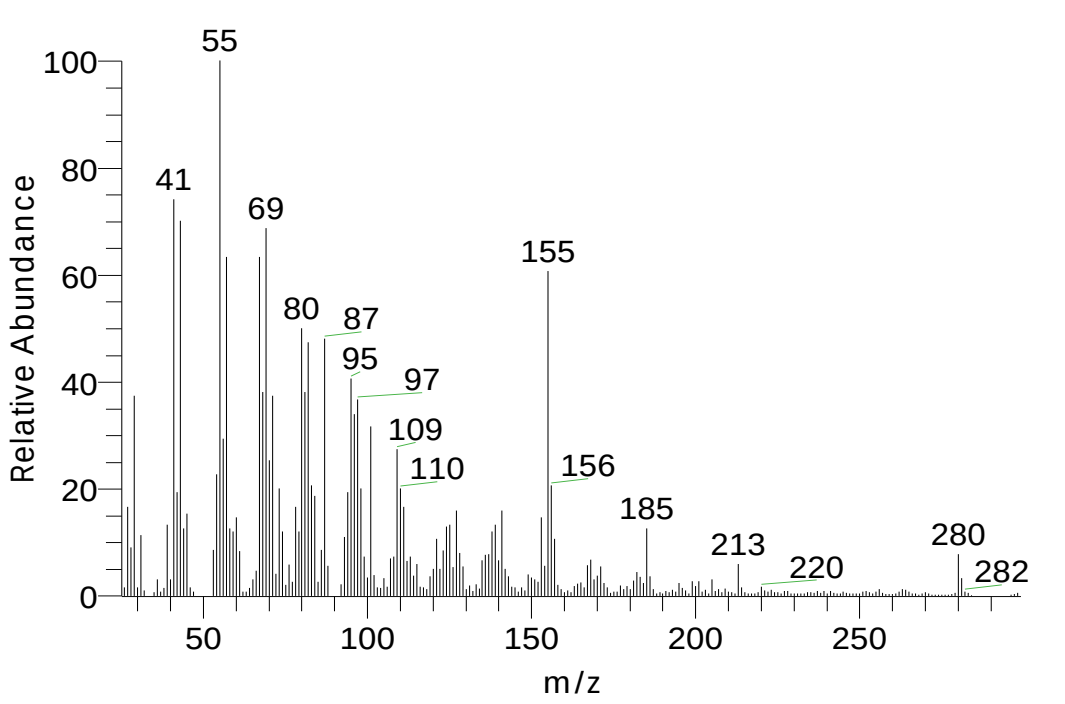

My GC-MS Report

Compound Structure | Hit Spectrum

CHOLESTAN-3-ONE, CYCLIC 1,2-ETHANEDIYL ACETAL, (5à)-
Formula C29H50O2, MW 430, CAS# 1858-14-6, Entry# 268398
3,3-ETHYLENEDIOXY-5à-CHOLESTANE

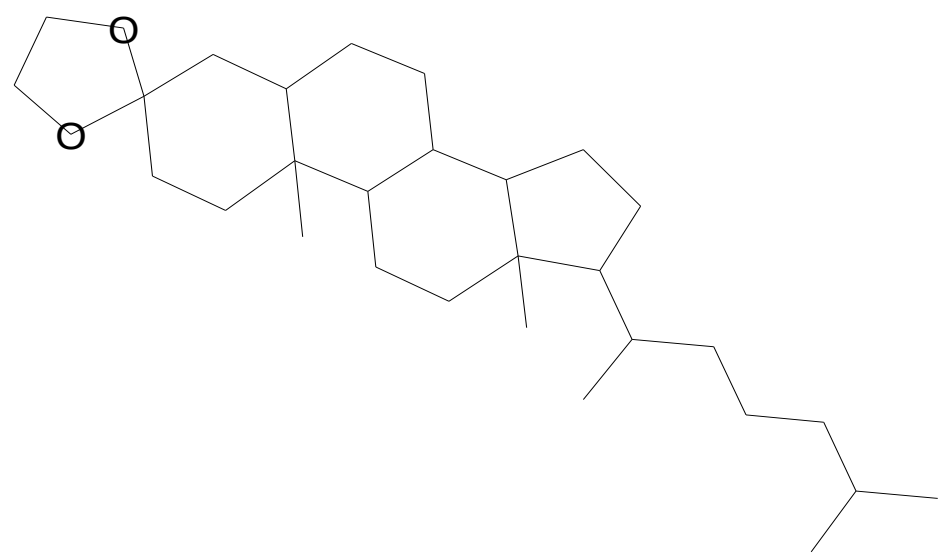

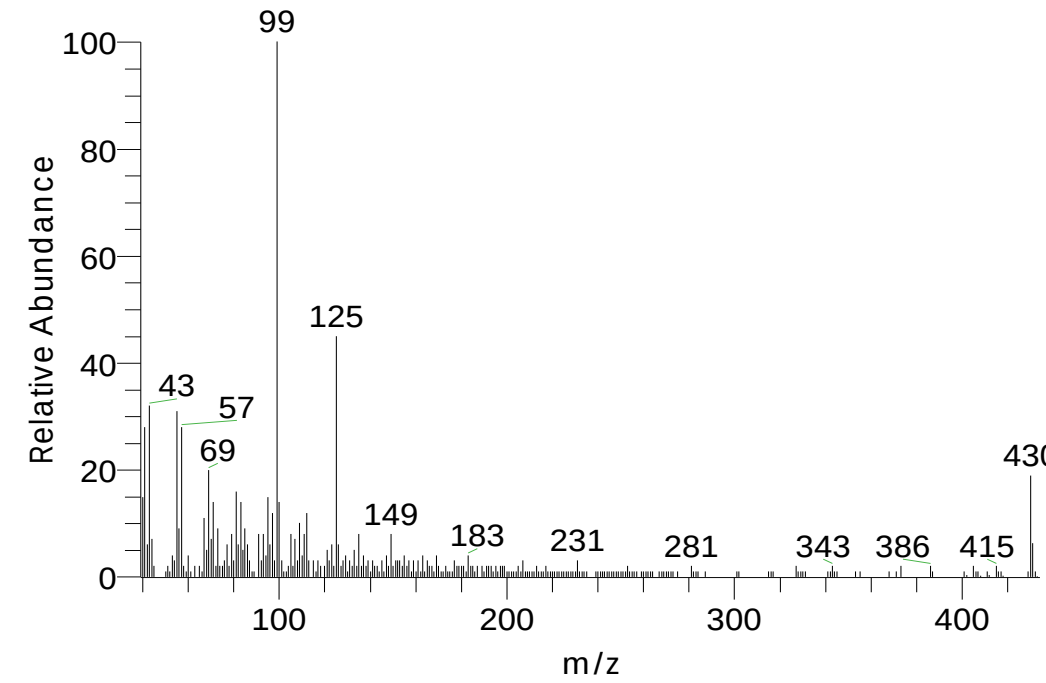

Cholestan-3-one, cyclic 1,2-ethanediyl aetal, (5á)-
Formula C29H50O2, MW 430, CAS# 25328-53-4, Entry# 72469
5á-Cholestan-3-one, cyclic ethylene acetal

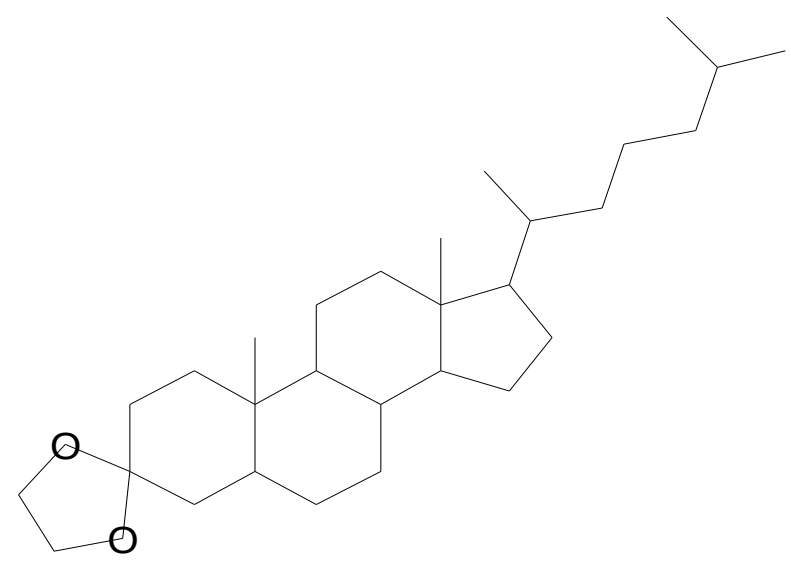

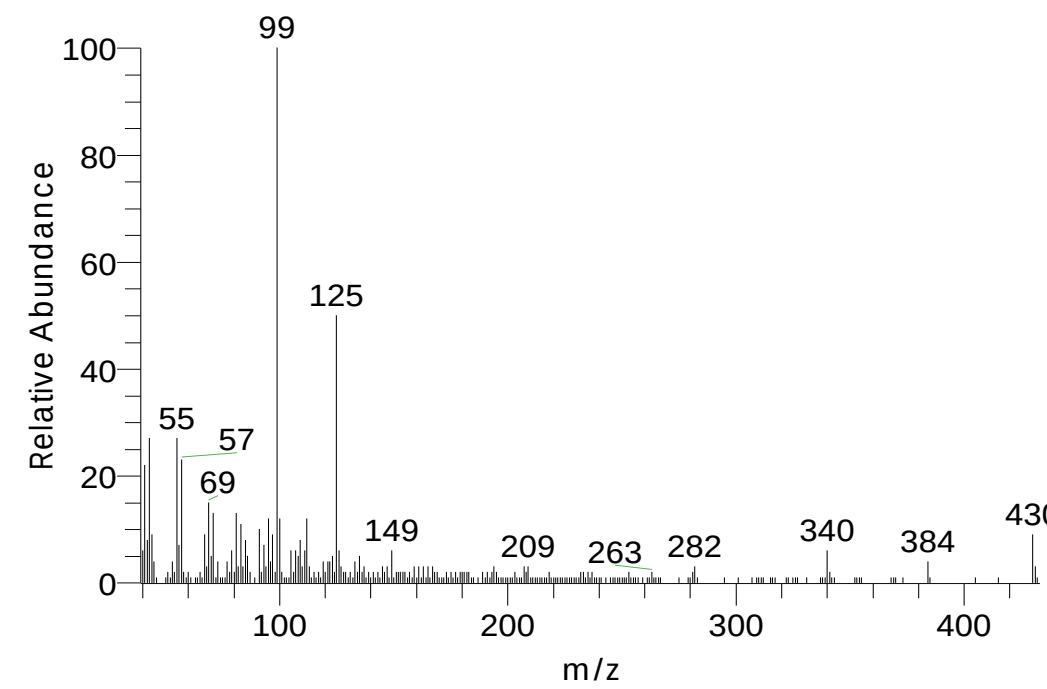

12-Methyl-E,E-2,13-octadecadien-1-ol
Formula C19H36O, MW 280, CAS# NA, Entry# 19016
(2E,15Z)-14-Methyl-2,15-octadecadien-1-ol #

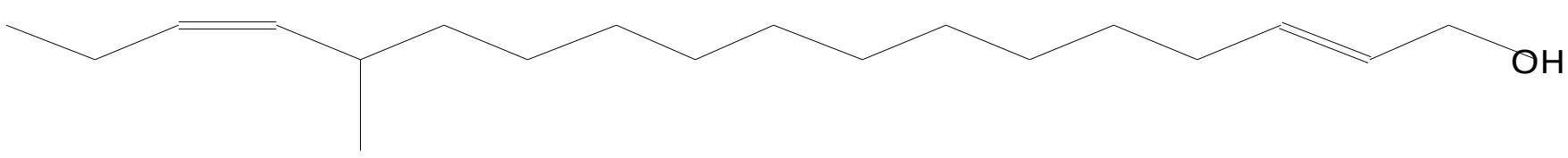

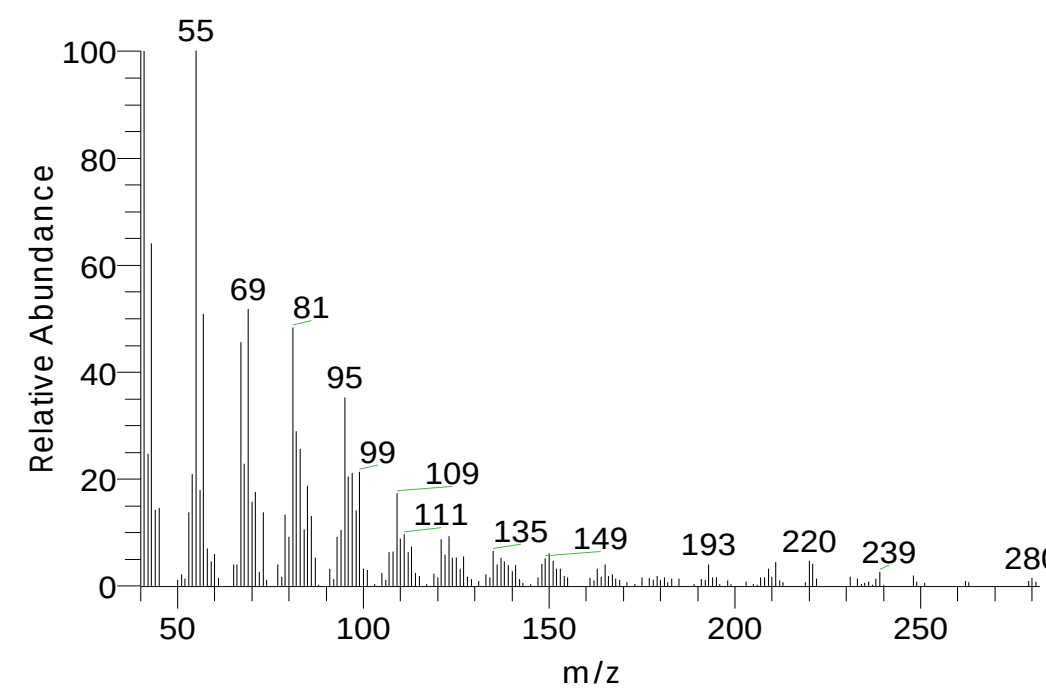

Oil_DrHafsa #8573 RT: 32.75 AV: 1 NL: 8.49E5
T: + c EI Full ms [50.000-750.000]

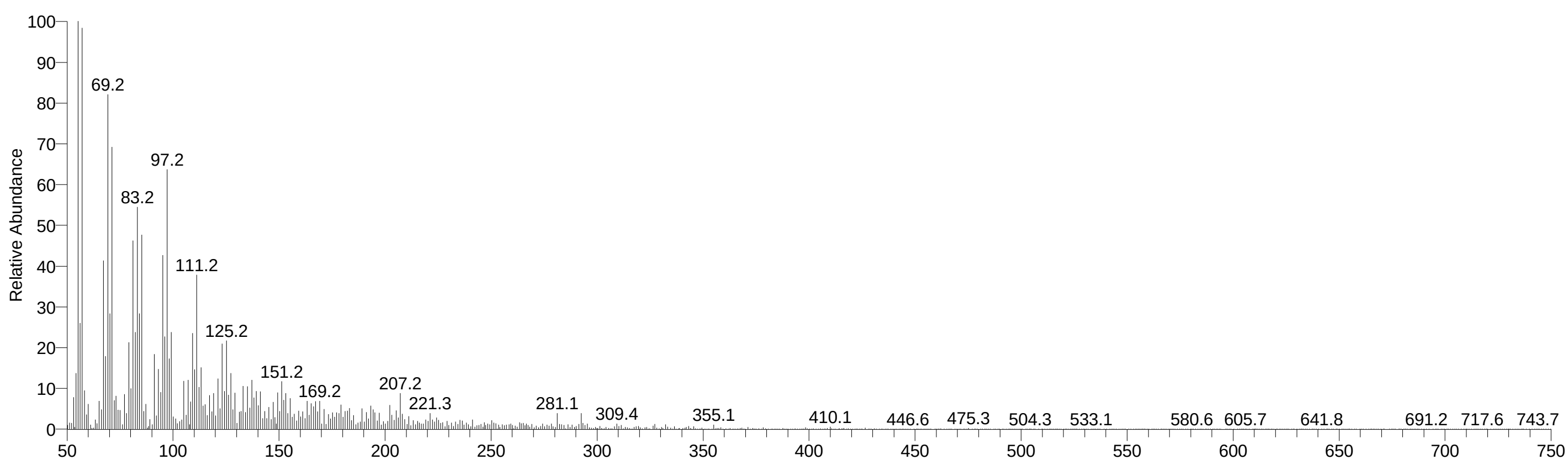

RT	Compound Name	Area %	MF	Molecular Formula	Molecular Weight	Cas #	Library
32.75	HAHNFETT	0.78	790	N/A	0	NA	WileyRegistry8e
32.75	HAHNFETT	0.78	790	N/A	0	NA	WileyRegistry8e

My GC-MS Report

RT	Compound Name	Area %	MF	Molecular Formula	Molecular Weight	Cas #	Library
32.75	14-á-H-PREGNA	0.78	832	C21H36	288	NA	WileyRegistry8e
32.75	14-á-H-PREGNA	0.78	802	C21H36	288	NA	WileyRegistry8e
32.75	cis-13-Eicosenoic acid	0.78	797	C20H38O2	310	17735-94-3	mainlib

Compound Structure	Hit Spectrum

HAHNFETT
Formula , MW 0, CAS# NA, Entry# 305496

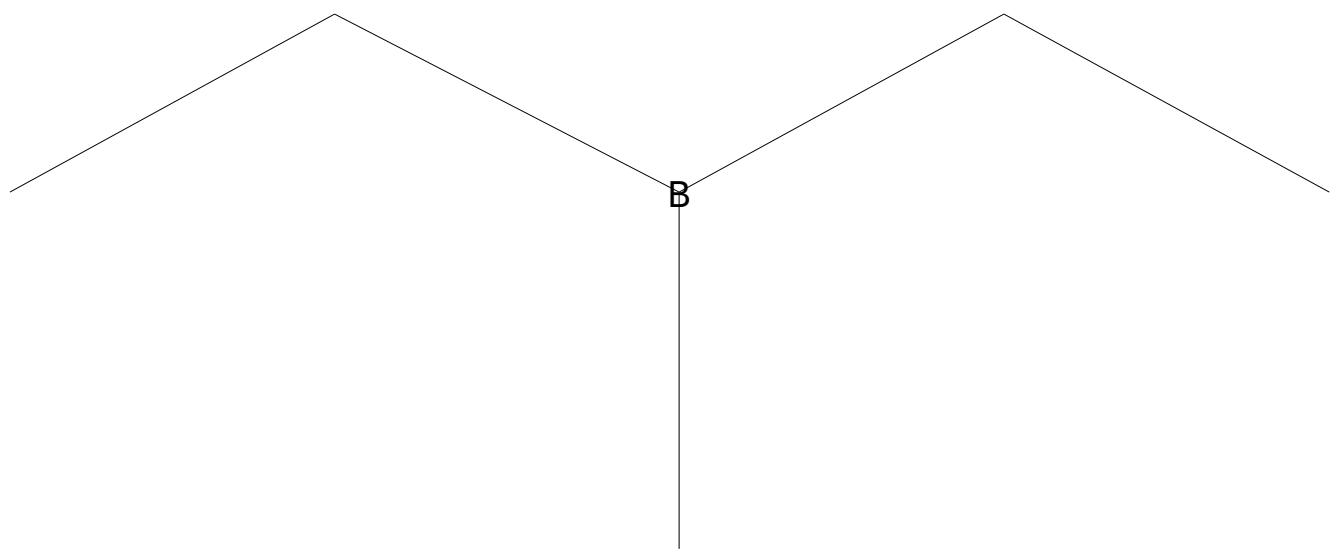

SI 781, RSI 790, WileyRegistry8e, Entry# 305496, CAS# NA, HAHNFETT

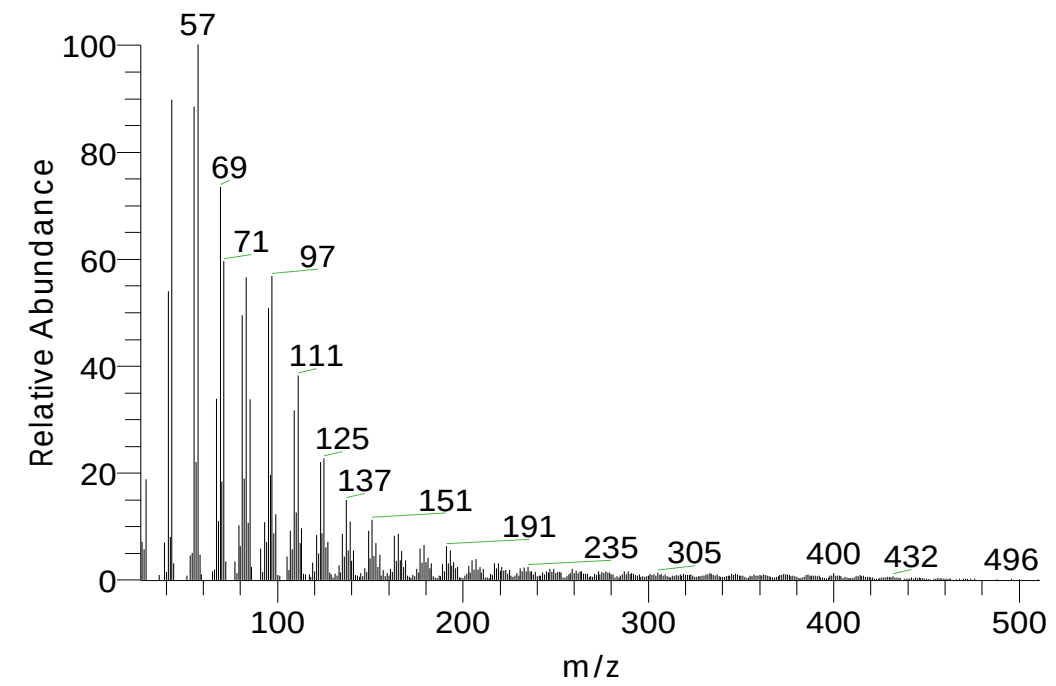

HAHNFETT
Formula , MW 0, CAS# NA, Entry# 391160

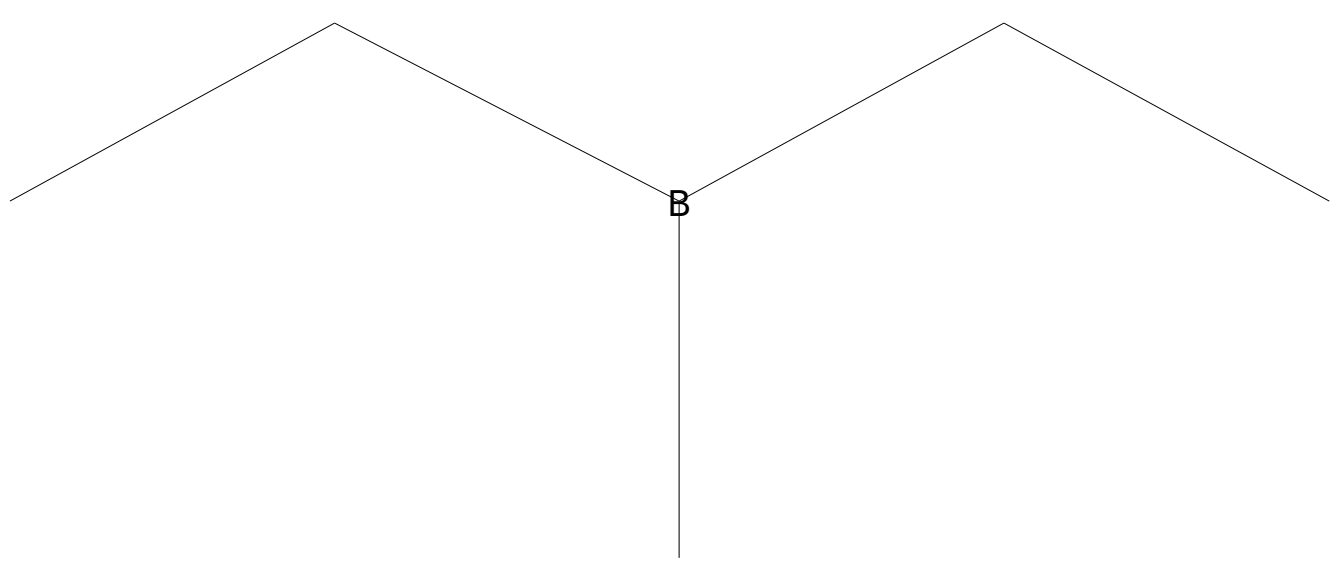

SI 781, RSI 790, WileyRegistry8e, Entry# 391160, CAS# NA, HAHNFETT

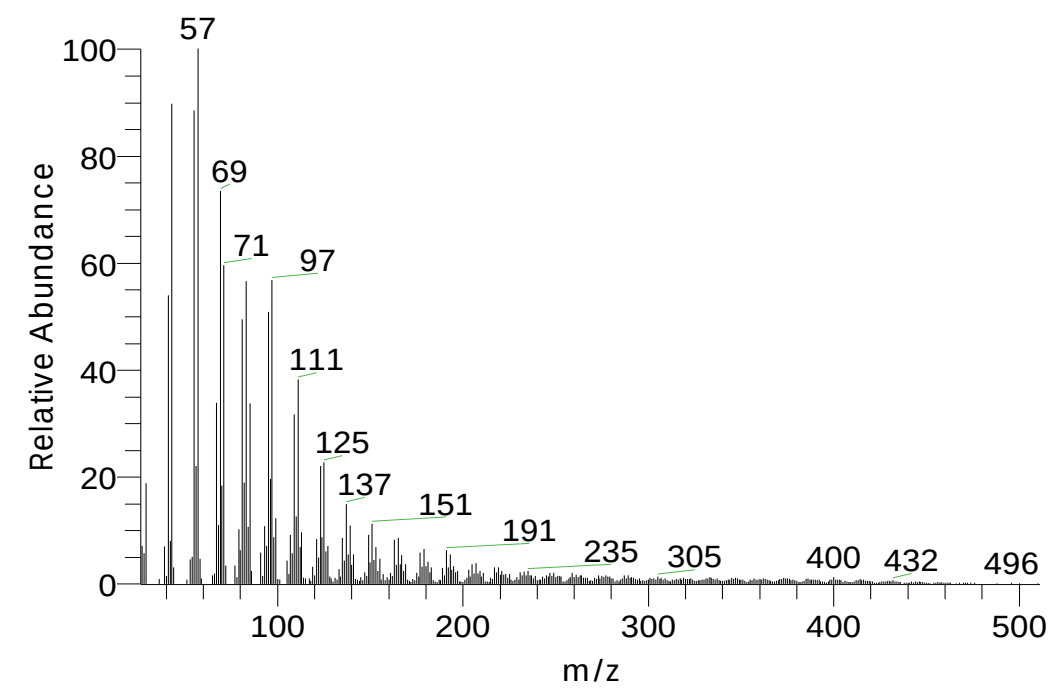

14-á-H-PREGNA
Formula C21H36, MW 288, CAS# NA, Entry# 178939
14-á-PREGNA

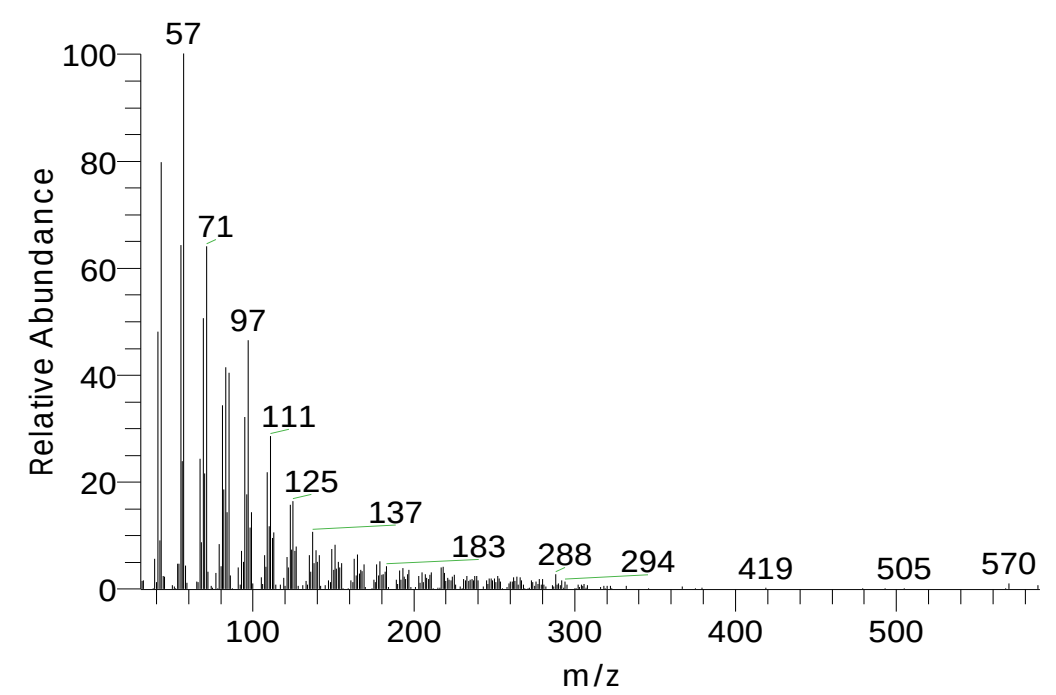

14-á-H-PREGNA
Formula C21H36, MW 288, CAS# NA, Entry# 178938
14-á-PREGNA

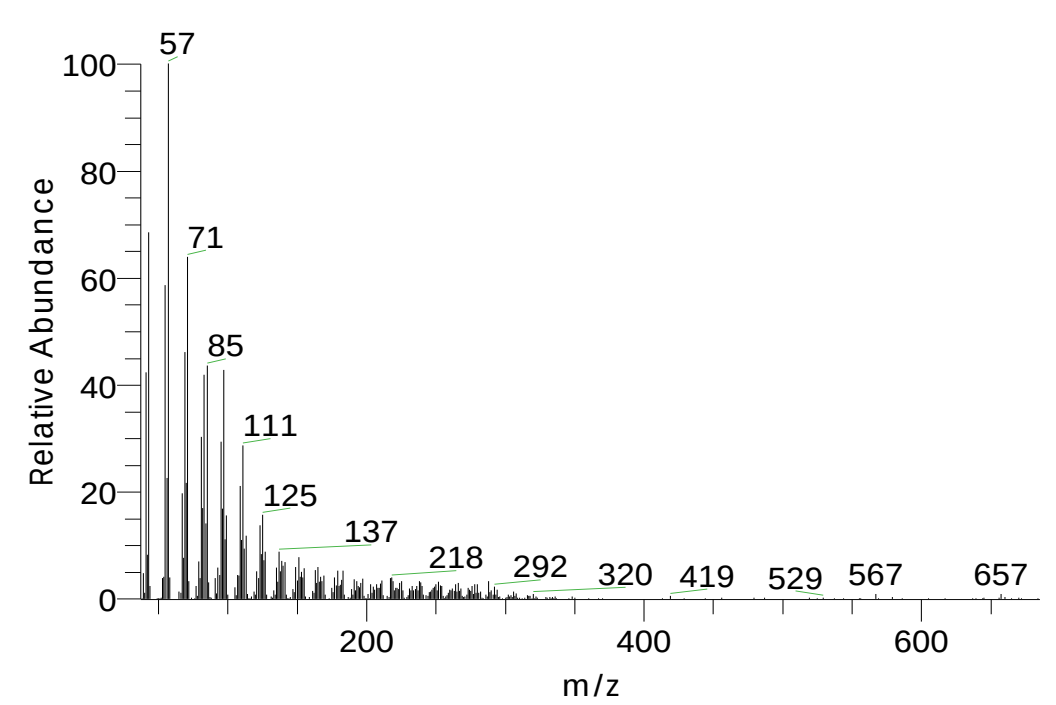

My GC-MS Report

Compound Structure | Hit Spectrum

cis-13-Eicosenoic acid
Formula C20H38O2, MW 310, CAS# 17735-94-3, Entry# 20259
$:28URXZXNYJPAJJOQ-FPLPWBNLSA-N

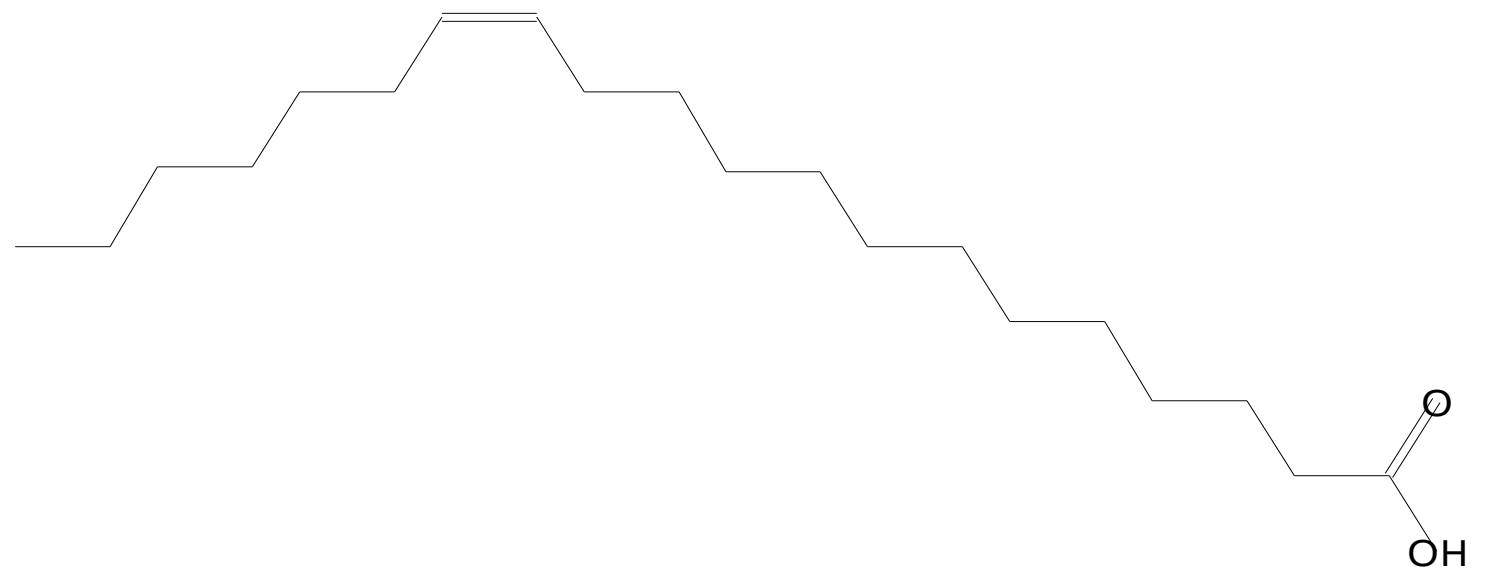

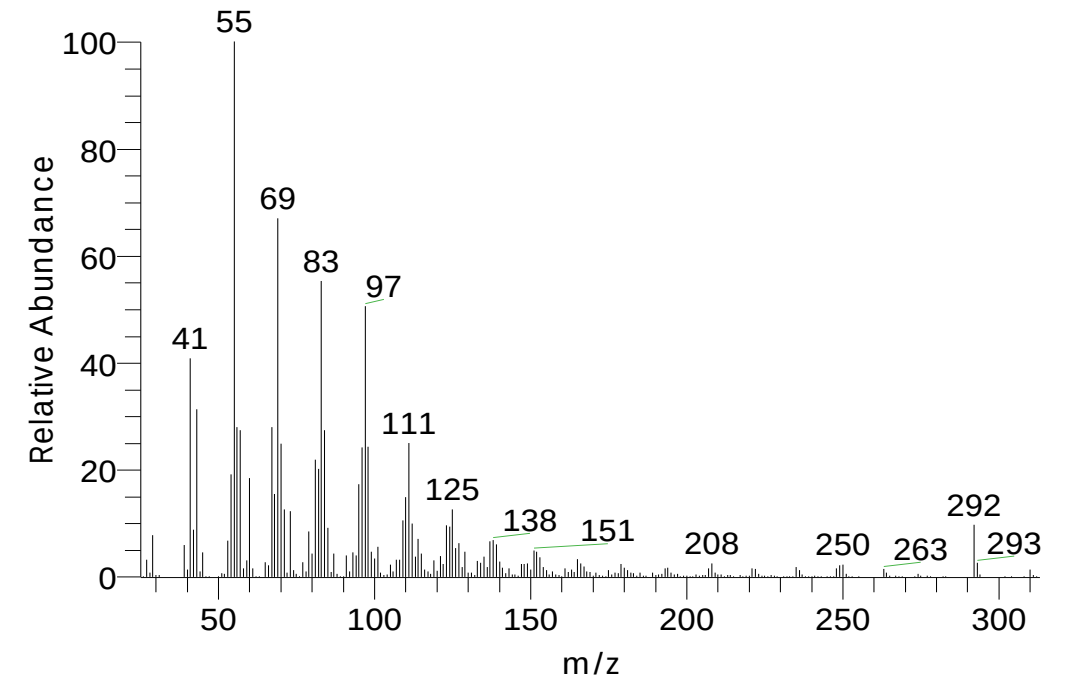

Oil_DrHafsa #8609 RT: 32.87 AV: 1 NL: 5.96E5
T: + c EI Full ms [50.000-750.000]

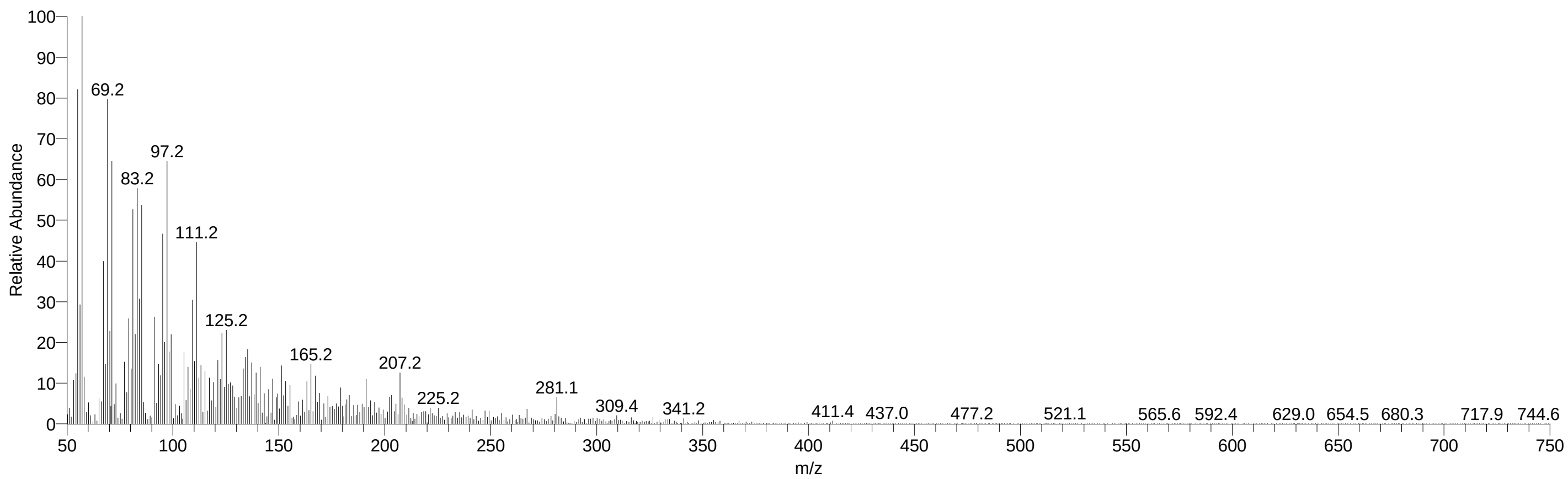

RT	Compound Name	Area %	MF	Molecular Formula	Molecular Weight	Cas #	Library
32.87	HAHNFETT	0.28	778	N/A	0	NA	WileyRegistry8e
32.87	HAHNFETT	0.28	778	N/A	0	NA	WileyRegistry8e
32.87	14-á-H-PREGNA	0.28	800	C21H36	288	NA	WileyRegistry8e
32.87	2-ACETYL-3-(2-CINNAMIDO)ETHYL-7-METHOXYINDOLE	0.28	785	C22H22N2O3	362	NA	WileyRegistry8e
32.87	14-á-H-PREGNA	0.28	766	C21H36	288	NA	WileyRegistry8e

Compound Structure | Hit Spectrum

HAHNFETT
Formula , MW 0, CAS# NA, Entry# 305496

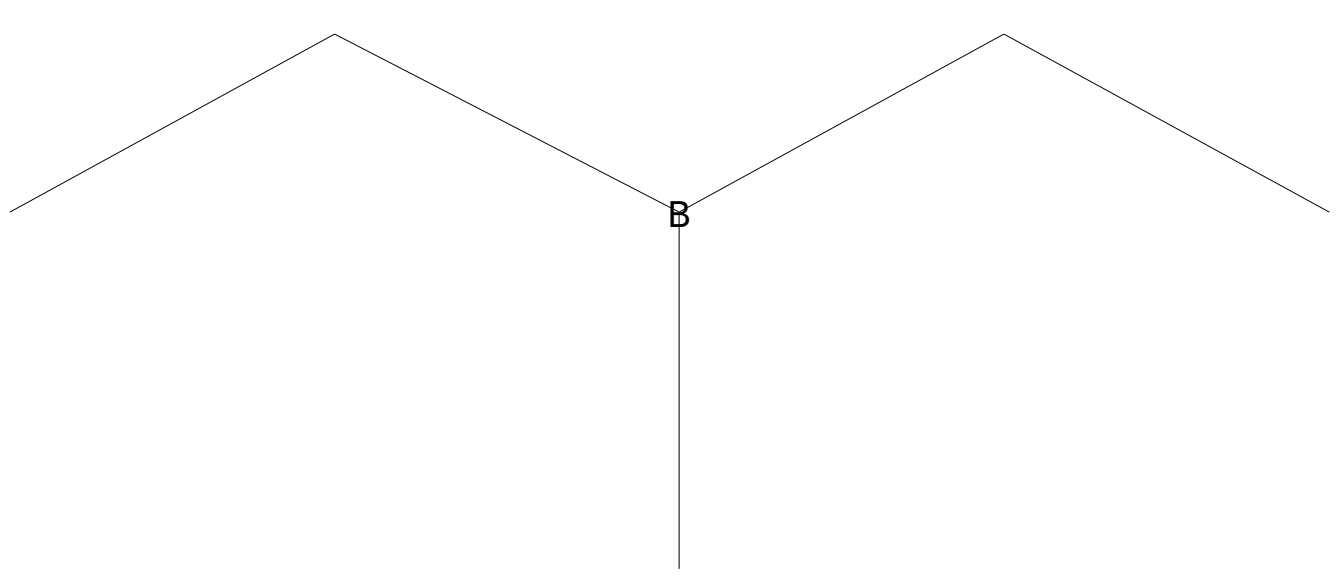

SI 772, RSI 778, WileyRegistry8e, Entry# 305496, CAS# NA, HAHNFETT

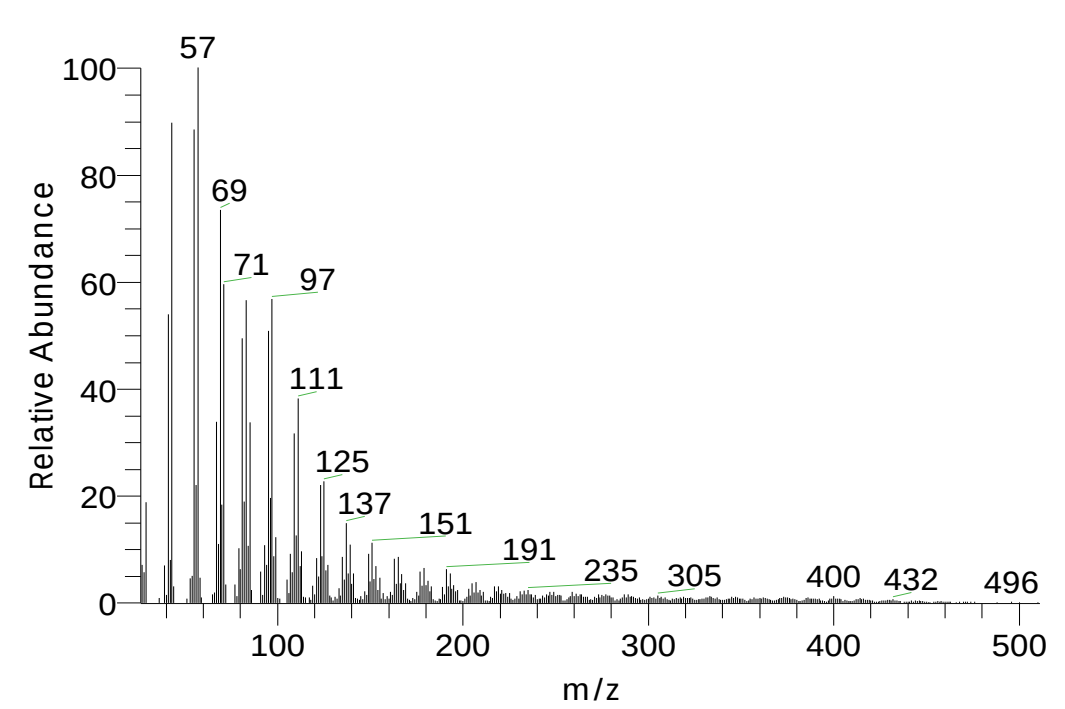

My GC-MS Report

Compound Structure | Hit Spectrum

HAHNFETT
Formula , MW 0, CAS# NA, Entry# 391160

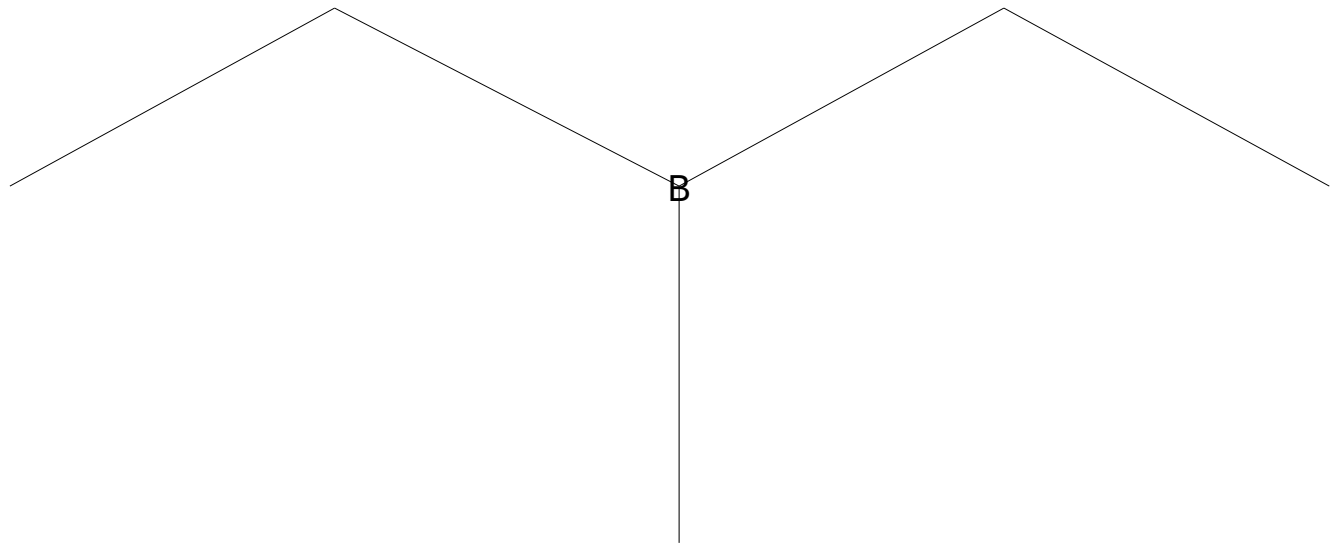

SI 772, RSI 778, WileyRegistry8e, Entry# 391160, CAS# NA, HAHNFETT

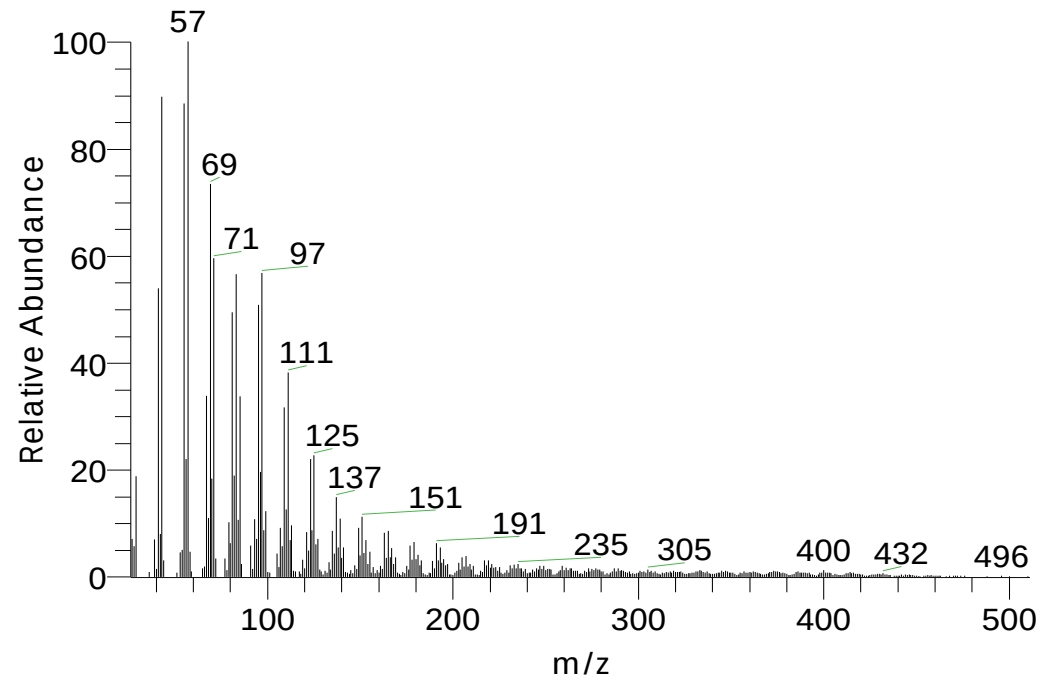

14-á-H-PREGNA
Formula C21H36, MW 288, CAS# NA, Entry# 178939
14-á-PREGNA

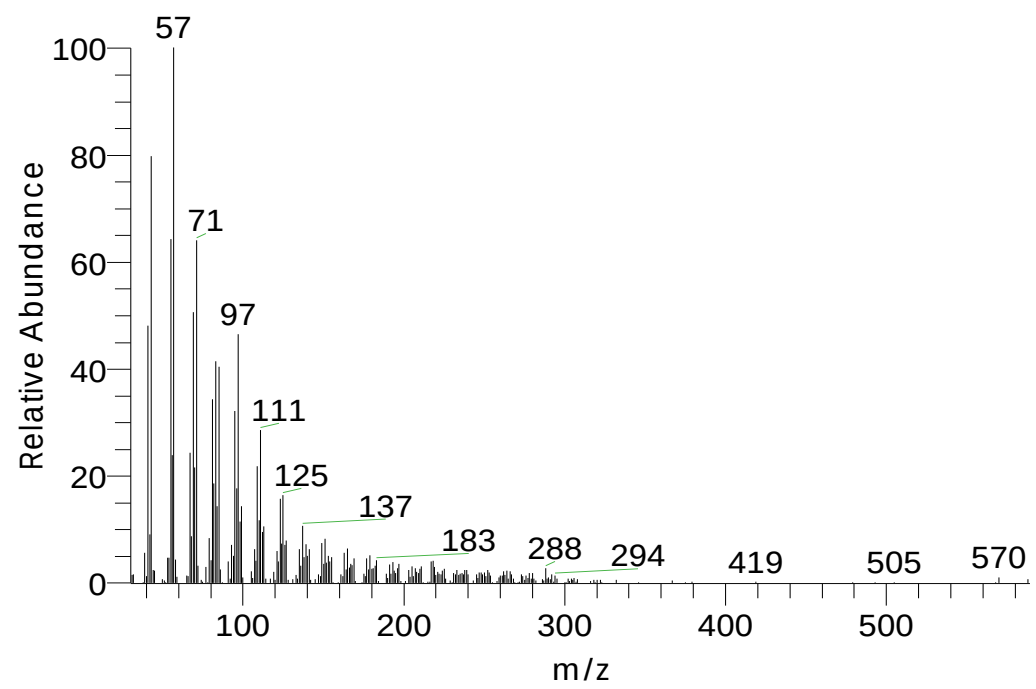

2-ACETYL-3-(2-CINNAMIDO)ETHYL-7-METHOXYINDOLE
Formula C22H22N2O3, MW 362, CAS# NA, Entry# 236392

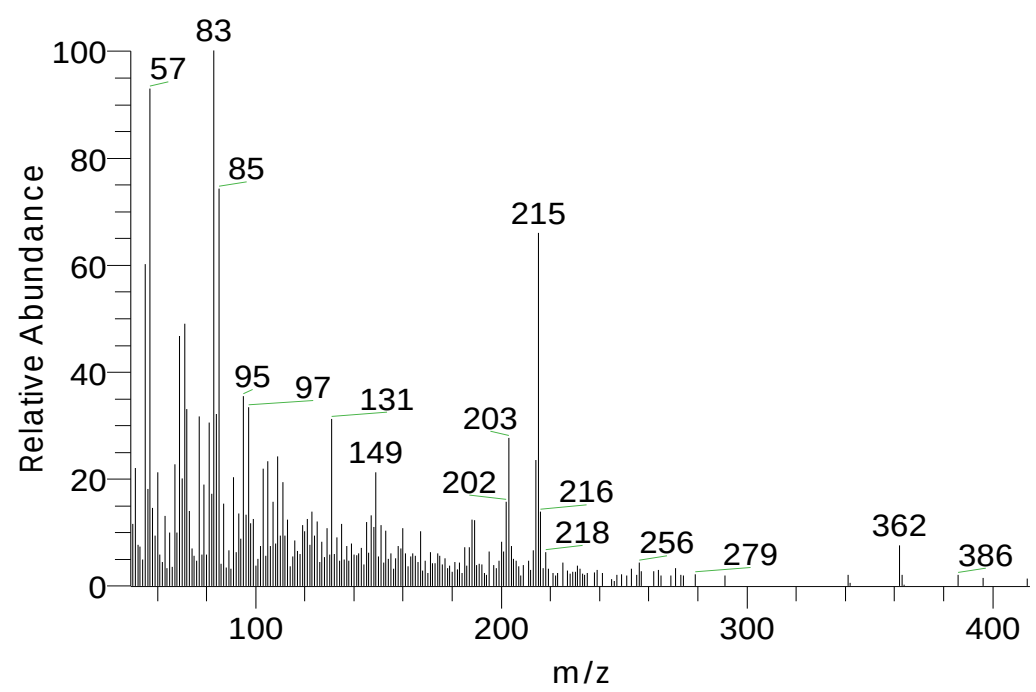

14-á-H-PREGNA
Formula C21H36, MW 288, CAS# NA, Entry# 178938
14-á-PREGNA

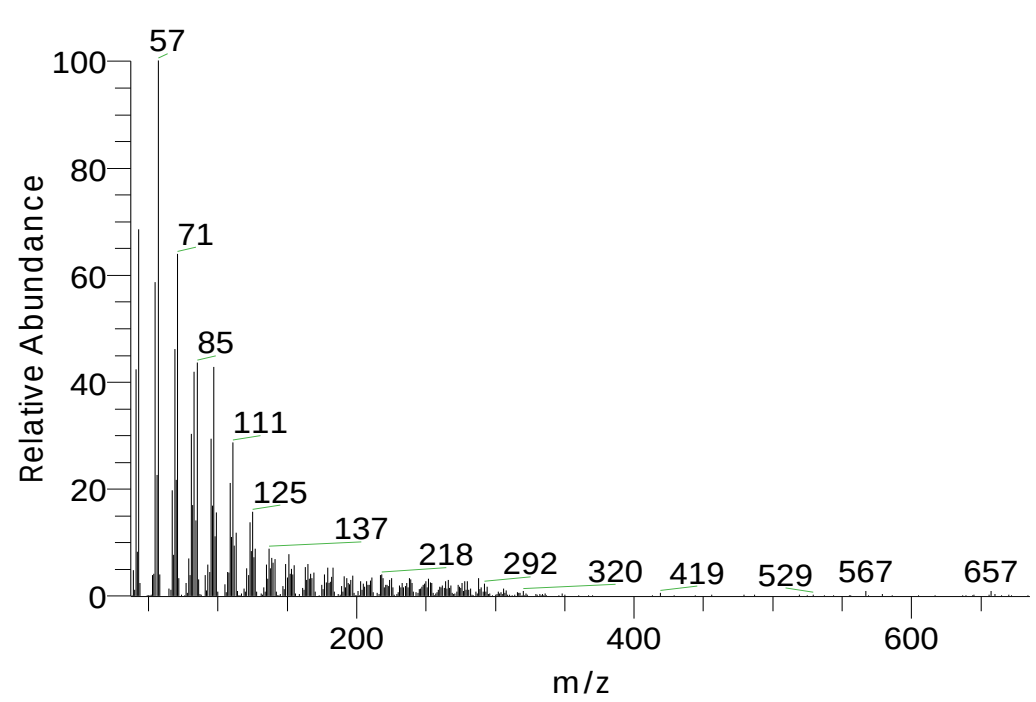

My GC-MS Report

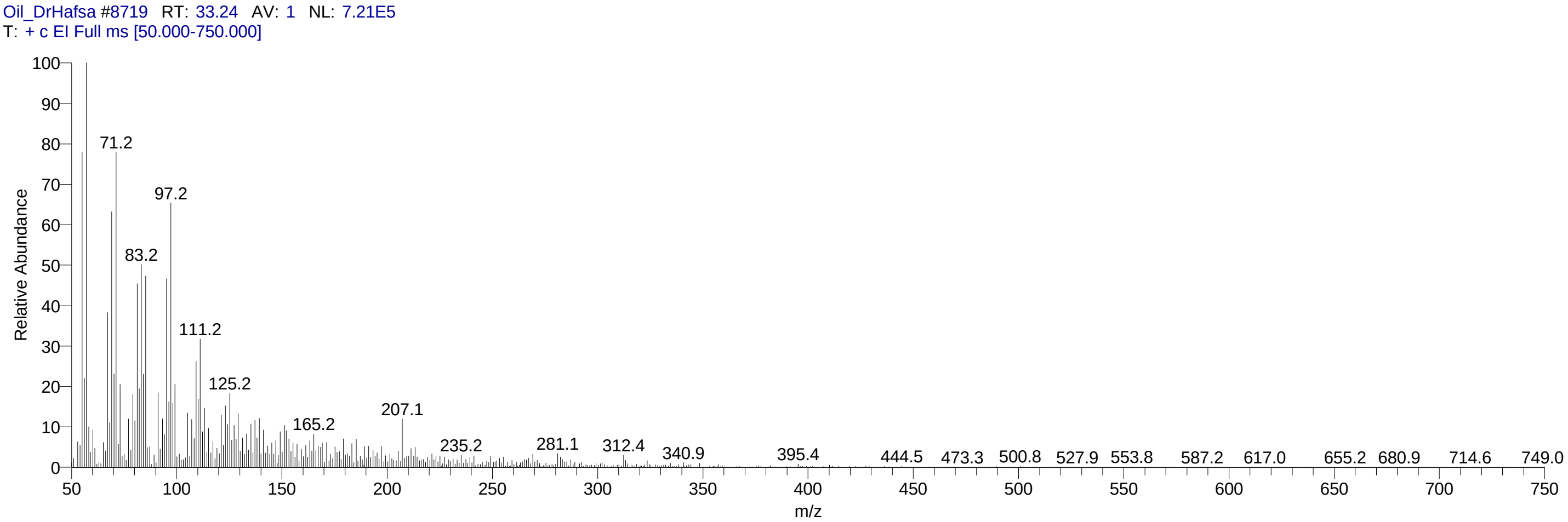

RT	Compound Name	Area %	MF	Molecular Formula	Molecular Weight	Cas #	Library
33.24	HAHNFETT	0.48	773	N/A	0	NA	WileyRegistry8e
33.24	HAHNFETT	0.48	773	N/A	0	NA	WileyRegistry8e
33.24	Z-5-Methyl-6-heneicosen-11-one	0.48	777	C22H42O	322	NA	mainlib
33.24	(22-Z)-DEHYDROCHOLESTEROL-1-ETHER	0.48	747	C28H46O	398	NA	WileyRegistry8e
33.24	14-á-H-PREGNA	0.48	778	C21H36	288	NA	WileyRegistry8e

Compound Structure	Hit Spectrum

HAHNFETT
Formula , MW 0, CAS# NA, Entry# 305496

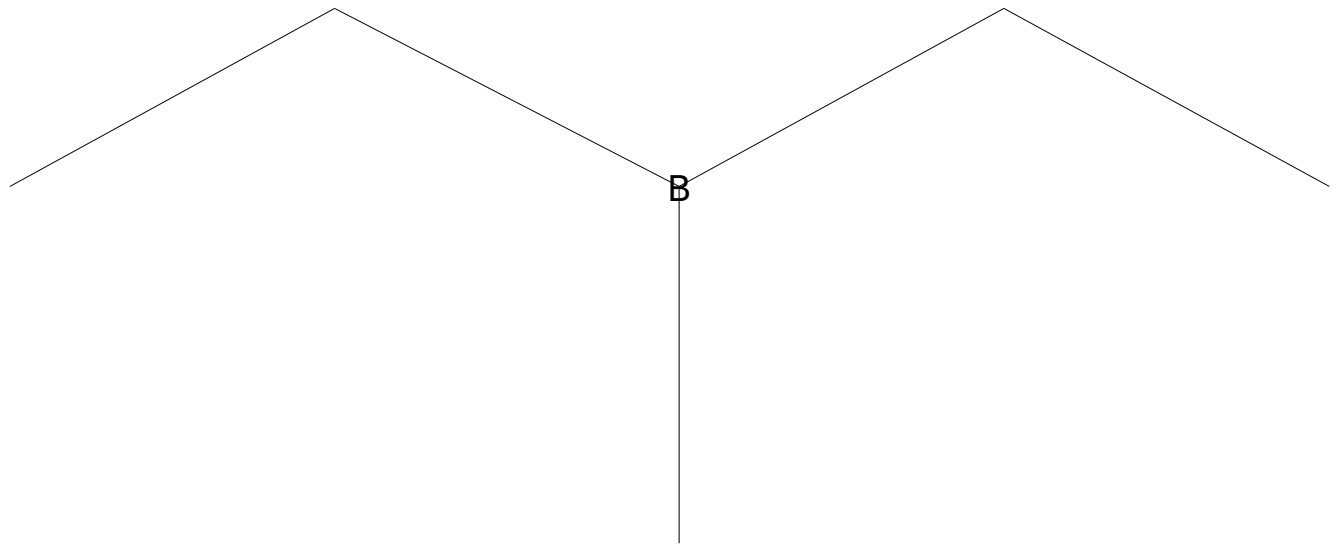

SI 762, RSI 773, WileyRegistry8e, Entry# 305496, CAS# NA, HAHNFETT

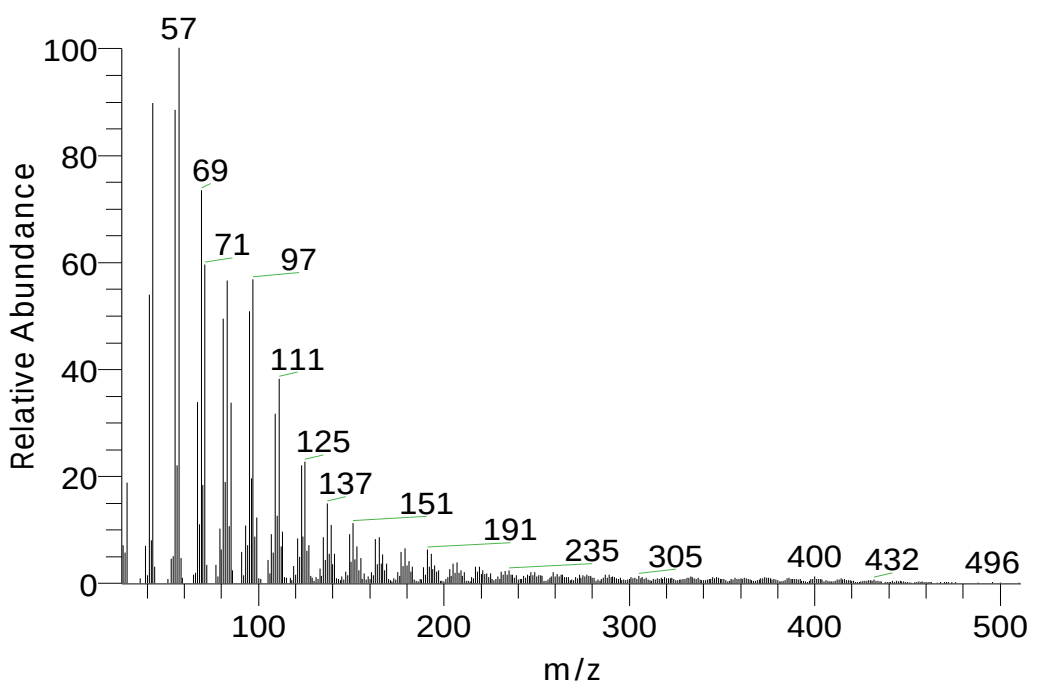

HAHNFETT
Formula , MW 0, CAS# NA, Entry# 391160

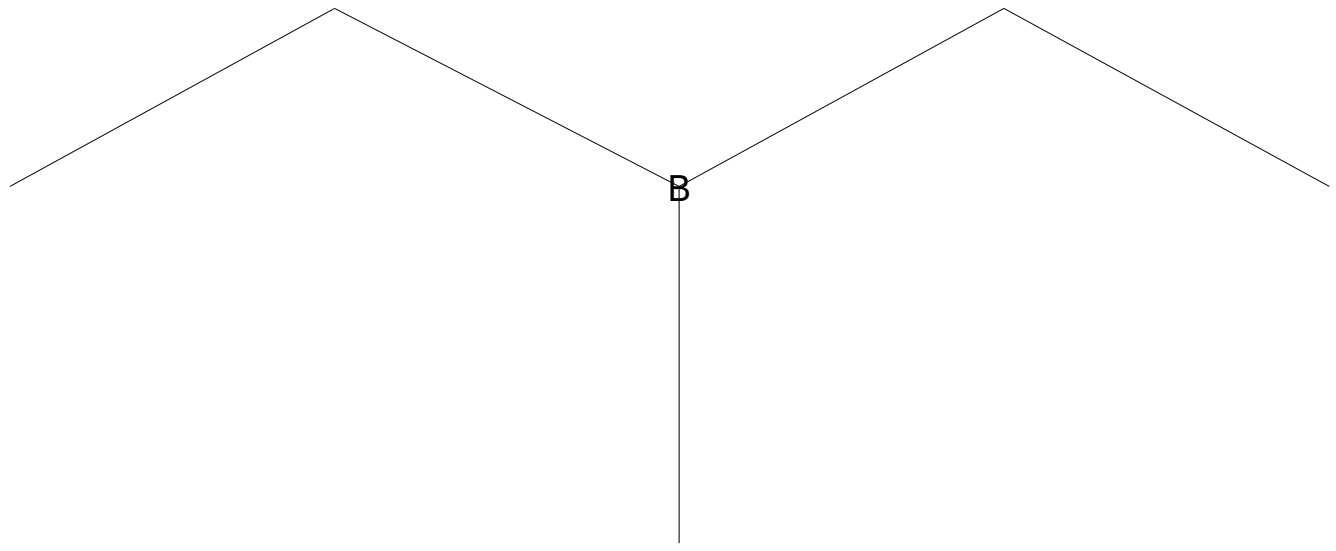

SI 762, RSI 773, WileyRegistry8e, Entry# 391160, CAS# NA, HAHNFETT

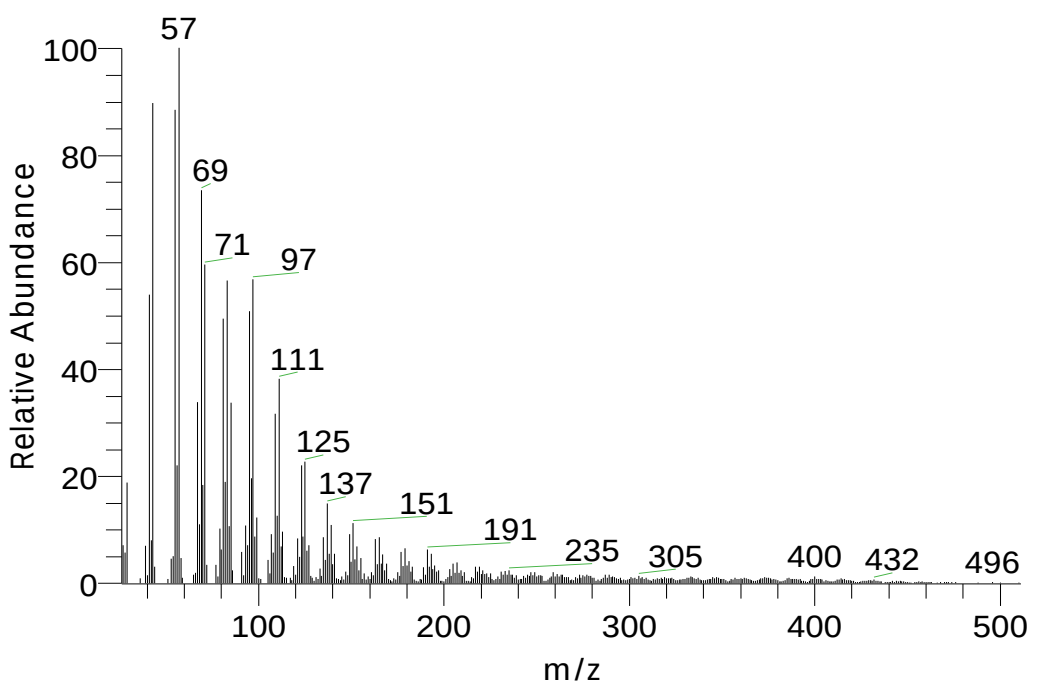

My GC-MS Report

Compound Structure | Hit Spectrum

Z-5-Methyl-6-heneicosen-11-one
Formula C22H42O, MW 322, CAS# NA, Entry# 7198
(6Z)-5-Methyl-6-henicosen-11-one #

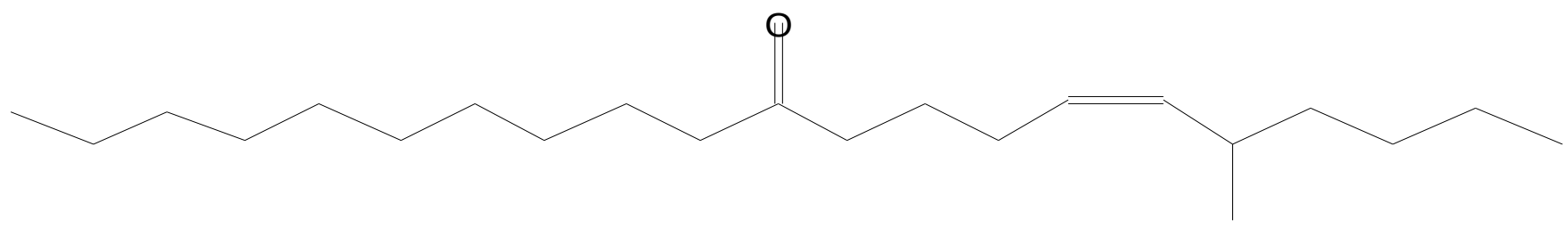

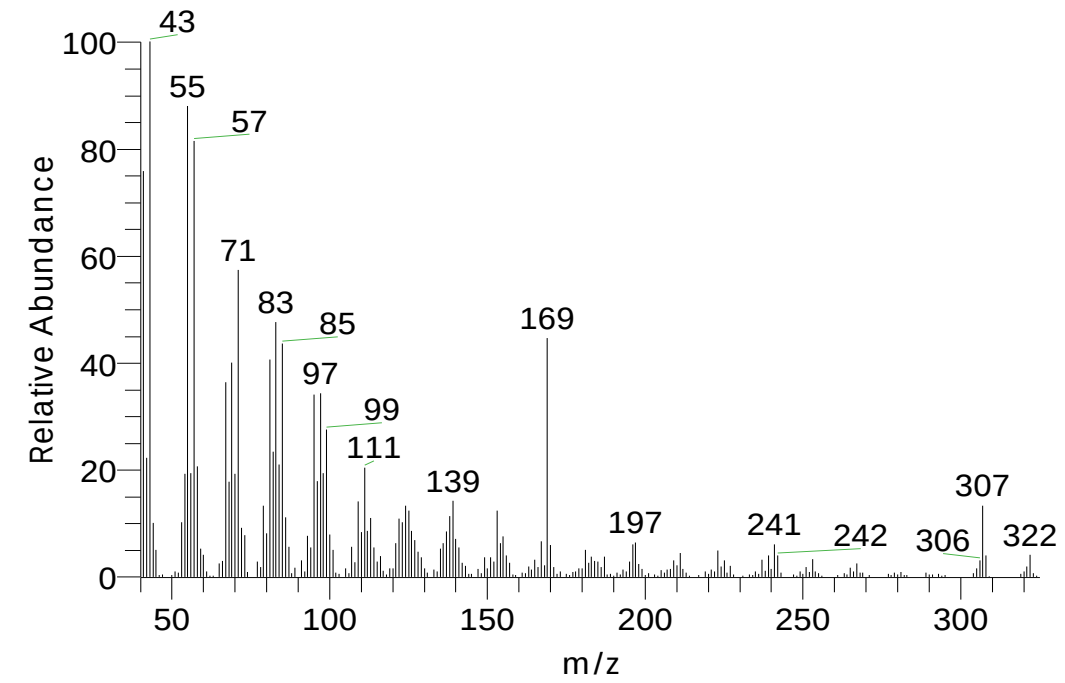

(22-Z)-DEHYDROCHOLESTEROL-1-ETHER
Formula C28H46O, MW 398, CAS# NA, Entry# 255363

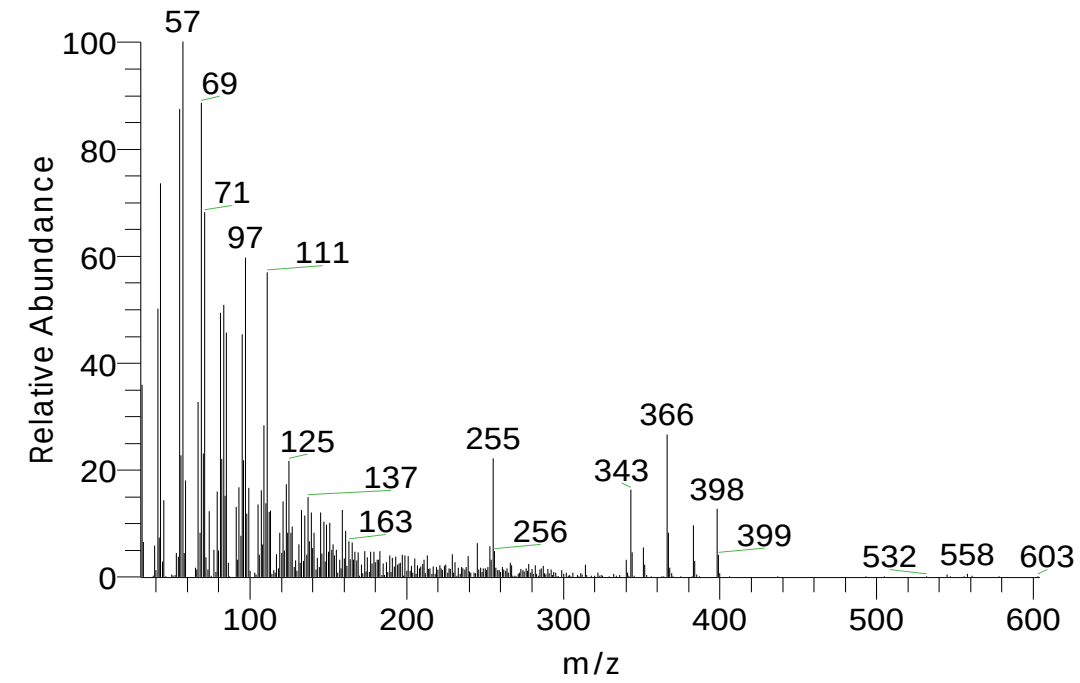

14-á-H-PREGNA
Formula C21H36, MW 288, CAS# NA, Entry# 178938
14-á-PREGNA

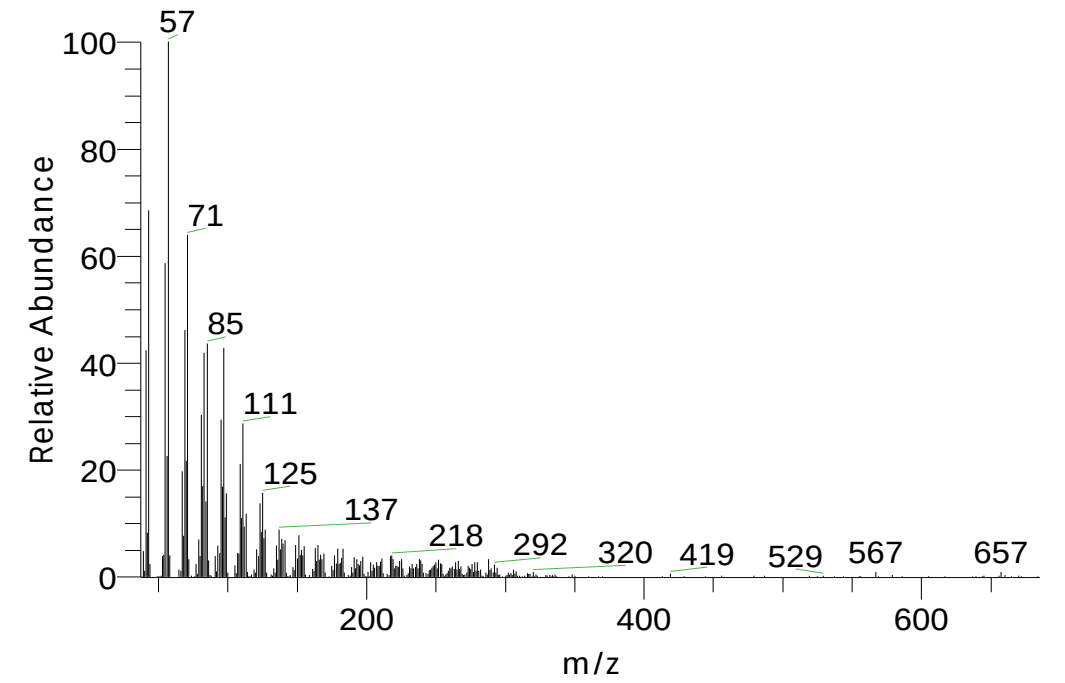

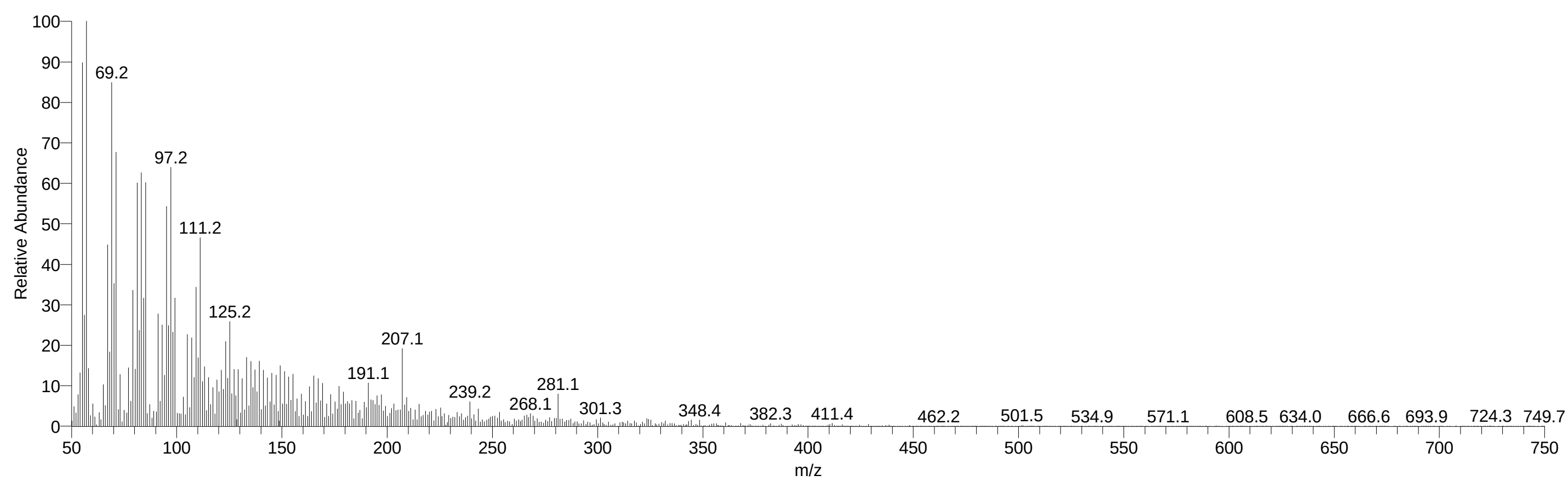

RT	Compound Name	Area %	MF	Molecular Formula	Molecular Weight	Cas #	Library
33.53	HAHNFETT	0.19	791	N/A	0	NA	WileyRegistry8e
33.53	HAHNFETT	0.19	791	N/A	0	NA	WileyRegistry8e

My GC-MS Report

RT	Compound Name	Area %	MF	Molecular Formula	Molecular Weight	Cas #	Library
33.53	2-ACETYL-3-(2-CINNAMIDO)ETHYL-7-METHOXYINDOLE	0.19	807	C22H22N2O3	362	NA	WileyRegistry8e
33.53	14-á-H-PREGNA	0.19	777	C21H36	288	NA	WileyRegistry8e
33.53	Oleic acid, 3-(octadecyloxy)propyl ester	0.19	761	C39H76O3	592	17367-41-8	mainlib

Compound Structure | Hit Spectrum

HAHNFETT
Formula , MW 0, CAS# NA, Entry# 305496

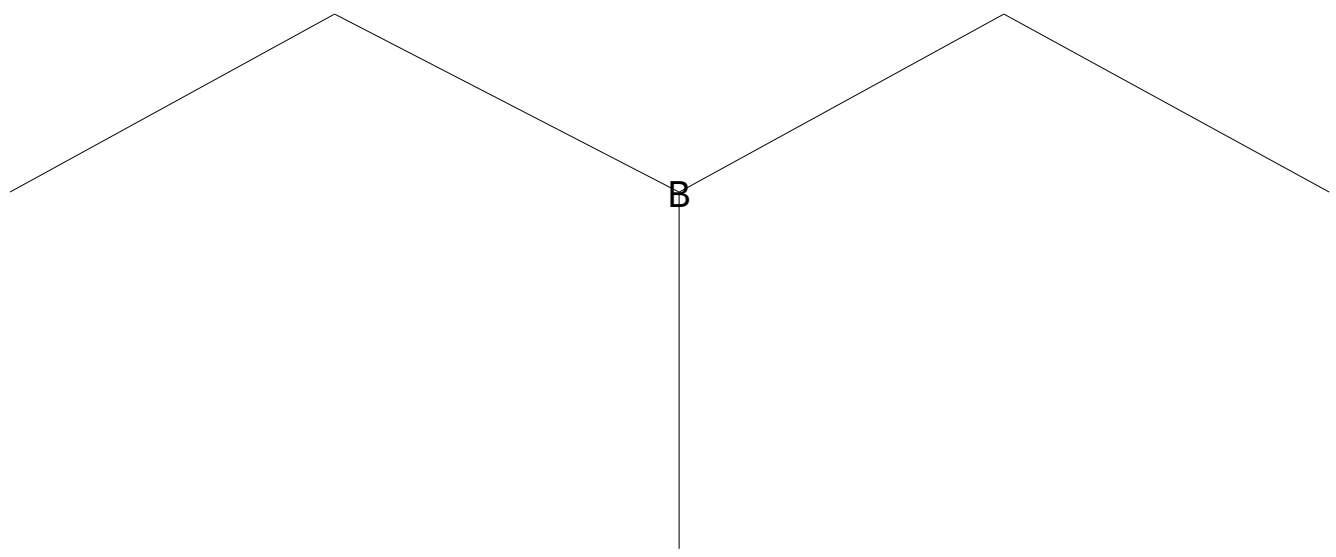

SI 782, RSI 791, WileyRegistry8e, Entry# 305496, CAS# NA, HAHNFETT

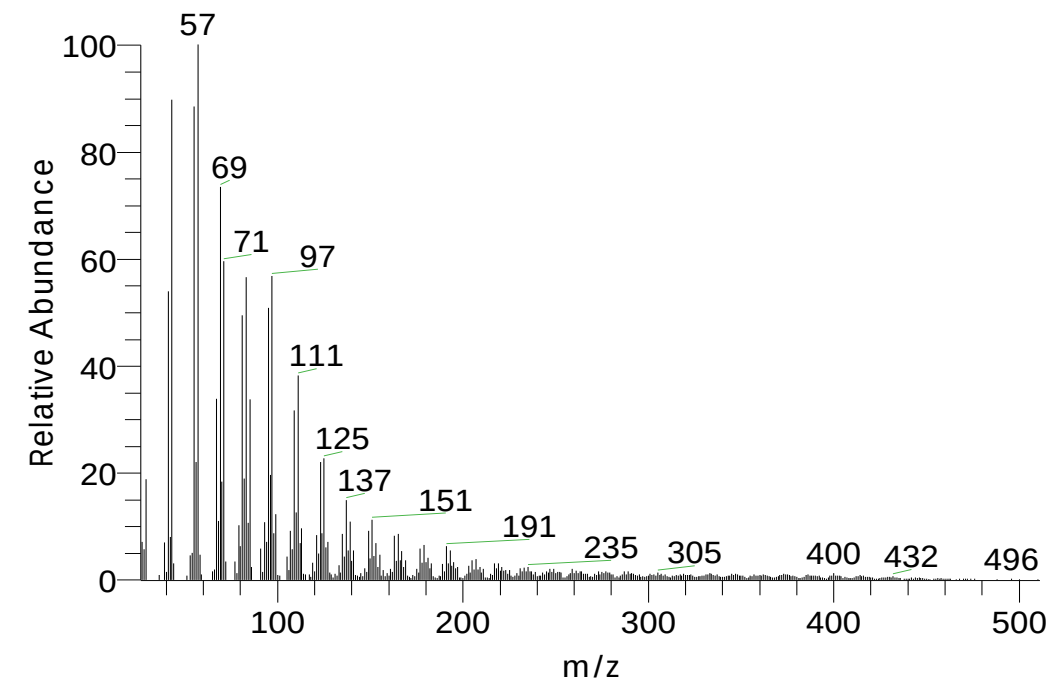

HAHNFETT
Formula , MW 0, CAS# NA, Entry# 391160

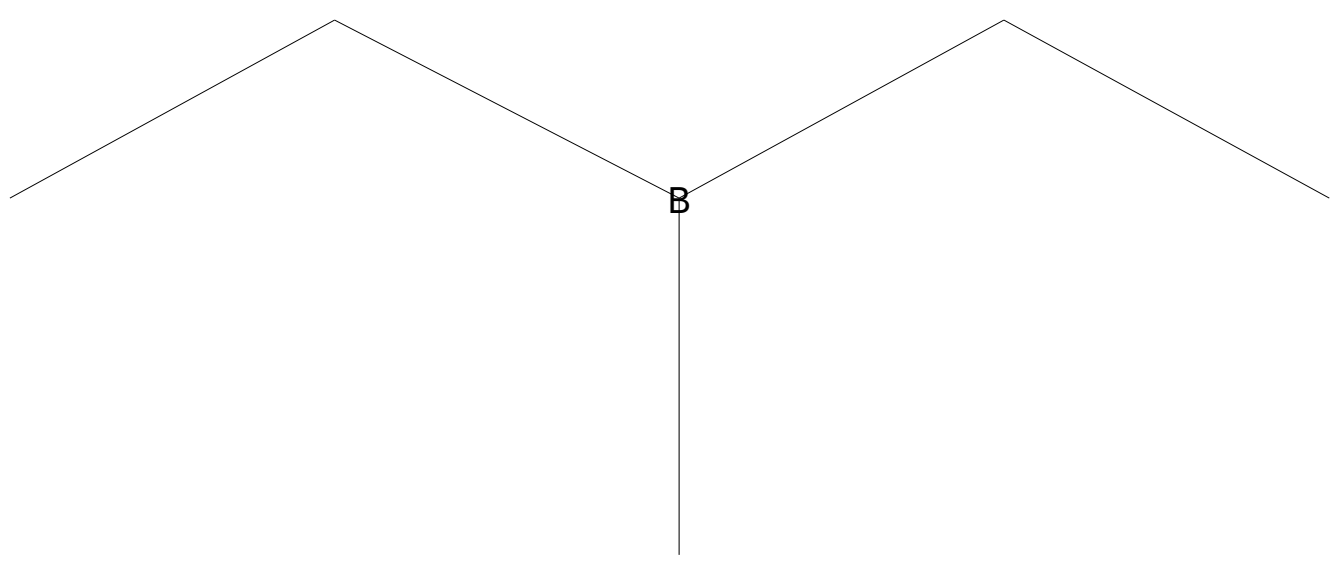

SI 782, RSI 791, WileyRegistry8e, Entry# 391160, CAS# NA, HAHNFETT

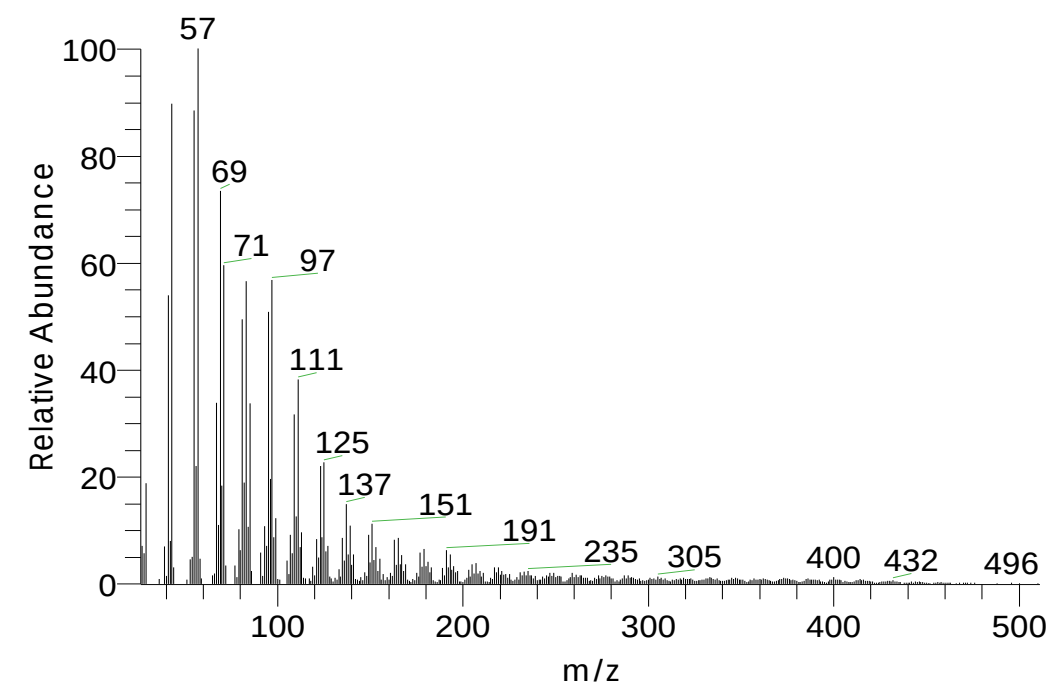

2-ACETYL-3-(2-CINNAMIDO)ETHYL-7-METHOXYINDOLE
Formula C22H22N2O3, MW 362, CAS# NA, Entry# 236392

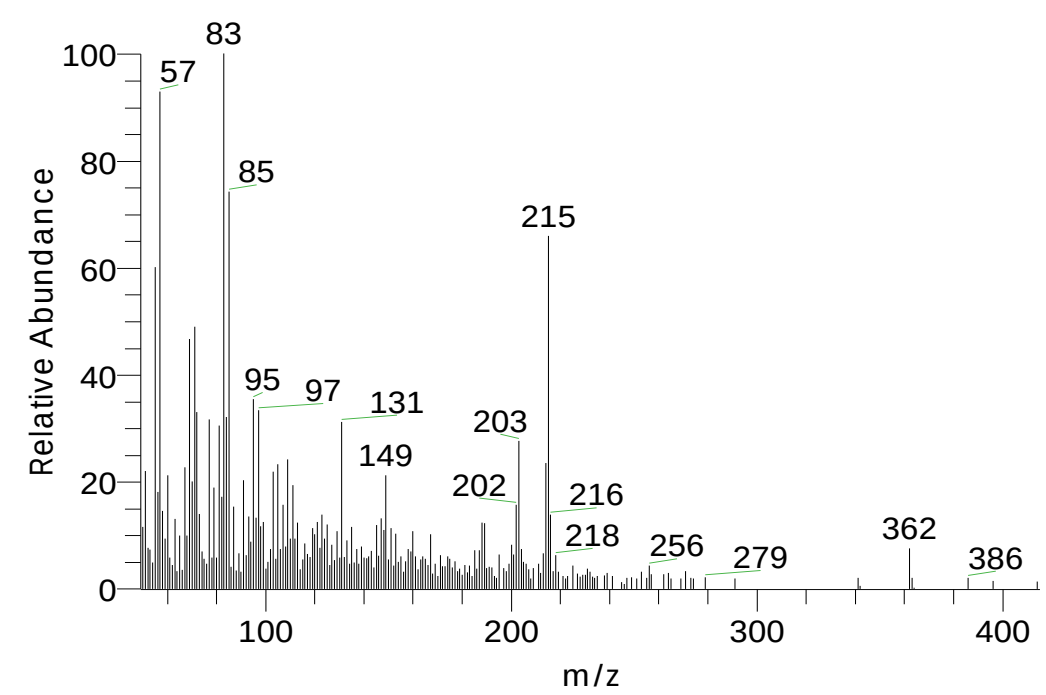

14-á-H-PREGNA
Formula C21H36, MW 288, CAS# NA, Entry# 178938
14-á-PREGNA

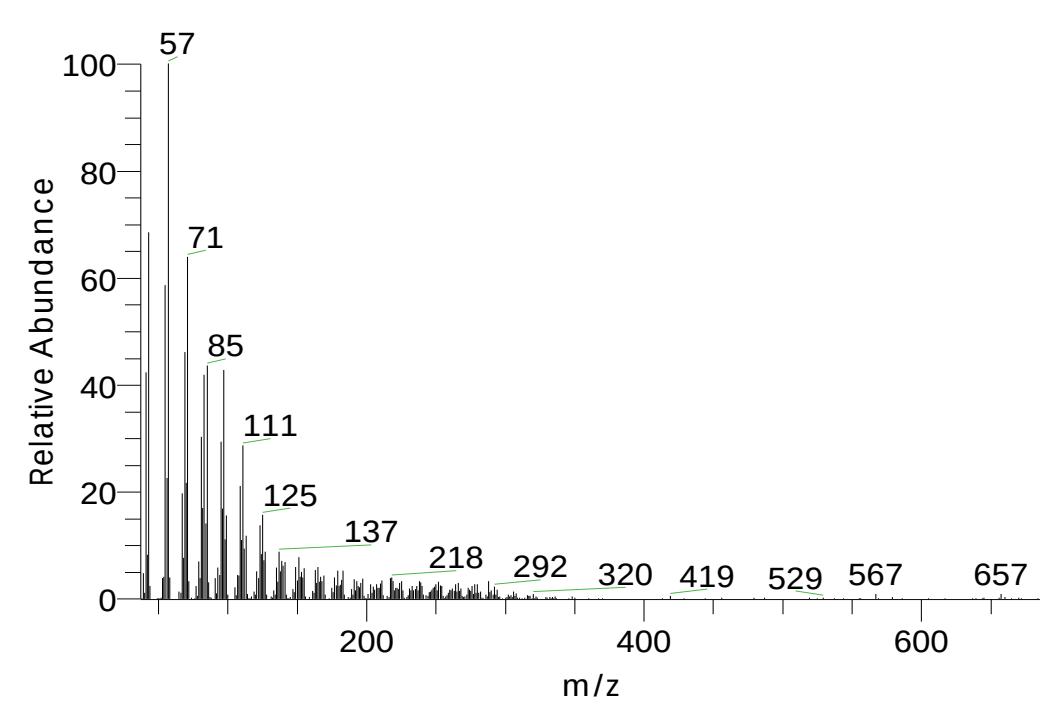

My GC-MS Report

Compound Structure | Hit Spectrum

Oleic acid, 3-(octadecyloxy)propyl ester
Formula C39H76O3, MW 592, CAS# 17367-41-8, Entry# 24115
3-(Octadecyloxy)propyl (9E)-9-octadecenoate #

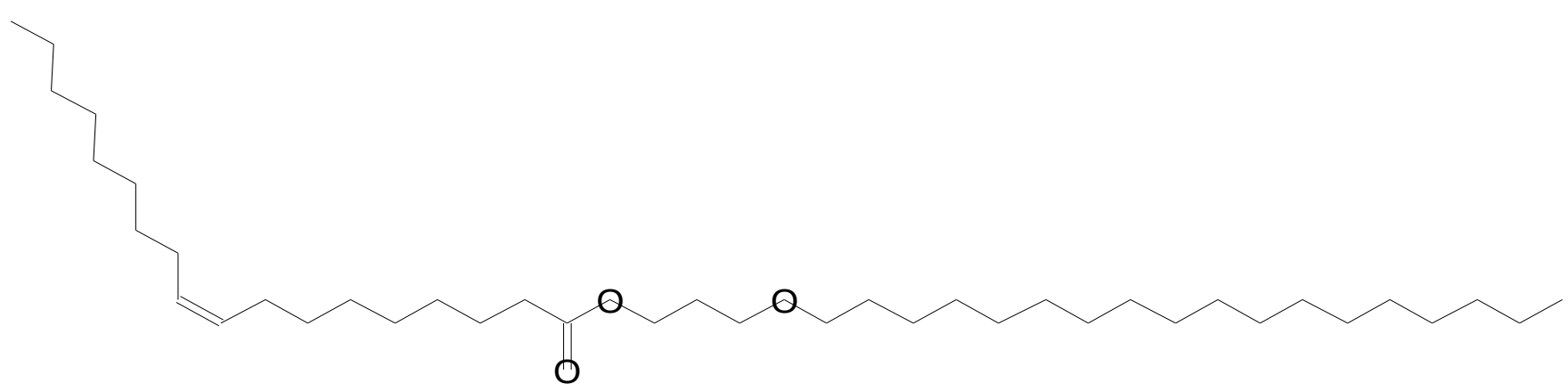

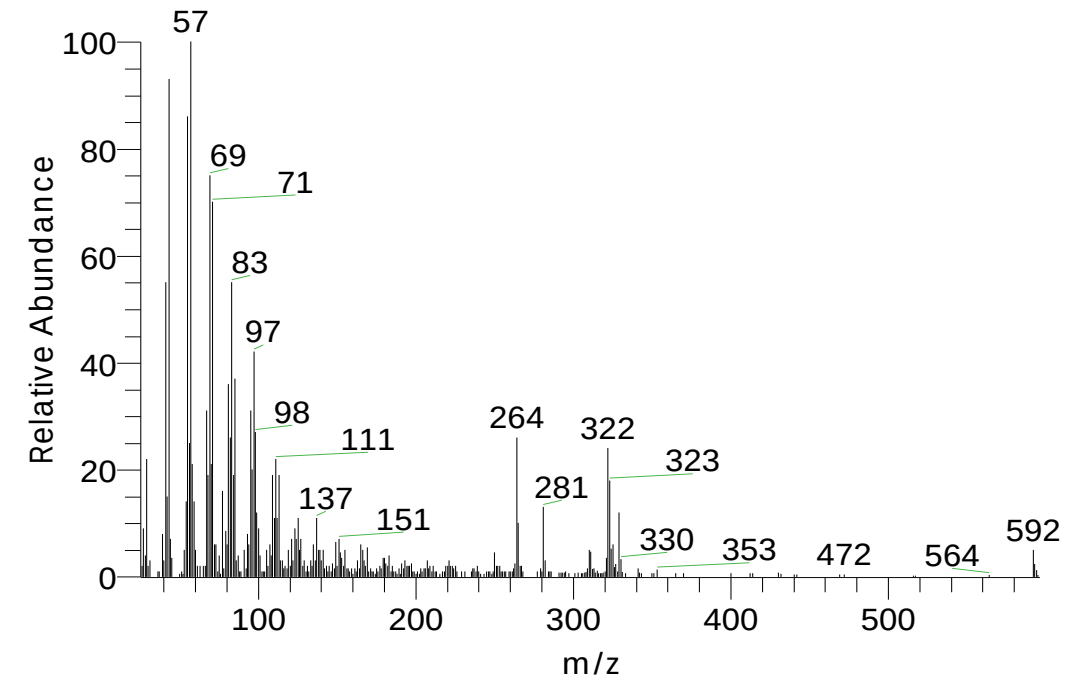

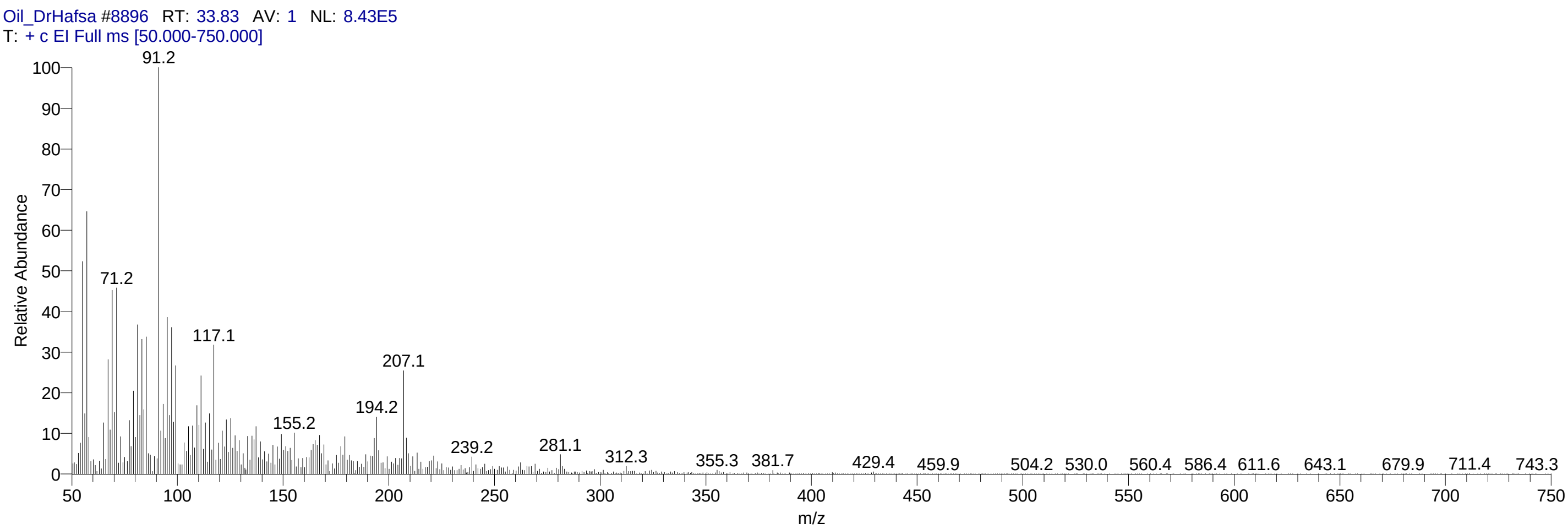

RT	Compound Name	Area %	MF	Molecular Formula	Molecular Weight	Cas #	Library
33.83	ISOCHIAPIN B	1.04	794	C19H22O6	346	NA	WileyRegistry8e
33.83	ISOCHIAPIN B %2<	1.04	794	C19H26O6	350	NA	WileyRegistry8e
33.83	1-Heptatriacotanol	1.04	790	C37H76O	536	105794-58-9	mainlib
33.83	4H-1-BENZOPYRAN-4-ONE, 2-(3,4-DIMETHOXYPHENYL)-3,5-DIHYDROXY-7-METHOXY-	1.04	758	C18H16O7	344	6068-80-0	WileyRegistry8e
33.83	DOTRIACONTANE	1.04	780	C32H66	450	544-85-4	WileyRegistry8e

Compound Structure | Hit Spectrum

ISOCHIAPIN B
Formula C19H22O6, MW 346, CAS# NA, Entry# 225807

SI 787, RSI 794, WileyRegistry8e, Entry# 225807, CAS# NA, ISOCHIAPIN B

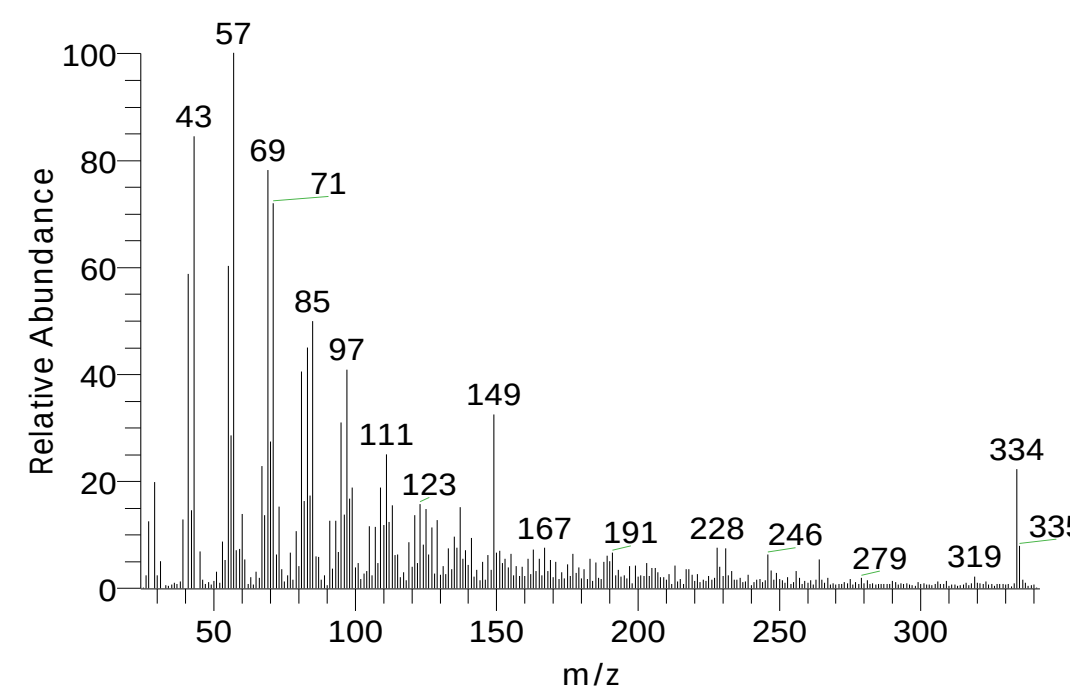

My GC-MS Report

Compound Structure | Hit Spectrum

ISOCHIAPIN B %2<
Formula C19H26O6, MW 350, CAS# NA, Entry# 228500

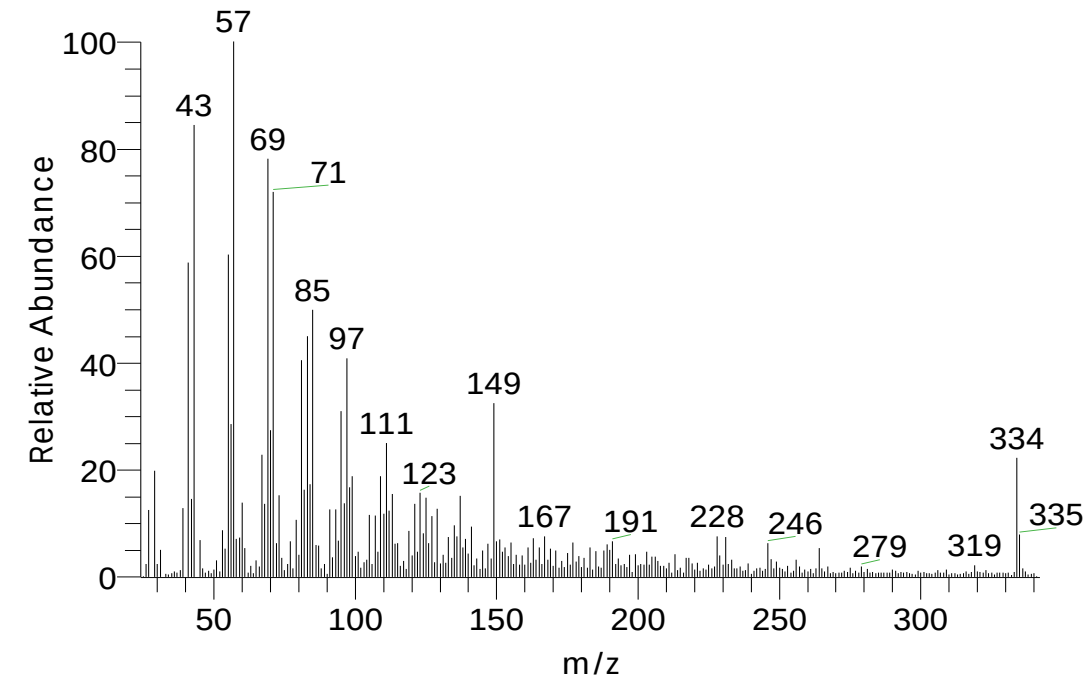

1-Heptatriacotanol
Formula C37H76O, MW 536, CAS# 105794-58-9, Entry# 7279
1-Heptatriacontanol #

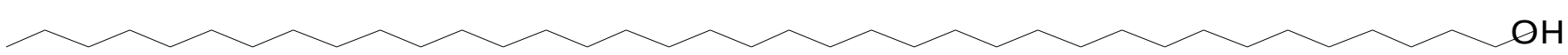

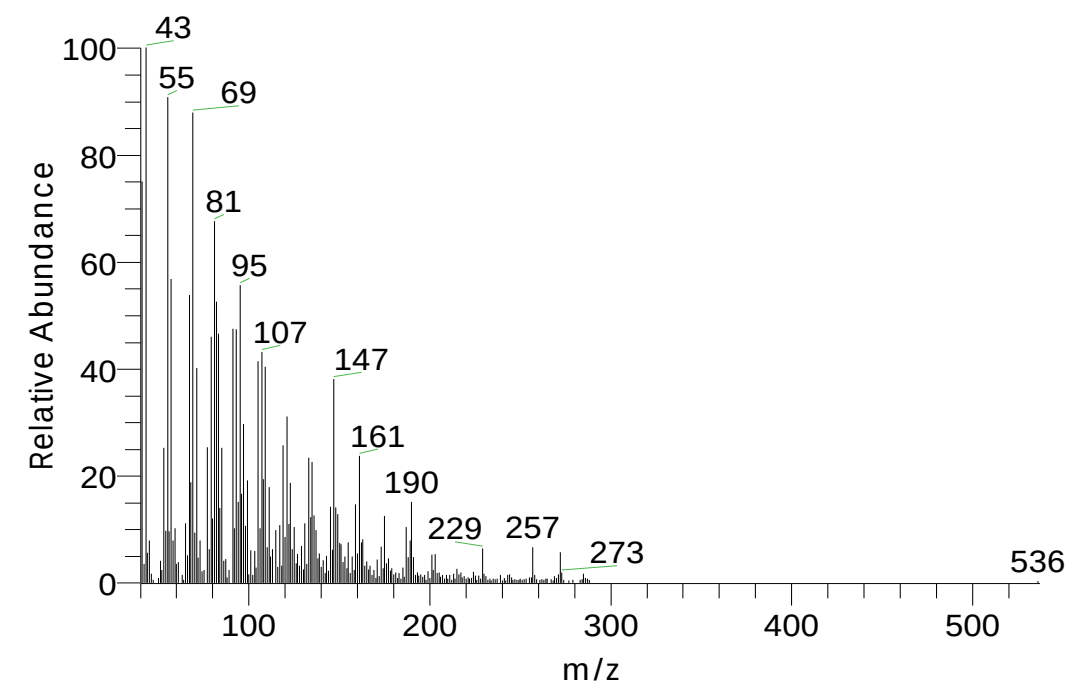

4H-1-BENZOPYRAN-4-ONE, 2-(3,4-DIMETHOXYPHENYL)-3,5-DIHYDROXY-7-METHOXY-
Formula C18H16O7, MW 344, CAS# 6068-80-0, Entry# 224392
3',4',7-TRIMETHYLQUERCETIN

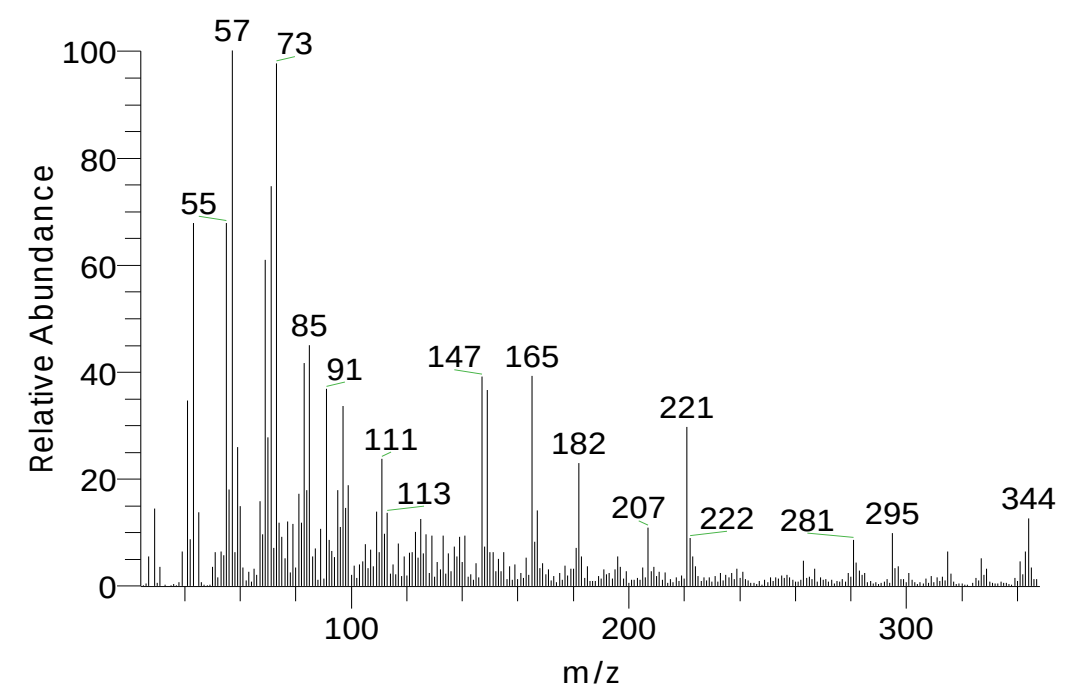

DOTRIACONTANE
Formula C32H66, MW 450, CAS# 544-85-4, Entry# 274478
AI3-52367

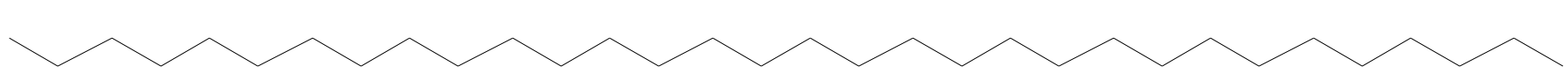

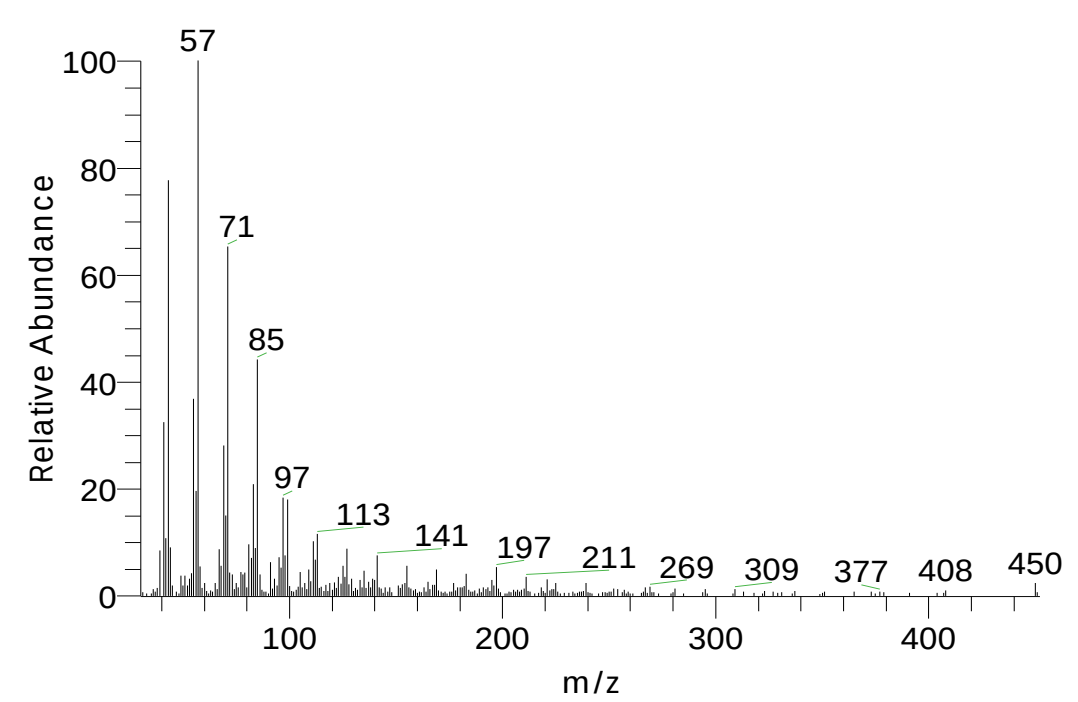

My GC-MS Report

Oil_DrHafsa #8935 RT: 33.96 AV: 1 NL: 4.95E5
T: + c EI Full ms [50.000-750.000]

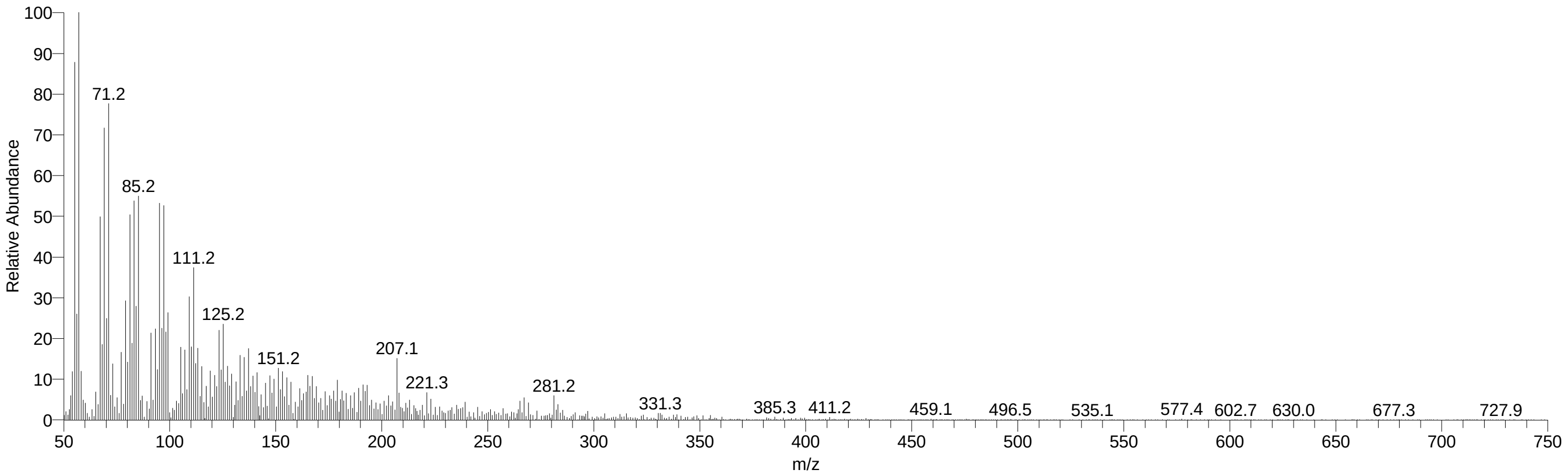

RT	Compound Name	Area %	MF	Molecular Formula	Molecular Weight	Cas #	Library
33.96	ISOCHIAPIN B	0.13	808	C19H22O6	346	NA	WileyRegistry8e
33.96	ISOCHIAPIN B %2<	0.13	808	C19H26O6	350	NA	WileyRegistry8e
33.96	HAHNFETT	0.13	783	N/A	0	NA	WileyRegistry8e
33.96	HAHNFETT	0.13	782	N/A	0	NA	WileyRegistry8e
33.96	4H-1-BENZOPYRAN-4-ONE, 2-(3,4-DIMETHOXYPHENYL)-3,5-DIHYDROXY-7-METHOXY-	0.13	776	C18H16O7	344	6068-80-0	WileyRegistry8e

Compound Structure

Hit Spectrum

ISOCHIAPIN B
Formula C19H22O6, MW 346, CAS# NA, Entry# 225807

SI 799, RSI 808, WileyRegistry8e, Entry# 225807, CAS# NA, ISOCHIAPIN B

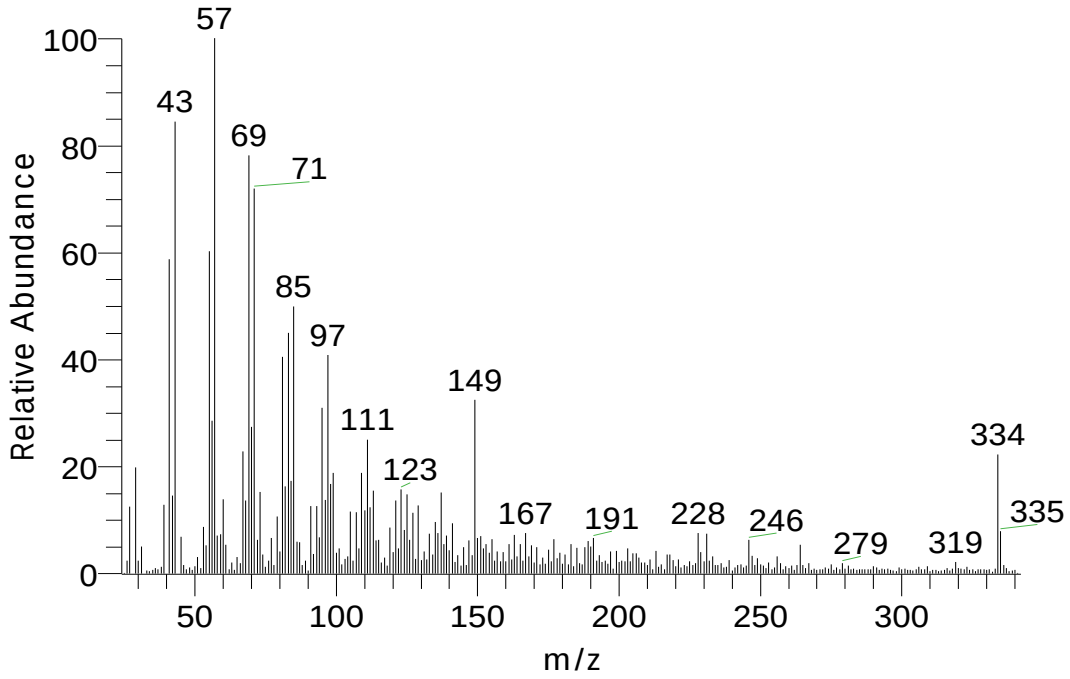

ISOCHIAPIN B %2<
Formula C19H26O6, MW 350, CAS# NA, Entry# 228500

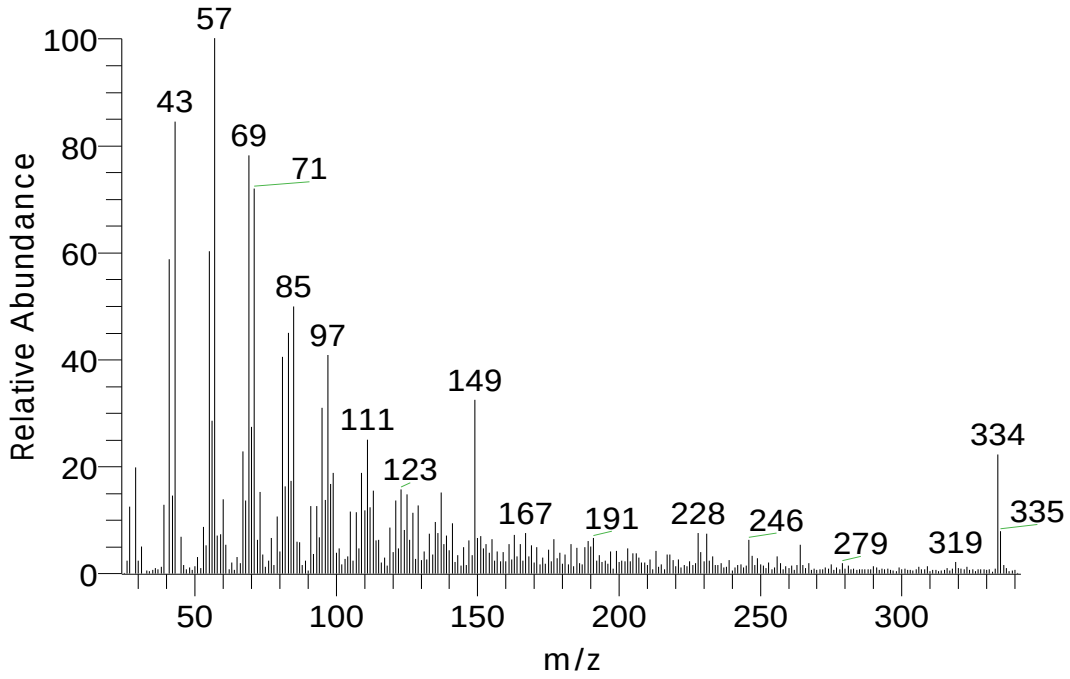

My GC-MS Report

Compound Structure | Hit Spectrum

HAHNFETT
Formula , MW 0, CAS# NA, Entry# 391160

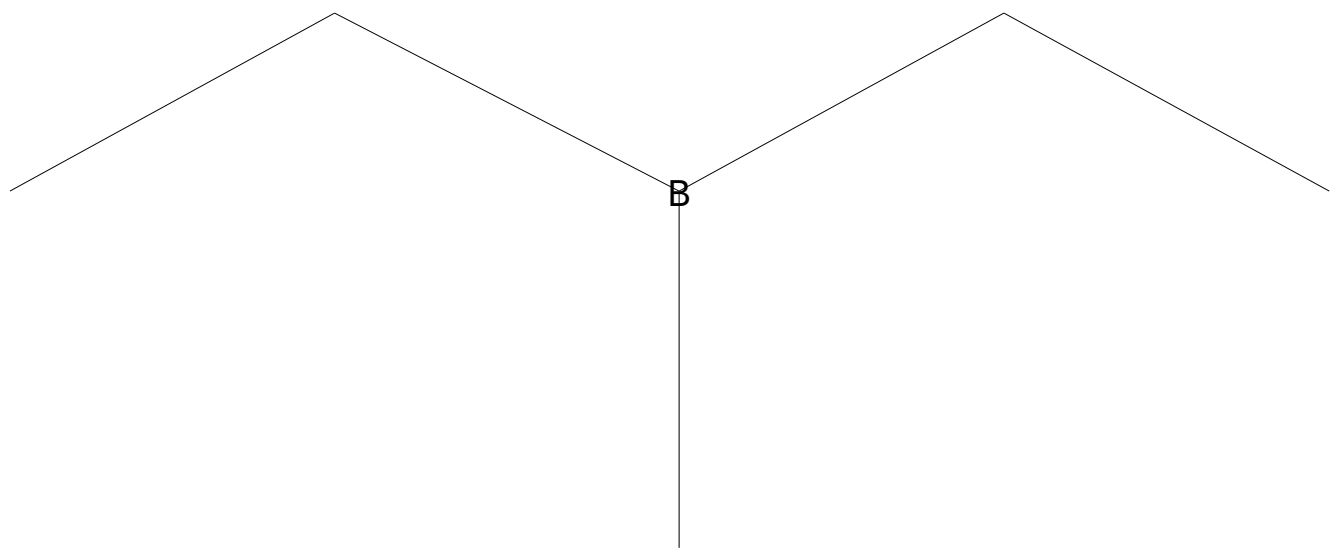

SI 774, RSI 783, WileyRegistry8e, Entry# 391160, CAS# NA, HAHNFETT

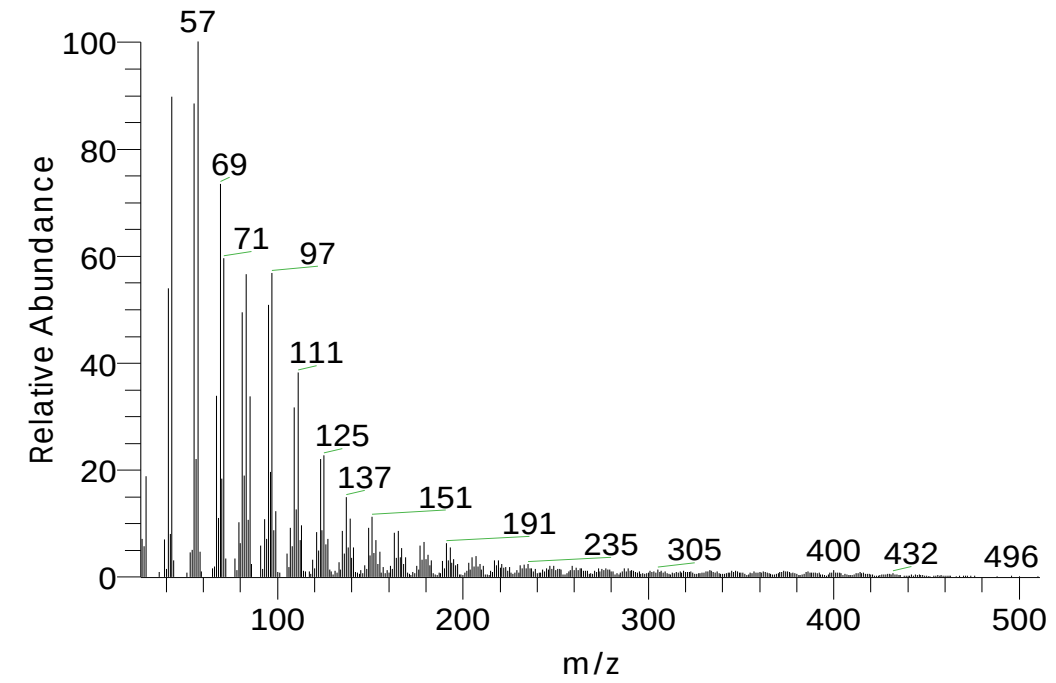

HAHNFETT
Formula , MW 0, CAS# NA, Entry# 305496

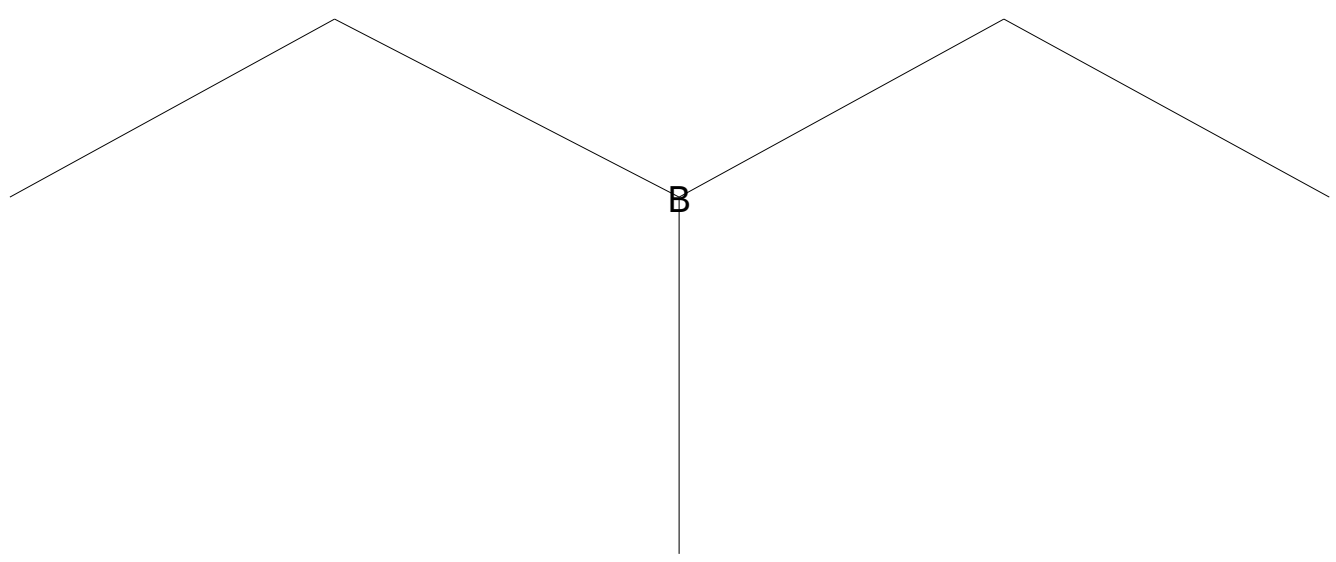

SI 774, RSI 782, WileyRegistry8e, Entry# 305496, CAS# NA, HAHNFETT

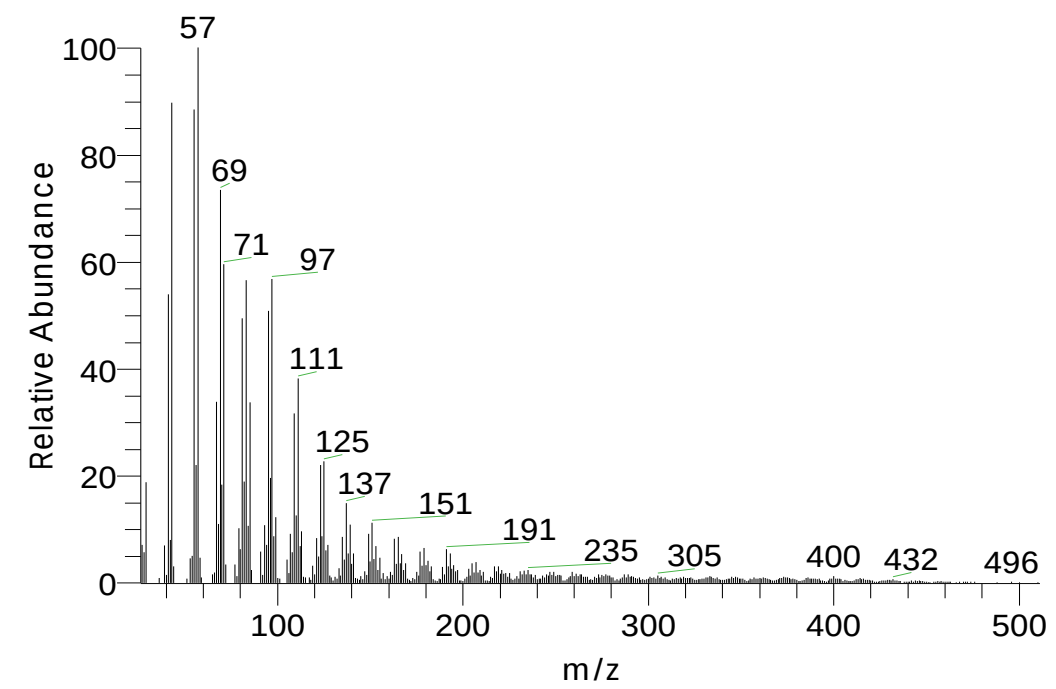

4H-1-BENZOPYRAN-4-ONE, 2-(3,4-DIMETHOXYPHENYL)-3,5-DIHYDROXY-7-METHOXY-
Formula C18H16O7, MW 344, CAS# 6068-80-0, Entry# 224392
3',4',7-TRIMETHYLQUERCETIN

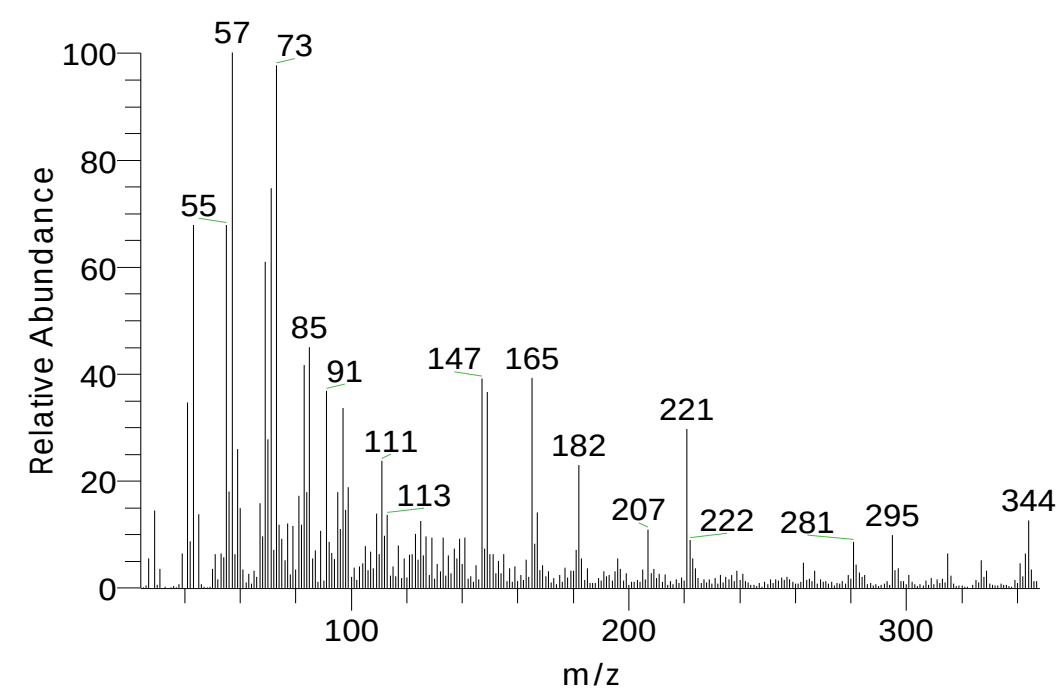

Oil_DrHafsa #9163 RT: 34.73 AV: 1 NL: 3.89E5
T: + c EI Full ms [50.000-750.000]

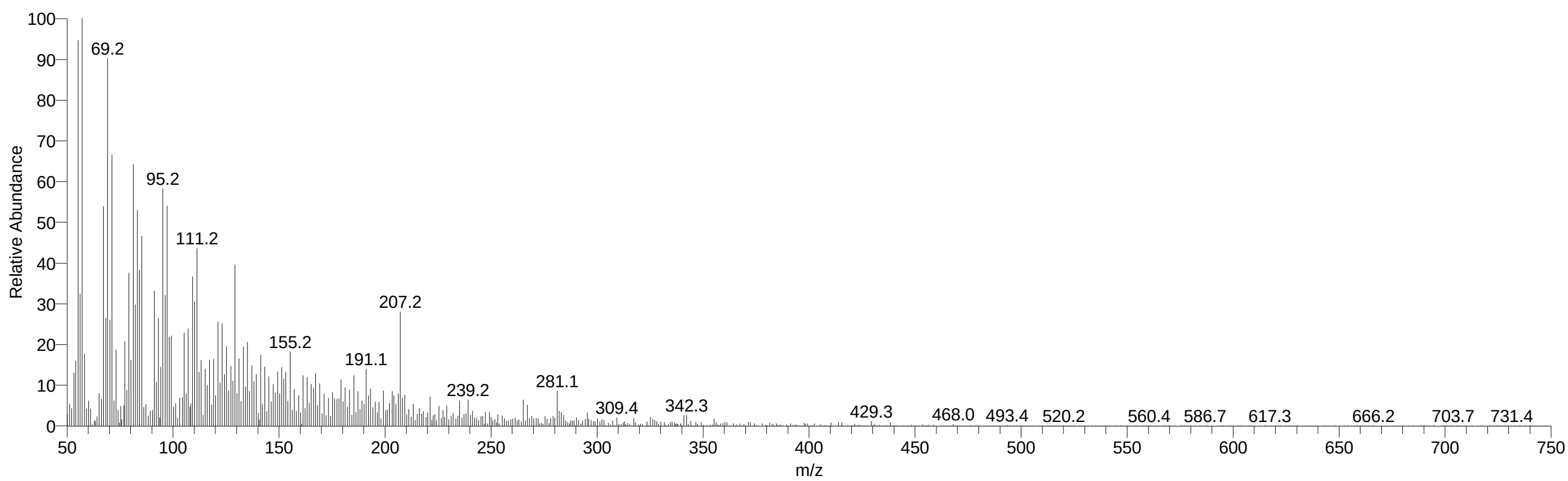

RT	Compound Name	Area %	MF	Molecular Formula	Molecular Weight	Cas #	Library
34.73	01297107001 TETRANEURIN - A - DIOL	0.32	814	C15H20O5	280	NA	WileyRegistry8e
34.73	1-Heptatriacotanol	0.32	813	C37H76O	536	105794-58-9	mainlib

My GC-MS Report

RT	Compound Name	Area %	MF	Molecular Formula	Molecular Weight	Cas #	Library
34.73	HAHNFETT	0.32	756	N/A	0	NA	WileyRegistry8e
34.73	HAHNFETT	0.32	756	N/A	0	NA	WileyRegistry8e
34.73	4H-1-BENZOPYRAN-4-ONE, 2-(3,4-DIHYDROXYPHENYL)-6,8-DI-á-D-GLUCOPYRANOSYL-5,7-DIHYDROXY-	0.32	741	C27H30O16	610	29428-58-8	WileyRegistry8e

Compound Structure | Hit Spectrum

01297107001 TETRANEURIN - A - DIOL
Formula C15H20O5, MW 280, CAS# NA, Entry# 170378

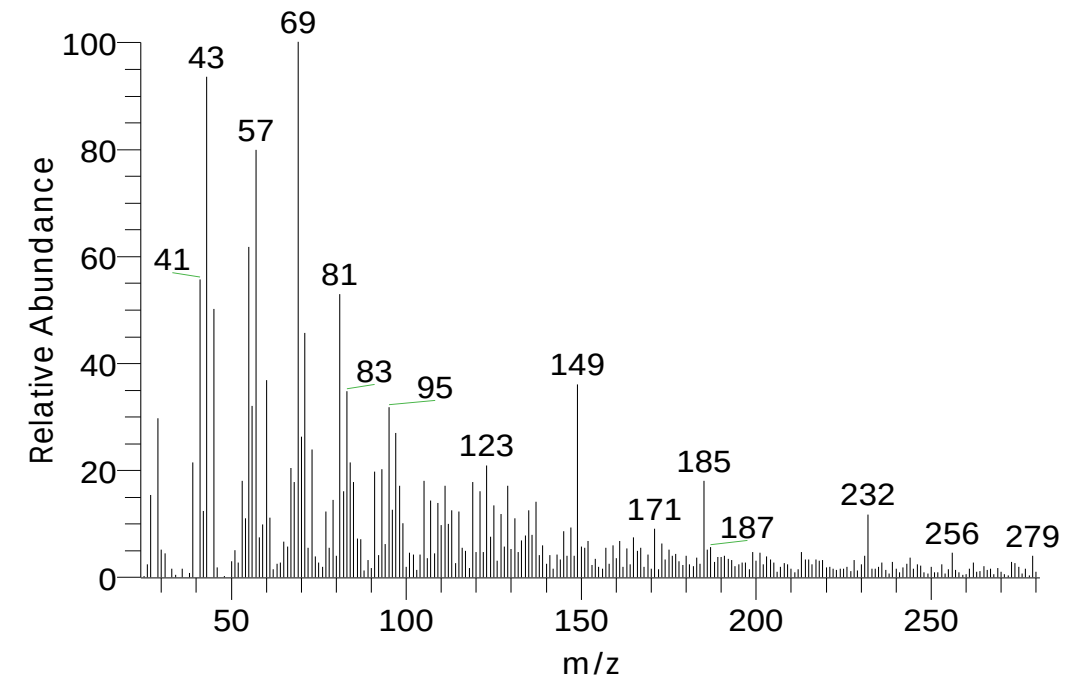

1-Heptatriacotanol
Formula C37H76O, MW 536, CAS# 105794-58-9, Entry# 7279
1-Heptatriacontanol #

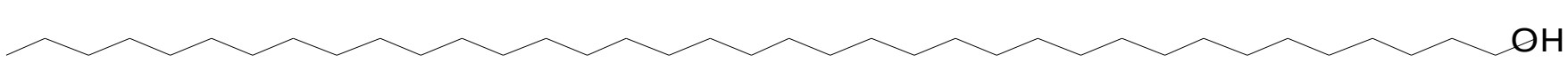

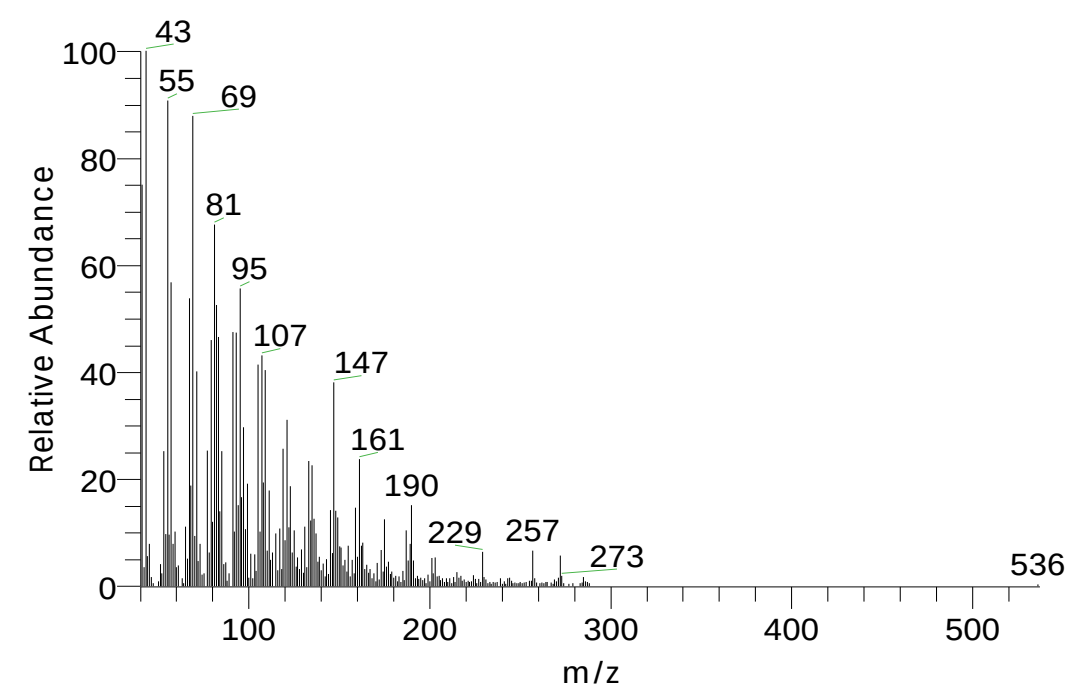

HAHNFETT
Formula , MW 0, CAS# NA, Entry# 305496

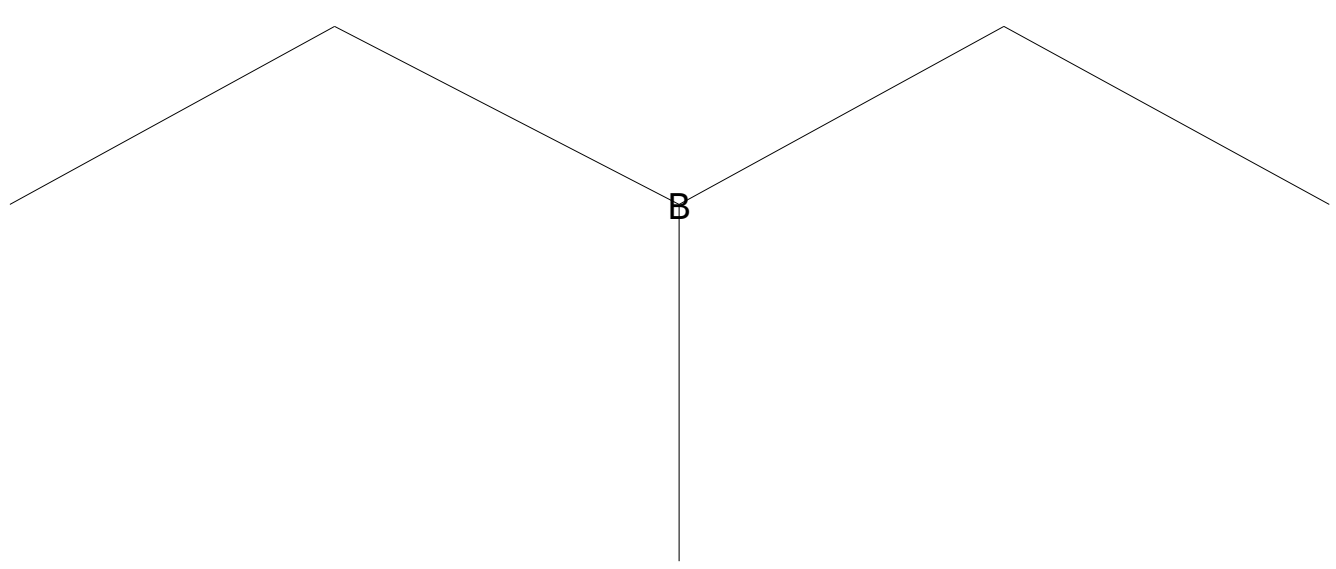

SI 746, RSI 756, WileyRegistry8e, Entry# 305496, CAS# NA, HAHNFETT

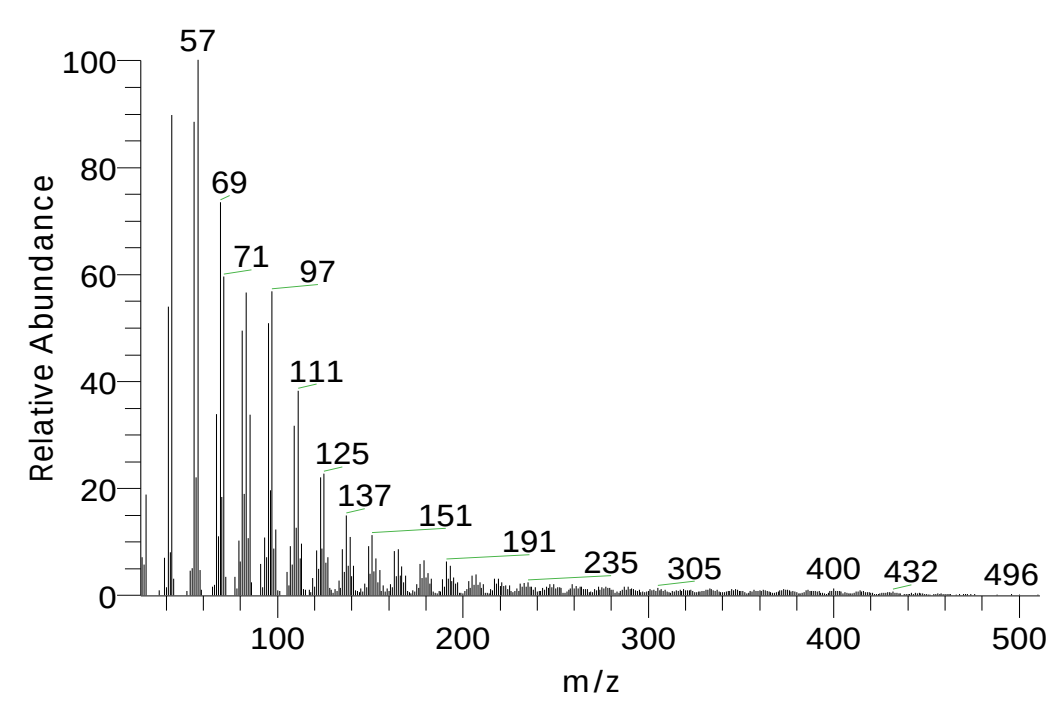

HAHNFETT
Formula , MW 0, CAS# NA, Entry# 391160

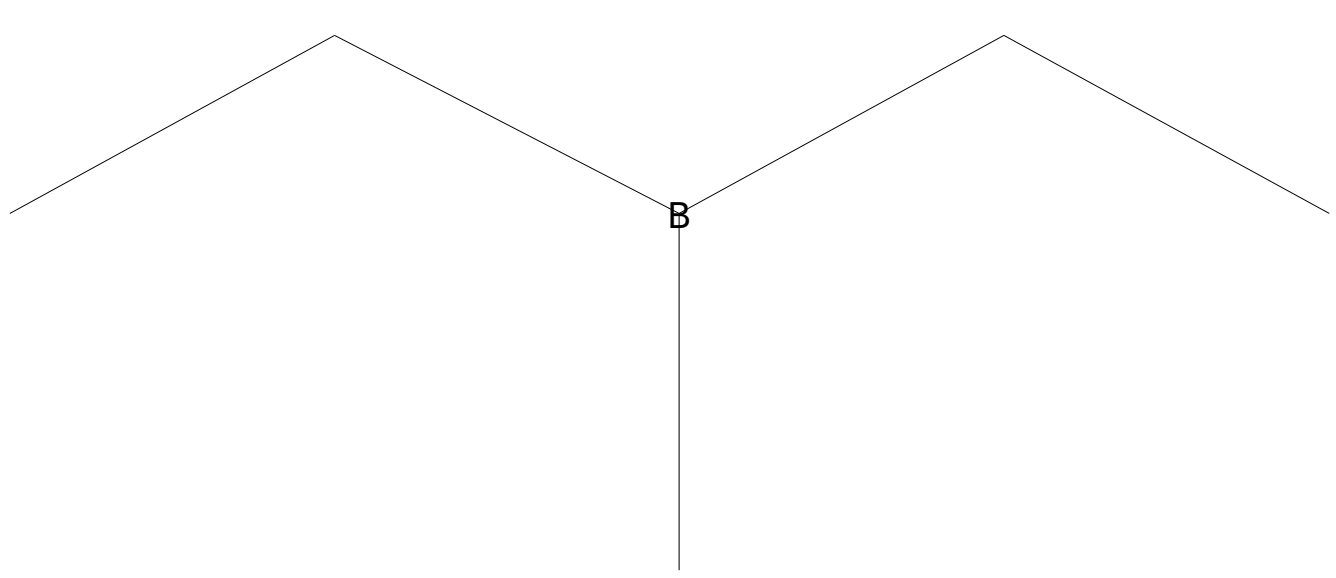

SI 746, RSI 756, WileyRegistry8e, Entry# 391160, CAS# NA, HAHNFETT

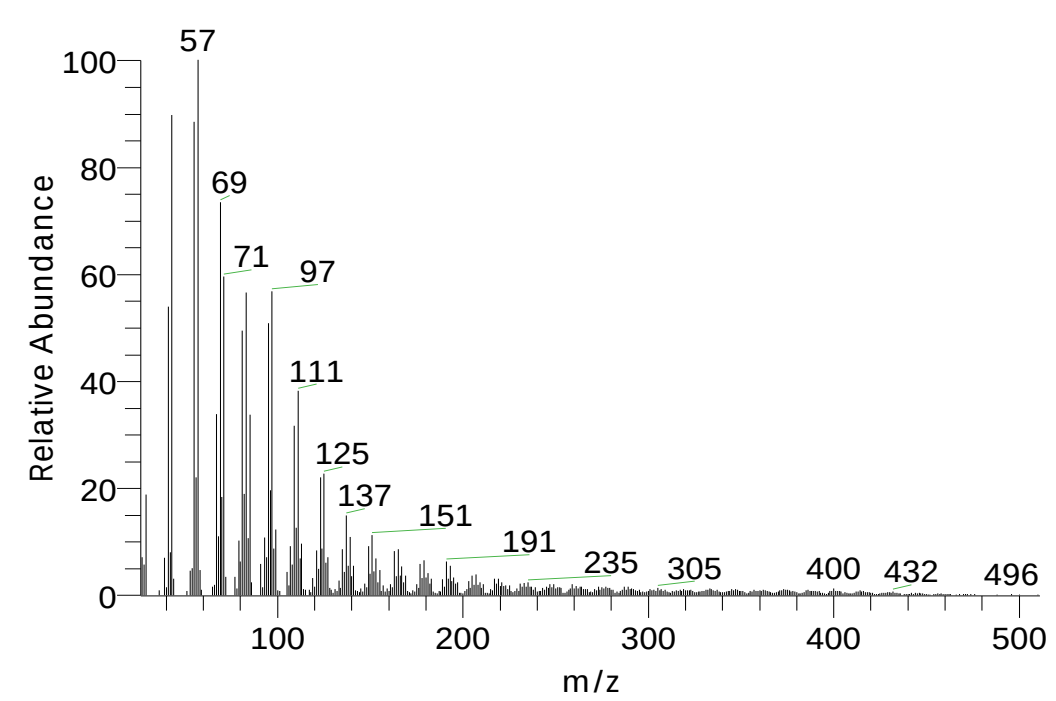

My GC-MS Report

Compound Structure | Hit Spectrum

Formula C27H30O16, MW 610, CAS# 29428-58-8, Entry# 297453
6,8-DI-C-á-GLUCOSYLLUTEOLIN

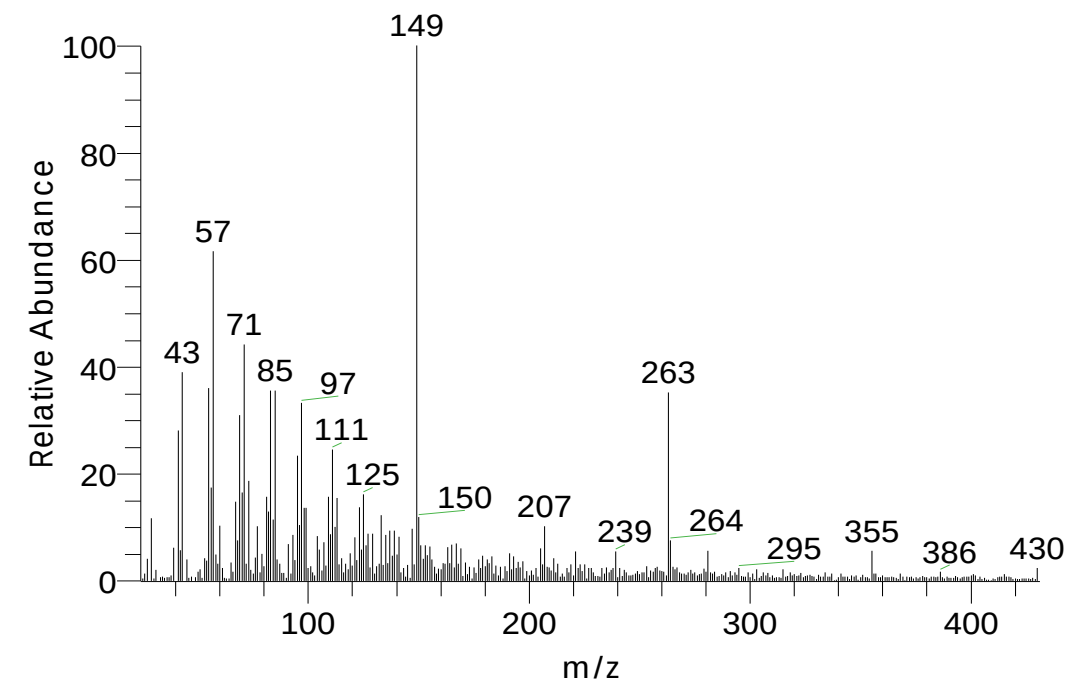

Oil_DrHafsa #9276 RT: 35.10 AV: 1 NL: 6.25E5
T: + c EI Full ms [50.000-750.000]

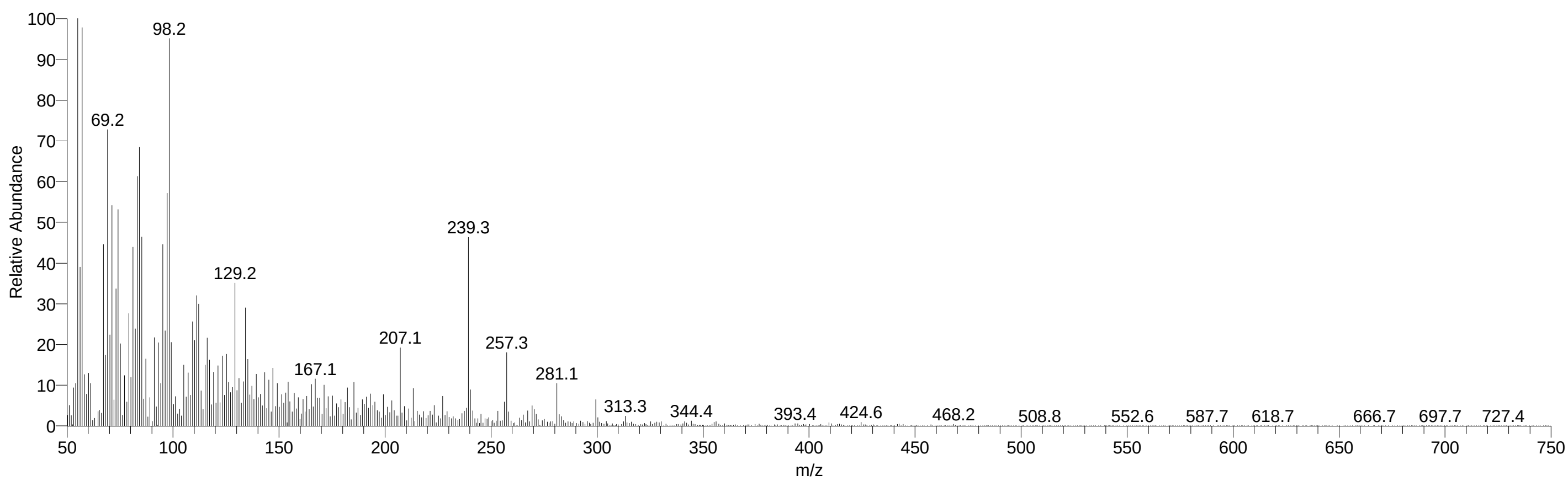

RT	Compound Name	Area %	MF	Molecular Formula	Molecular Weight	Cas #	Library
35.10	Hexadecanoic acid, 1-(hydroxymethyl)-1,2-ethanediyl ester	1.27	782	C35H68O5	568	761-35-3	mainlib
35.10	HEXADECANOIC ACID, 1-(HYDROXYMETHYL)-1,2-ETHANEDIYL ESTER	1.27	782	C35H68O5	568	761-35-3	WileyRegistry8e
35.10	HEXADECANOIC ACID, 1-(HYDROXYMETHYL)-1,2-ETHANEDIYL ESTER	1.27	788	C35H68O5	568	761-35-3	WileyRegistry8e
35.10	HEXADECANOIC ACID, 2,3-DIHYDROXYPROPYL ESTER	1.27	780	C19H38O4	330	542-44-9	WileyRegistry8e
35.10	HEXADECANOIC ACID, 2-HYDROXY-1,3-PROPANEDIYL ESTER	1.27	775	C35H68O5	568	502-52-3	WileyRegistry8e

Compound Structure | Hit Spectrum

Hexadecanoic acid, 1-(hydroxymethyl)-1,2-ethanediyl ester
Formula C35H68O5, MW 568, CAS# 761-35-3, Entry# 7720
Palmitin, 1,2-di-

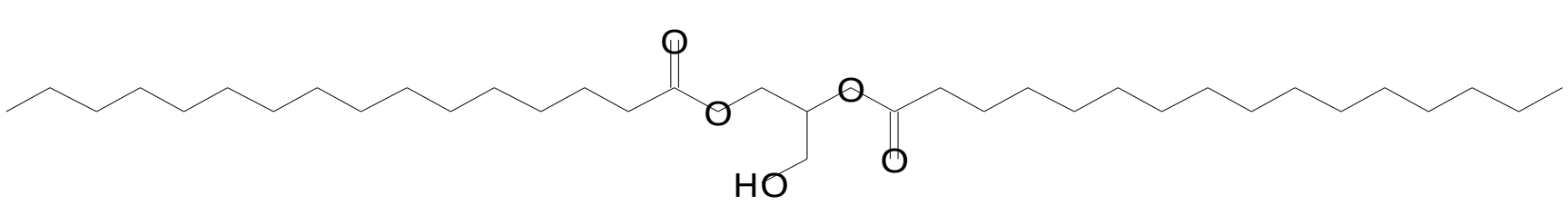

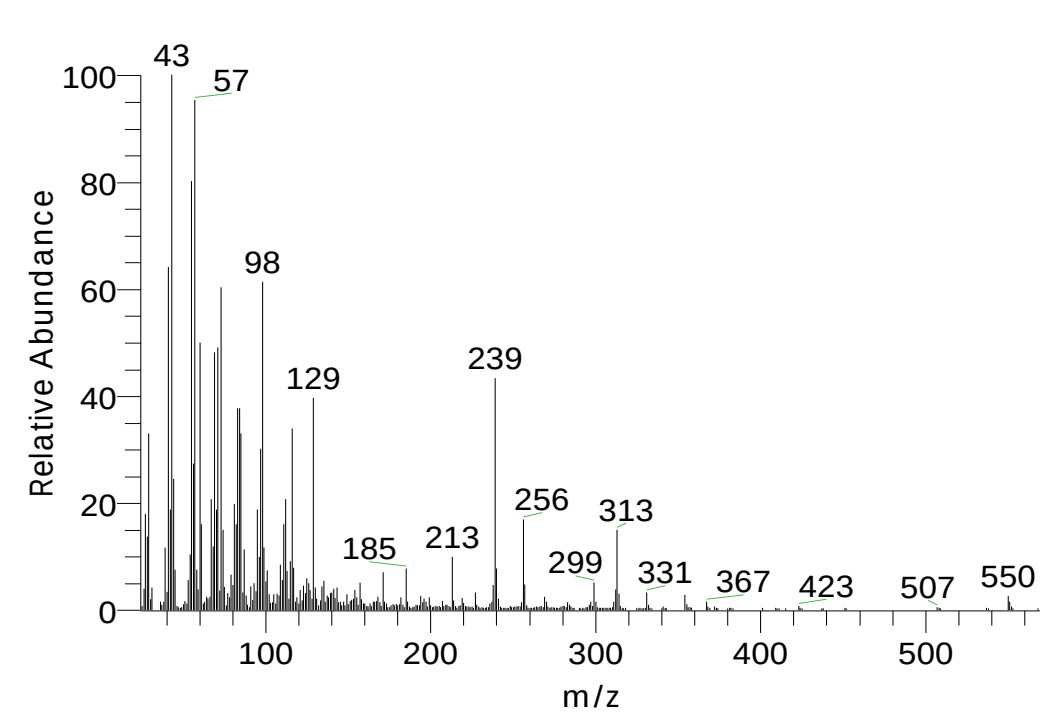

My GC-MS Report

Compound Structure | Hit Spectrum

HEXADECANOIC ACID, 1-(HYDROXYMETHYL)-1,2-ETHANEDIYL ESTER
Formula C35H68O5, MW 568, CAS# 761-35-3, Entry# 294145
2-HYDROXY-1-[(PALMITOYLOXY)METHYL]ETHYL PALMITATE #

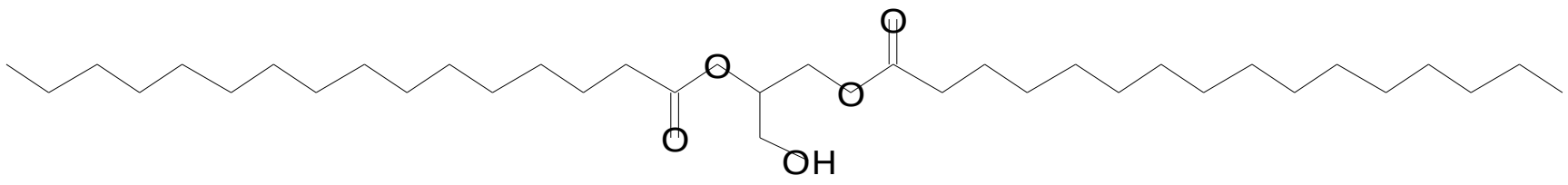

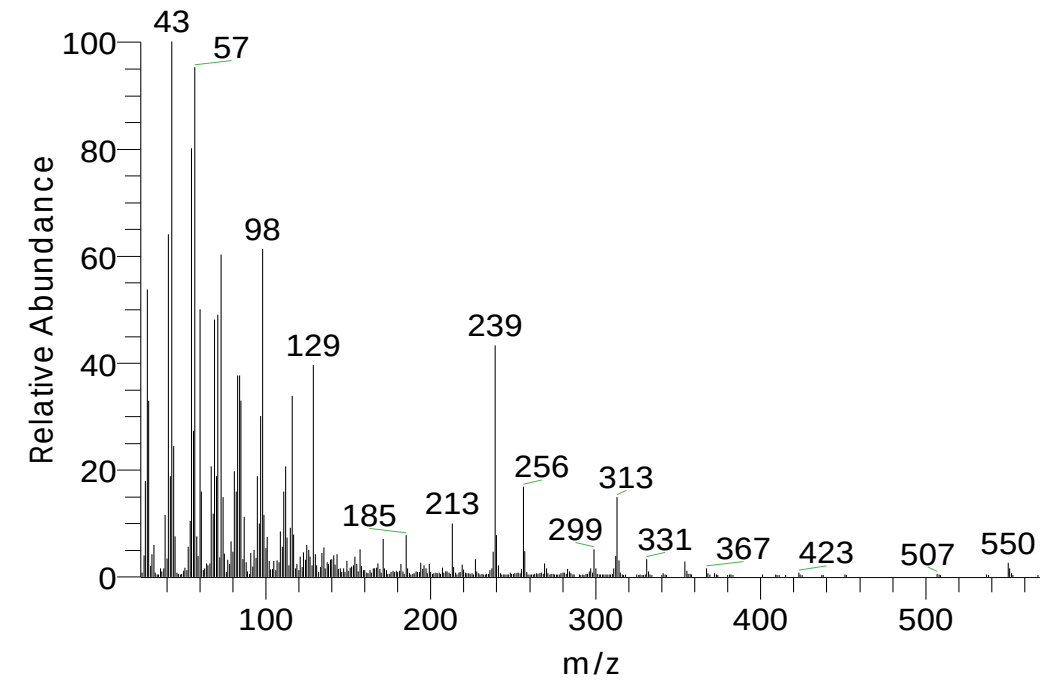

HEXADECANOIC ACID, 1-(HYDROXYMETHYL)-1,2-ETHANEDIYL ESTER
Formula C35H68O5, MW 568, CAS# 761-35-3, Entry# 294146
2-HYDROXY-1-[(PALMITOYLOXY)METHYL]ETHYL PALMITATE #

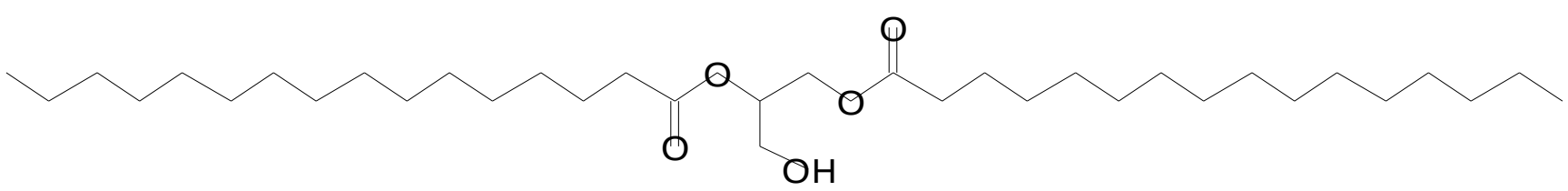

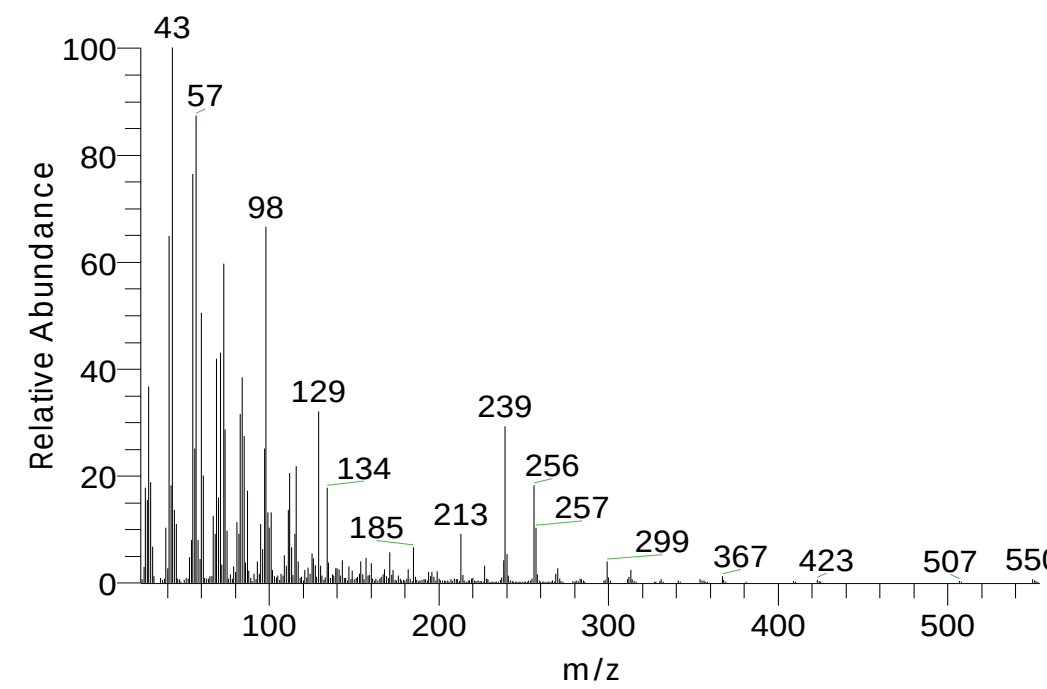

HEXADECANOIC ACID, 2,3-DIHYDROXYPROPYL ESTER
Formula C19H38O4, MW 330, CAS# 542-44-9, Entry# 214589
2,3-DIHYDROXYPROPYL PALMITATE #

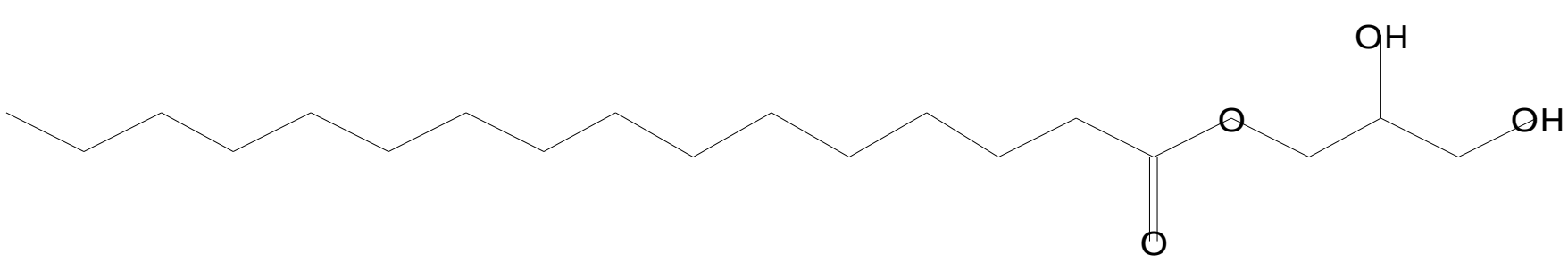

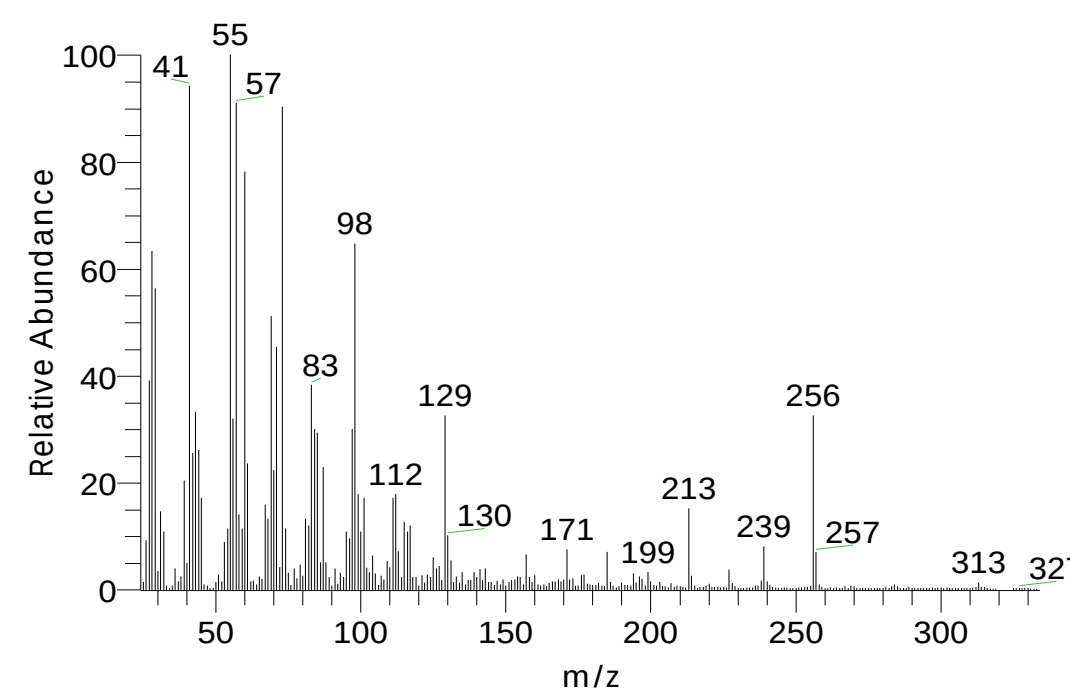

HEXADECANOIC ACID, 2-HYDROXY-1,3-PROPANEDIYL ESTER
Formula C35H68O5, MW 568, CAS# 502-52-3, Entry# 294143
2-HYDROXY-3-(PALMITOYLOXY)PROPYL PALMITATE #

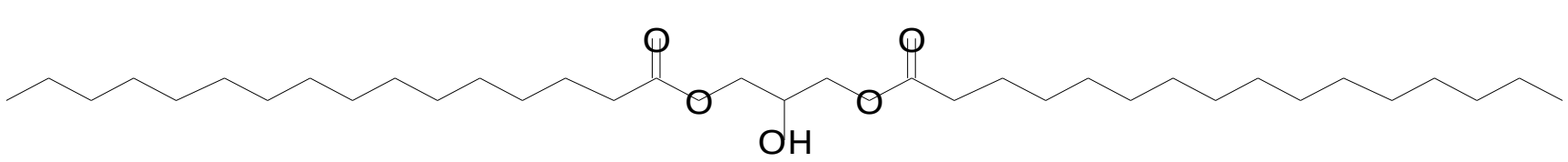

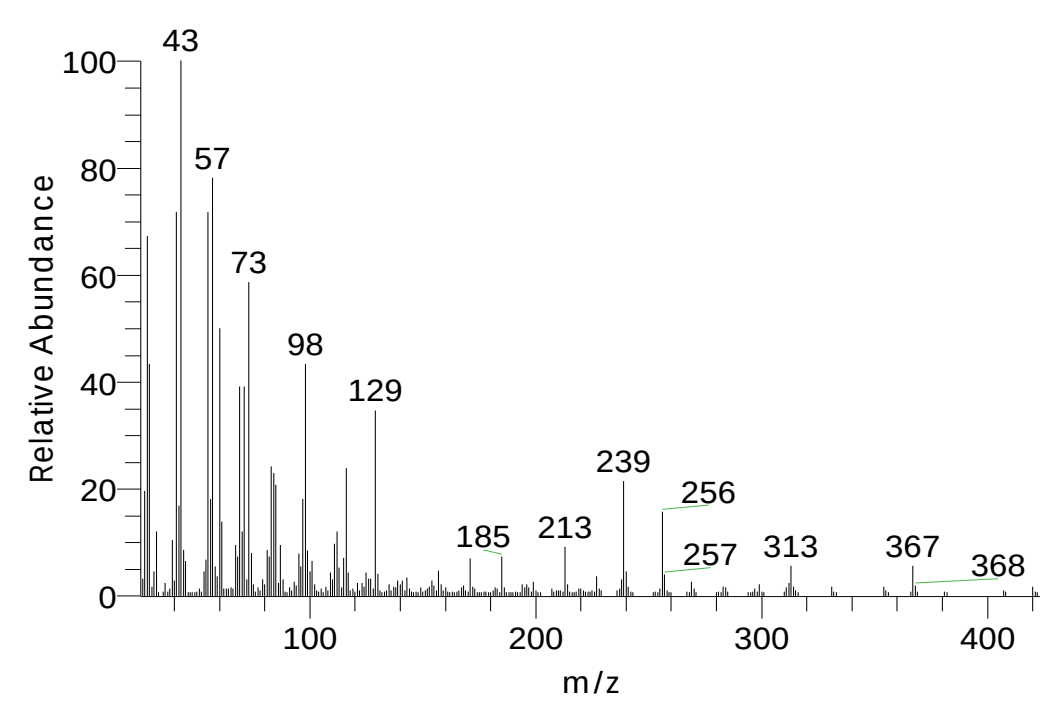

My GC-MS Report

Oil_DrHafsa #9318 RT: 35.25 AV: 1 NL: 3.45E5
T: + c EI Full ms [50.000-750.000]

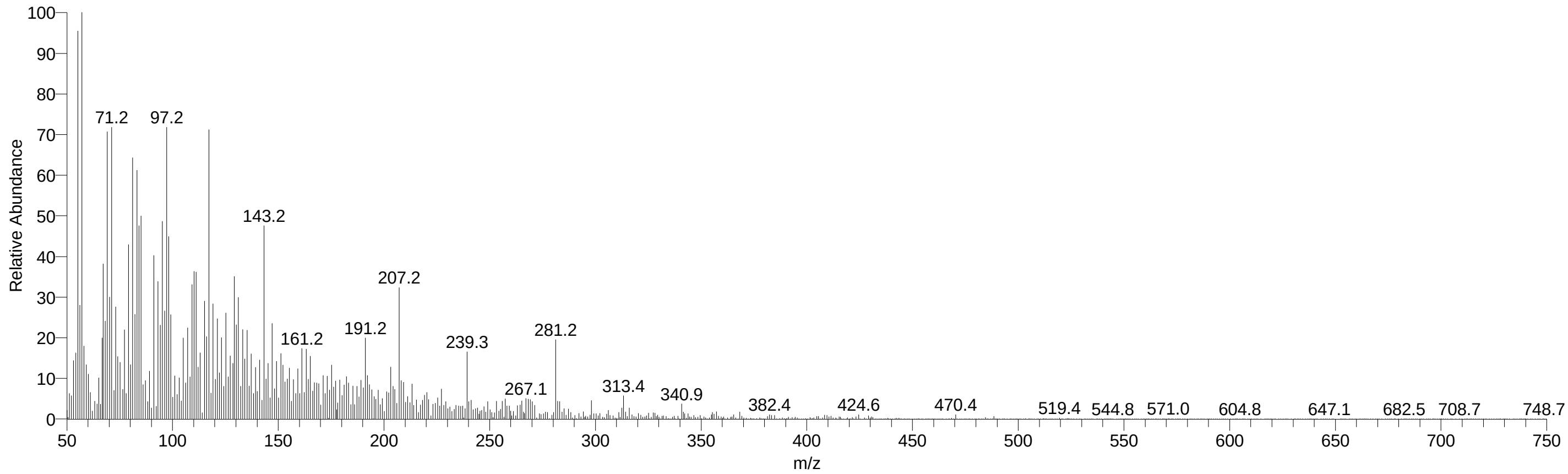

RT	Compound Name	Area %	MF	Molecular Formula	Molecular Weight	Cas #	Library
35.25	ISOCHIAPIN B	0.51	790	C19H22O6	346	NA	WileyRegistry8e
35.25	ISOCHIAPIN B %2<	0.51	790	C19H26O6	350	NA	WileyRegistry8e
35.25	4H-1-BENZOPYRAN-4-ONE, 2-(3,4-DIMETHOXYPHENYL)-3,5-DIHYDROXY-7-METHOXY-	0.51	757	C18H16O7	344	6068-80-0	WileyRegistry8e
35.25	4H-1-BENZOPYRAN-4-ONE, 2-(3,4-DIHYDROXYPHENYL)-6,8-DI-á-D-GLUCOPYRANOSYL-5,7-DIHYDROXY-	0.51	739	C27H30O16	610	29428-58-8	WileyRegistry8e
35.25	DOTRIACONTANE	0.51	775	C32H66	450	544-85-4	WileyRegistry8e

Compound Structure	Hit Spectrum

ISOCHIAPIN B
Formula C19H22O6, MW 346, CAS# NA, Entry# 225807

SI 774, RSI 790, WileyRegistry8e, Entry# 225807, CAS# NA, ISOCHIAPIN B

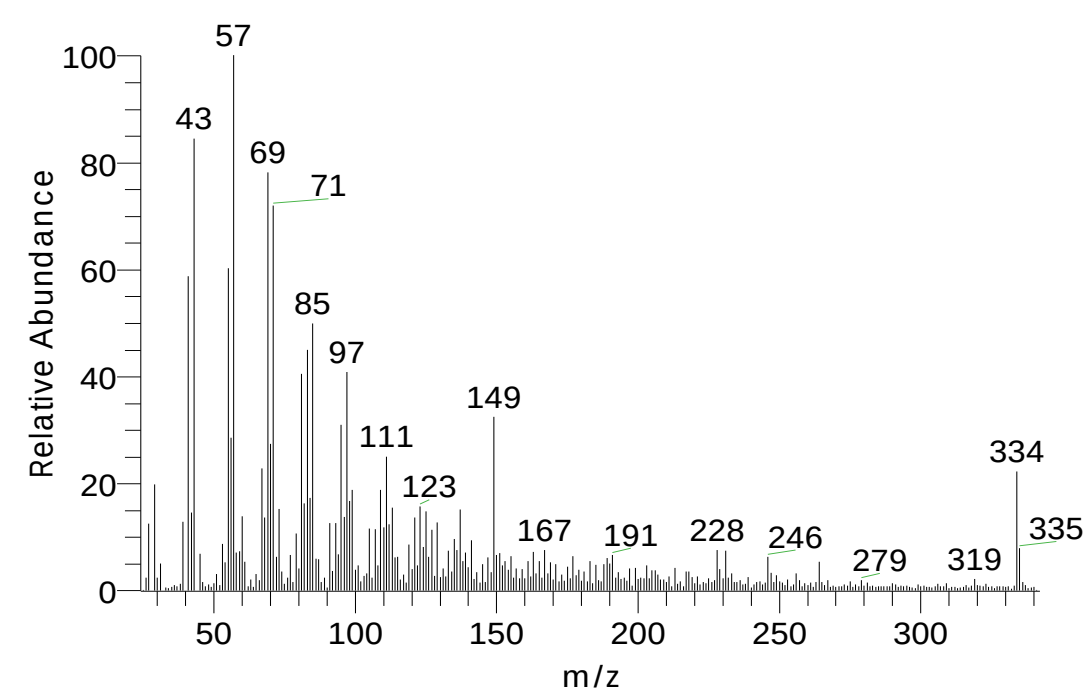

ISOCHIAPIN B %2<
Formula C19H26O6, MW 350, CAS# NA, Entry# 228500

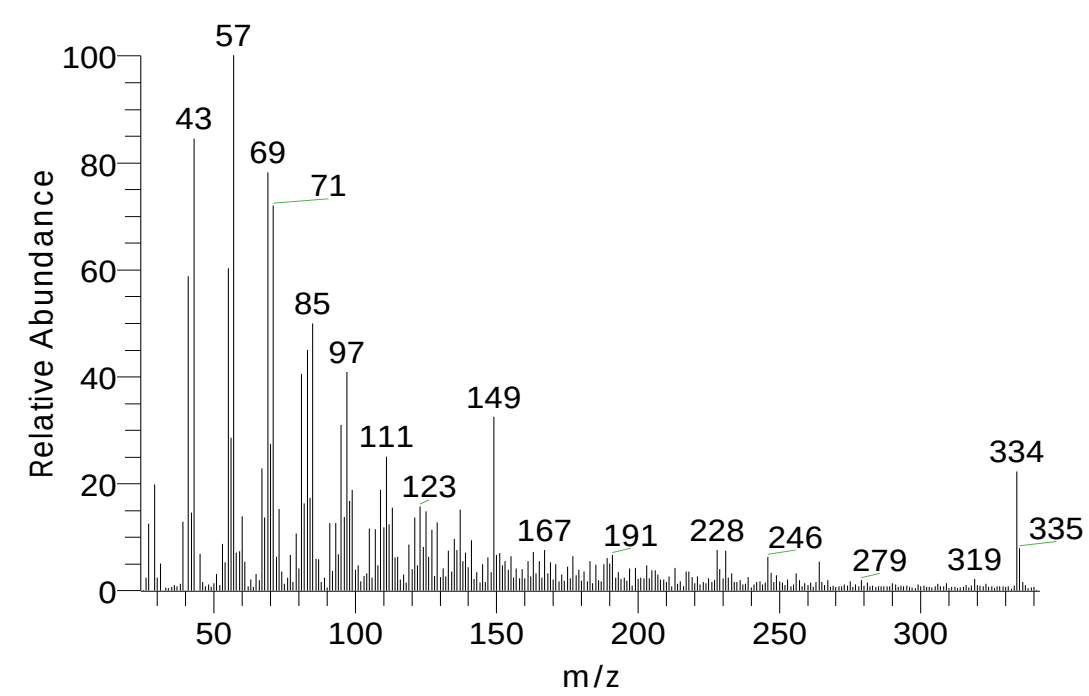

My GC-MS Report

Compound Structure | Hit Spectrum

4H-1-BENZOPYRAN-4-ONE, 2-(3,4-DIMETHOXYPHENYL)-3,5-DIHYDROXY-7-METHOXY-
Formula C18H16O7, MW 344, CAS# 6068-80-0, Entry# 224392
3',4',7-TRIMETHYLQUERCETIN

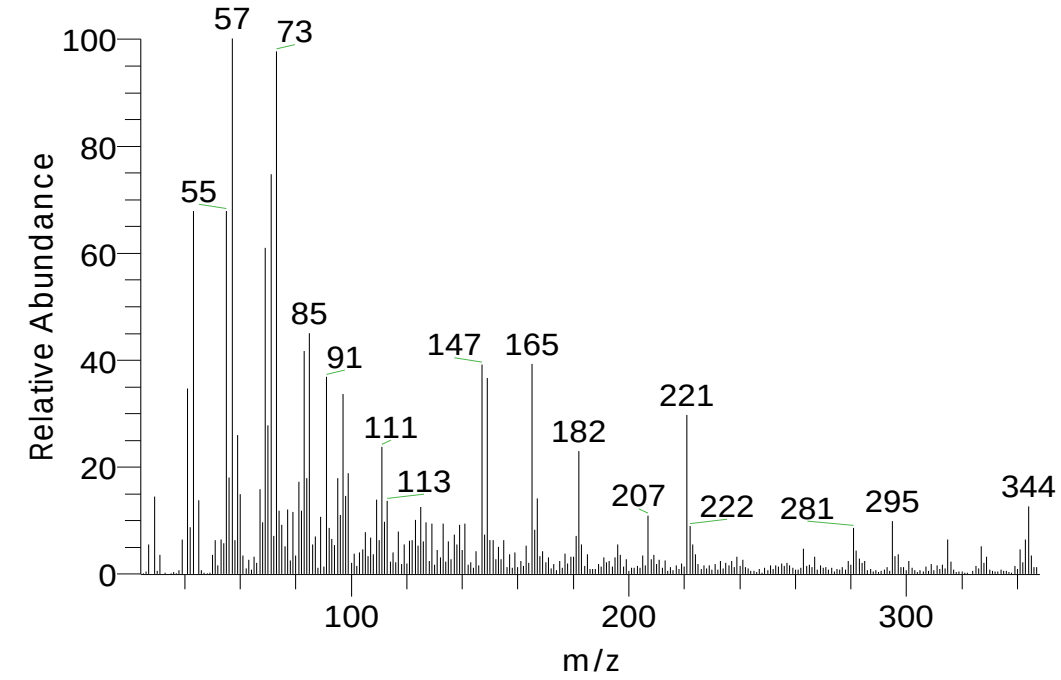

Formula C27H30O16, MW 610, CAS# 29428-58-8, Entry# 297453
6,8-DI-C-á-GLUCOSYLLUTEOLIN

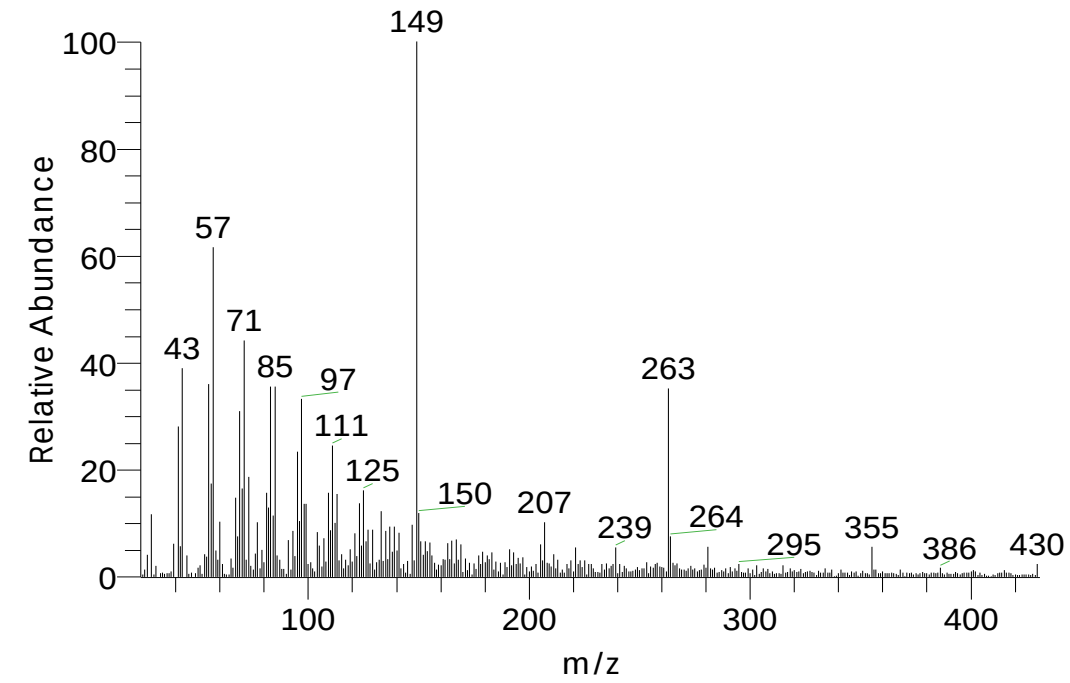

DOTRIACONTANE
Formula C32H66, MW 450, CAS# 544-85-4, Entry# 274478
AI3-52367

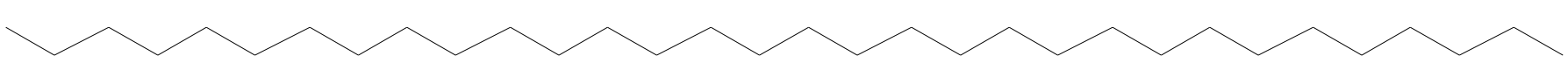

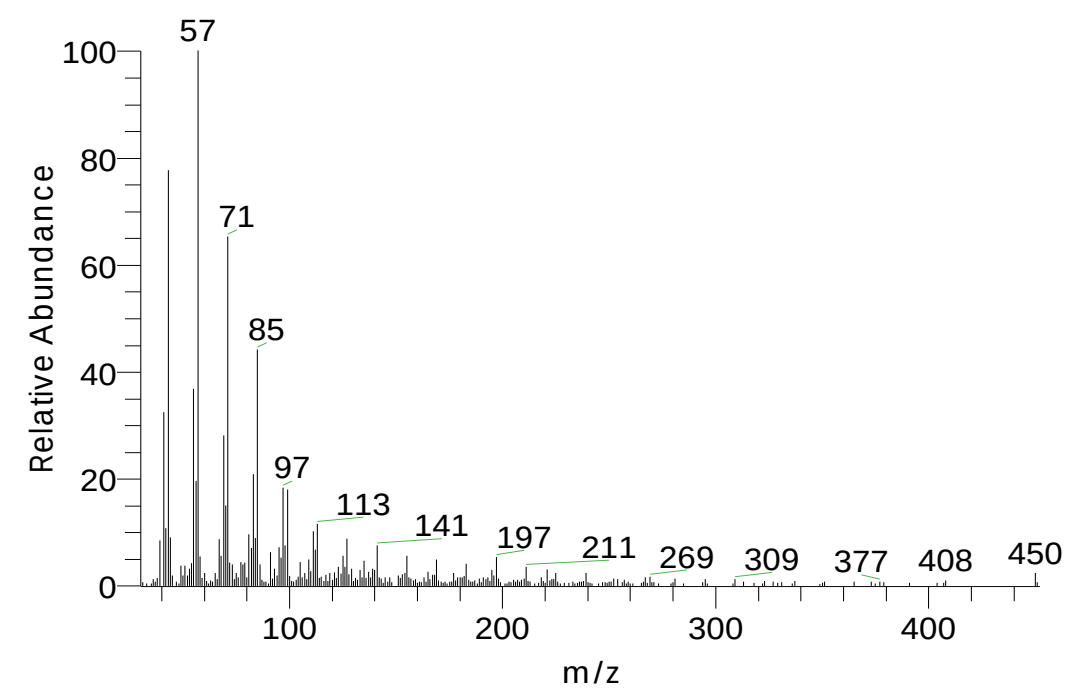

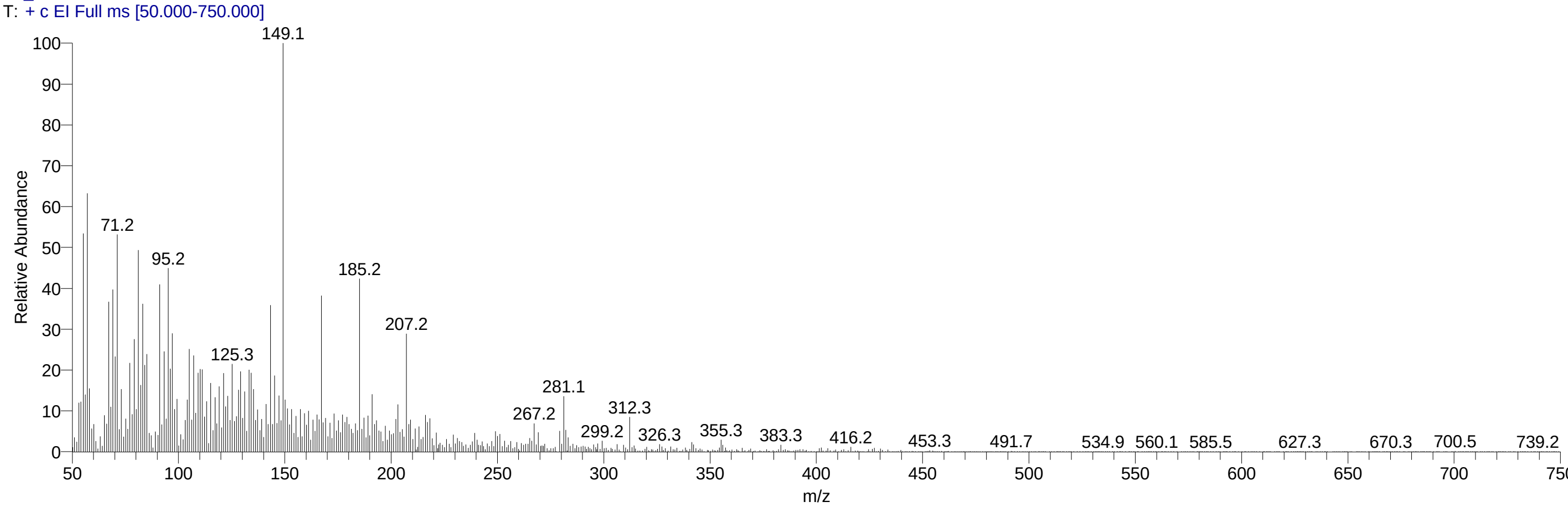

RT	Compound Name	Area %	MF	Molecular Formula	Molecular Weight	Cas #	Library
35.71	4H-1-BENZOPYRAN-4-ONE, 2-(3,4-DIHYDROXYPHENYL)-6,8-DI-á-D-GLUCOPYRANOSYL-5,7-DIHYDROXY-	0.38	754	C27H30O16	610	29428-58-8	WileyRegistry8e

My GC-MS Report

RT	Compound Name	Area %	MF	Molecular Formula	Molecular Weight	Cas #	Library
35.71	1-Heptatriacotanol	0.38	781	C37H76O	536	105794-58-9	mainlib
35.71	Rhodopin	0.38	715	C40H58O	554	105-92-0	mainlib
35.71	.PSI.,.PSI.-CAROTENE, 1,2-DIHYDRO-1-HYDROXY-	0.38	712	C40H58O	554	105-92-0	WileyRegistry8e
35.71	03027205002 FLAVONE 4'-OH,5-OH,7-DI-O-GLUCOSIDE	0.38	696	C27H30O15	594	NA	WileyRegistry8e

Compound Structure | Hit Spectrum

Formula C27H30O16, MW 610, CAS# 29428-58-8, Entry# 297453
6,8-DI-C-á-GLUCOSYLLUTEOLIN

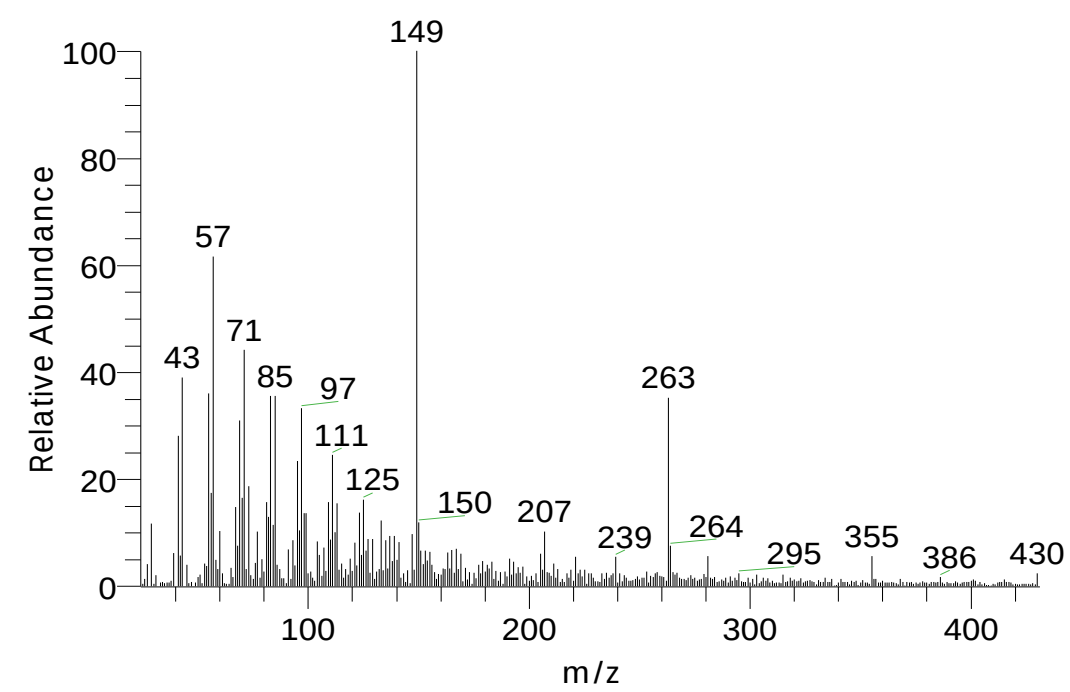

1-Heptatriacotanol
Formula C37H76O, MW 536, CAS# 105794-58-9, Entry# 7279
1-Heptatriacontanol #

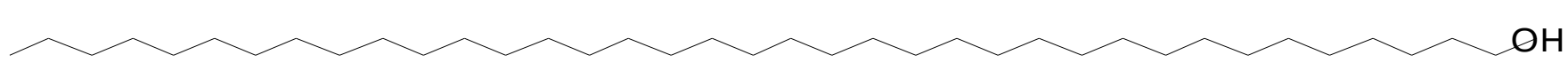

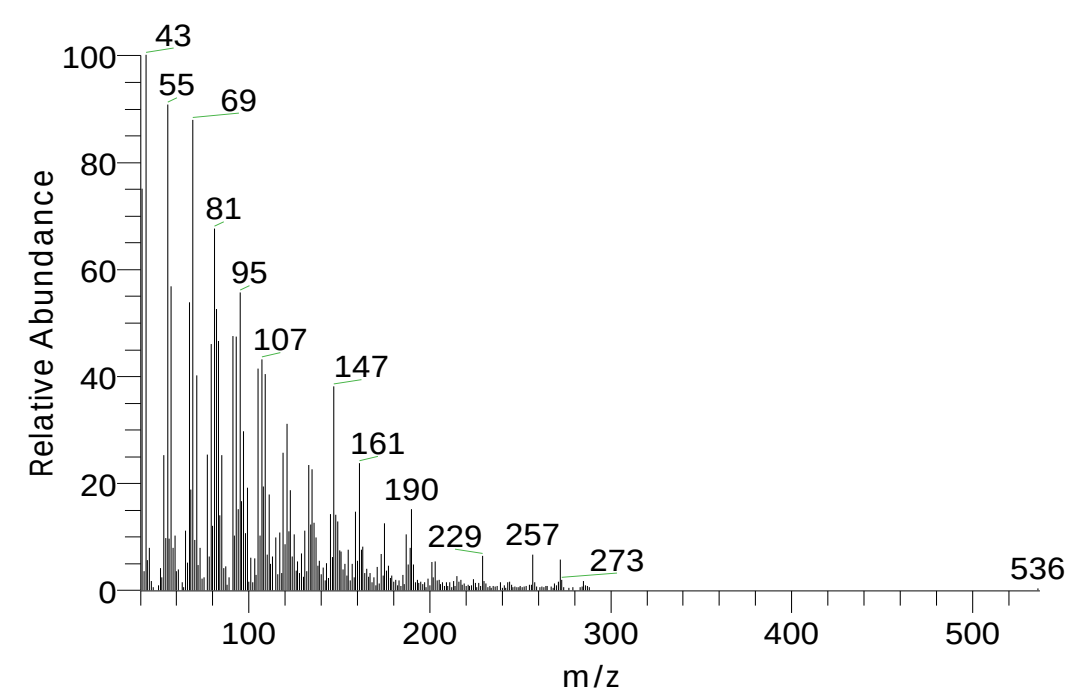

Rhodopin
Formula C40H58O, MW 554, CAS# 105-92-0, Entry# 34358
.psi.,.psi.-Carotene, 1,2-dihydro-1-hydroxy-

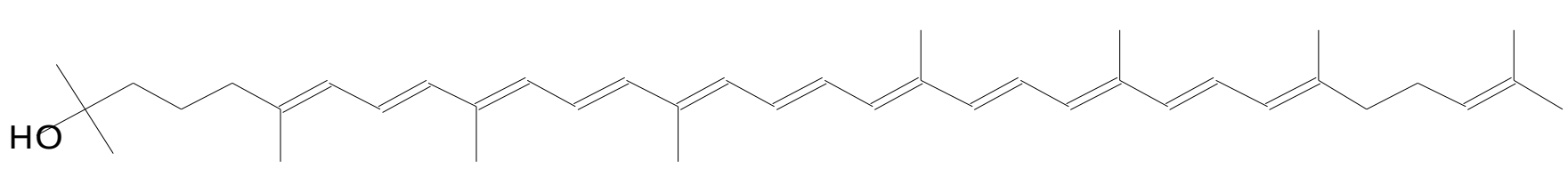

SI 714, RSI 715, mainlib, Entry# 34358, CAS# 105-92-0, Rhodopin

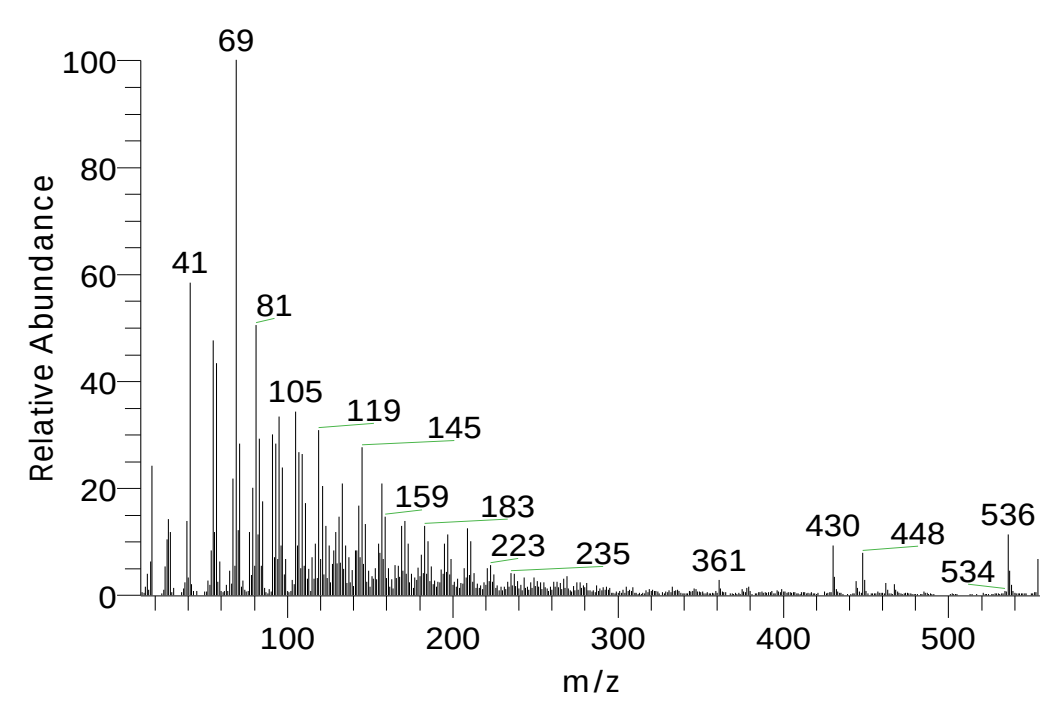

.PSI.,.PSI.-CAROTENE, 1,2-DIHYDRO-1-HYDROXY-
Formula C40H58O, MW 554, CAS# 105-92-0, Entry# 292854
1,2-DIHYDRO-PSI,PSI-CAROTENE #

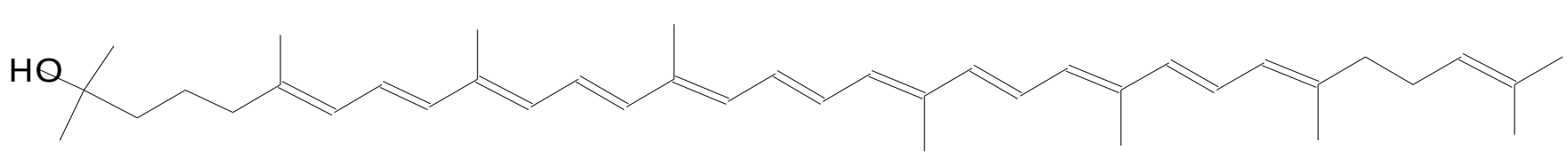

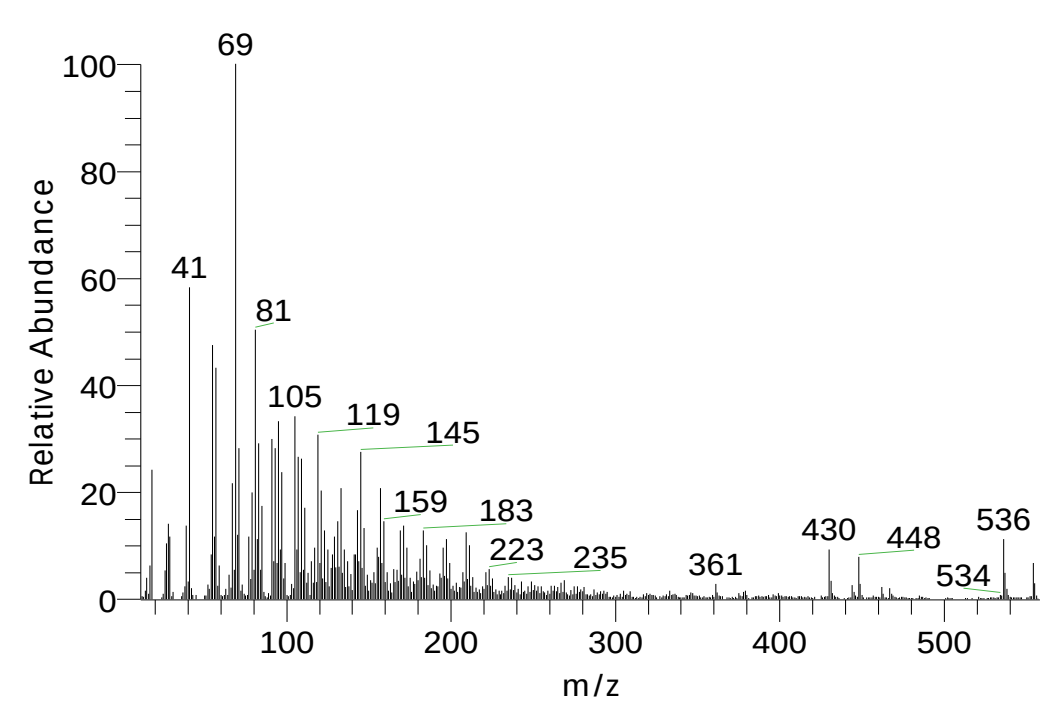

My GC-MS Report

Compound Structure | Hit Spectrum

03027205002 FLAVONE 4'-OH,5-OH,7-DI-O-GLUCOSIDE
Formula C27H30O15, MW 594, CAS# NA, Entry# 296184

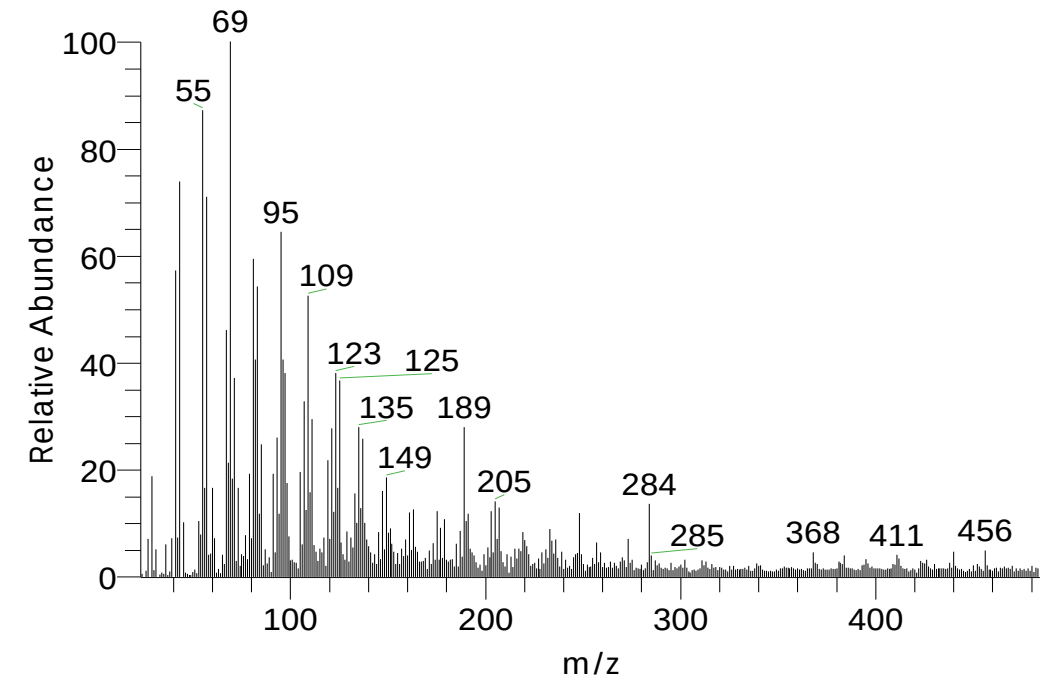

Oil_DrHafsa #9504 RT: 35.87 AV: 1 NL: 3.03E5
T: + c EI Full ms [50.000-750.000]

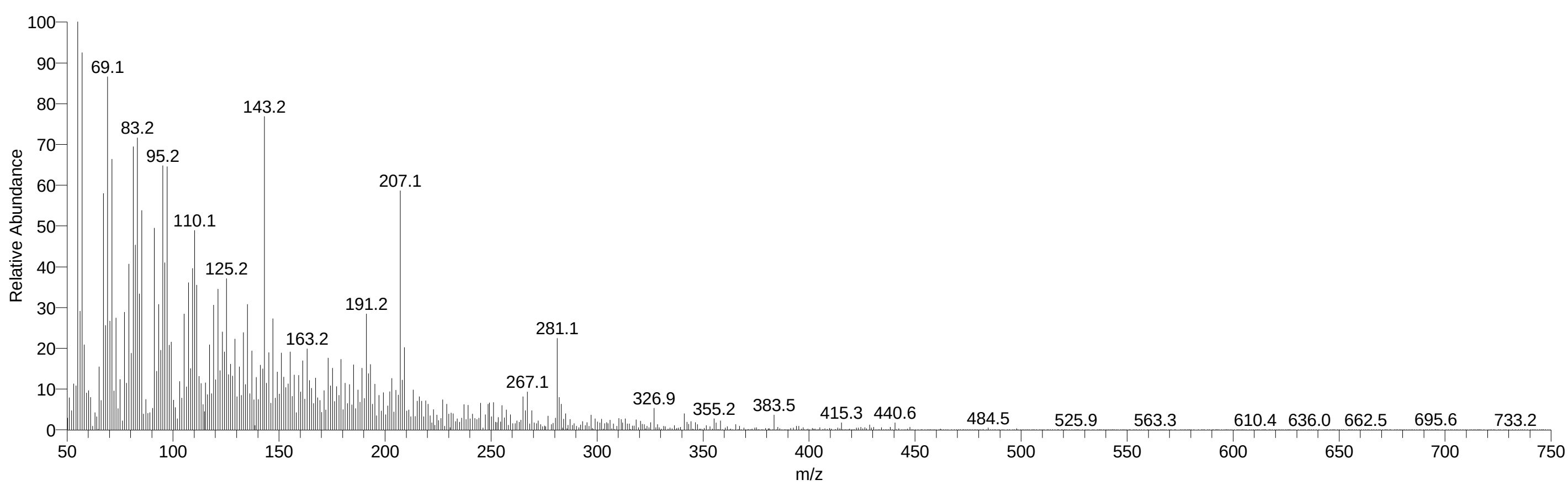

RT	Compound Name	Area %	MF	Molecular Formula	Molecular Weight	Cas #	Library
35.87	Ethyl iso-allocholate	0.26	772	C26H44O5	436	NA	mainlib
35.87	ETHYL ISO-ALLOCHOLATE	0.26	772	C26H44O5	436	NA	WileyRegistry8e
35.87	1-Heptatriacotanol	0.26	796	C37H76O	536	105794-58-9	mainlib
35.87	01297107001 TETRANEURIN - A - DIOL	0.26	792	C15H20O5	280	NA	WileyRegistry8e
35.87	7,8-Epoxylanostan-11-ol, 3-acetoxy-	0.26	726	C32H54O4	502	NA	mainlib

Compound Structure | Hit Spectrum

Ethyl iso-allocholate
Formula C26H44O5, MW 436, CAS# NA, Entry# 7020
Ethyl 3,7,12-trihydroxycholan-24-oate #

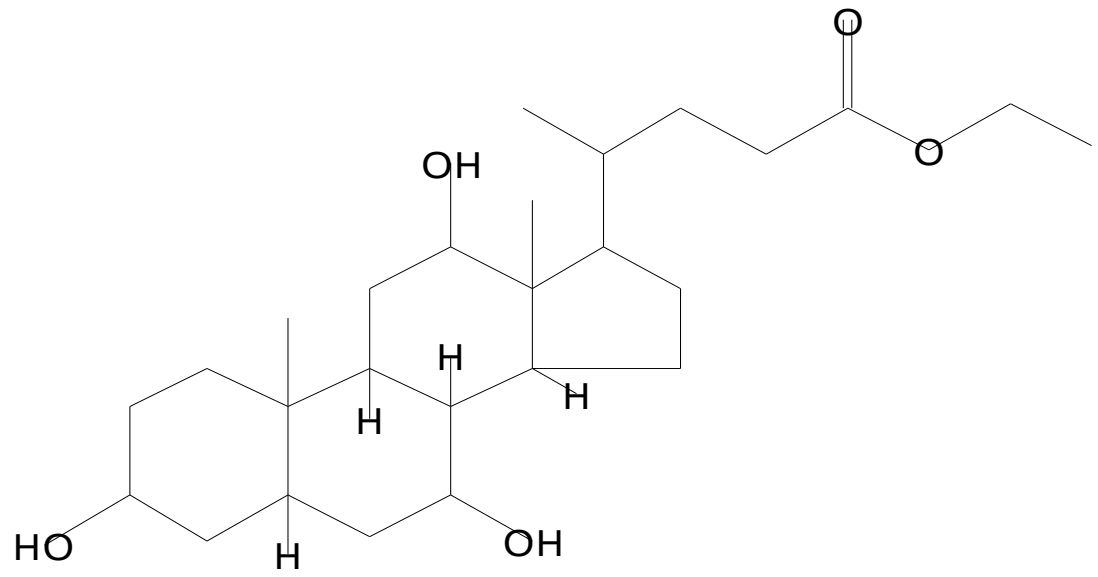

SI 764, RSI 772, mainlib, Entry# 7020, CAS# NA, Ethyl iso-allocholate

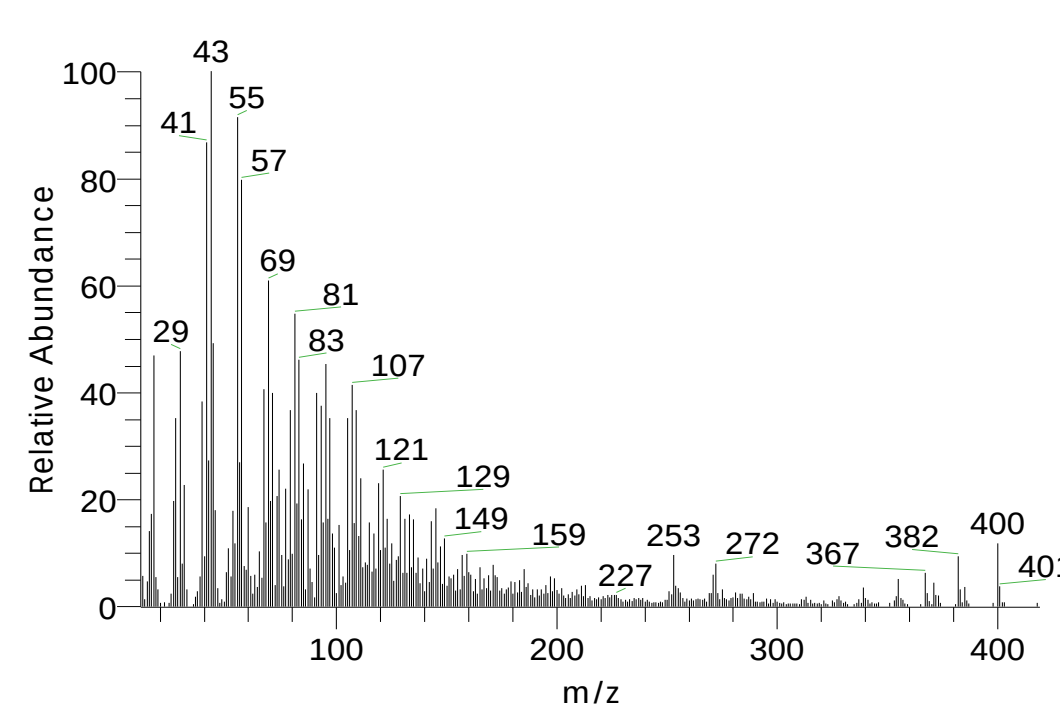

My GC-MS Report

Compound Structure | Hit Spectrum

ETHYL ISO-ALLOCHOLATE
Formula C26H44O5, MW 436, CAS# NA, Entry# 270212

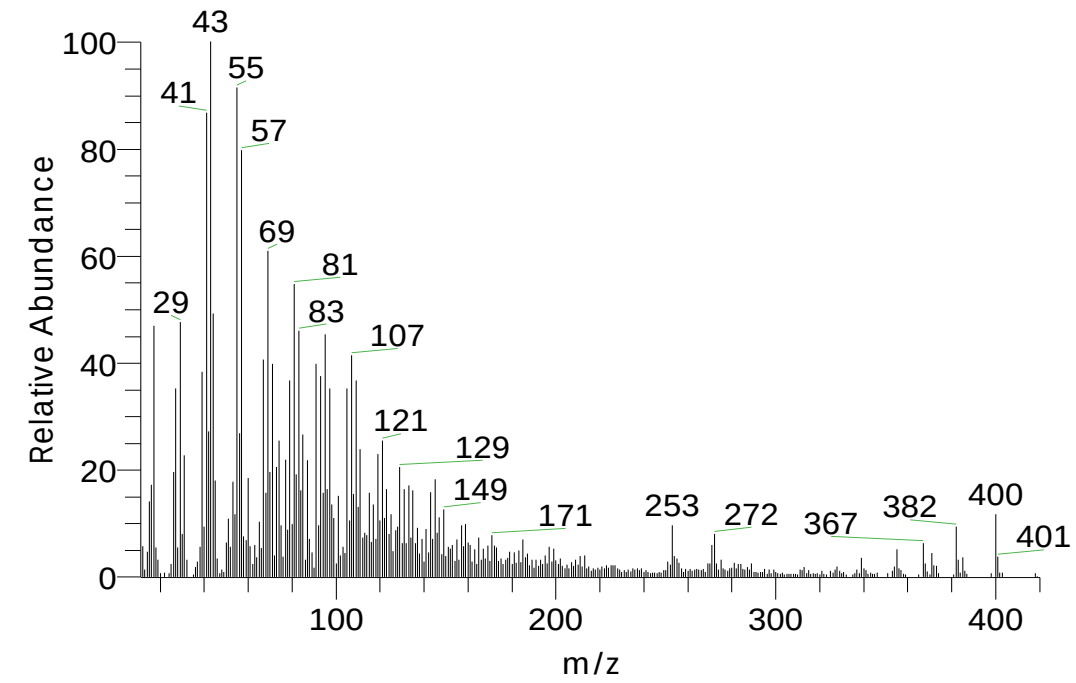

1-Heptatriacotanol
Formula C37H76O, MW 536, CAS# 105794-58-9, Entry# 7279
1-Heptatriacontanol #

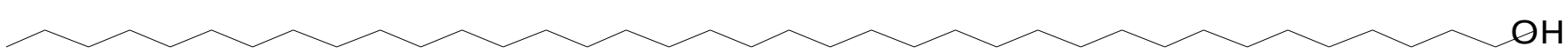

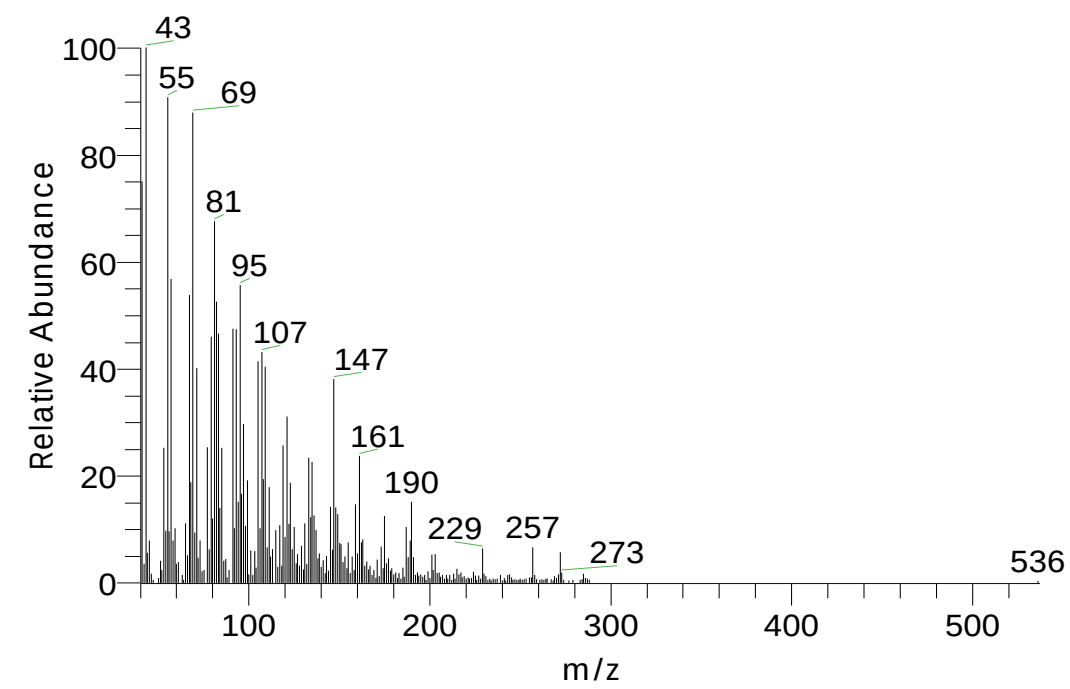

01297107001 TETRANEURIN - A - DIOL
Formula C15H20O5, MW 280, CAS# NA, Entry# 170378

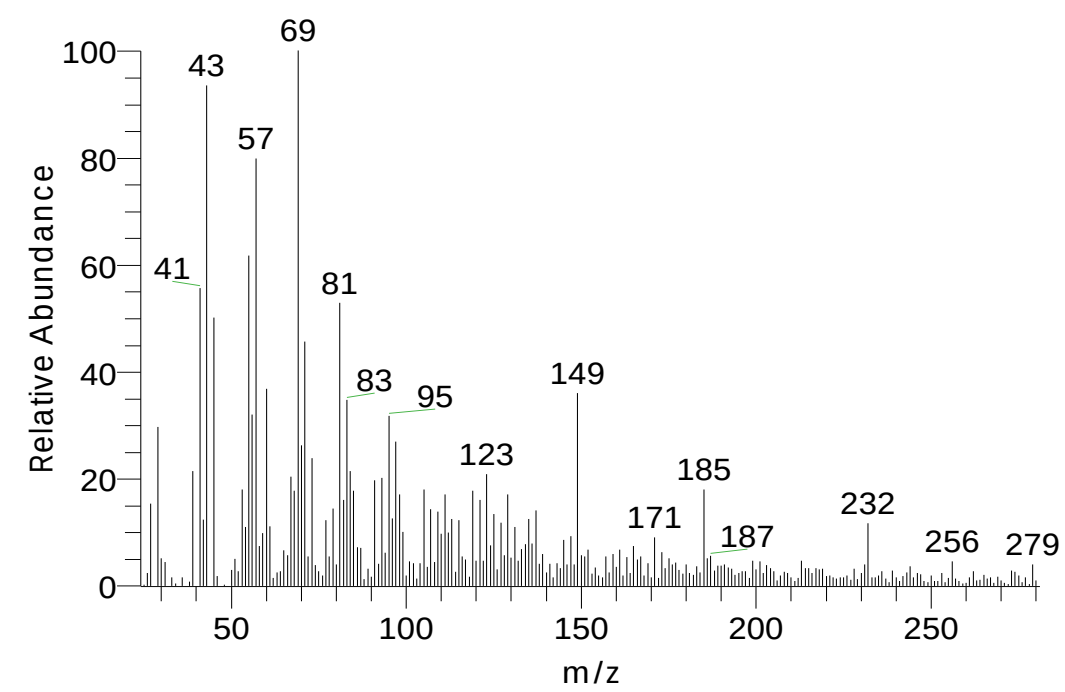

7,8-Epoxylanostan-11-ol, 3-acetoxy-
Formula C32H54O4, MW 502, CAS# NA, Entry# 15359

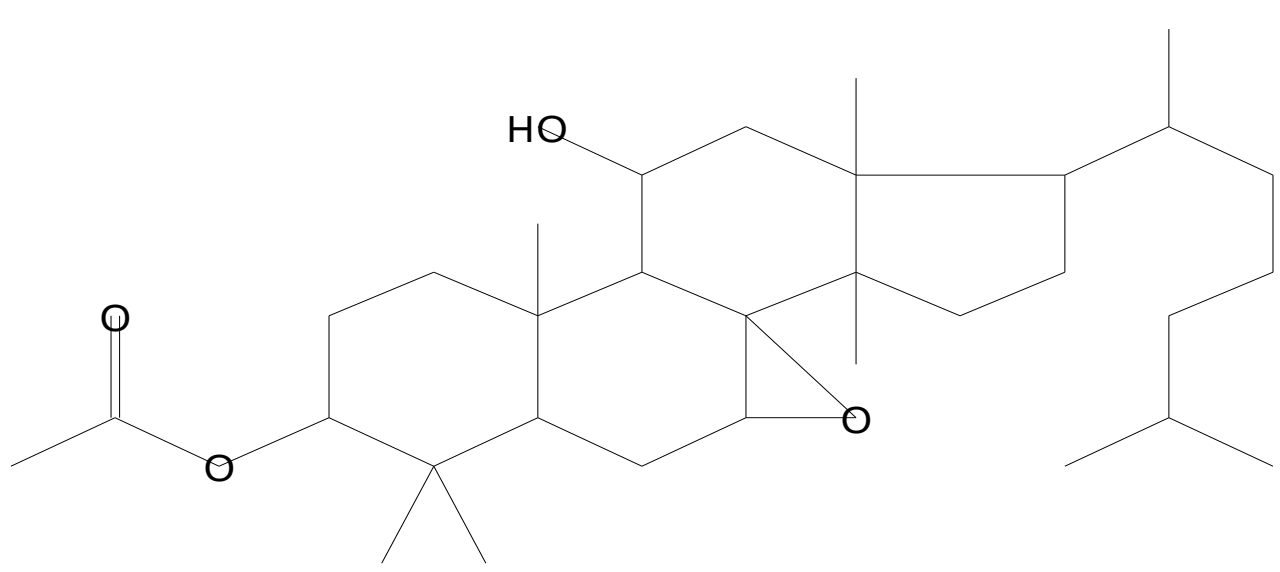

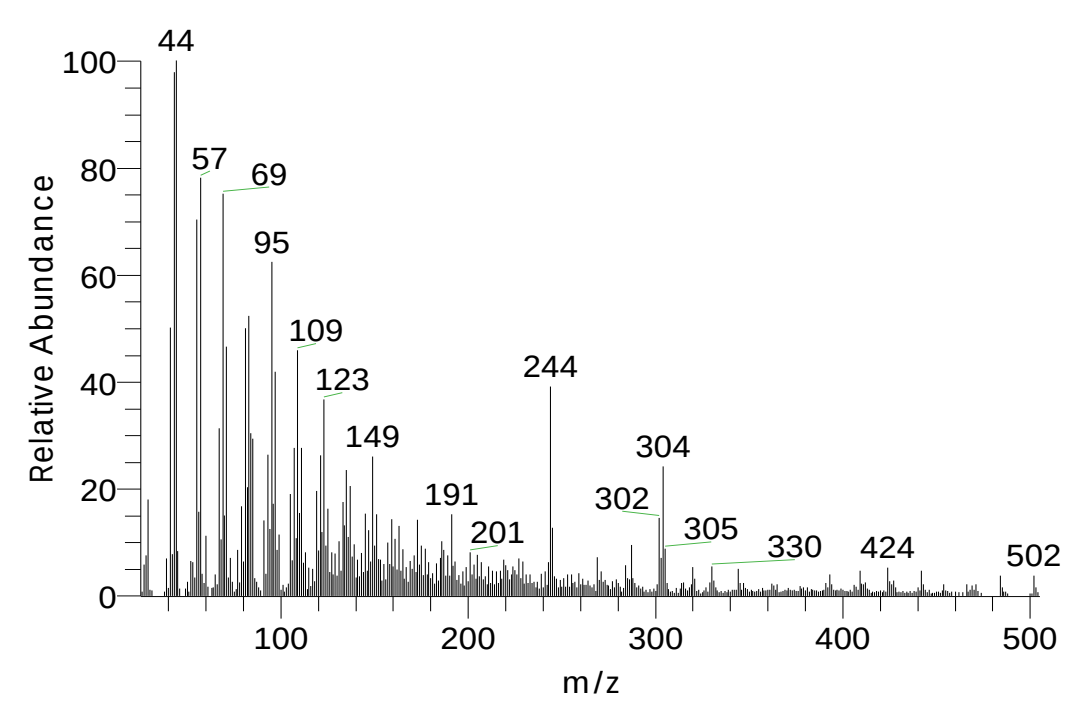

My GC-MS Report

Oil_DrHafsa #9665 RT: 36.41 AV: 1 NL: 1.10E6
T: + c EI Full ms [50.000-750.000]

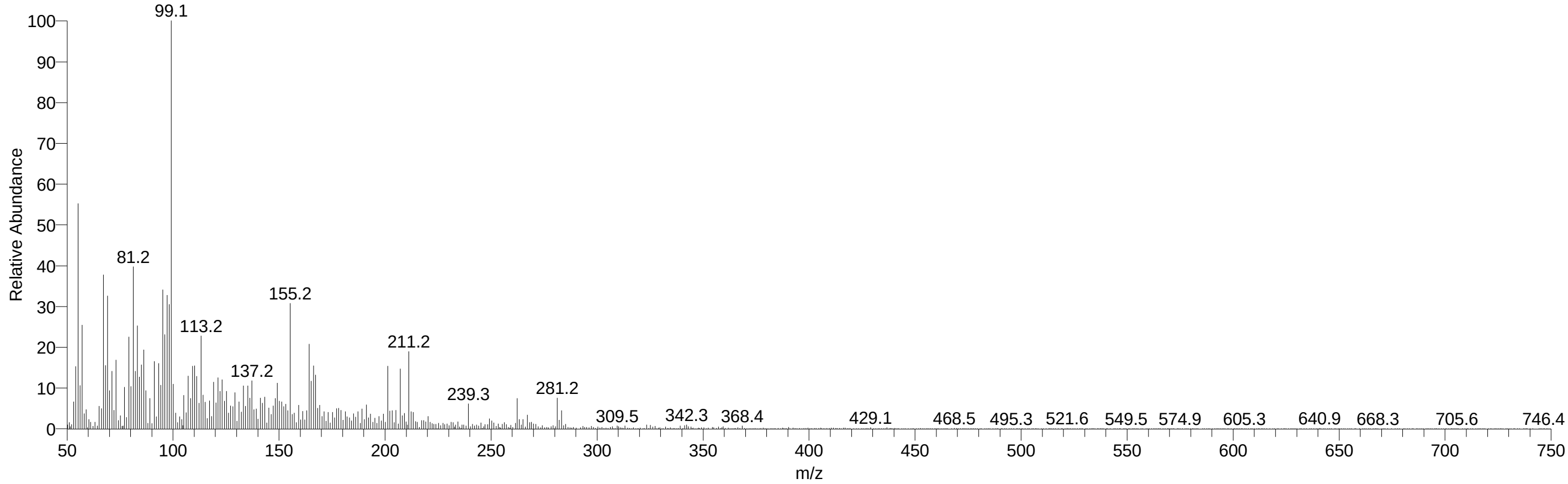

RT	Compound Name	Area %	MF	Molecular Formula	Molecular Weight	Cas #	Library
36.41	9,12,15-OCTADECATRIENOIC ACID, 2-[(TRIMETHYLSILYL)OXY]-1-[[(TRIMETHYLSILYL)OXY]METHYL]ETHYL ESTER, (Z,Z,Z)-	1.26	751	C27H52O4Si2	496	55521-23-8	WileyRegistry8e
36.41	Linoleic acid ethyl ester	1.26	730	C20H36O2	308	544-35-4	replib
36.41	TRIDEUTERIOMETHYL 10-EPOXY-7-ETHYL-3,11-DIMETHYLTRIDECA-2,6-DIENOATE	1.26	749	C18H27D3O3	297	56805-11-9	WileyRegistry8e
36.41	1,4-Dioxaspiro[4.5]decane-7-butanoic acid, 6-methyl-, 2-(methylsulfonyloxy)ethyl ester	1.26	729	C16H28O7S	364	NA	mainlib
36.41	2-[(METHYLSULFONYL)OXY]ETHYL 4-(6-METHYL-1,4-DIOXASPIRO[4.5]DEC-7-YL)BUTANOATE	1.26	729	C16H28O7S	364	NA	WileyRegistry8e

Compound Structure | Hit Spectrum

Formula C27H52O4Si2, MW 496, CAS# 55521-23-8, Entry# 284836

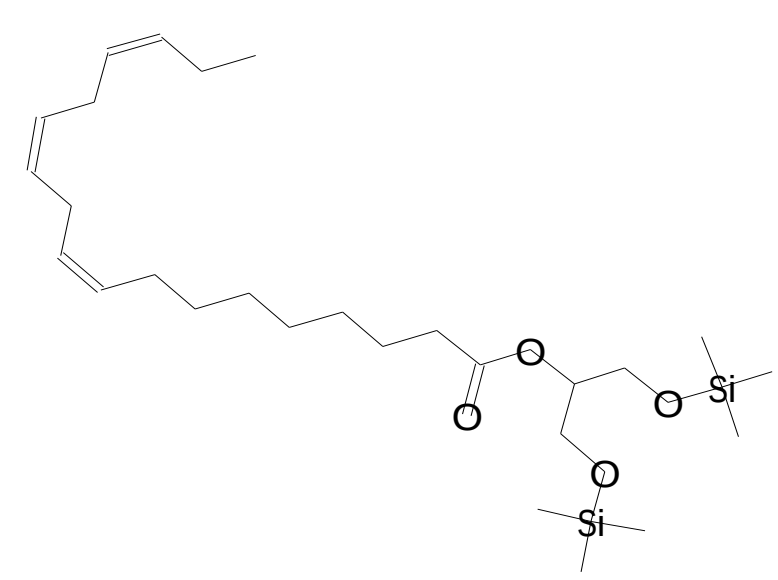

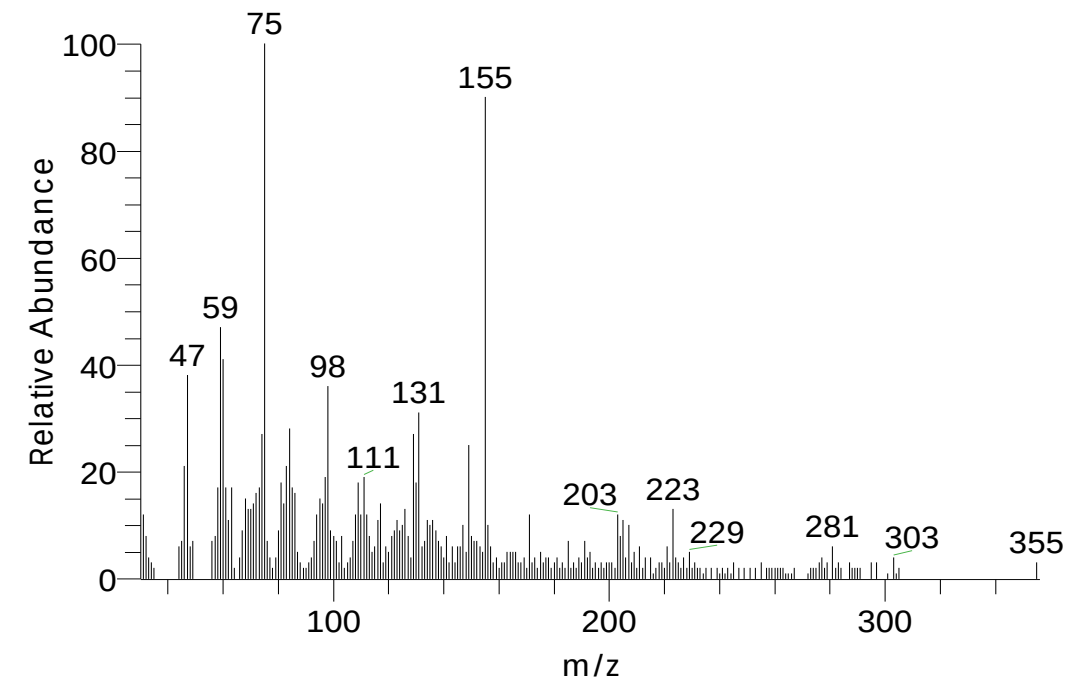

Linoleic acid ethyl ester
Formula C20H36O2, MW 308, CAS# 544-35-4, Entry# 8097
Ethyl linoleate

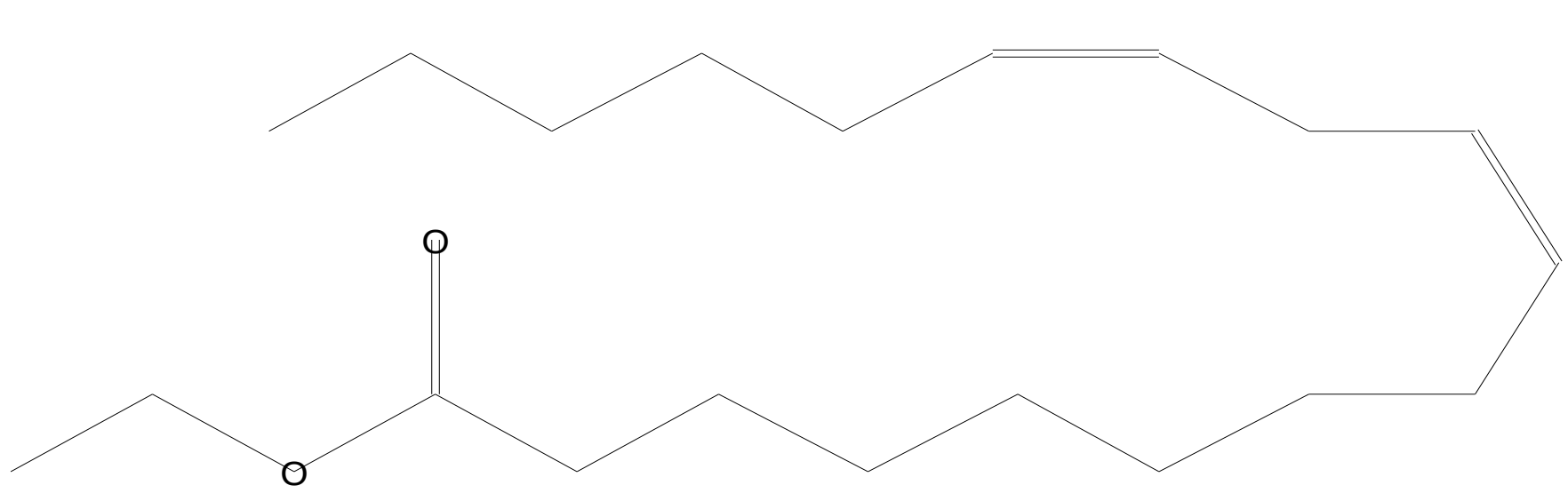

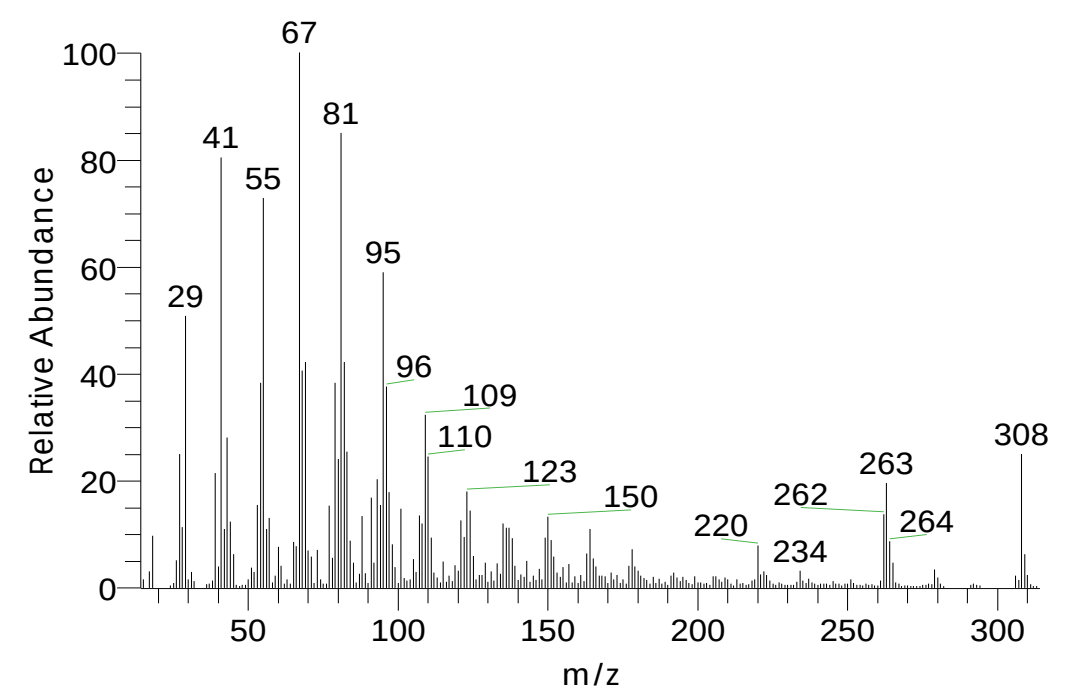

My GC-MS Report

Compound Structure | Hit Spectrum

TRIDEUTERIOMETHYL 10-EPOXY-7-ETHYL-3,11-DIMETHYLTRIDECA-2,6-DIENOATE
Formula C18H27D3O3, MW 297, CAS# 56805-11-9, Entry# 184042

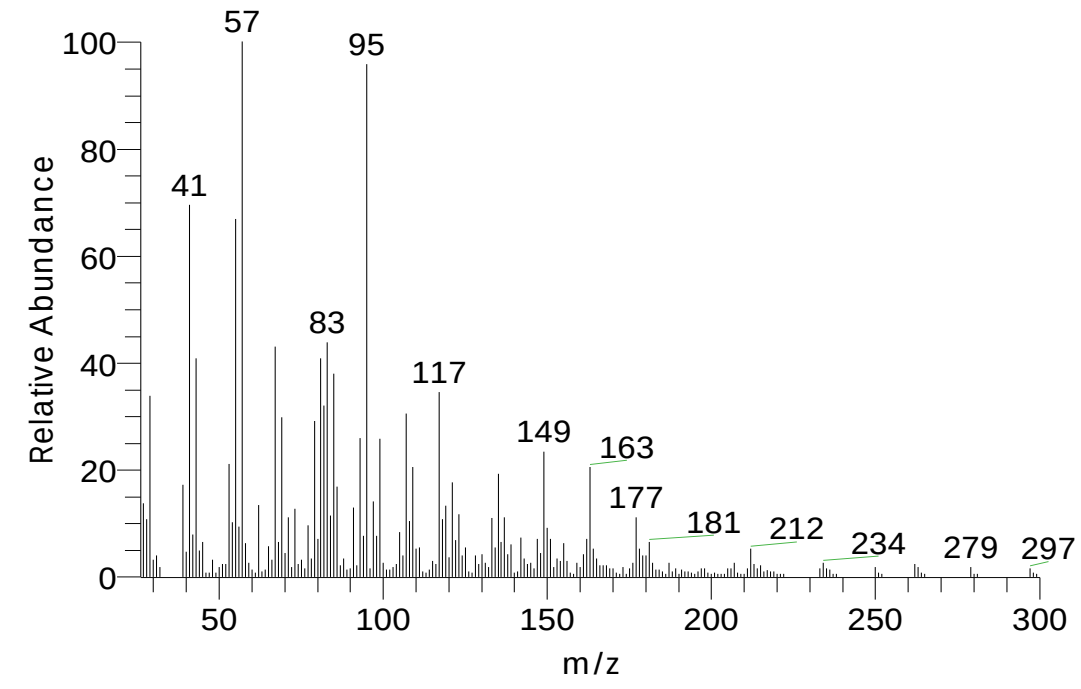

1,4-Dioxaspiro[4.5]decane-7-butanoic acid, 6-methyl-, 2-(methylsulfonyloxy)ethyl ester
Formula C16H28O7S, MW 364, CAS# NA, Entry# 72418
2-[(Methylsulfonyl)oxy]ethyl 4-(6-methyl-1,4-dioxaspiro[4.5]dec-7-yl)butanoate #

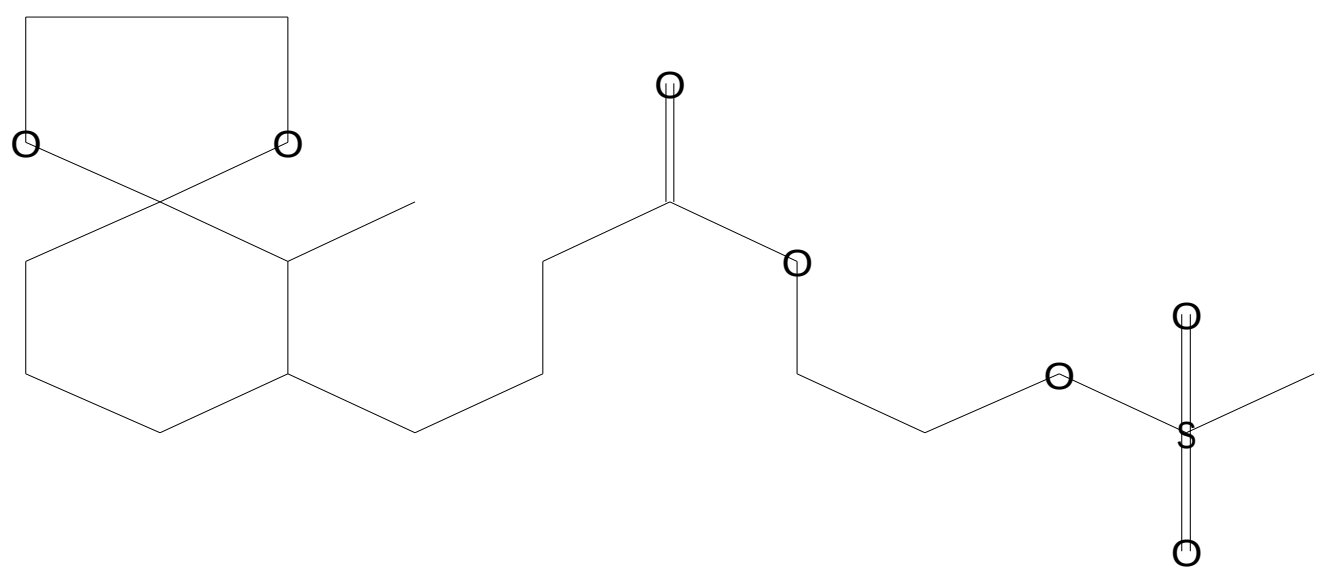

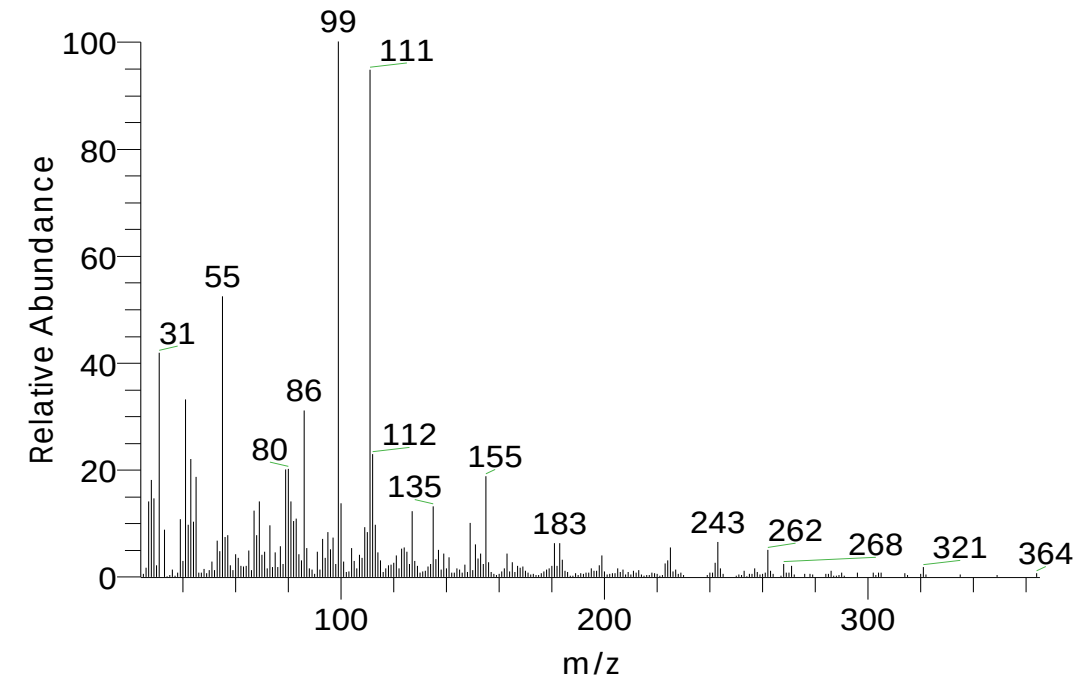

2-[(METHYLSULFONYL)OXY]ETHYL 4-(6-METHYL-1,4-DIOXASPIRO[4.5]DEC-7-YL)BUTANOATE
Formula C16H28O7S, MW 364, CAS# NA, Entry# 365591

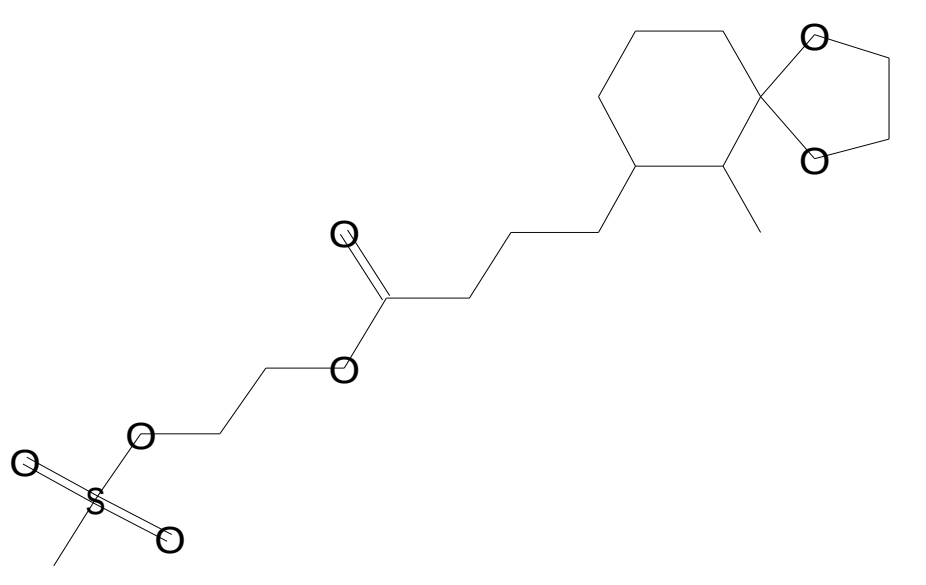

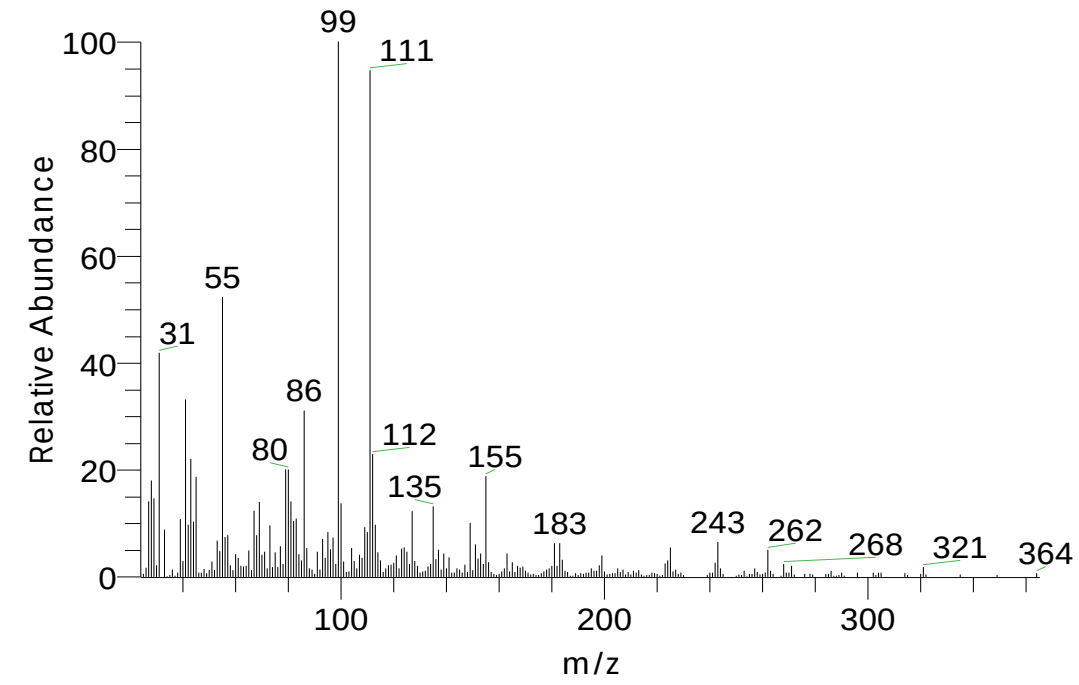

Oil_DrHafsa #9934 RT: 37.31 AV: 1 NL: 9.14E5
T: + c EI Full ms [50.000-750.000]

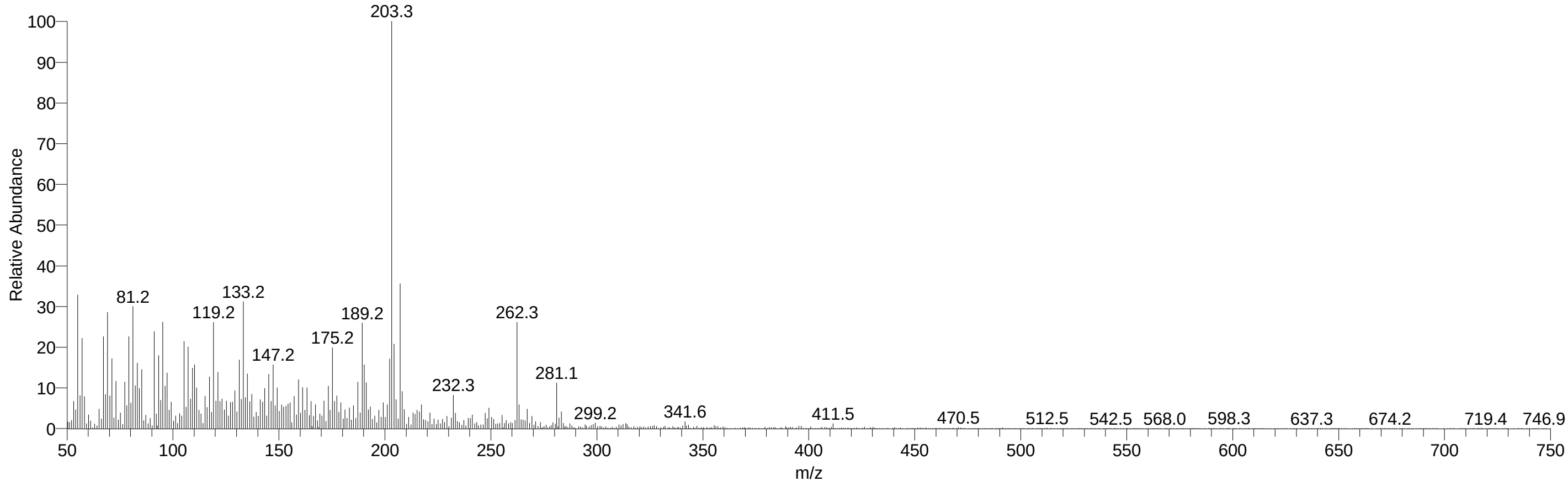

RT	Compound Name	Area %	MF	Molecular Formula	Molecular Weight	Cas #	Library
37.31	Urs-12-en-28-oic acid, 3-hydroxy-, methyl ester, (3á)-	1.21	832	C31H50O3	470	32208-45-0	mainlib
37.31	URS-12-EN-28-OIC ACID, 3-HYDROXY-, METHYL ESTER, (3á)-	1.21	829	C31H50O3	470	32208-45-0	WileyRegistry8e

My GC-MS Report

RT	Compound Name	Area %	MF	Molecular Formula	Molecular Weight	Cas #	Library
37.31	Betulin	1.21	753	C30H50O2	442	473-98-3	replib
37.31	LUP-20(29)-ENE-3,28-DIOL, (3á)-	1.21	800	C30H50O2	442	473-98-3	WileyRegistry8e
37.31	Betulinaldehyde	1.21	747	C30H48O2	440	13159-28-9	mainlib

Compound Structure	Hit Spectrum

Urs-12-en-28-oic acid, 3-hydroxy-, methyl ester, (3á)-
Formula C31H50O3, MW 470, CAS# 32208-45-0, Entry# 214160
Urs-12-en-28-oic acid, 3á-hydroxy-, methyl ester

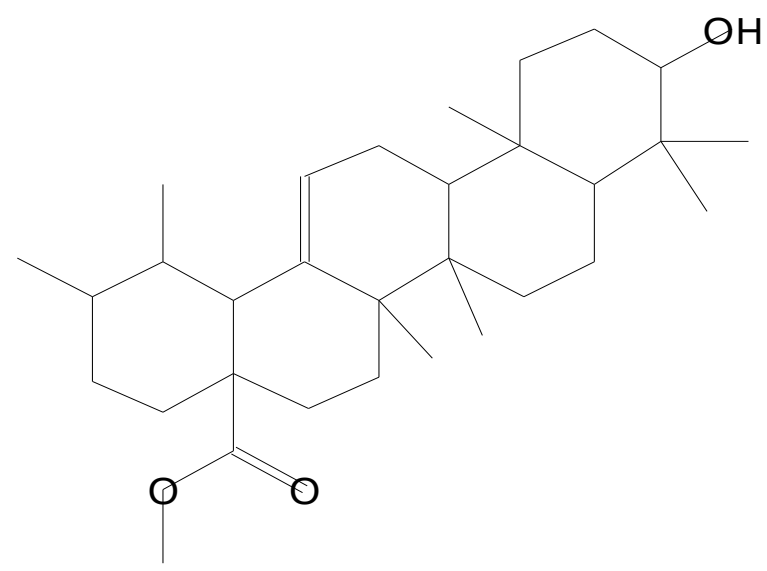

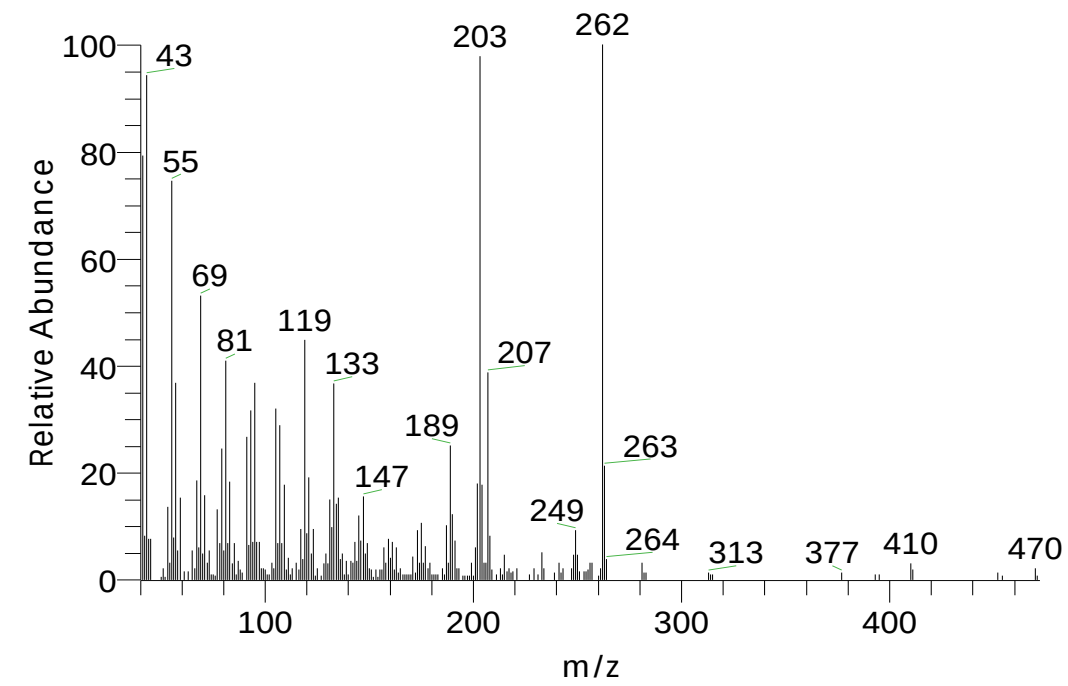

URS-12-EN-28-OIC ACID, 3-HYDROXY-, METHYL ESTER, (3á)-
Formula C31H50O3, MW 470, CAS# 32208-45-0, Entry# 279567
METHYL 3-HYDROXYURS-12-EN-28-OATE #

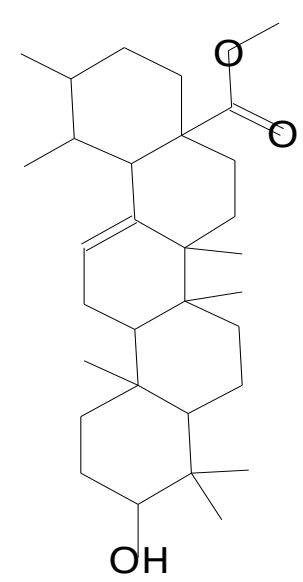

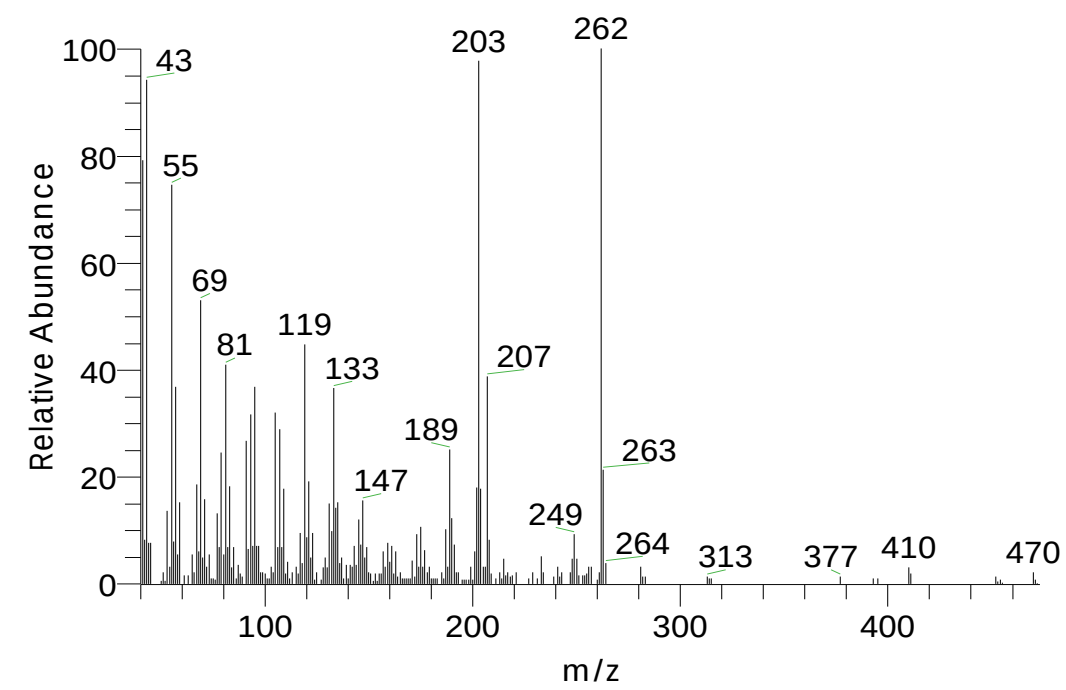

Betulin
Formula C30H50O2, MW 442, CAS# 473-98-3, Entry# 27738
Lup-20(29)-ene-3,28-diol, (3á)-

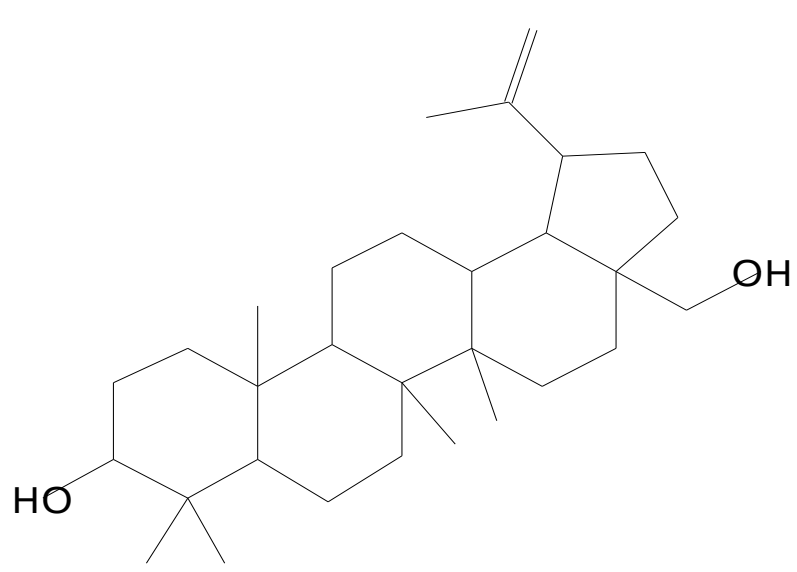

SI 751, RSI 753, replib, Entry# 27738, CAS# 473-98-3, Betulin

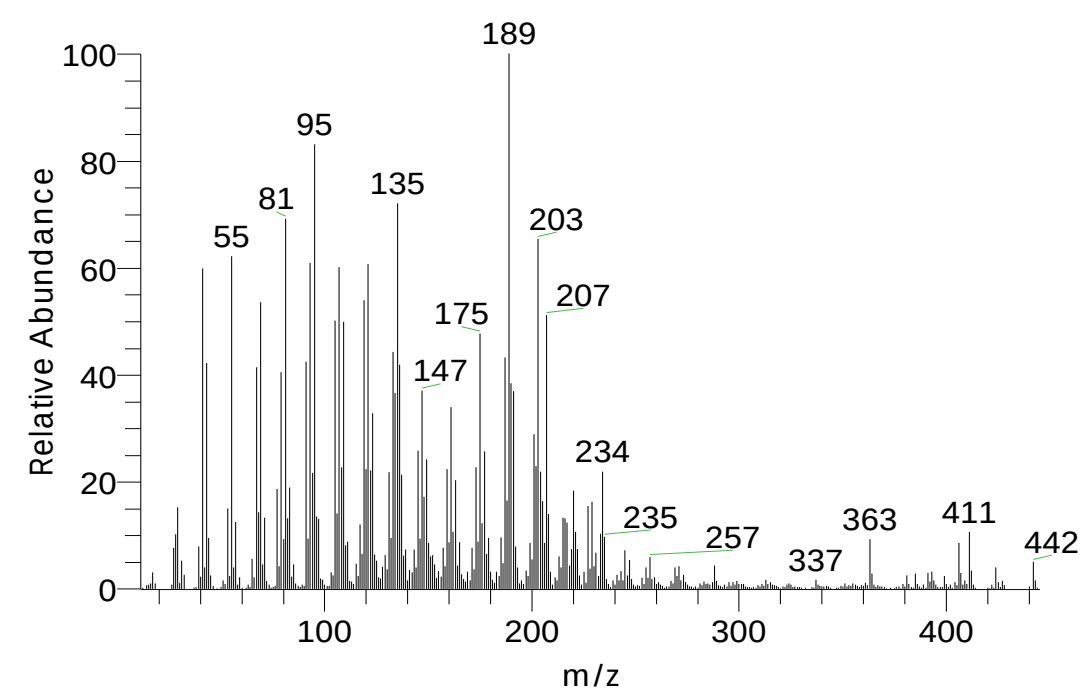

LUP-20(29)-ENE-3,28-DIOL, (3á)-
Formula C30H50O2, MW 442, CAS# 473-98-3, Entry# 349524
LUP-20(29)-ENE-3,28-DIOL #

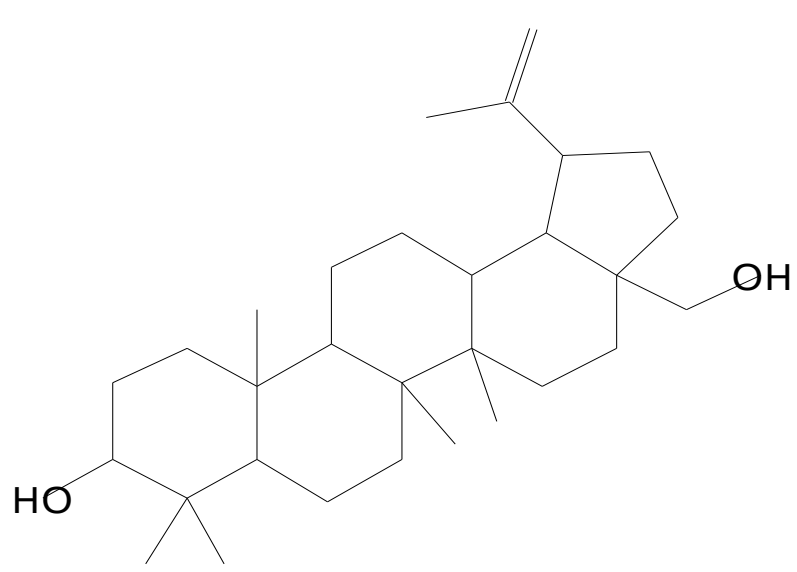

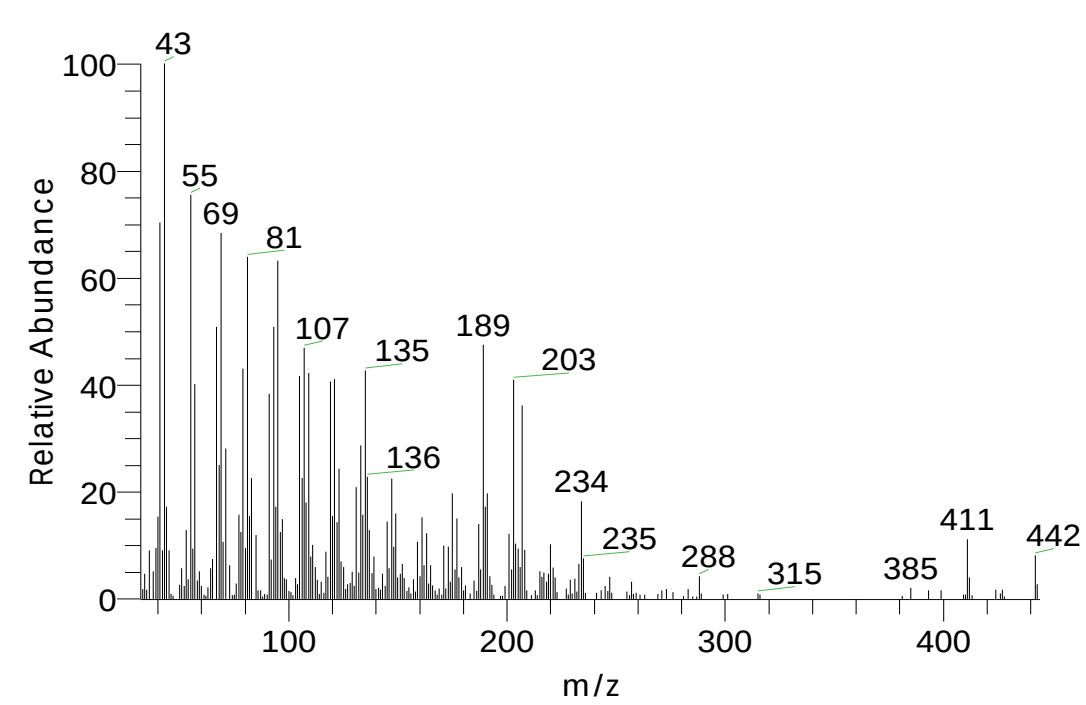

My GC-MS Report

Compound Structure | Hit Spectrum

Betulinaldehyde
Formula C30H48O2, MW 440, CAS# 13159-28-9, Entry# 175929
$:28FELCJAPFJOPHSD-UHFFFAOYSA-N

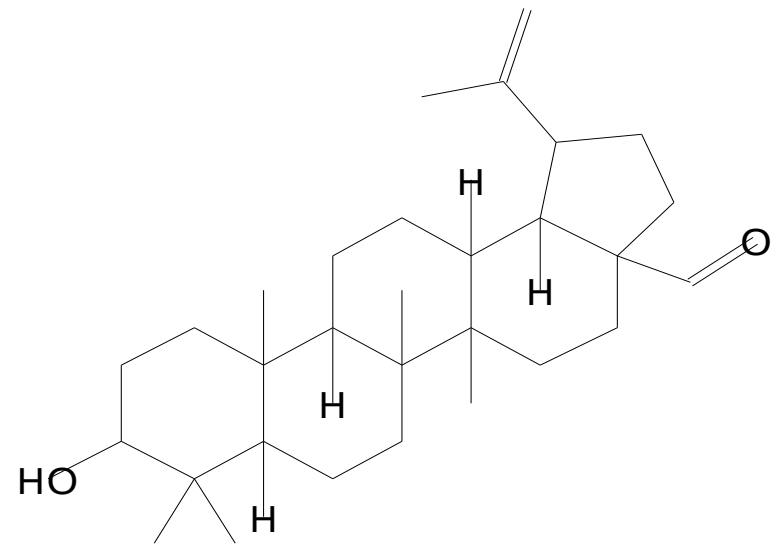

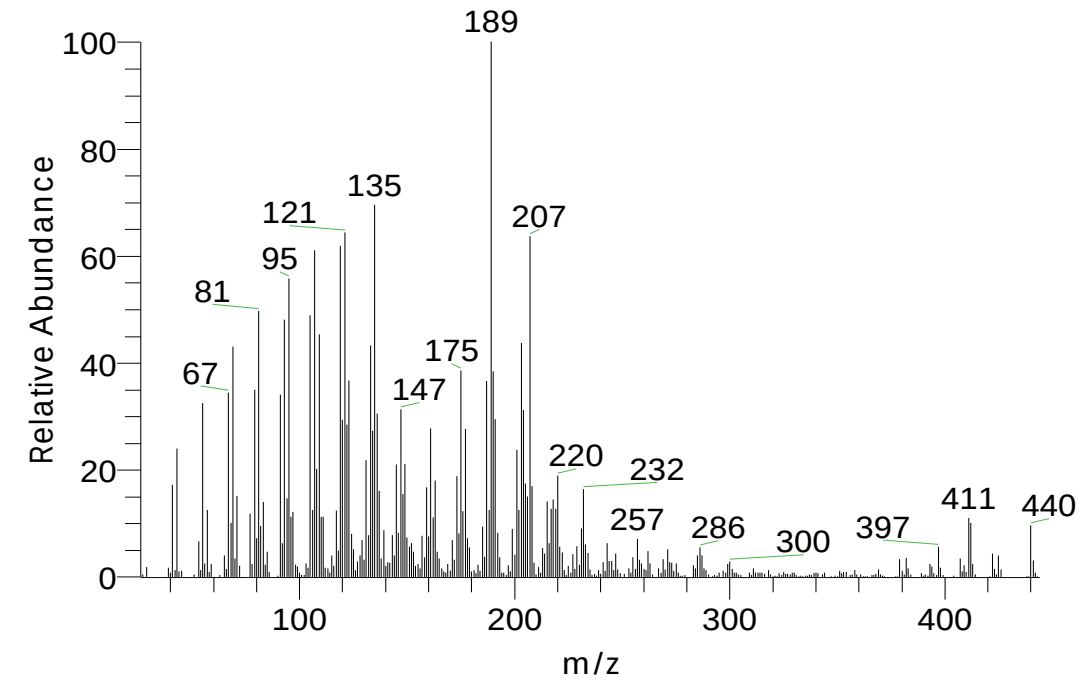

Oil_DrHafsa #9976 RT: 37.45 AV: 1 NL: 8.56E5
T: + c EI Full ms [50.000-750.000]

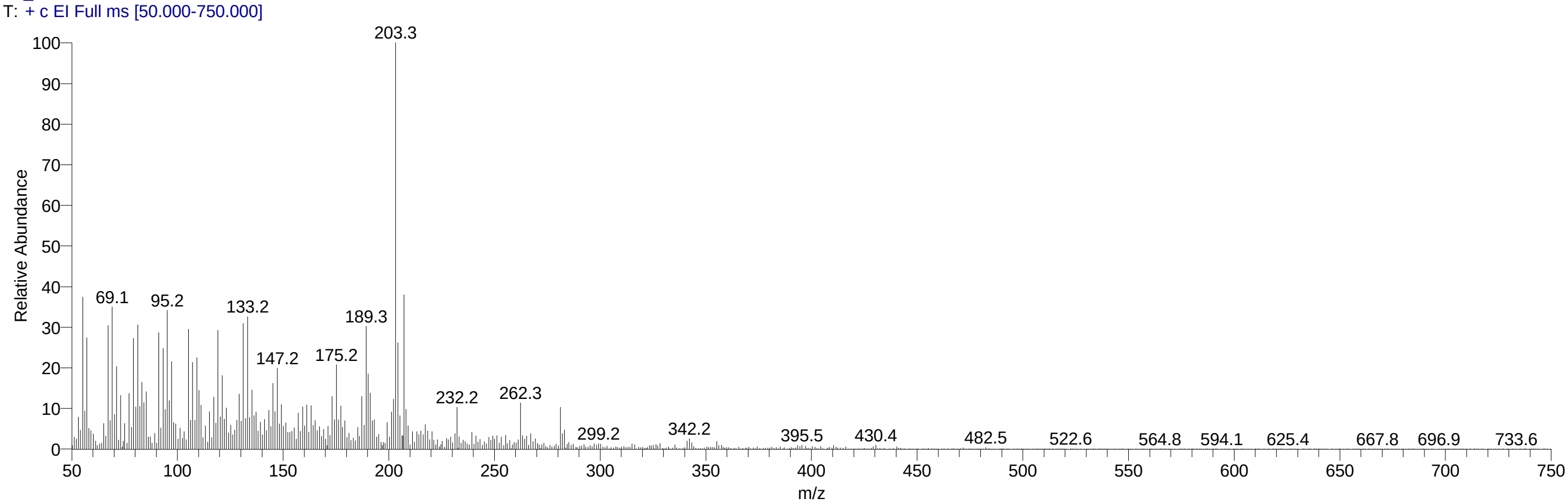

RT	Compound Name	Area %	MF	Molecular Formula	Molecular Weight	Cas #	Library
37.45	Betulin	0.28	771	C30H50O2	442	473-98-3	replib
37.45	Urs-12-en-28-oic acid, 3-hydroxy-, methyl ester, (3á)-	0.28	820	C31H50O3	470	32208-45-0	mainlib
37.45	URS-12-EN-28-OIC ACID, 3-HYDROXY-, METHYL ESTER, (3á)-	0.28	816	C31H50O3	470	32208-45-0	WileyRegistry8e
37.45	Betulinaldehyde	0.28	766	C30H48O2	440	13159-28-9	mainlib
37.45	LUP-20(29)-ENE-3,28-DIOL, (3á)-	0.28	807	C30H50O2	442	473-98-3	WileyRegistry8e

Compound Structure | Hit Spectrum

Betulin
Formula C30H50O2, MW 442, CAS# 473-98-3, Entry# 27738
Lup-20(29)-ene-3,28-diol, (3á)-

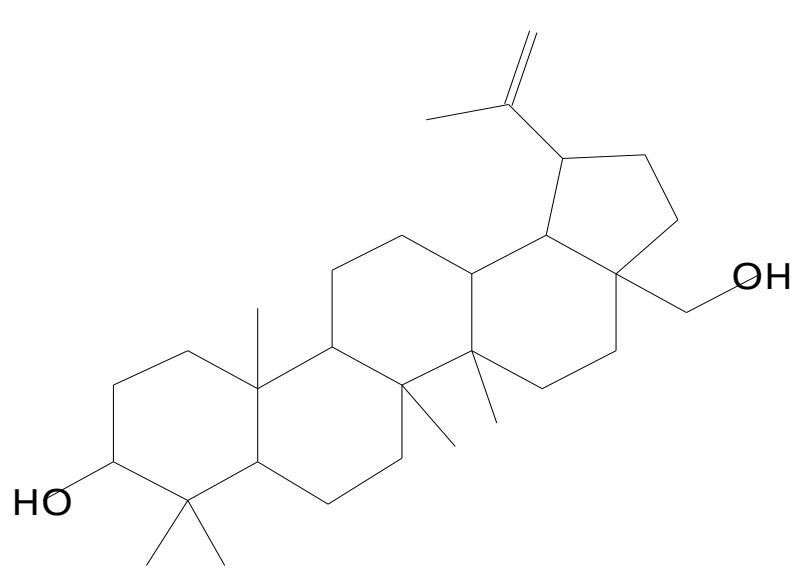

SI 768, RSI 771, replib, Entry# 27738, CAS# 473-98-3, Betulin

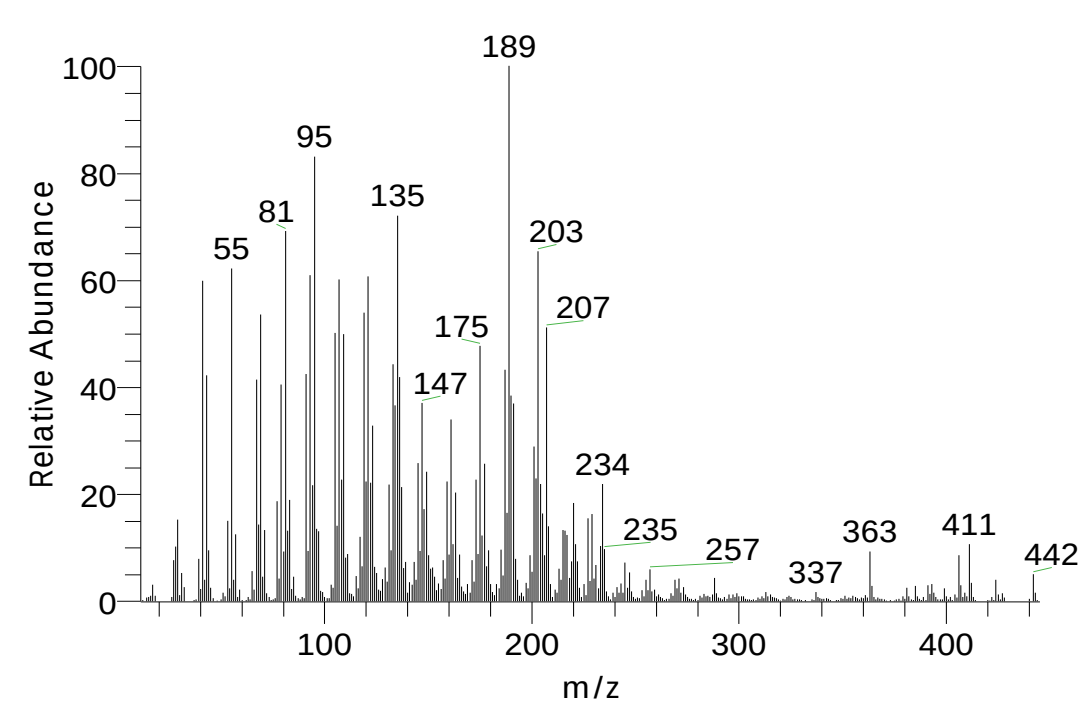

My GC-MS Report

Compound Structure | Hit Spectrum

Urs-12-en-28-oic acid, 3-hydroxy-, methyl ester, (3á)-
Formula C31H50O3, MW 470, CAS# 32208-45-0, Entry# 214160
Urs-12-en-28-oic acid, 3á-hydroxy-, methyl ester

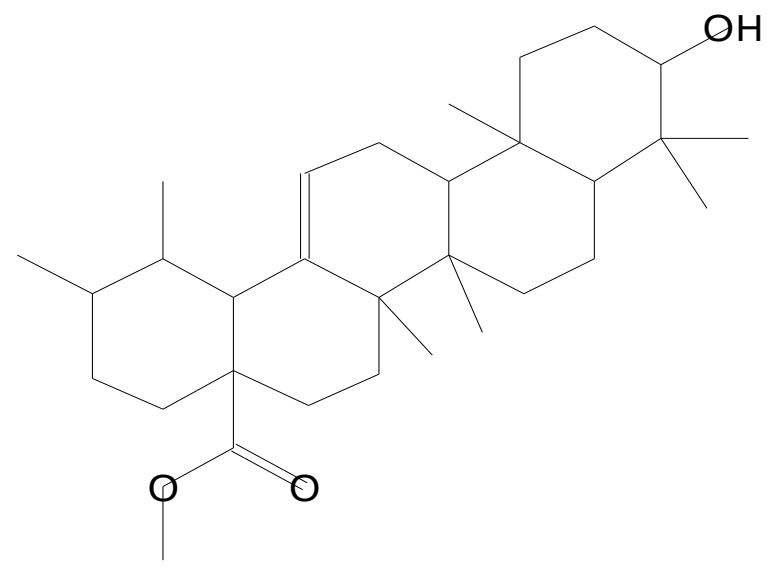

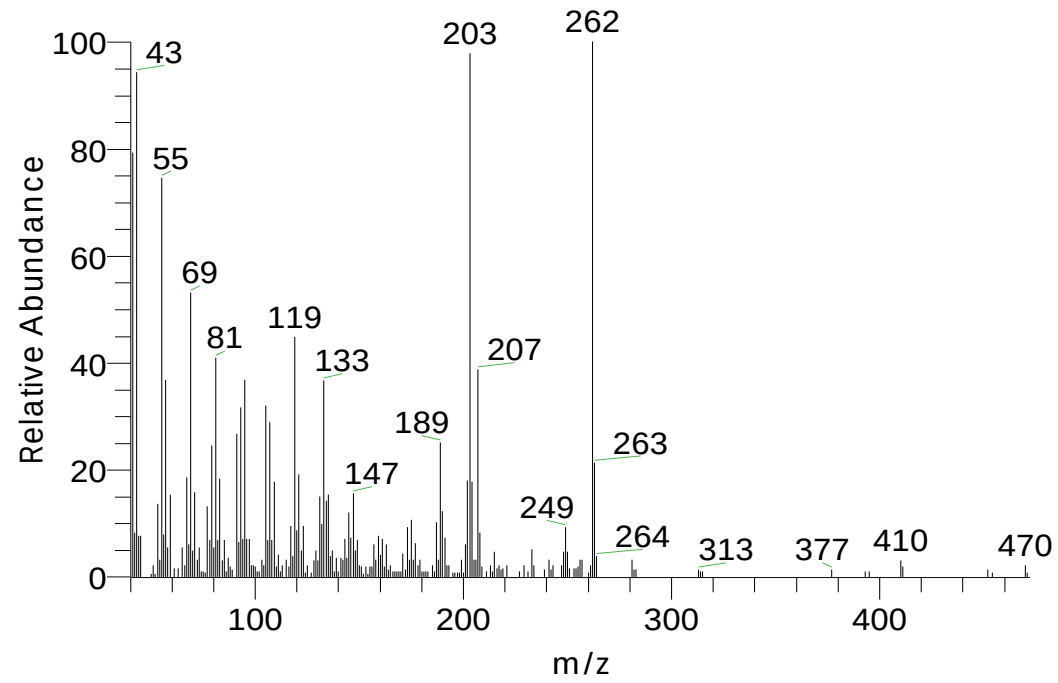

URS-12-EN-28-OIC ACID, 3-HYDROXY-, METHYL ESTER, (3á)-
Formula C31H50O3, MW 470, CAS# 32208-45-0, Entry# 279567
METHYL 3-HYDROXYURS-12-EN-28-OATE #

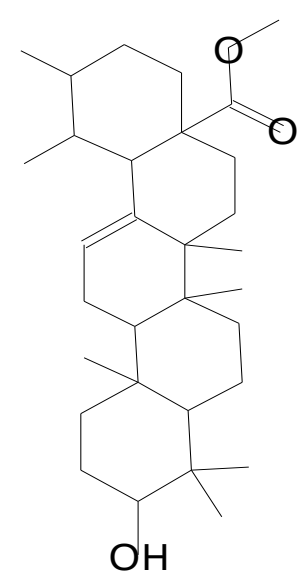

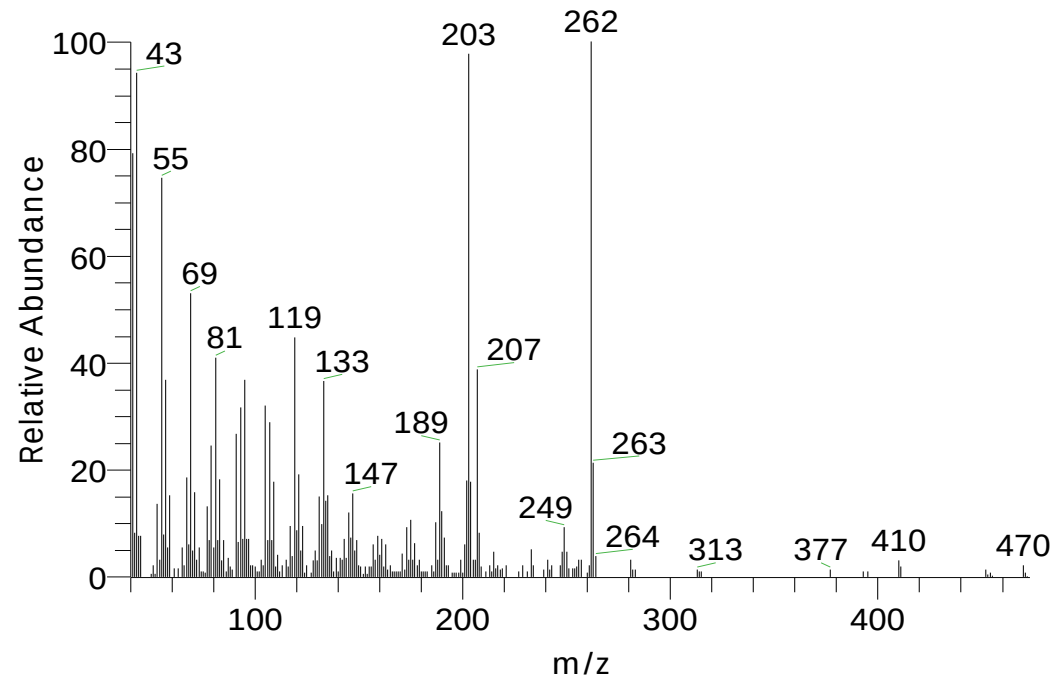

Betulinaldehyde
Formula C30H48O2, MW 440, CAS# 13159-28-9, Entry# 175929
$:28FELCJAPFJOPHSD-UHFFFAOYSA-N

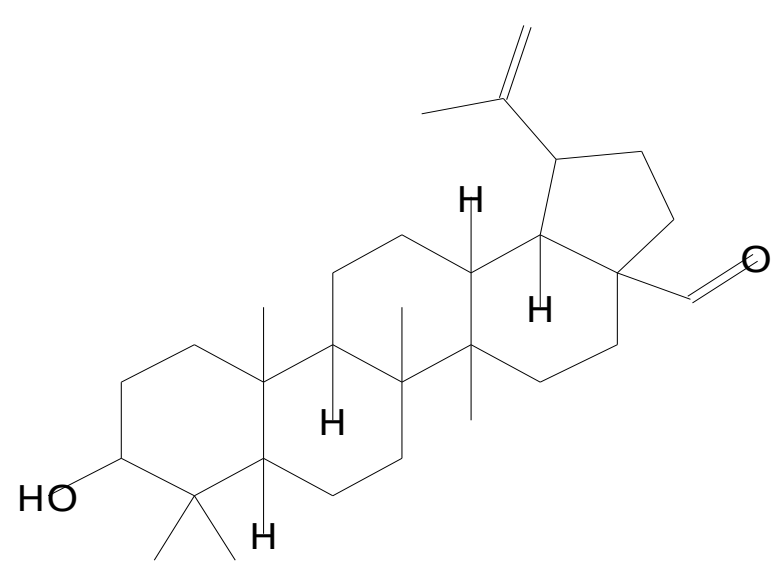

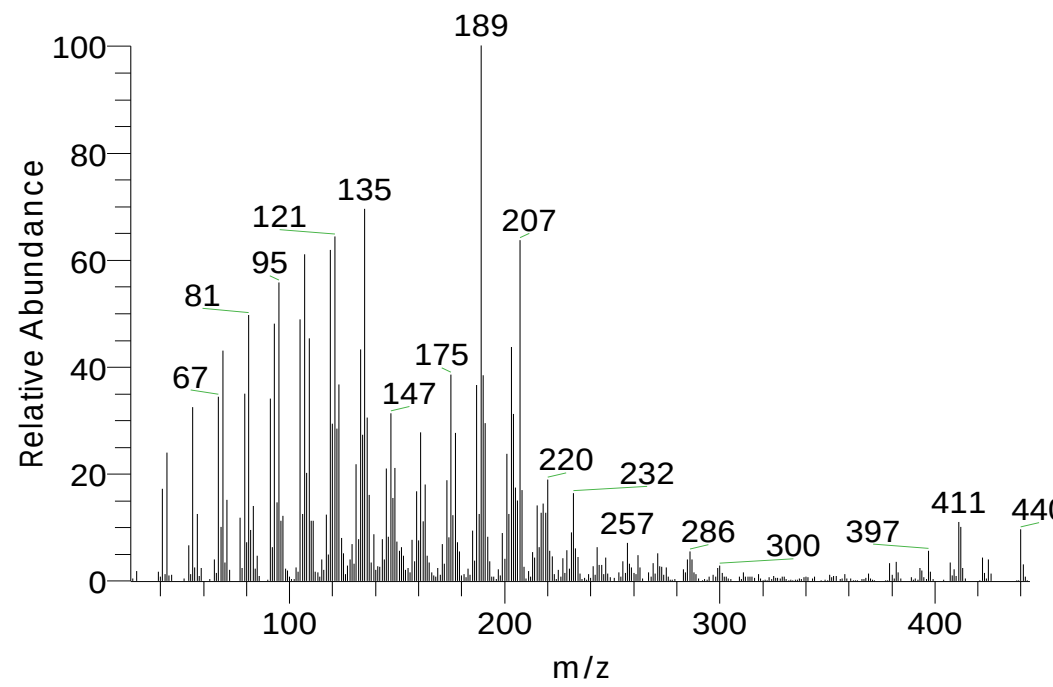

LUP-20(29)-ENE-3,28-DIOL, (3á)-
Formula C30H50O2, MW 442, CAS# 473-98-3, Entry# 349524
LUP-20(29)-ENE-3,28-DIOL #

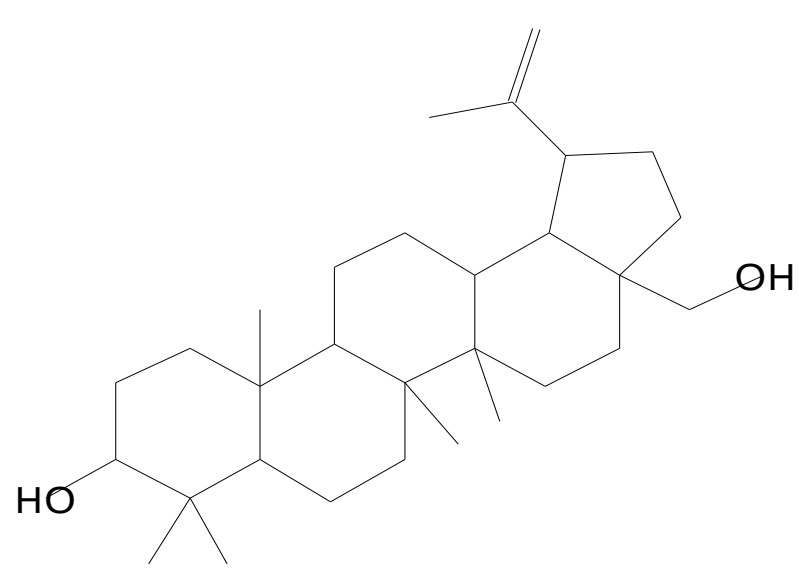

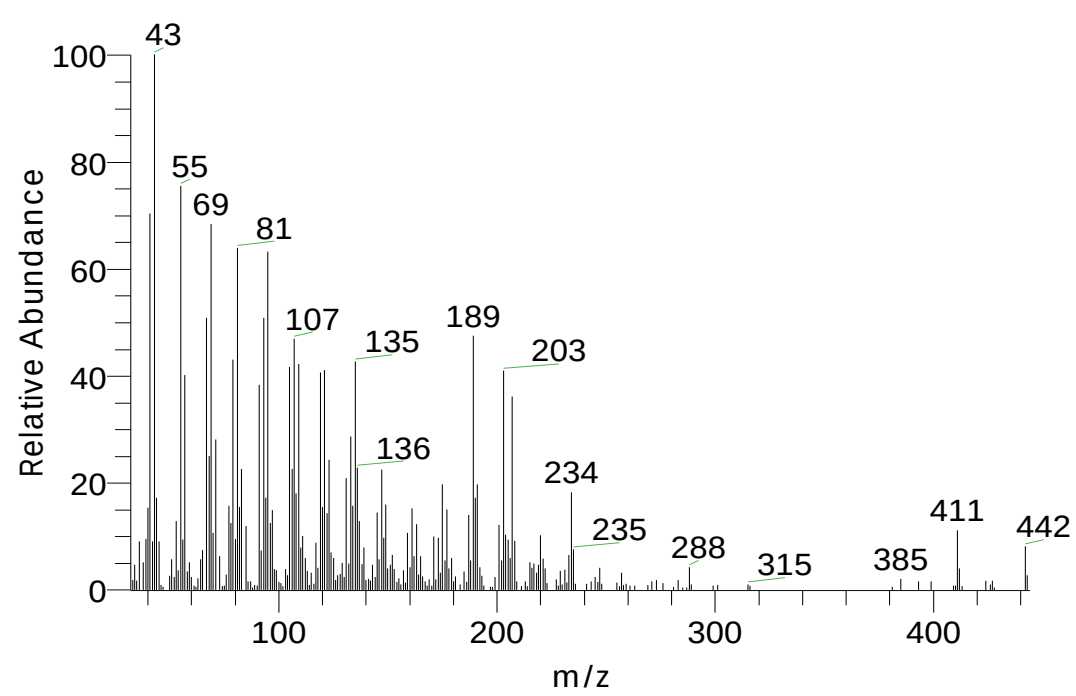

My GC-MS Report

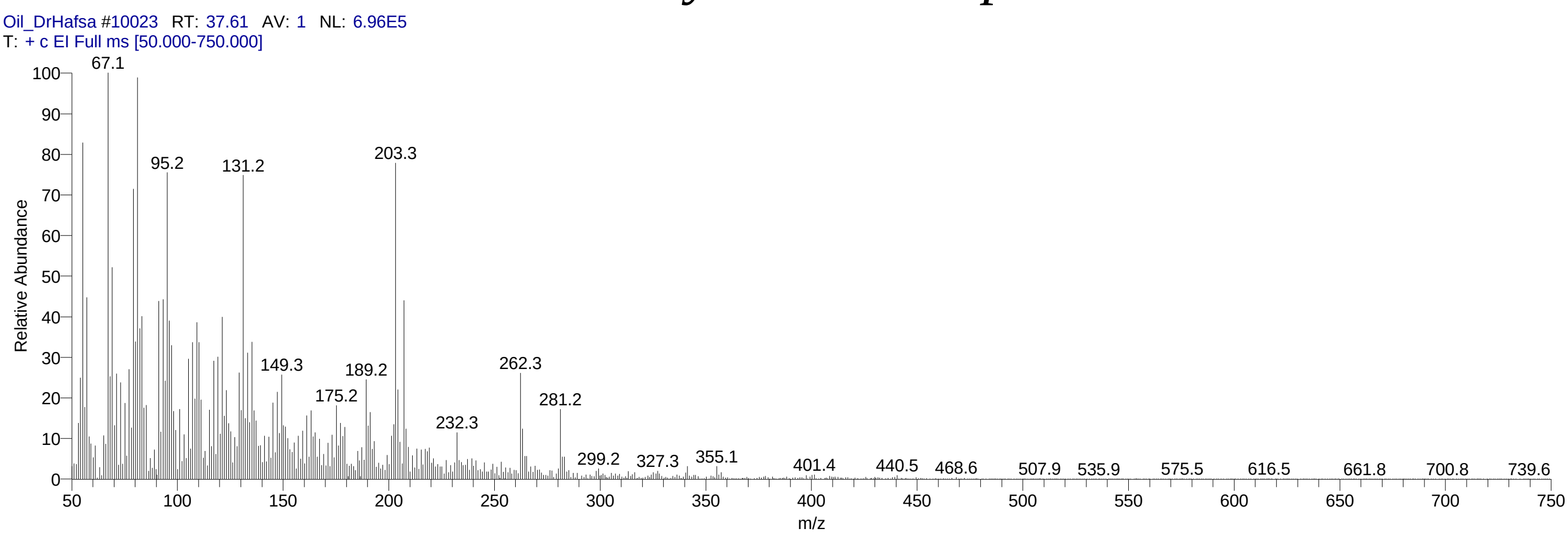

RT	Compound Name	Area %	MF	Molecular Formula	Molecular Weight	Cas #	Library
37.61	Urs-12-en-28-oic acid, 3-hydroxy-, methyl ester, (3á)-	0.29	797	C31H50O3	470	32208-45-0	mainlib
37.61	URS-12-EN-28-OIC ACID, 3-HYDROXY-, METHYL ESTER, (3á)-	0.29	796	C31H50O3	470	32208-45-0	WileyRegistry8e
37.61	9,12,15-OCTADECATRIENOIC ACID, 2,3-BIS[(TRIMETHYLSILYL)OXY]PROPYL ESTER, (Z,Z,Z)-	0.29	767	C27H52O4Si2	496	55521-22-7	WileyRegistry8e
37.61	LUP-20(29)-ENE-3,28-DIOL, (3á)-	0.29	797	C30H50O2	442	473-98-3	WileyRegistry8e
37.61	PROPANOIC ACID, 2-METHYL-, (DECAHYDRO-6A-HYDROXY-9A-METHYL-3-METHYLENE-2,9-DIOXOAZULENO[4,5-B]FURAN-6-YL)METHYL ESTER, [3AS-(3Aà,6á,6Aà,9Aá,9Bà)]-	0.29	774	C19H26O6	350	33649-17-1	WileyRegistry8e

Compound Structure	Hit Spectrum

Urs-12-en-28-oic acid, 3-hydroxy-, methyl ester, (3á)-
Formula C31H50O3, MW 470, CAS# 32208-45-0, Entry# 214160
Urs-12-en-28-oic acid, 3á-hydroxy-, methyl ester

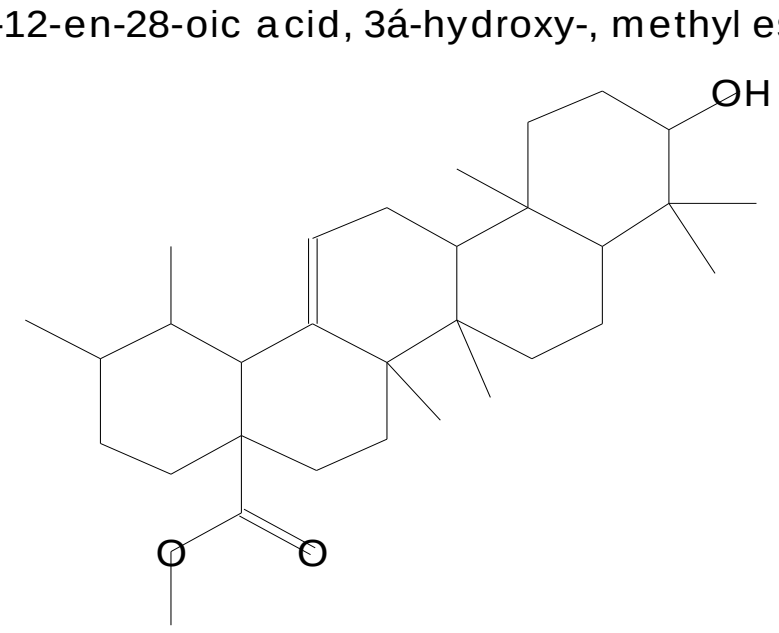

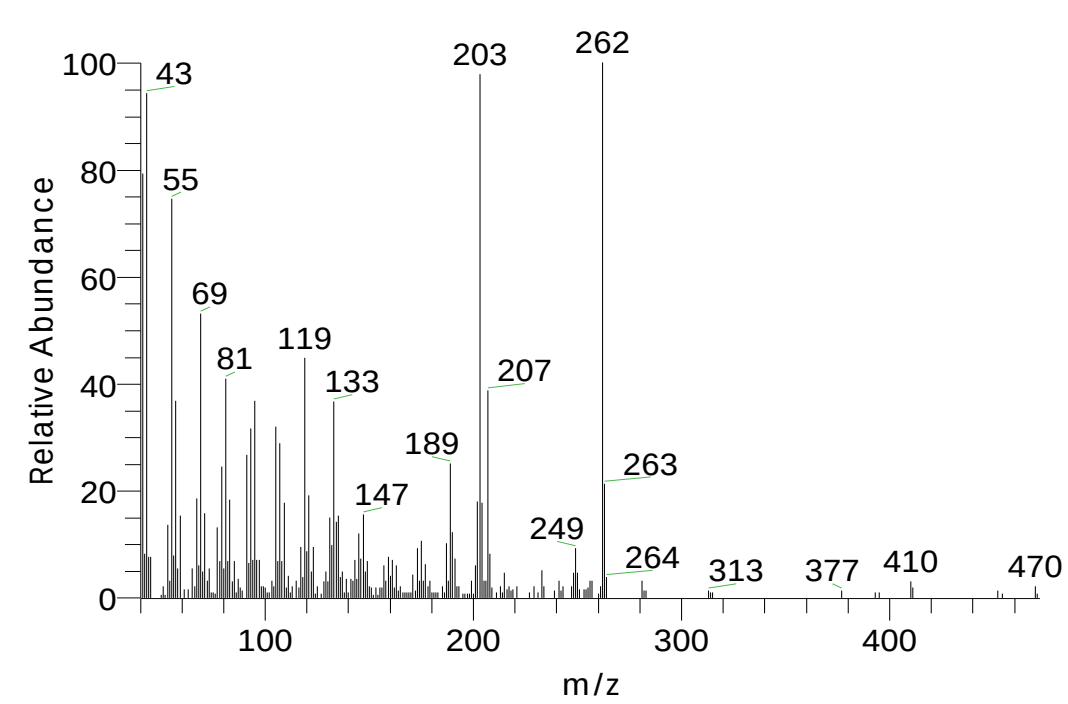

URS-12-EN-28-OIC ACID, 3-HYDROXY-, METHYL ESTER, (3á)-
Formula C31H50O3, MW 470, CAS# 32208-45-0, Entry# 279567
METHYL 3-HYDROXYURS-12-EN-28-OATE #

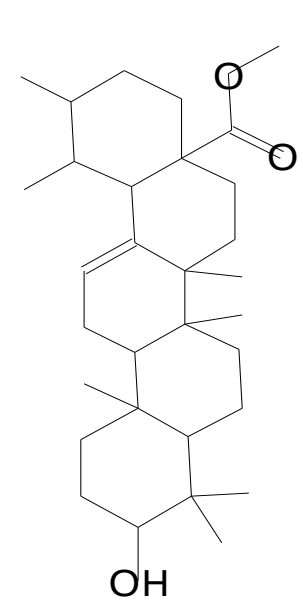

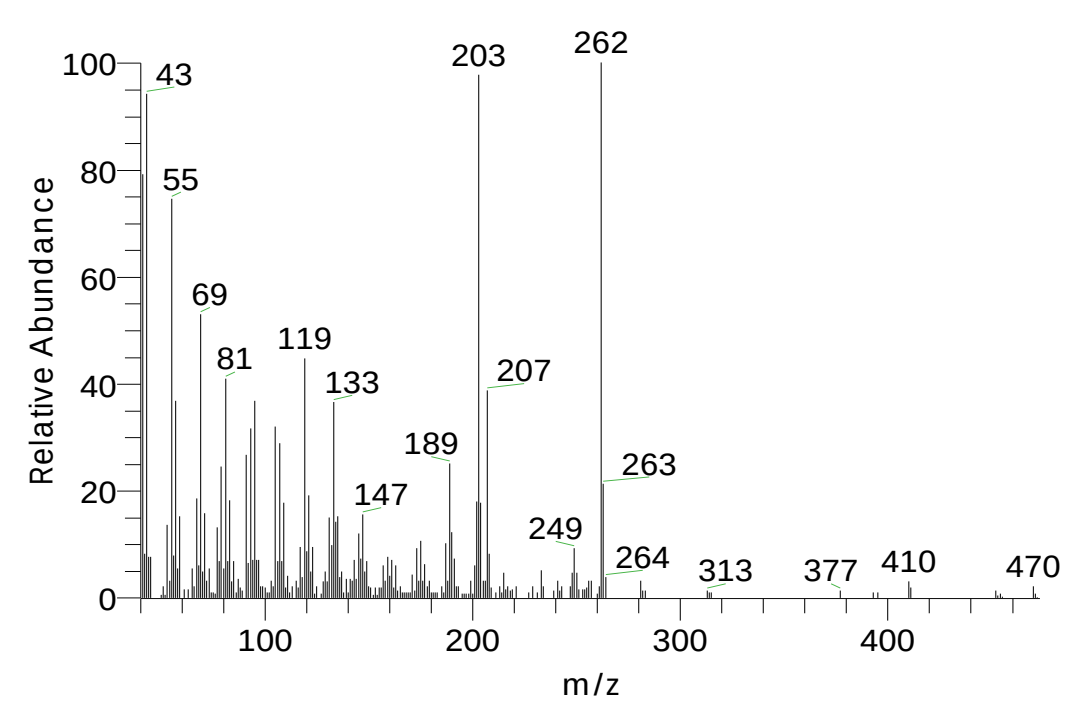

My GC-MS Report

Compound Structure | Hit Spectrum

9,12,15-OCTADECATRIENOIC ACID, 2,3-BIS[(TRIMETHYLSILYL)OXY]PROPYL ESTER, (Z,Z,Z)-
Formula C27H52O4Si2, MW 496, CAS# 55521-22-7, Entry# 284834
2,3-BIS[(TRIMETHYLSILYL)OXY]PROPYL (9E,12E,15E)-9,12,15-OCTADECATRIENOATE #

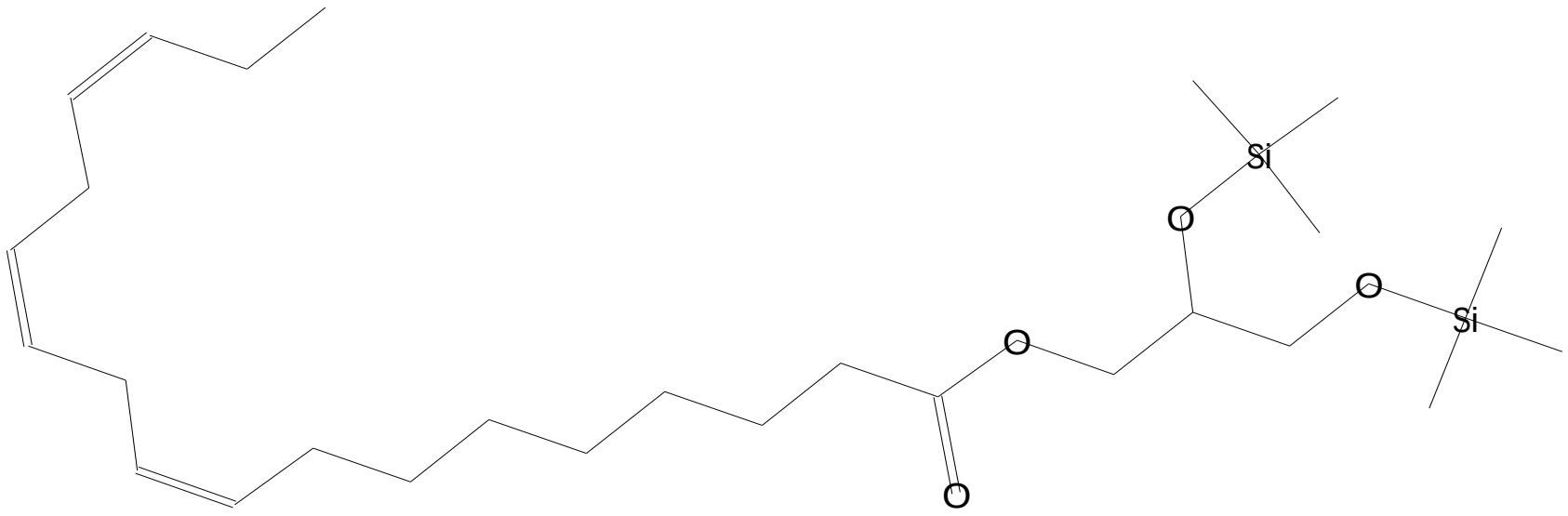

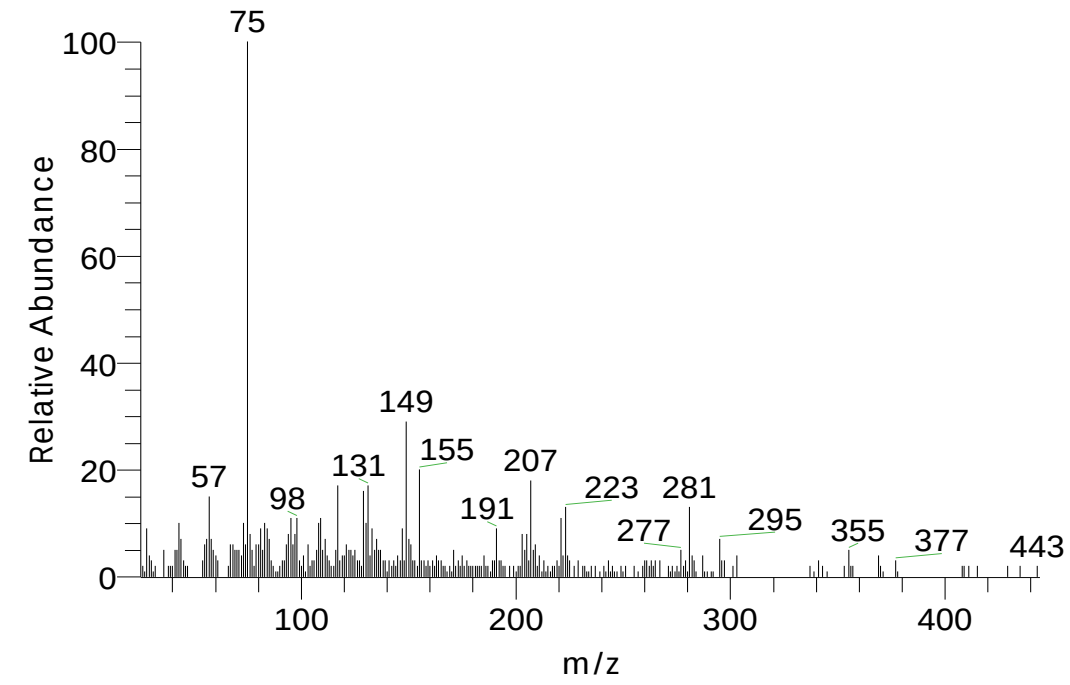

LUP-20(29)-ENE-3,28-DIOL, (3á)-
Formula C30H50O2, MW 442, CAS# 473-98-3, Entry# 349524
LUP-20(29)-ENE-3,28-DIOL #

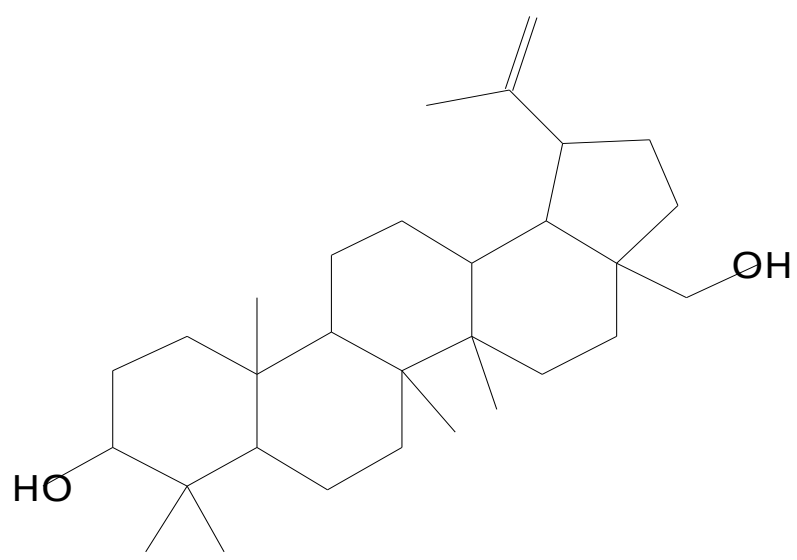

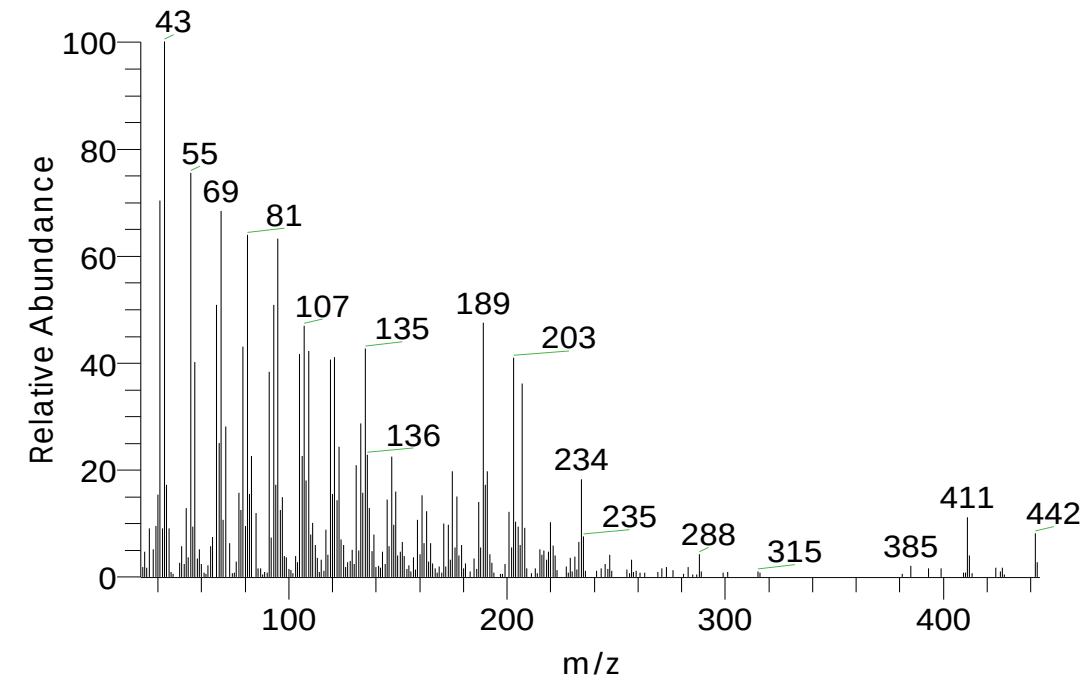

Formula C19H26O6, MW 350, CAS# 33649-17-1, Entry# 228497

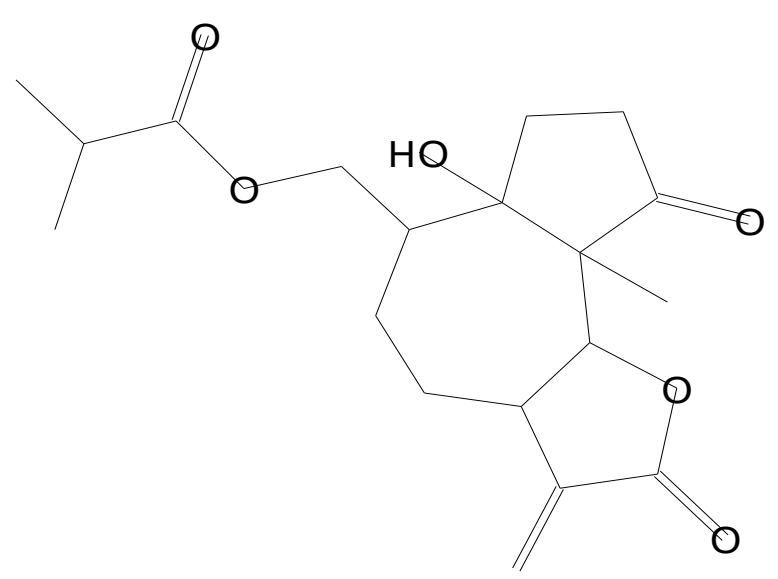

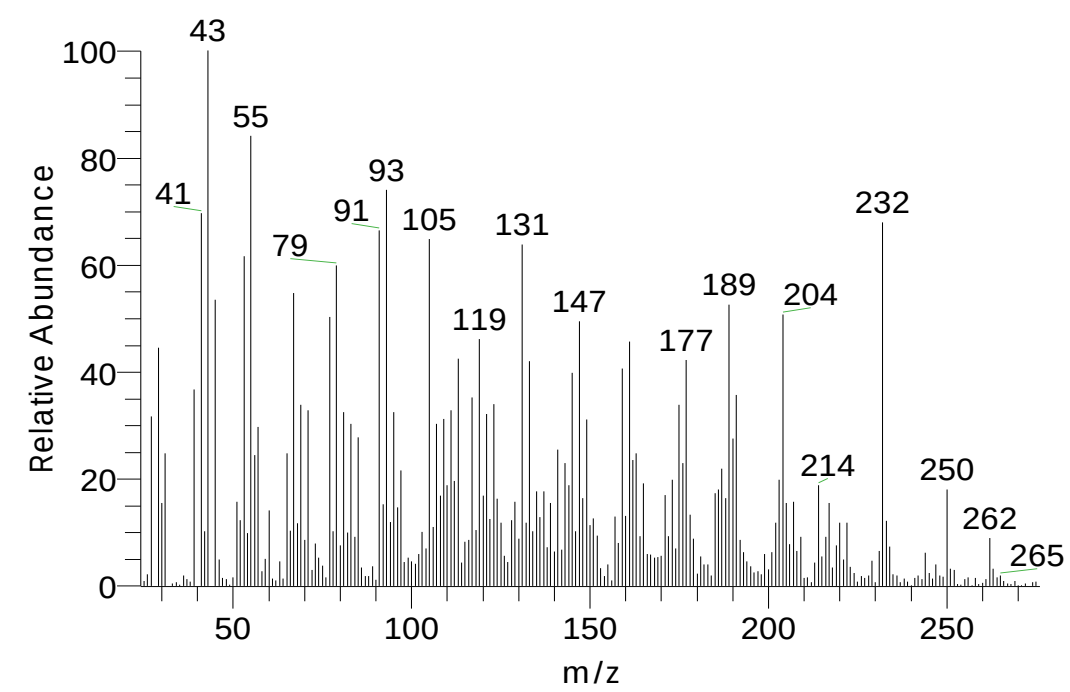

Oil_DrHafsa #10061 RT: 37.74 AV: 1 NL: 1.69E6
T: + c EI Full ms [50.000-750.000]

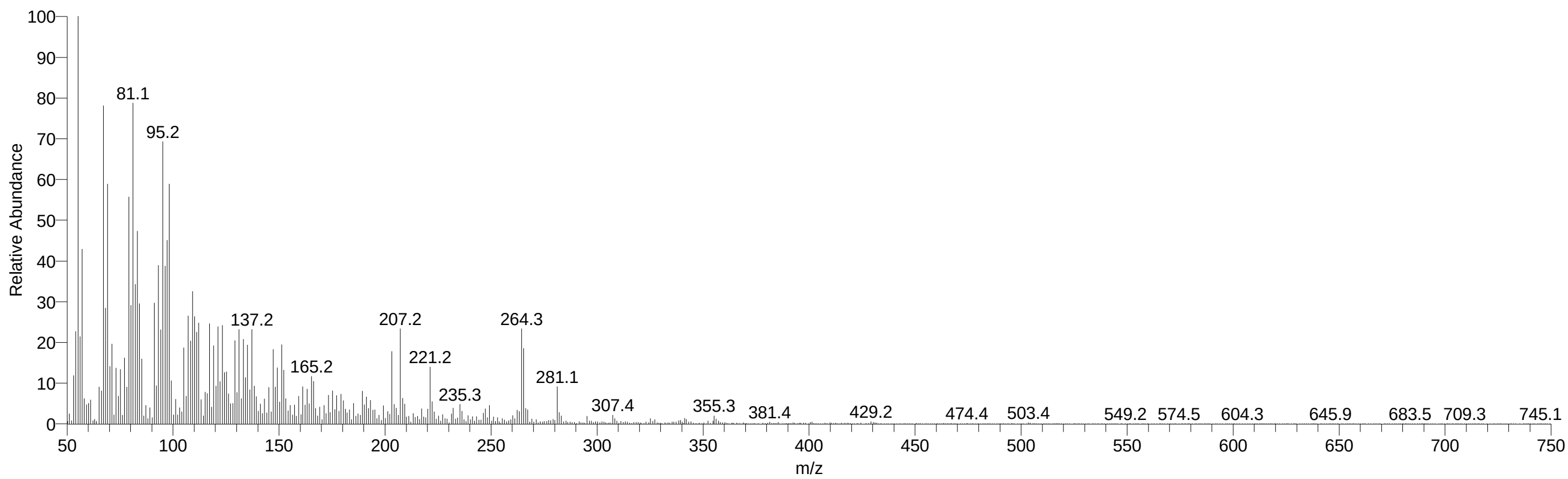

RT	Compound Name	Area %	MF	Molecular Formula	Molecular Weight	Cas #	Library
37.74	9-Octadecenoic acid (Z)-, 2-hydroxy-1-(hydroxymethyl)ethyl ester	4.91	836	C21H40O4	356	3443-84-3	mainlib

My GC-MS Report

RT	Compound Name	Area %	MF	Molecular Formula	Molecular Weight	Cas #	Library
37.74	9-OCTADECENOIC ACID (Z)-, 2-HYDROXY-1-(HYDROXYMETHYL)ETHYL ESTER	4.91	830	C21H40O4	356	3443-84-3	WileyRegistry8e
37.74	Glycidyl oleate	4.91	819	C21H38O3	338	NA	mainlib
37.74	2-HYDROXY-3-[(9E)-9-OCTADECENOYLOXY]PROPYL (9E)-9-OCTADECENOATE #	4.91	787	C39H72O5	620	2465-32-9	WileyRegistry8e
37.74	2,3-Dihydroxypropyl elaidate	4.91	785	C21H40O4	356	2716-53-2	mainlib

Compound Structure | Hit Spectrum

9-Octadecenoic acid (Z)-, 2-hydroxy-1-(hydroxymethyl)ethyl ester
Formula C21H40O4, MW 356, CAS# 3443-84-3, Entry# 19276
Olein, 2-mono-

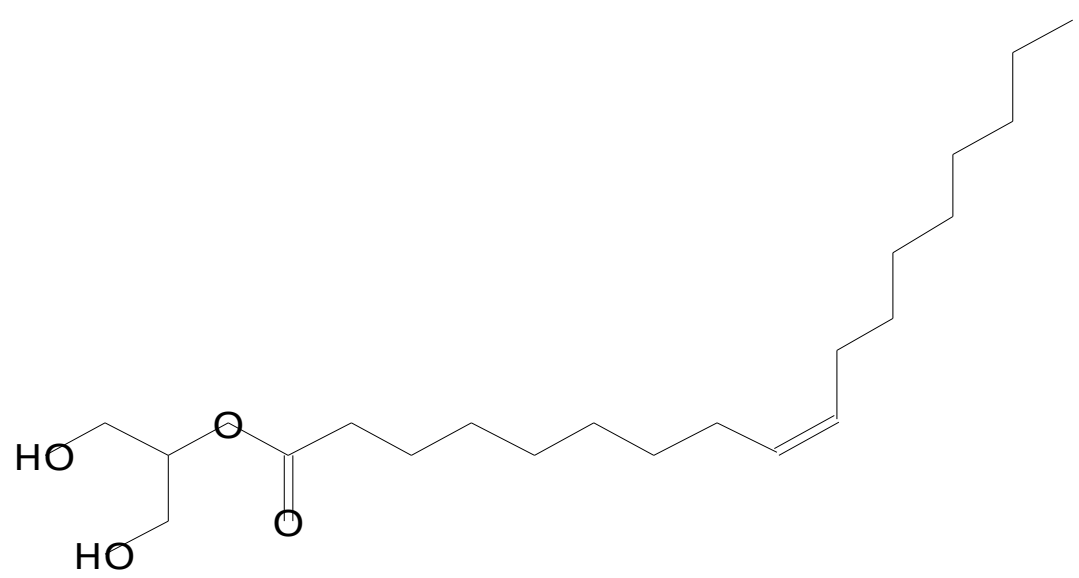

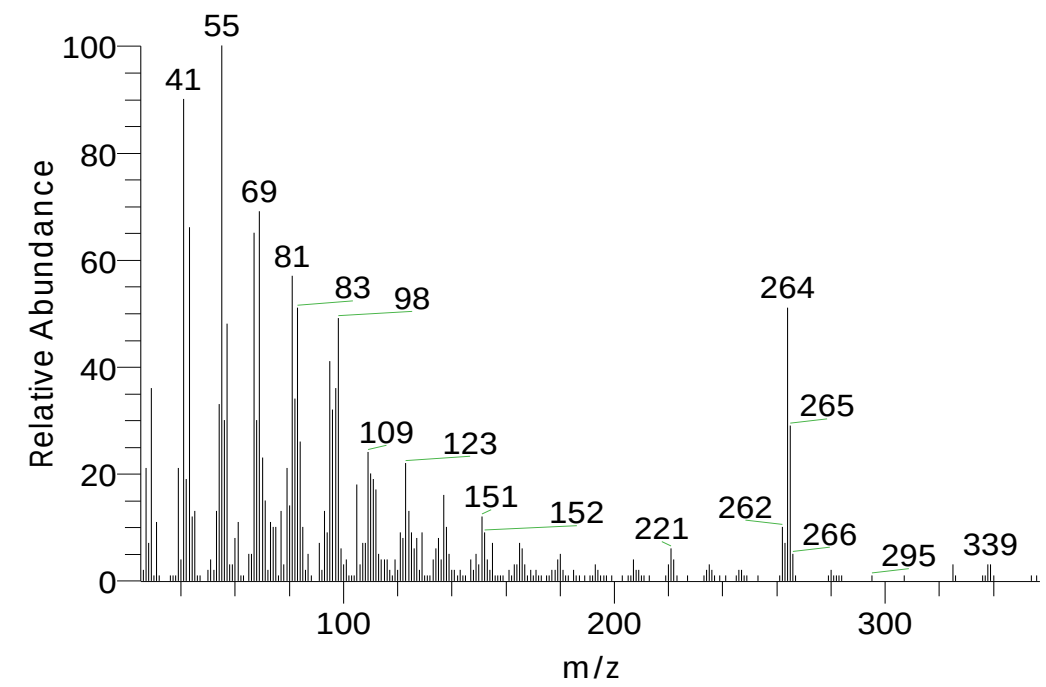

9-OCTADECENOIC ACID (Z)-, 2-HYDROXY-1-(HYDROXYMETHYL)ETHYL ESTER
Formula C21H40O4, MW 356, CAS# 3443-84-3, Entry# 232585
2-HYDROXY-1-(HYDROXYMETHYL)ETHYL (9Z)-9-OCTADECENOATE #

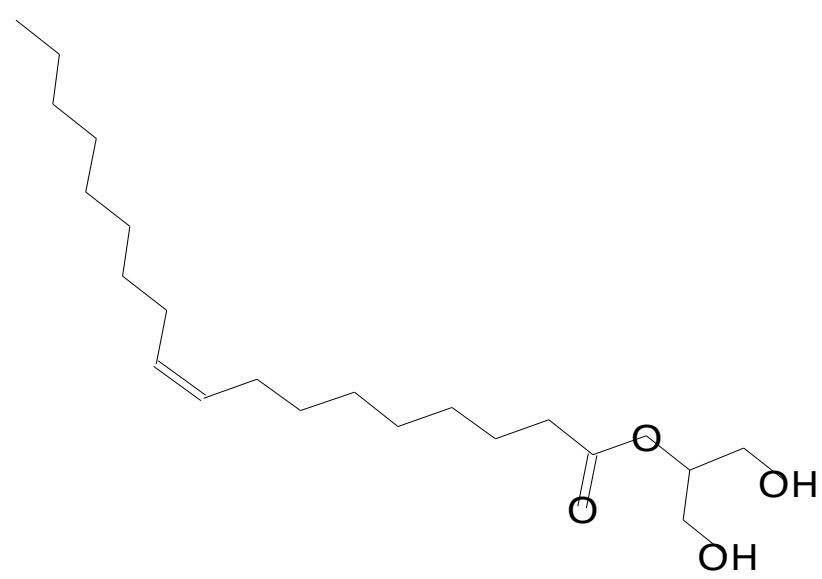

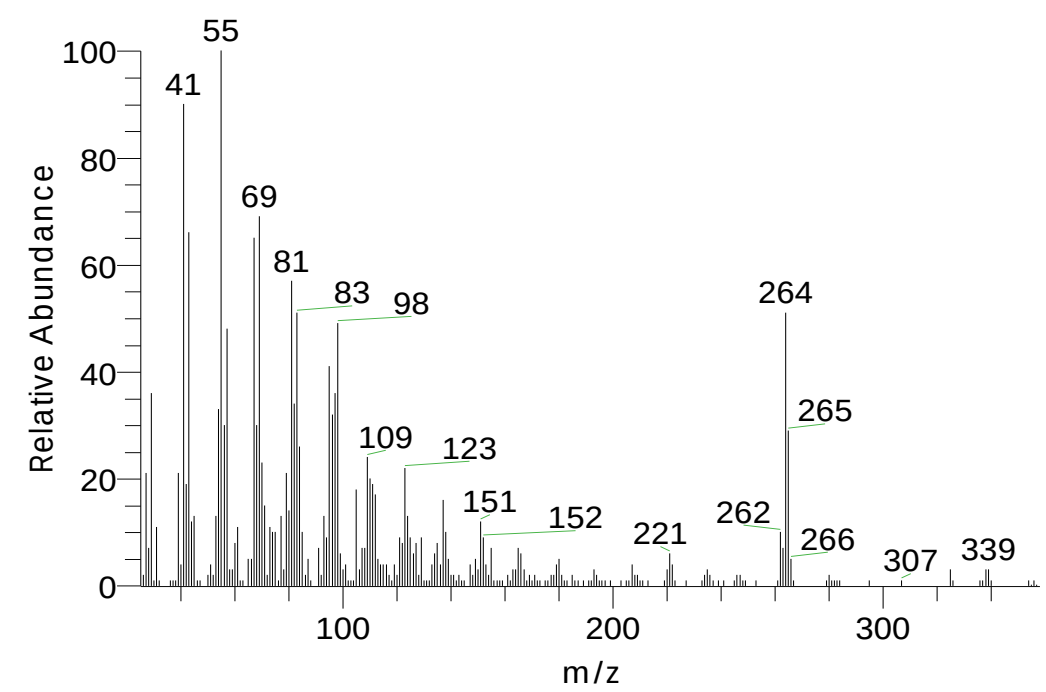

Glycidyl oleate
Formula C21H38O3, MW 338, CAS# NA, Entry# 112970
$:28VWYIWOYBERNXLX-KTKRTIGZSA-N

SI 776, RSI 819, mainlib, Entry# 112970, CAS# NA, Glycidyl oleate

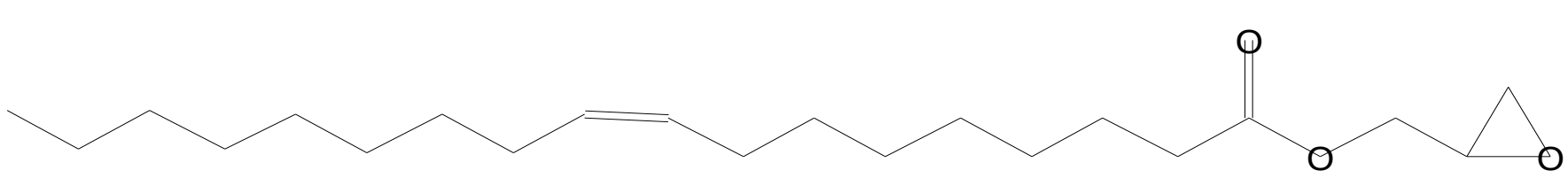

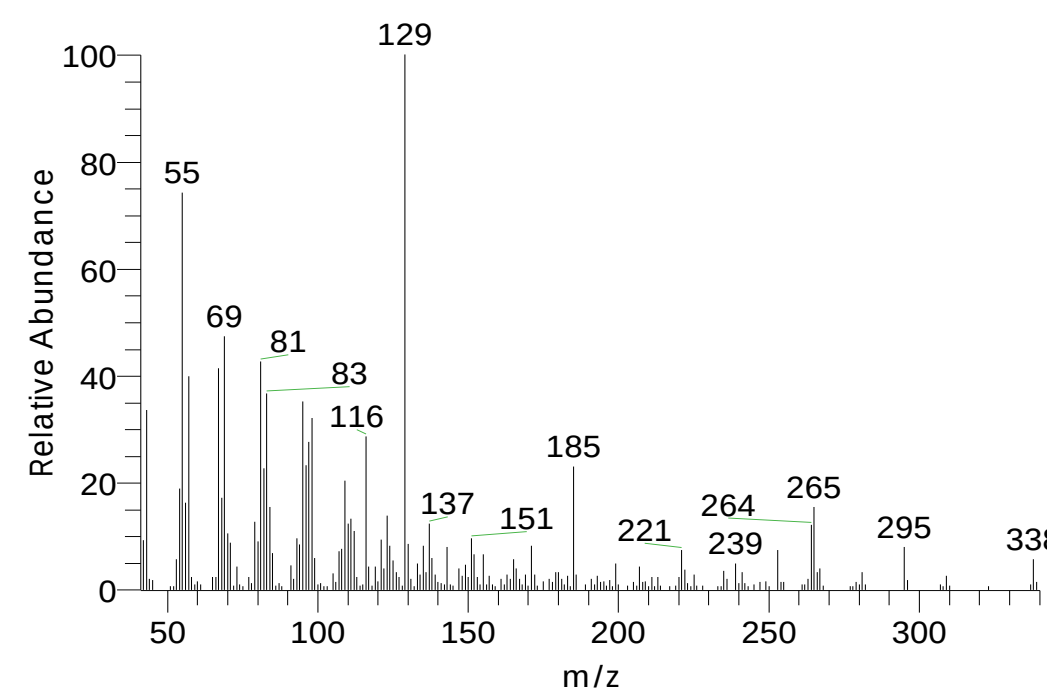

2-HYDROXY-3-[(9E)-9-OCTADECENOYLOXY]PROPYL (9E)-9-OCTADECENOATE #
Formula C39H72O5, MW 620, CAS# 2465-32-9, Entry# 298152
(Z,Z)-1,3-DIOCTADECENOYL GLYCEROL

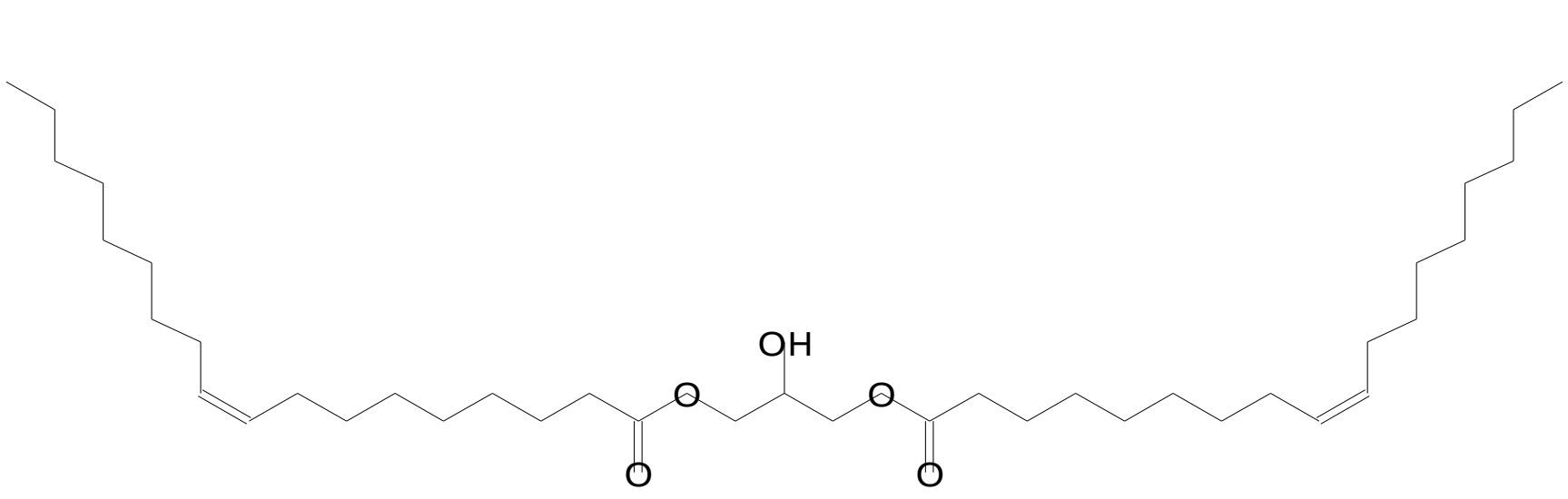

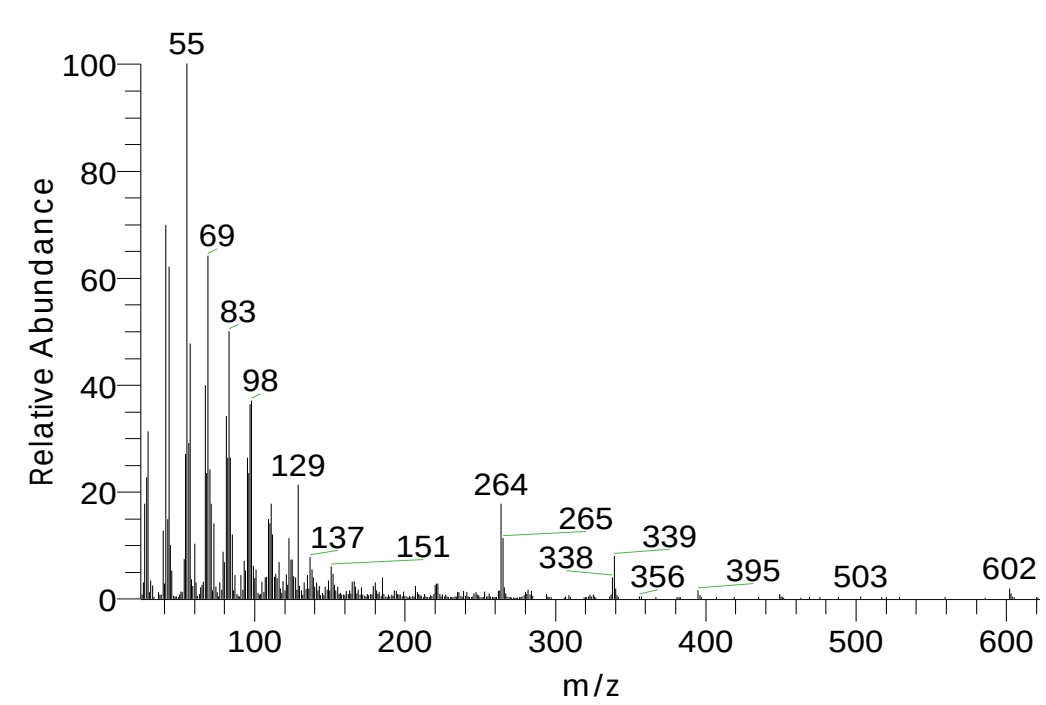

My GC-MS Report

Compound Structure | Hit Spectrum

2,3-Dihydroxypropyl elaidate
Formula C21H40O4, MW 356, CAS# 2716-53-2, Entry# 21817
2,3-Dihydroxypropyl (9E)-9-octadecenoate #

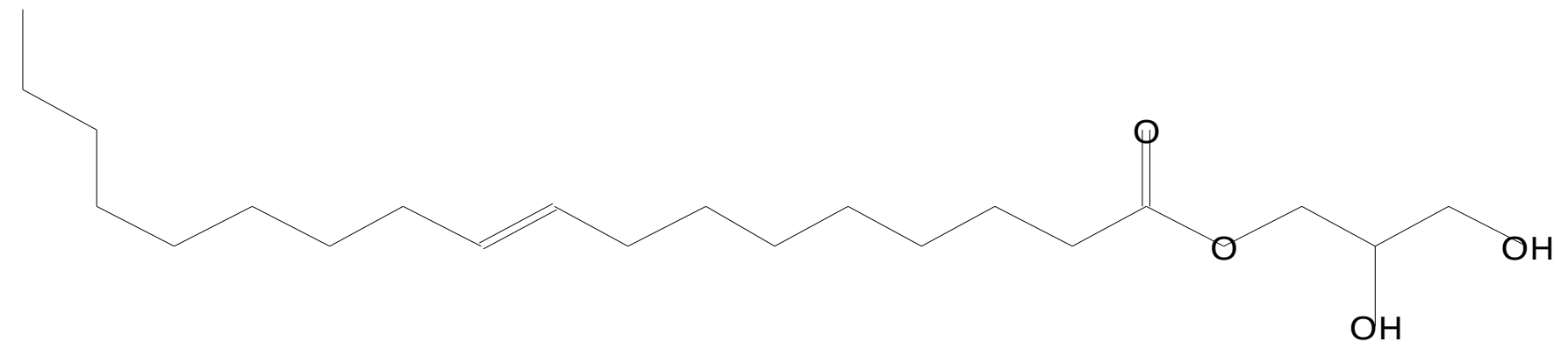

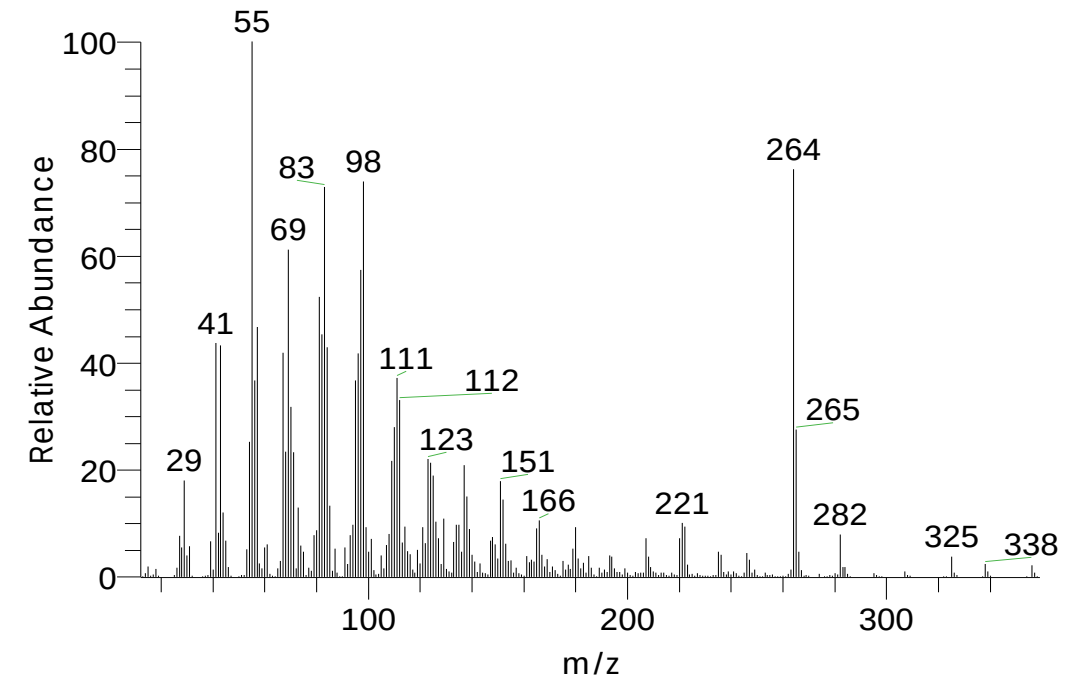

Oil_DrHafsa #10197 RT: 38.19 AV: 1 NL: 4.31E5
T: + c EI Full ms [50.000-750.000]

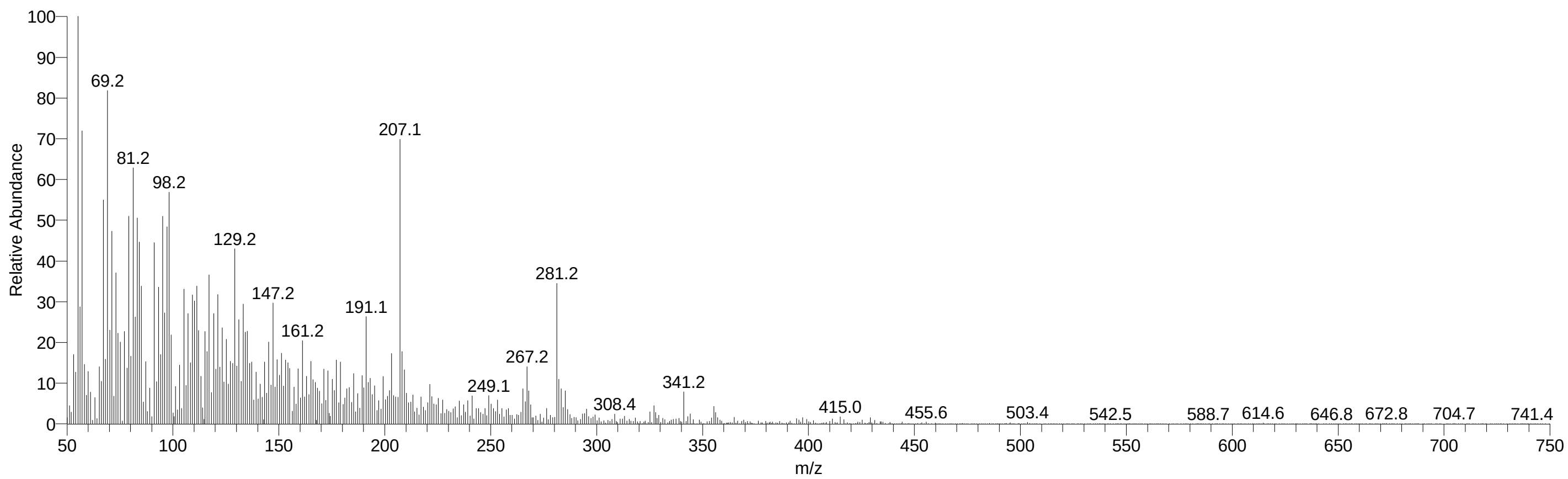

RT	Compound Name	Area %	MF	Molecular Formula	Molecular Weight	Cas #	Library
38.19	Ethyl iso-allocholate	0.43	796	C26H44O5	436	NA	mainlib
38.19	ETHYL ISO-ALLOCHOLATE	0.43	796	C26H44O5	436	NA	WileyRegistry8e
38.19	1-Heptatriacotanol	0.43	807	C37H76O	536	105794-58-9	mainlib
38.19	01297107001 TETRANEURIN - A - DIOL	0.43	804	C15H20O5	280	NA	WileyRegistry8e
38.19	03027205002 FLAVONE 4'-OH,5-OH,7-DI-O-GLUCOSIDE	0.43	722	C27H30O15	594	NA	WileyRegistry8e

Compound Structure | Hit Spectrum

Ethyl iso-allocholate
Formula C26H44O5, MW 436, CAS# NA, Entry# 7020
Ethyl 3,7,12-trihydroxycholan-24-oate #

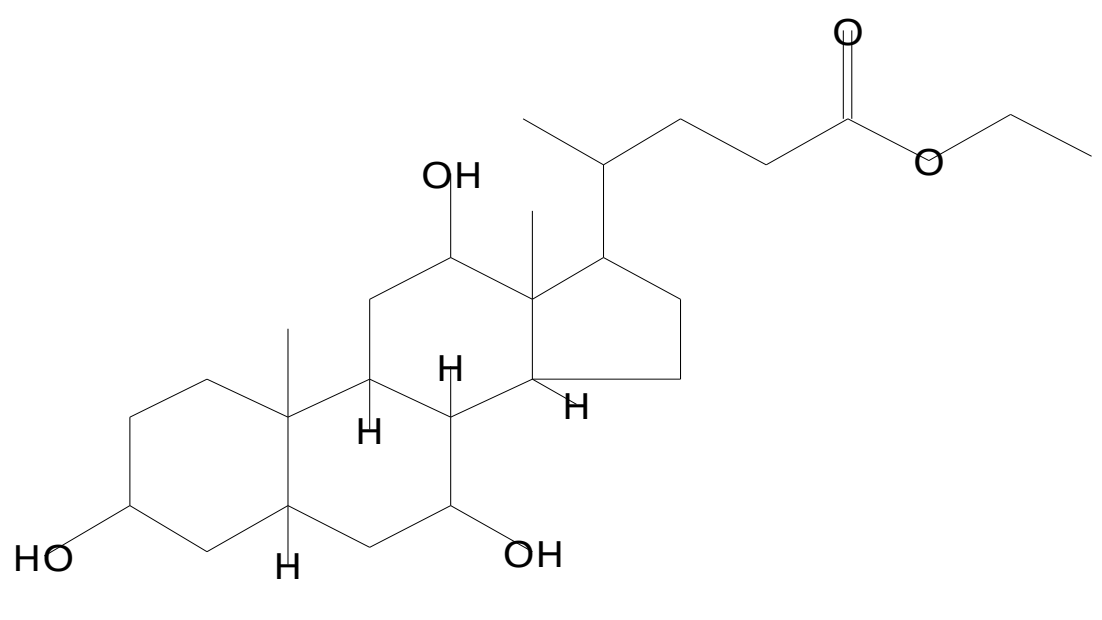

SI 786, RSI 796, mainlib, Entry# 7020, CAS# NA, Ethyl iso-allocholate

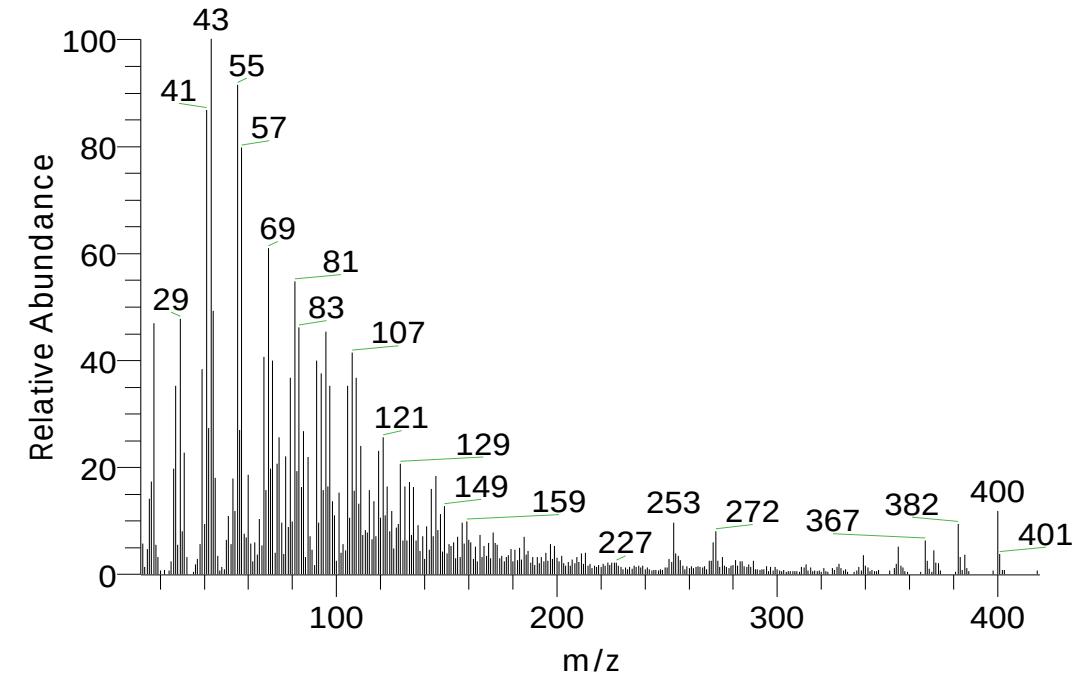

My GC-MS Report

Compound Structure | Hit Spectrum

ETHYL ISO-ALLOCHOLATE
Formula C26H44O5, MW 436, CAS# NA, Entry# 270212

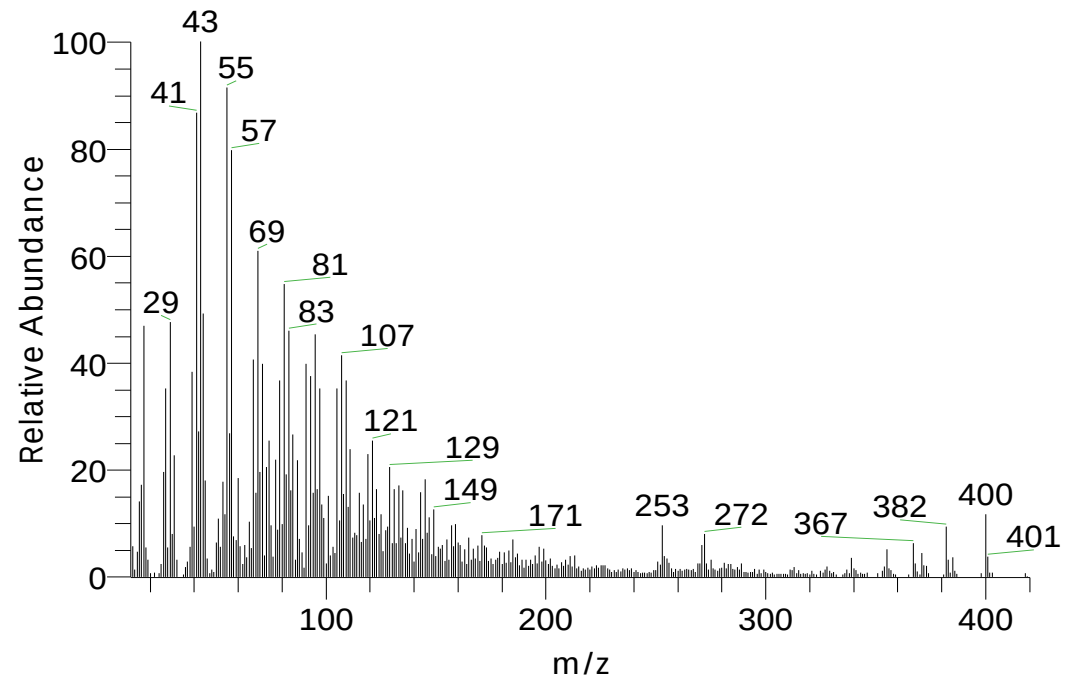

1-Heptatriacotanol
Formula C37H76O, MW 536, CAS# 105794-58-9, Entry# 7279
1-Heptatriacontanol #

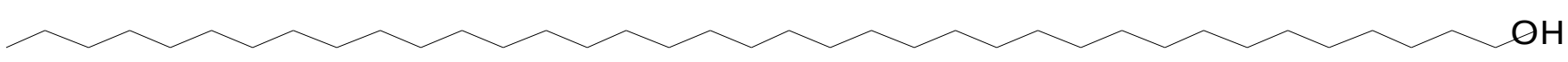

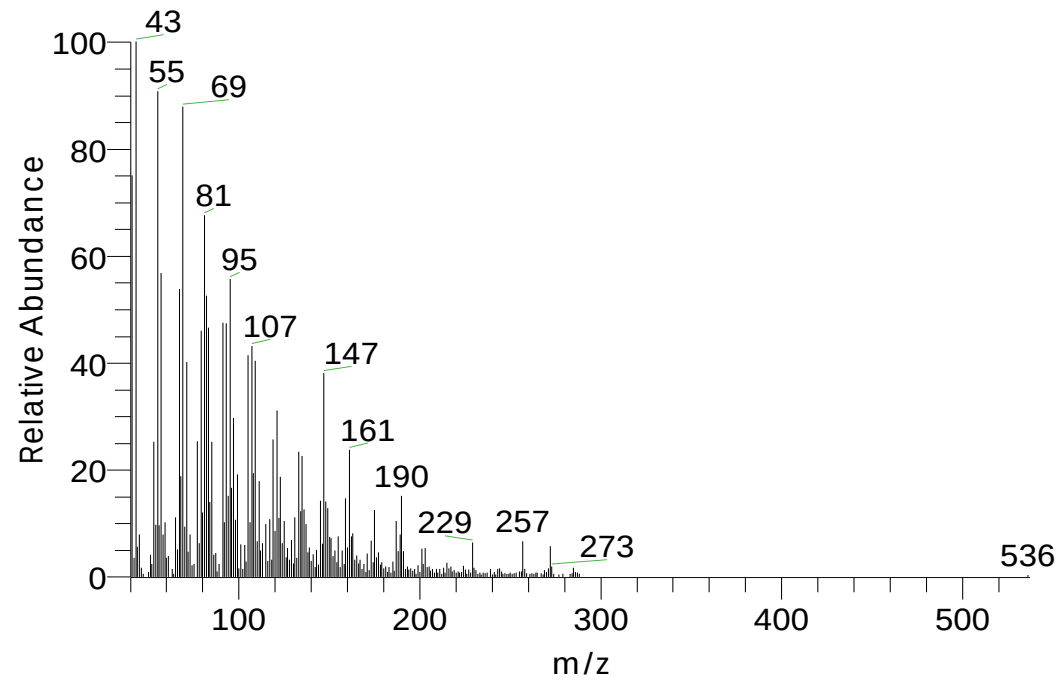

01297107001 TETRANEURIN - A - DIOL
Formula C15H20O5, MW 280, CAS# NA, Entry# 170378

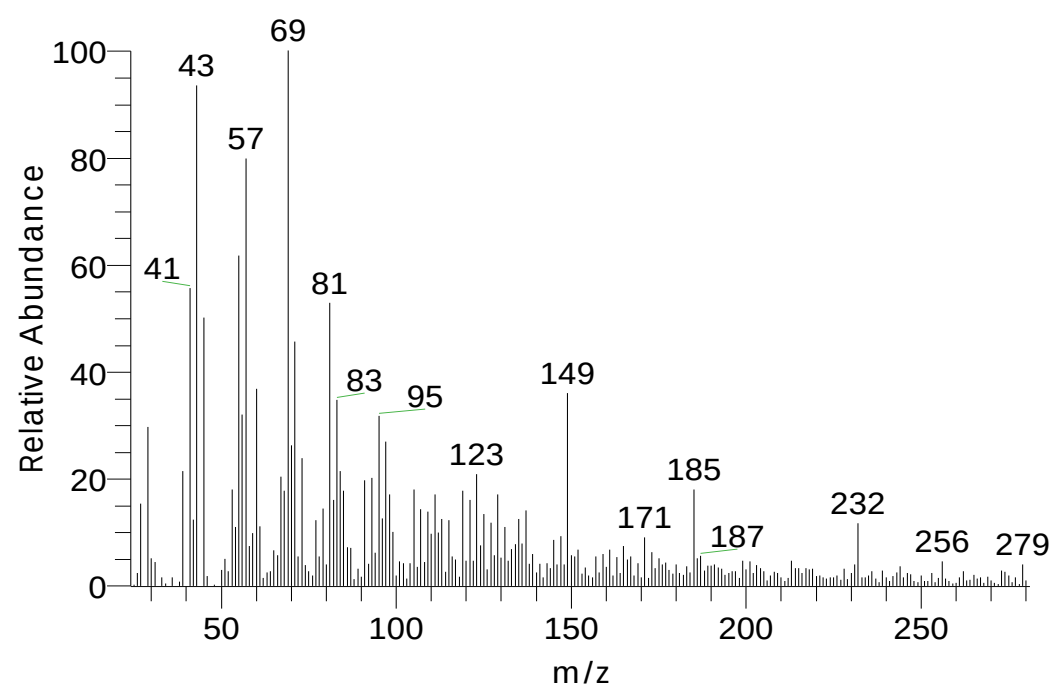

03027205002 FLAVONE 4'-OH,5-OH,7-DI-O-GLUCOSIDE
Formula C27H30O15, MW 594, CAS# NA, Entry# 296184

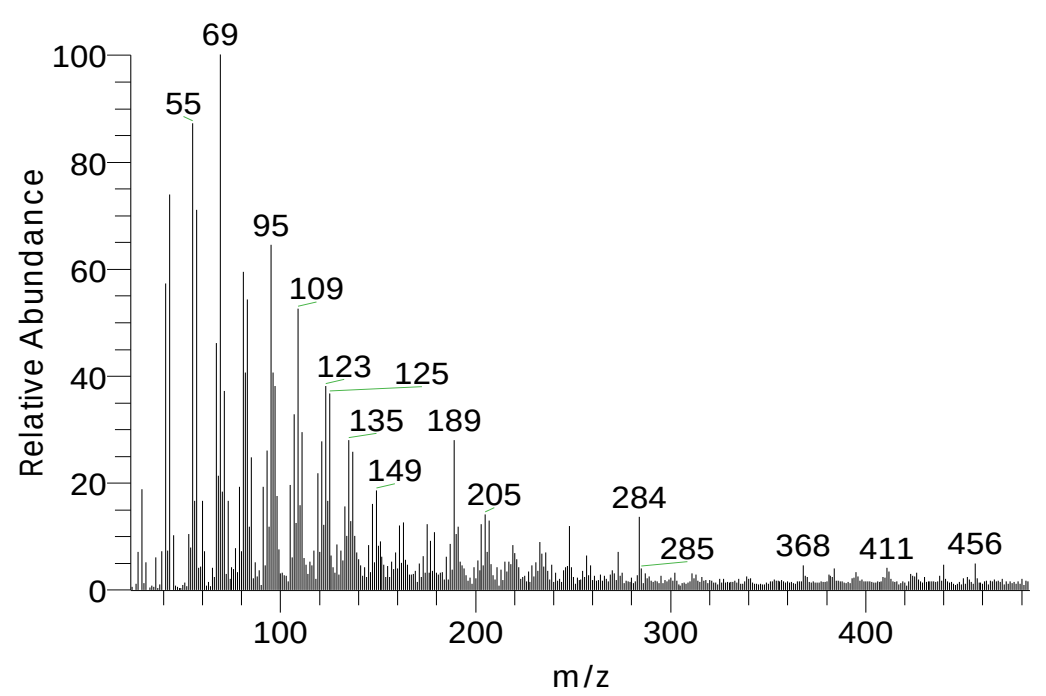

My GC-MS Report

Oil_DrHafsa #10262 RT: 38.41 AV: 1 NL: 3.14E5
T: + c EI Full ms [50.000-750.000]

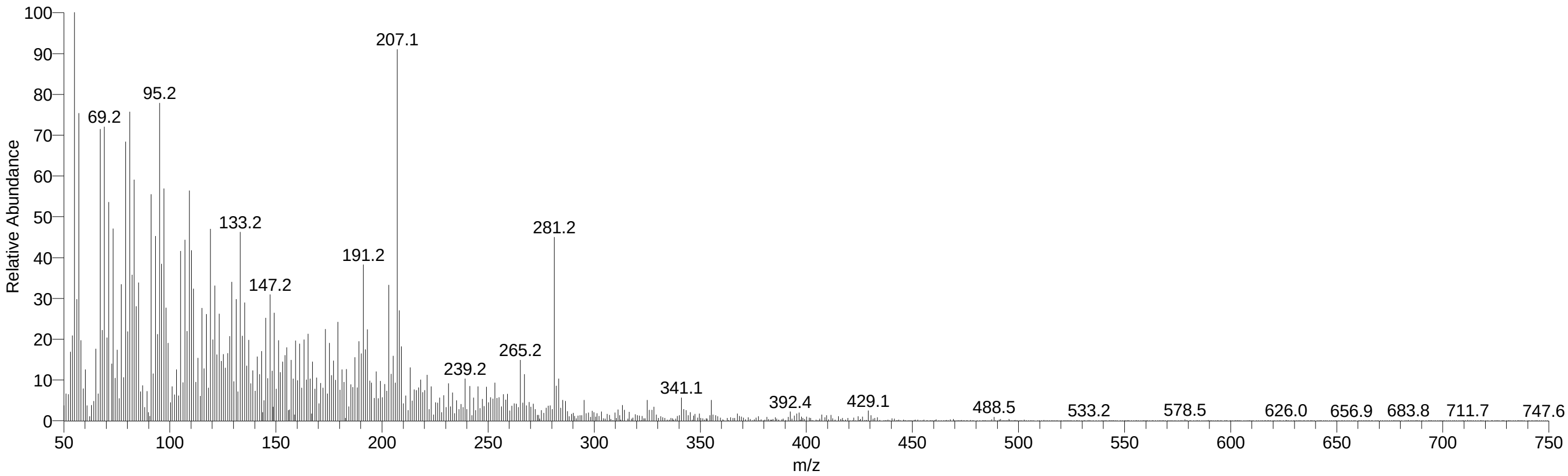

RT	Compound Name	Area %	MF	Molecular Formula	Molecular Weight	Cas #	Library
38.41	Ethyl iso-allocholate	0.20	798	C26H44O5	436	NA	mainlib
38.41	ETHYL ISO-ALLOCHOLATE	0.20	798	C26H44O5	436	NA	WileyRegistry8e
38.41	1-Heptatriacotanol	0.20	802	C37H76O	536	105794-58-9	mainlib
38.41	9,12,15-OCTADECATRIENOIC ACID, 2,3-BIS[(TRIMETHYLSILYL)OXY]PROPYL ESTER, (Z,Z,Z)-	0.20	792	C27H52O4Si2	496	55521-22-7	WileyRegistry8e
38.41	7,8-Epoxylanostan-11-ol, 3-acetoxy-	0.20	737	C32H54O4	502	NA	mainlib

Compound Structure

Hit Spectrum

Ethyl iso-allocholate
Formula C26H44O5, MW 436, CAS# NA, Entry# 7020
Ethyl 3,7,12-trihydroxycholan-24-oate #

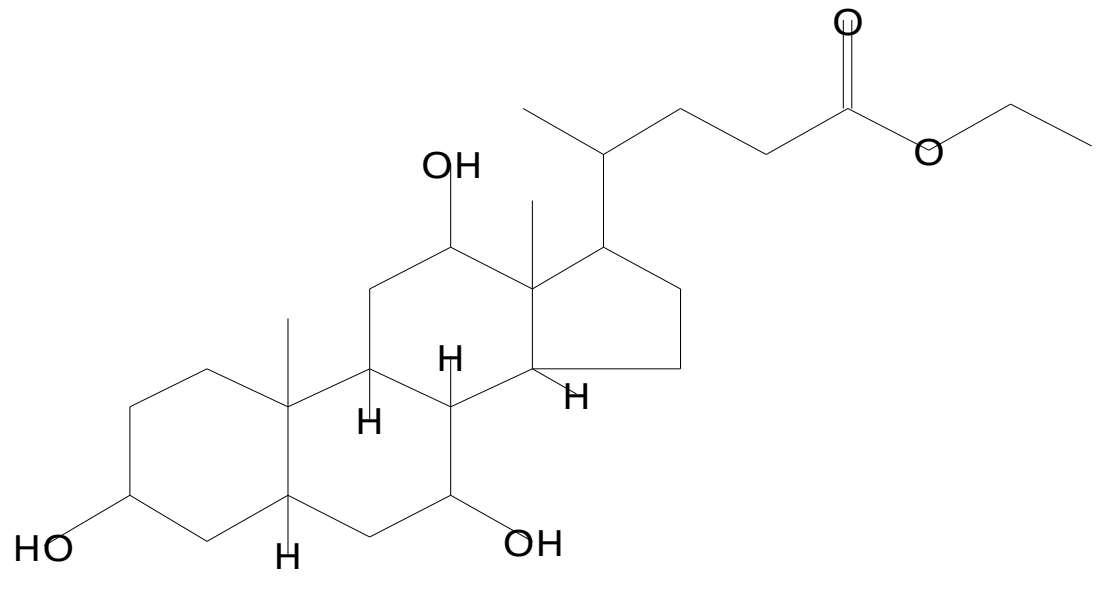

ETHYL ISO-ALLOCHOLATE
Formula C26H44O5, MW 436, CAS# NA, Entry# 270212

SI 785, RSI 798, mainlib, Entry# 7020, CAS# NA, Ethyl iso-allocholate

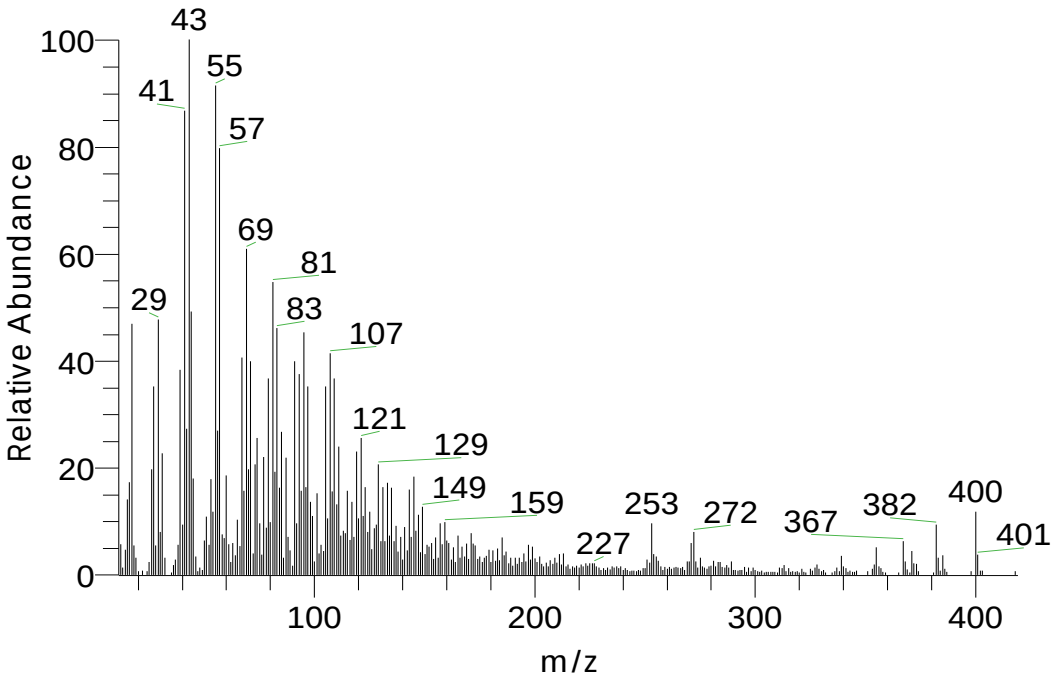

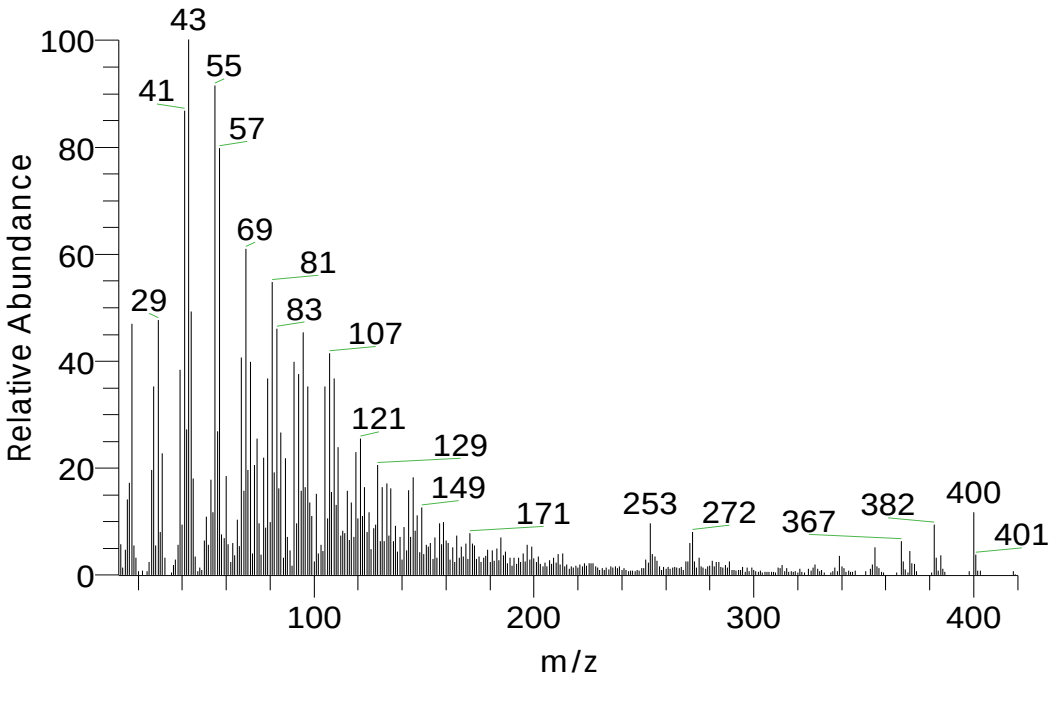

My GC-MS Report

Compound Structure | Hit Spectrum

1-Heptatriacotanol
Formula C37H76O, MW 536, CAS# 105794-58-9, Entry# 7279
1-Heptatriacontanol #

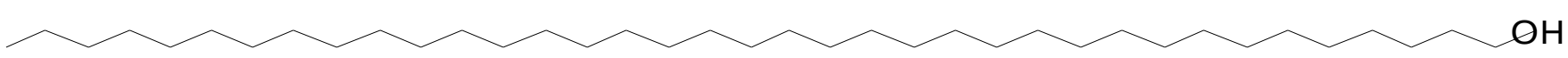

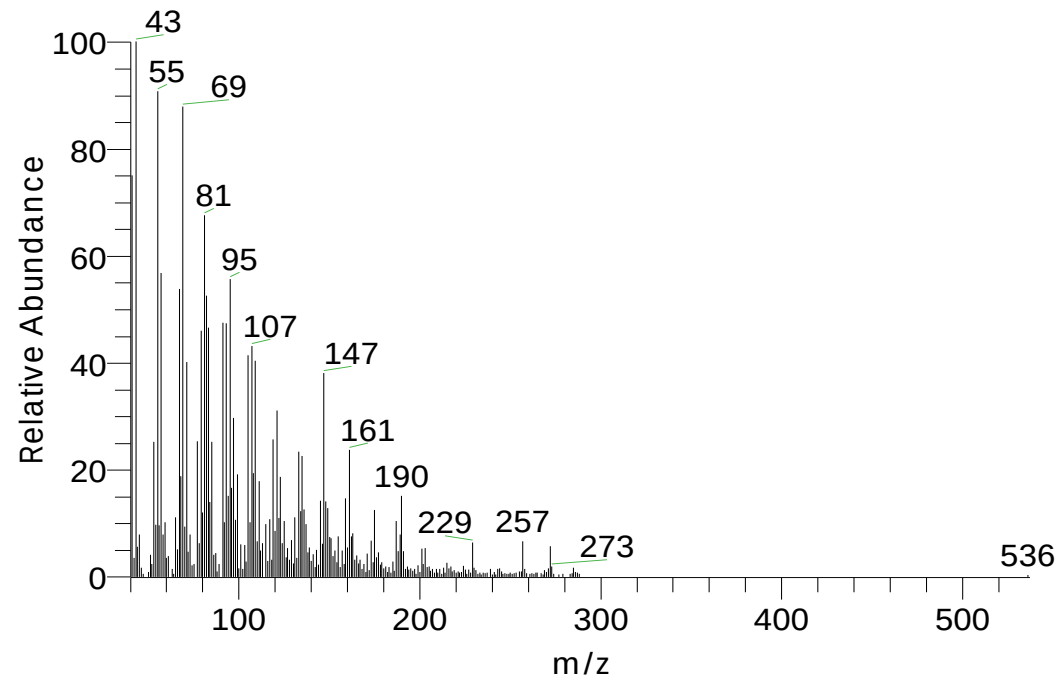

9,12,15-OCTADECATRIENOIC ACID, 2,3-BIS[(TRIMETHYLSILYL)OXY]PROPYL ESTER, (Z,Z,Z)-
Formula C27H52O4Si2, MW 496, CAS# 55521-22-7, Entry# 284834
2,3-BIS[(TRIMETHYLSILYL)OXY]PROPYL (9E,12E,15E)-9,12,15-OCTADECATRIENOATE #

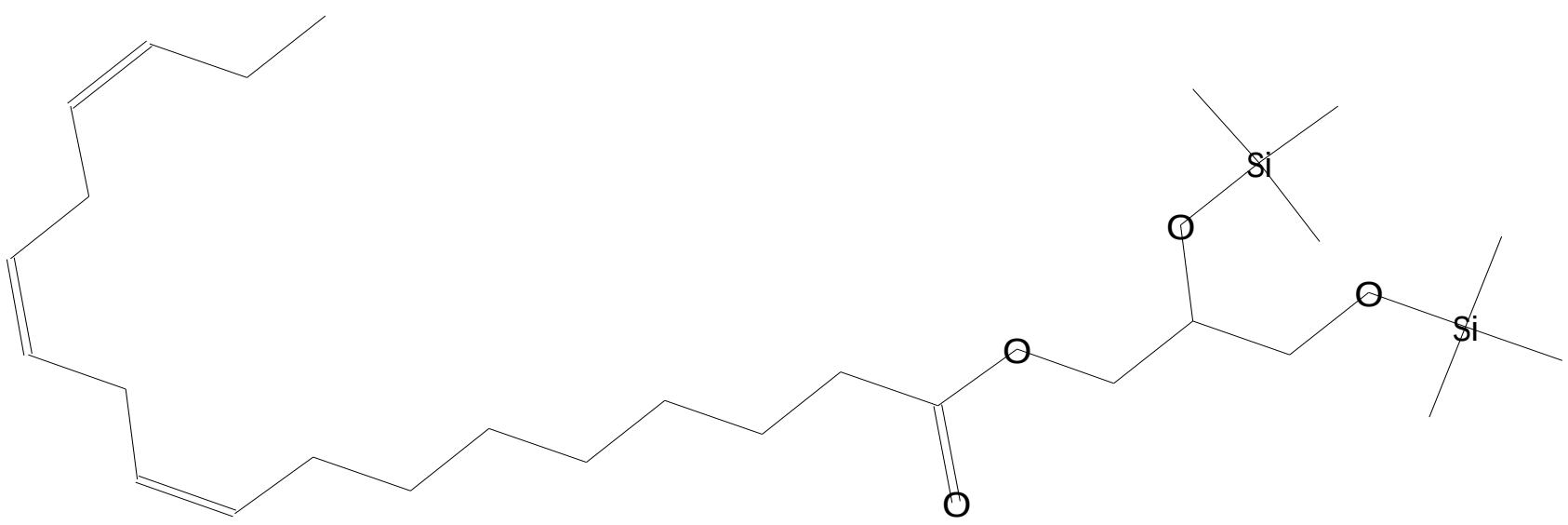

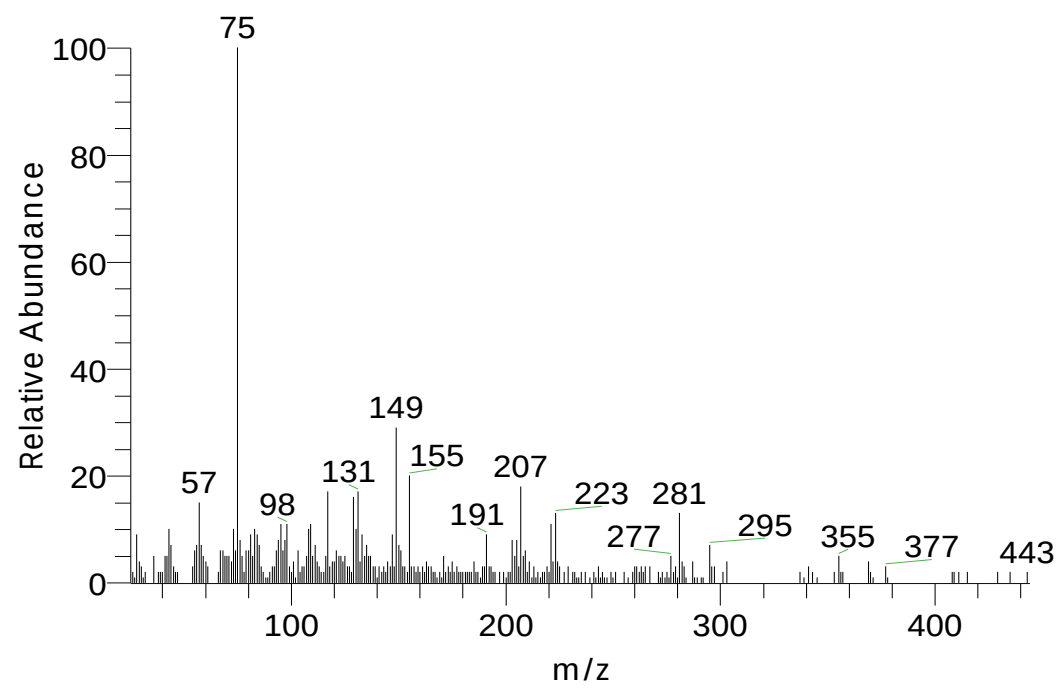

7,8-Epoxylanostan-11-ol, 3-acetoxy-
Formula C32H54O4, MW 502, CAS# NA, Entry# 15359

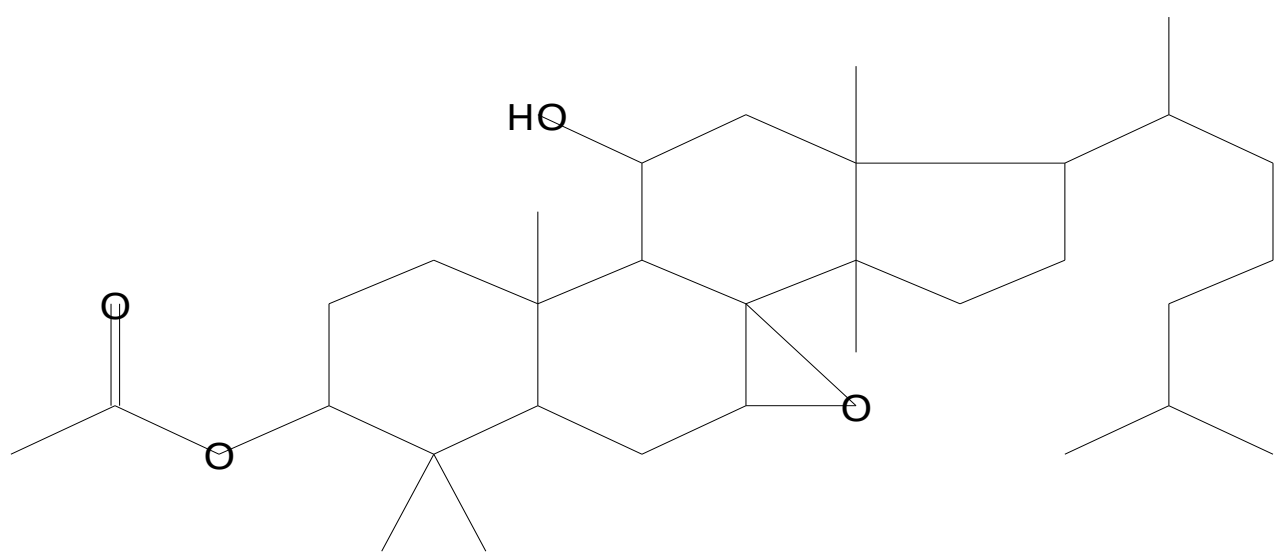

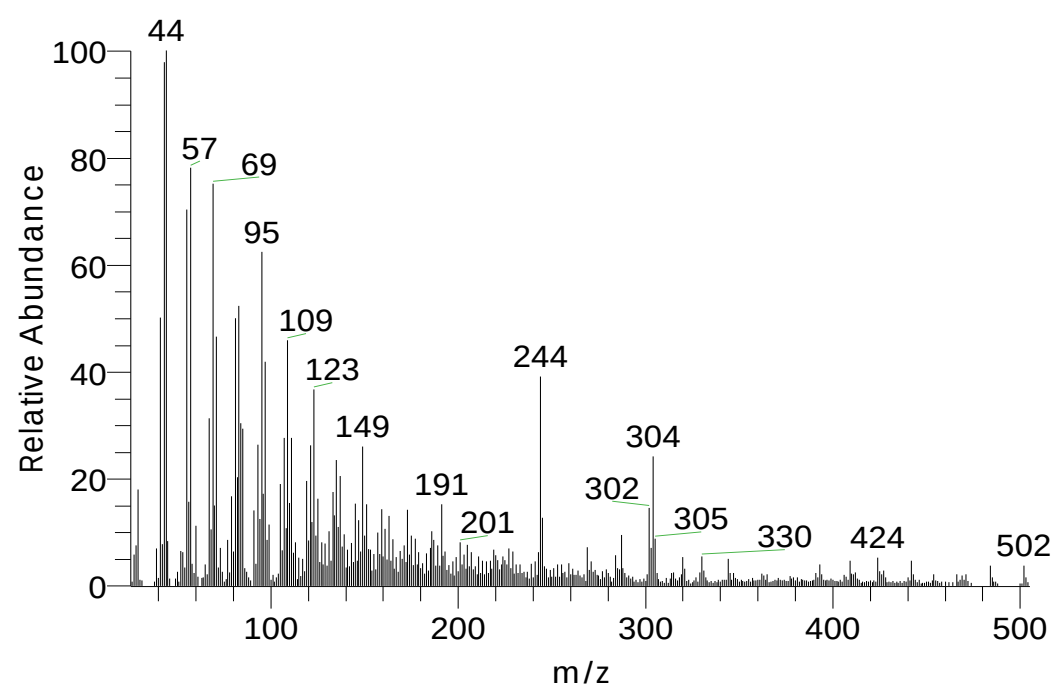

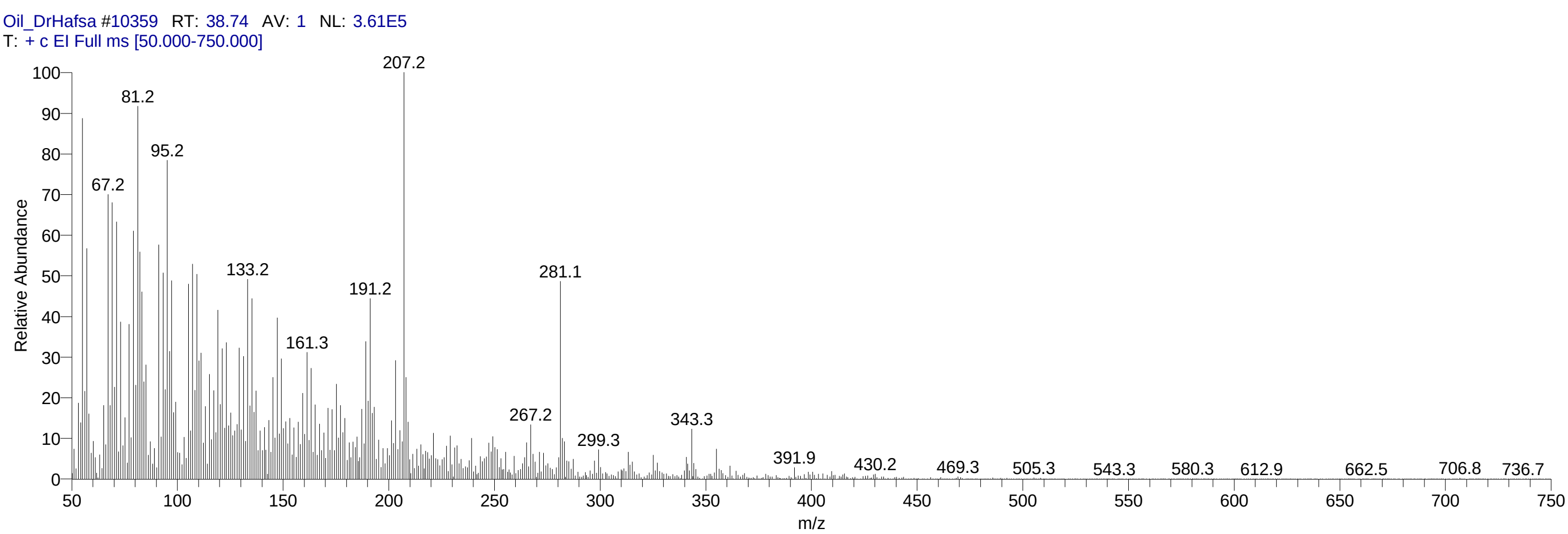

RT	Compound Name	Area %	MF	Molecular Formula	Molecular Weight	Cas #	Library
38.74	Betulin	0.58	747	C30H50O2	442	473-98-3	replib

My GC-MS Report

RT	Compound Name	Area %	MF	Molecular Formula	Molecular Weight	Cas #	Library
38.74	9,12,15-OCTADECATRIENOIC ACID, 2,3-BIS[(TRIMETHYLSILYL)OXY]PROPYL ESTER, (Z,Z,Z)-	0.58	782	C27H52O4Si2	496	55521-22-7	WileyRegistry8e
38.74	Tricyclo[20.8.0.0(7,16)]triacontane, 1(22),7(16)-diepoxy-	0.58	797	C30H52O2	444	NA	mainlib
38.74	TRICYCLO[20.8.0.0E7,16]TRIACONTAN, 1(22),7(16)-DIEPOXY-	0.58	797	C30H52O2	444	NA	WileyRegistry8e
38.74	Betulinaldehyde	0.58	748	C30H48O2	440	13159-28-9	mainlib

Compound Structure	Hit Spectrum

Betulin
Formula C30H50O2, MW 442, CAS# 473-98-3, Entry# 27738
Lup-20(29)-ene-3,28-diol, (3á)-

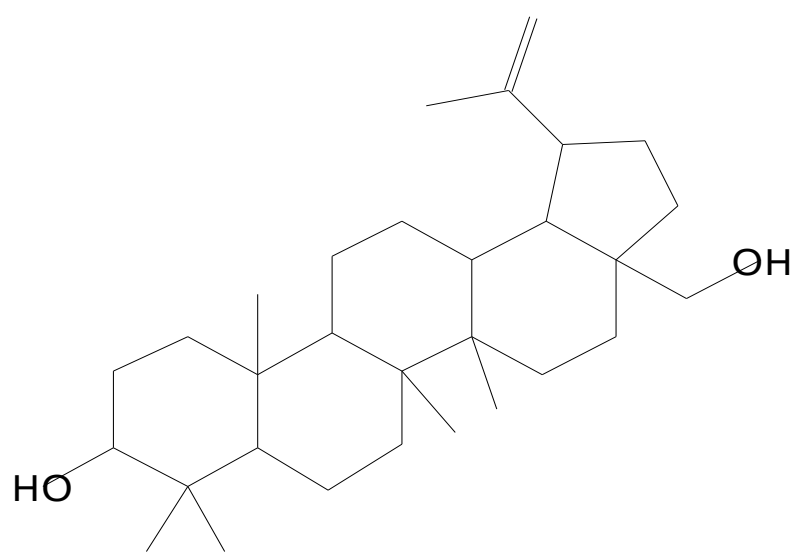

SI 741, RSI 747, replib, Entry# 27738, CAS# 473-98-3, Betulin

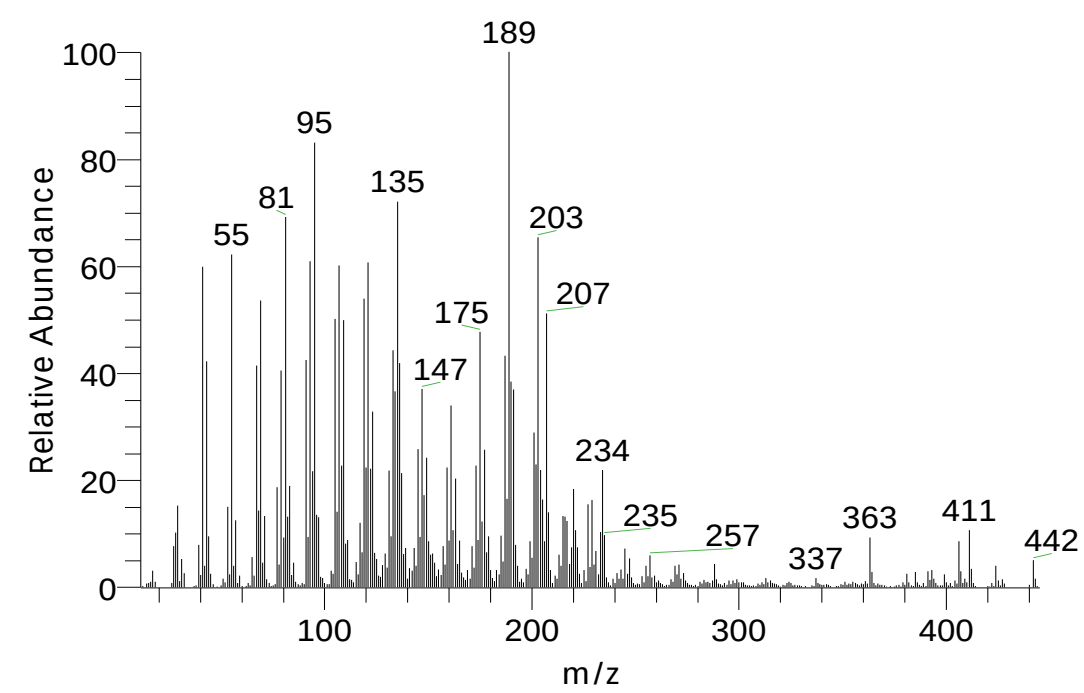

9,12,15-OCTADECATRIENOIC ACID, 2,3-BIS[(TRIMETHYLSILYL)OXY]PROPYL ESTER, (Z,Z,Z)-
Formula C27H52O4Si2, MW 496, CAS# 55521-22-7, Entry# 284834
2,3-BIS[(TRIMETHYLSILYL)OXY]PROPYL (9E,12E,15E)-9,12,15-OCTADECATRIENOATE #

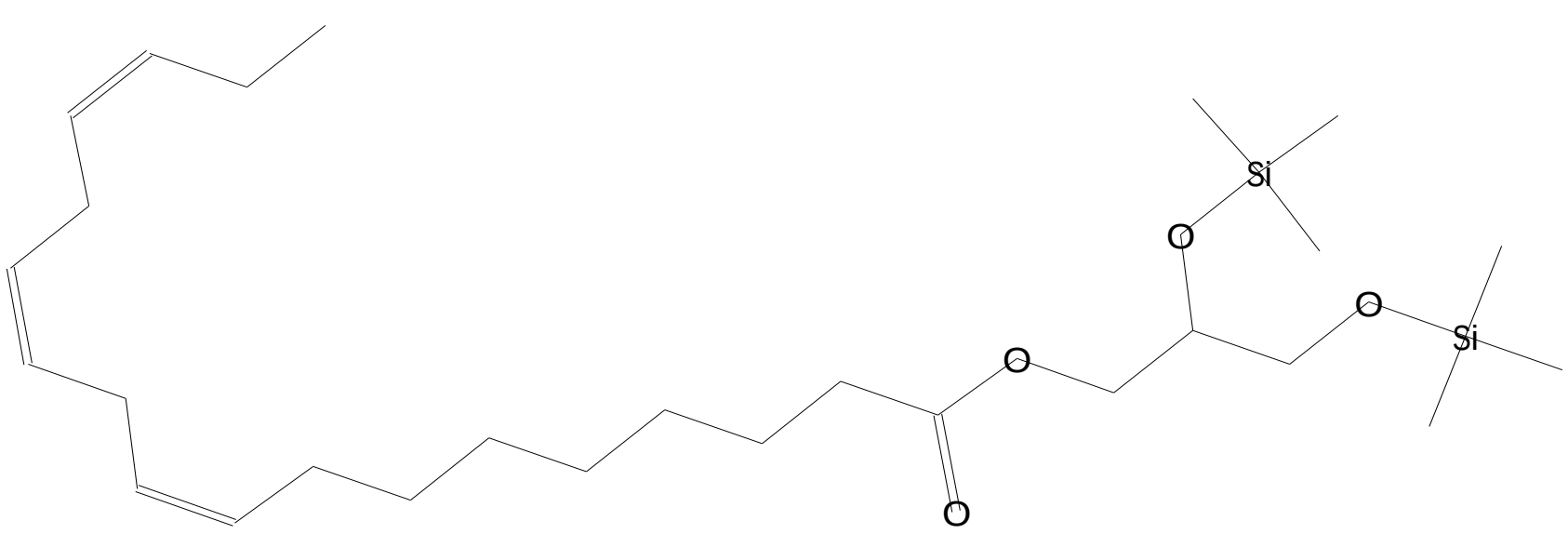

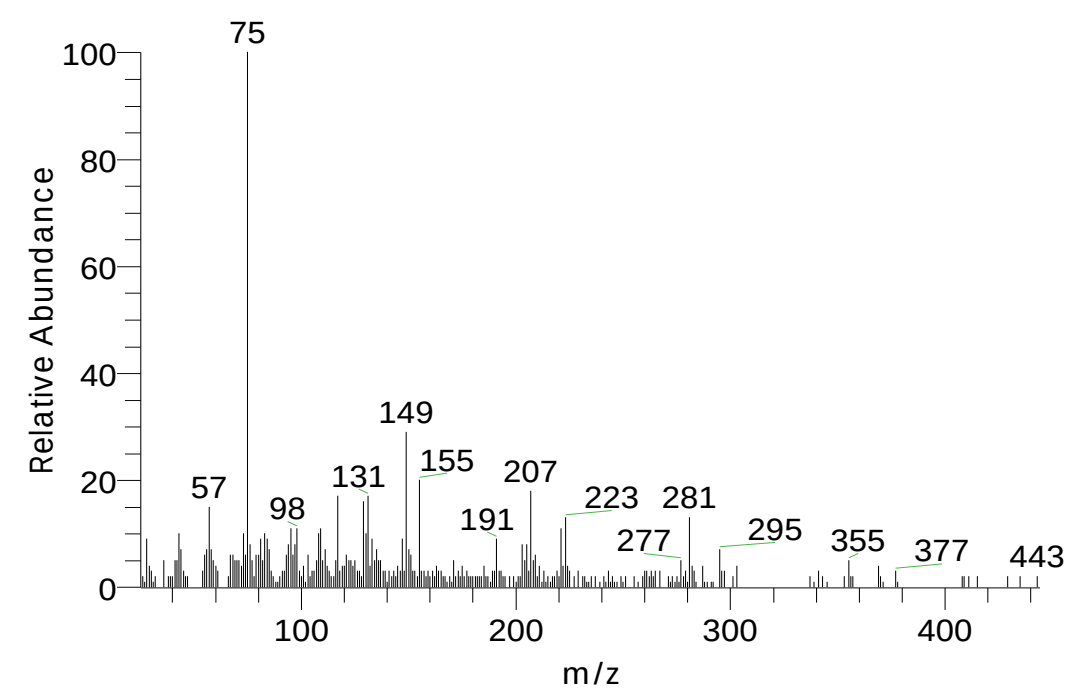

Tricyclo[20.8.0.0(7,16)]triacontane, 1(22),7(16)-diepoxy-
Formula C30H52O2, MW 444, CAS# NA, Entry# 20028
$:28XVGPDAFFXRGERF-UHFFFAOYSA-N

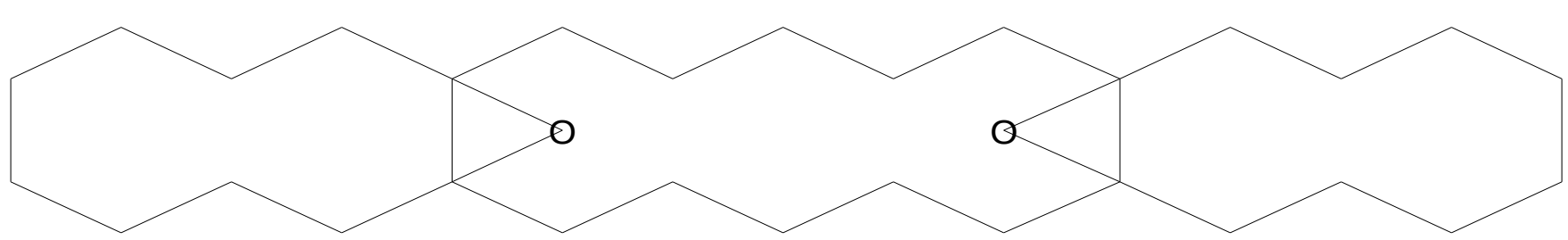

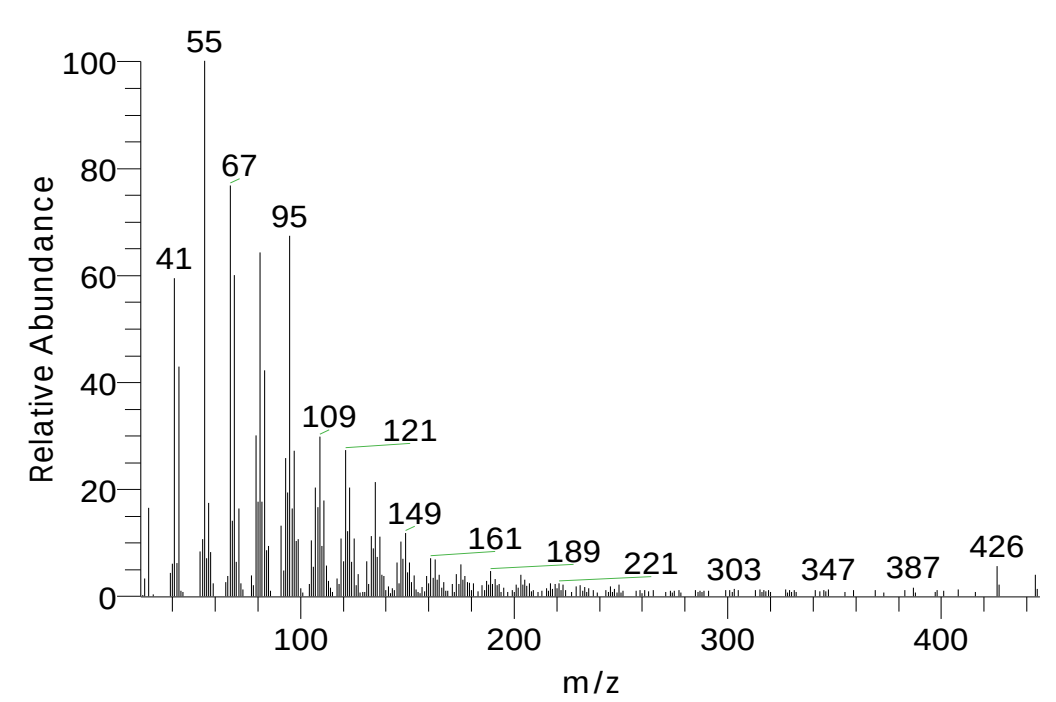

My GC-MS Report

Compound Structure | Hit Spectrum

TRICYCLO[20.8.0.0E7,16]TRIACONTAN, 1(22),7(16)-DIEPOXY-
Formula C30H52O2, MW 444, CAS# NA, Entry# 388503

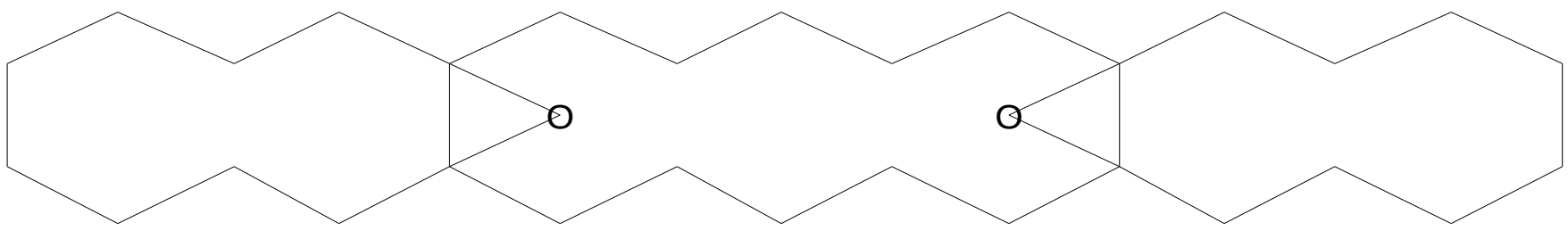

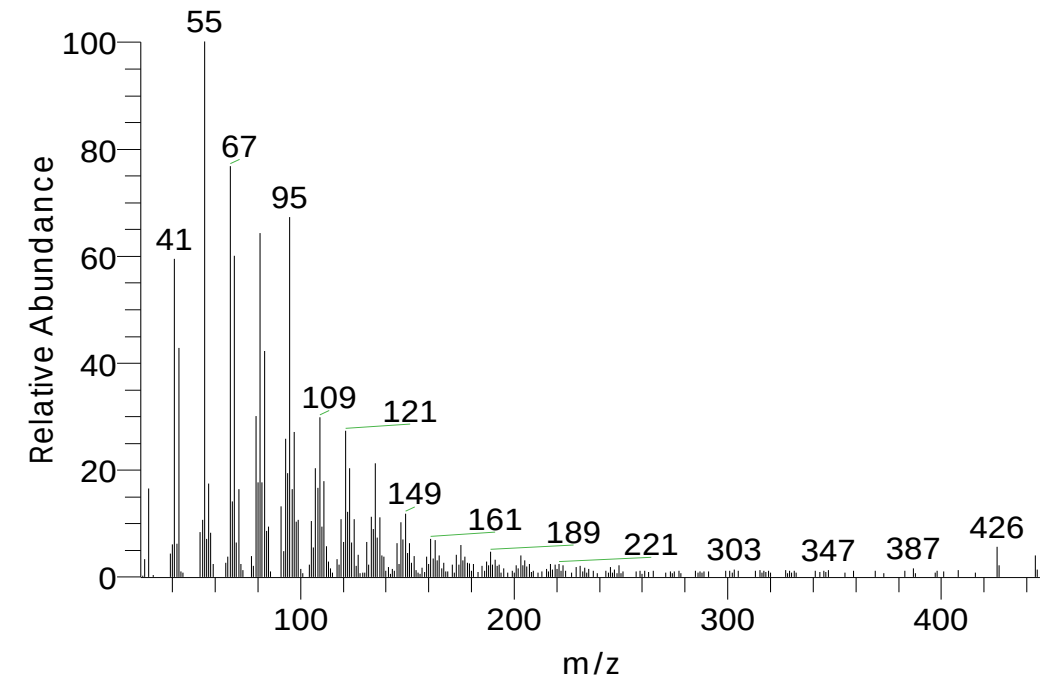

Betulinaldehyde
Formula C30H48O2, MW 440, CAS# 13159-28-9, Entry# 175929
$:28FELCJAPFJOPHSD-UHFFFAOYSA-N

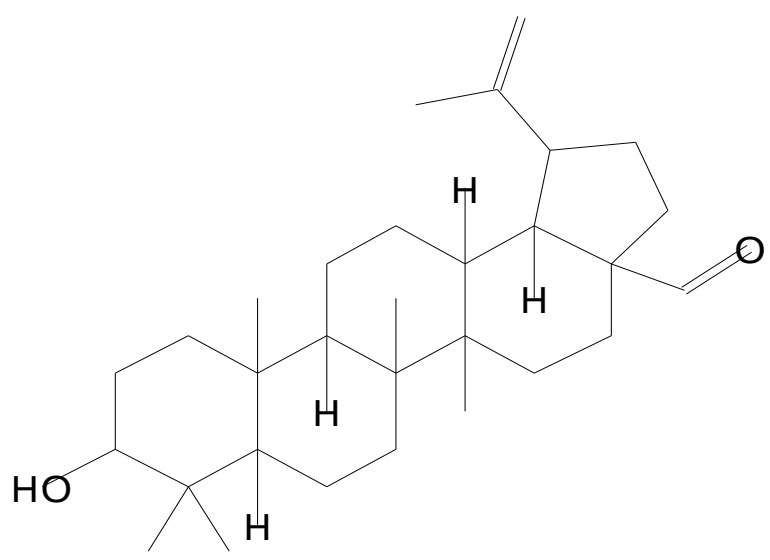

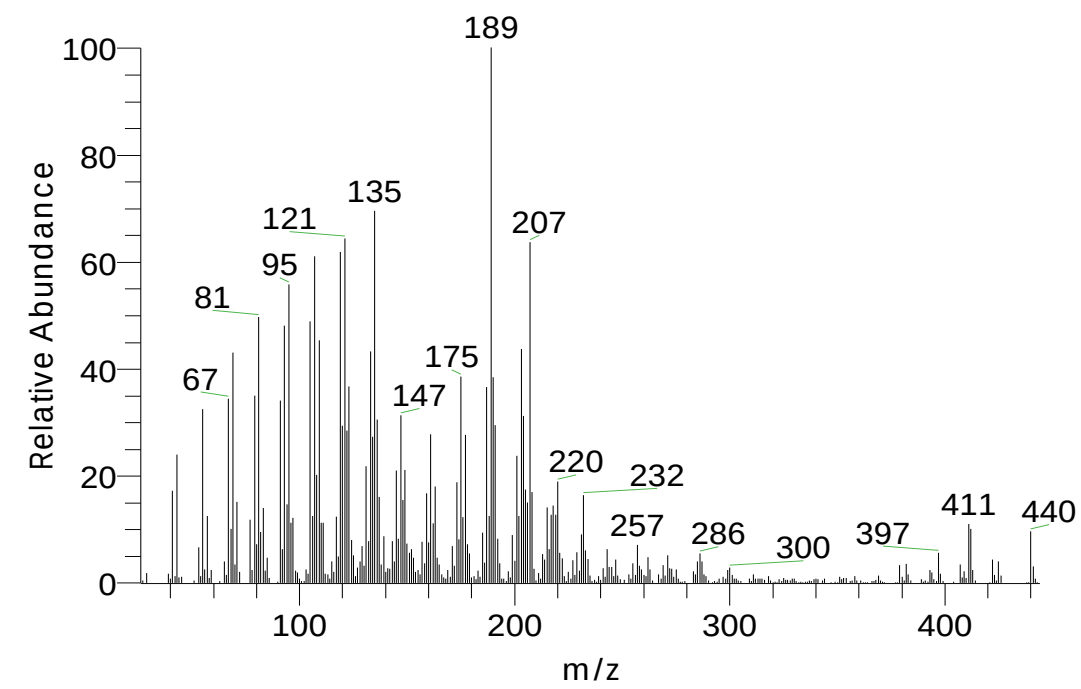

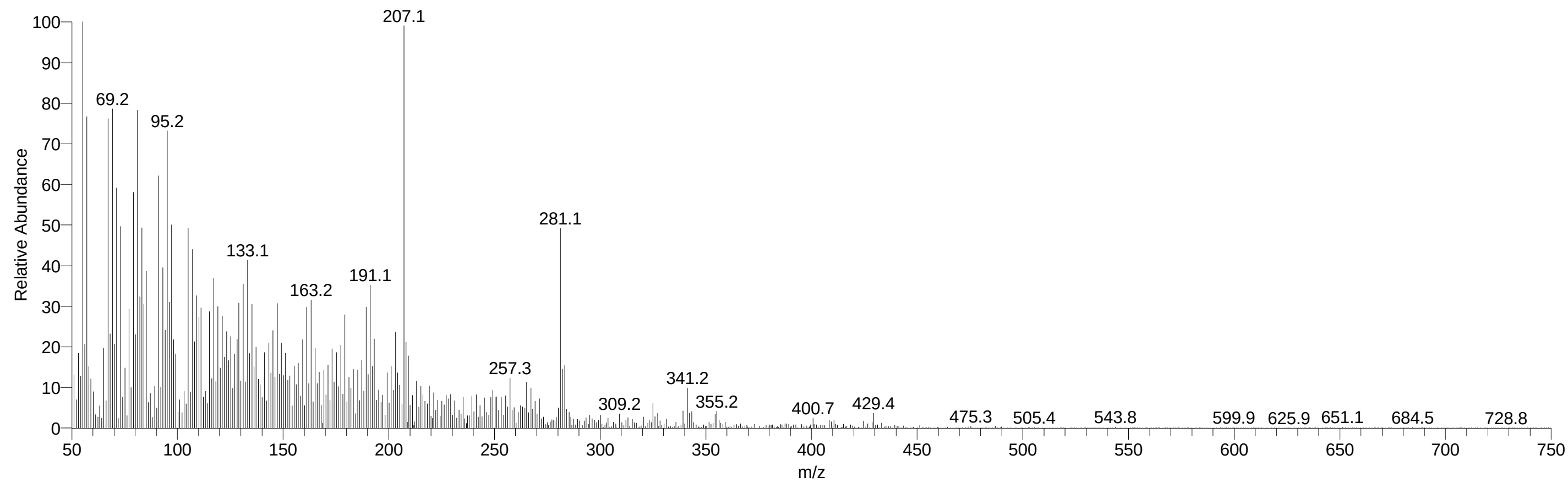

RT	Compound Name	Area %	MF	Molecular Formula	Molecular Weight	Cas #	Library
39.23	ETHYL ISO-ALLOCHOLATE	0.17	783	C26H44O5	436	NA	WileyRegistry8e
39.23	Ethyl iso-allocholate	0.17	783	C26H44O5	436	NA	mainlib
39.23	1-Heptatriacotanol	0.17	807	C37H76O	536	105794-58-9	mainlib
39.23	03027205002 FLAVONE 4'-OH,5-OH,7-DI-O-GLUCOSIDE	0.17	738	C27H30O15	594	NA	WileyRegistry8e
39.23	Betulin	0.17	737	C30H50O2	442	473-98-3	replib

My GC-MS Report

Compound Structure | Hit Spectrum

ETHYL ISO-ALLOCHOLATE
Formula C26H44O5, MW 436, CAS# NA, Entry# 270212

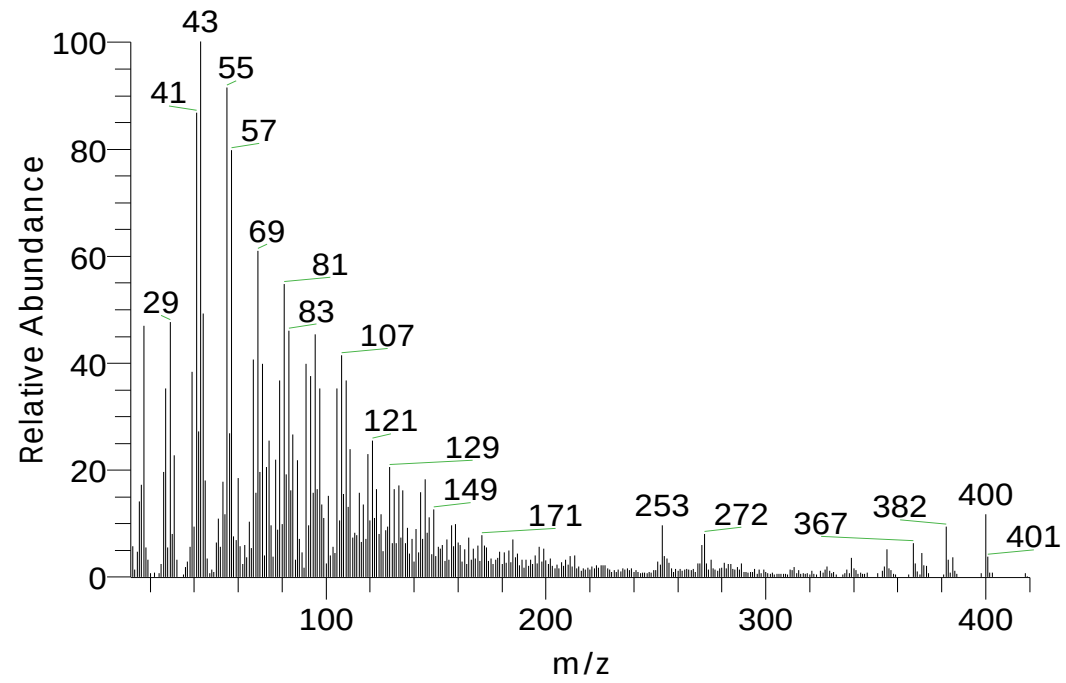

SI 769, RSI 783, mainlib, Entry# 7020, CAS# NA, Ethyl iso-allocholate

Ethyl iso-allocholate
Formula C26H44O5, MW 436, CAS# NA, Entry# 7020
Ethyl 3,7,12-trihydroxycholan-24-oate #

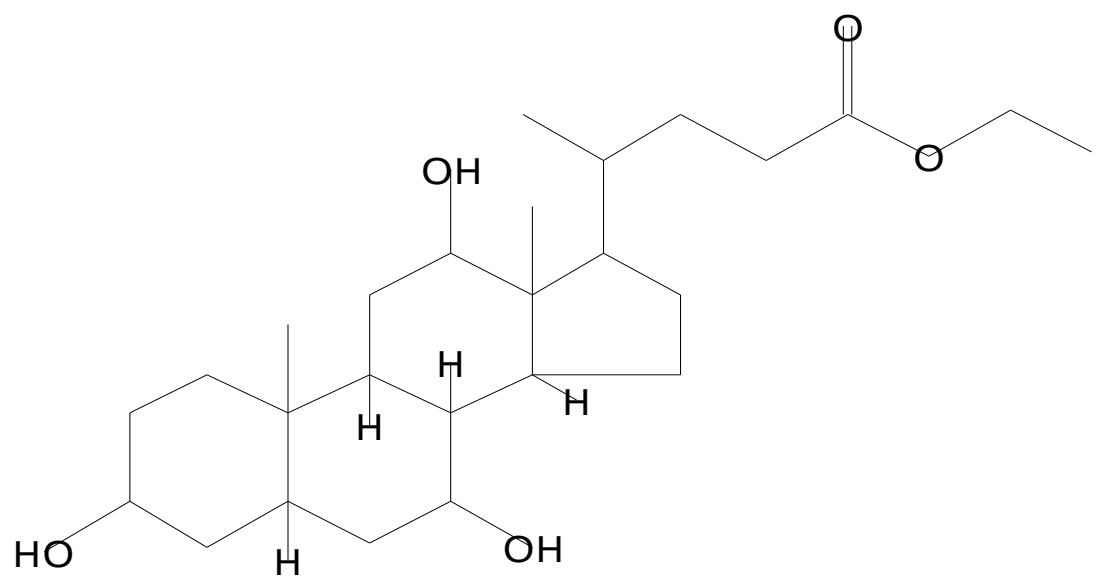

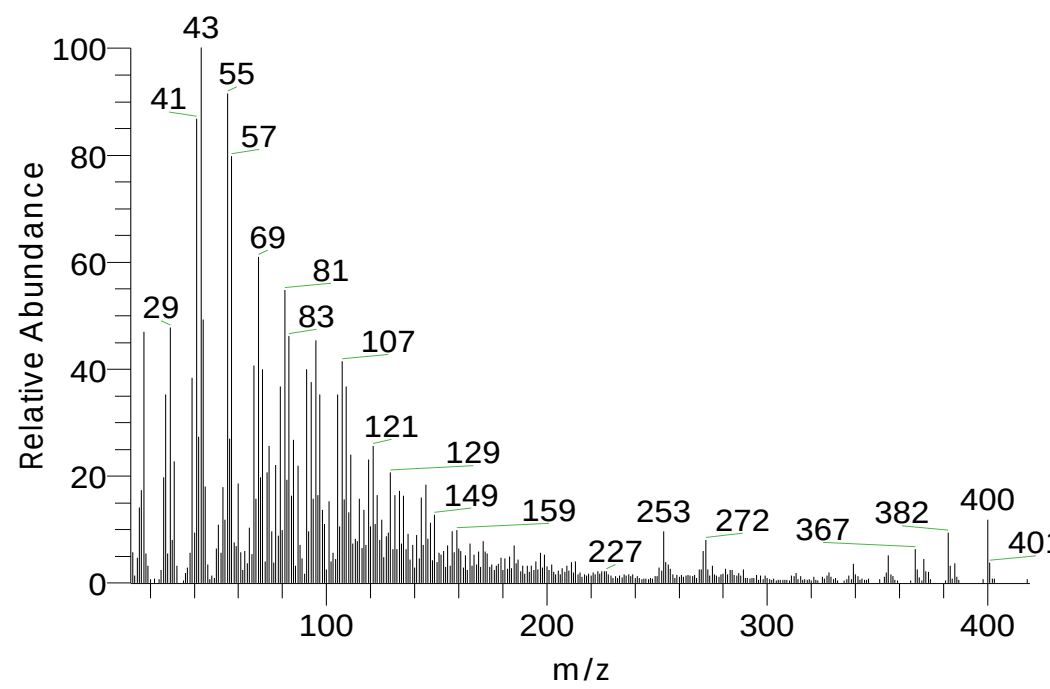

1-Heptatriacotanol
Formula C37H76O, MW 536, CAS# 105794-58-9, Entry# 7279
1-Heptatriacontanol #

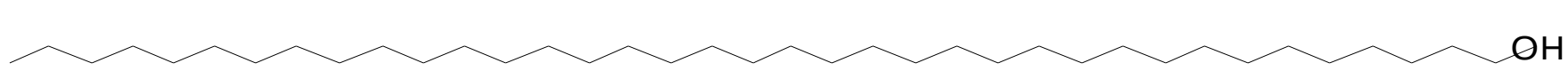

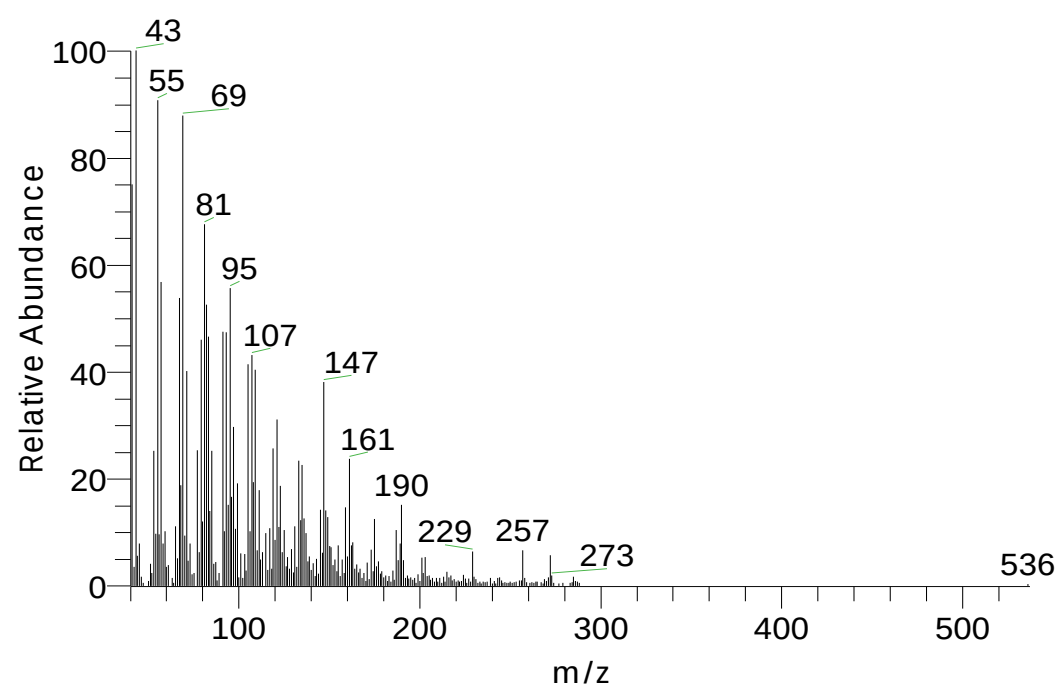

03027205002 FLAVONE 4'-OH,5-OH,7-DI-O-GLUCOSIDE
Formula C27H30O15, MW 594, CAS# NA, Entry# 296184

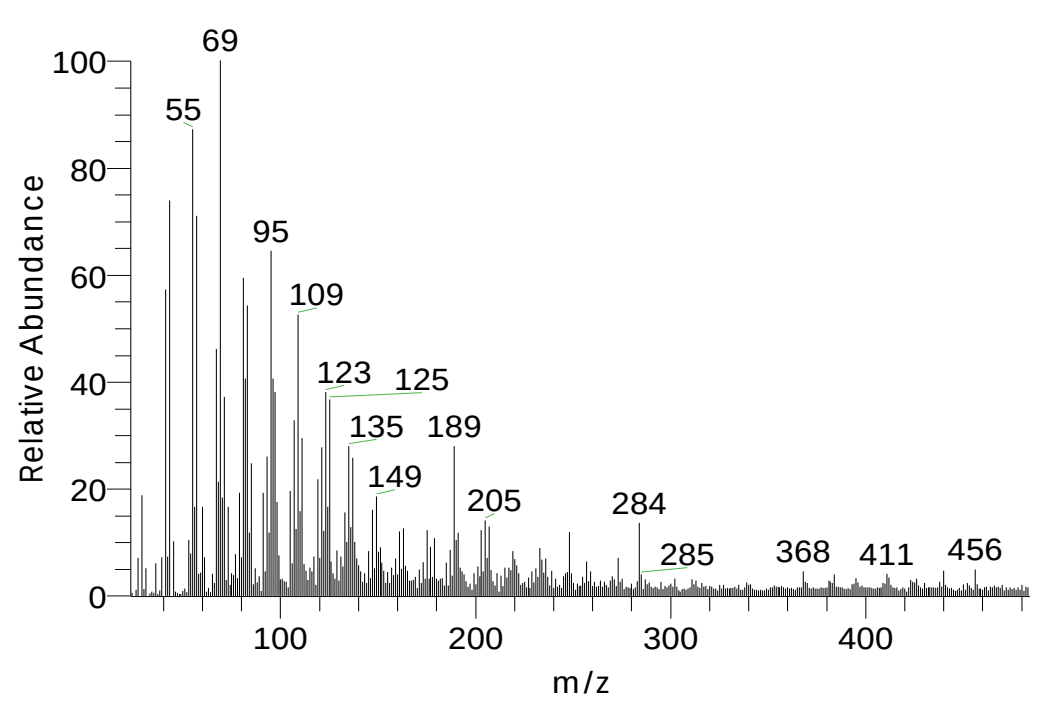

My GC-MS Report

Compound Structure | Hit Spectrum

Betulin
Formula C30H50O2, MW 442, CAS# 473-98-3, Entry# 27738
Lup-20(29)-ene-3,28-diol, (3á)-

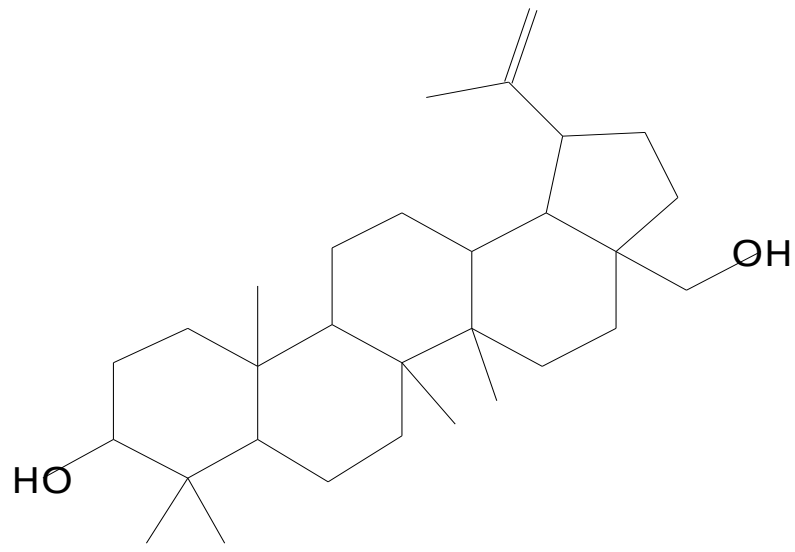

SI 732, RSI 737, replib, Entry# 27738, CAS# 473-98-3, Betulin

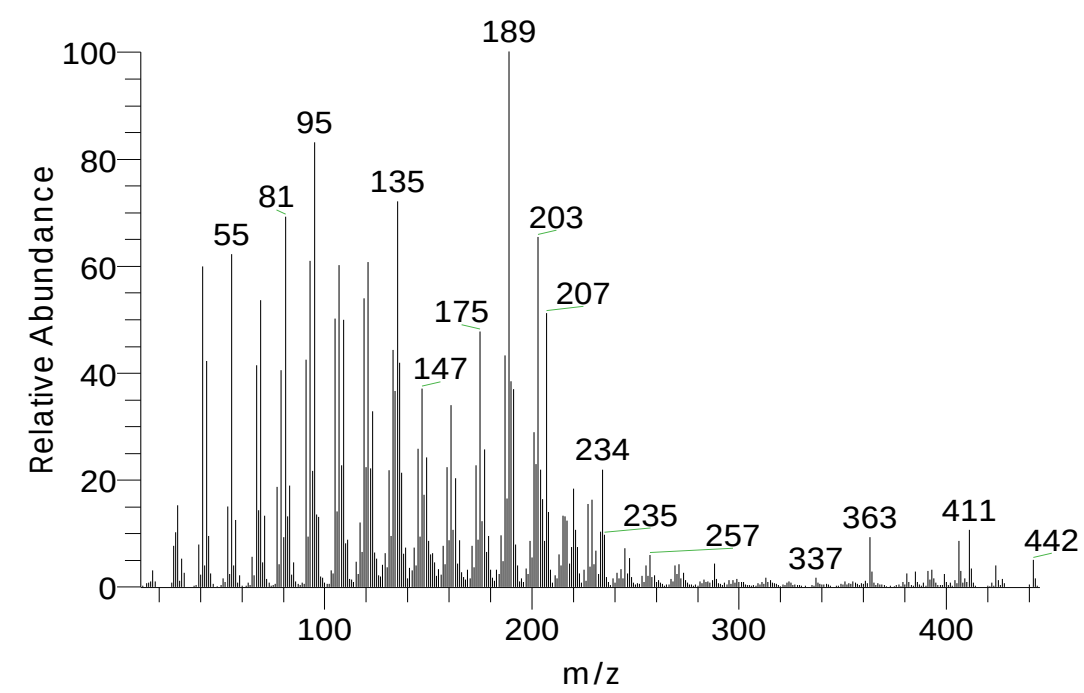

Oil_DrHafsa #10531 RT: 39.31 AV: 1 NL: 4.00E5
T: + c EI Full ms [50.000-750.000]

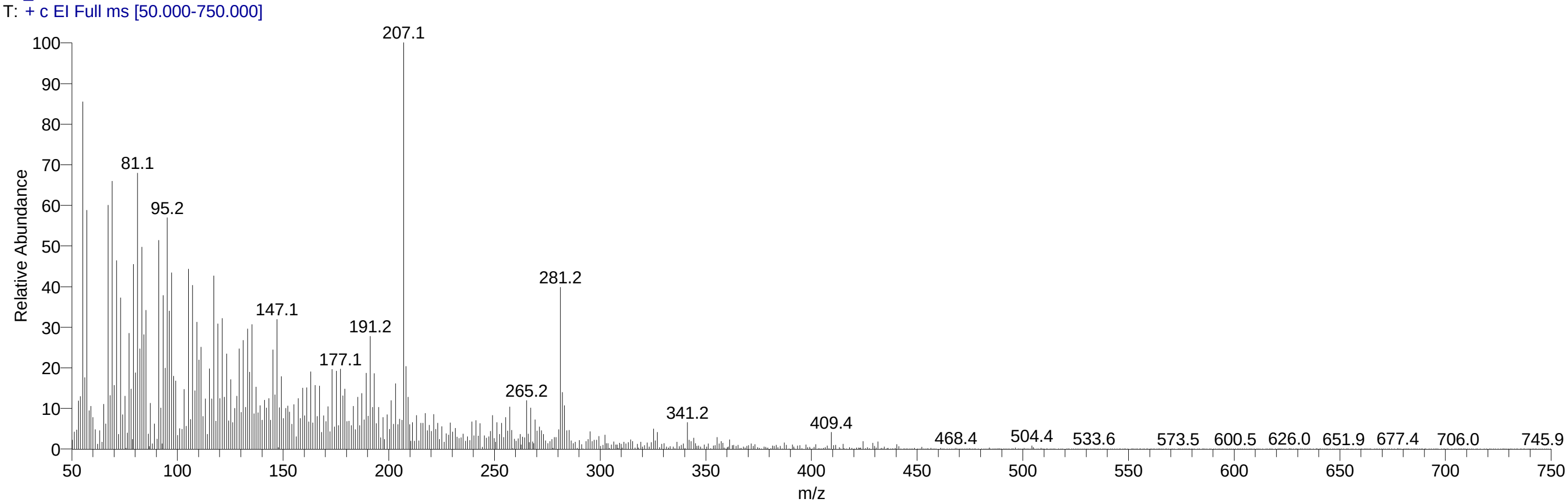

RT	Compound Name	Area %	MF	Molecular Formula	Molecular Weight	Cas #	Library
39.31	Ethyl iso-allocholate	0.25	780	C26H44O5	436	NA	mainlib
39.31	ETHYL ISO-ALLOCHOLATE	0.25	780	C26H44O5	436	NA	WileyRegistry8e
39.31	03027205002 FLAVONE 4'-OH,5-OH,7-DI-O-GLUCOSIDE	0.25	742	C27H30O15	594	NA	WileyRegistry8e
39.31	1-Heptatriacotanol	0.25	804	C37H76O	536	105794-58-9	mainlib
39.31	01297107001 TETRANEURIN - A - DIOL	0.25	793	C15H20O5	280	NA	WileyRegistry8e

Compound Structure | Hit Spectrum

Ethyl iso-allocholate
Formula C26H44O5, MW 436, CAS# NA, Entry# 7020
Ethyl 3,7,12-trihydroxycholan-24-oate #

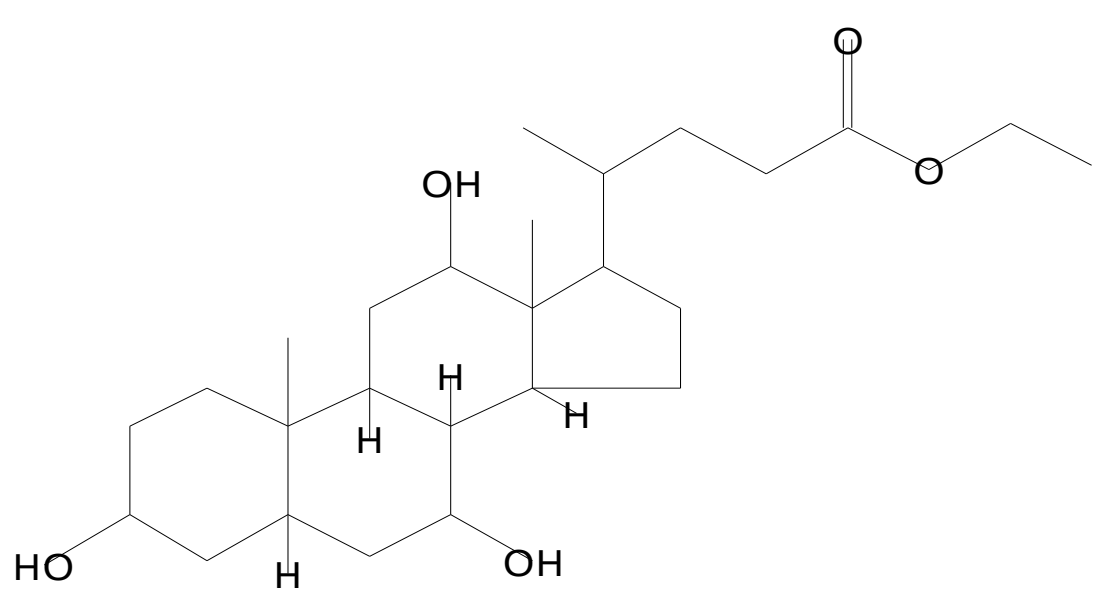

SI 766, RSI 780, mainlib, Entry# 7020, CAS# NA, Ethyl iso-allocholate

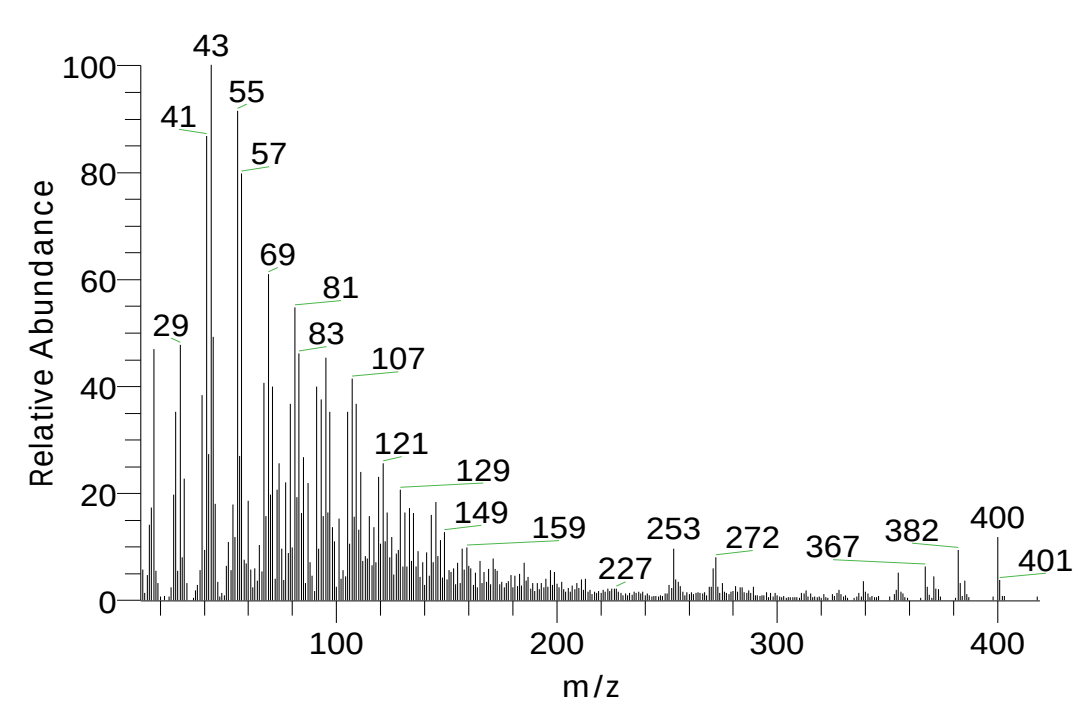

My GC-MS Report

Compound Structure | Hit Spectrum

ETHYL ISO-ALLOCHOLATE
Formula $C_{26}H_{44}O_5$, MW 436, CAS# NA, Entry# 270212

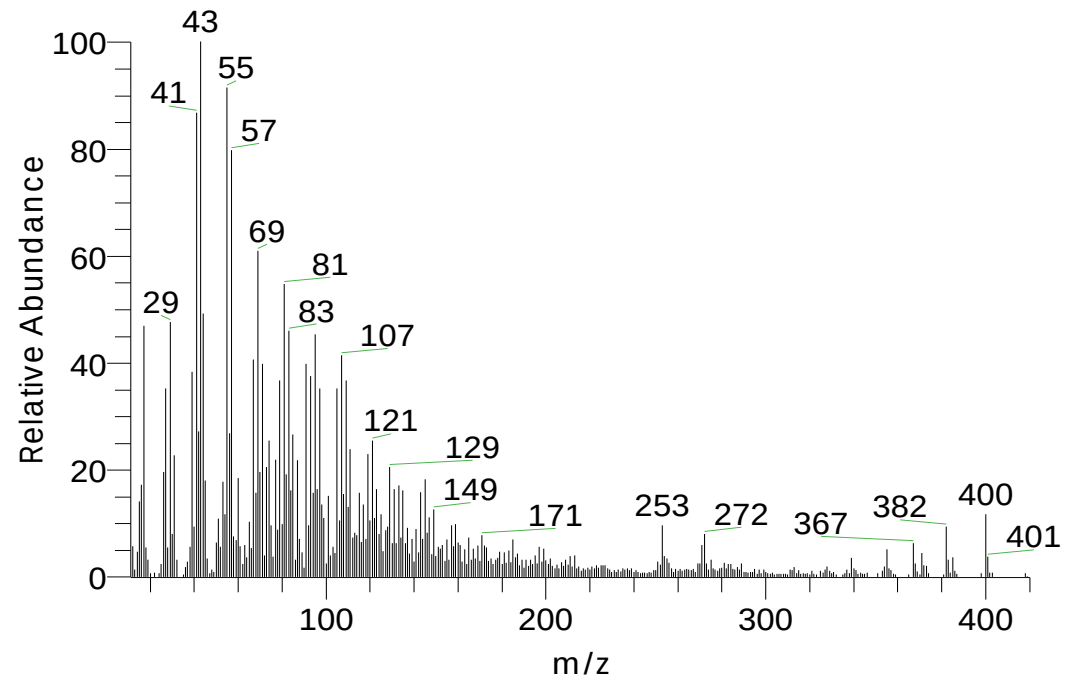

03027205002 FLAVONE 4'-OH,5-OH,7-DI-O-GLUCOSIDE
Formula $C_{27}H_{30}O_{15}$, MW 594, CAS# NA, Entry# 296184

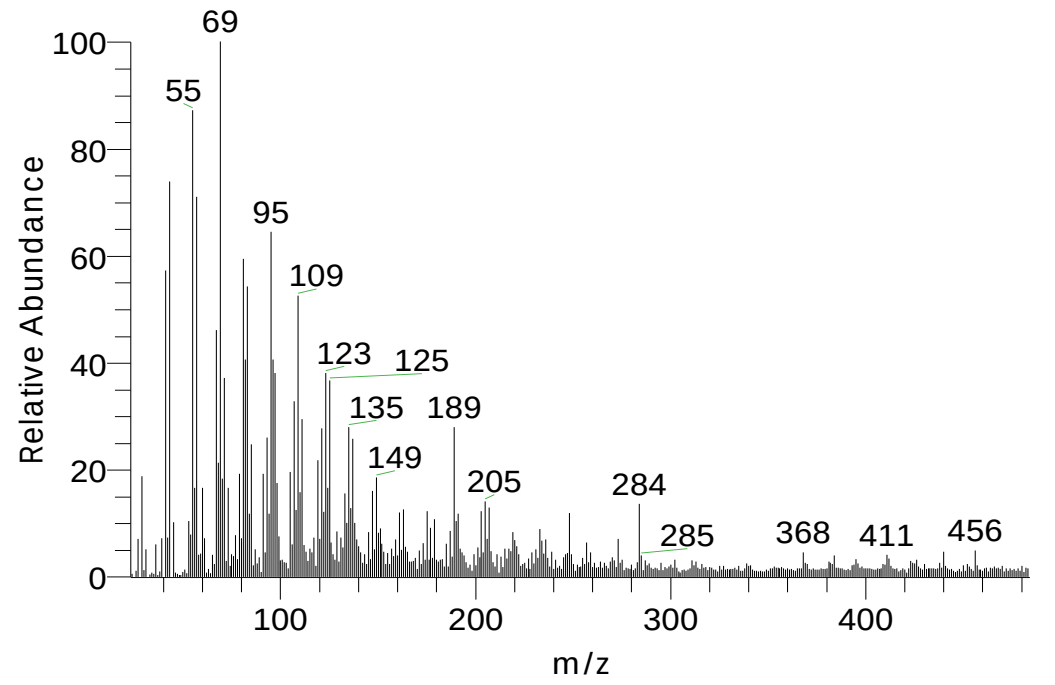

1-Heptatriacotanol
Formula $C_{37}H_{76}O$, MW 536, CAS# 105794-58-9, Entry# 7279
1-Heptatriacontanol #

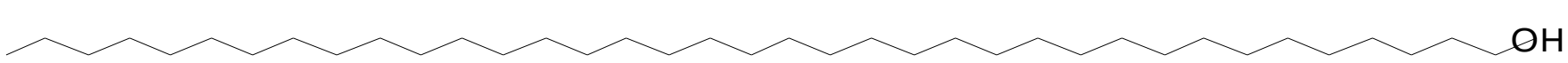

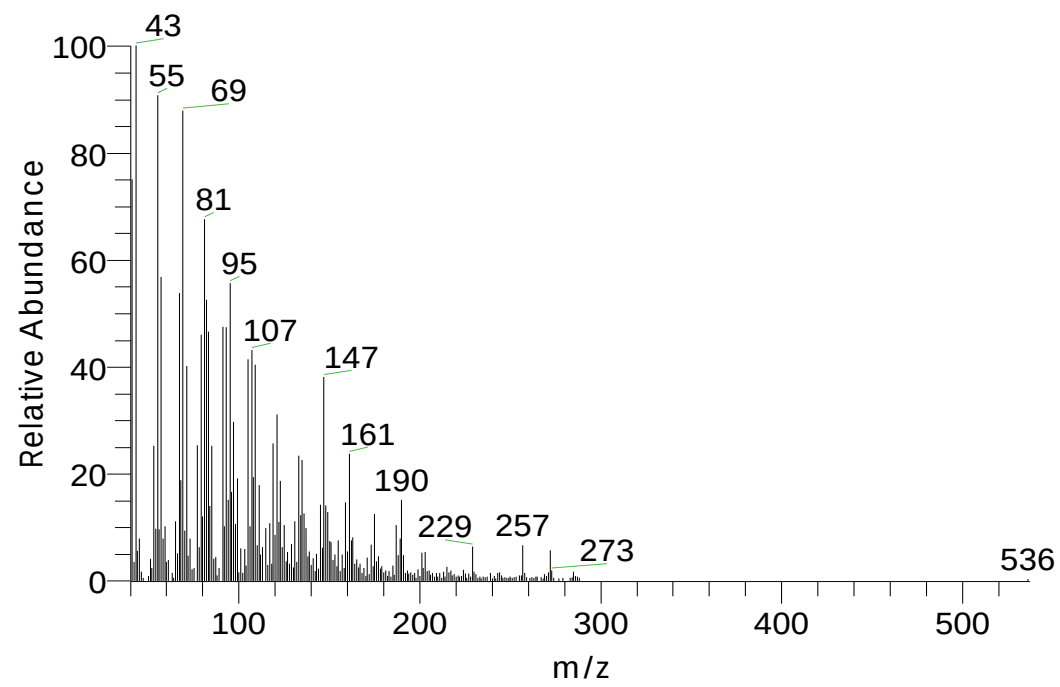

01297107001 TETRANEURIN - A - DIOL
Formula $C_{15}H_{20}O_5$, MW 280, CAS# NA, Entry# 170378

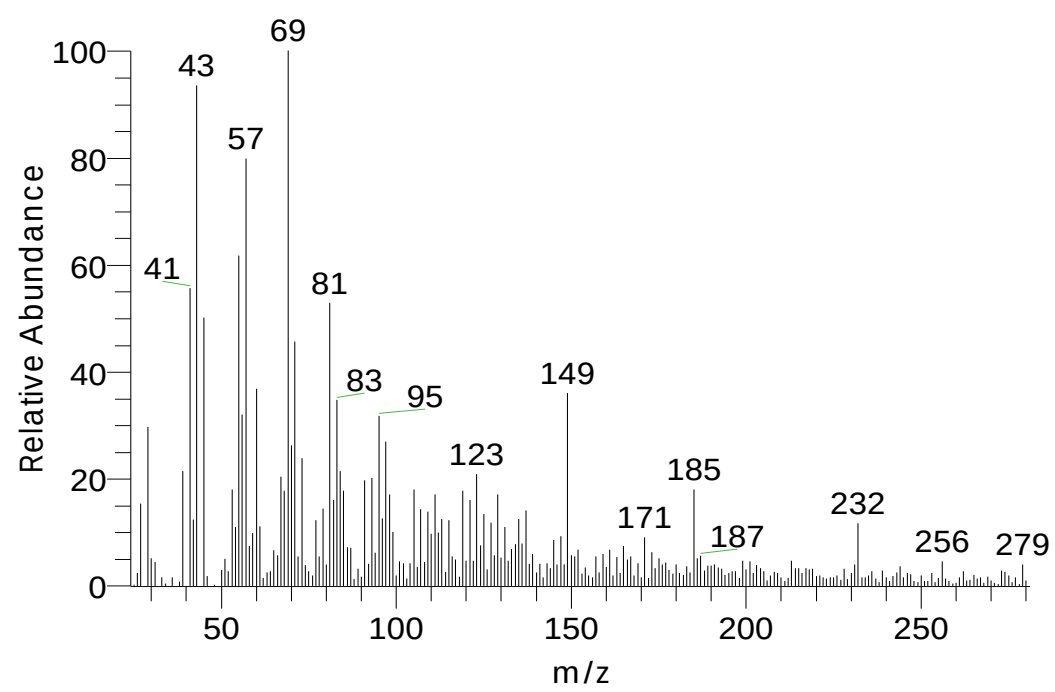

My GC-MS Report

Oil_DrHafsa #10755 RT: 40.06 AV: 1 NL: 4.45E6
T: + c EI Full ms [50.000-750.000]

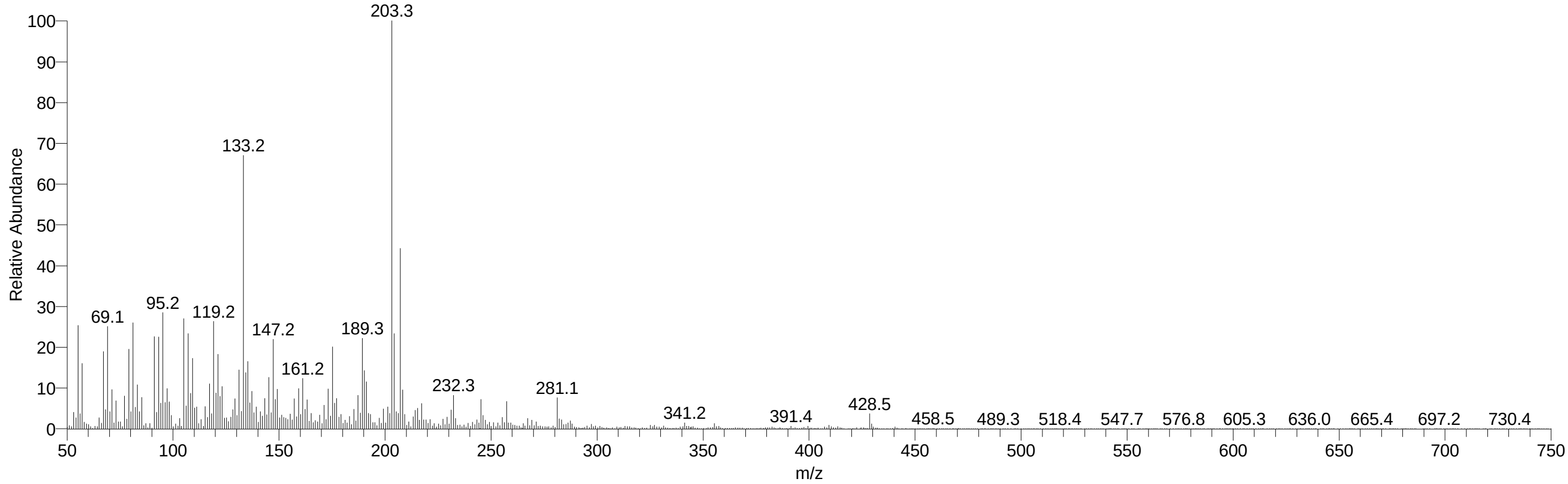

RT	Compound Name	Area %	MF	Molecular Formula	Molecular Weight	Cas #	Library
40.06	Betulin	8.64	802	C30H50O2	442	473-98-3	replib
40.06	Ursolic aldehyde	8.64	845	C30H48O2	440	19132-81-1	mainlib
40.06	Betulinaldehyde	8.64	803	C30H48O2	440	13159-28-9	mainlib
40.06	Urs-12-en-28-al, 3-(acetyloxy)-, (3á)-	8.64	791	C32H50O3	482	86996-88-5	mainlib
40.06	28-OXOURS-12-EN-3-YL ACETATE #	8.64	791	C32H50O3	482	86996-88-5	WileyRegistry8e

Compound Structure

Hit Spectrum

Betulin
Formula C30H50O2, MW 442, CAS# 473-98-3, Entry# 27738
Lup-20(29)-ene-3,28-diol, (3á)-

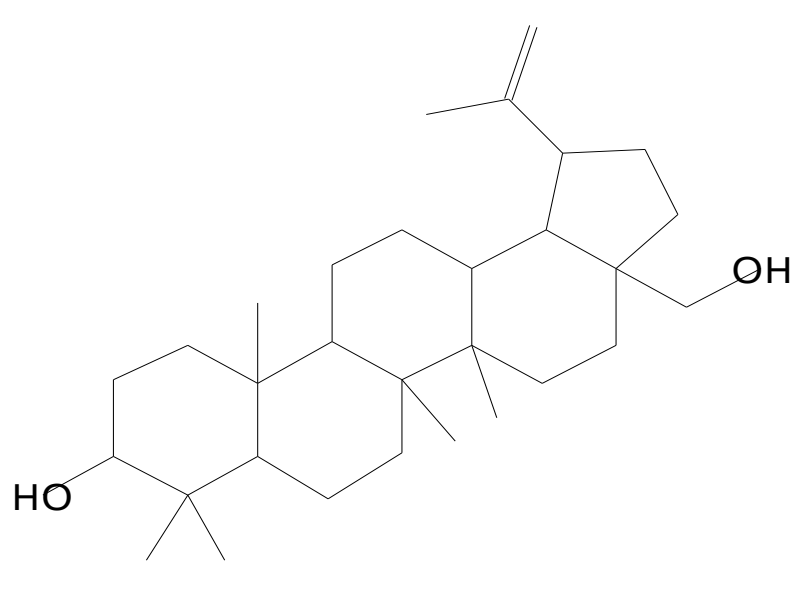

SI 799, RSI 802, replib, Entry# 27738, CAS# 473-98-3, Betulin

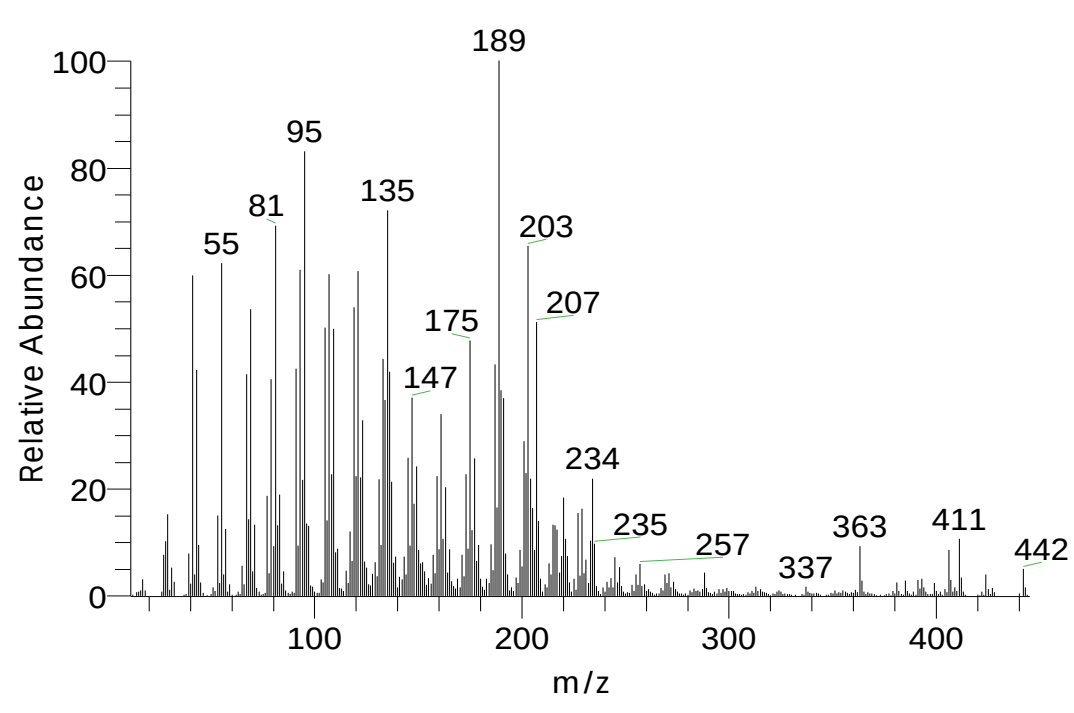

Ursolic aldehyde
Formula C30H48O2, MW 440, CAS# 19132-81-1, Entry# 186335

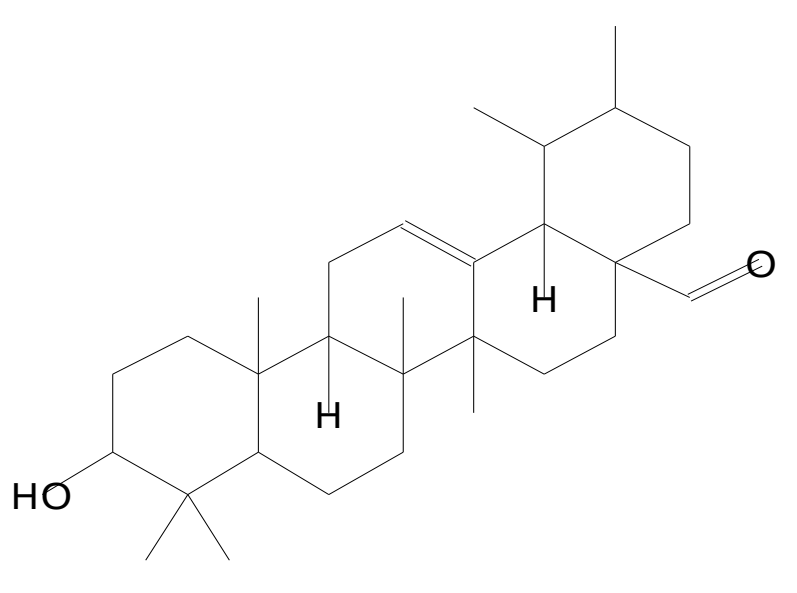

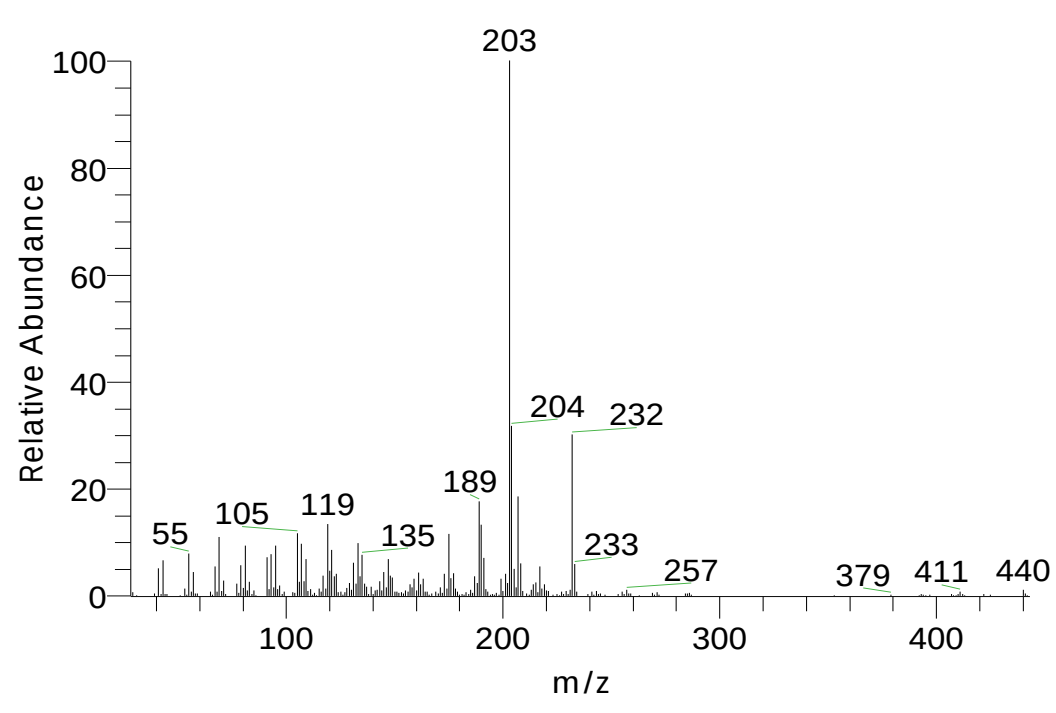

My GC-MS Report

Compound Structure | Hit Spectrum

Betulinaldehyde
Formula C30H48O2, MW 440, CAS# 13159-28-9, Entry# 175929
$:28FELCJAPFJOPHSD-UHFFFAOYSA-N

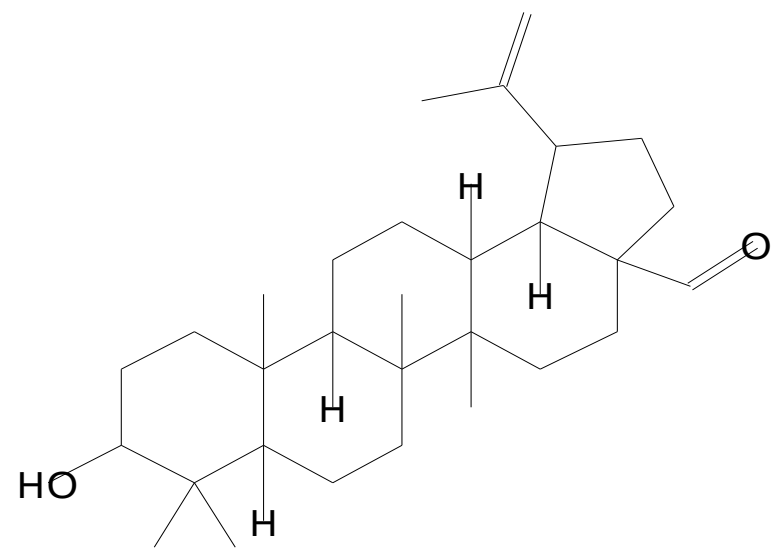

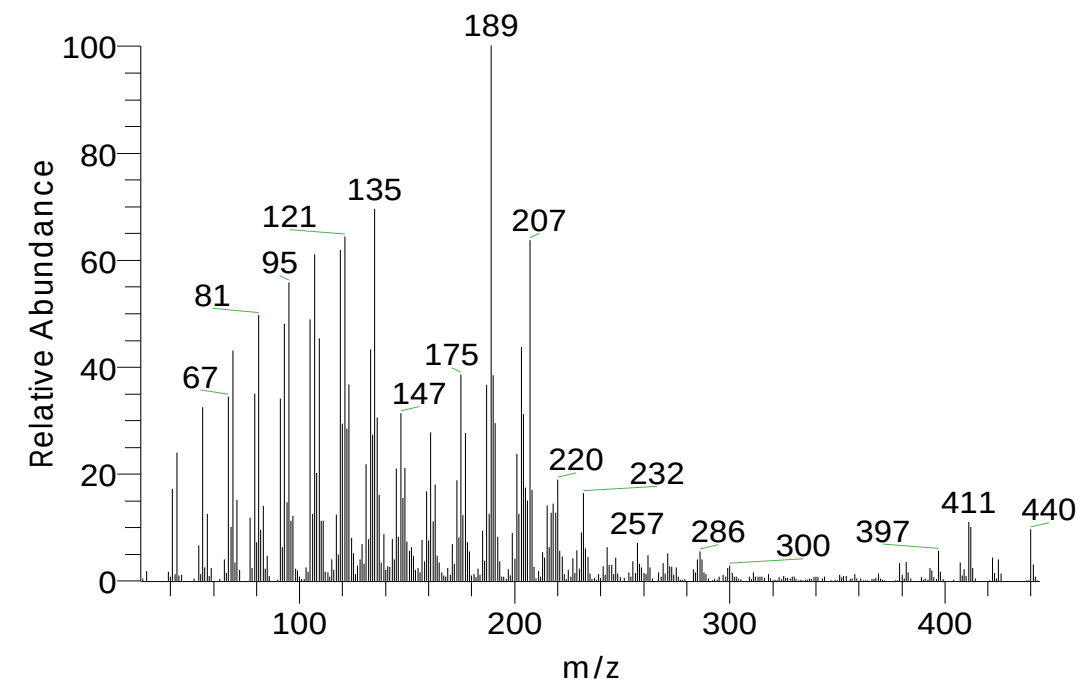

Urs-12-en-28-al, 3-(acetyloxy)-, (3á)-
Formula C32H50O3, MW 482, CAS# 86996-88-5, Entry# 186162
28-Oxours-12-en-3-yl acetate #

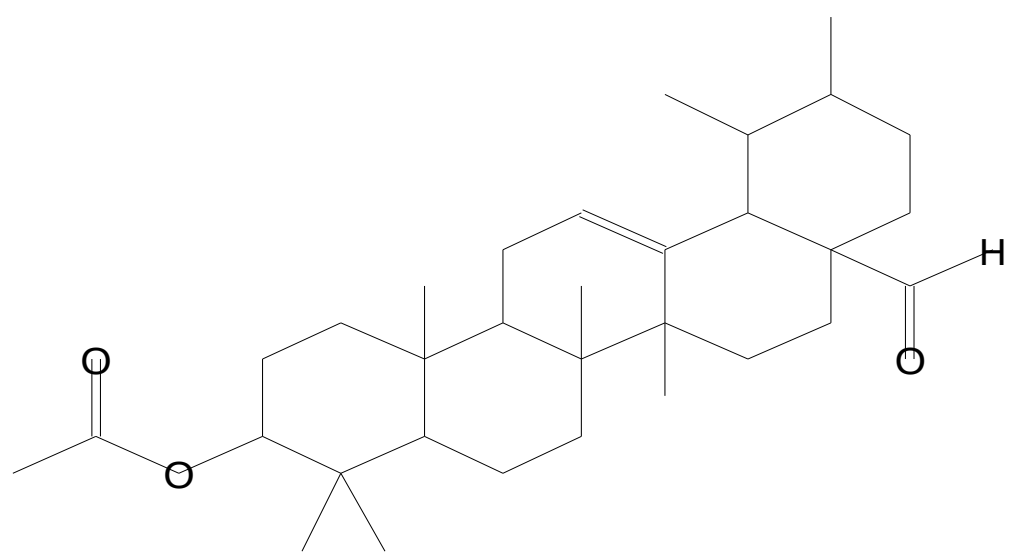

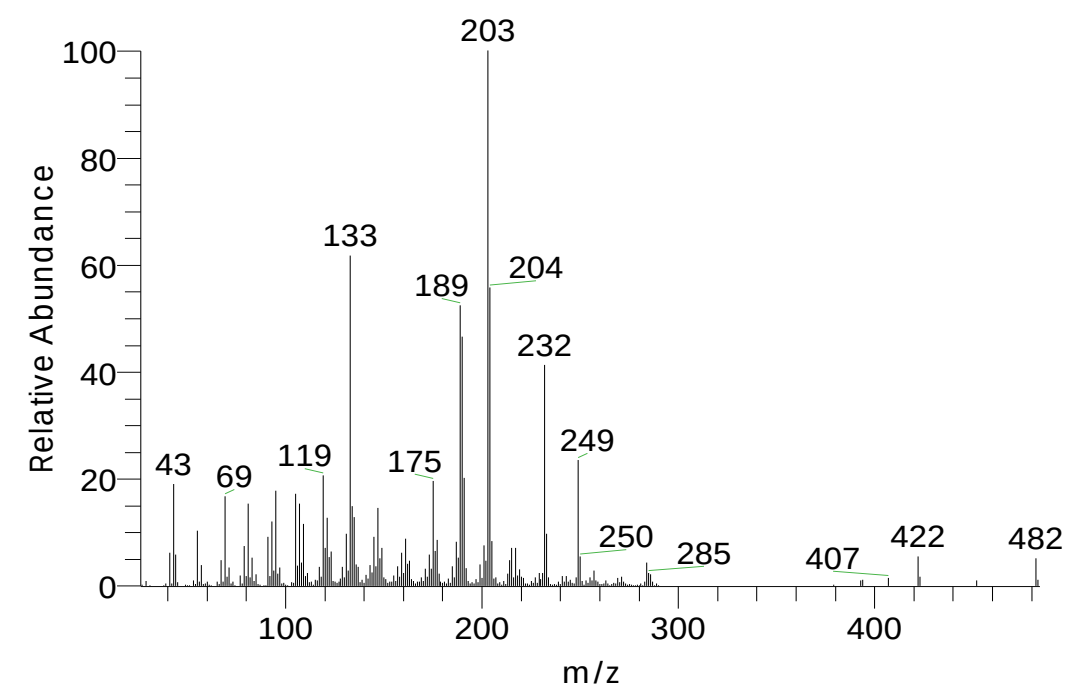

28-OXOURS-12-EN-3-YL ACETATE #
Formula C32H50O3, MW 482, CAS# 86996-88-5, Entry# 374690
28-OXOURS-12-EN-3-YL ACETATE

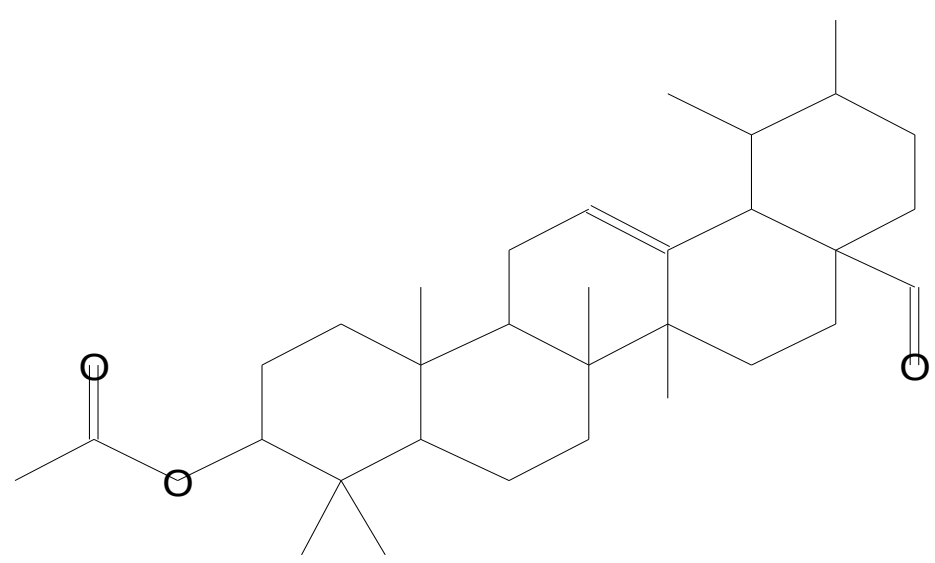

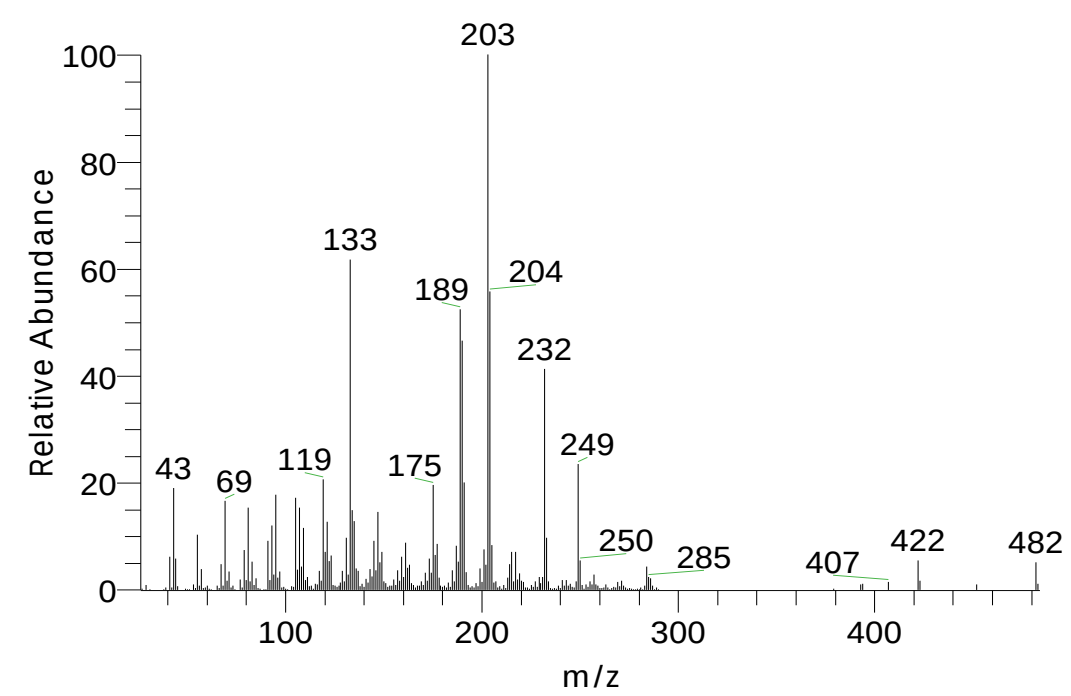

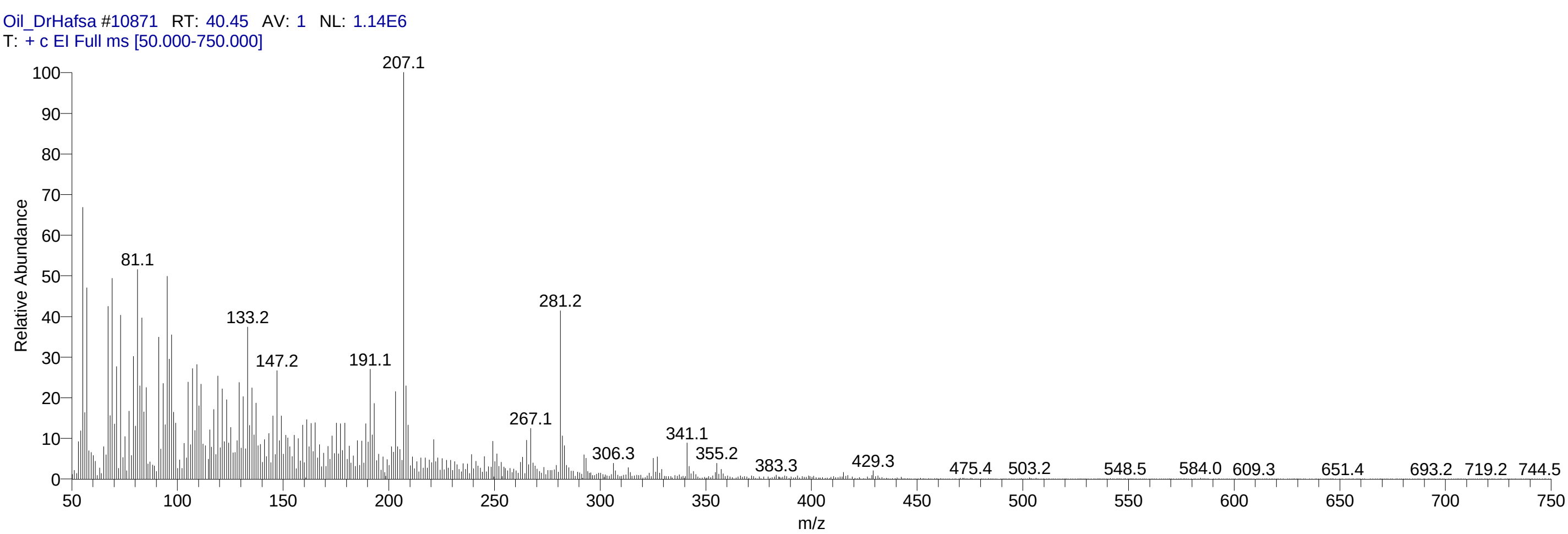

RT	Compound Name	Area %	MF	Molecular Formula	Molecular Weight	Cas #	Library
40.45	Ethyl iso-allocholate	0.32	775	C26H44O5	436	NA	mainlib
40.45	ETHYL ISO-ALLOCHOLATE	0.32	775	C26H44O5	436	NA	WileyRegistry8e
40.45	03027205002 FLAVONE 4'-OH,5-OH,7-DI-O-GLUCOSIDE	0.32	746	C27H30O15	594	NA	WileyRegistry8e

My GC-MS Report

RT	Compound Name	Area %	MF	Molecular Formula	Molecular Weight	Cas #	Library
40.45	9,12,15-OCTADECATRIENOIC ACID, 2,3-BIS[(TRIMETHYLSILYL)OXY]PROPYL ESTER, (Z,Z,Z)-	0.32	795	C27H52O4Si2	496	55521-22-7	WileyRegistry8e
40.45	7,8-Epoxylanostan-11-ol, 3-acetoxy-	0.32	736	C32H54O4	502	NA	mainlib

Compound Structure | Hit Spectrum

Ethyl iso-allocholate
Formula C26H44O5, MW 436, CAS# NA, Entry# 7020
Ethyl 3,7,12-trihydroxycholan-24-oate #

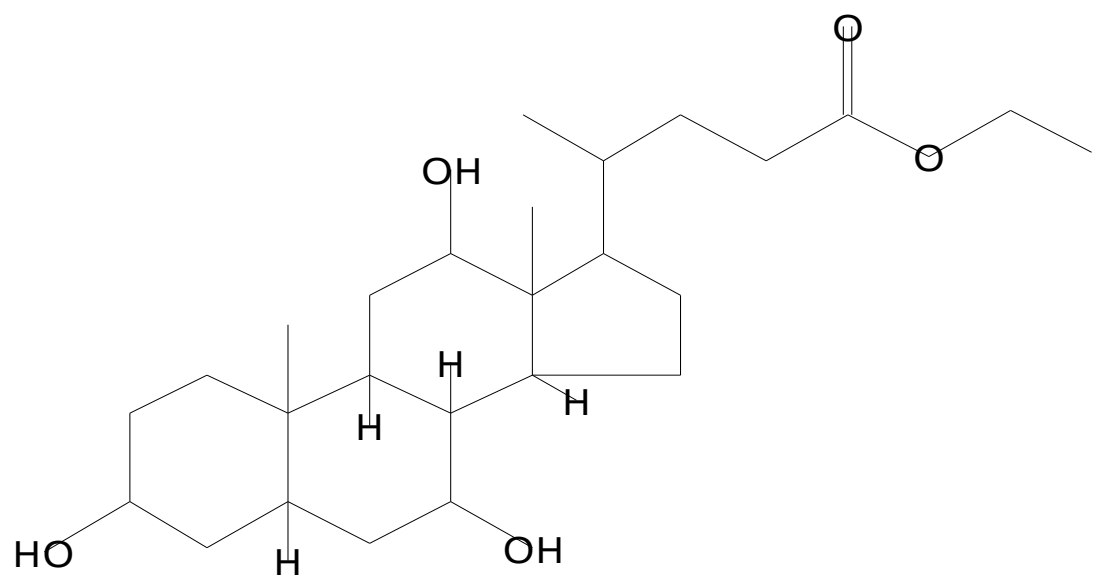

SI 763, RSI 775, mainlib, Entry# 7020, CAS# NA, Ethyl iso-allocholate

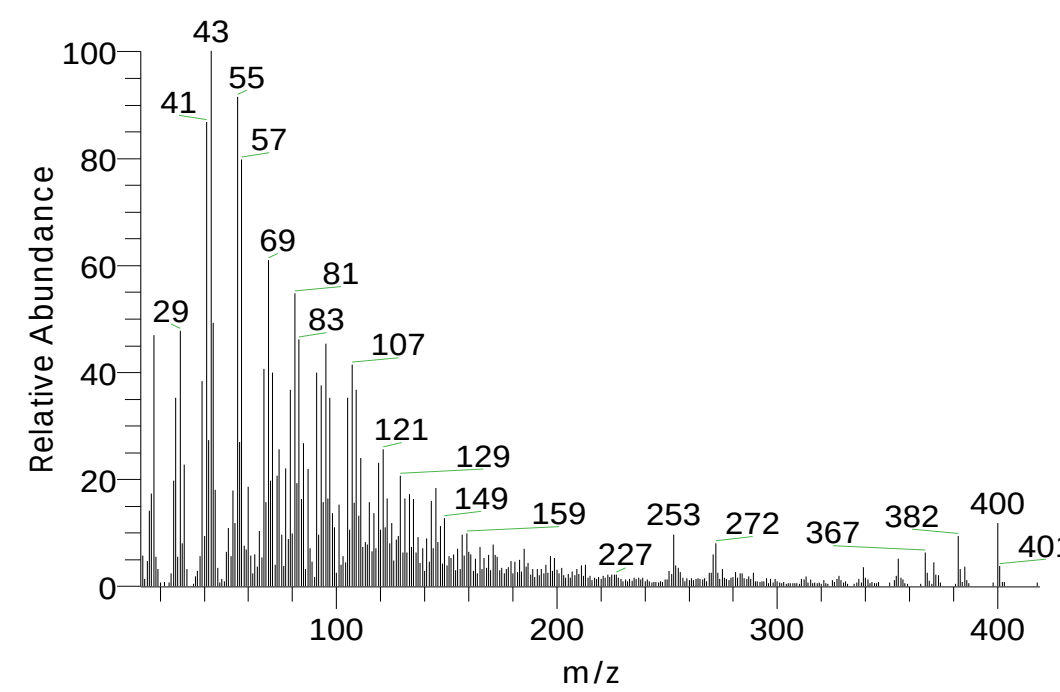

ETHYL ISO-ALLOCHOLATE
Formula C26H44O5, MW 436, CAS# NA, Entry# 270212

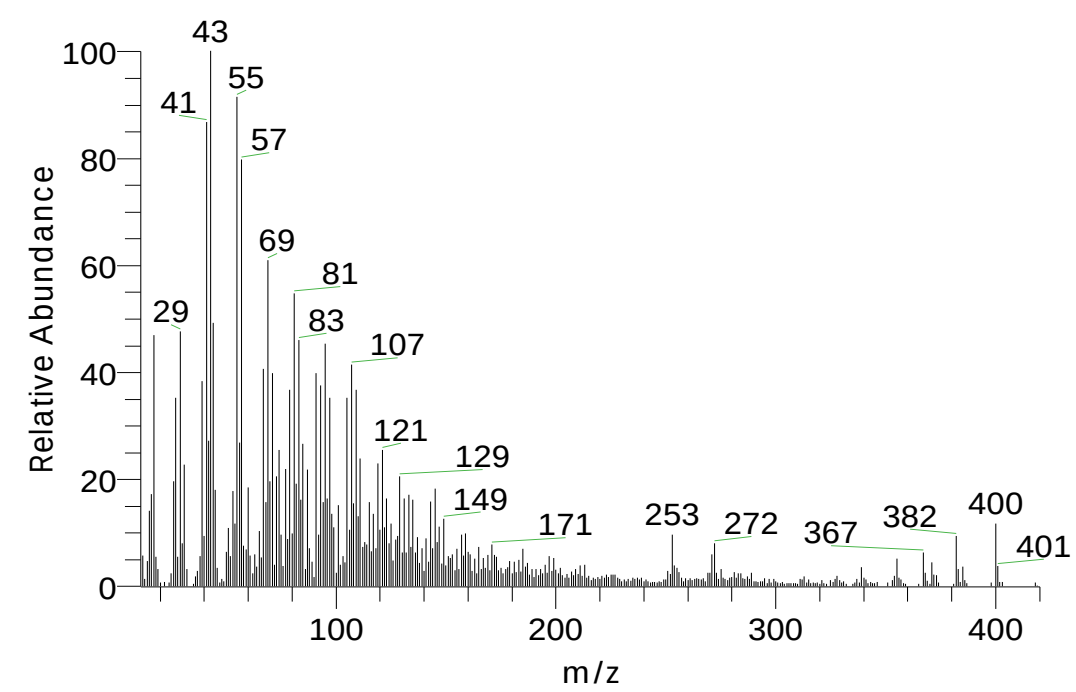

03027205002 FLAVONE 4'-OH,5-OH,7-DI-O-GLUCOSIDE
Formula C27H30O15, MW 594, CAS# NA, Entry# 296184

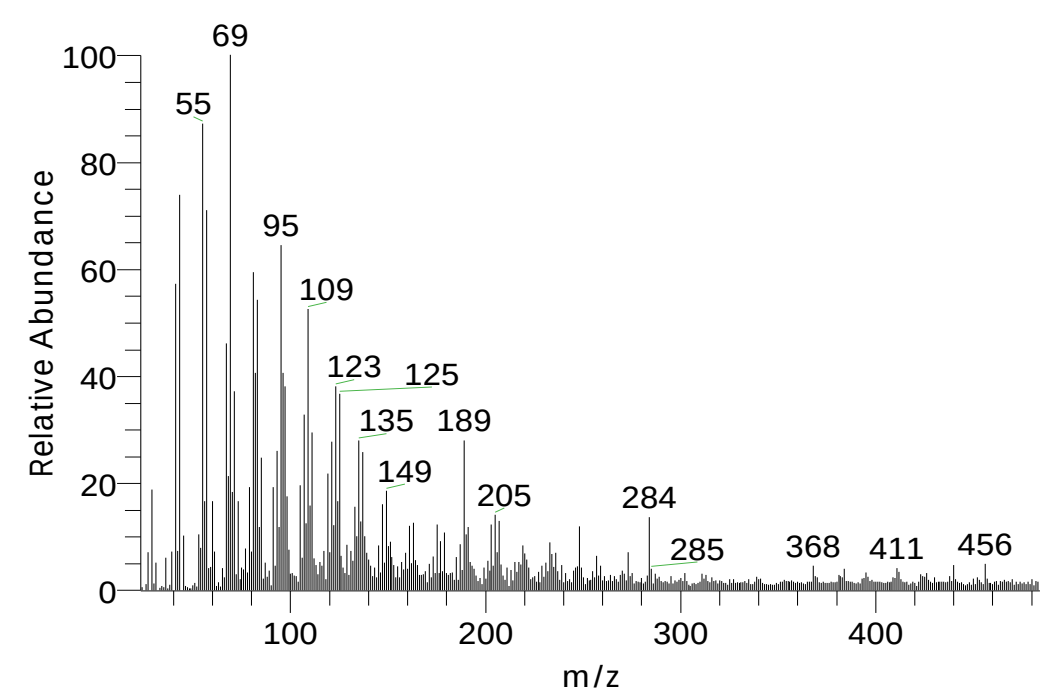

9,12,15-OCTADECATRIENOIC ACID, 2,3-BIS[(TRIMETHYLSILYL)OXY]PROPYL ESTER, (Z,Z,Z)-
Formula C27H52O4Si2, MW 496, CAS# 55521-22-7, Entry# 284834
2,3-BIS[(TRIMETHYLSILYL)OXY]PROPYL (9E,12E,15E)-9,12,15-OCTADECATRIENOATE #

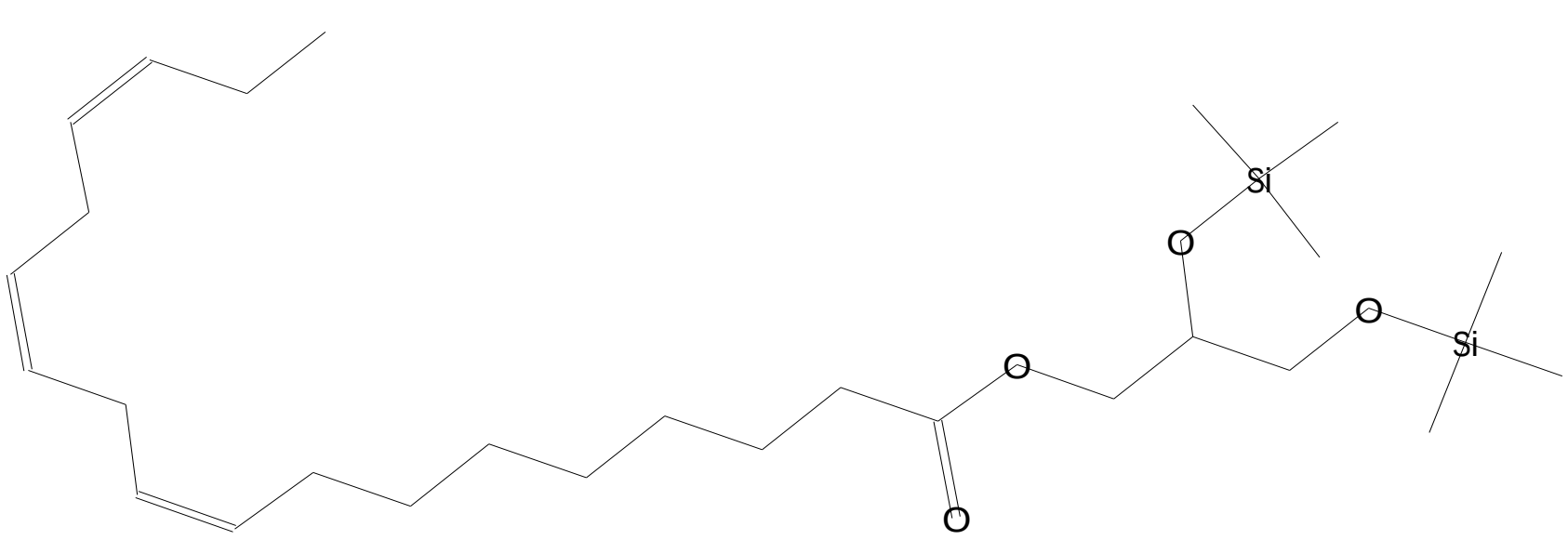

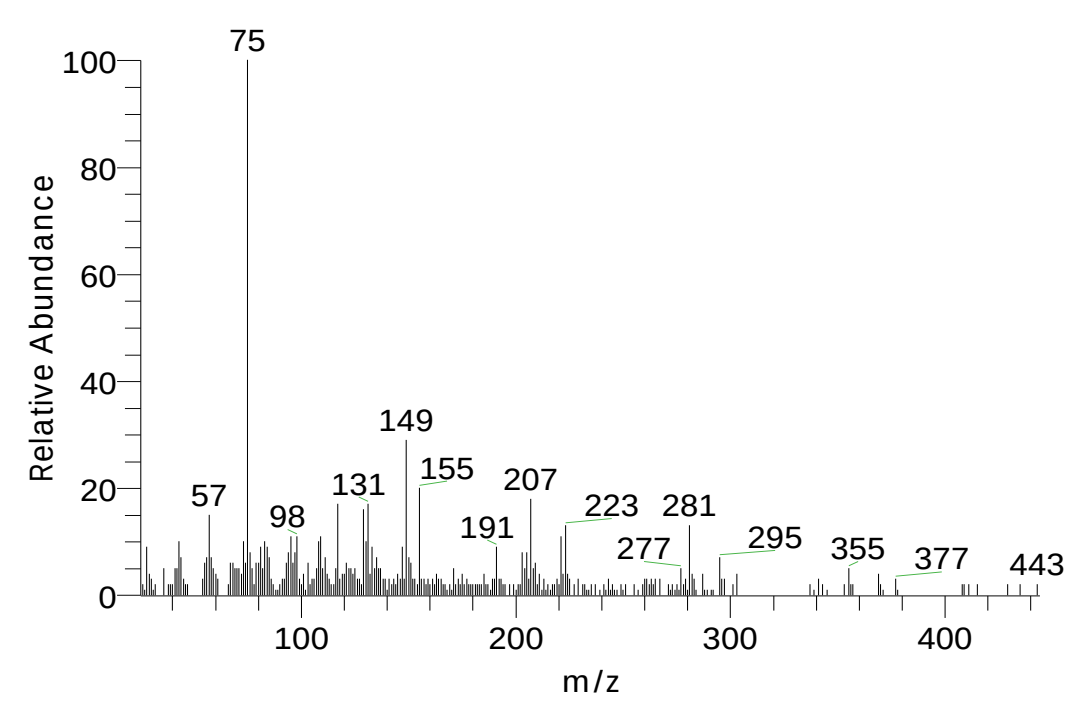

My GC-MS Report

Compound Structure | Hit Spectrum

7,8-Epoxylanostan-11-ol, 3-acetoxy-
Formula C32H54O4, MW 502, CAS# NA, Entry# 15359

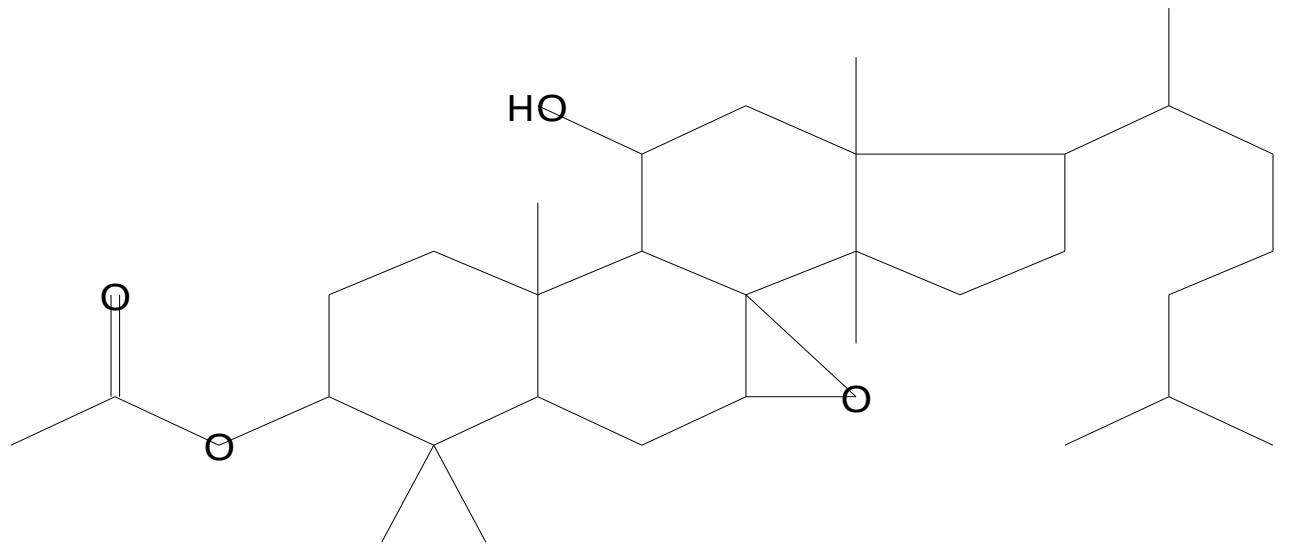

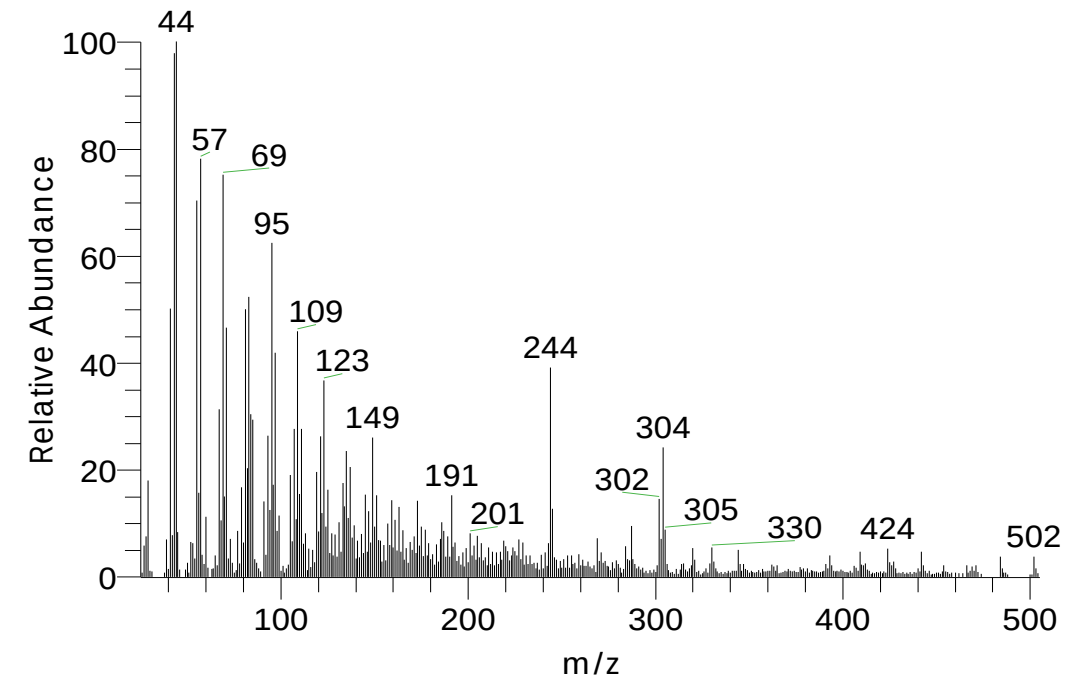

Oil_DrHafsa #10923 RT: 40.63 AV: 1 NL: 1.31E6
T: + c EI Full ms [50.000-750.000]

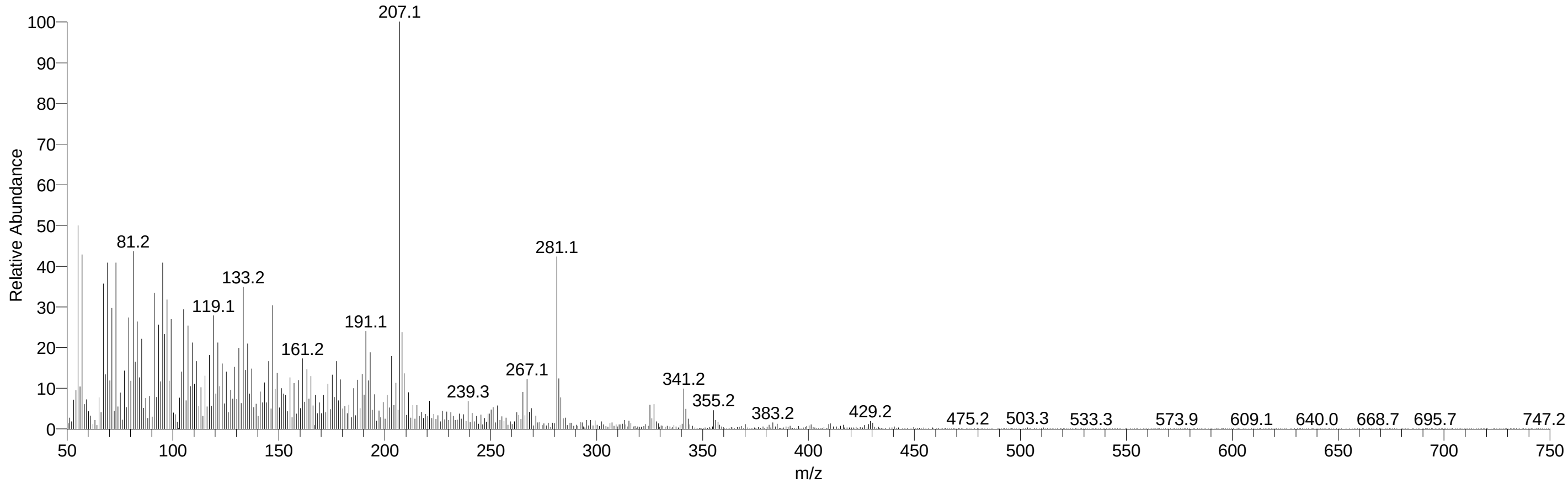

RT	Compound Name	Area %	MF	Molecular Formula	Molecular Weight	Cas #	Library
40.63	03027205002 FLAVONE 4'-OH,5-OH,7-DI-O-GLUCOSIDE	0.51	740	C27H30O15	594	NA	WileyRegistry8e
40.63	9,12,15-OCTADECATRIENOIC ACID, 2,3-BIS[(TRIMETHYLSILYL)OXY]PROPYL ESTER, (Z,Z,Z)-	0.51	781	C27H52O4Si2	496	55521-22-7	WileyRegistry8e
40.63	1-Heptatriacotanol	0.51	790	C37H76O	536	105794-58-9	mainlib
40.63	Rhodopin	0.51	719	C40H58O	554	105-92-0	mainlib
40.63	.PSI.,.PSI.-CAROTENE, 1,2-DIHYDRO-1-HYDROXY-	0.51	716	C40H58O	554	105-92-0	WileyRegistry8e

Compound Structure | Hit Spectrum

03027205002 FLAVONE 4'-OH,5-OH,7-DI-O-GLUCOSIDE
Formula C27H30O15, MW 594, CAS# NA, Entry# 296184

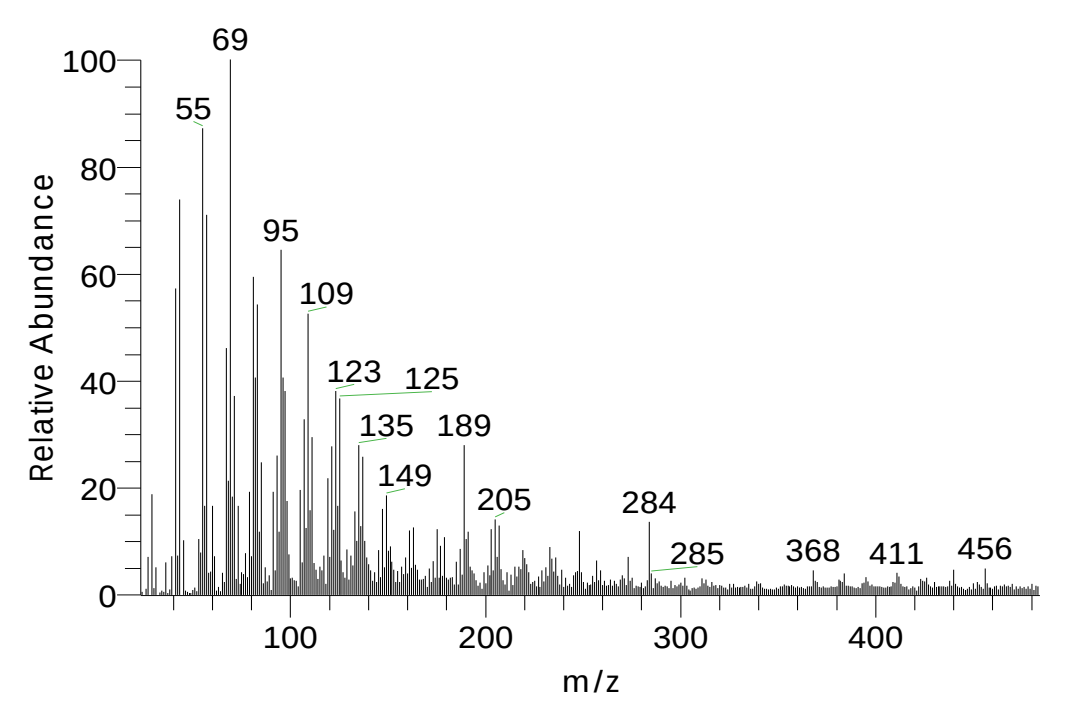

My GC-MS Report

Compound Structure	Hit Spectrum

9,12,15-OCTADECATRIENOIC ACID, 2,3-BIS[(TRIMETHYLSILYL)OXY]PROPYL ESTER, (Z,Z,Z)-
Formula C27H52O4Si2, MW 496, CAS# 55521-22-7, Entry# 284834
2,3-BIS[(TRIMETHYLSILYL)OXY]PROPYL (9E,12E,15E)-9,12,15-OCTADECATRIENOATE #

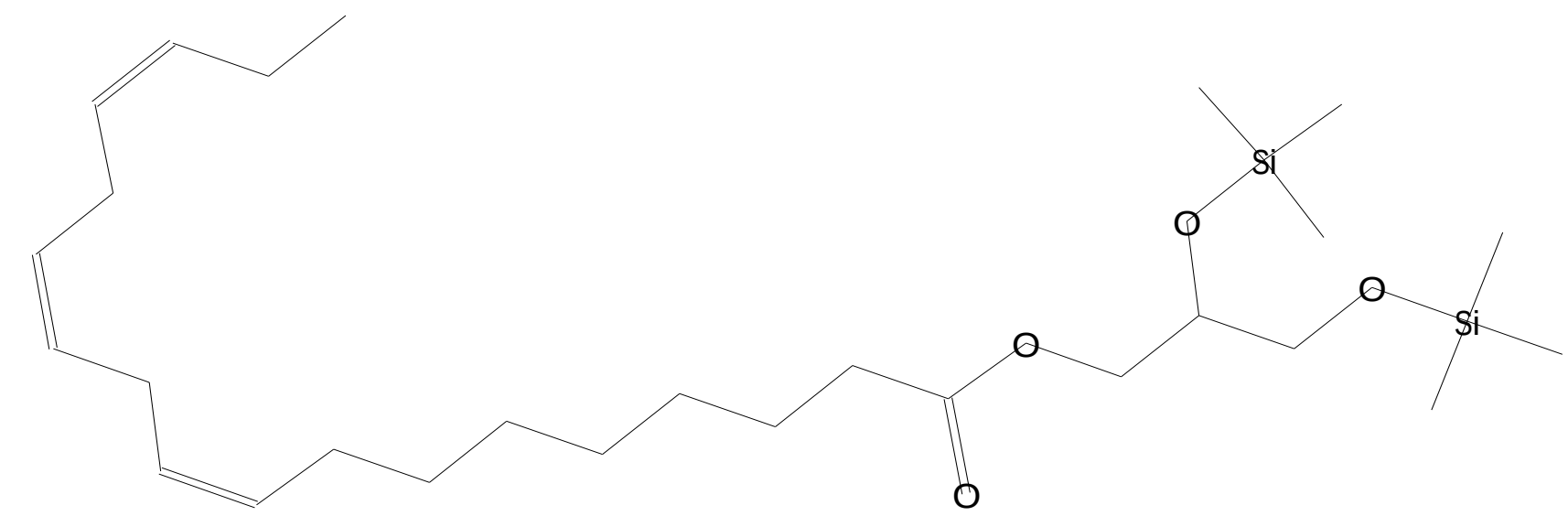

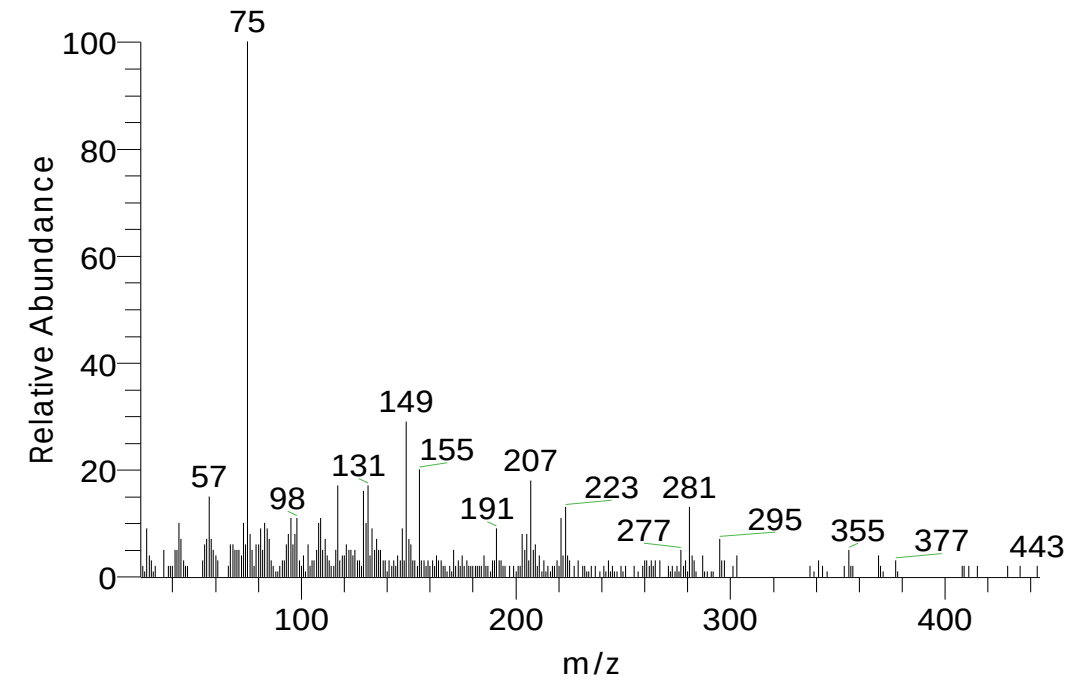

1-Heptatriacotanol
Formula C37H76O, MW 536, CAS# 105794-58-9, Entry# 7279
1-Heptatriacontanol #

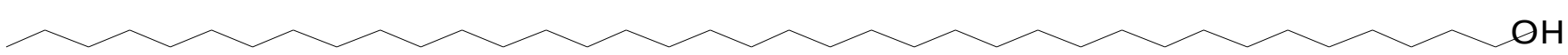

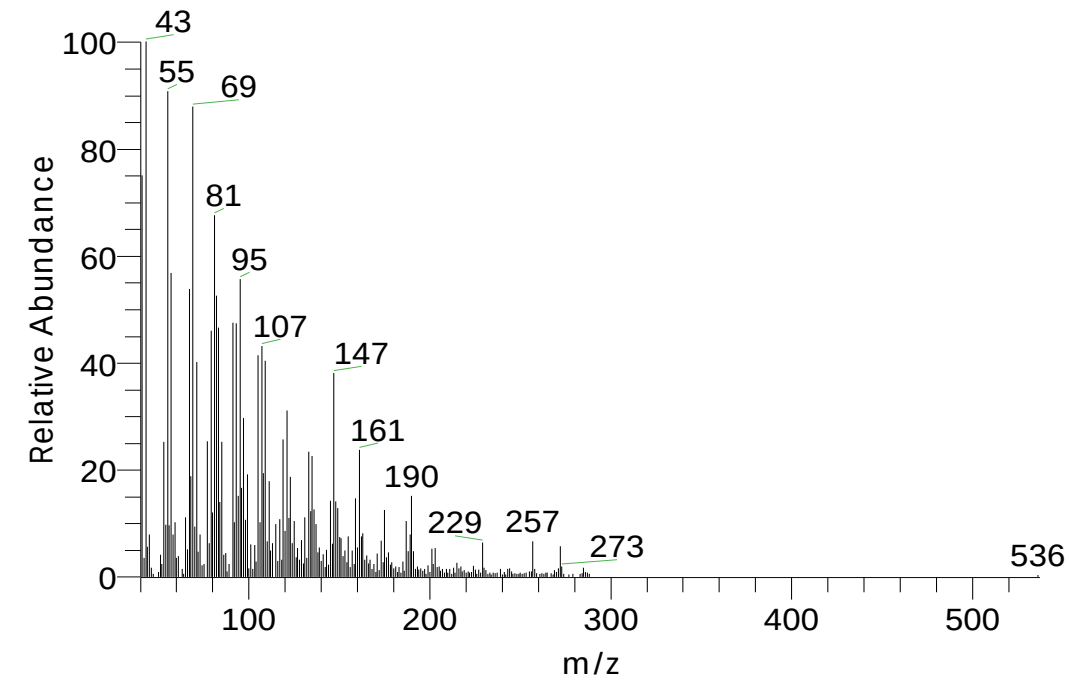

Rhodopin
Formula C40H58O, MW 554, CAS# 105-92-0, Entry# 34358
.psi.,.psi.-Carotene, 1,2-dihydro-1-hydroxy-

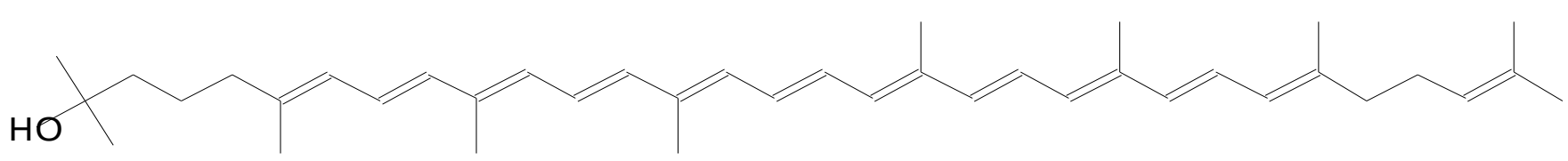

SI 717, RSI 719, mainlib, Entry# 34358, CAS# 105-92-0, Rhodopin

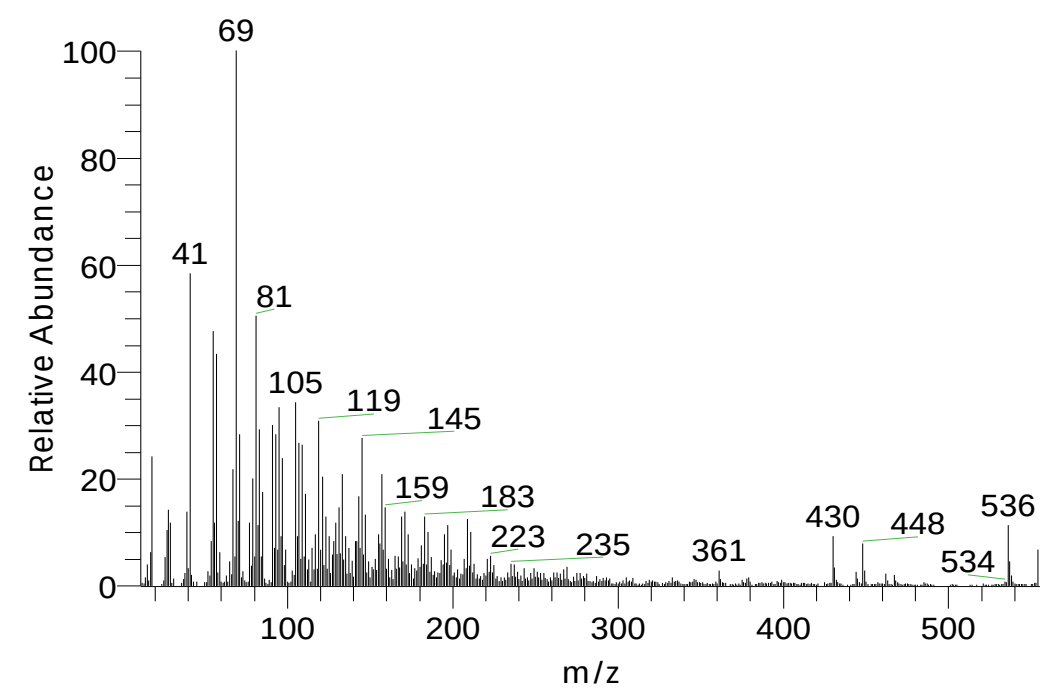

.PSI.,.PSI.-CAROTENE, 1,2-DIHYDRO-1-HYDROXY-
Formula C40H58O, MW 554, CAS# 105-92-0, Entry# 292854
1,2-DIHYDRO-PSI,PSI-CAROTENE #

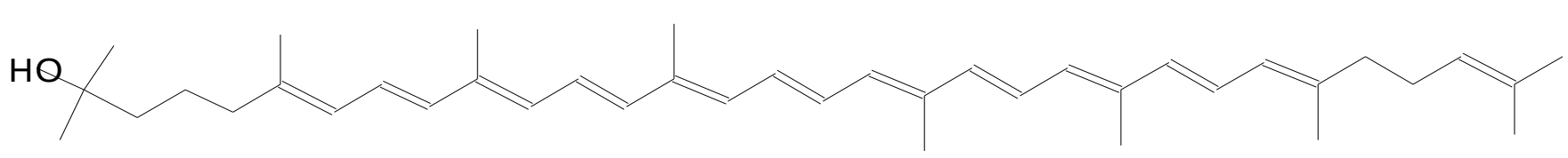

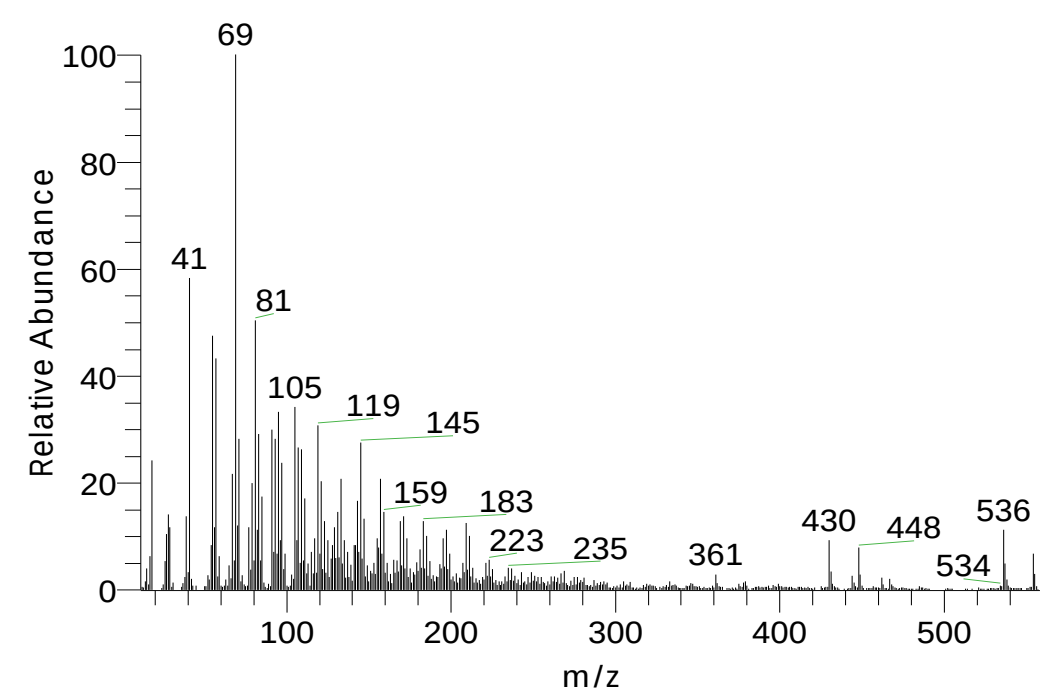

My GC-MS Report

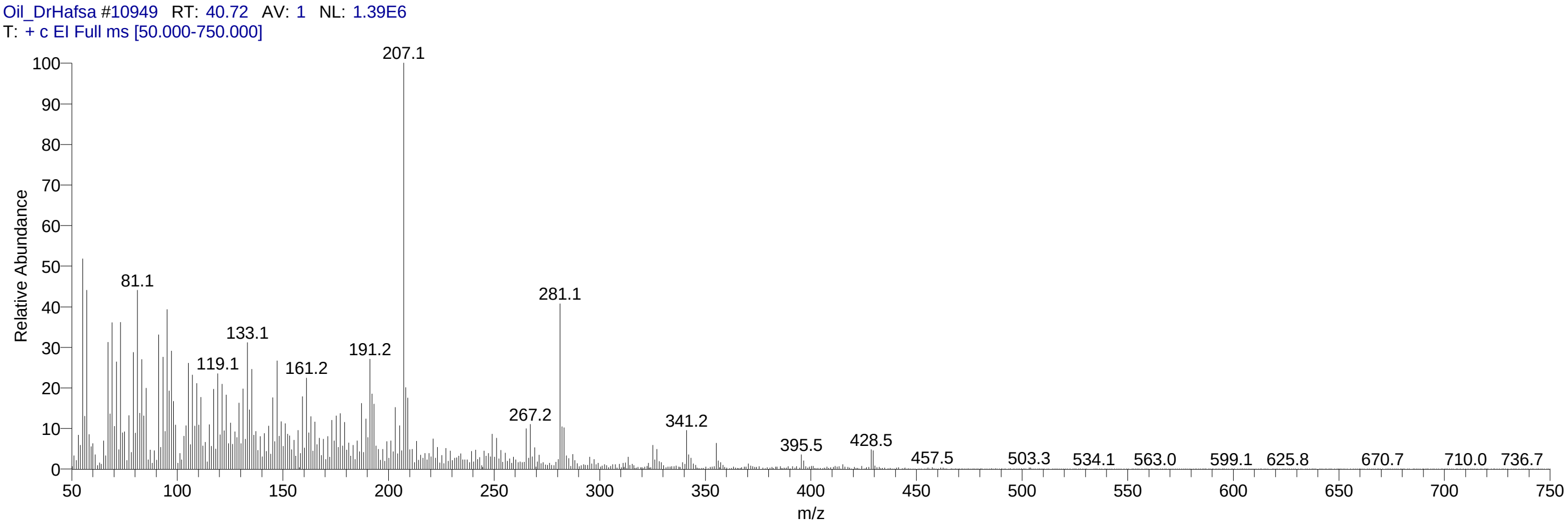

RT	Compound Name	Area %	MF	Molecular Formula	Molecular Weight	Cas #	Library
40.72	03027205002 FLAVONE 4'-OH,5-OH,7-DI-O-GLUCOSIDE	1.02	739	C27H30O15	594	NA	WileyRegistry8e
40.72	9,19-Cyclolanostan-3-ol, 24,24-epoxymethano-, acetate	1.02	734	C33H54O3	498	NA	mainlib
40.72	1-[3-(2-ISOPROPYL-2-OXIRANYL)-1-METHYLPROPYL]-3A,6,6,12A-TETRAMETHYLTETRADECAHYDRO-1H-CYCLOPENTA[A]CYCLOPROPA[E]PHENANTHREN-7-YL ACETATE	1.02	734	C33H54O3	498	NA	WileyRegistry8e
40.72	Rhodopin	1.02	718	C40H58O	554	105-92-0	mainlib
40.72	.PSI.,.PSI.-CAROTENE, 1,2-DIHYDRO-1-HYDROXY-	1.02	715	C40H58O	554	105-92-0	WileyRegistry8e

Compound Structure	Hit Spectrum

03027205002 FLAVONE 4'-OH,5-OH,7-DI-O-GLUCOSIDE
Formula C27H30O15, MW 594, CAS# NA, Entry# 296184

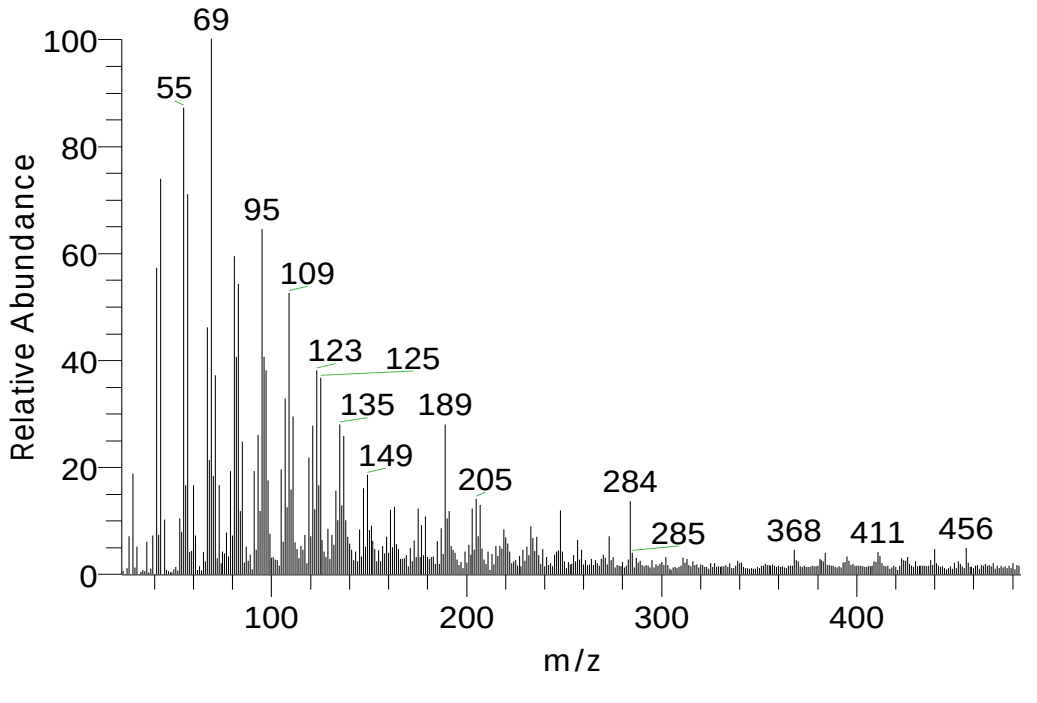

9,19-Cyclolanostan-3-ol, 24,24-epoxymethano-, acetate
Formula C33H54O3, MW 498, CAS# NA, Entry# 24072

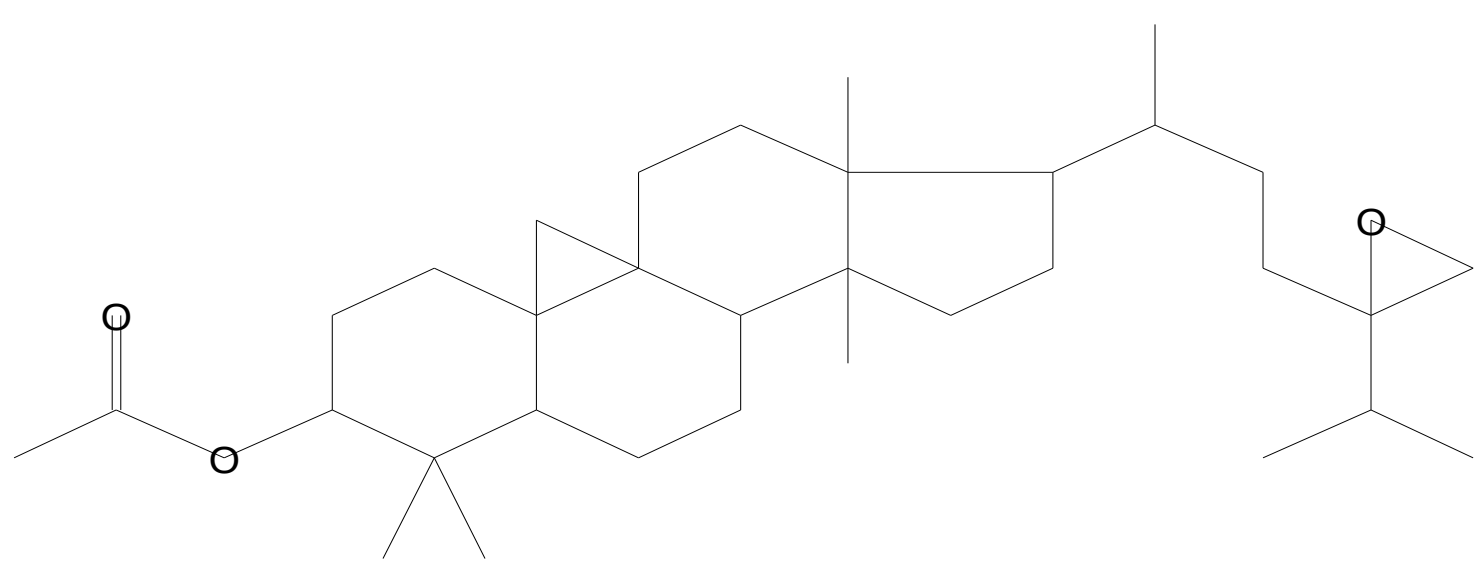

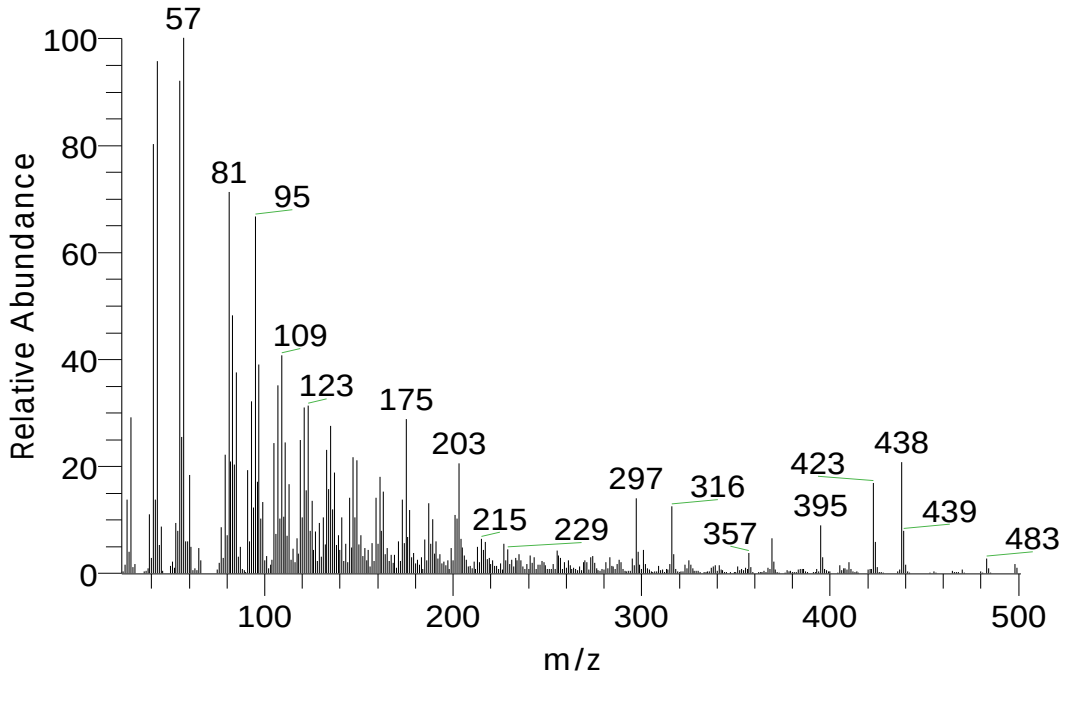

My GC-MS Report

Compound Structure | Hit Spectrum

Formula C33H54O3, MW 498, CAS# NA, Entry# 371150

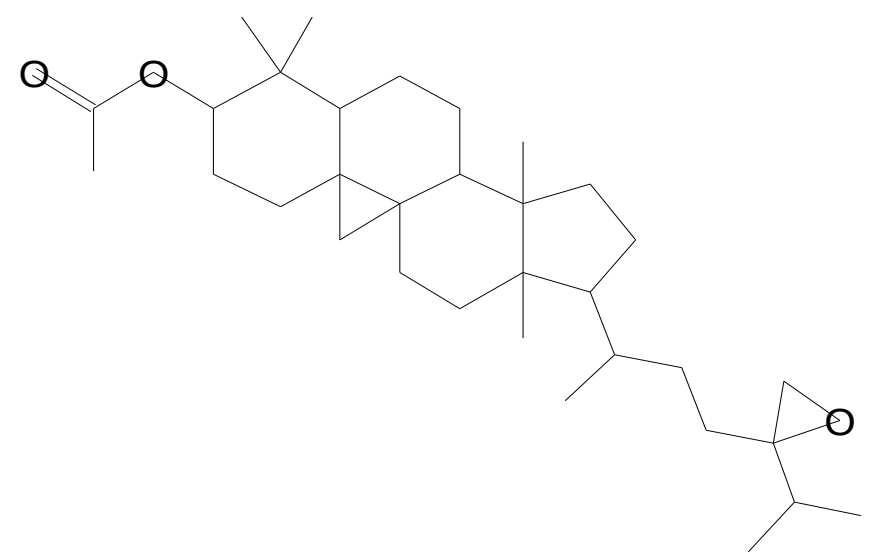

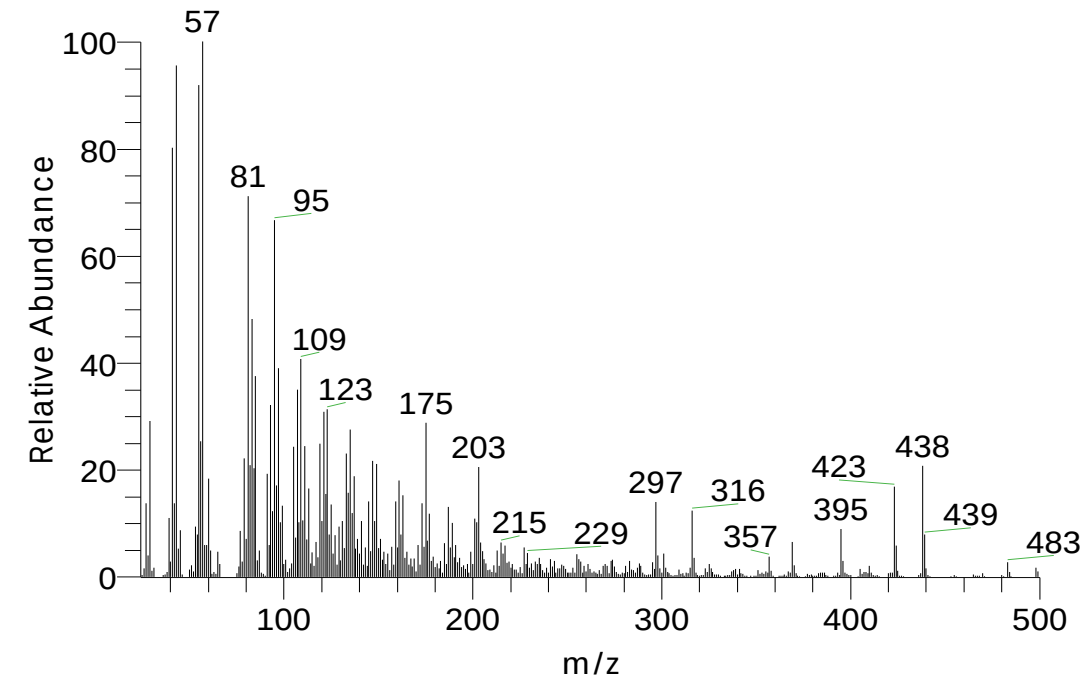

SI 716, RSI 718, mainlib, Entry# 34358, CAS# 105-92-0, Rhodopin

Rhodopin
Formula C40H58O, MW 554, CAS# 105-92-0, Entry# 34358
.psi.,.psi.-Carotene, 1,2-dihydro-1-hydroxy-

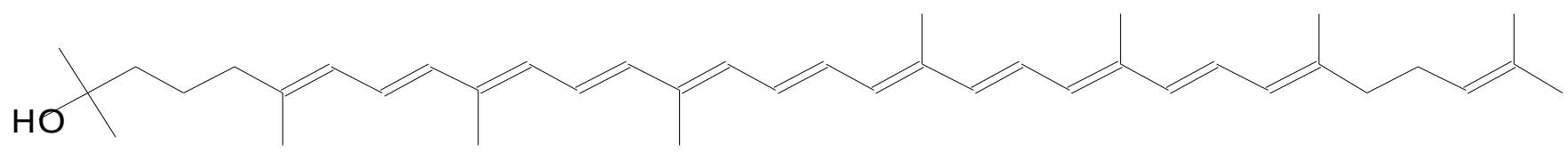

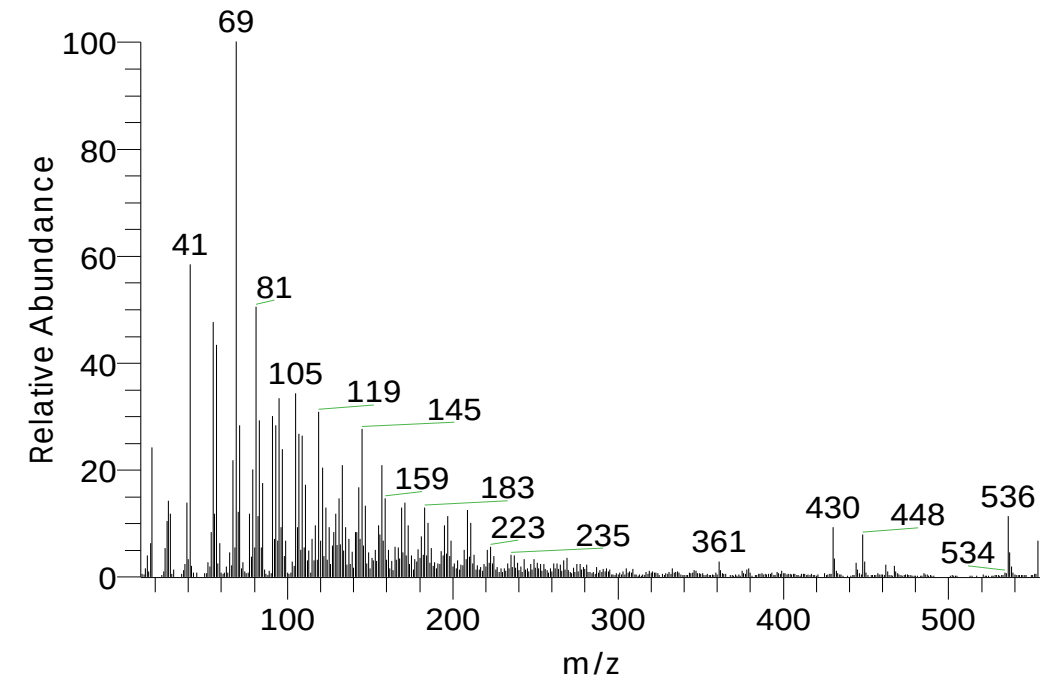

.PSI.,.PSI.-CAROTENE, 1,2-DIHYDRO-1-HYDROXY-
Formula C40H58O, MW 554, CAS# 105-92-0, Entry# 292854
1,2-DIHYDRO-PSI,PSI-CAROTENE #

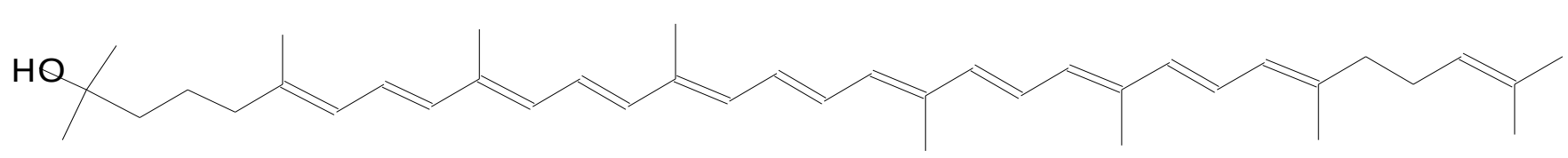

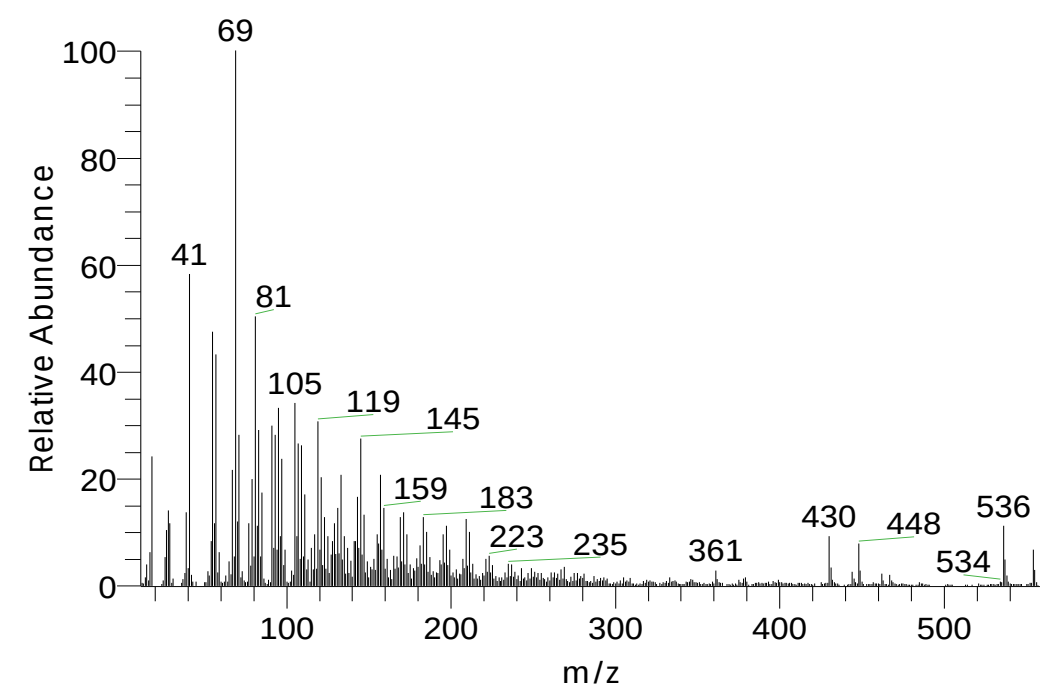

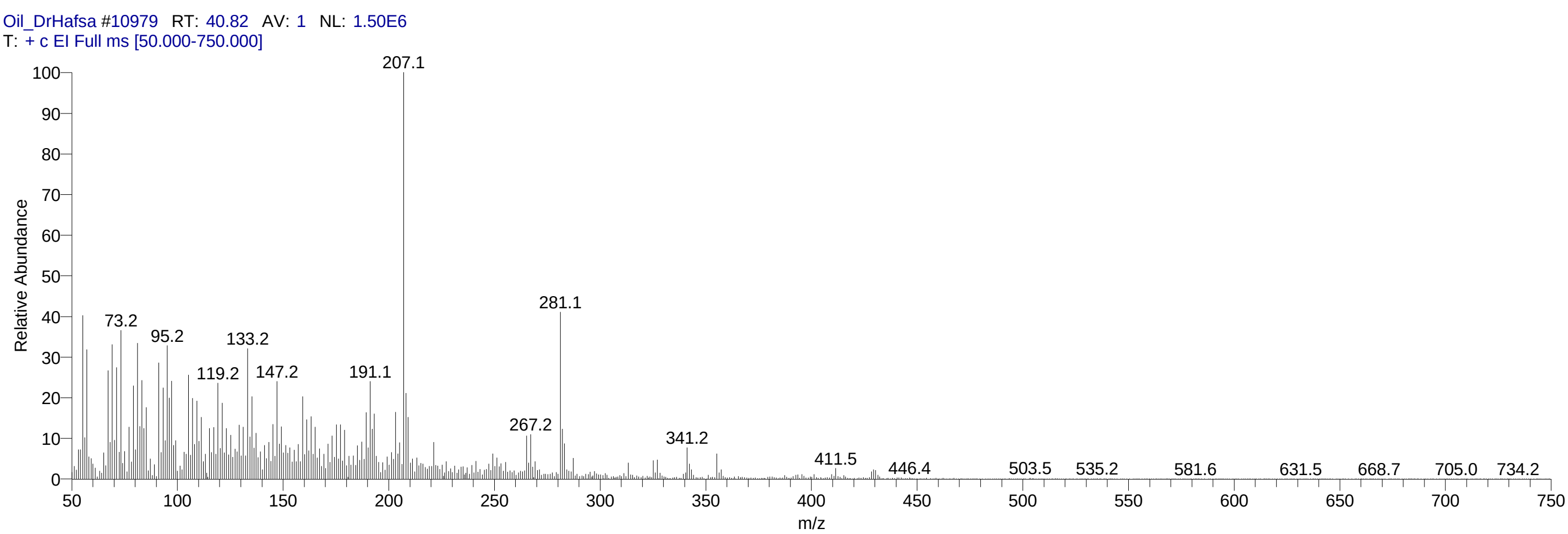

RT	Compound Name	Area %	MF	Molecular Formula	Molecular Weight	Cas #	Library
40.82	.psi.,.psi.-Carotene, 1,1',2,2'-tetrahydro-1,1'-dimethoxy-	0.19	728	C42H64O2	600	13833-01-7	mainlib
40.82	.PSI.,.PSI.-CAROTENE, 1,1',2,2'-TETRAHYDRO-1,1'-DIMETHOXY-	0.19	727	C42H64O2	600	13833-01-7	WileyRegistry8e

My GC-MS Report

RT	Compound Name	Area %	MF	Molecular Formula	Molecular Weight	Cas #	Library
40.82	9,12,15-OCTADECATRIENOIC ACID, 2,3-BIS[(TRIMETHYLSILYL)OXY]PROPYL ESTER, (Z,Z,Z)-	0.19	780	C27H52O4Si2	496	55521-22-7	WileyRegistry8e
40.82	9-OCTADECENOIC ACID (Z)-, 2-[(TRIMETHYLSILYL)OXY]-1-[[(TRIMETHYLSILYL)OXY]METHYL]ETHYL ESTER	0.19	749	C27H56O4Si2	500	54284-48-9	WileyRegistry8e
40.82	9,12-OCTADECADIENOIC ACID (Z,Z)-, 2,3-BIS[(TRIMETHYLSILYL)OXY]PROPYL ESTER	0.19	767	C27H54O4Si2	498	54284-45-6	WileyRegistry8e

Compound Structure | Hit Spectrum

.psi.,.psi.-Carotene, 1,1',2,2'-tetrahydro-1,1'-dimethoxy-
Formula C42H64O2, MW 600, CAS# 13833-01-7, Entry# 41205
Lycopene, 1,1',2,2'-tetrahydro-1,1'-dimethoxy-, all-trans-

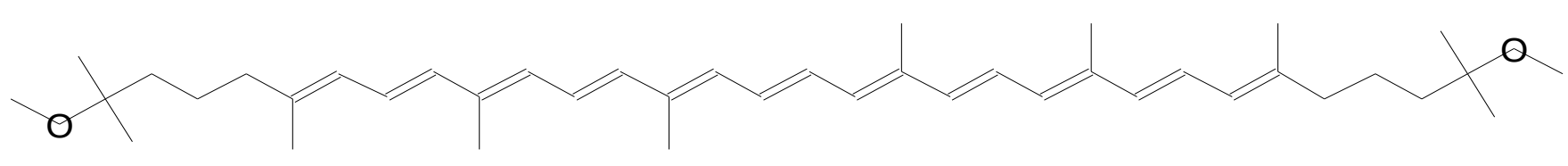

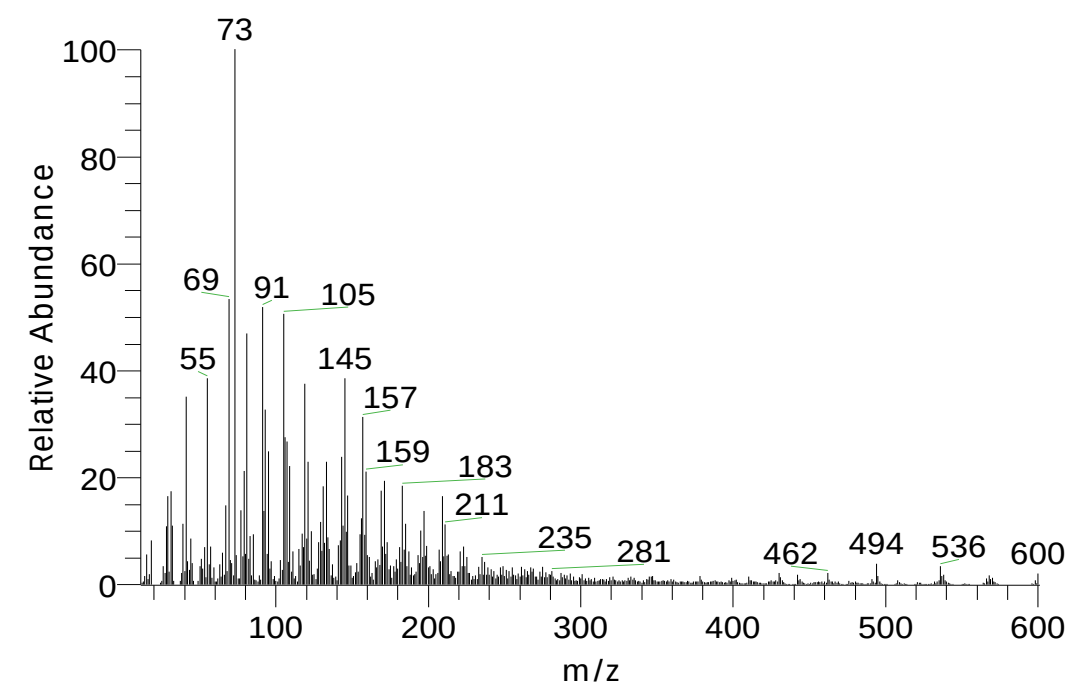

.PSI.,.PSI.-CAROTENE, 1,1',2,2'-TETRAHYDRO-1,1'-DIMETHOXY-
Formula C42H64O2, MW 600, CAS# 13833-01-7, Entry# 296796
1,1',2,2'-TETRAHYDRO-PSI,PSI-CAROTENE #

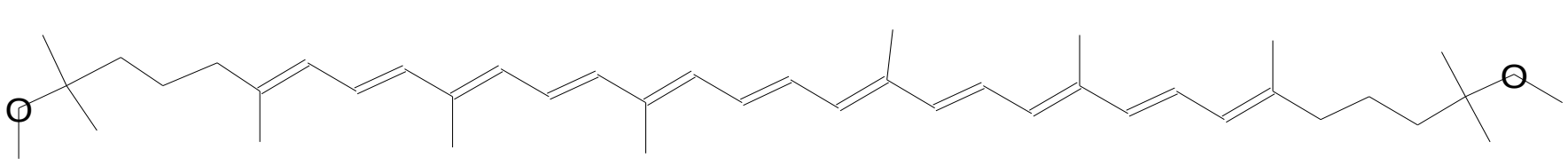

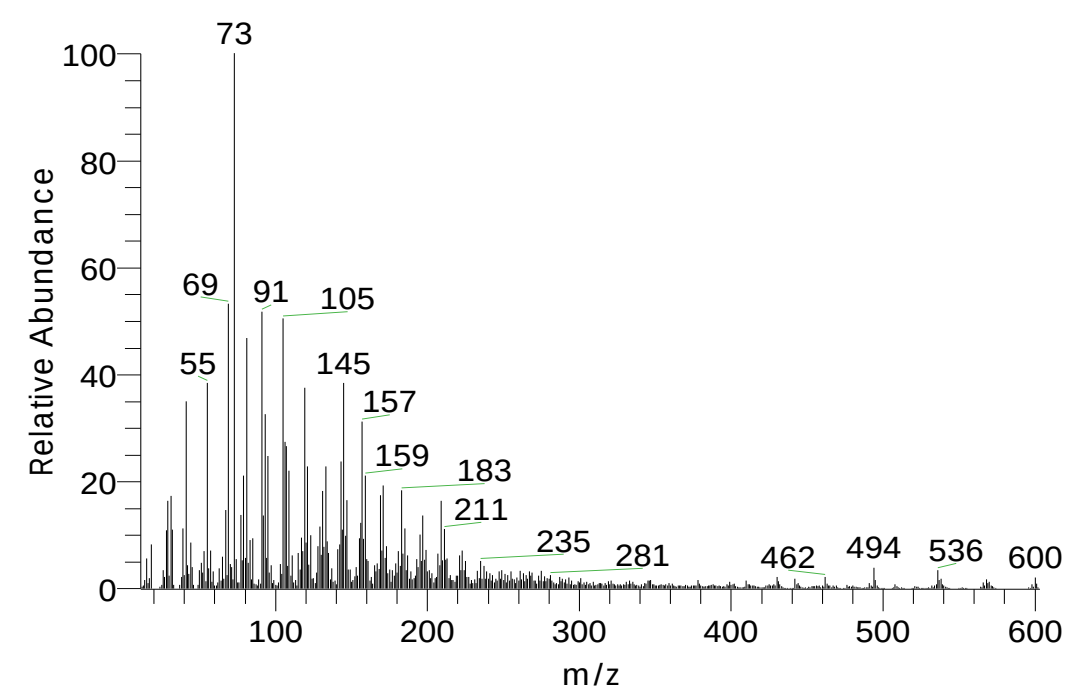

9,12,15-OCTADECATRIENOIC ACID, 2,3-BIS[(TRIMETHYLSILYL)OXY]PROPYL ESTER, (Z,Z,Z)-
Formula C27H52O4Si2, MW 496, CAS# 55521-22-7, Entry# 284834
2,3-BIS[(TRIMETHYLSILYL)OXY]PROPYL (9E,12E,15E)-9,12,15-OCTADECATRIENOATE #

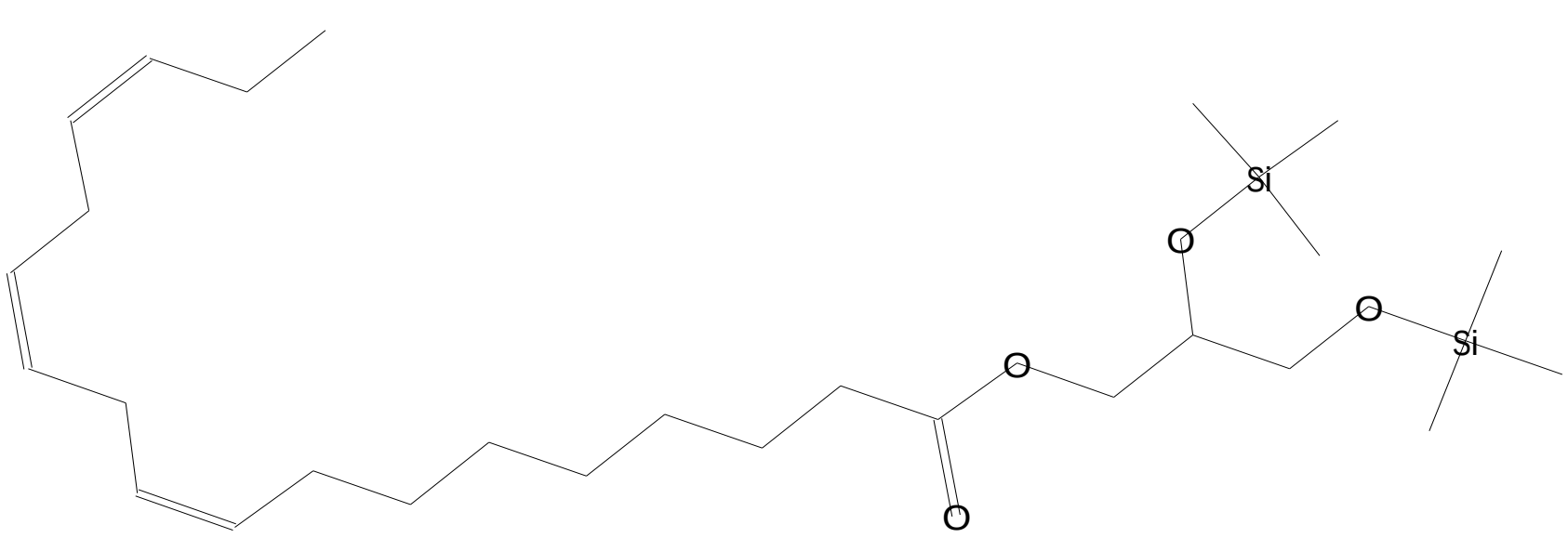

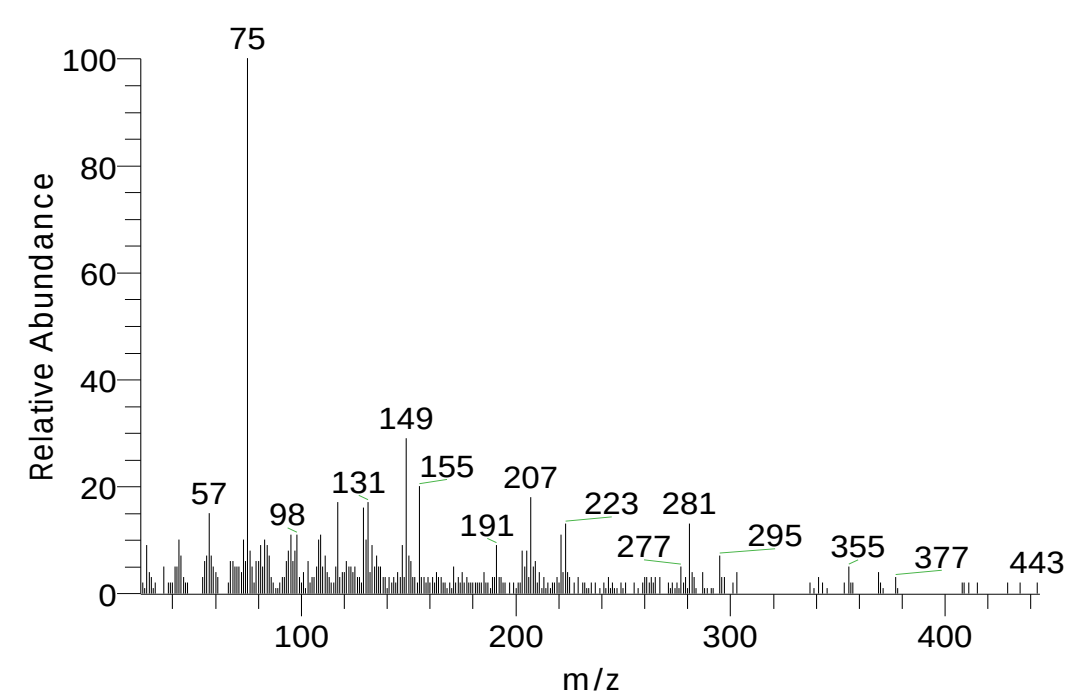

My GC-MS Report

Compound Structure | Hit Spectrum

Formula C27H56O4Si2, MW 500, CAS# 54284-48-9, Entry# 285456
2-MONOOLEOYLGLYCEROL TRIMETHYLSILYL ETHER

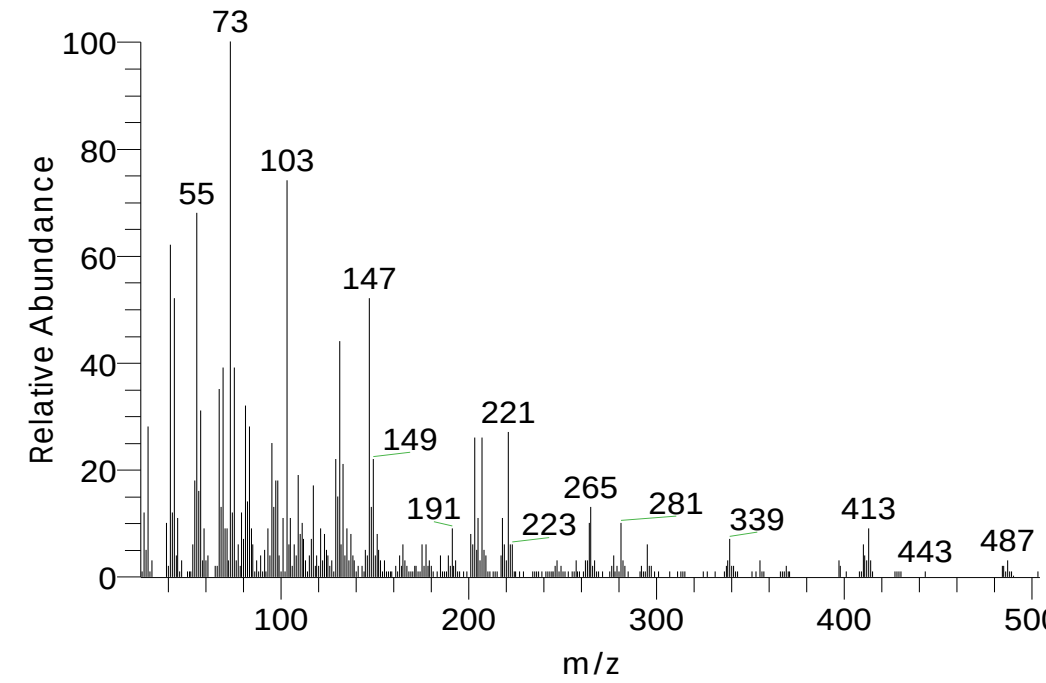

9,12-OCTADECADIENOIC ACID (Z,Z)-, 2,3-BIS[(TRIMETHYLSILYL)OXY]PROPYL ESTER
Formula C27H54O4Si2, MW 498, CAS# 54284-45-6, Entry# 285148
2,3-BIS[(TRIMETHYLSILYL)OXY]PROPYL (9Z,12Z)-9,12-OCTADECADIENOATE #

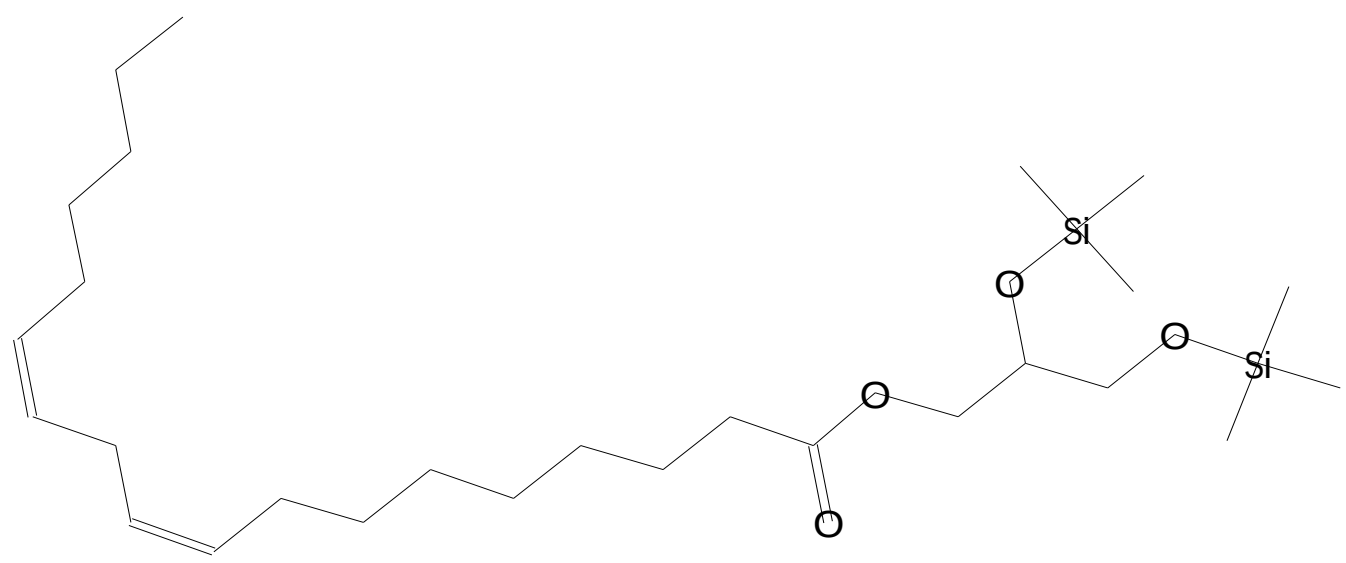

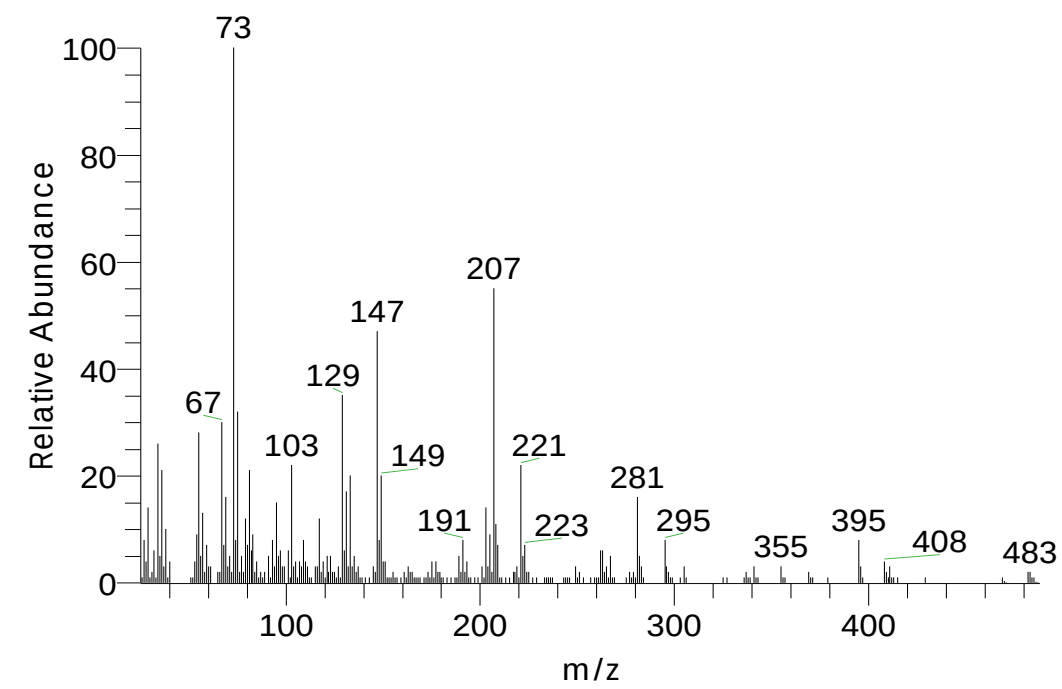

Oil_DrHafsa #11016 RT: 40.94 AV: 1 NL: 1.67E6
T: + c EI Full ms [50.000-750.000]

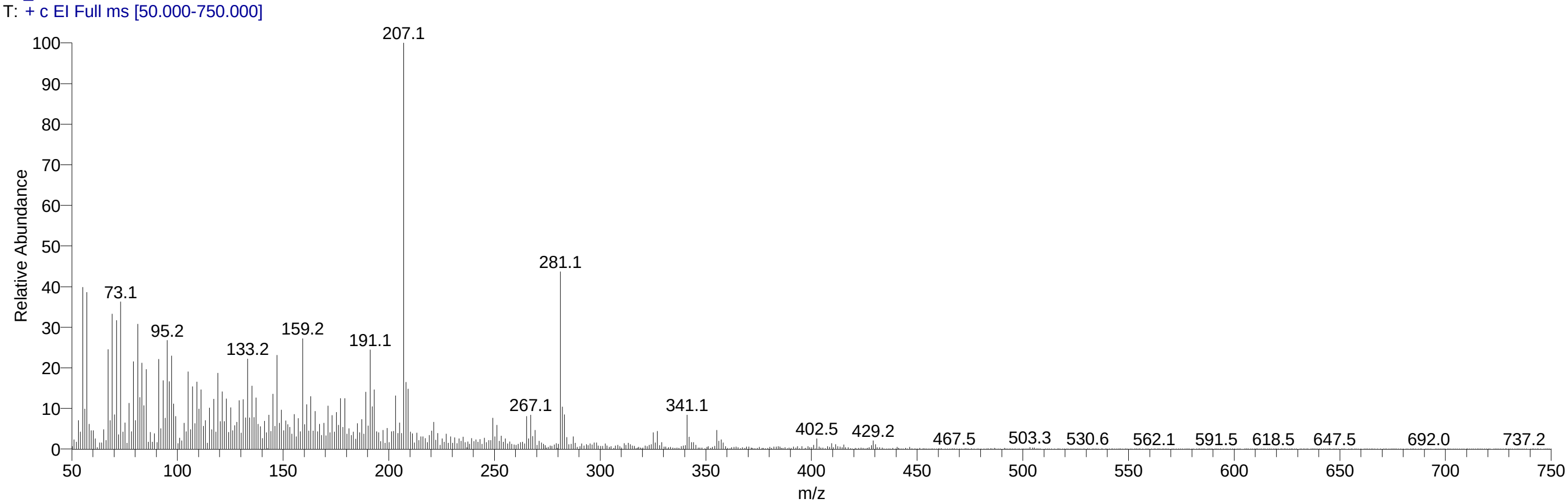

RT	Compound Name	Area %	MF	Molecular Formula	Molecular Weight	Cas #	Library
40.94	4H-1-BENZOPYRAN-4-ONE, 2-(3,4-DIMETHOXYPHENYL)-3,5-DIHYDROXY-7-METHOXY-	0.24	766	C18H16O7	344	6068-80-0	WileyRegistry8e
40.94	9,12,15-OCTADECATRIENOIC ACID, 2,3-BIS[(TRIMETHYLSILYL)OXY]PROPYL ESTER, (Z,Z,Z)-	0.24	788	C27H52O4Si2	496	55521-22-7	WileyRegistry8e
40.94	9,12-OCTADECADIENOIC ACID (Z,Z)-, 2,3-BIS[(TRIMETHYLSILYL)OXY]PROPYL ESTER	0.24	775	C27H54O4Si2	498	54284-45-6	WileyRegistry8e
40.94	3-(TETRADECANOYLOXY)-2-[(TRIMETHYLSILYL)OXY]PROPYL MYRISTATE	0.24	668	C34H68O5Si	584	NA	WileyRegistry8e
40.94	4H-1-BENZOPYRAN-4-ONE, 2-(3,4-DIMETHOXYPHENYL)-5,7-DIHYDROXY-	0.24	698	C17H14O6	314	4712-12-3	WileyRegistry8e

My GC-MS Report

Compound Structure	Hit Spectrum

4H-1-BENZOPYRAN-4-ONE, 2-(3,4-DIMETHOXYPHENYL)-3,5-DIHYDROXY-7-METHOXY-
Formula C18H16O7, MW 344, CAS# 6068-80-0, Entry# 224392
3',4',7-TRIMETHYLQUERCETIN

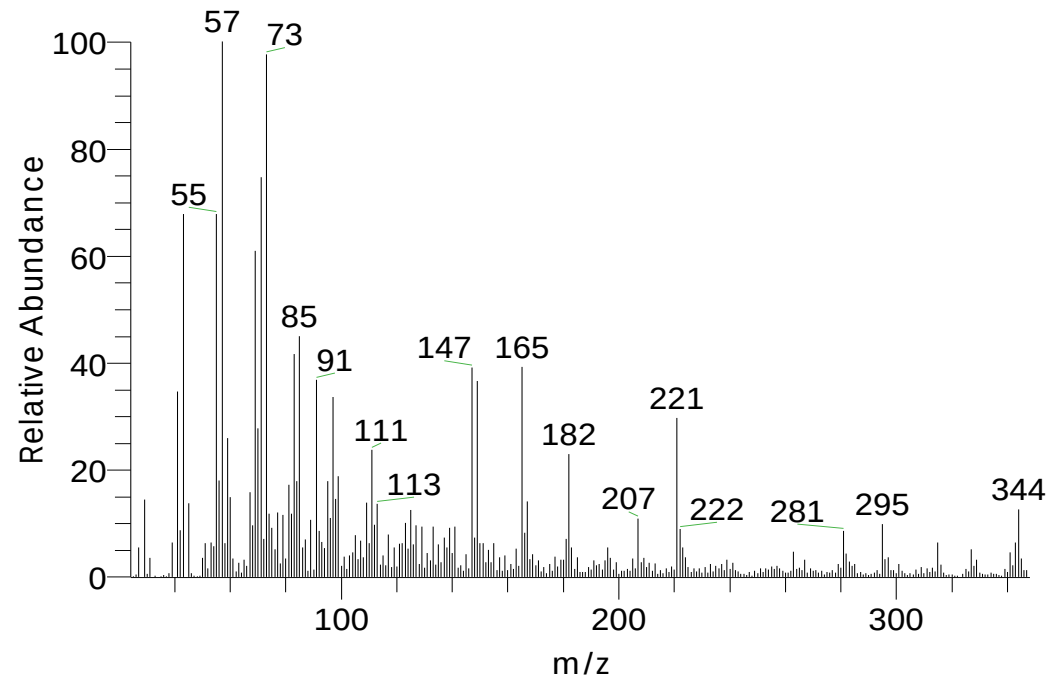

9,12,15-OCTADECATRIENOIC ACID, 2,3-BIS[(TRIMETHYLSILYL)OXY]PROPYL ESTER, (Z,Z,Z)-
Formula C27H52O4Si2, MW 496, CAS# 55521-22-7, Entry# 284834
2,3-BIS[(TRIMETHYLSILYL)OXY]PROPYL (9E,12E,15E)-9,12,15-OCTADECATRIENOATE #

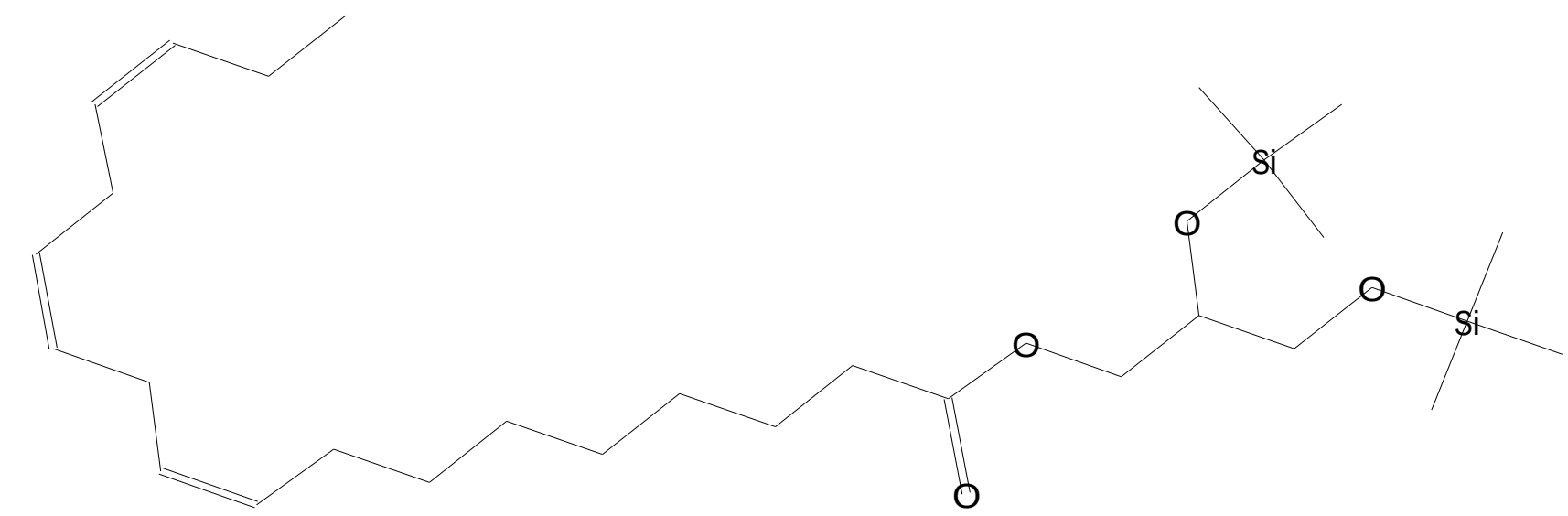

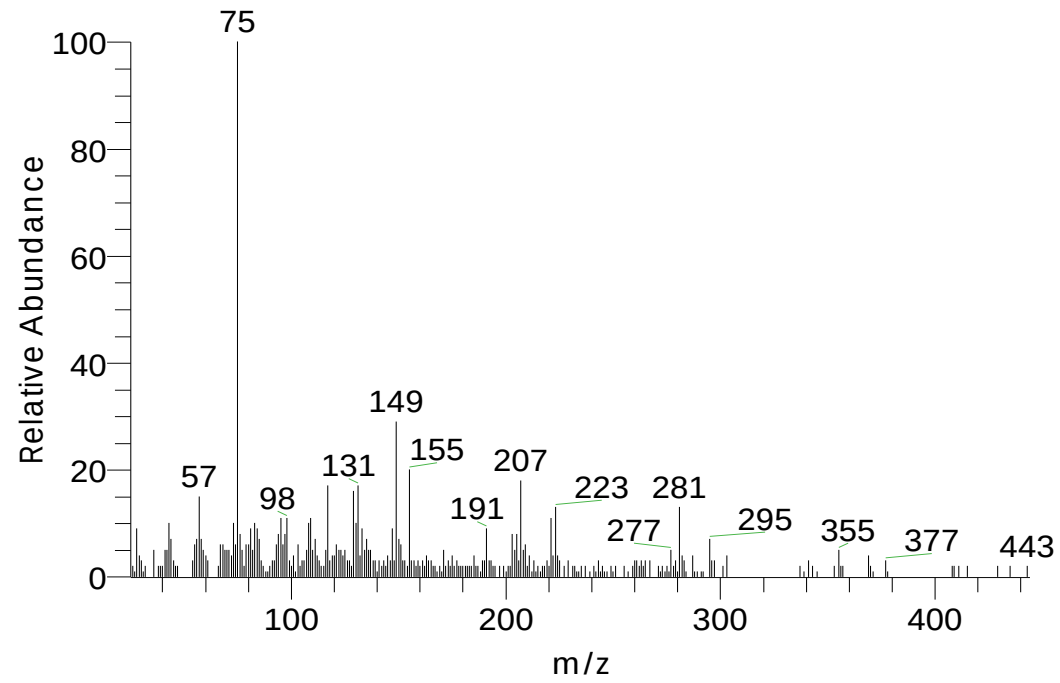

9,12-OCTADECADIENOIC ACID (Z,Z)-, 2,3-BIS[(TRIMETHYLSILYL)OXY]PROPYL ESTER
Formula C27H54O4Si2, MW 498, CAS# 54284-45-6, Entry# 285148
2,3-BIS[(TRIMETHYLSILYL)OXY]PROPYL (9Z,12Z)-9,12-OCTADECADIENOATE #

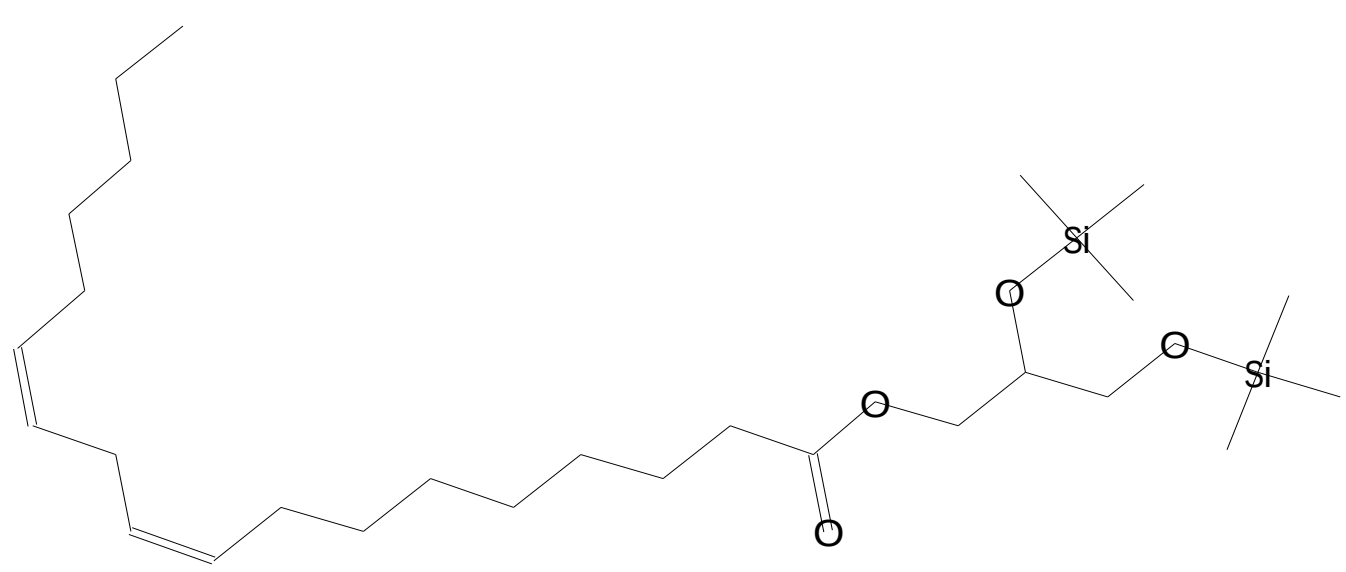

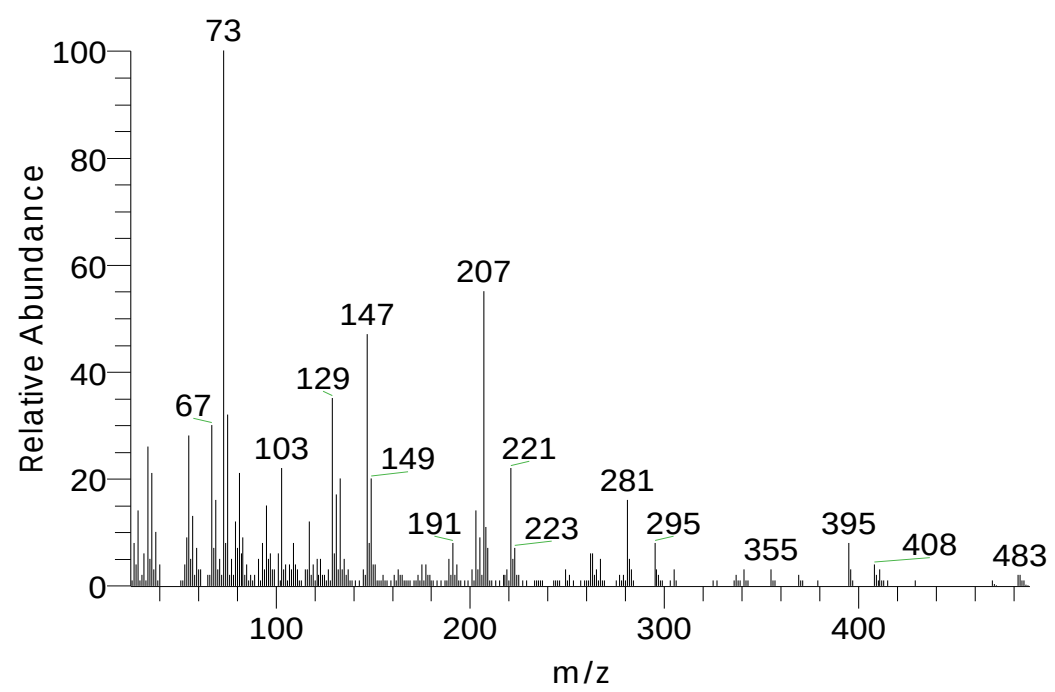

3-(TETRADECANOYLOXY)-2-[(TRIMETHYLSILYL)OXY]PROPYL MYRISTATE
Formula C34H68O5Si, MW 584, CAS# NA, Entry# 366625

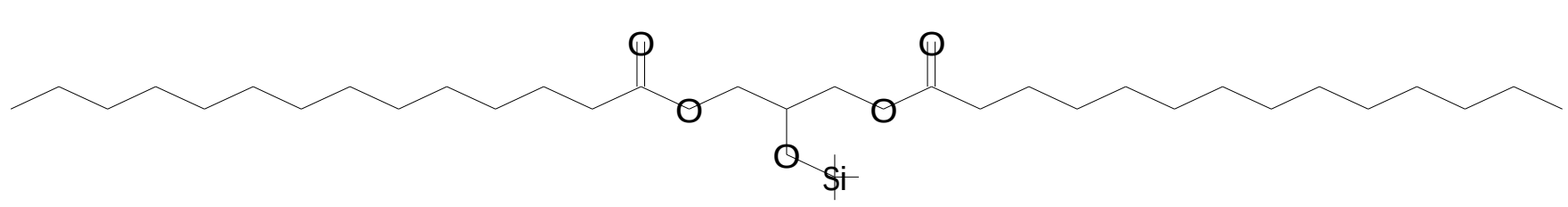

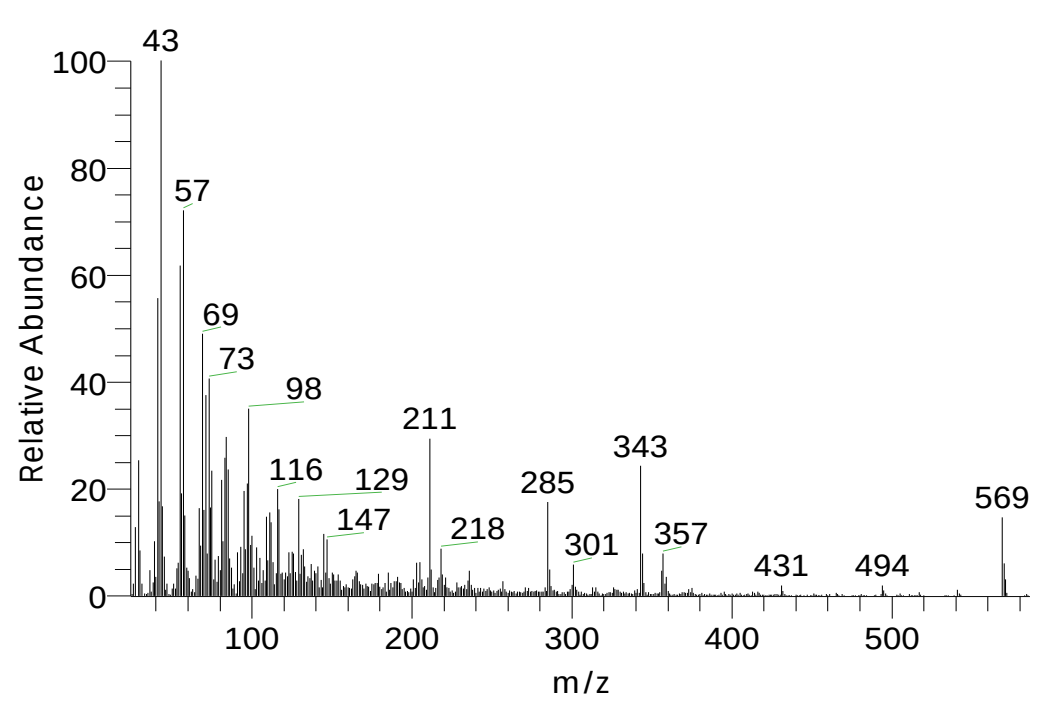

My GC-MS Report

Compound Structure | Hit Spectrum

4H-1-BENZOPYRAN-4-ONE, 2-(3,4-DIMETHOXYPHENYL)-5,7-DIHYDROXY-
Formula C17H14O6, MW 314, CAS# 4712-12-3, Entry# 201548
08027209001 FLAVONE 5,7-OH,3',4'-OME

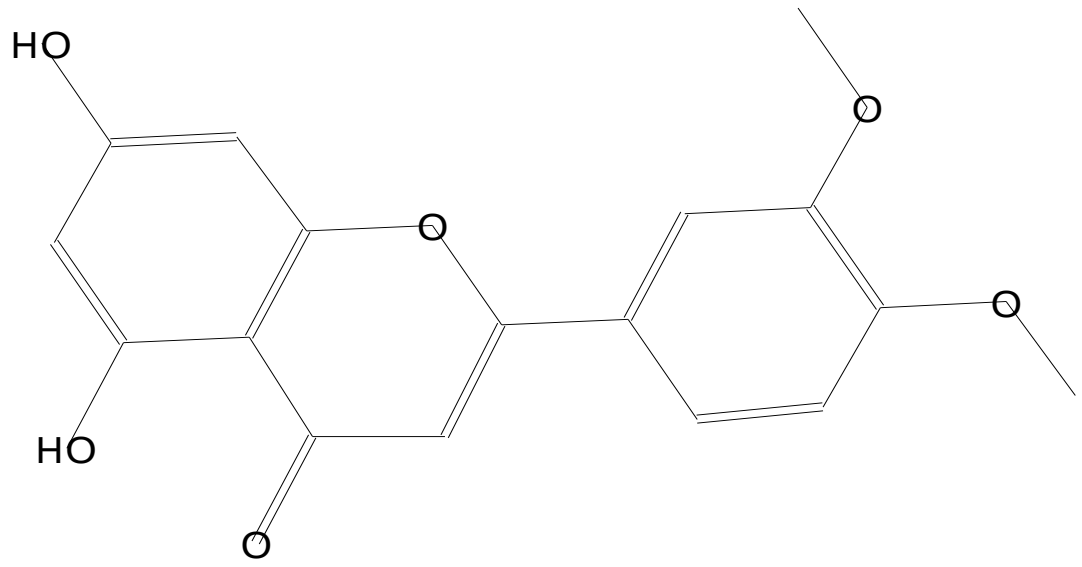

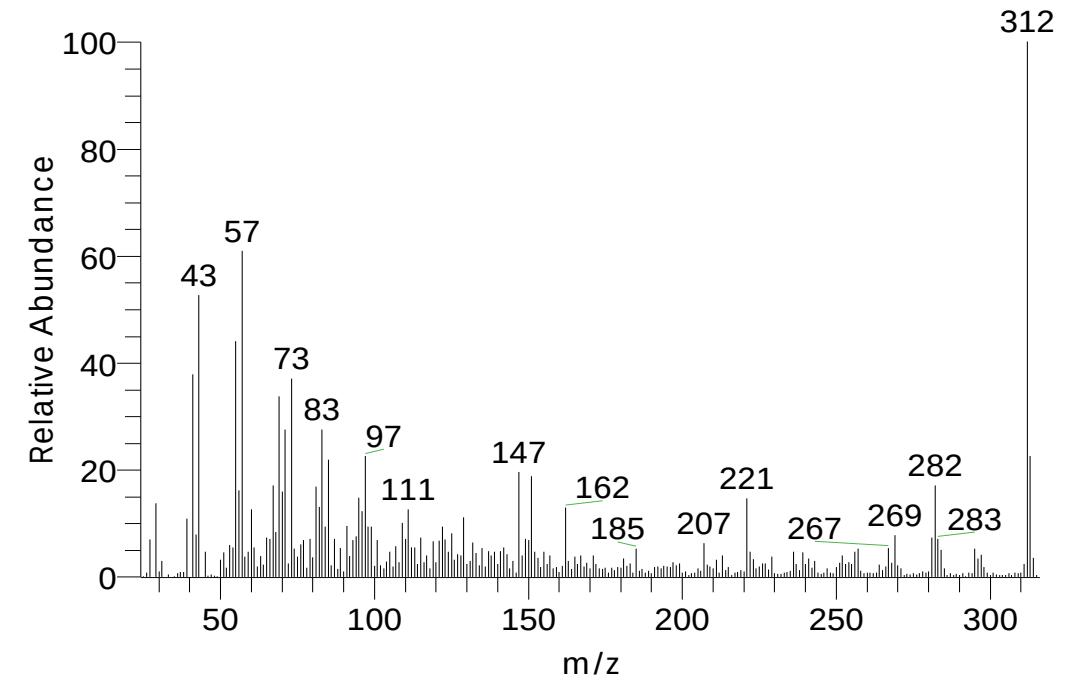

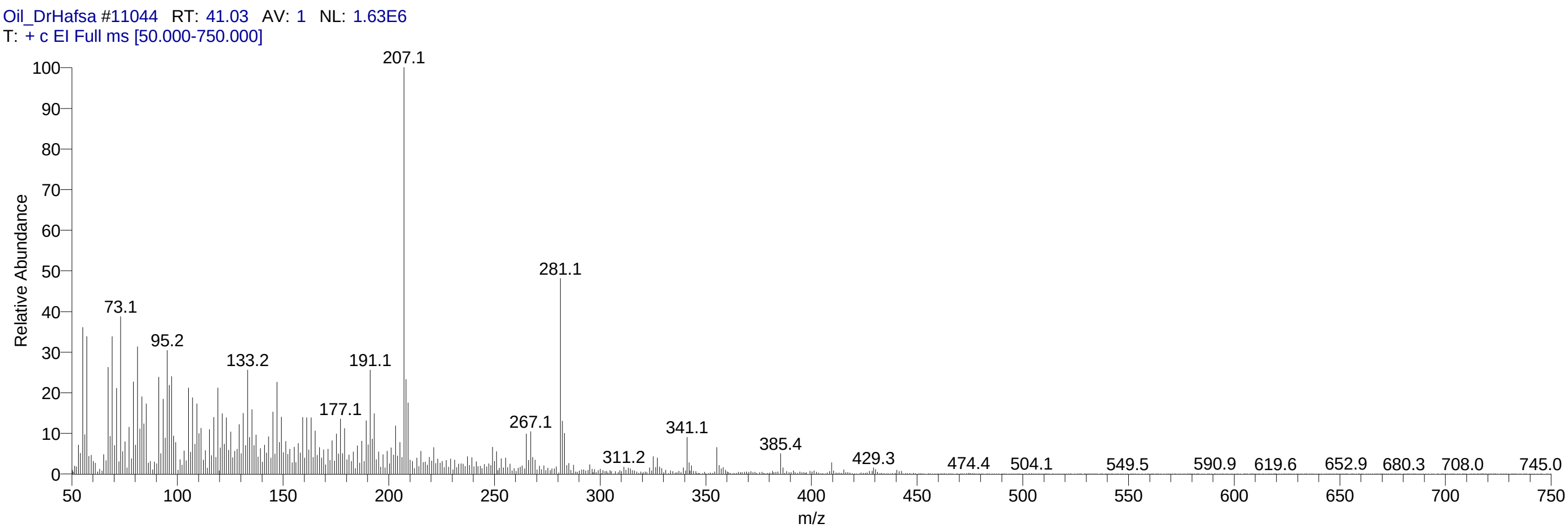

RT	Compound Name	Area %	MF	Molecular Formula	Molecular Weight	Cas #	Library
41.03	4H-1-BENZOPYRAN-4-ONE, 2-(3,4-DIMETHOXYPHENYL)-3,5-DIHYDROXY-7-METHOXY-	0.27	764	C18H16O7	344	6068-80-0	WileyRegistry8e
41.03	3-(TETRADECANOYLOXY)-2-[(TRIMETHYLSILYL)OXY]PROPYL MYRISTATE	0.27	668	C34H68O5Si	584	NA	WileyRegistry8e
41.03	Acetic acid, 17-(4-hydroxy-5-methoxy-1,5-dimethylhexyl)-4,4,10,13,14-pentamethyl-2,3,4,5,6,7,10,11,12,13,14,15,16,17-tetradecahydrocyclopenta[a]phenanthryl ester	0.27	666	C33H56O4	516	NA	mainlib
41.03	24-HYDROXY-25-METHOXYLANOST-8-EN-3-YL ACETATE	0.27	666	C33H56O4	516	NA	WileyRegistry8e
41.03	4H-1-BENZOPYRAN-4-ONE, 2-(3,4-DIMETHOXYPHENYL)-5,7-DIHYDROXY-	0.27	697	C17H14O6	314	4712-12-3	WileyRegistry8e

Compound Structure | Hit Spectrum

4H-1-BENZOPYRAN-4-ONE, 2-(3,4-DIMETHOXYPHENYL)-3,5-DIHYDROXY-7-METHOXY-
Formula C18H16O7, MW 344, CAS# 6068-80-0, Entry# 224392
3',4',7-TRIMETHYLQUERCETIN

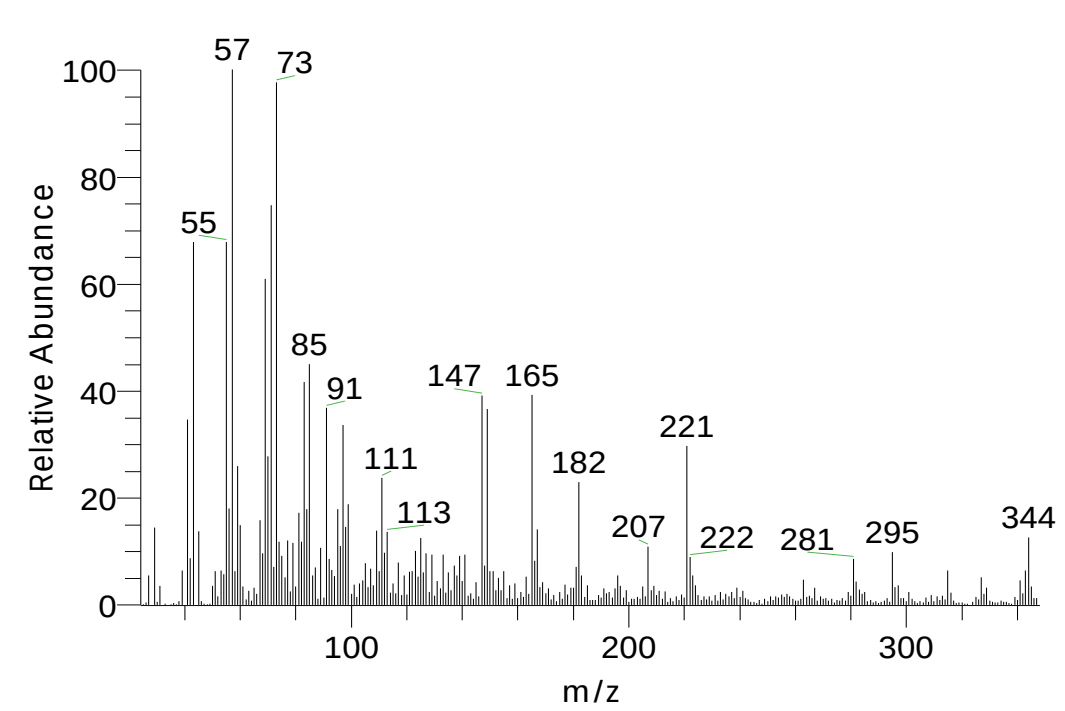

My GC-MS Report

Compound Structure | Hit Spectrum

3-(TETRADECANOYLOXY)-2-[(TRIMETHYLSILYL)OXY]PROPYL MYRISTATE
Formula C34H68O5Si, MW 584, CAS# NA, Entry# 366625

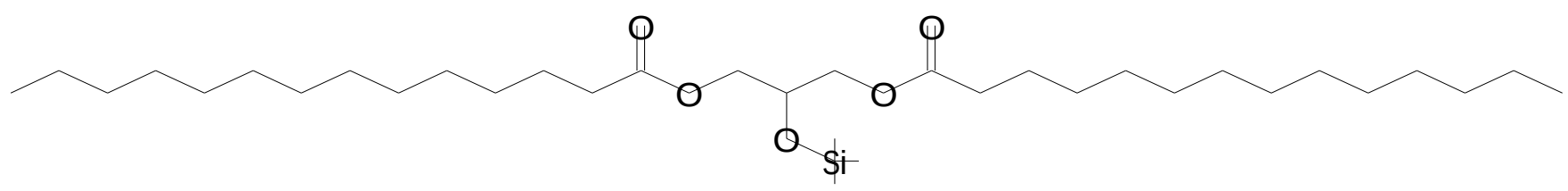

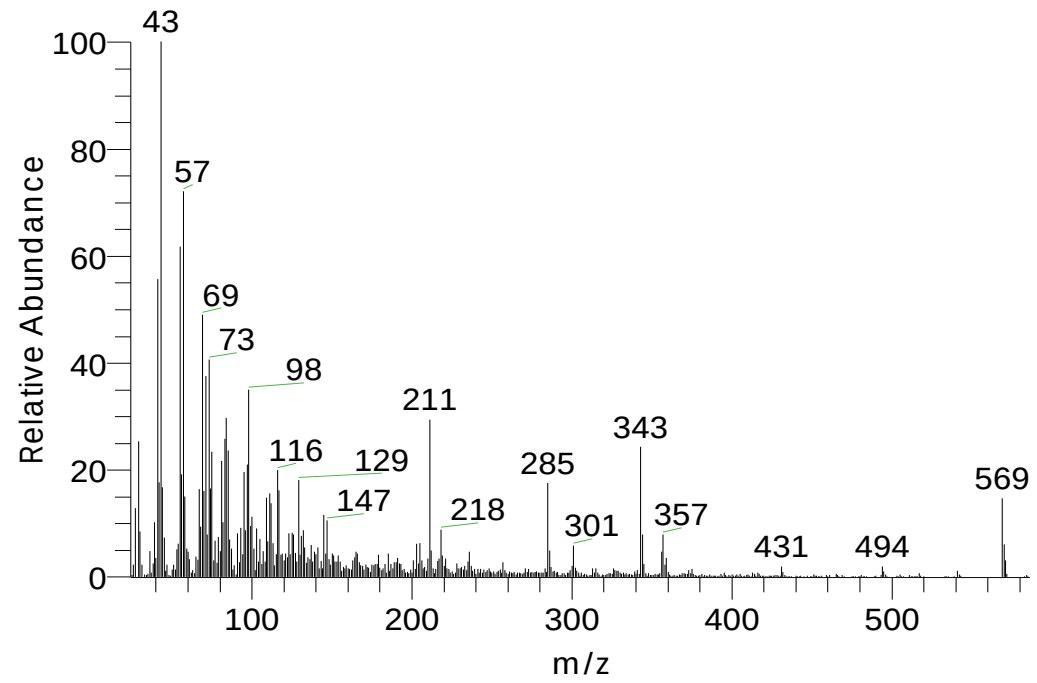

Formula C33H56O4, MW 516, CAS# NA, Entry# 40616
24-Hydroxy-25-methoxylanost-8-en-3-yl acetate #

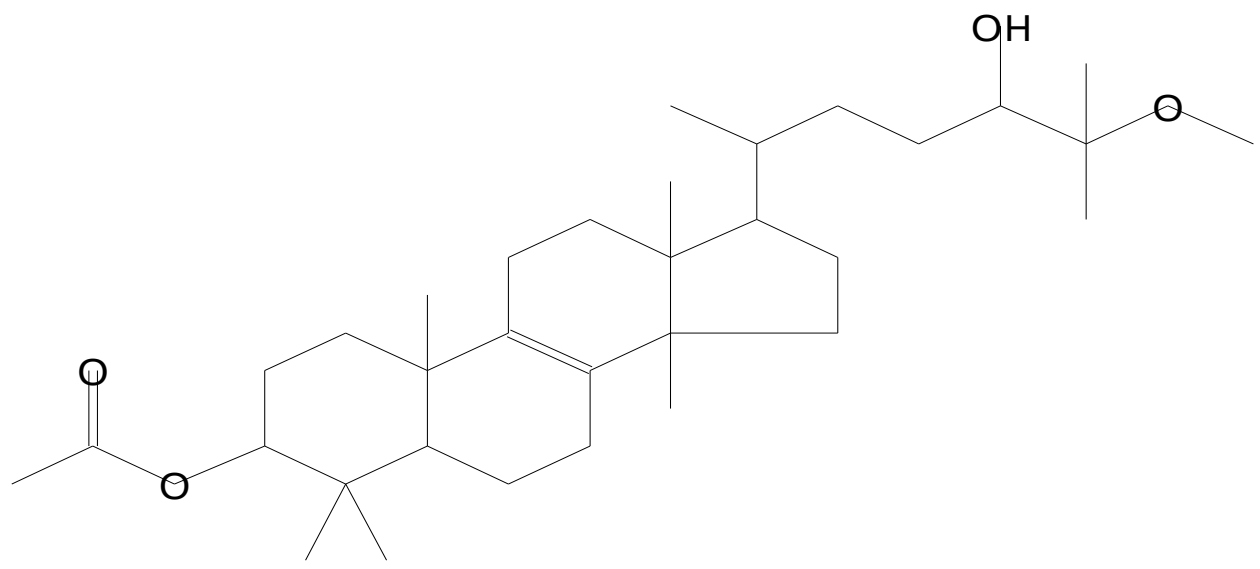

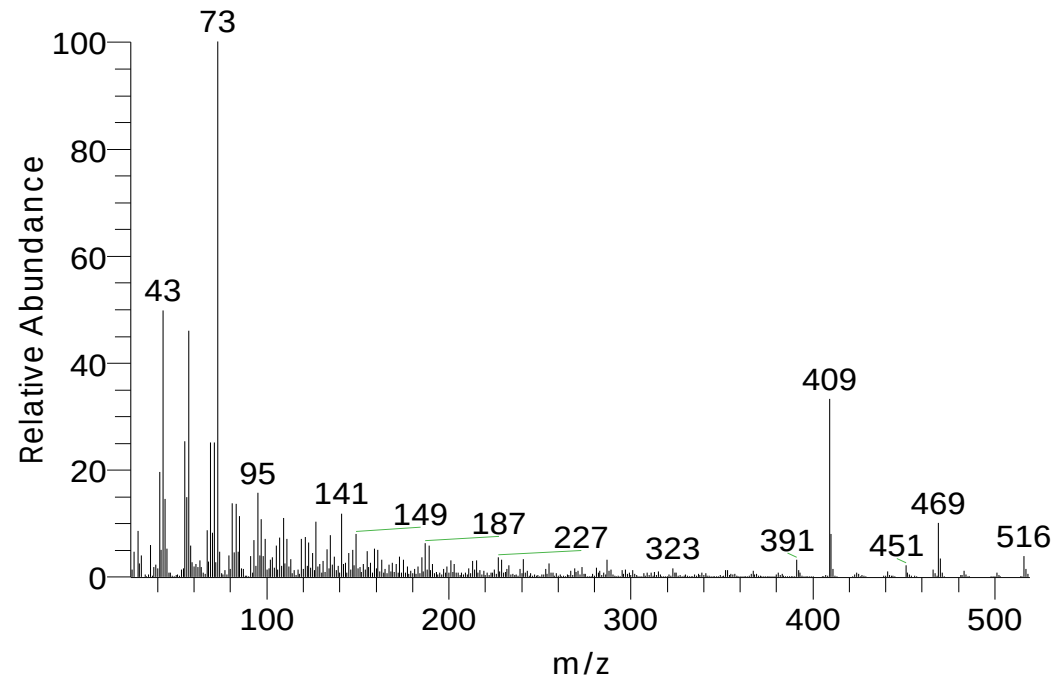

24-HYDROXY-25-METHOXYLANOST-8-EN-3-YL ACETATE
Formula C33H56O4, MW 516, CAS# NA, Entry# 366616

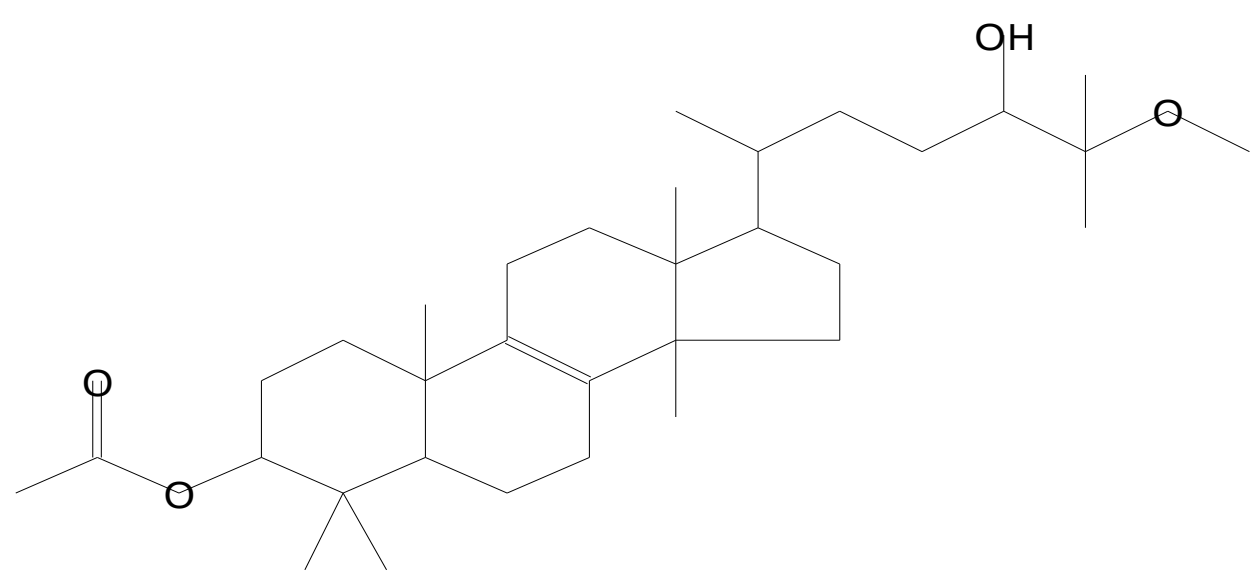

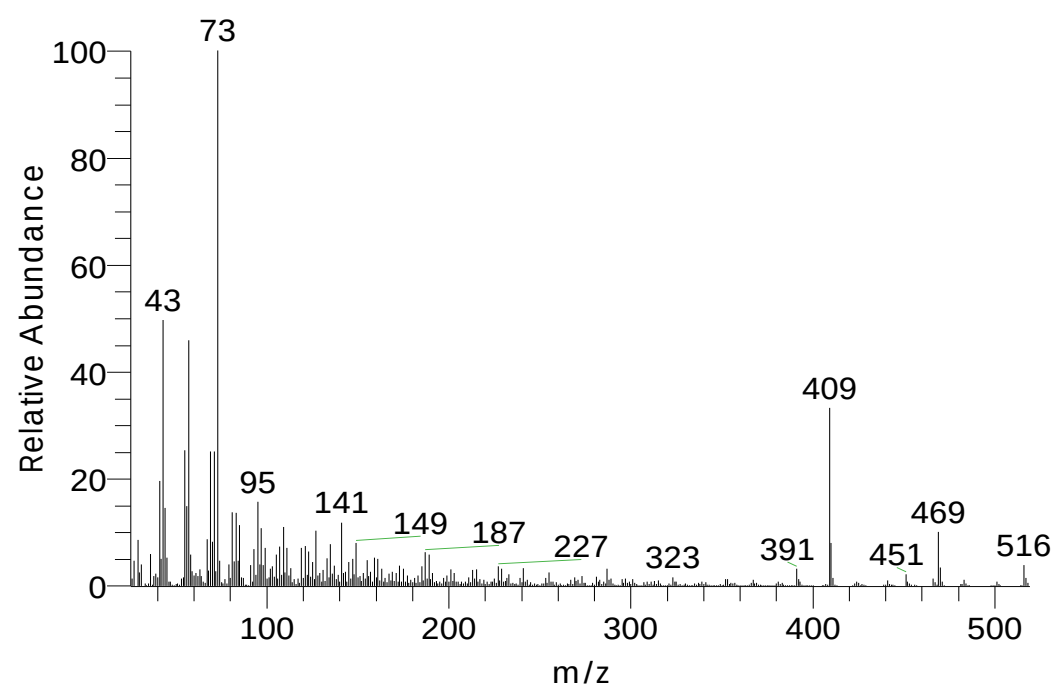

4H-1-BENZOPYRAN-4-ONE, 2-(3,4-DIMETHOXYPHENYL)-5,7-DIHYDROXY-
Formula C17H14O6, MW 314, CAS# 4712-12-3, Entry# 201548
08027209001 FLAVONE 5,7-OH,3',4'-OME

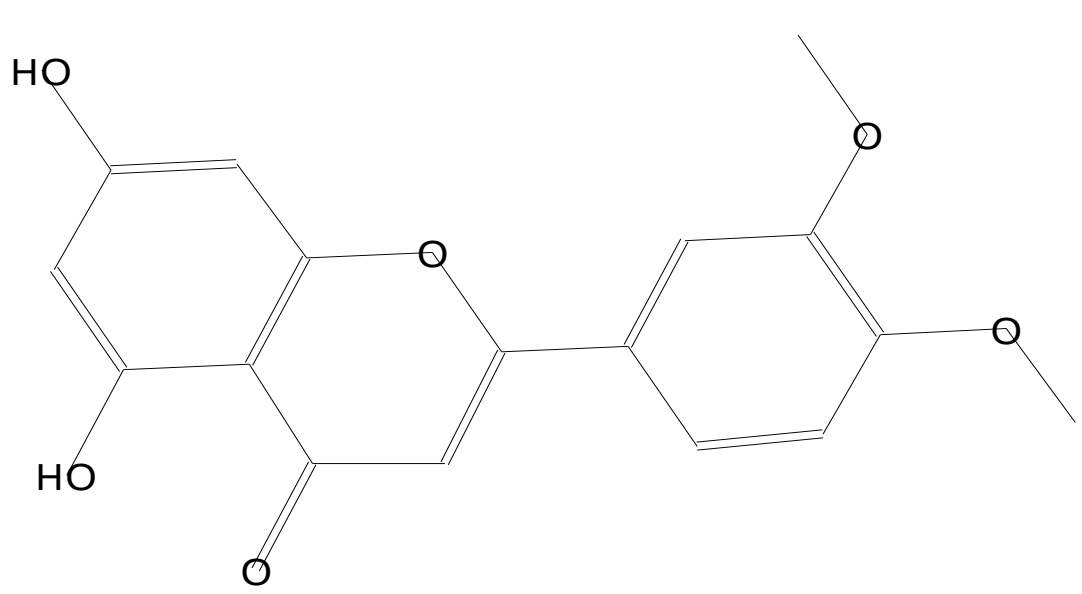

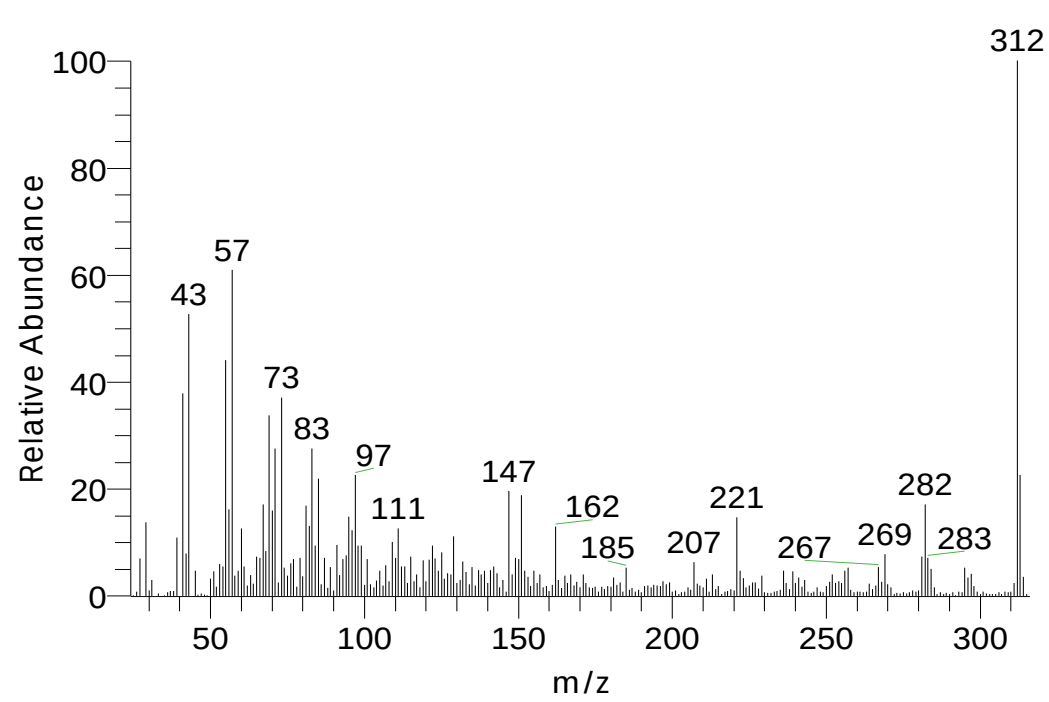

My GC-MS Report

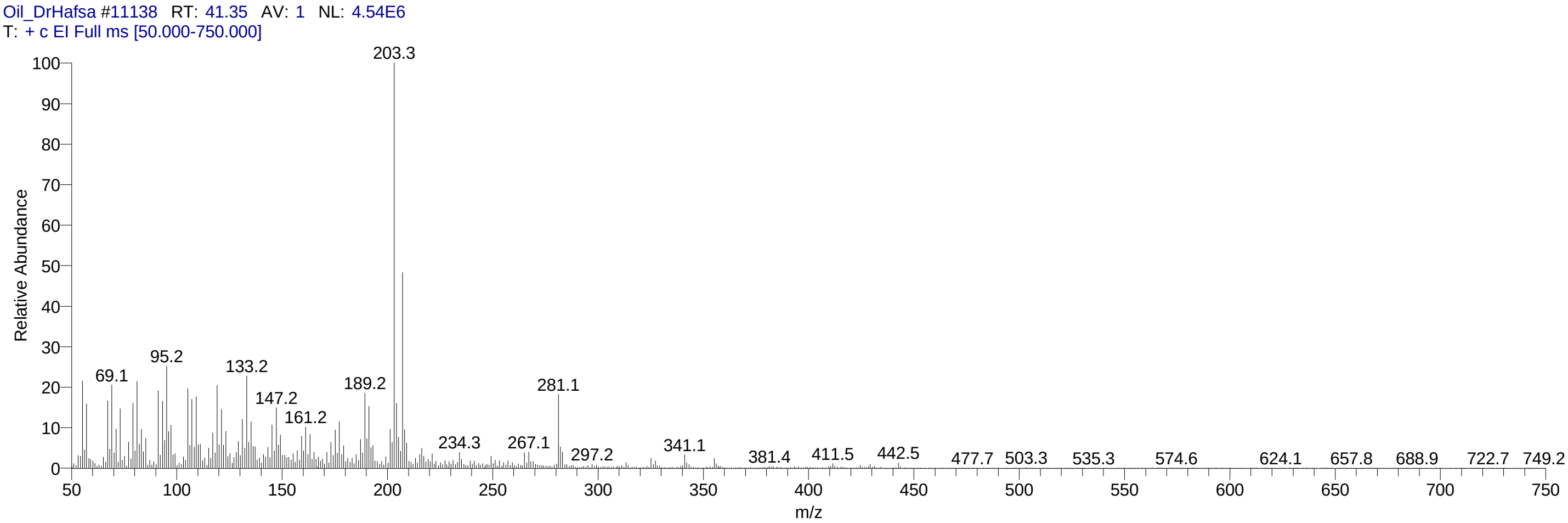

RT	Compound Name	Area %	MF	Molecular Formula	Molecular Weight	Cas #	Library
41.35	Betulin	5.15	788	C30H50O2	442	473-98-3	replib
41.35	LUP-20(29)-ENE-3,28-DIOL, (3á)-	5.15	822	C30H50O2	442	473-98-3	WileyRegistry8e
41.35	Betulin	5.15	793	C30H50O2	442	473-98-3	replib
41.35	Betulinaldehyde	5.15	775	C30H48O2	440	13159-28-9	mainlib
41.35	Uvaol	5.15	819	C30H50O2	442	545-46-0	mainlib

Compound Structure	Hit Spectrum

Betulin
Formula C30H50O2, MW 442, CAS# 473-98-3, Entry# 27738
Lup-20(29)-ene-3,28-diol, (3á)-

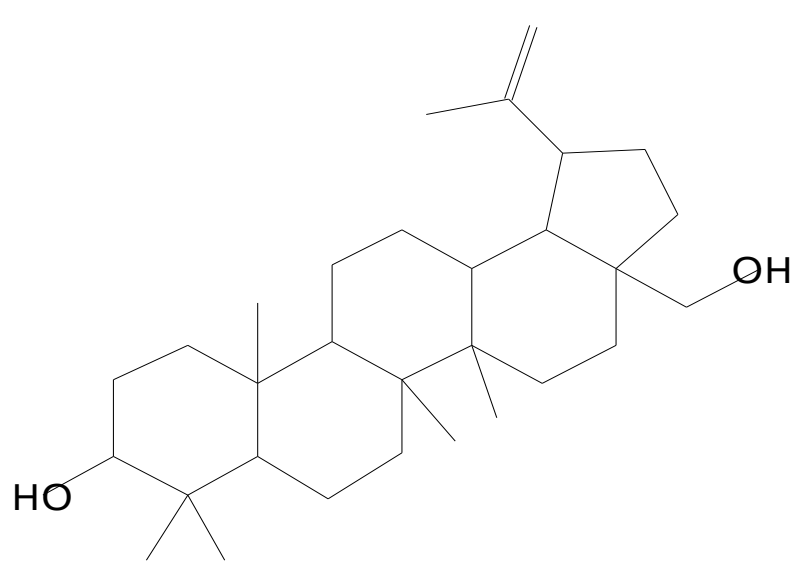

SI 785, RSI 788, replib, Entry# 27738, CAS# 473-98-3, Betulin

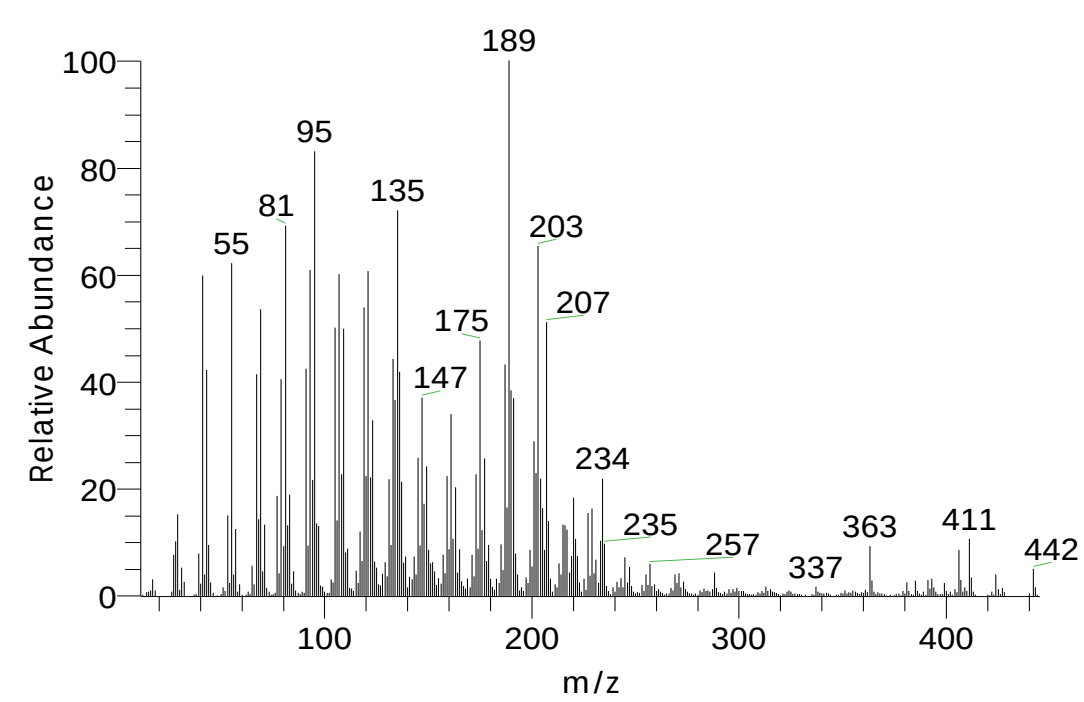

LUP-20(29)-ENE-3,28-DIOL, (3á)-
Formula C30H50O2, MW 442, CAS# 473-98-3, Entry# 349524
LUP-20(29)-ENE-3,28-DIOL #

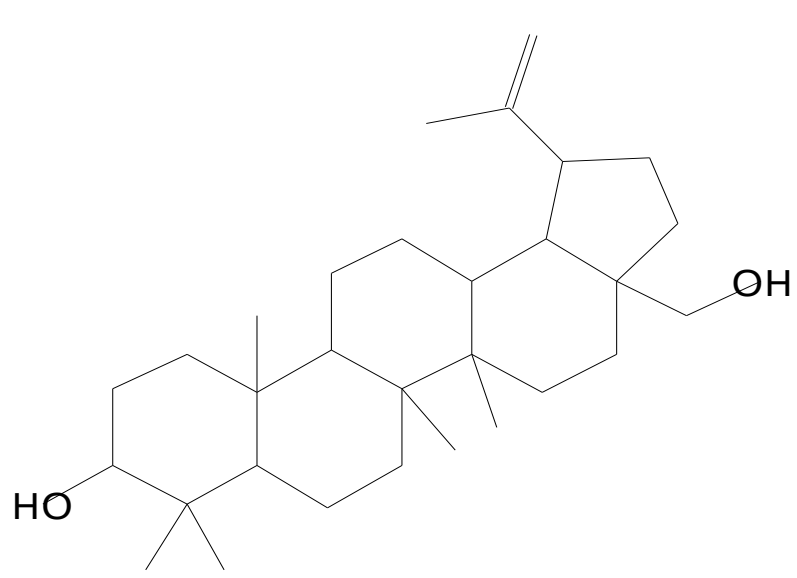

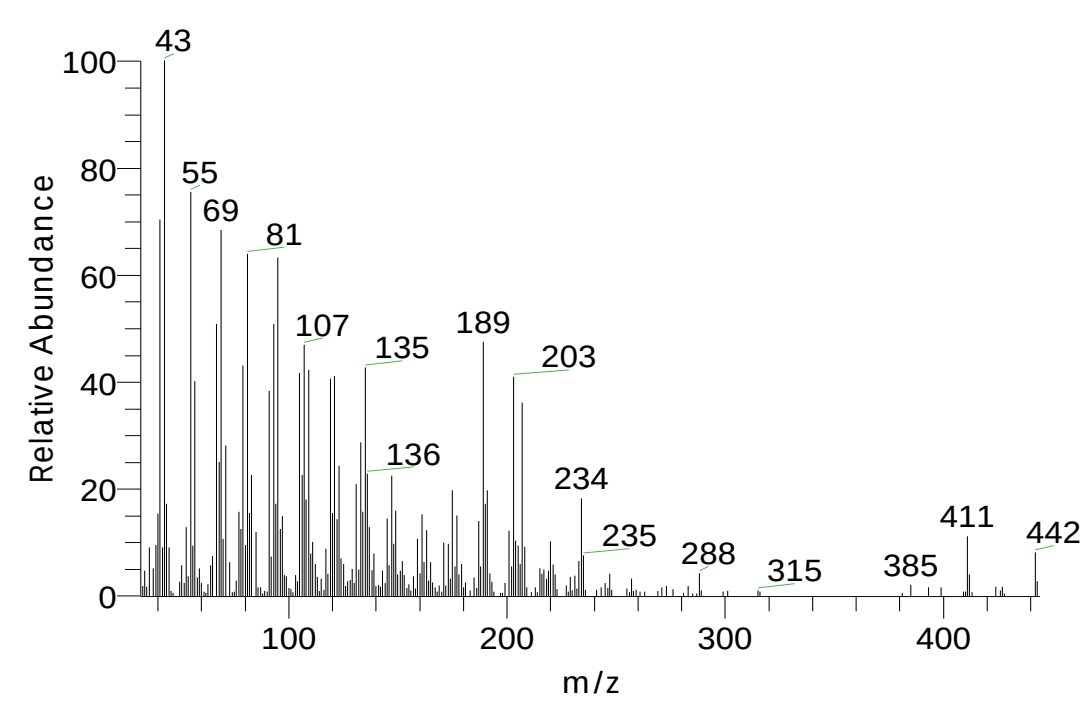

My GC-MS Report

Compound Structure | Hit Spectrum

Betulin
Formula C30H50O2, MW 442, CAS# 473-98-3, Entry# 27797
Lup-20(29)-ene-3,28-diol, (3á)-

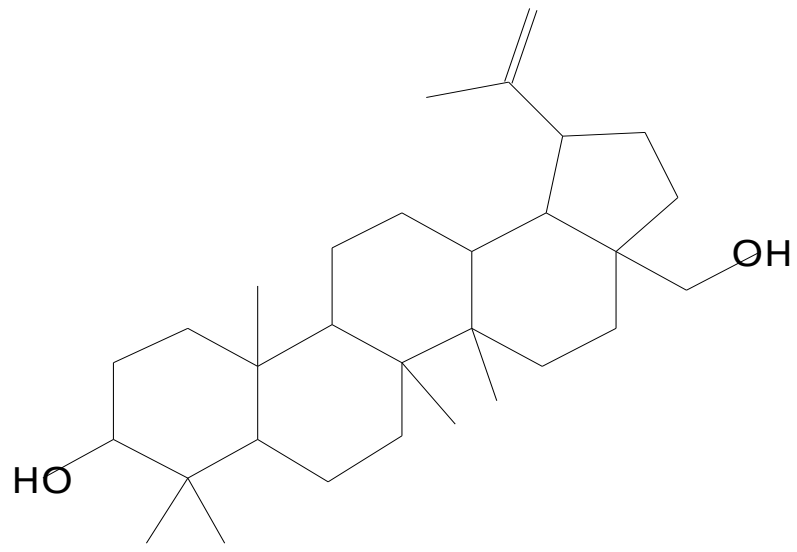

SI 754, RSI 793, replib, Entry# 27797, CAS# 473-98-3, Betulin

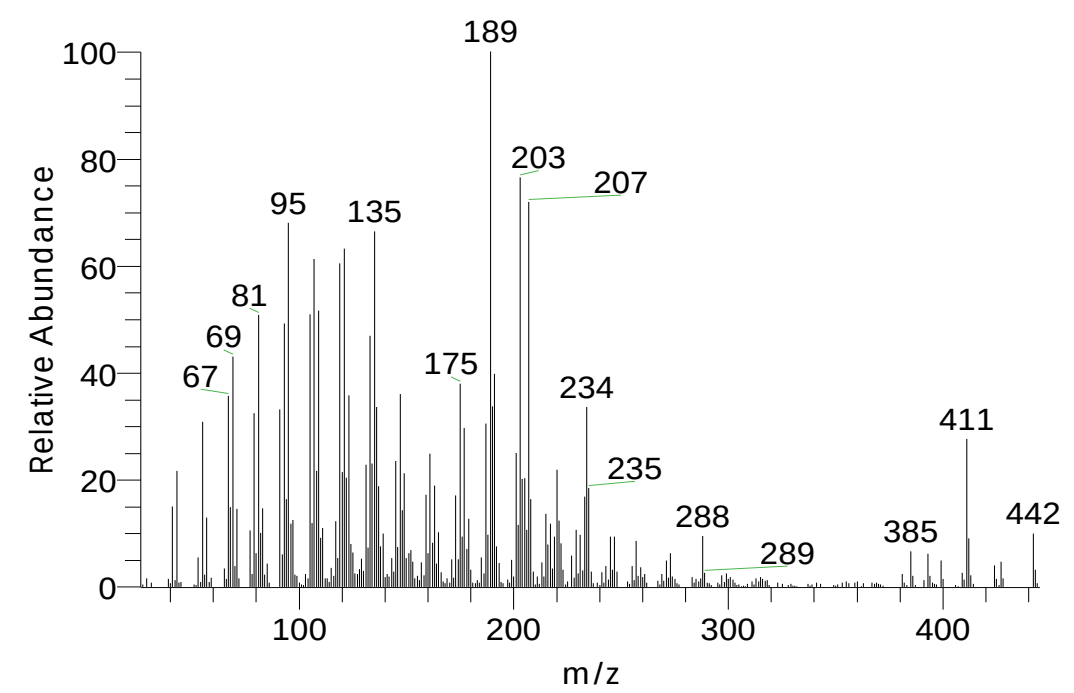

Betulinaldehyde
Formula C30H48O2, MW 440, CAS# 13159-28-9, Entry# 175929
$:28FELCJAPFJOPHSD-UHFFFAOYSA-N

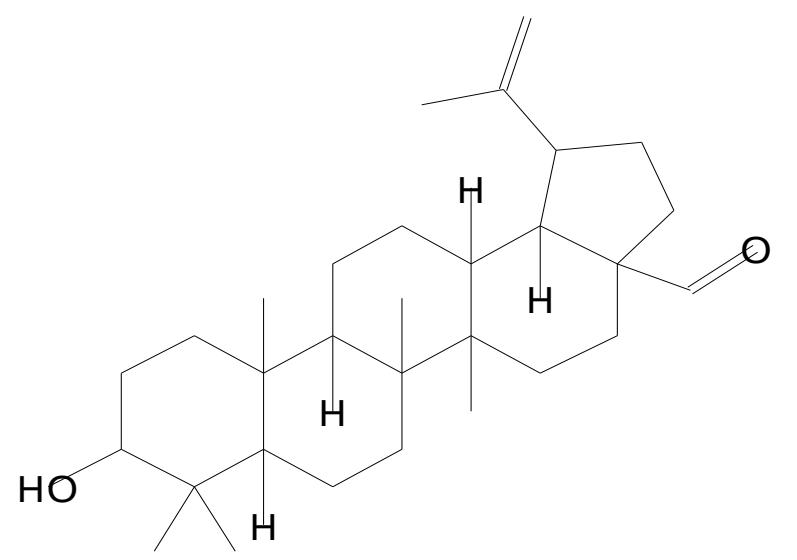

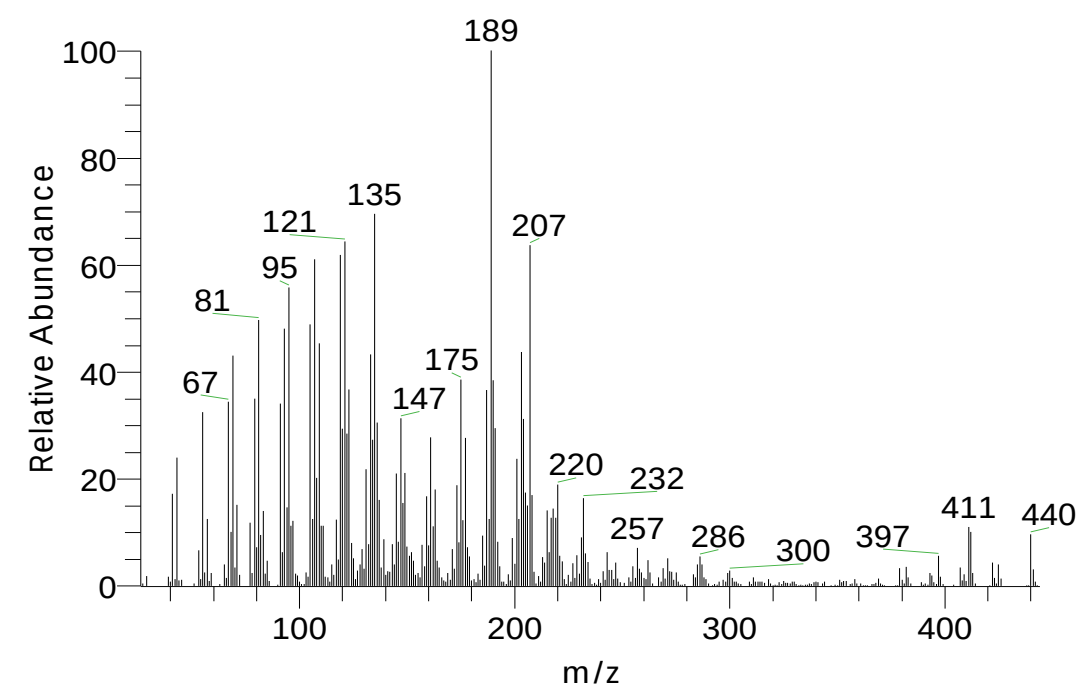

Uvaol
Formula C30H50O2, MW 442, CAS# 545-46-0, Entry# 186336
Urs-12-ene-3,28-diol, (3á)-

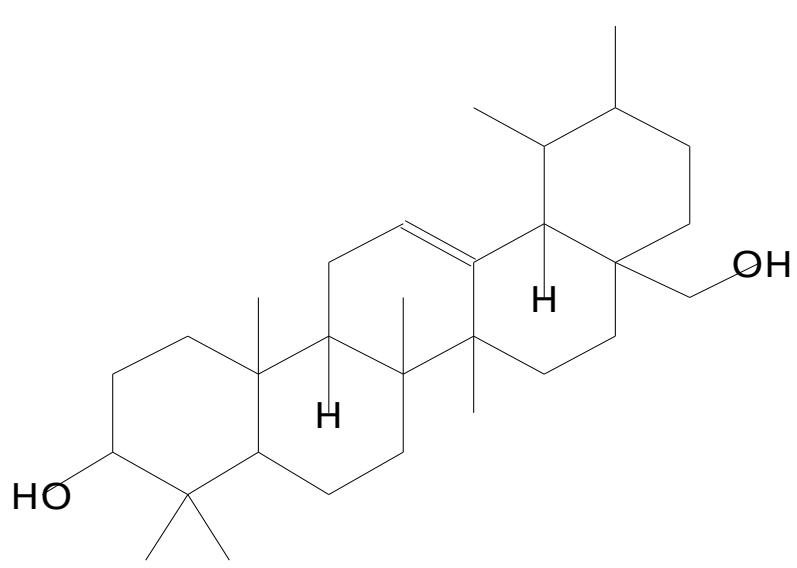

SI 737, RSI 819, mainlib, Entry# 186336, CAS# 545-46-0, Uvaol

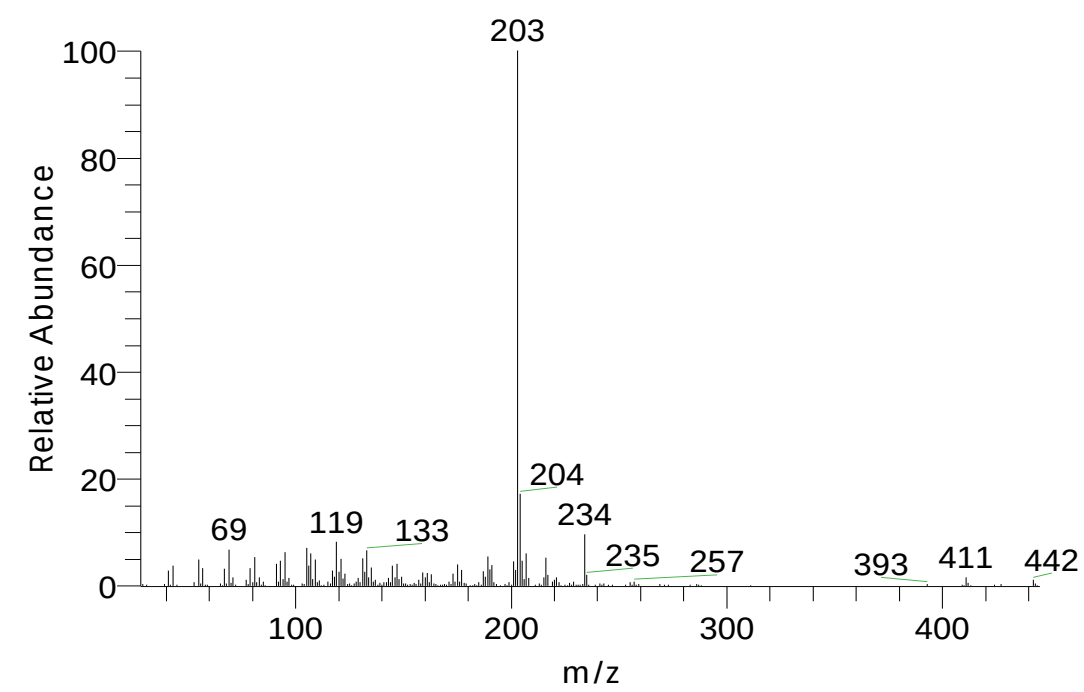

Oil_DrHafsa #11260 RT: 41.76 AV: 1 NL: 1.72E6
T: + c EI Full ms [50.000-750.000]

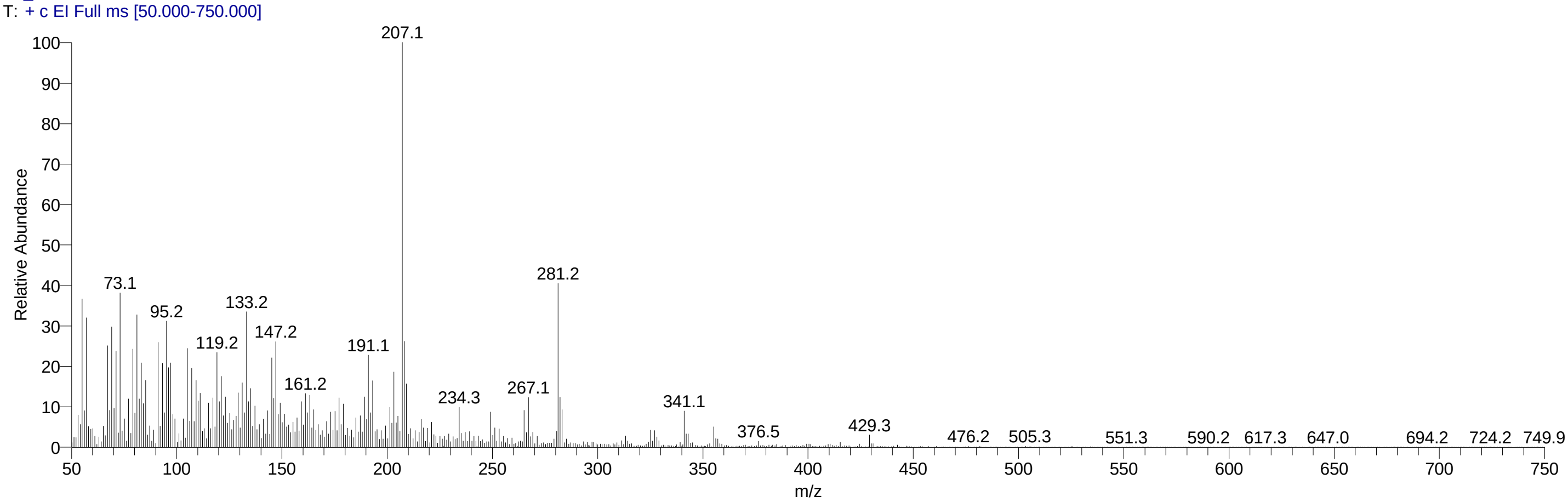

RT	Compound Name	Area %	MF	Molecular Formula	Molecular Weight	Cas #	Library
41.76	9,12,15-OCTADECATRIENOIC ACID, 2,3-BIS[(TRIMETHYLSILYL)OXY]PROPYL ESTER, (Z,Z,Z)-	0.94	775	C27H52O4Si2	496	55521-22-7	WileyRegistry8e

My GC-MS Report

RT	Compound Name	Area %	MF	Molecular Formula	Molecular Weight	Cas #	Library
41.76	9,12-OCTADECADIENOIC ACID (Z,Z)-, 2,3-BIS[(TRIMETHYLSILYL)OXY]PROPYL ESTER	0.94	775	C27H54O4Si2	498	54284-45-6	WileyRegistry8e
41.76	.psi.,.psi.-Carotene, 1,1',2,2'-tetrahydro-1,1'-dimethoxy-	0.94	715	C42H64O2	600	13833-01-7	mainlib
41.76	.PSI.,.PSI.-CAROTENE, 1,1',2,2'-TETRAHYDRO-1,1'-DIMETHOXY-	0.94	714	C42H64O2	600	13833-01-7	WileyRegistry8e
41.76	9-OCTADECENOIC ACID (Z)-, 2-[(TRIMETHYLSILYL)OXY]-1-[[(TRIMETHYLSILYL)OXY]METHYL]ETHYL ESTER	0.94	735	C27H56O4Si2	500	54284-48-9	WileyRegistry8e

Compound Structure | Hit Spectrum

9,12,15-OCTADECATRIENOIC ACID, 2,3-BIS[(TRIMETHYLSILYL)OXY]PROPYL ESTER, (Z,Z,Z)-
Formula C27H52O4Si2, MW 496, CAS# 55521-22-7, Entry# 284834
2,3-BIS[(TRIMETHYLSILYL)OXY]PROPYL (9E,12E,15E)-9,12,15-OCTADECATRIENOATE #

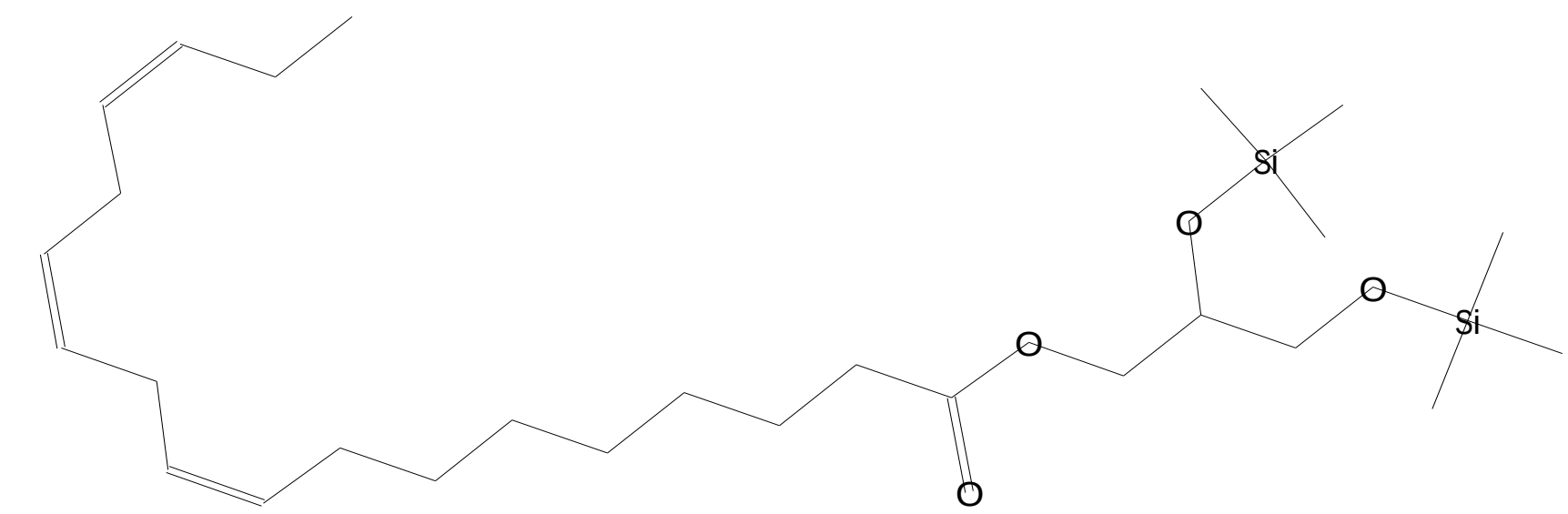

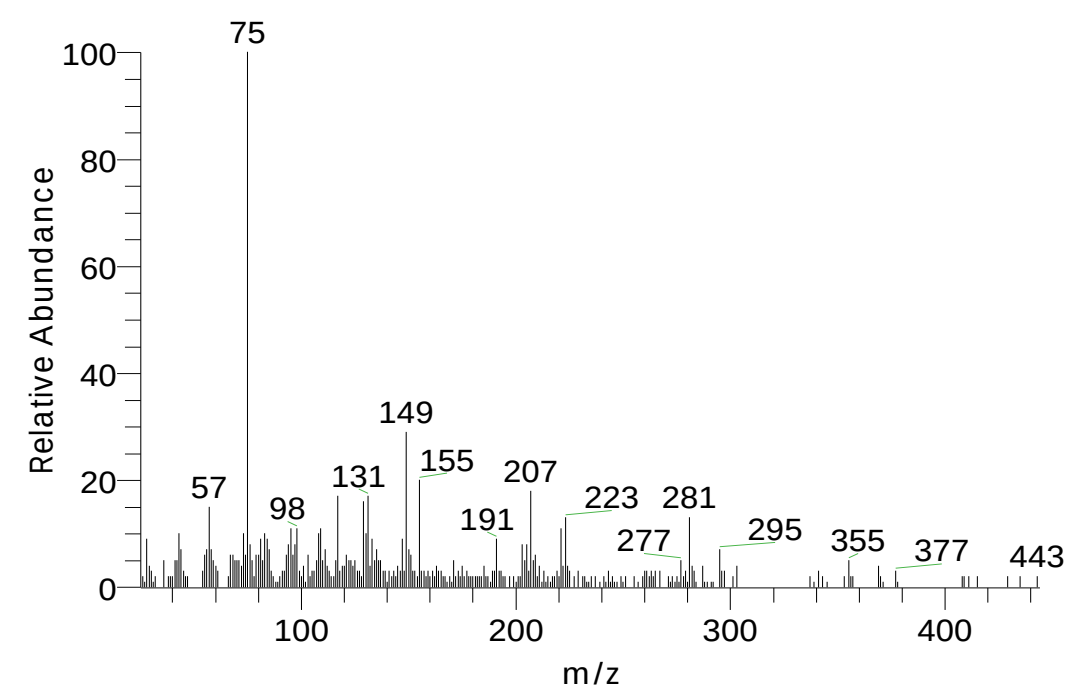

9,12-OCTADECADIENOIC ACID (Z,Z)-, 2,3-BIS[(TRIMETHYLSILYL)OXY]PROPYL ESTER
Formula C27H54O4Si2, MW 498, CAS# 54284-45-6, Entry# 285148
2,3-BIS[(TRIMETHYLSILYL)OXY]PROPYL (9Z,12Z)-9,12-OCTADECADIENOATE #

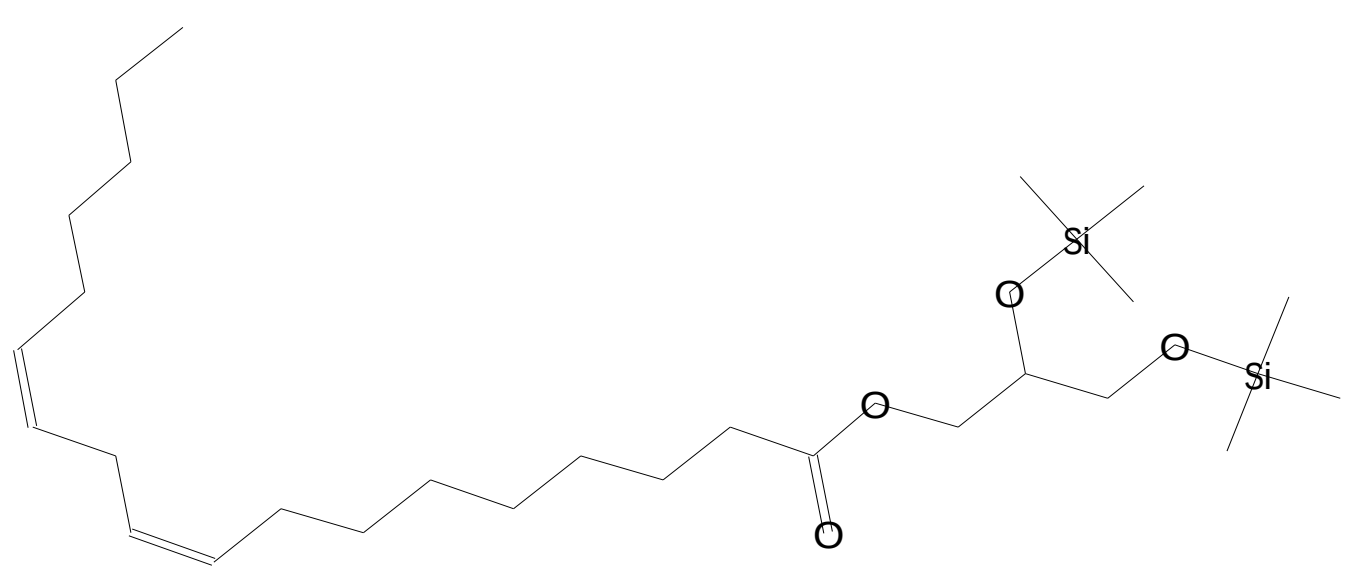

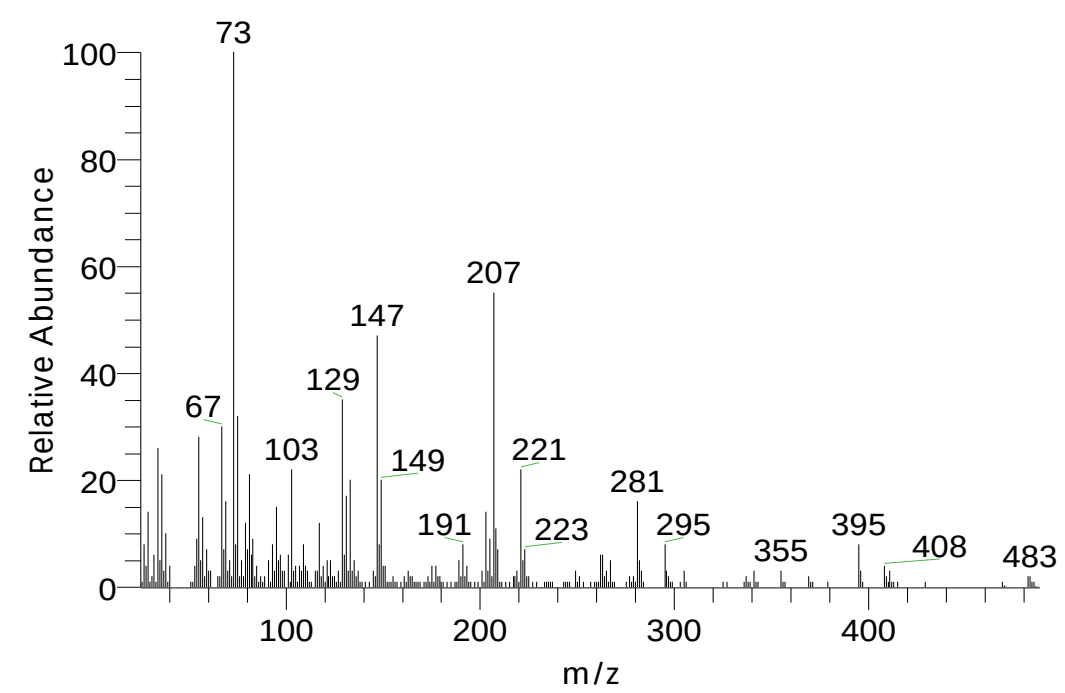

.psi.,.psi.-Carotene, 1,1',2,2'-tetrahydro-1,1'-dimethoxy-
Formula C42H64O2, MW 600, CAS# 13833-01-7, Entry# 41205
Lycopene, 1,1',2,2'-tetrahydro-1,1'-dimethoxy-, all-trans-

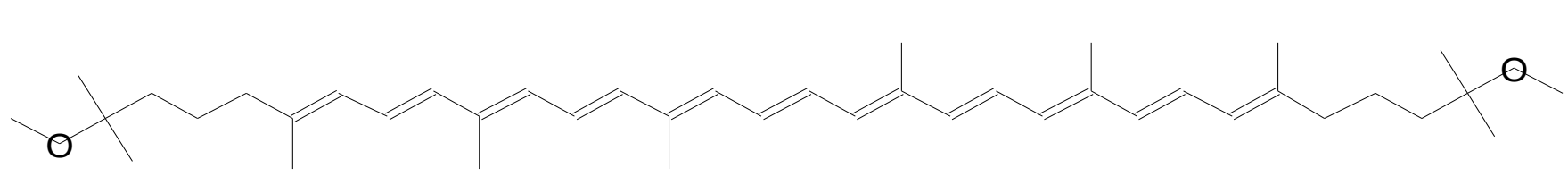

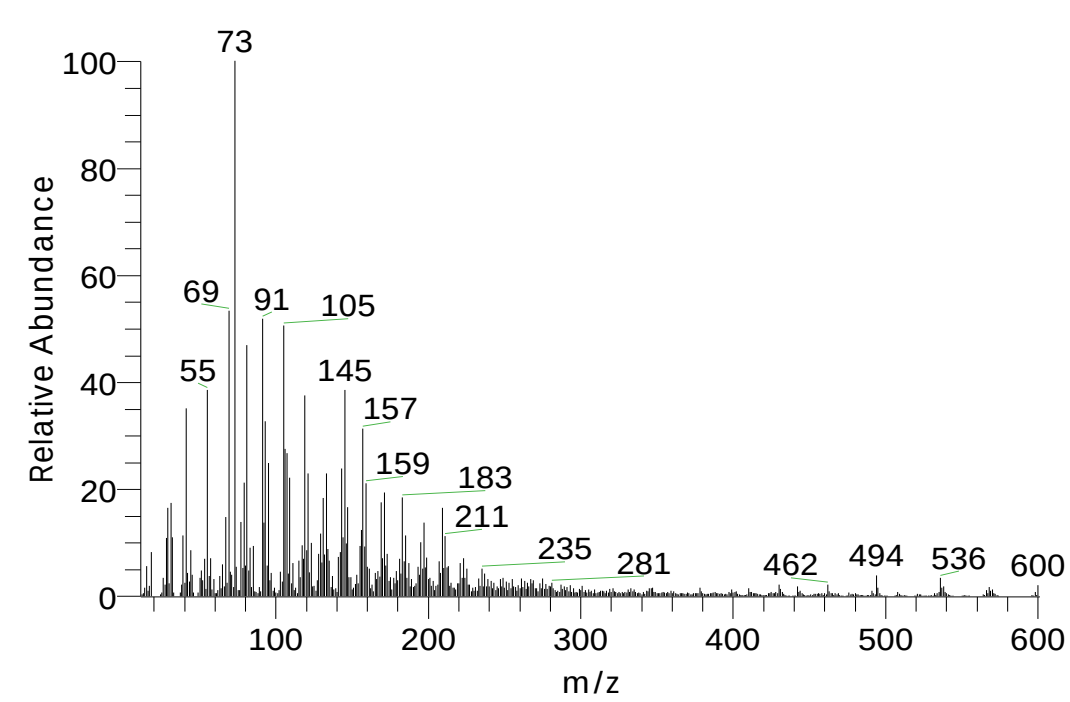

My GC-MS Report

Compound Structure | Hit Spectrum

.PSI.,.PSI.-CAROTENE, 1,1',2,2'-TETRAHYDRO-1,1'-DIMETHOXY-
Formula C42H64O2, MW 600, CAS# 13833-01-7, Entry# 296796
1,1',2,2'-TETRAHYDRO-PSI,PSI-CAROTENE #

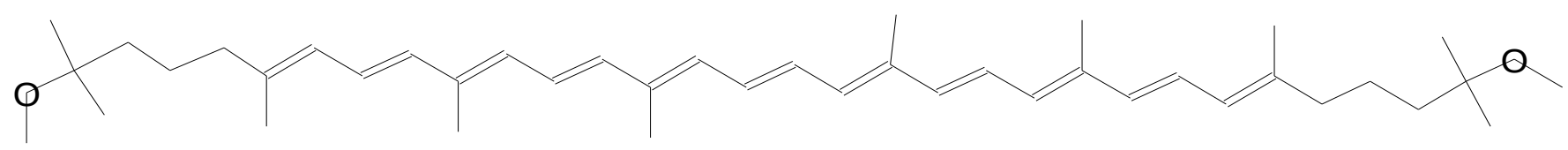

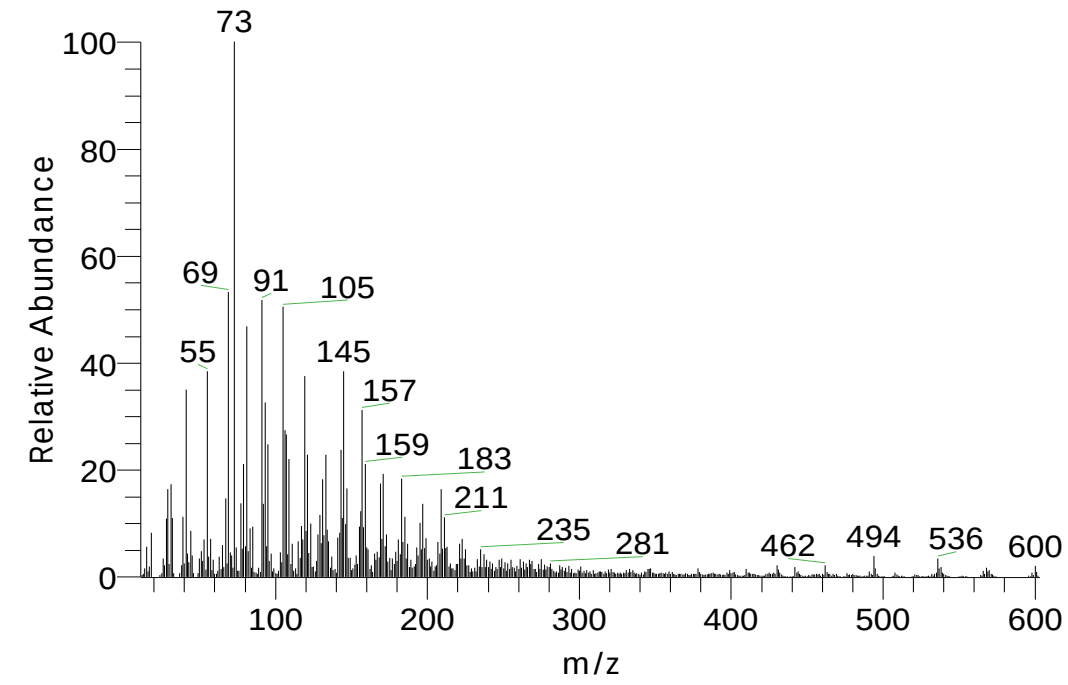

Formula C27H56O4Si2, MW 500, CAS# 54284-48-9, Entry# 285456
2-MONOOLEOYLGLYCEROL TRIMETHYLSILYL ETHER

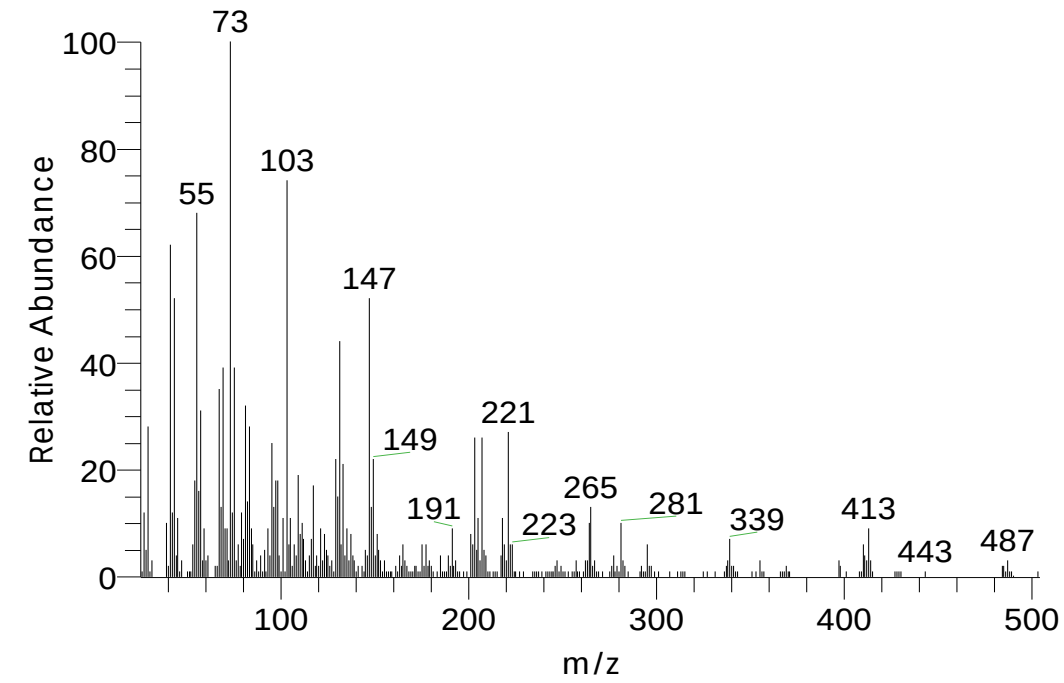

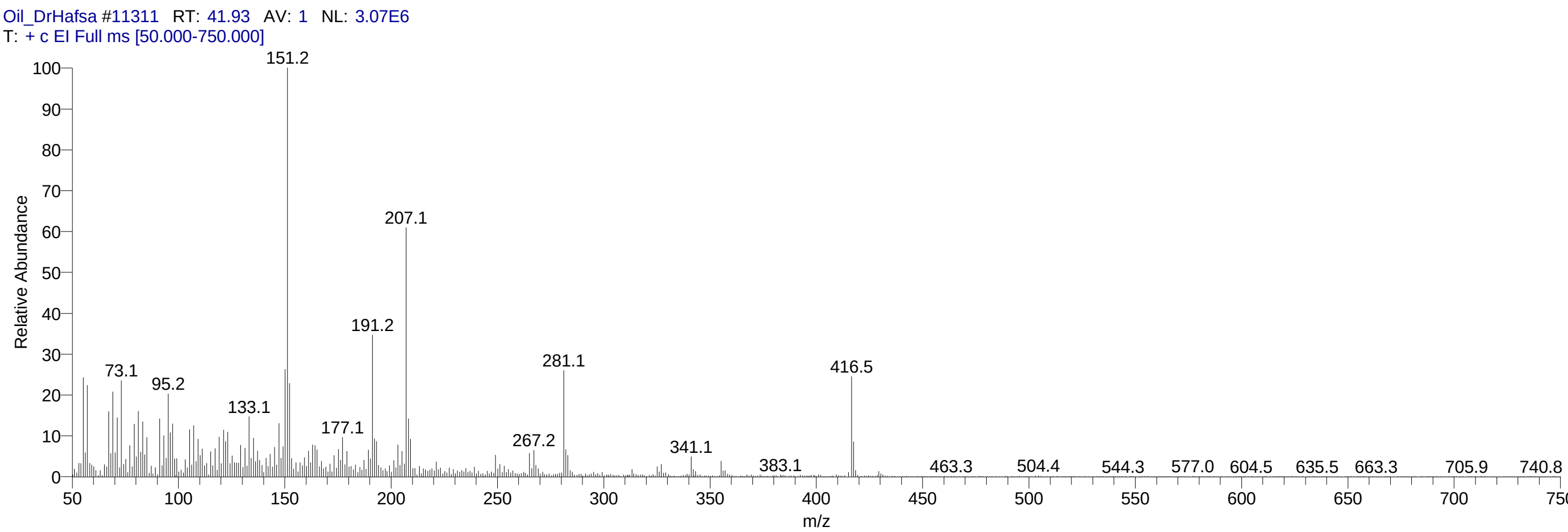

RT	Compound Name	Area %	MF	Molecular Formula	Molecular Weight	Cas #	Library
41.93	Loperamide	1.01	616	C29H33ClN2O2	476	34552-83-5	CaymanSpectralLibrary-NIST.HP
41.93	W-18	1.01	610	C19H20ClN3O4S	421	93101-02-1	CaymanSpectralLibrary-NIST.HP
41.93	5-fluoro ADB metabolite 7	1.01	591	C19H26FN3O3	363	NA	CaymanSpectralLibrary-NIST.HP
41.93	CHLOROANTHRAQUINONE	1.01	712	C14H7ClO2	242	26264-07-3	WileyRegistry8e
41.93	ARABINITOL, PENTAACETATE	1.01	690	C15H22O10	362	26674-23-7	WileyRegistry8e

My GC-MS Report

Compound Structure | Hit Spectrum

Loperamide
Formula C29H33ClN2O2, MW 476, CAS# 34552-83-5, Entry# 1181

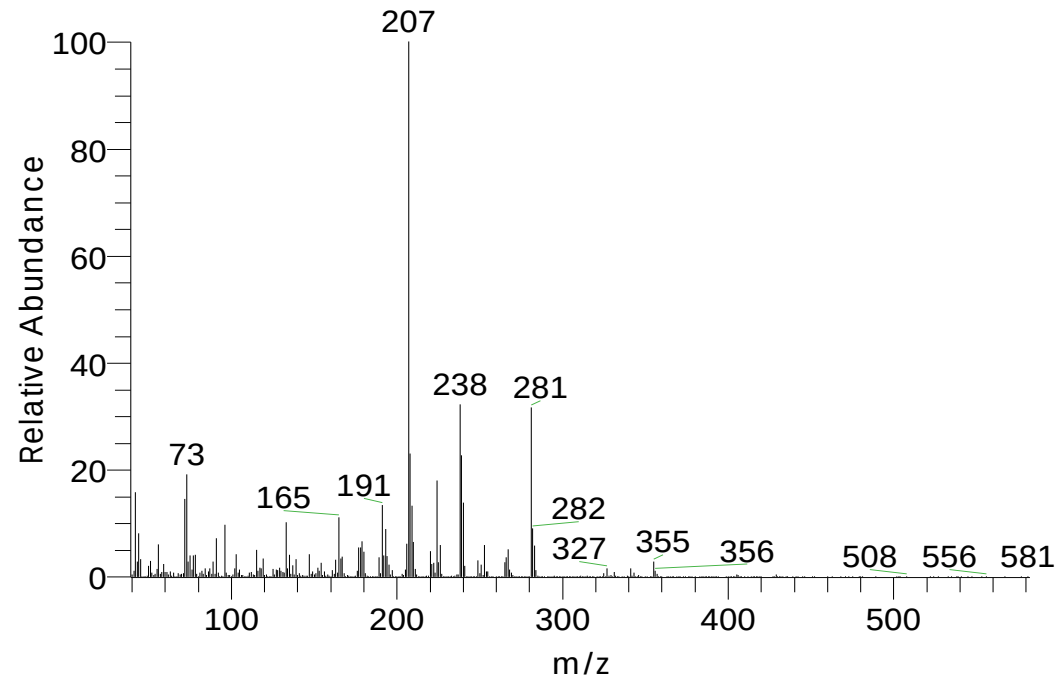

W-18
Formula C19H20ClN3O4S, MW 421, CAS# 93101-02-1, Entry# 949

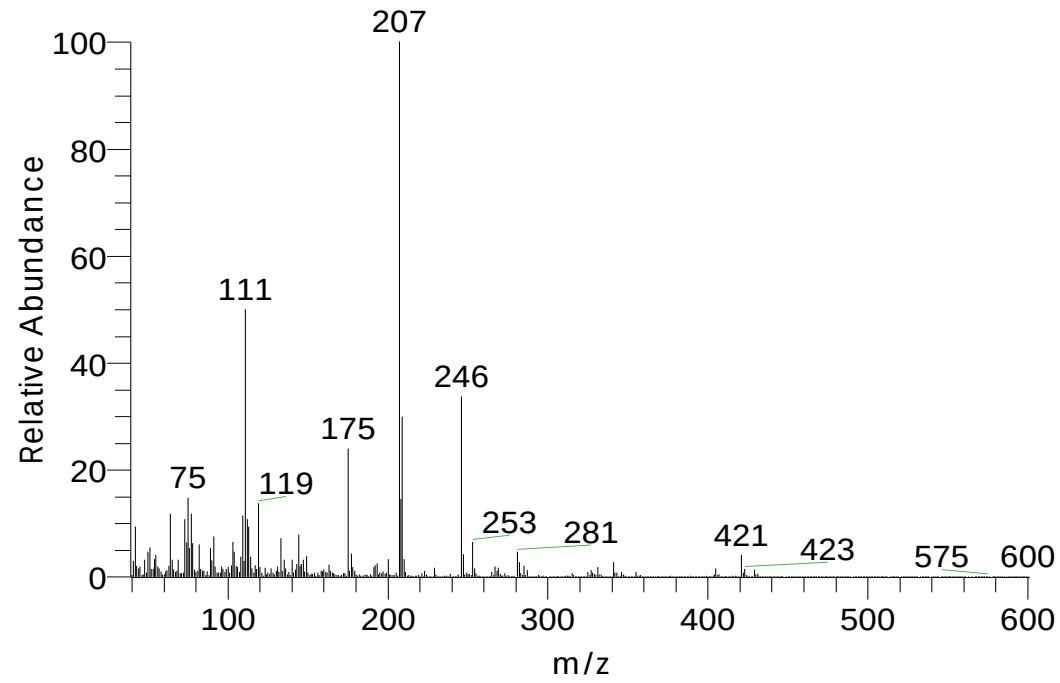

5-fluoro ADB metabolite 7
Formula C19H26FN3O3, MW 363, CAS# NA, Entry# 1218

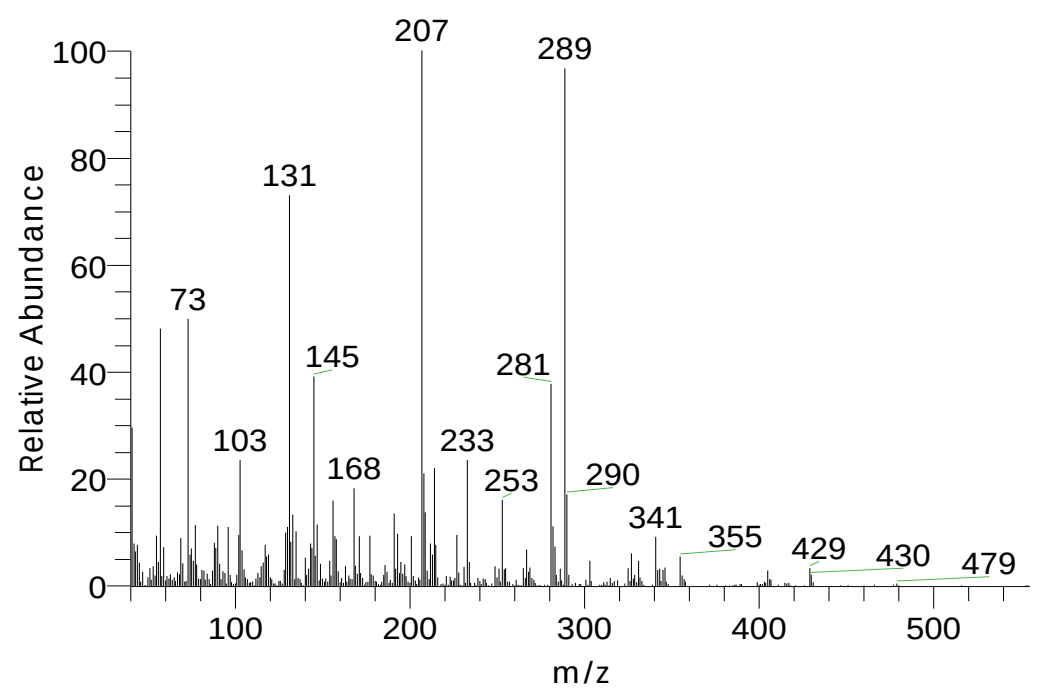

CHLOROANTHRAQUINONE
Formula C14H7ClO2, MW 242, CAS# 26264-07-3, Entry# 131613

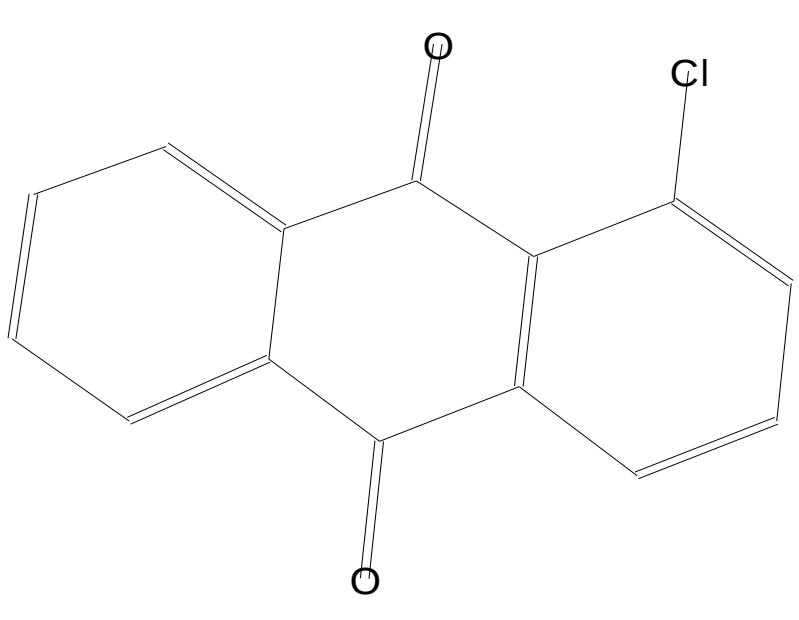

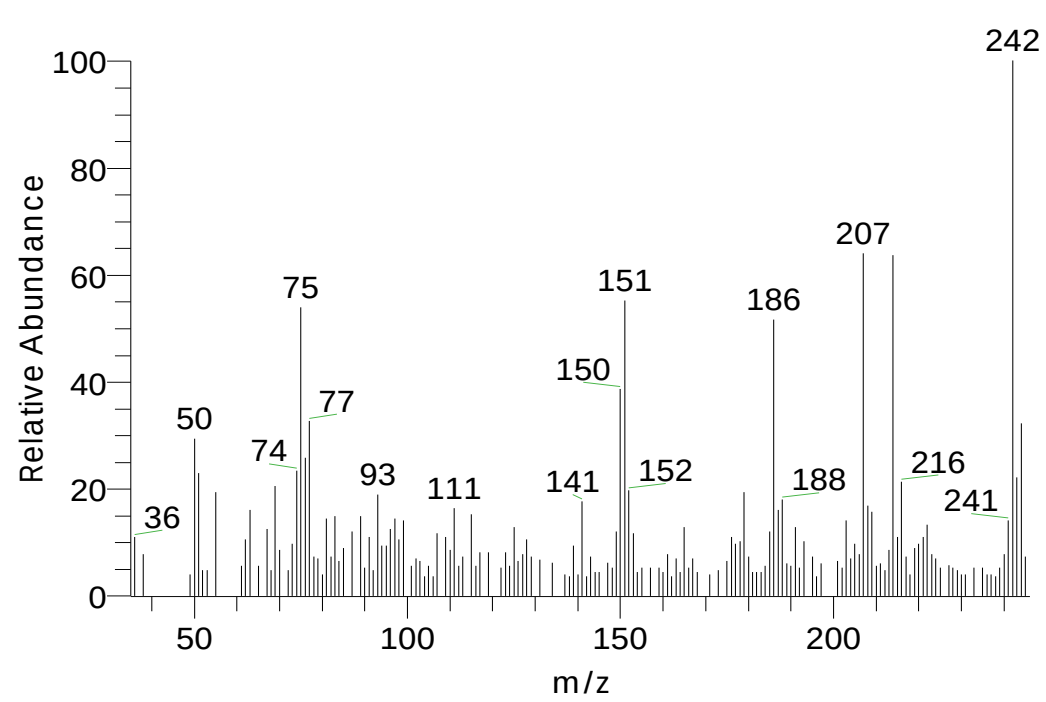

My GC-MS Report

Compound Structure | Hit Spectrum

ARABINITOL, PENTAACETATE
Formula C15H22O10, MW 362, CAS# 26674-23-7, Entry# 235892
1,2,3,4,5-PENTA-O-ACETYLPENTITOL #

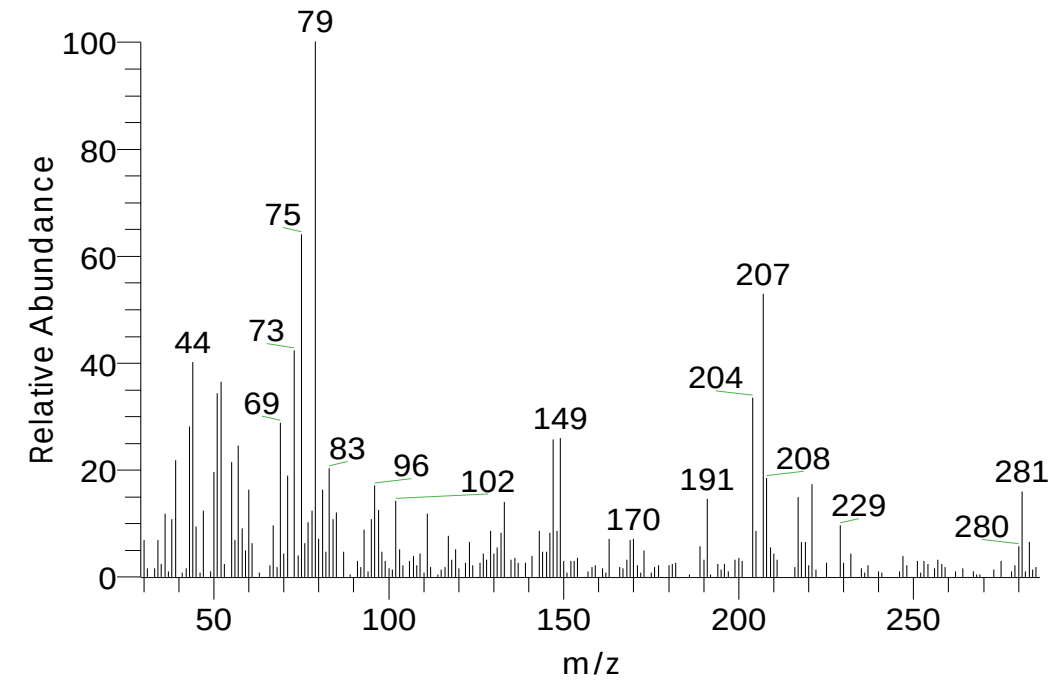

Oil_DrHafsa #11383 RT: 42.17 AV: 1 NL: 1.86E6
T: + c EI Full ms [50.000-750.000]

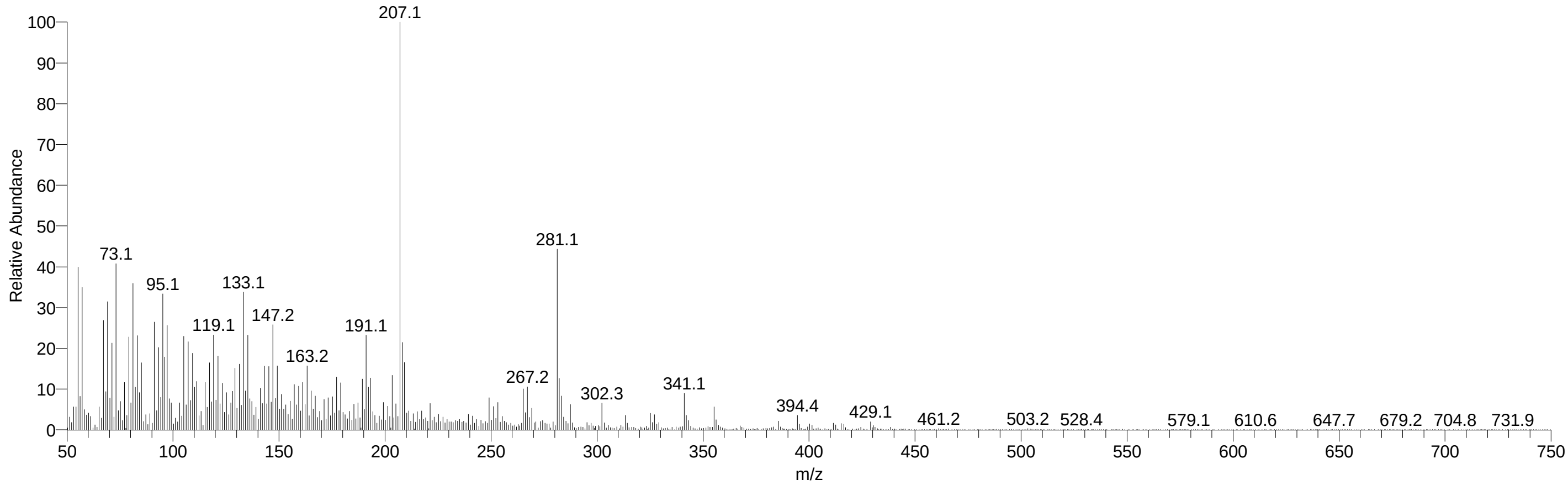

RT	Compound Name	Area %	MF	Molecular Formula	Molecular Weight	Cas #	Library
42.17	Ethyl iso-allocholate	0.54	765	C26H44O5	436	NA	mainlib
42.17	ETHYL ISO-ALLOCHOLATE	0.54	765	C26H44O5	436	NA	WileyRegistry8e
42.17	4H-1-BENZOPYRAN-4-ONE, 2-(3,4-DIMETHOXYPHENYL)-3,5-DIHYDROXY-7-METHOXY-	0.54	758	C18H16O7	344	6068-80-0	WileyRegistry8e
42.17	9,12-OCTADECADIENOIC ACID (Z,Z)-, 2,3-BIS[(TRIMETHYLSILYL)OXY]PROPYL ESTER	0.54	787	C27H54O4Si2	498	54284-45-6	WileyRegistry8e
42.17	03027205002 FLAVONE 4'-OH,5-OH,7-DI-O-GLUCOSIDE	0.54	723	C27H30O15	594	NA	WileyRegistry8e

Compound Structure | Hit Spectrum

Ethyl iso-allocholate
Formula C26H44O5, MW 436, CAS# NA, Entry# 7020
Ethyl 3,7,12-trihydroxycholan-24-oate #

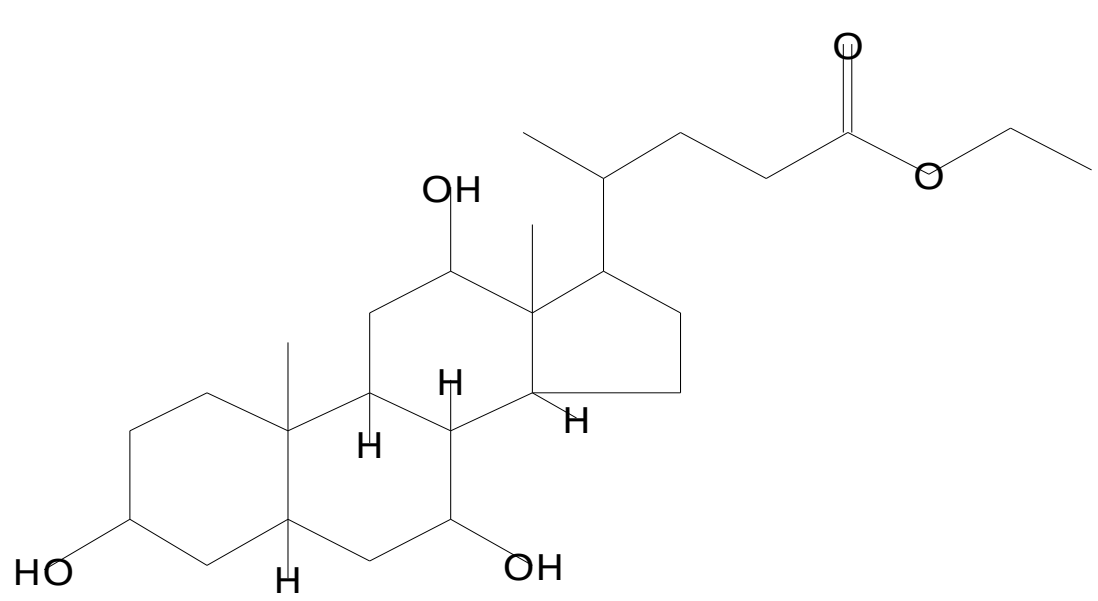

SI 752, RSI 765, mainlib, Entry# 7020, CAS# NA, Ethyl iso-allocholate

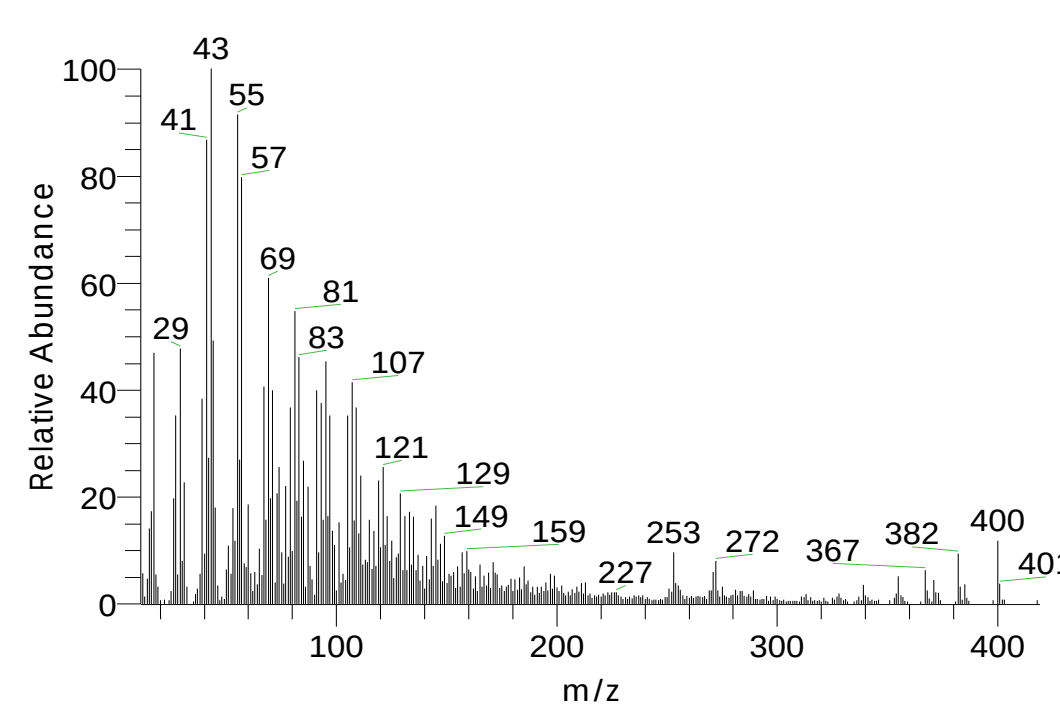

My GC-MS Report

Compound Structure | Hit Spectrum

ETHYL ISO-ALLOCHOLATE
Formula C26H44O5, MW 436, CAS# NA, Entry# 270212

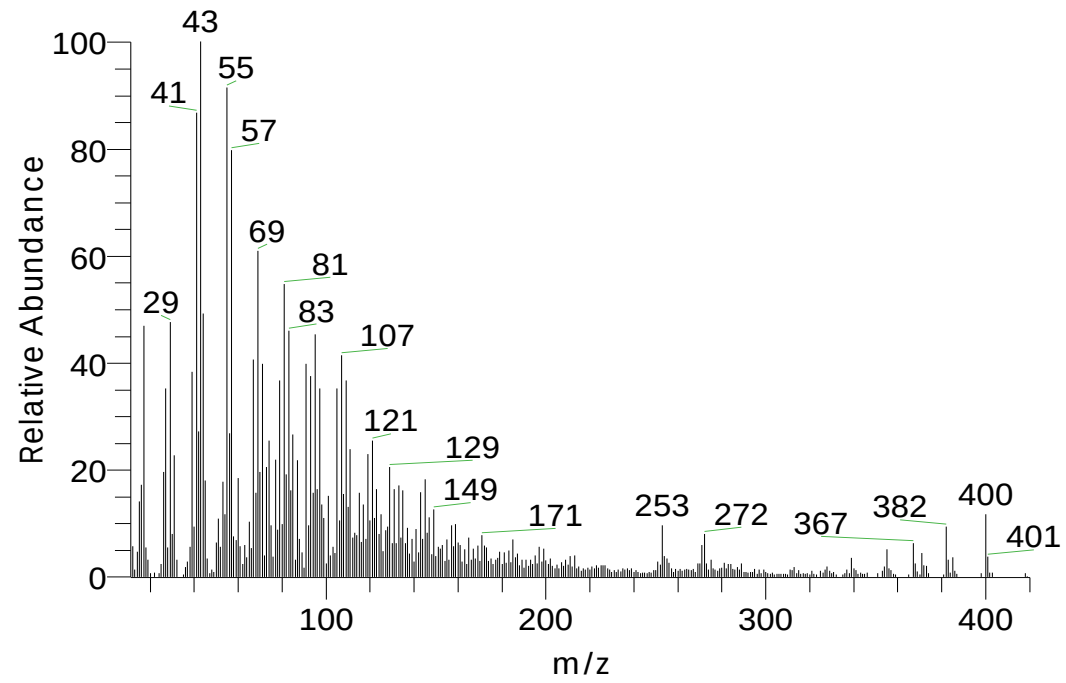

4H-1-BENZOPYRAN-4-ONE, 2-(3,4-DIMETHOXYPHENYL)-3,5-DIHYDROXY-7-METHOXY-
Formula C18H16O7, MW 344, CAS# 6068-80-0, Entry# 224392
3',4',7-TRIMETHYLQUERCETIN

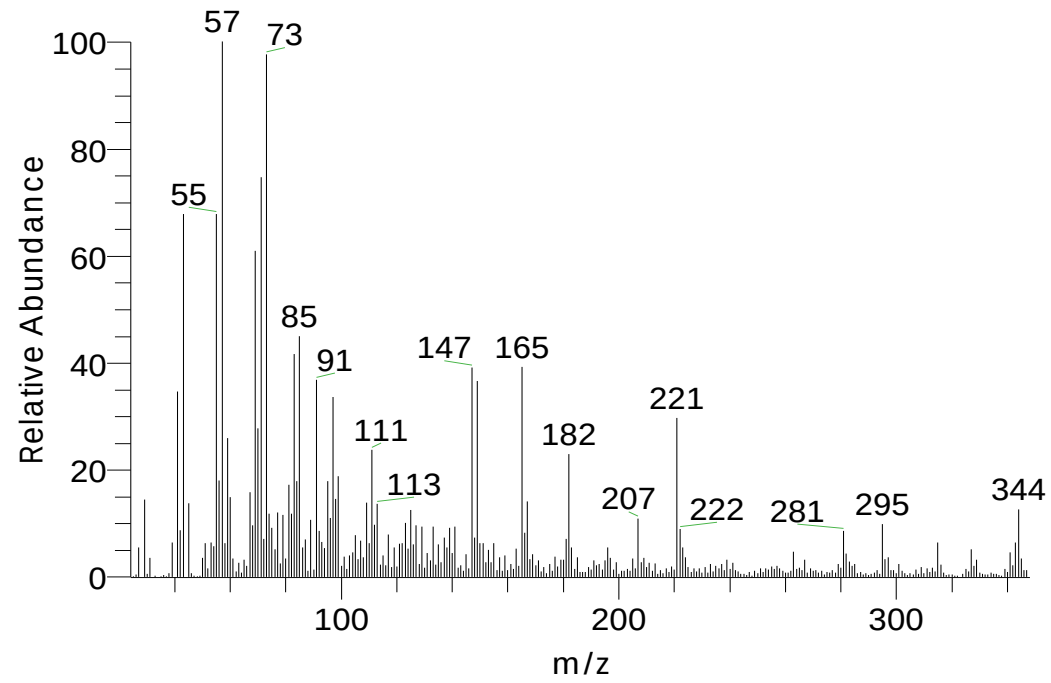

9,12-OCTADECADIENOIC ACID (Z,Z)-, 2,3-BIS[(TRIMETHYLSILYL)OXY]PROPYL ESTER
Formula C27H54O4Si2, MW 498, CAS# 54284-45-6, Entry# 285148
2,3-BIS[(TRIMETHYLSILYL)OXY]PROPYL (9Z,12Z)-9,12-OCTADECADIENOATE #

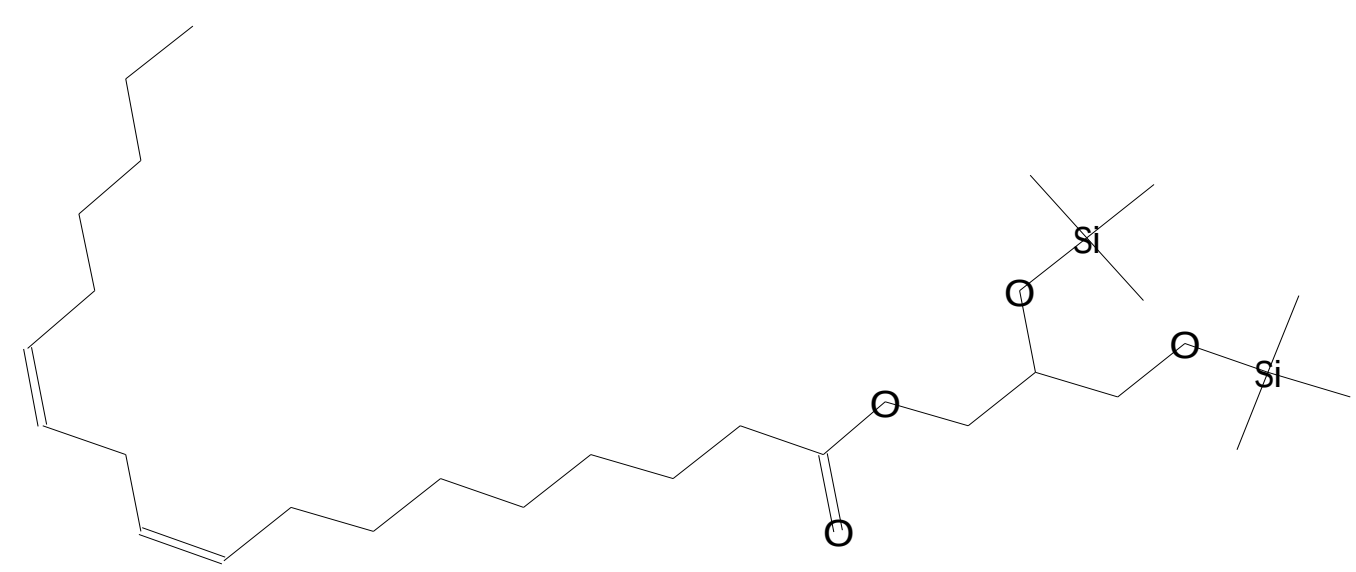

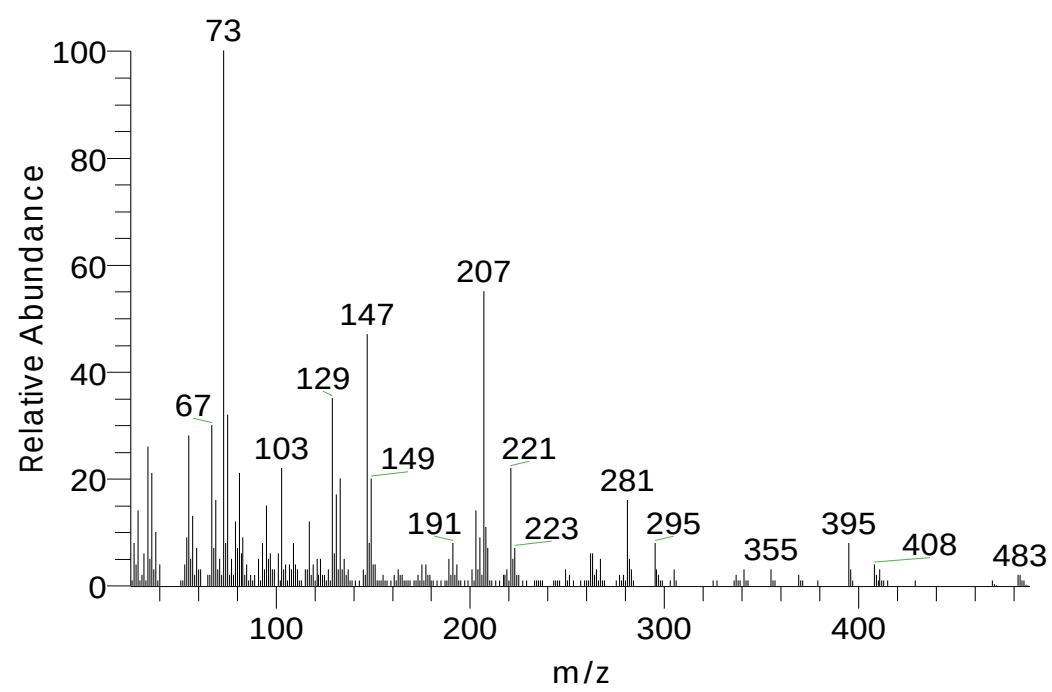

03027205002 FLAVONE 4'-OH,5-OH,7-DI-O-GLUCOSIDE
Formula C27H30O15, MW 594, CAS# NA, Entry# 296184

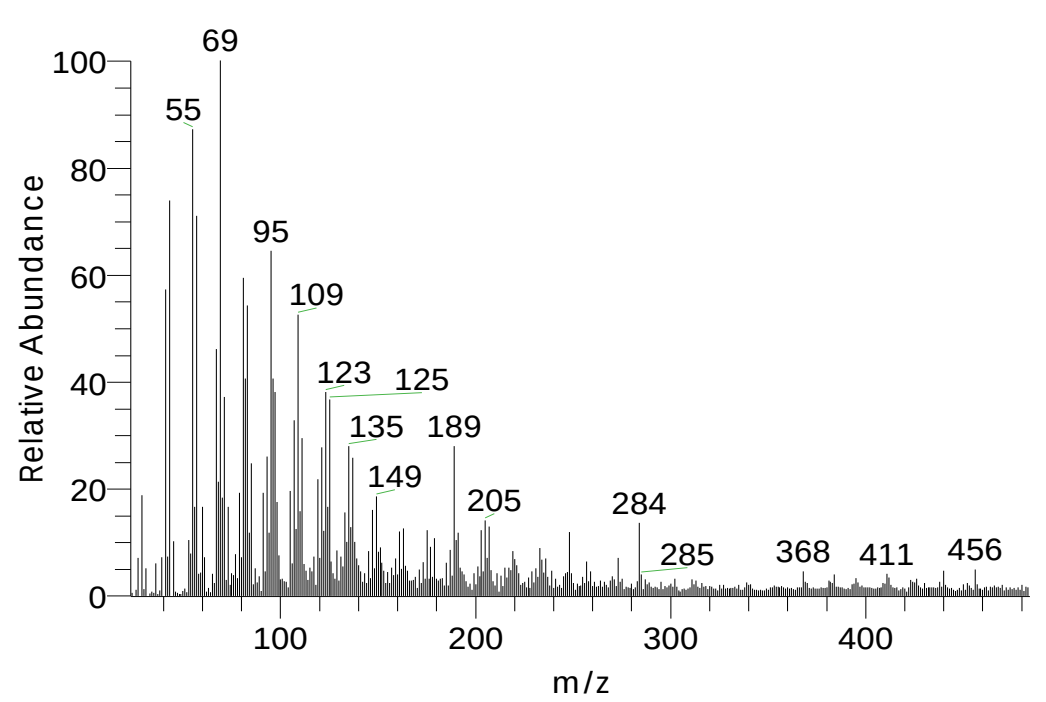

My GC-MS Report

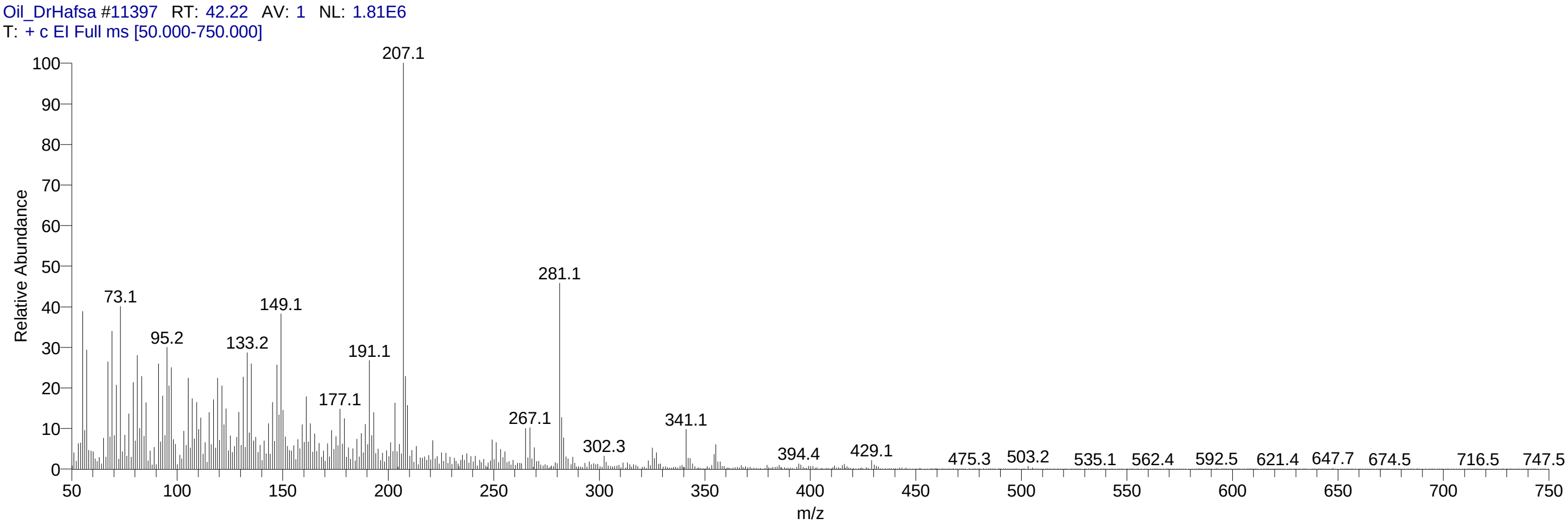

RT	Compound Name	Area %	MF	Molecular Formula	Molecular Weight	Cas #	Library
42.22	9,12-OCTADECADIENOIC ACID (Z,Z)-, 2,3-BIS[(TRIMETHYLSILYL)OXY]PROPYL ESTER	0.72	796	C27H54O4Si2	498	54284-45-6	WileyRegistry8e
42.22	9,12,15-OCTADECATRIENOIC ACID, 2,3-BIS[(TRIMETHYLSILYL)OXY]PROPYL ESTER, (Z,Z,Z)-	0.72	787	C27H52O4Si2	496	55521-22-7	WileyRegistry8e
42.22	4H-1-BENZOPYRAN-4-ONE, 2-(3,4-DIMETHOXYPHENYL)-3,5-DIHYDROXY-7-METHOXY-	0.72	754	C18H16O7	344	6068-80-0	WileyRegistry8e
42.22	4H-1-BENZOPYRAN-4-ONE, 2-(3,4-DIHYDROXYPHENYL)-6,8-DI-á-D-GLUCOPYRANOSYL-5,7-DIHYDROXY-	0.72	730	C27H30O16	610	29428-58-8	WileyRegistry8e
42.22	.psi.,.psi.-Carotene, 1,1',2,2'-tetrahydro-1,1'-dimethoxy-	0.72	712	C42H64O2	600	13833-01-7	mainlib

Compound Structure	Hit Spectrum

9,12-OCTADECADIENOIC ACID (Z,Z)-, 2,3-BIS[(TRIMETHYLSILYL)OXY]PROPYL ESTER
Formula C27H54O4Si2, MW 498, CAS# 54284-45-6, Entry# 285148
2,3-BIS[(TRIMETHYLSILYL)OXY]PROPYL (9Z,12Z)-9,12-OCTADECADIENOATE #

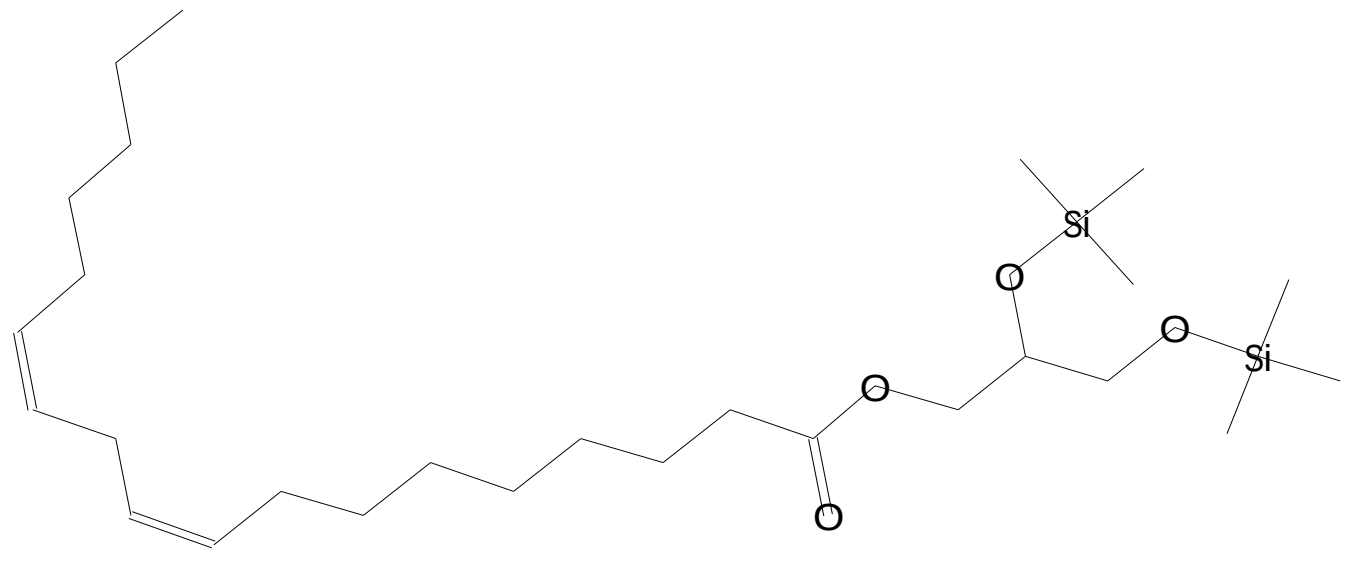

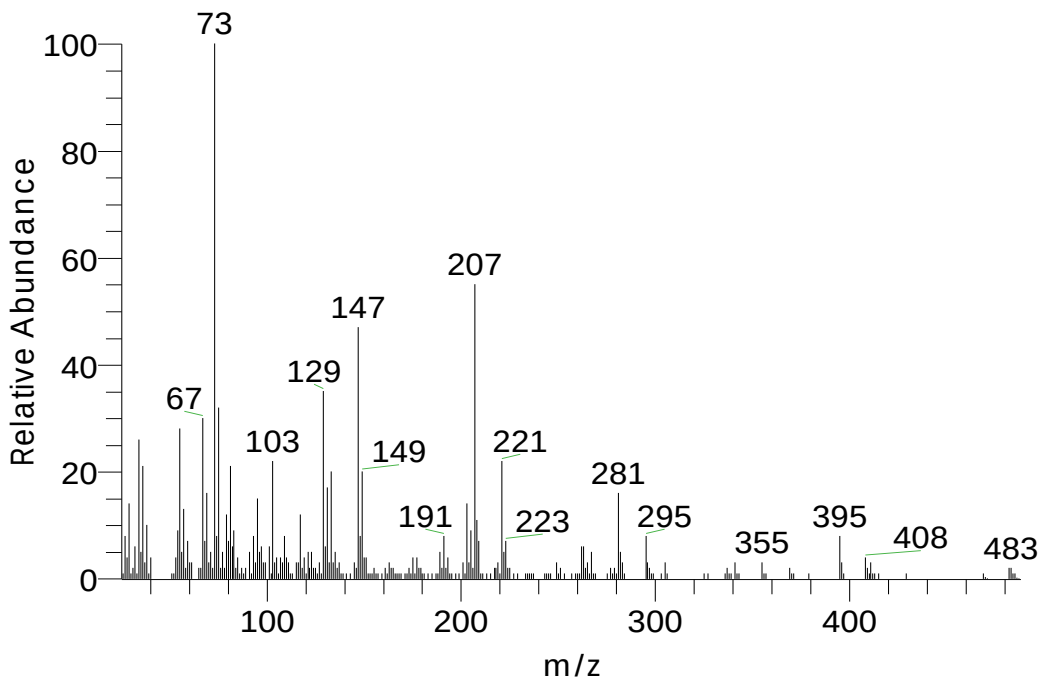

9,12,15-OCTADECATRIENOIC ACID, 2,3-BIS[(TRIMETHYLSILYL)OXY]PROPYL ESTER, (Z,Z,Z)-
Formula C27H52O4Si2, MW 496, CAS# 55521-22-7, Entry# 284834
2,3-BIS[(TRIMETHYLSILYL)OXY]PROPYL (9E,12E,15E)-9,12,15-OCTADECATRIENOATE #

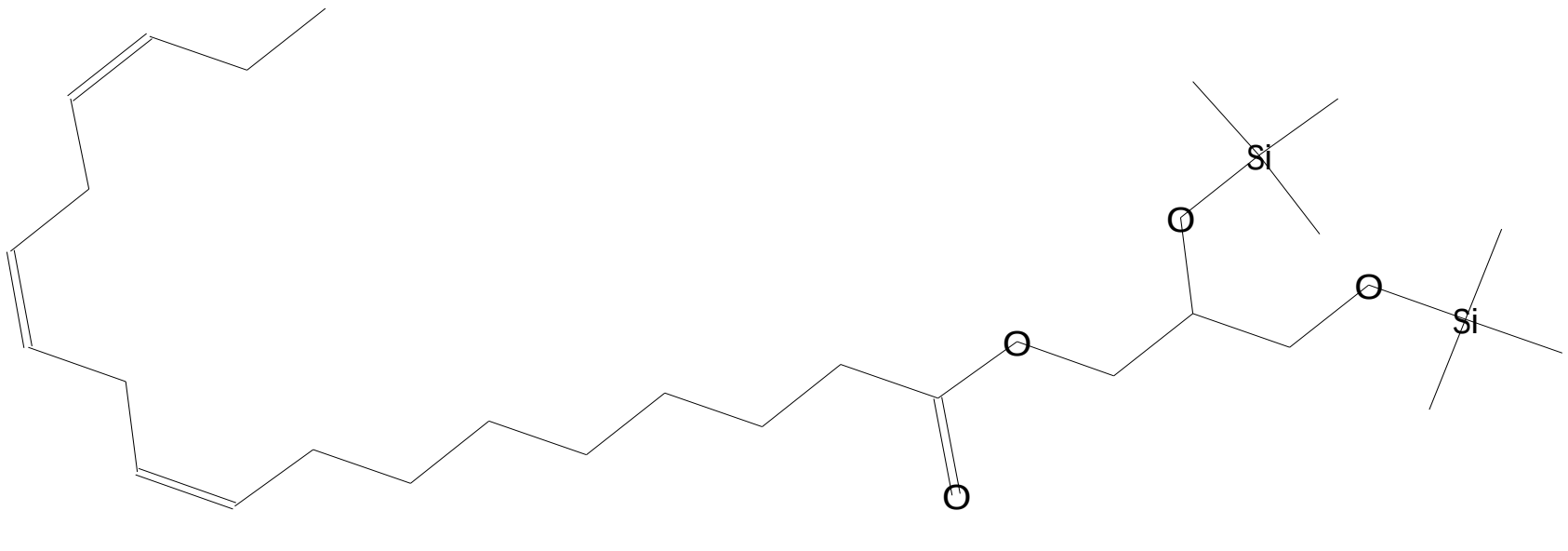

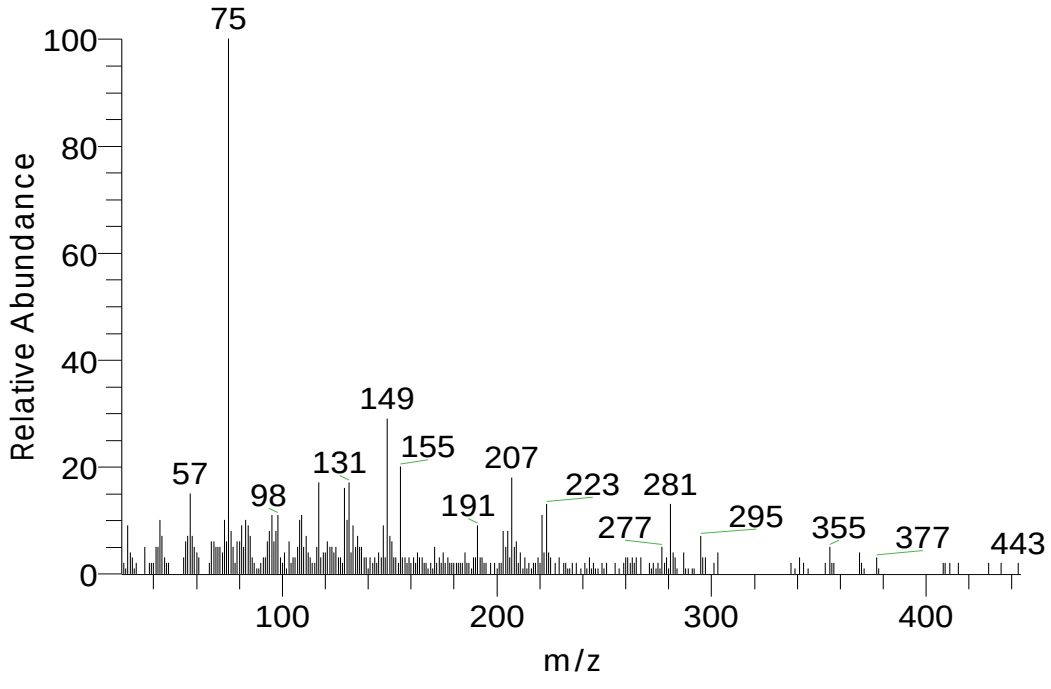

My GC-MS Report

Compound Structure	Hit Spectrum

4H-1-BENZOPYRAN-4-ONE, 2-(3,4-DIMETHOXYPHENYL)-3,5-DIHYDROXY-7-METHOXY-
Formula C18H16O7, MW 344, CAS# 6068-80-0, Entry# 224392
3',4',7-TRIMETHYLQUERCETIN

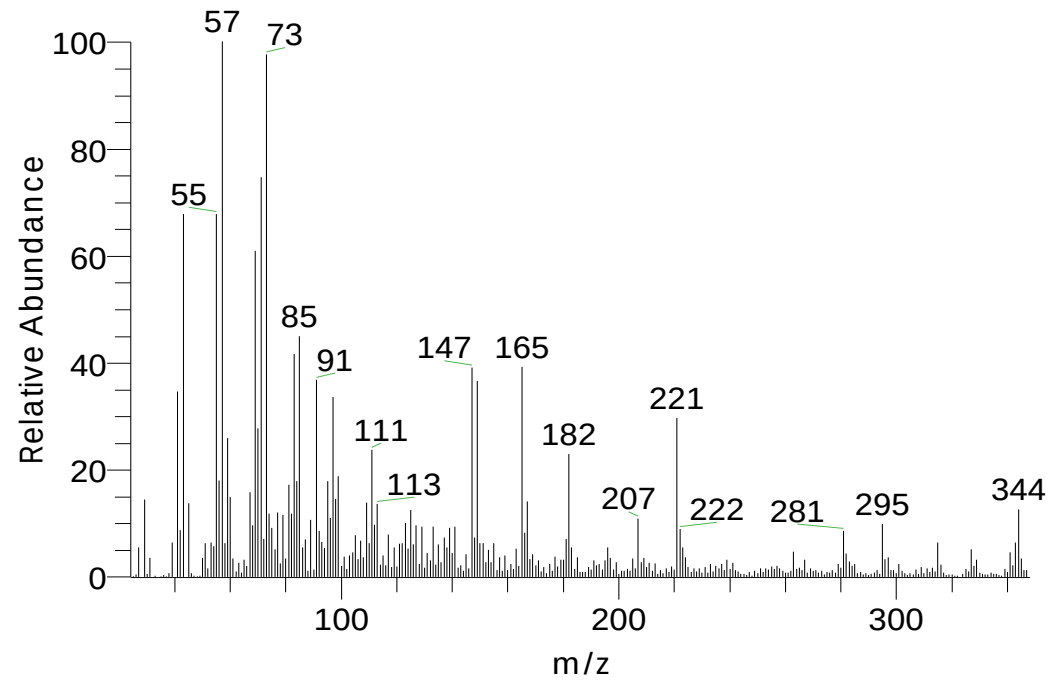

Formula C27H30O16, MW 610, CAS# 29428-58-8, Entry# 297453
6,8-DI-C-á-GLUCOSYLLUTEOLIN

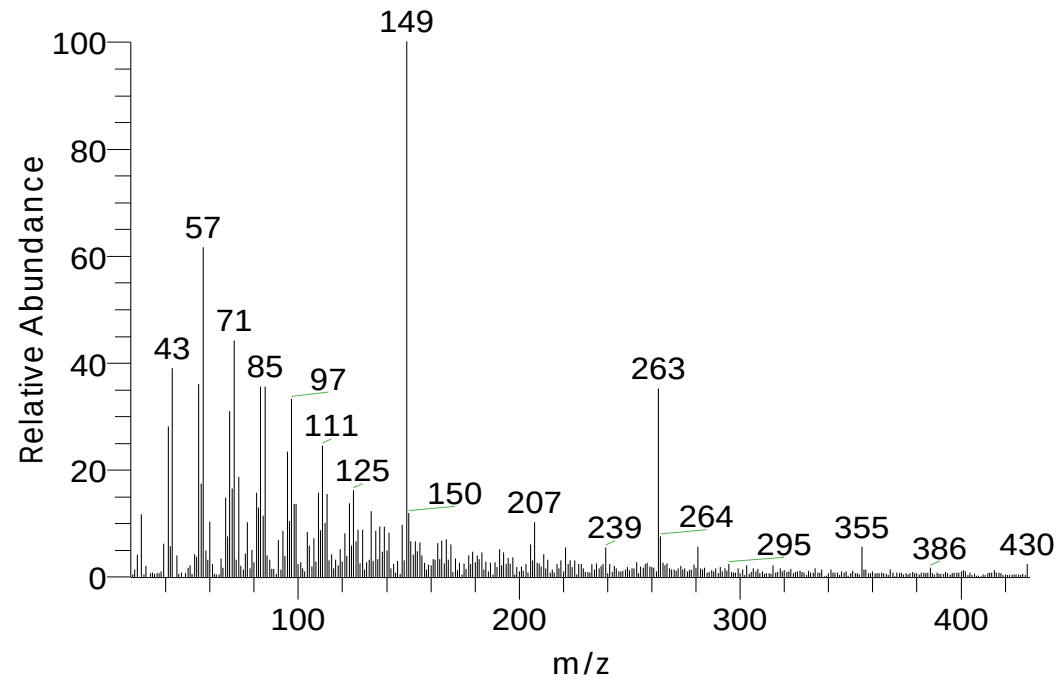

.psi.,.psi.-Carotene, 1,1',2,2'-tetrahydro-1,1'-dimethoxy-
Formula C42H64O2, MW 600, CAS# 13833-01-7, Entry# 41205
Lycopene, 1,1',2,2'-tetrahydro-1,1'-dimethoxy-, all-trans-

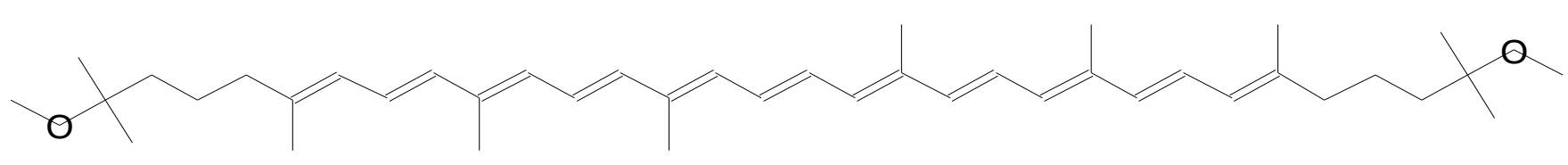

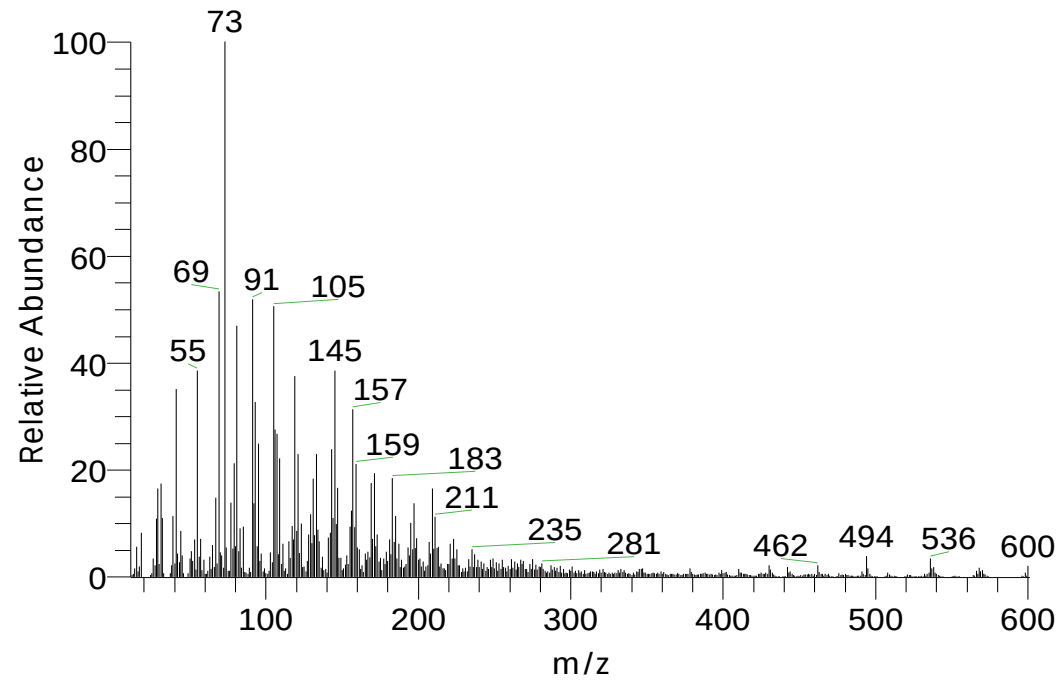

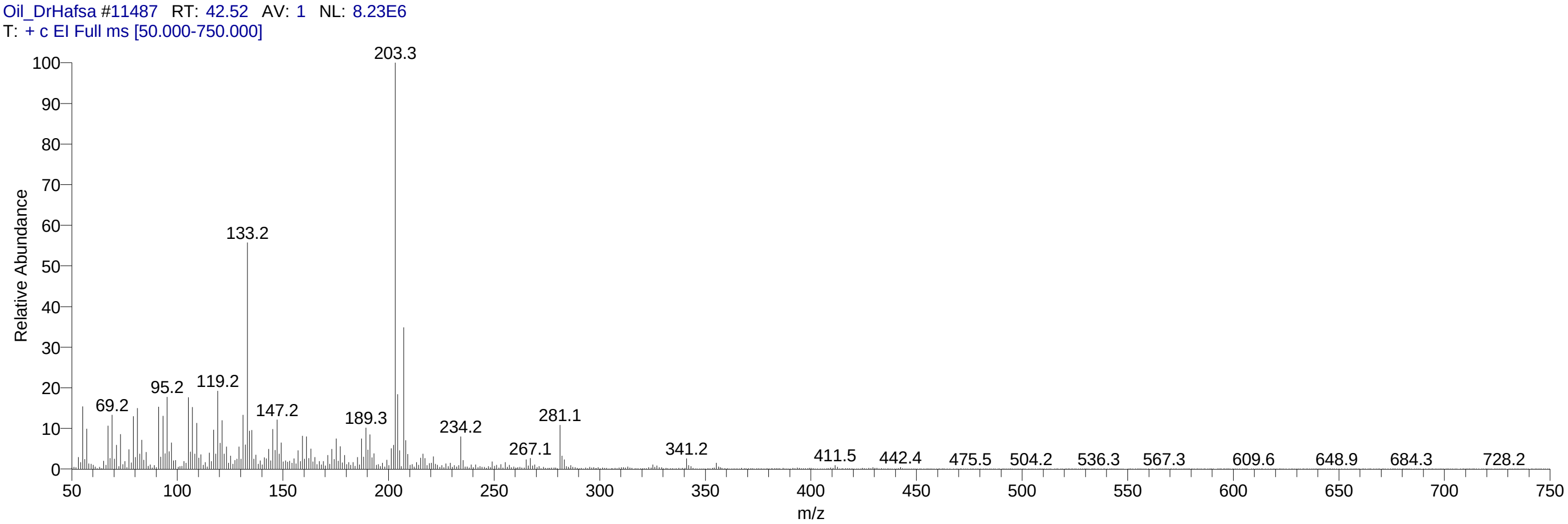

RT	Compound Name	Area %	MF	Molecular Formula	Molecular Weight	Cas #	Library
42.52	Uvaol	12.17	851	C30H50O2	442	545-46-0	mainlib
42.52	Betulin	12.17	784	C30H50O2	442	473-98-3	replib

My GC-MS Report

RT	Compound Name	Area %	MF	Molecular Formula	Molecular Weight	Cas #	Library
42.52	Olean-12-ene-3,28-diol, (3á)-	12.17	831	C30H50O2	442	545-48-2	mainlib
42.52	OLEAN-12-ENE-3,28-DIOL, (3á)-	12.17	830	C30H50O2	442	545-48-2	WileyRegistry8e
42.52	LUP-20(29)-ENE-3,28-DIOL, (3á)-	12.17	802	C30H50O2	442	473-98-3	WileyRegistry8e

Compound Structure | Hit Spectrum

Uvaol
Formula C30H50O2, MW 442, CAS# 545-46-0, Entry# 186336
Urs-12-ene-3,28-diol, (3á)-

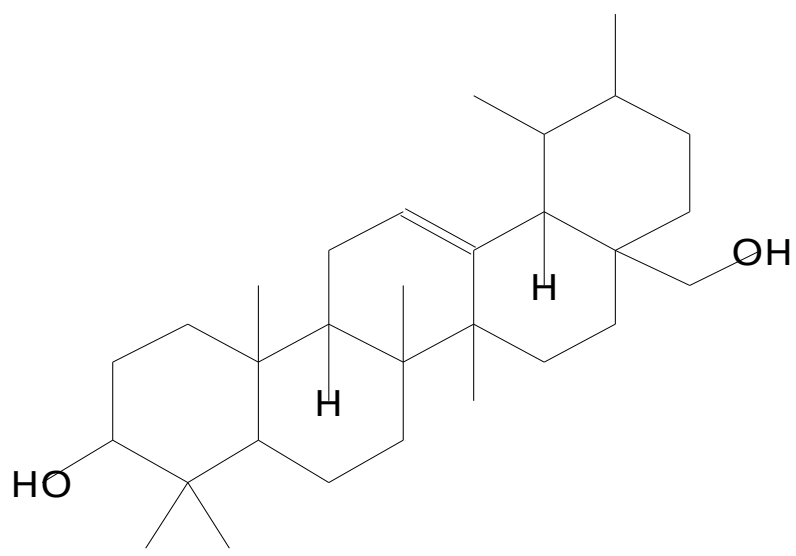

SI 794, RSI 851, mainlib, Entry# 186336, CAS# 545-46-0, Uvaol

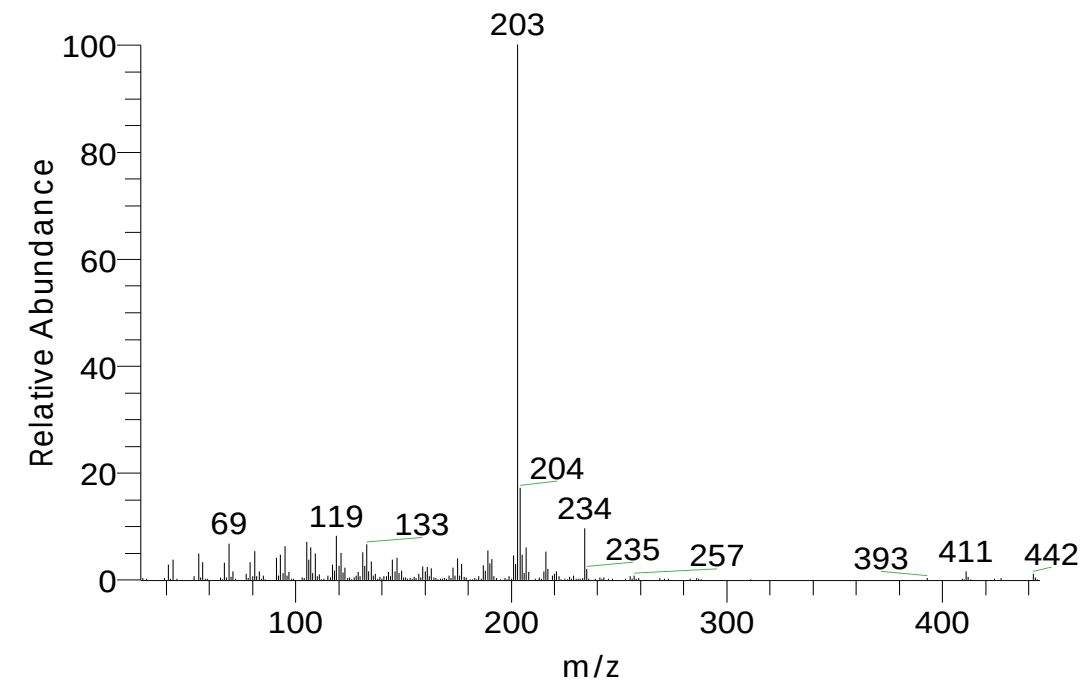

Betulin
Formula C30H50O2, MW 442, CAS# 473-98-3, Entry# 27738
Lup-20(29)-ene-3,28-diol, (3á)-

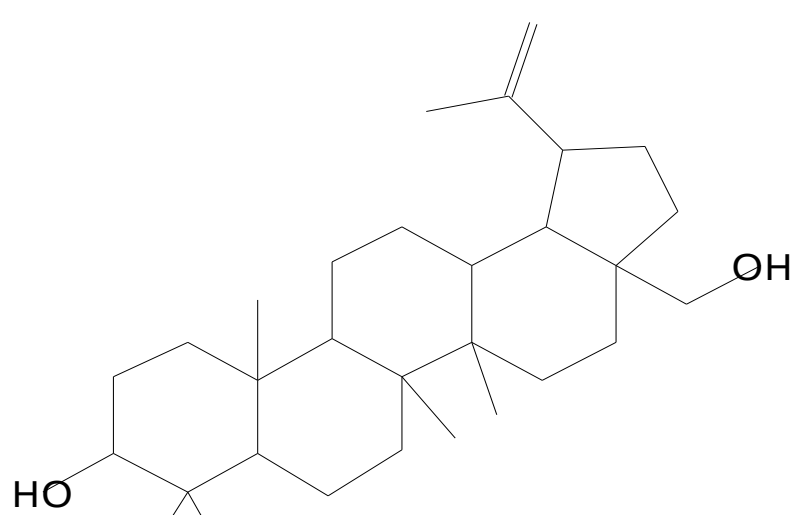

SI 783, RSI 784, replib, Entry# 27738, CAS# 473-98-3, Betulin

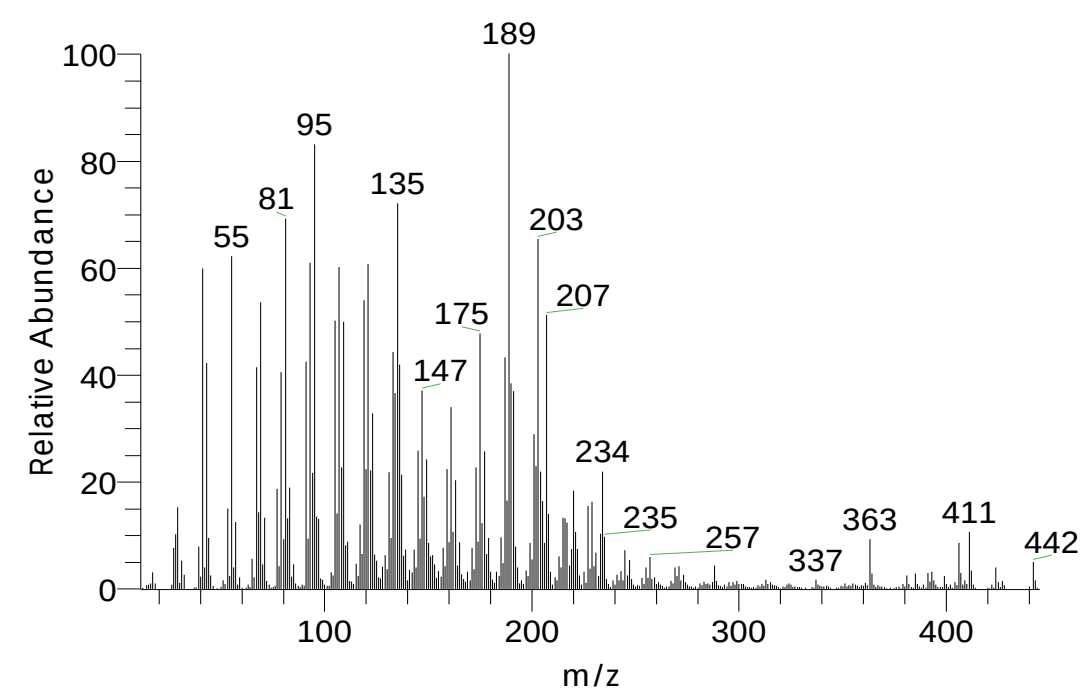

Olean-12-ene-3,28-diol, (3á)-
Formula C30H50O2, MW 442, CAS# 545-48-2, Entry# 185945
Olean-12-ene-3á,28-diol

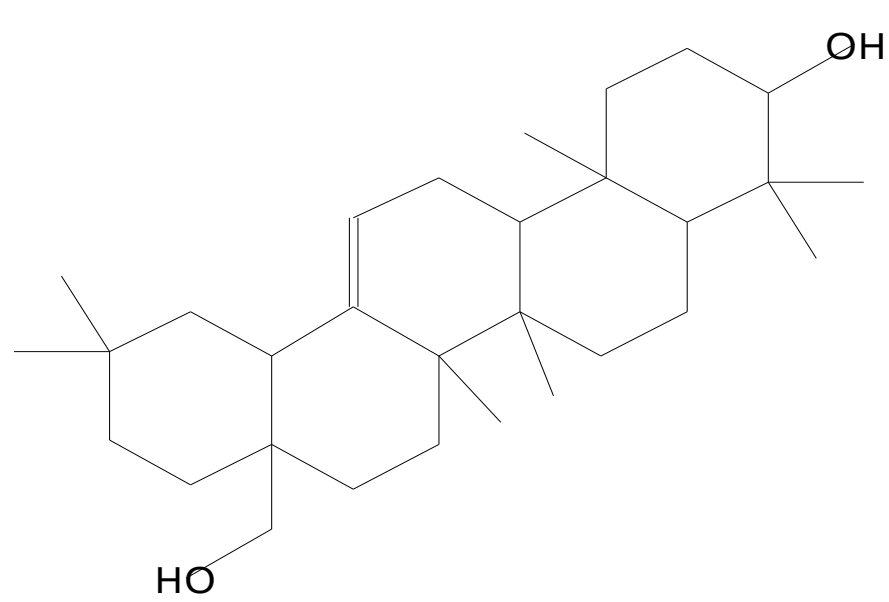

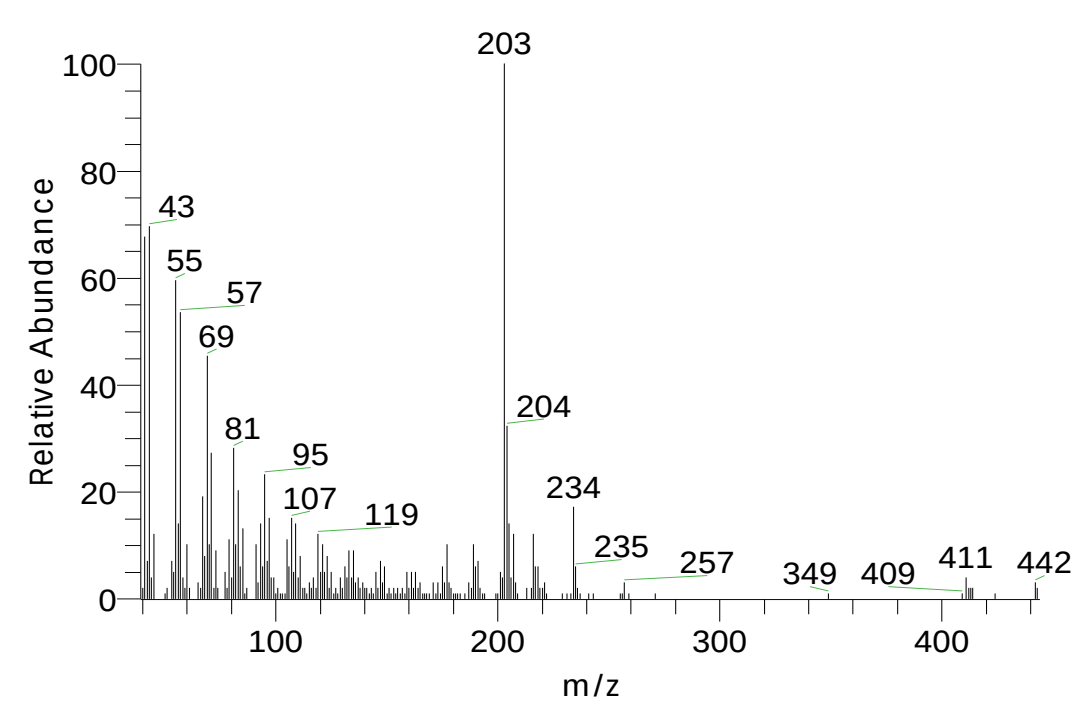

OLEAN-12-ENE-3,28-DIOL, (3á)-
Formula C30H50O2, MW 442, CAS# 545-48-2, Entry# 272140
OLEAN-12-ENE-3,28-DIOL #

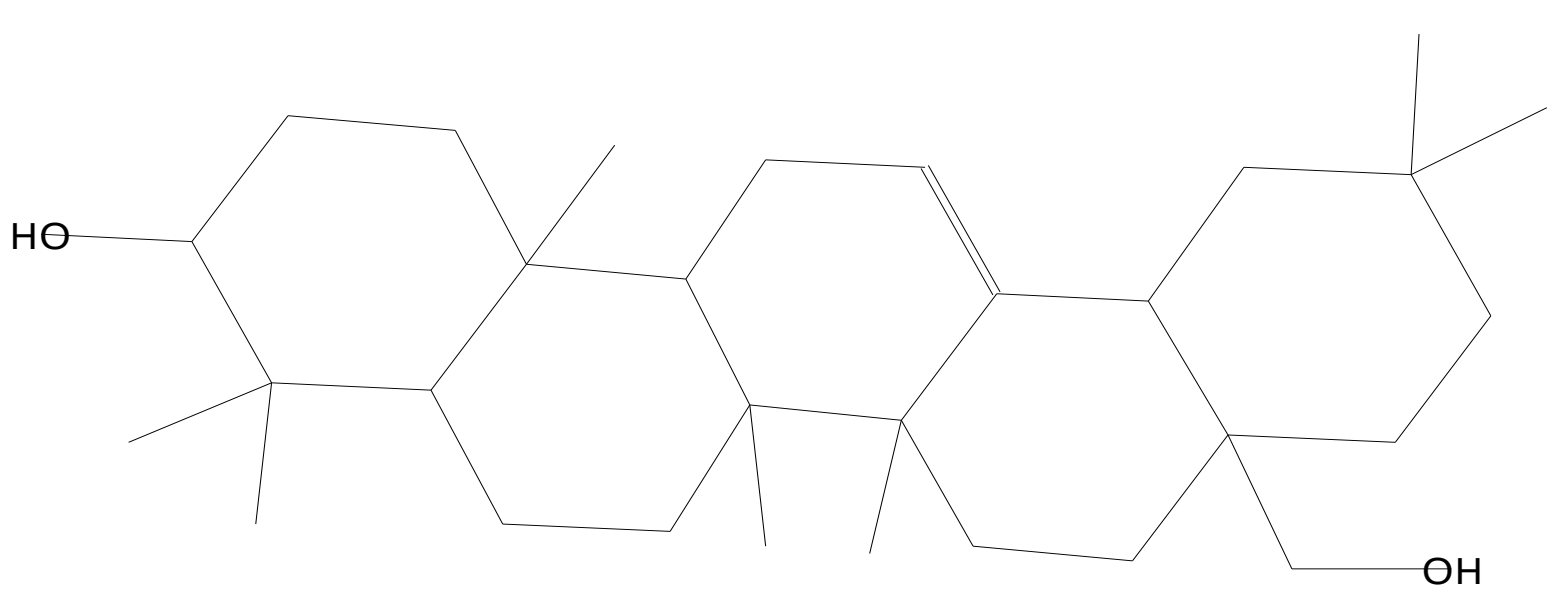

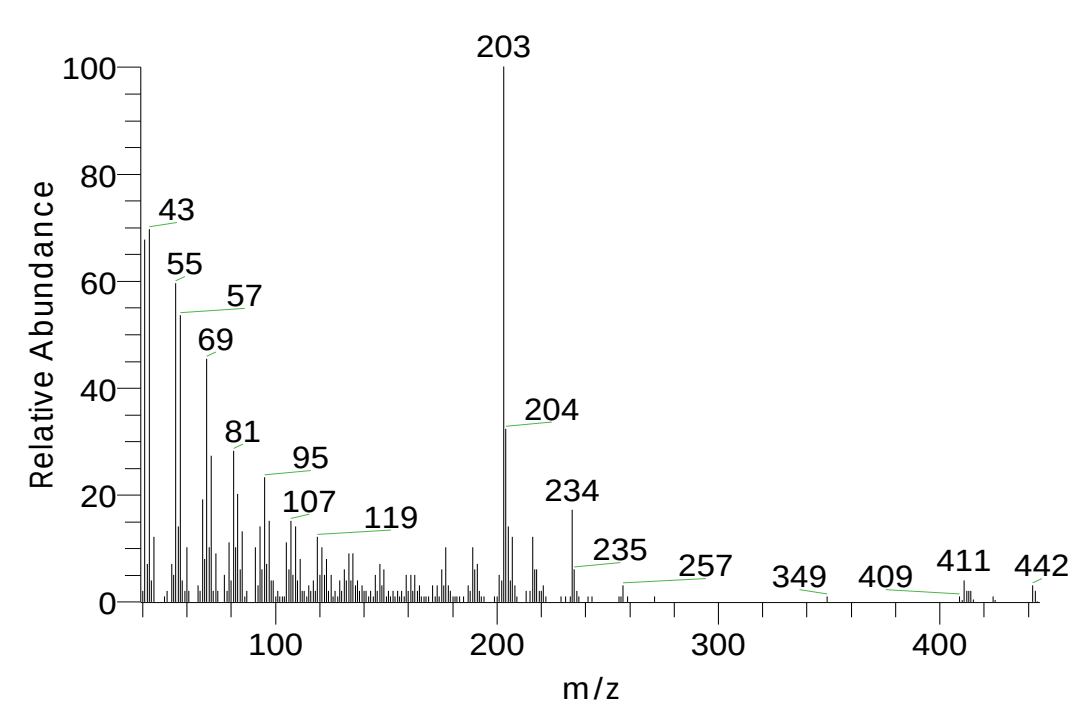

My GC-MS Report

Compound Structure | Hit Spectrum

LUP-20(29)-ENE-3,28-DIOL, (3á)-
Formula C30H50O2, MW 442, CAS# 473-98-3, Entry# 349524
LUP-20(29)-ENE-3,28-DIOL #

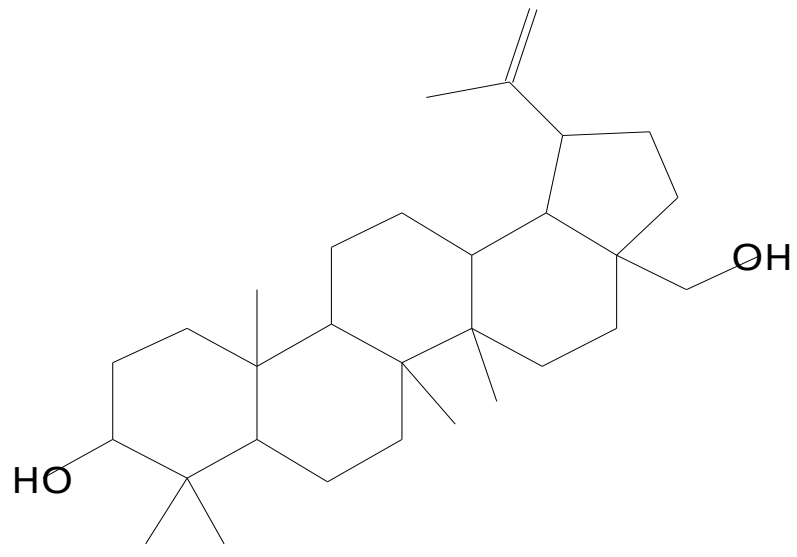

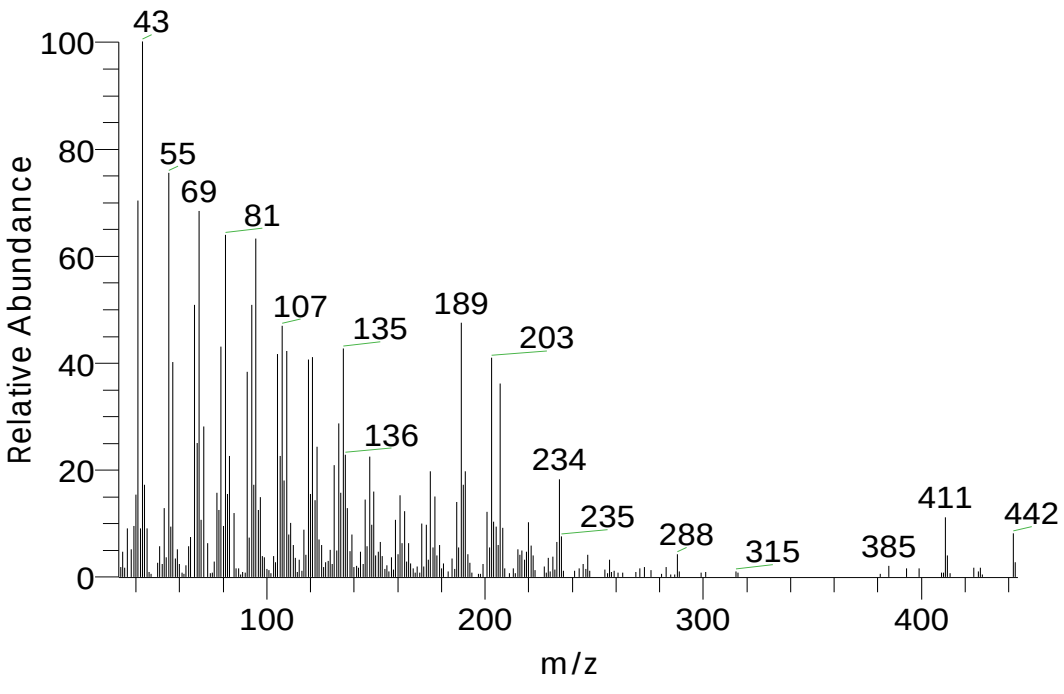

Oil_DrHafsa #11539 RT: 42.69 AV: 1 NL: 1.88E6
T: + c EI Full ms [50.000-750.000]

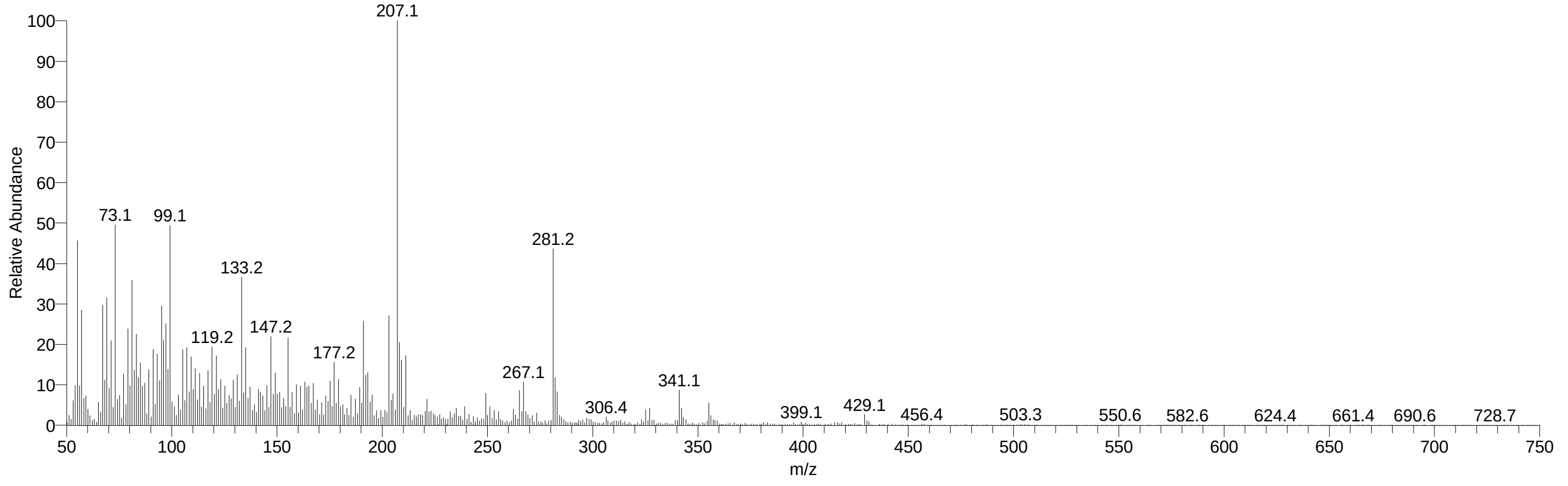

RT	Compound Name	Area %	MF	Molecular Formula	Molecular Weight	Cas #	Library
42.69	9,12,15-OCTADECATRIENOIC ACID, 2,3-BIS[(TRIMETHYLSILYL)OXY]PROPYL ESTER, (Z,Z,Z)-	0.84	783	C27H52O4Si2	496	55521-22-7	WileyRegistry8e
42.69	9,12-OCTADECADIENOIC ACID (Z,Z)-, 2,3-BIS[(TRIMETHYLSILYL)OXY]PROPYL ESTER	0.84	767	C27H54O4Si2	498	54284-45-6	WileyRegistry8e
42.69	9-OCTADECENOIC ACID (Z)-, 2-[(TRIMETHYLSILYL)OXY]-1-[[(TRIMETHYLSILYL)OXY]METHYL]ETHYL ESTER	0.84	733	C27H56O4Si2	500	54284-48-9	WileyRegistry8e
42.69	à-D-GALACTOPYRANOSIDE, METHYL 2,3-BIS-O-(TRIMETHYLSILYL)-, CYCLIC BUTYLBORONATE	0.84	734	C17H37BO6Si2	404	56211-11-1	WileyRegistry8e
42.69	Loperamide	0.84	654	C29H33ClN2O2	476	34552-83-5	CaymanSpectralLibrary-NIST.HP

My GC-MS Report

Compound Structure | Hit Spectrum

9,12,15-OCTADECATRIENOIC ACID, 2,3-BIS[(TRIMETHYLSILYL)OXY]PROPYL ESTER, (Z,Z,Z)-
Formula C27H52O4Si2, MW 496, CAS# 55521-22-7, Entry# 284834
2,3-BIS[(TRIMETHYLSILYL)OXY]PROPYL (9E,12E,15E)-9,12,15-OCTADECATRIENOATE #

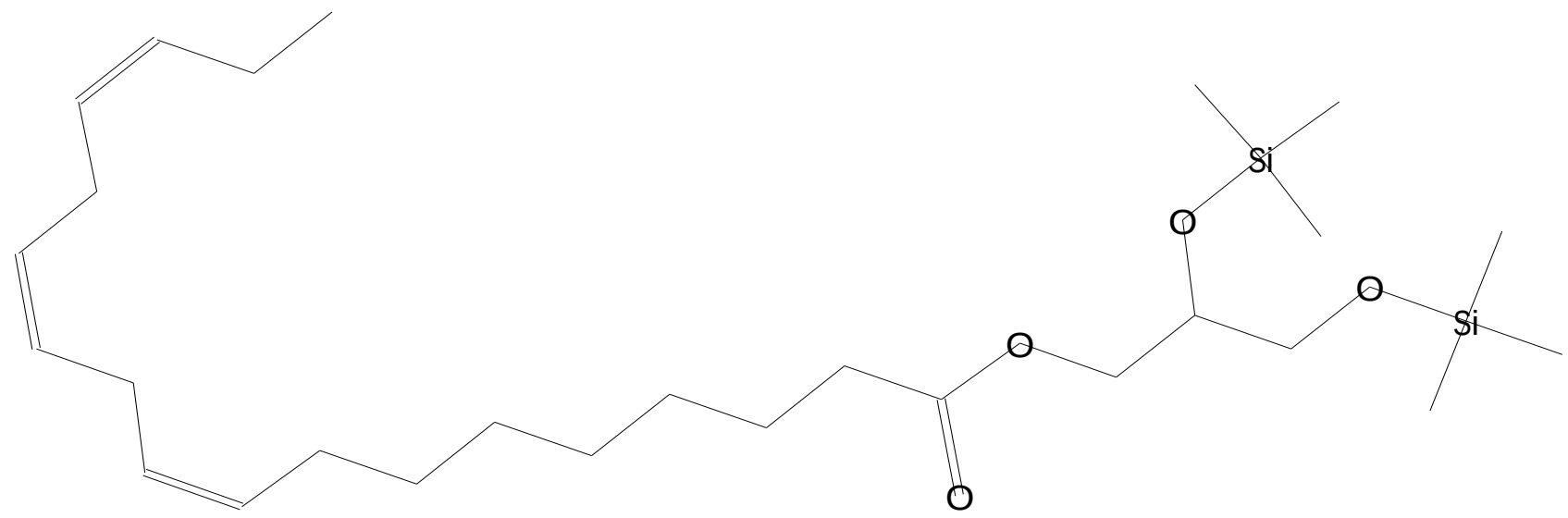

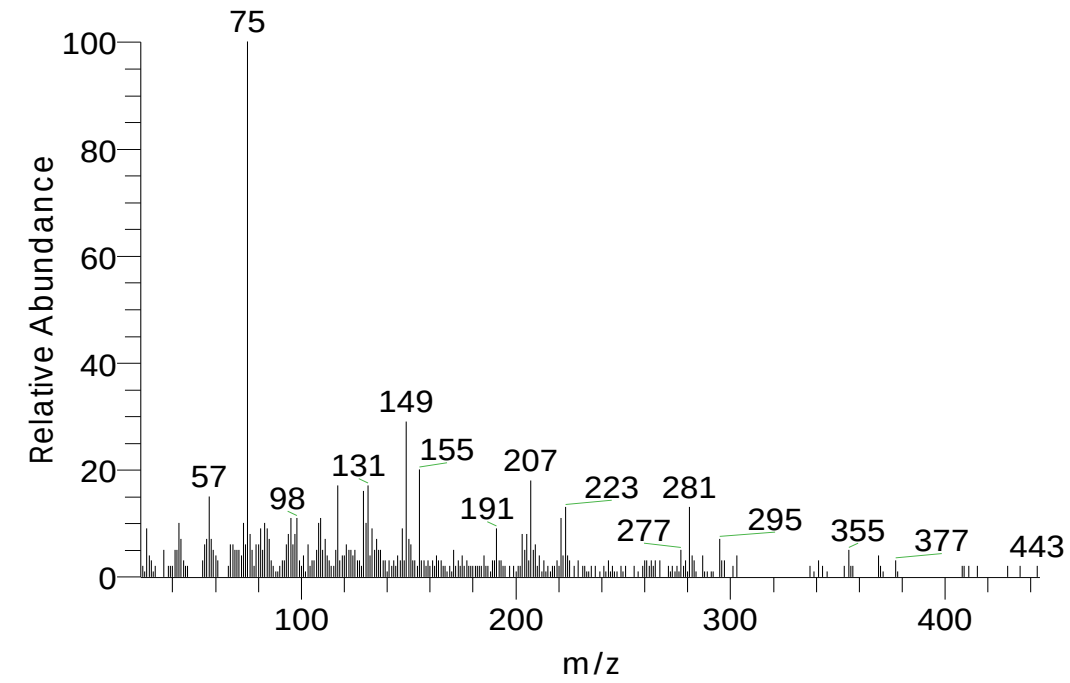

9,12-OCTADECADIENOIC ACID (Z,Z)-, 2,3-BIS[(TRIMETHYLSILYL)OXY]PROPYL ESTER
Formula C27H54O4Si2, MW 498, CAS# 54284-45-6, Entry# 285148
2,3-BIS[(TRIMETHYLSILYL)OXY]PROPYL (9Z,12Z)-9,12-OCTADECADIENOATE #

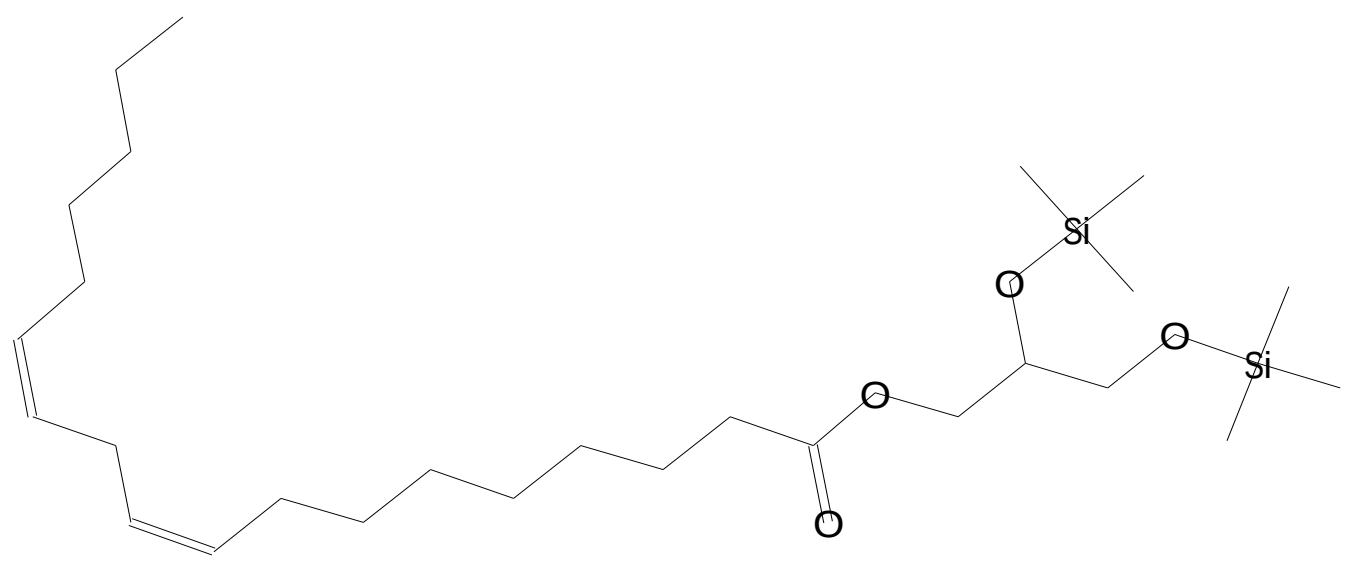

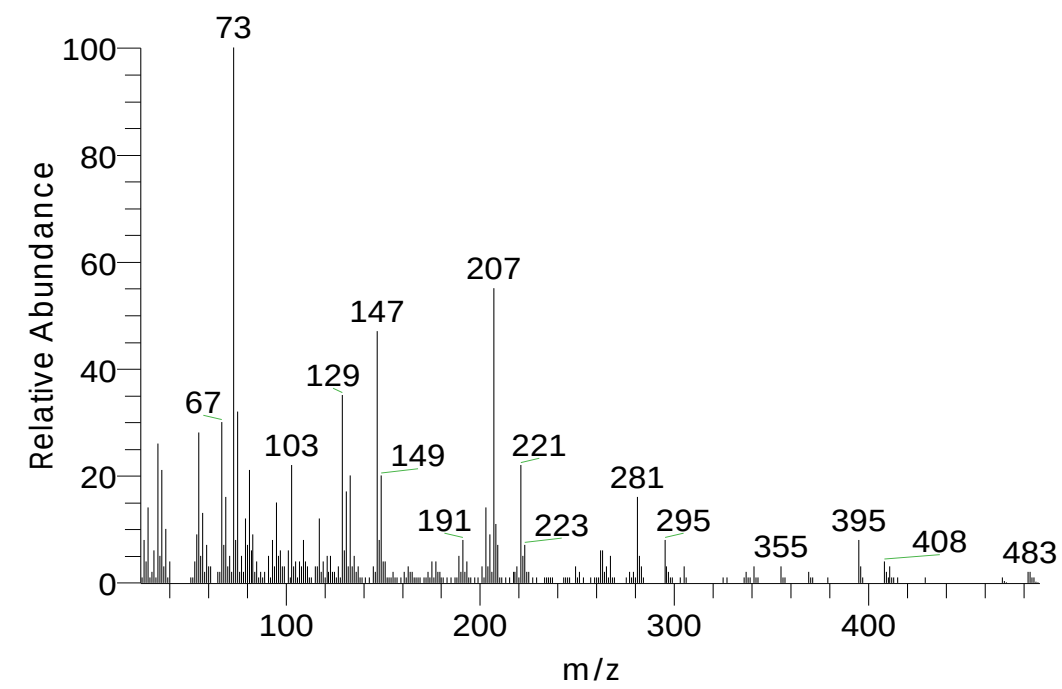

Formula C27H56O4Si2, MW 500, CAS# 54284-48-9, Entry# 285456
2-MONOOLEOYLGLYCEROL TRIMETHYLSILYL ETHER

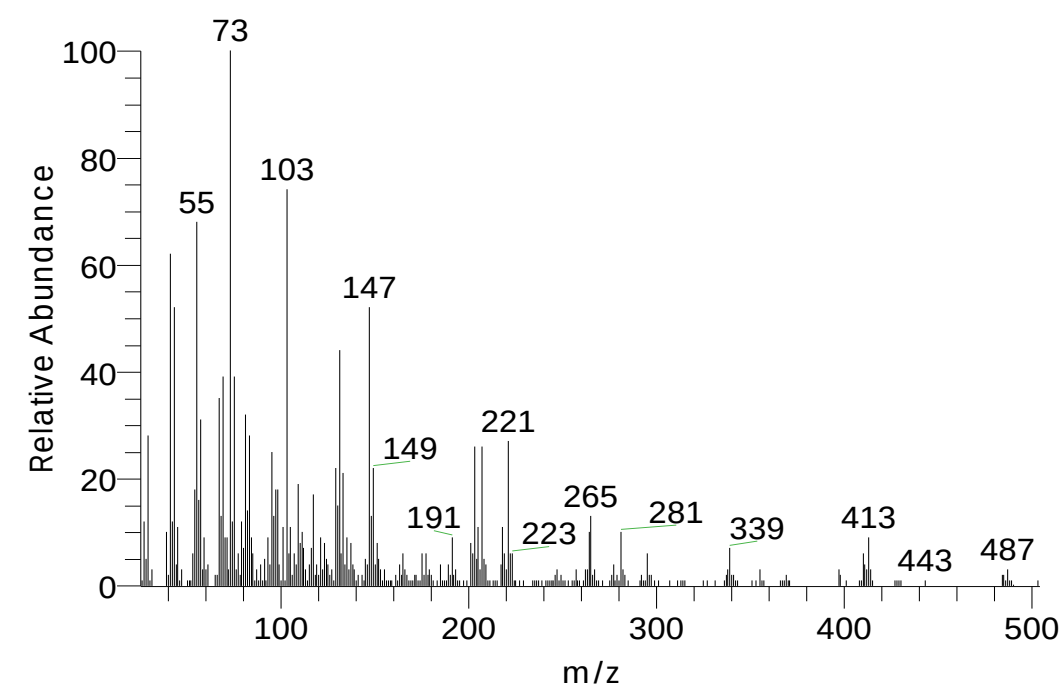

à-D-GALACTOPYRANOSIDE, METHYL 2,3-BIS-O-(TRIMETHYLSILYL)-, CYCLIC BUTYLBORONATE
Formula C17H37BO6Si2, MW 404, CAS# 56211-11-1, Entry# 257663

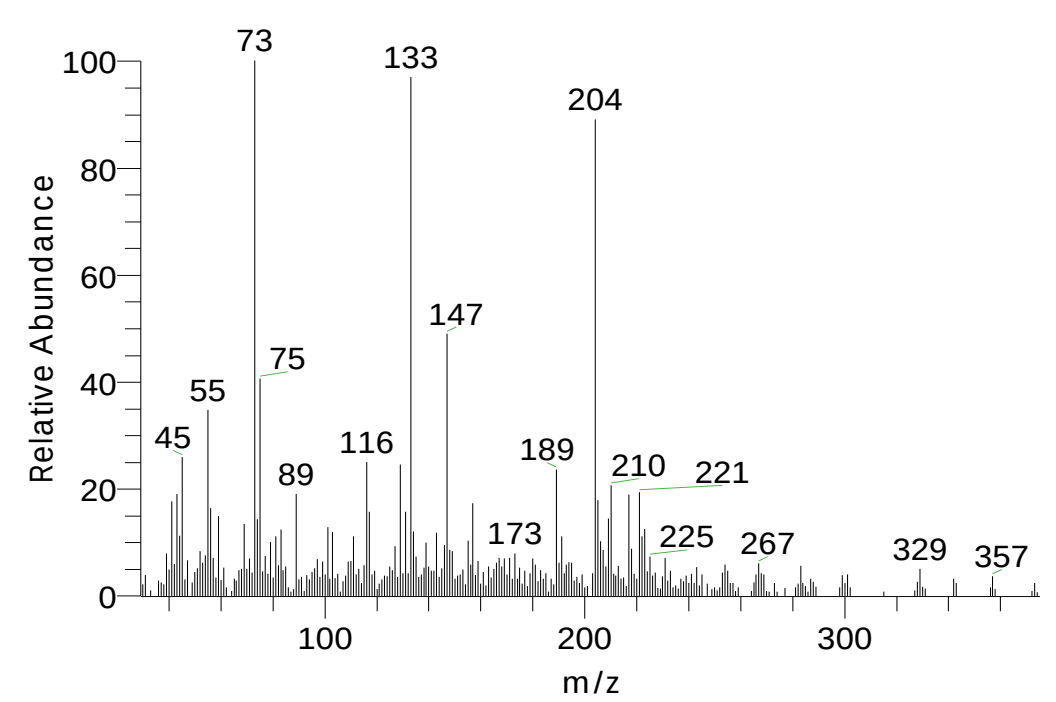

My GC-MS Report

Compound Structure | Hit Spectrum

Loperamide
Formula C29H33ClN2O2, MW 476, CAS# 34552-83-5, Entry# 1181

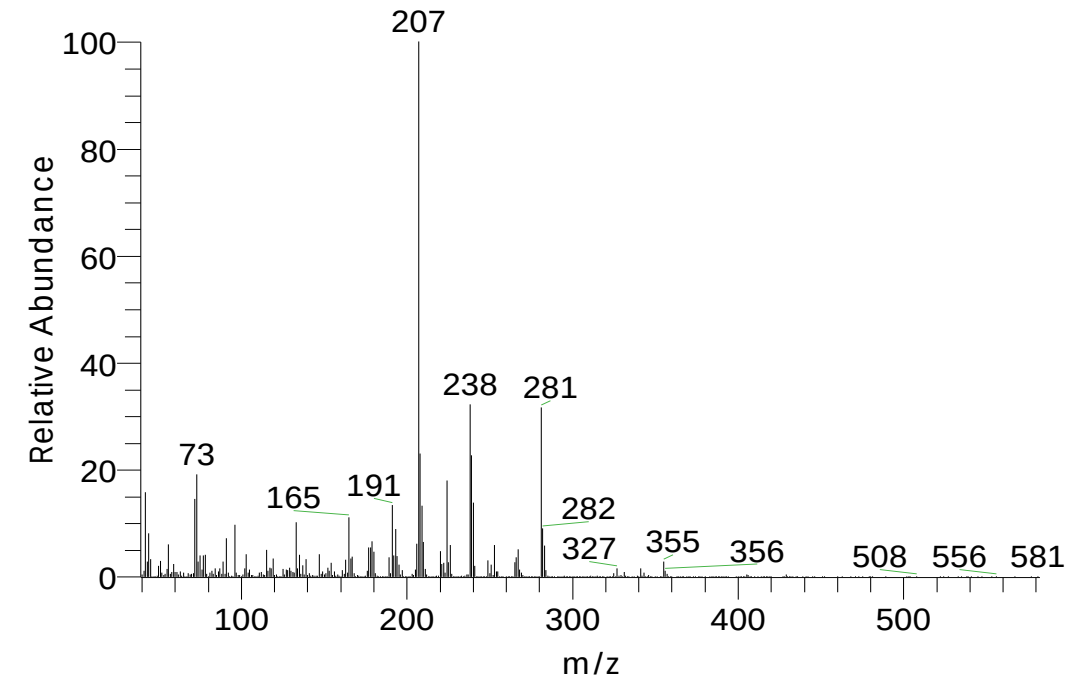

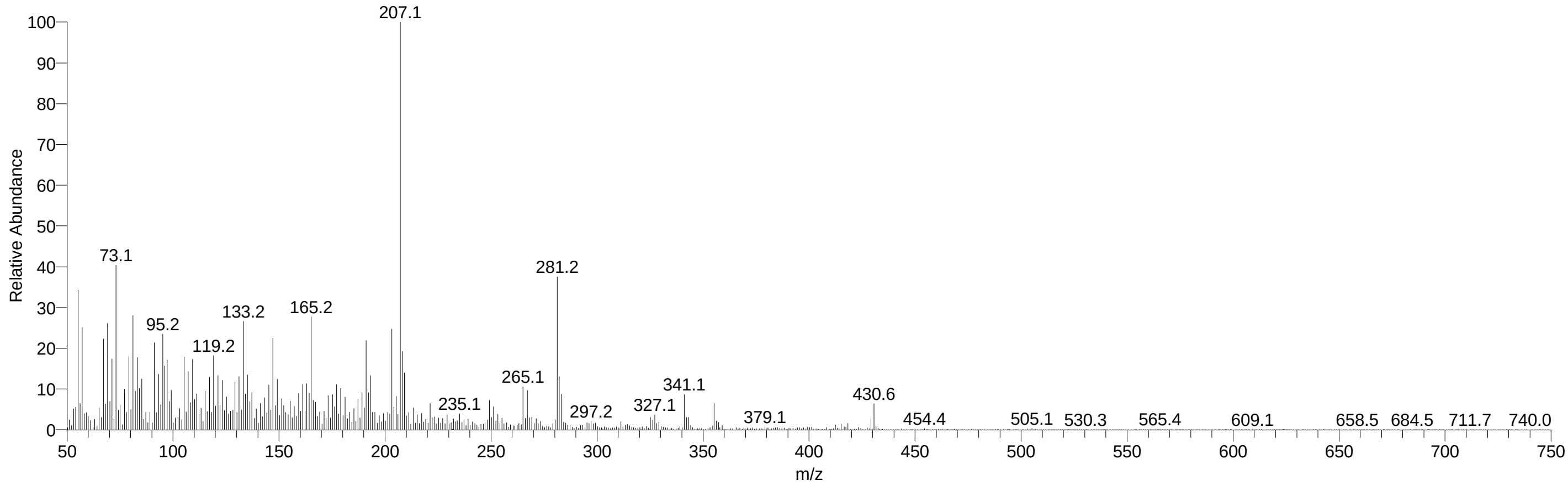

RT	Compound Name	Area %	MF	Molecular Formula	Molecular Weight	Cas #	Library
42.79	9,12,15-OCTADECATRIENOIC ACID, 2,3-BIS[(TRIMETHYLSILYL)OXY]PROPYL ESTER, (Z,Z,Z)-	0.43	783	C27H52O4Si2	496	55521-22-7	WileyRegistry8e
42.79	9,12-OCTADECADIENOIC ACID (Z,Z)-, 2,3-BIS[(TRIMETHYLSILYL)OXY]PROPYL ESTER	0.43	786	C27H54O4Si2	498	54284-45-6	WileyRegistry8e
42.79	.psi.,.psi.-Carotene, 1,1',2,2'-tetrahydro-1,1'-dimethoxy-	0.43	709	C42H64O2	600	13833-01-7	mainlib
42.79	.PSI.,.PSI.-CAROTENE, 1,1',2,2'-TETRAHYDRO-1,1'-DIMETHOXY-	0.43	708	C42H64O2	600	13833-01-7	WileyRegistry8e
42.79	9-OCTADECENOIC ACID (Z)-, 2-[(TRIMETHYLSILYL)OXY]-1-[[(TRIMETHYLSILYL)OXY]METHYL]ETHYL ESTER	0.43	742	C27H56O4Si2	500	54284-48-9	WileyRegistry8e

Compound Structure | Hit Spectrum

9,12,15-OCTADECATRIENOIC ACID, 2,3-BIS[(TRIMETHYLSILYL)OXY]PROPYL ESTER, (Z,Z,Z)-
Formula C27H52O4Si2, MW 496, CAS# 55521-22-7, Entry# 284834
2,3-BIS[(TRIMETHYLSILYL)OXY]PROPYL (9E,12E,15E)-9,12,15-OCTADECATRIENOATE #

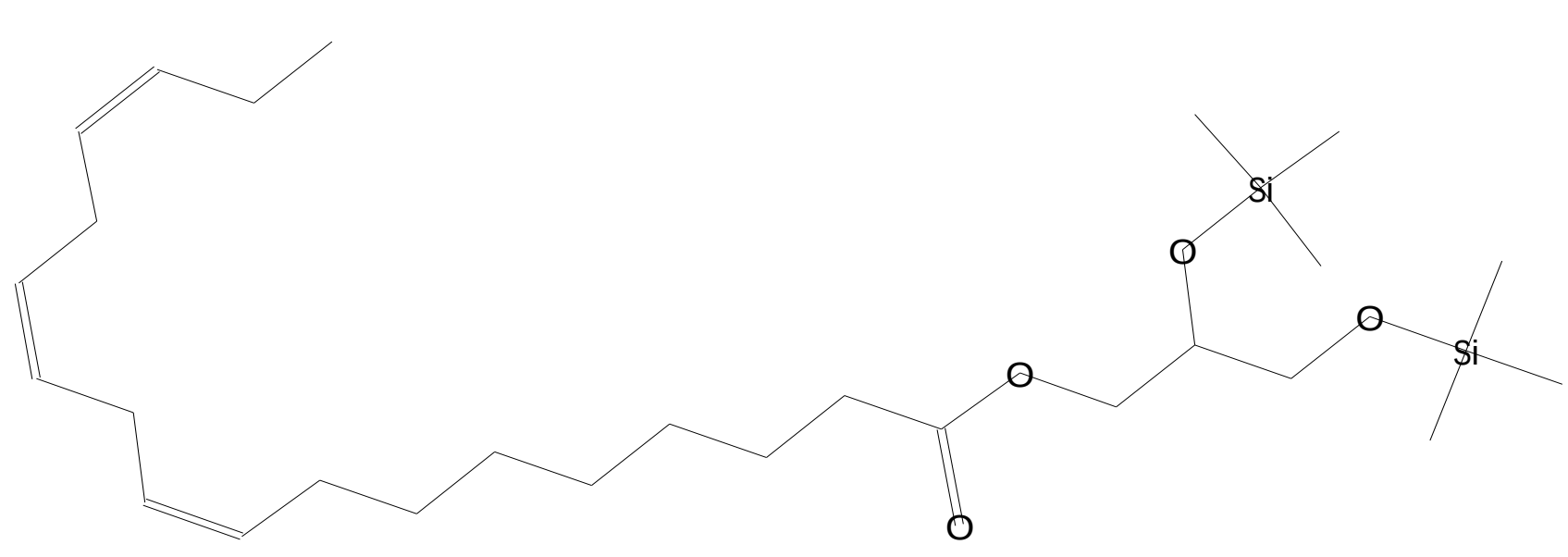

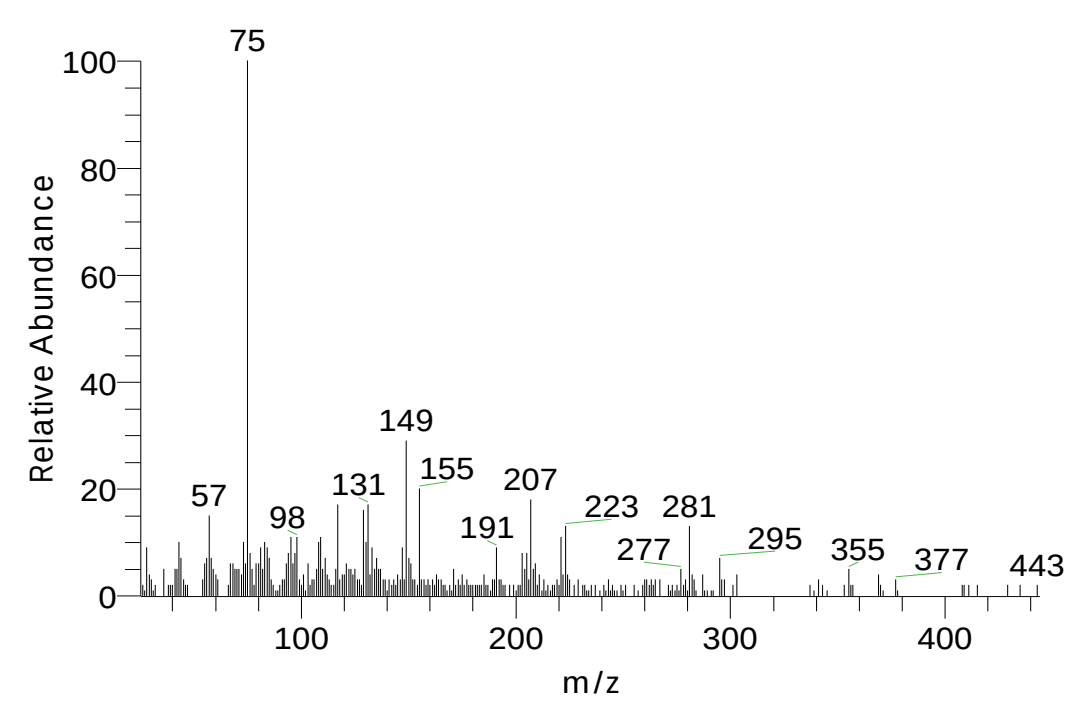

My GC-MS Report

Compound Structure | Hit Spectrum

9,12-OCTADECADIENOIC ACID (Z,Z)-, 2,3-BIS[(TRIMETHYLSILYL)OXY]PROPYL ESTER
Formula C27H54O4Si2, MW 498, CAS# 54284-45-6, Entry# 285148
2,3-BIS[(TRIMETHYLSILYL)OXY]PROPYL (9Z,12Z)-9,12-OCTADECADIENOATE #

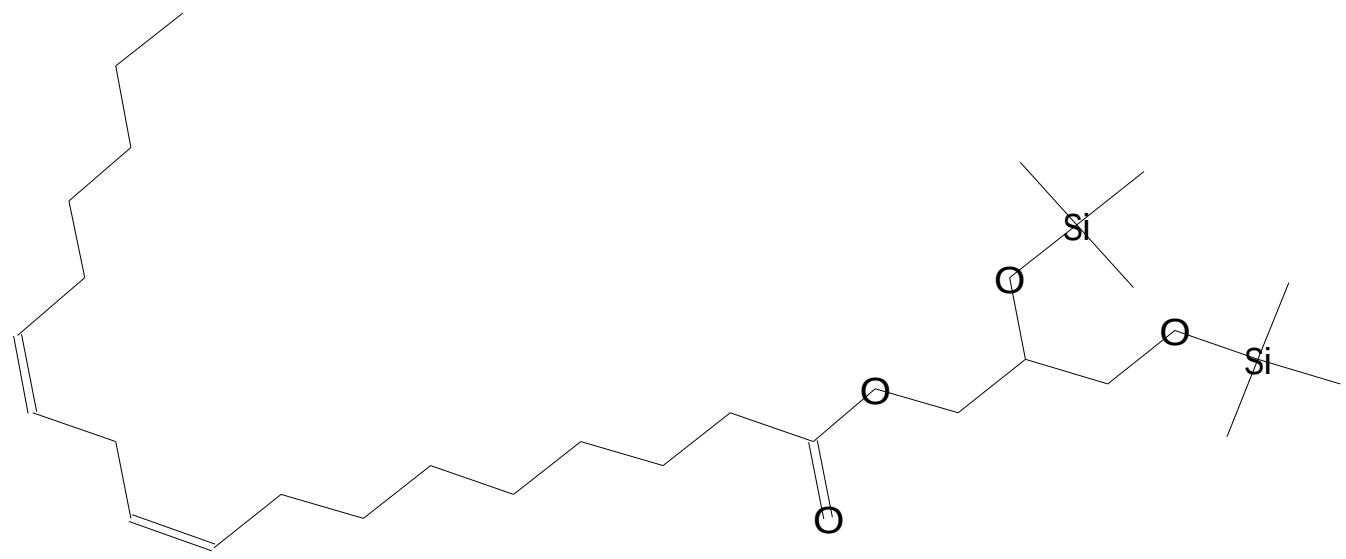

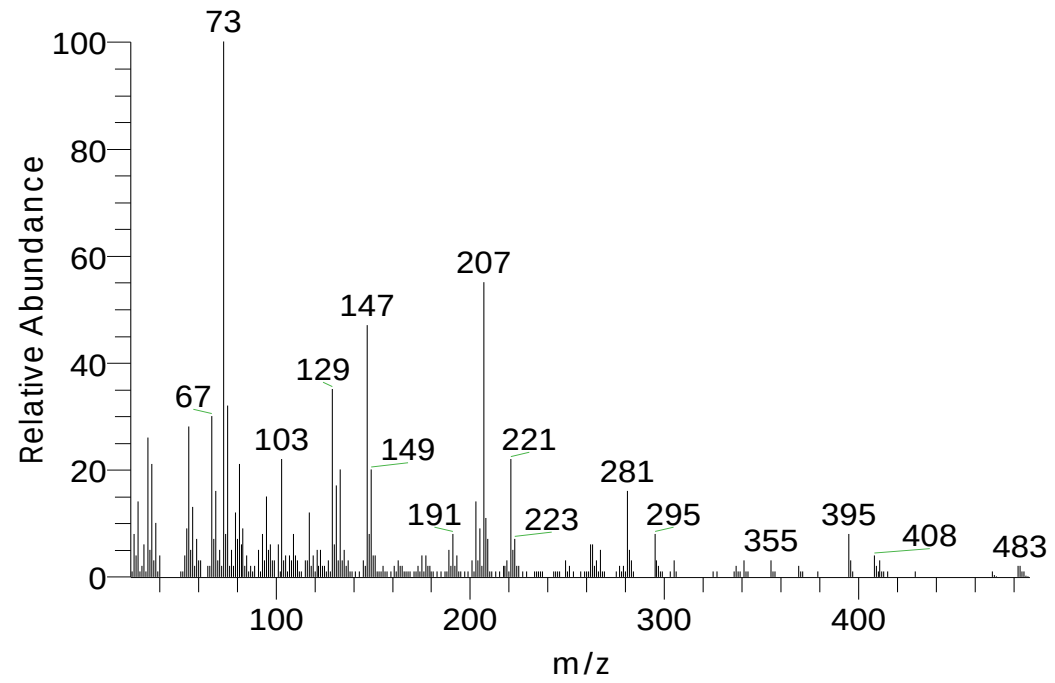

.psi.,.psi.-Carotene, 1,1',2,2'-tetrahydro-1,1'-dimethoxy-
Formula C42H64O2, MW 600, CAS# 13833-01-7, Entry# 41205
Lycopene, 1,1',2,2'-tetrahydro-1,1'-dimethoxy-, all-trans-

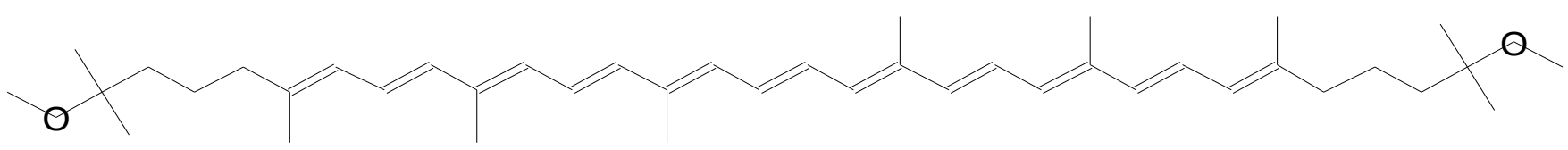

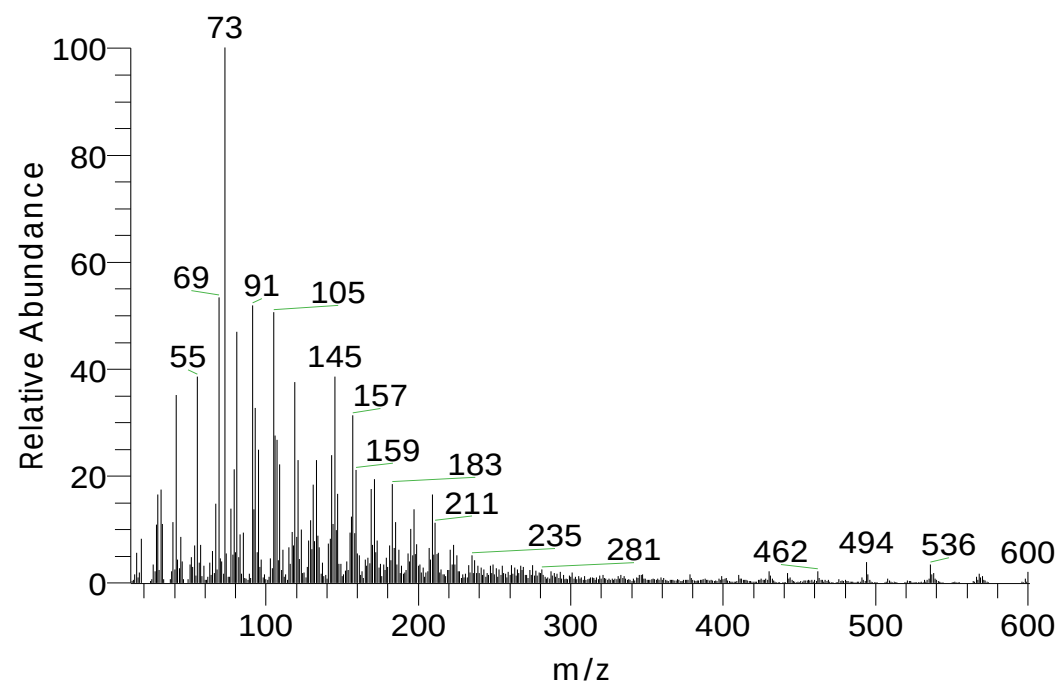

.PSI.,.PSI.-CAROTENE, 1,1',2,2'-TETRAHYDRO-1,1'-DIMETHOXY-
Formula C42H64O2, MW 600, CAS# 13833-01-7, Entry# 296796
1,1',2,2'-TETRAHYDRO-PSI,PSI-CAROTENE #

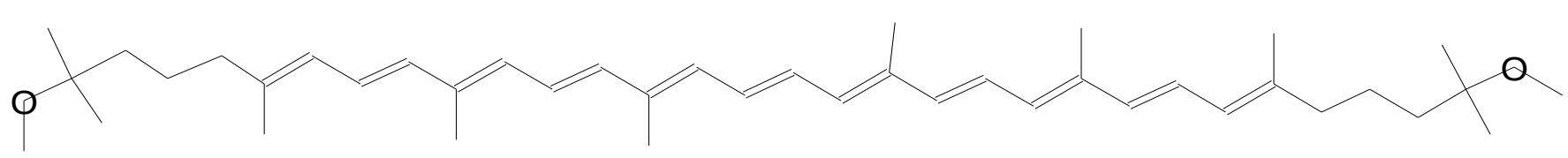

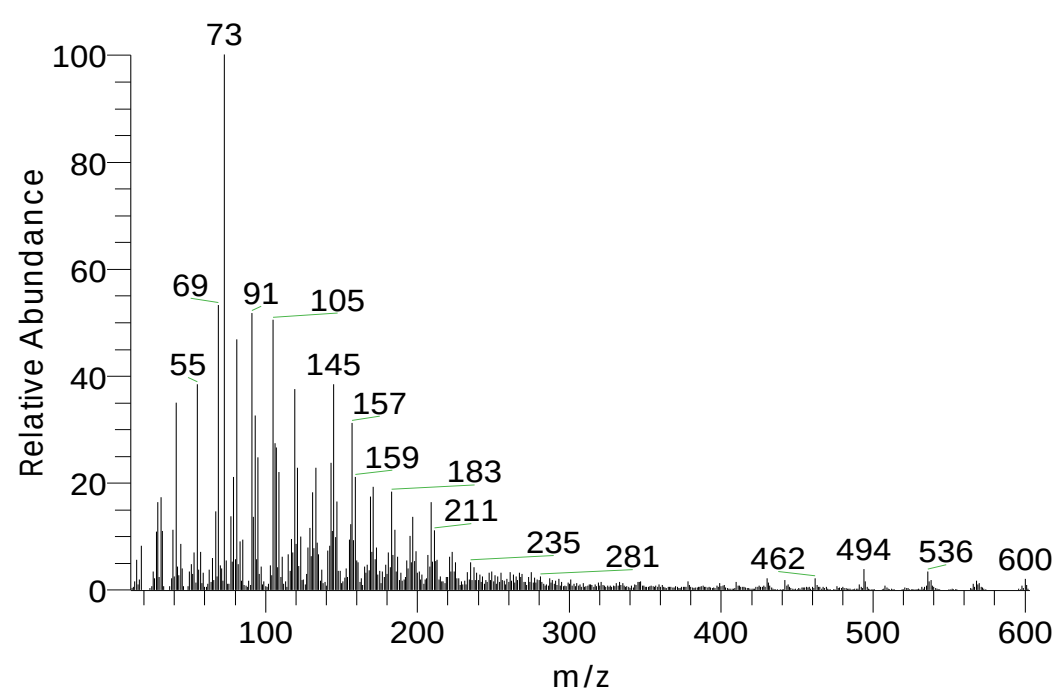

Formula C27H56O4Si2, MW 500, CAS# 54284-48-9, Entry# 285456
2-MONOOLEOYLGLYCEROL TRIMETHYLSILYL ETHER

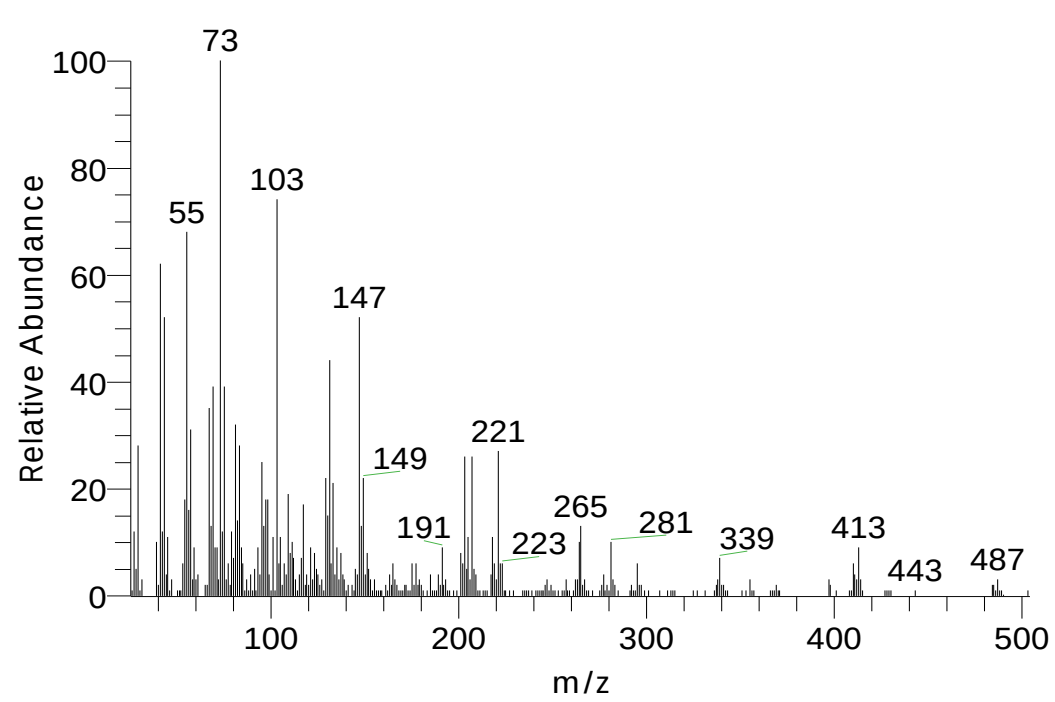

My GC-MS Report

Oil_DrHafsa #11650 RT: 43.07 AV: 1 NL: 1.87E6
T: + c EI Full ms [50.000-750.000]

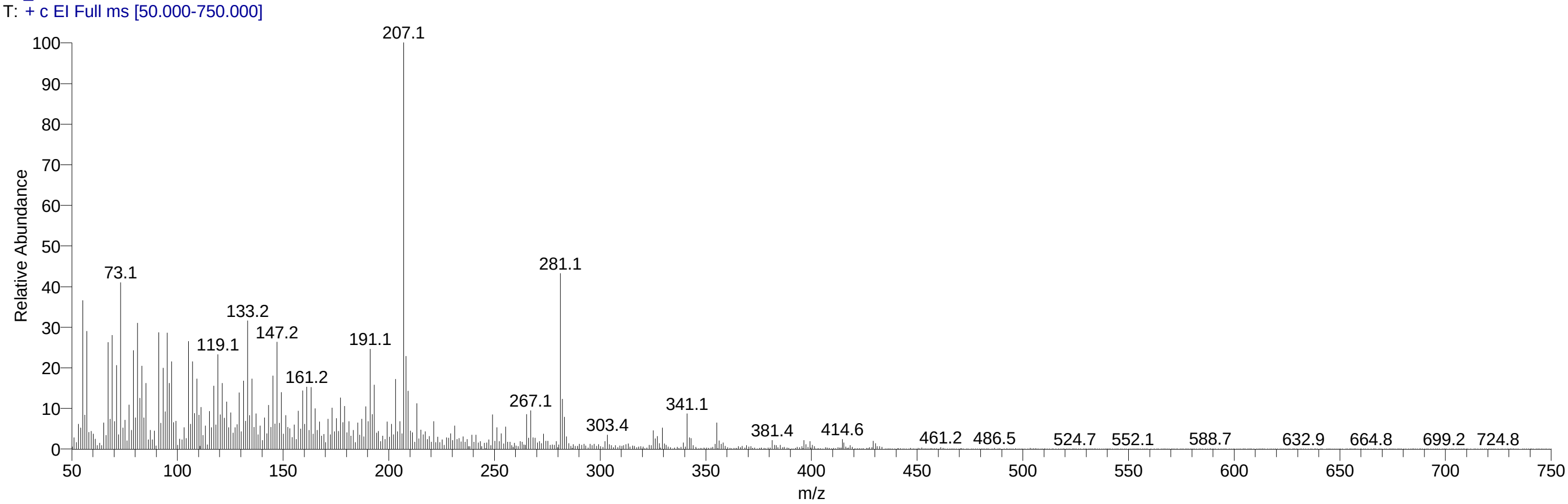

RT	Compound Name	Area %	MF	Molecular Formula	Molecular Weight	Cas #	Library
43.07	9,12,15-OCTADECATRIENOIC ACID, 2,3-BIS[(TRIMETHYLSILYL)OXY]PROPYL ESTER, (Z,Z,Z)-	1.53	782	C27H52O4Si2	496	55521-22-7	WileyRegistry8e
43.07	9,12-OCTADECADIENOIC ACID (Z,Z)-, 2,3-BIS[(TRIMETHYLSILYL)OXY]PROPYL ESTER	1.53	790	C27H54O4Si2	498	54284-45-6	WileyRegistry8e
43.07	.psi.,.psi.-Carotene, 1,1',2,2'-tetrahydro-1,1'-dimethoxy-	1.53	721	C42H64O2	600	13833-01-7	mainlib
43.07	.PSI.,.PSI.-CAROTENE, 1,1',2,2'-TETRAHYDRO-1,1'-DIMETHOXY-	1.53	720	C42H64O2	600	13833-01-7	WileyRegistry8e
43.07	9-OCTADECENOIC ACID (Z)-, 2-[(TRIMETHYLSILYL)OXY]-1-[[(TRIMETHYLSILYL)OXY]METHYL]ETHYL ESTER	1.53	743	C27H56O4Si2	500	54284-48-9	WileyRegistry8e

Compound Structure | Hit Spectrum

9,12,15-OCTADECATRIENOIC ACID, 2,3-BIS[(TRIMETHYLSILYL)OXY]PROPYL ESTER, (Z,Z,Z)-
Formula C27H52O4Si2, MW 496, CAS# 55521-22-7, Entry# 284834
2,3-BIS[(TRIMETHYLSILYL)OXY]PROPYL (9E,12E,15E)-9,12,15-OCTADECATRIENOATE #

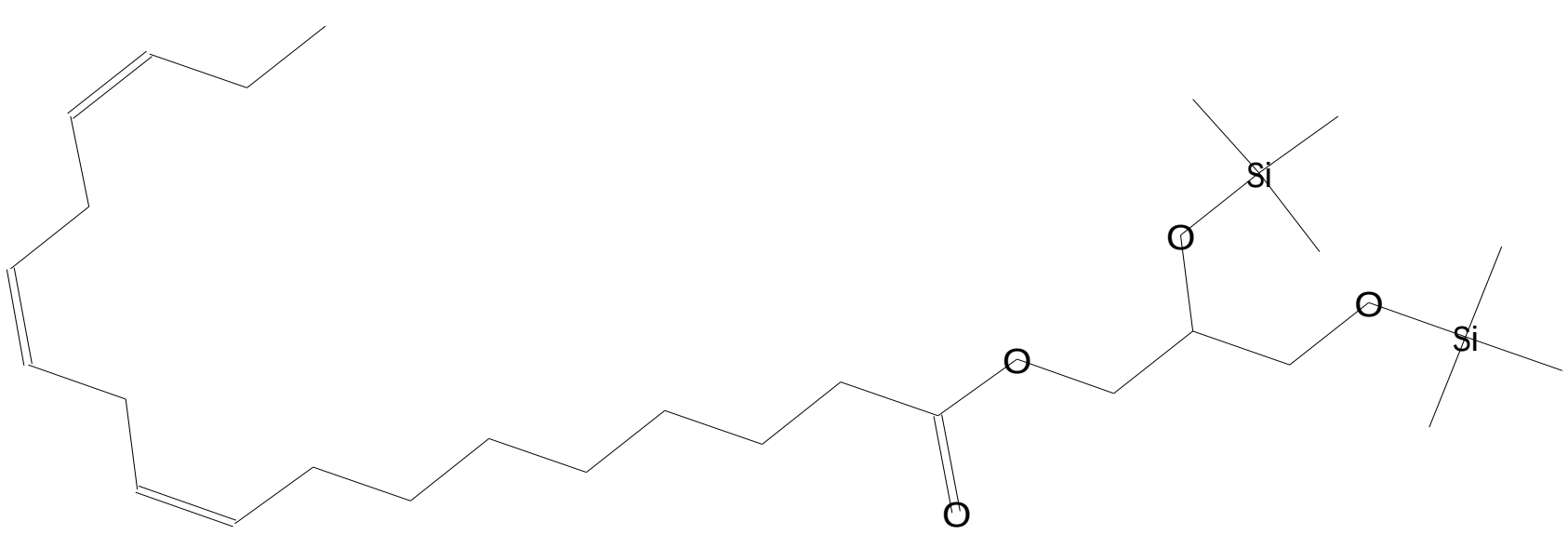

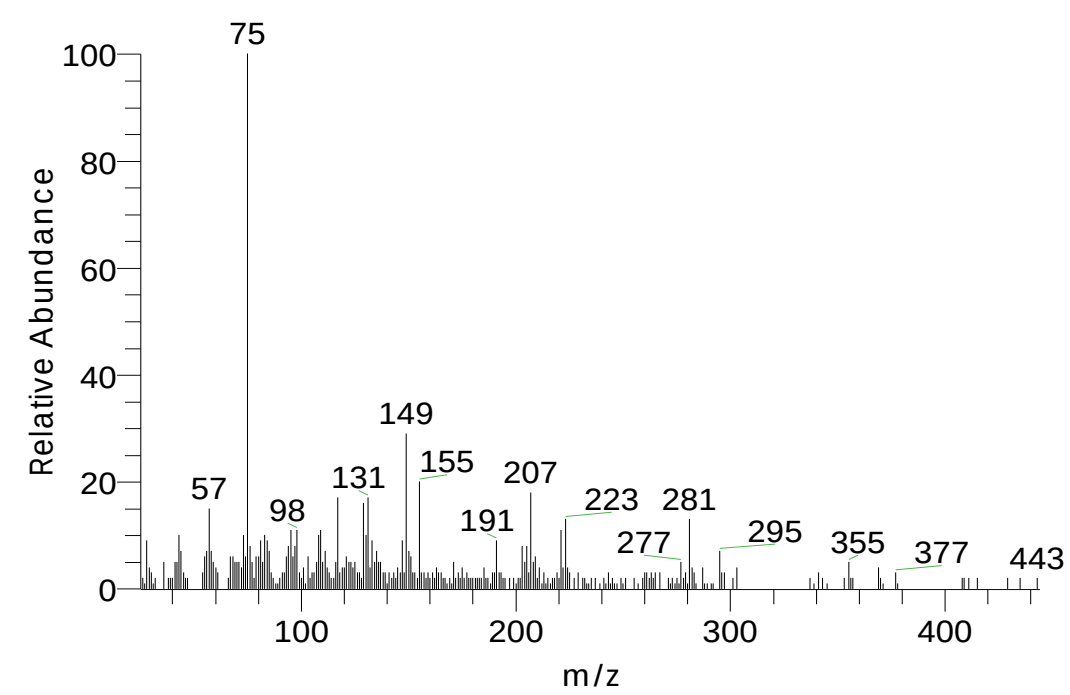

9,12-OCTADECADIENOIC ACID (Z,Z)-, 2,3-BIS[(TRIMETHYLSILYL)OXY]PROPYL ESTER
Formula C27H54O4Si2, MW 498, CAS# 54284-45-6, Entry# 285148
2,3-BIS[(TRIMETHYLSILYL)OXY]PROPYL (9Z,12Z)-9,12-OCTADECADIENOATE #

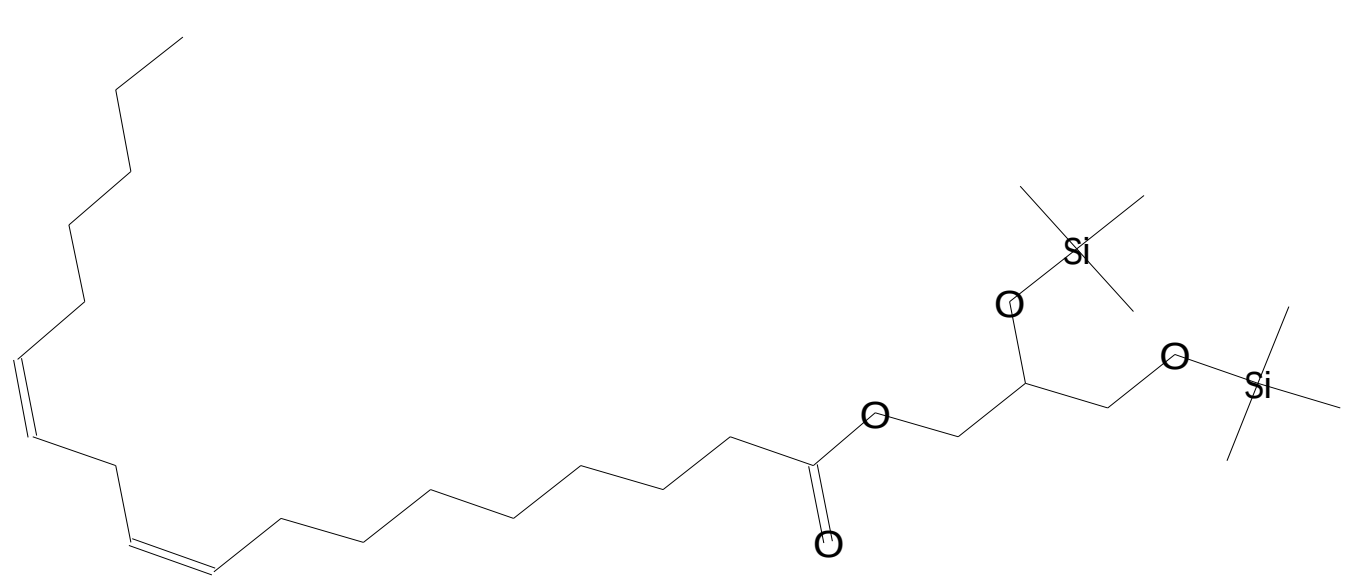

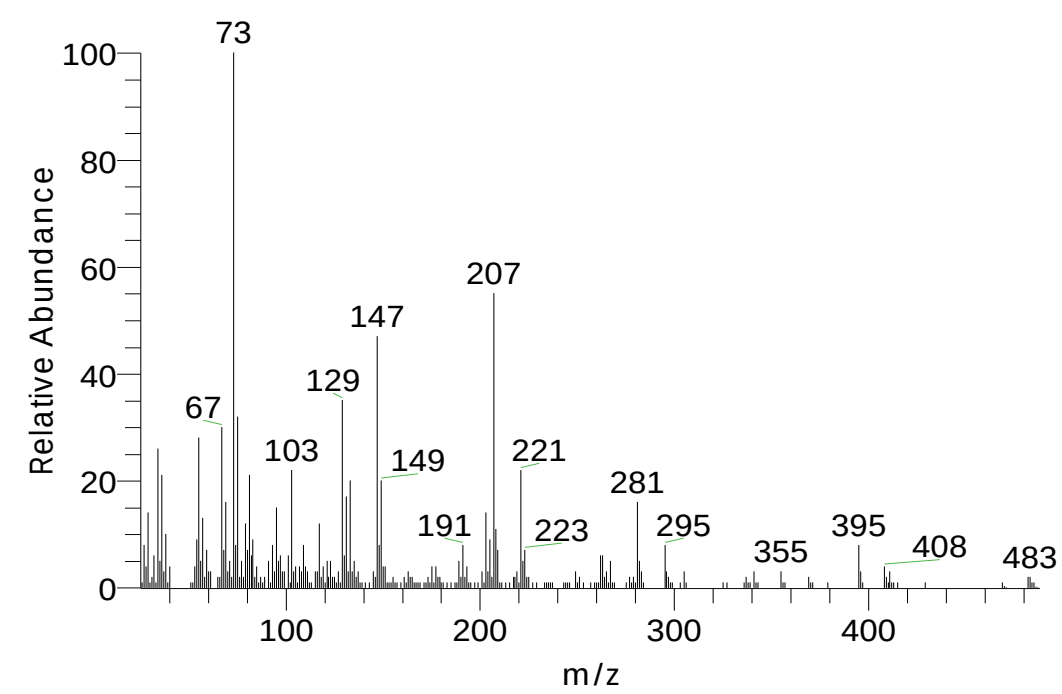

My GC-MS Report

Compound Structure | Hit Spectrum

.psi.,.psi.-Carotene, 1,1',2,2'-tetrahydro-1,1'-dimethoxy-
Formula C42H64O2, MW 600, CAS# 13833-01-7, Entry# 41205
Lycopene, 1,1',2,2'-tetrahydro-1,1'-dimethoxy-, all-trans-

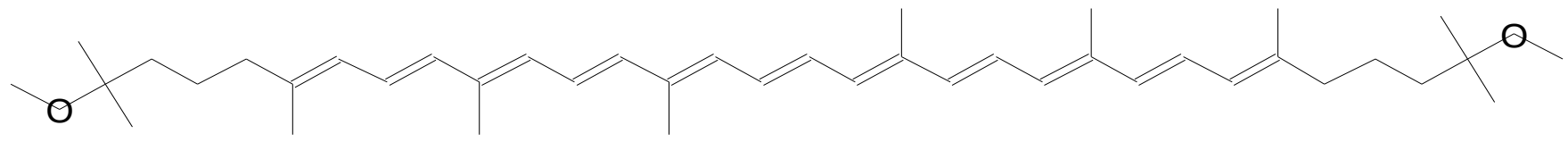

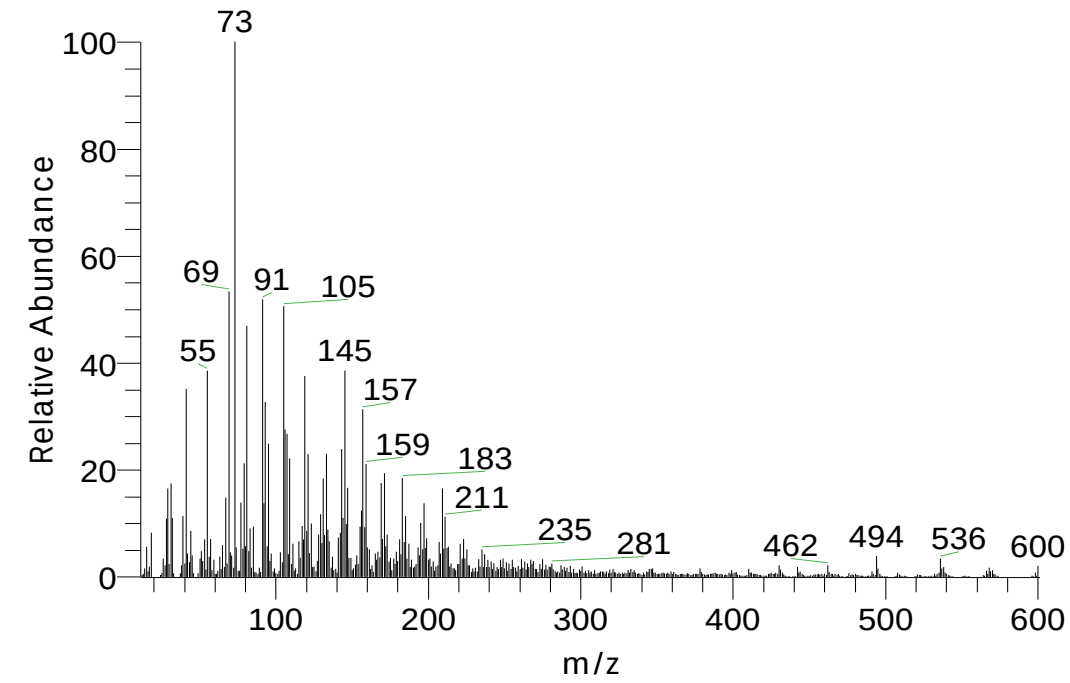

.PSI.,.PSI.-CAROTENE, 1,1',2,2'-TETRAHYDRO-1,1'-DIMETHOXY-
Formula C42H64O2, MW 600, CAS# 13833-01-7, Entry# 296796
1,1',2,2'-TETRAHYDRO-PSI,PSI-CAROTENE #

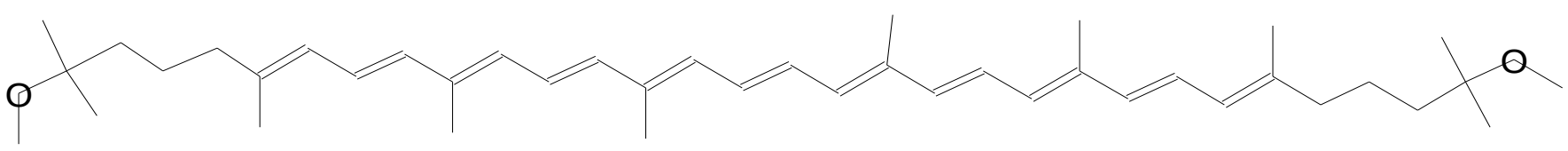

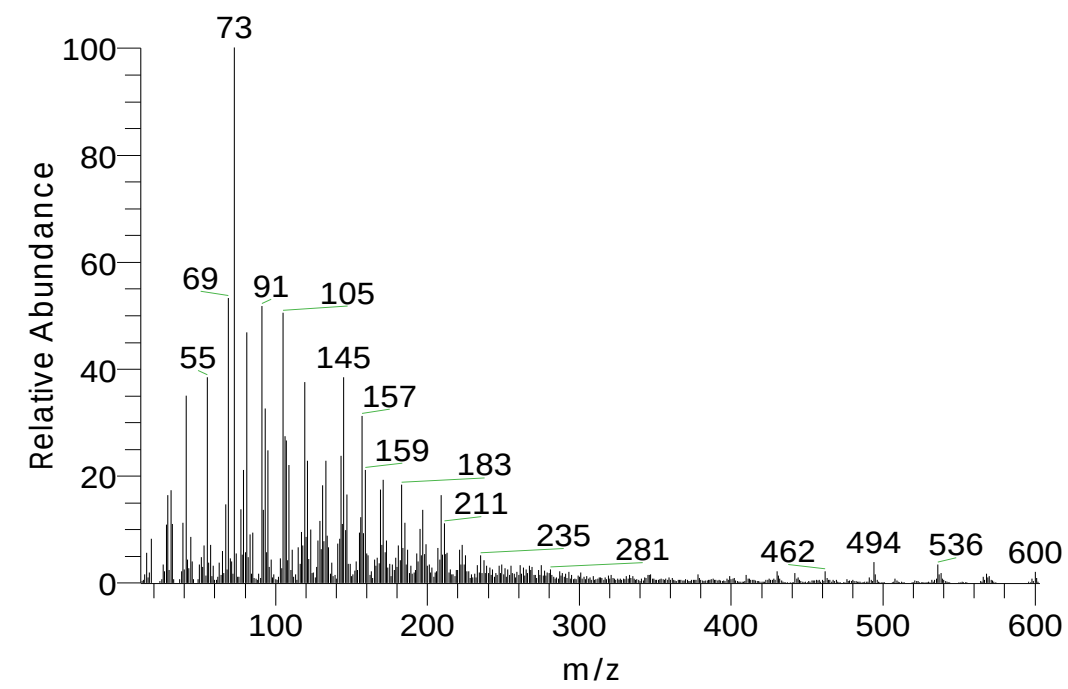

Formula C27H56O4Si2, MW 500, CAS# 54284-48-9, Entry# 285456
2-MONOOLEOYLGLYCEROL TRIMETHYLSILYL ETHER

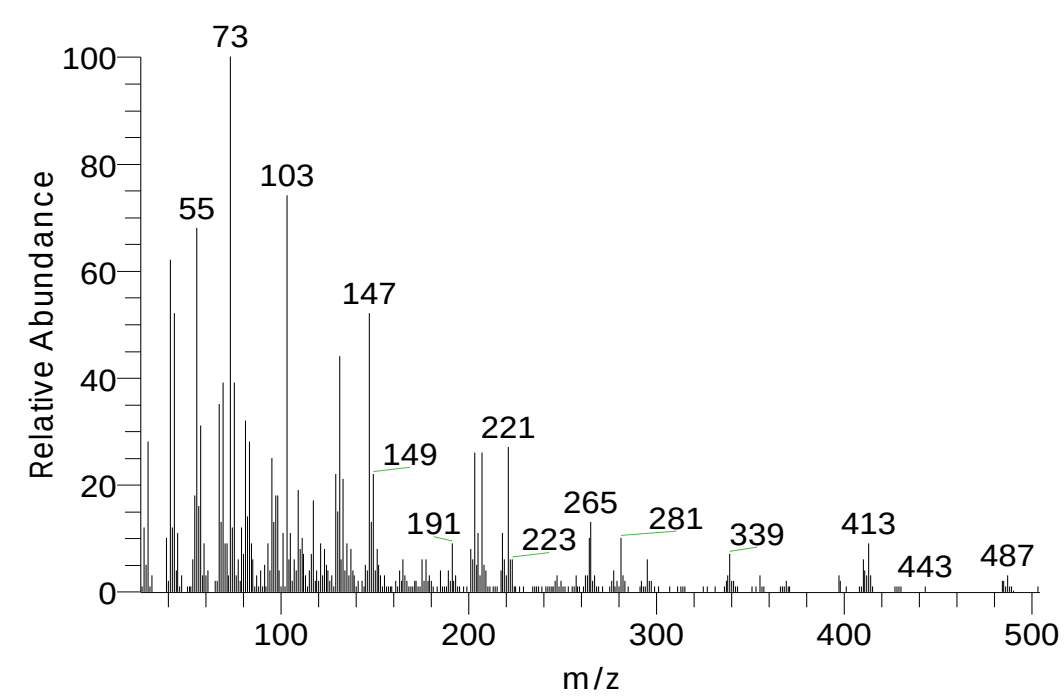

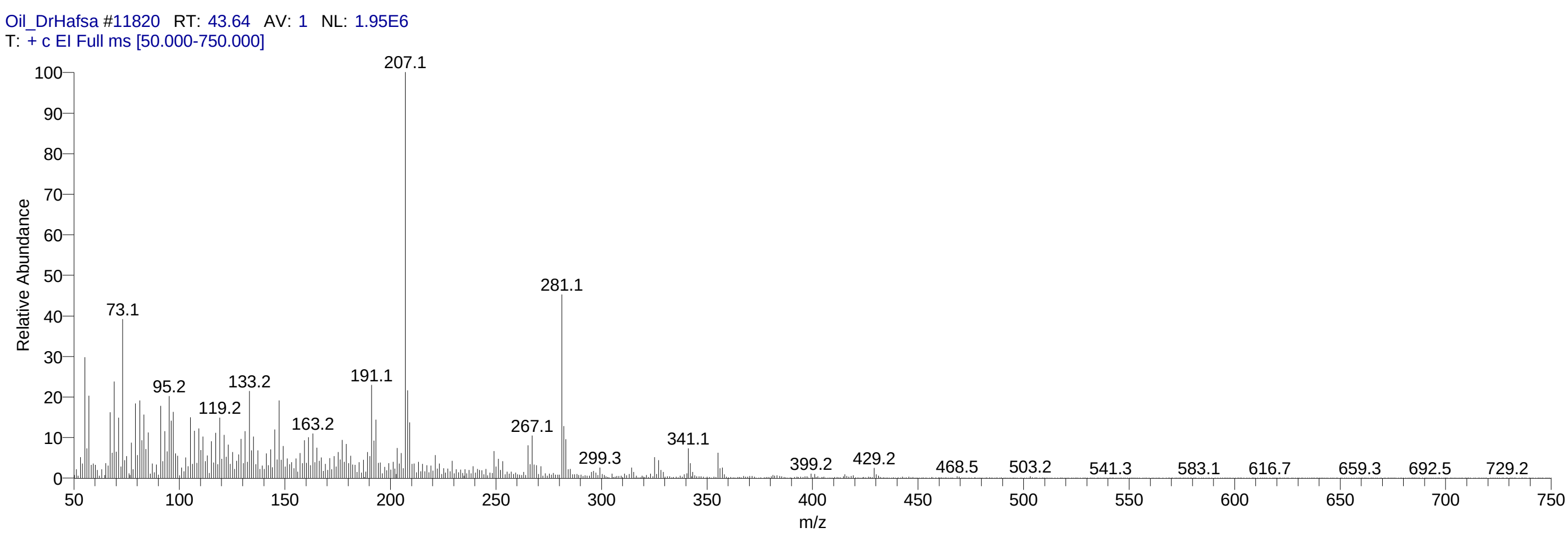

RT	Compound Name	Area %	MF	Molecular Formula	Molecular Weight	Cas #	Library
43.64	4H-1-BENZOPYRAN-4-ONE, 2-(3,4-DIMETHOXYPHENYL)-3,5-DIHYDROXY-7-METHOXY-	0.33	750	C18H16O7	344	6068-80-0	WileyRegistry8e

My GC-MS Report

RT	Compound Name	Area %	MF	Molecular Formula	Molecular Weight	Cas #	Library
43.64	9,12-OCTADECADIENOIC ACID (Z,Z)-, 2,3-BIS[(TRIMETHYLSILYL)OXY]PROPYL ESTER	0.33	769	C27H54O4Si2	498	54284-45-6	WileyRegistry8e
43.64	.psi.,.psi.-Carotene, 1,1',2,2'-tetrahydro-1,1'-dimethoxy-	0.33	698	C42H64O2	600	13833-01-7	mainlib
43.64	.PSI.,.PSI.-CAROTENE, 1,1',2,2'-TETRAHYDRO-1,1'-DIMETHOXY-	0.33	697	C42H64O2	600	13833-01-7	WileyRegistry8e
43.64	Loperamide	0.33	674	C29H33ClN2O2	476	34552-83-5	CaymanSpectralLibrary-NIST.HP

Compound Structure | Hit Spectrum

4H-1-BENZOPYRAN-4-ONE, 2-(3,4-DIMETHOXYPHENYL)-3,5-DIHYDROXY-7-METHOXY-
Formula C18H16O7, MW 344, CAS# 6068-80-0, Entry# 224392
3',4',7-TRIMETHYLQUERCETIN

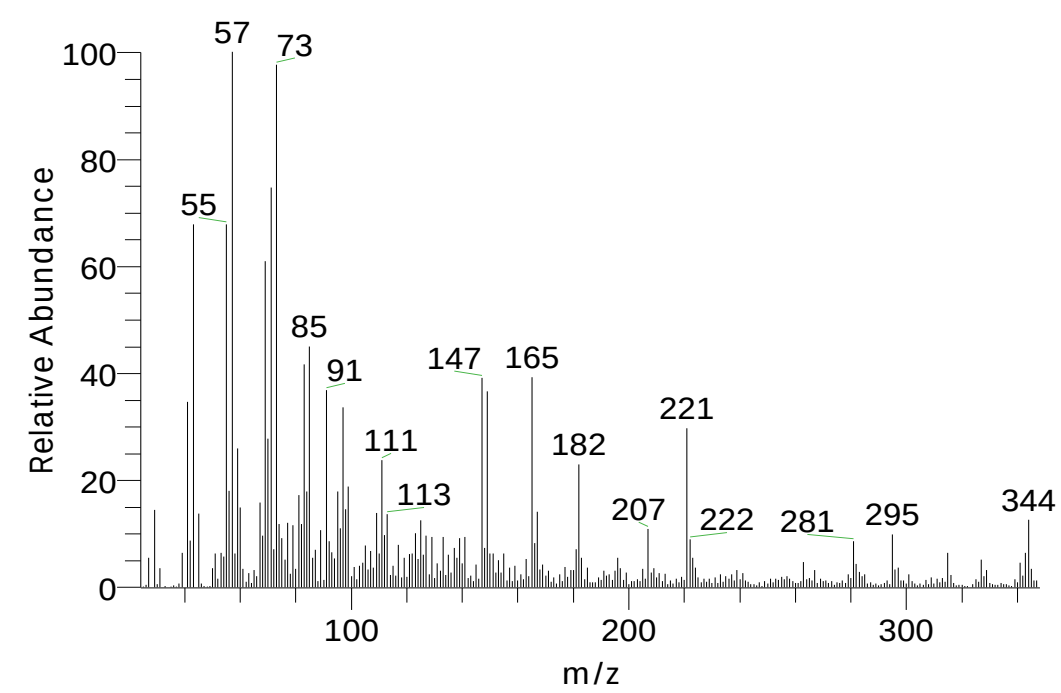

9,12-OCTADECADIENOIC ACID (Z,Z)-, 2,3-BIS[(TRIMETHYLSILYL)OXY]PROPYL ESTER
Formula C27H54O4Si2, MW 498, CAS# 54284-45-6, Entry# 285148
2,3-BIS[(TRIMETHYLSILYL)OXY]PROPYL (9Z,12Z)-9,12-OCTADECADIENOATE #

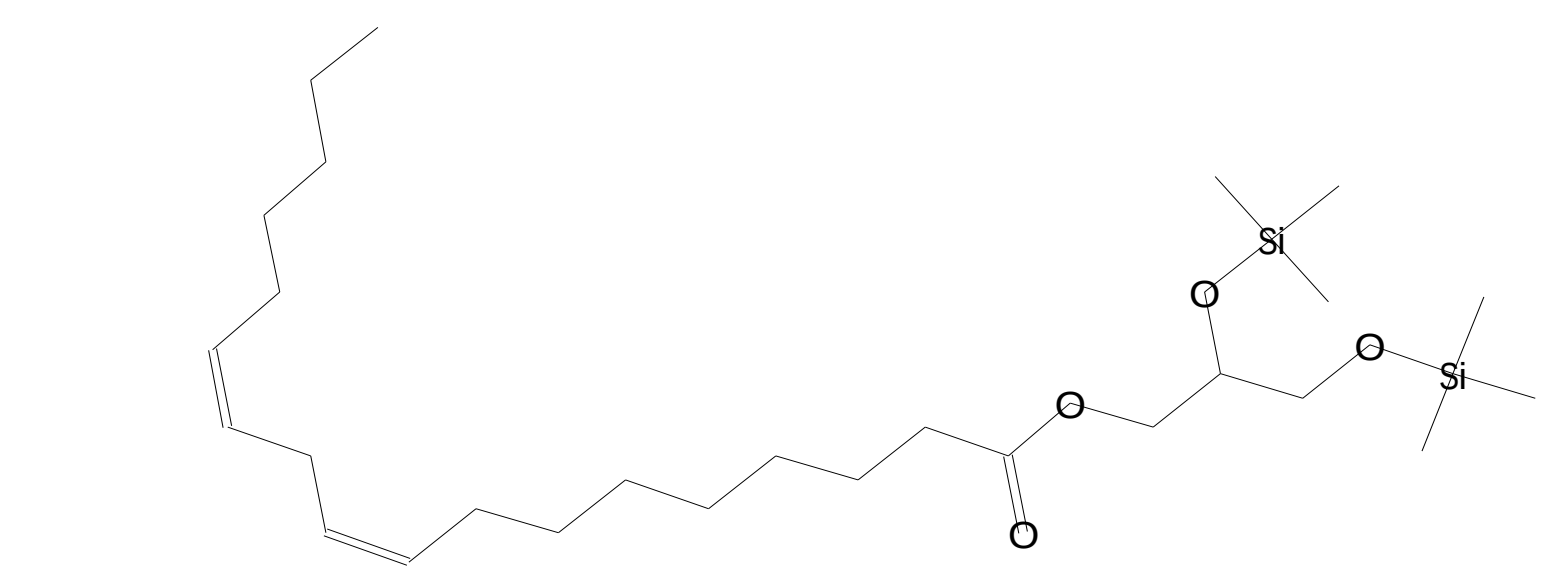

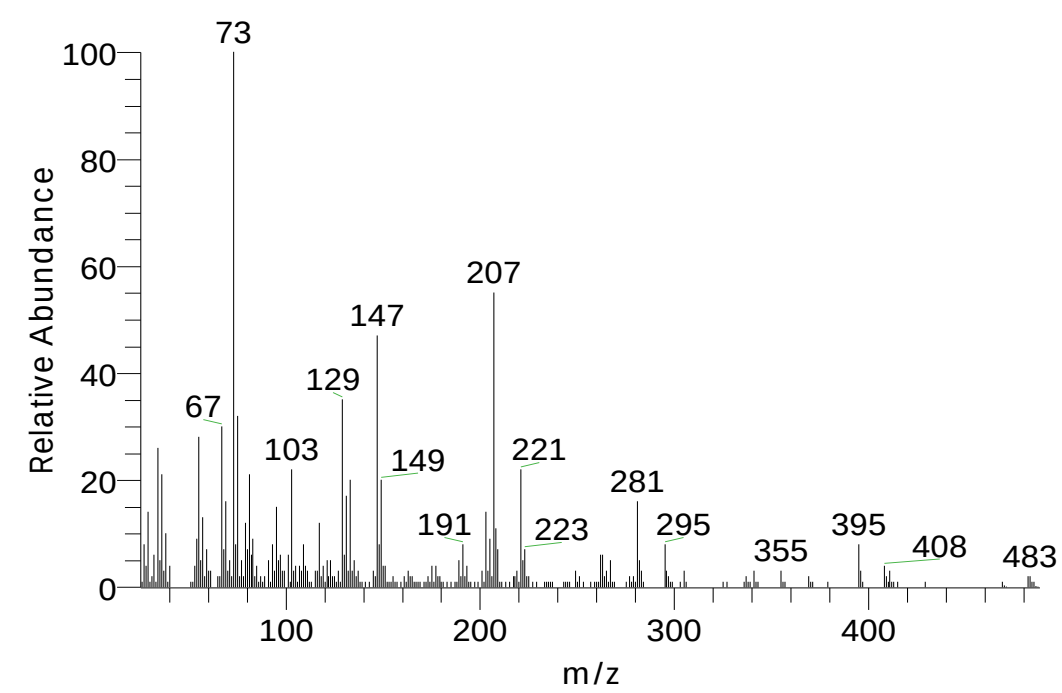

.psi.,.psi.-Carotene, 1,1',2,2'-tetrahydro-1,1'-dimethoxy-
Formula C42H64O2, MW 600, CAS# 13833-01-7, Entry# 41205
Lycopene, 1,1',2,2'-tetrahydro-1,1'-dimethoxy-, all-trans-

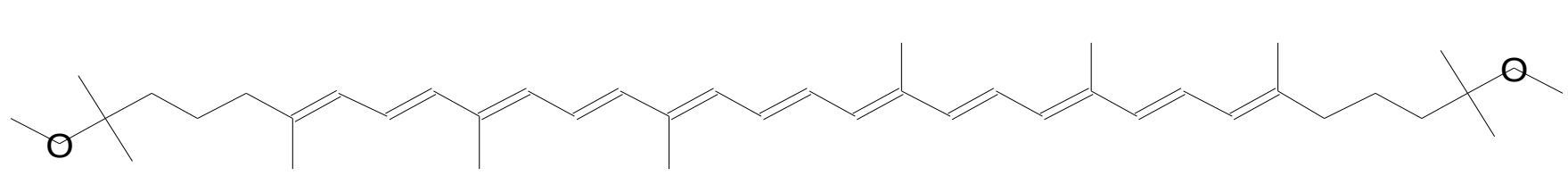

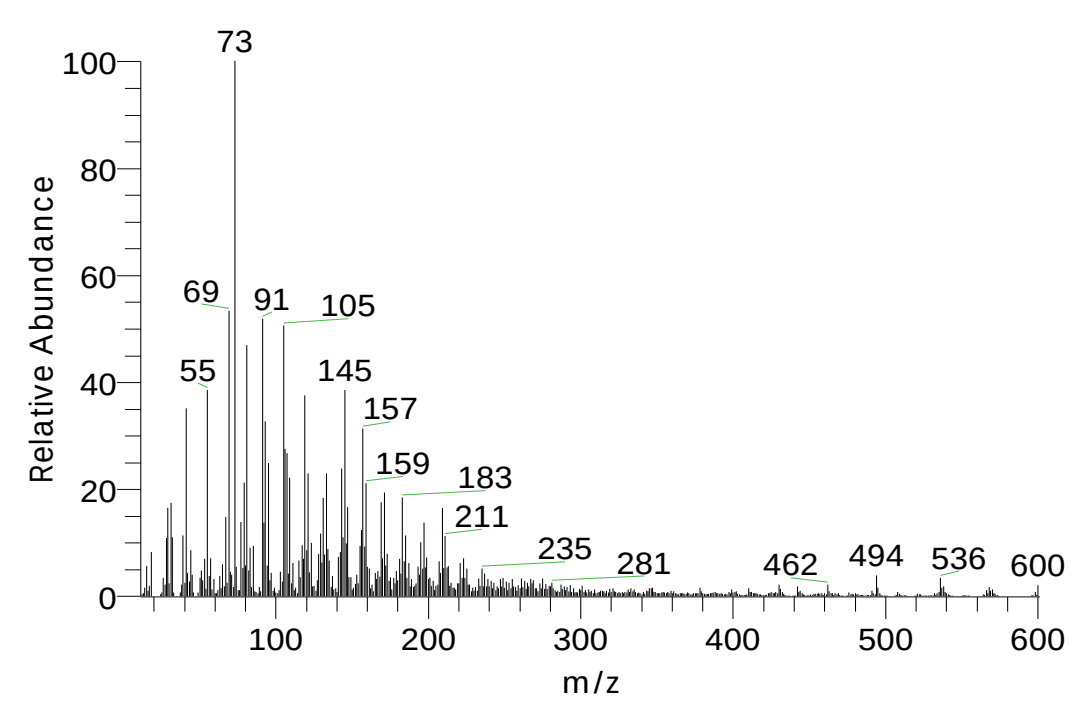

My GC-MS Report

Compound Structure | Hit Spectrum

.PSI.,.PSI.-CAROTENE, 1,1',2,2'-TETRAHYDRO-1,1'-DIMETHOXY-
Formula C42H64O2, MW 600, CAS# 13833-01-7, Entry# 296796
1,1',2,2'-TETRAHYDRO-PSI,PSI-CAROTENE #

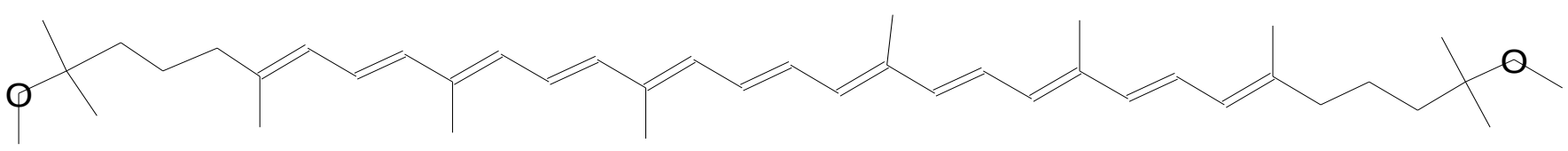

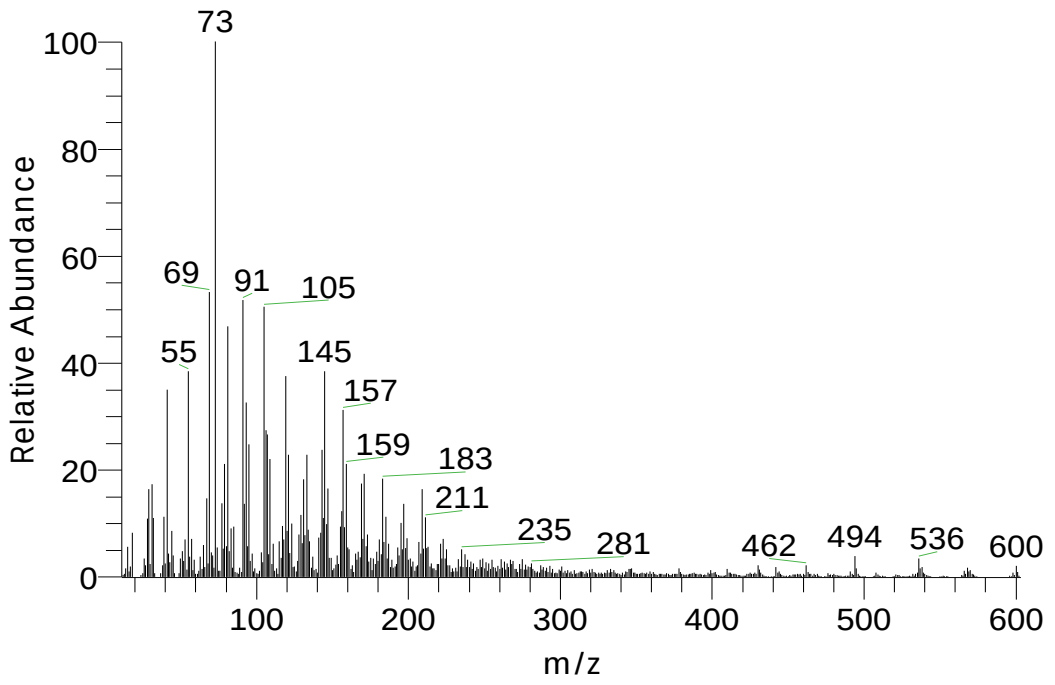

Loperamide
Formula C29H33ClN2O2, MW 476, CAS# 34552-83-5, Entry# 1181

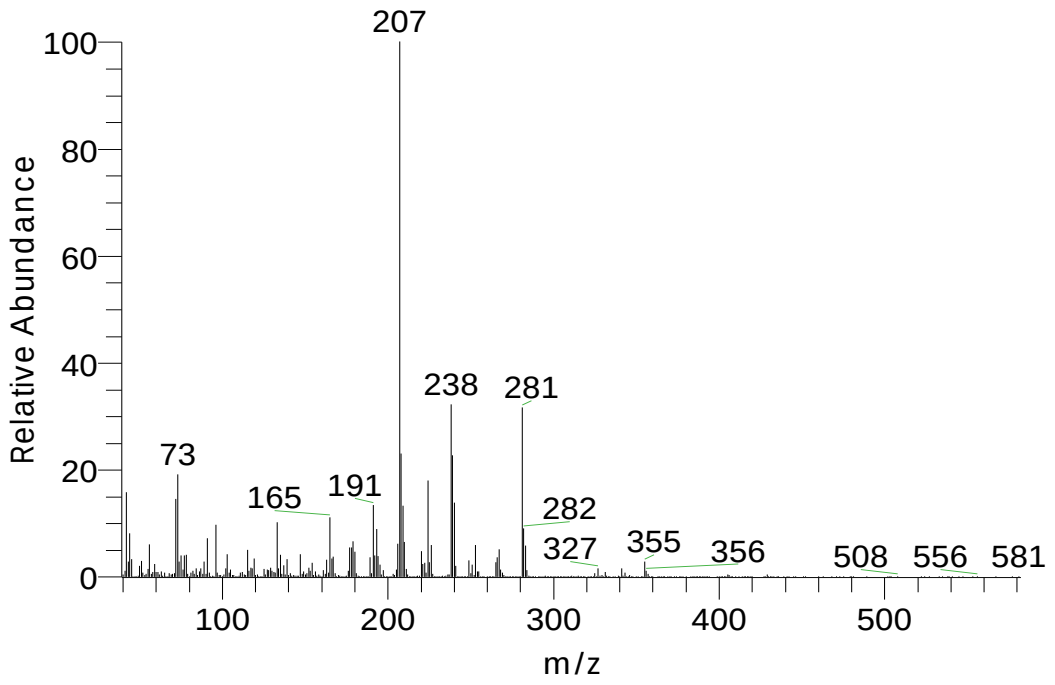

Oil_DrHafsa #11860 RT: 43.77 AV: 1 NL: 1.81E6
T: + c EI Full ms [50.000-750.000]

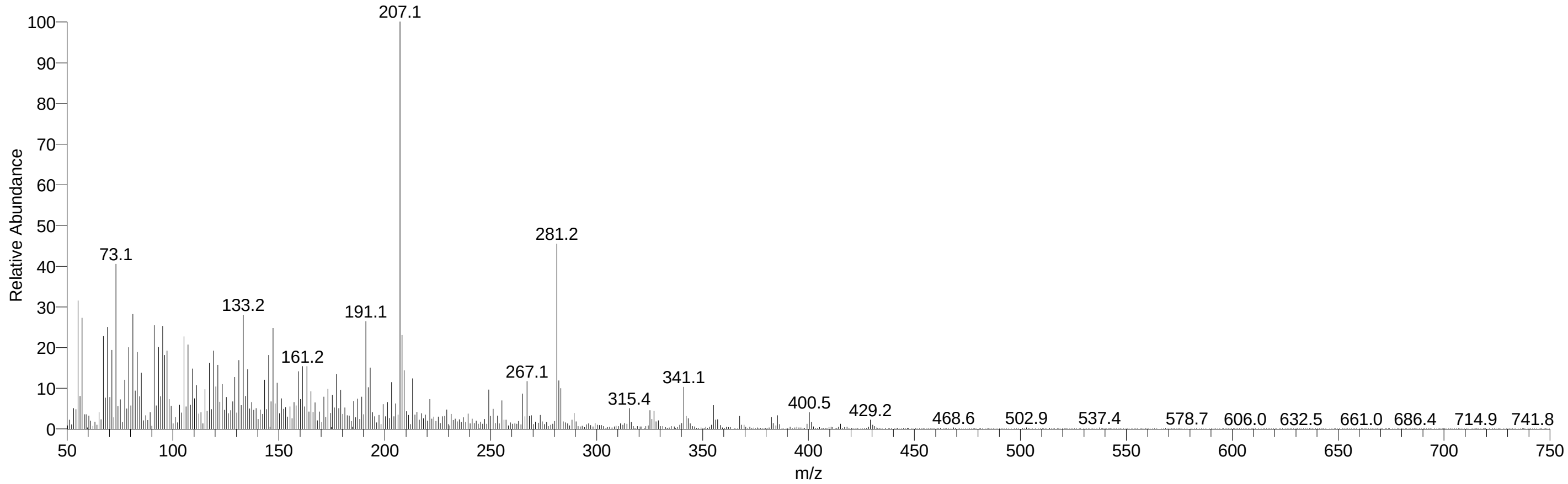

RT	Compound Name	Area %	MF	Molecular Formula	Molecular Weight	Cas #	Library
43.77	9,12-OCTADECADIENOIC ACID (Z,Z)-, 2,3-BIS[(TRIMETHYLSILYL)OXY]PROPYL ESTER	1.10	780	C27H54O4Si2	498	54284-45-6	WileyRegistry8e
43.77	.psi.,.psi.-Carotene, 1,1',2,2'-tetrahydro-1,1'-dimethoxy-	1.10	706	C42H64O2	600	13833-01-7	mainlib
43.77	.PSI.,.PSI.-CAROTENE, 1,1',2,2'-TETRAHYDRO-1,1'-DIMETHOXY-	1.10	705	C42H64O2	600	13833-01-7	WileyRegistry8e
43.77	à-D-GALACTOPYRANOSIDE, METHYL 2,3-BIS-O-(TRIMETHYLSILYL)-, CYCLIC BUTYLBORONATE	1.10	727	C17H37BO6Si2	404	56211-11-1	WileyRegistry8e
43.77	SILANE, TRIMETHYL[[(3á)-STIGMAST-5-EN-3-YL]OXY]-	1.10	749	C32H58OSi	486	2625-46-9	WileyRegistry8e

My GC-MS Report

Compound Structure	Hit Spectrum

9,12-OCTADECADIENOIC ACID (Z,Z)-, 2,3-BIS[(TRIMETHYLSILYL)OXY]PROPYL ESTER
Formula C27H54O4Si2, MW 498, CAS# 54284-45-6, Entry# 285148
2,3-BIS[(TRIMETHYLSILYL)OXY]PROPYL (9Z,12Z)-9,12-OCTADECADIENOATE #

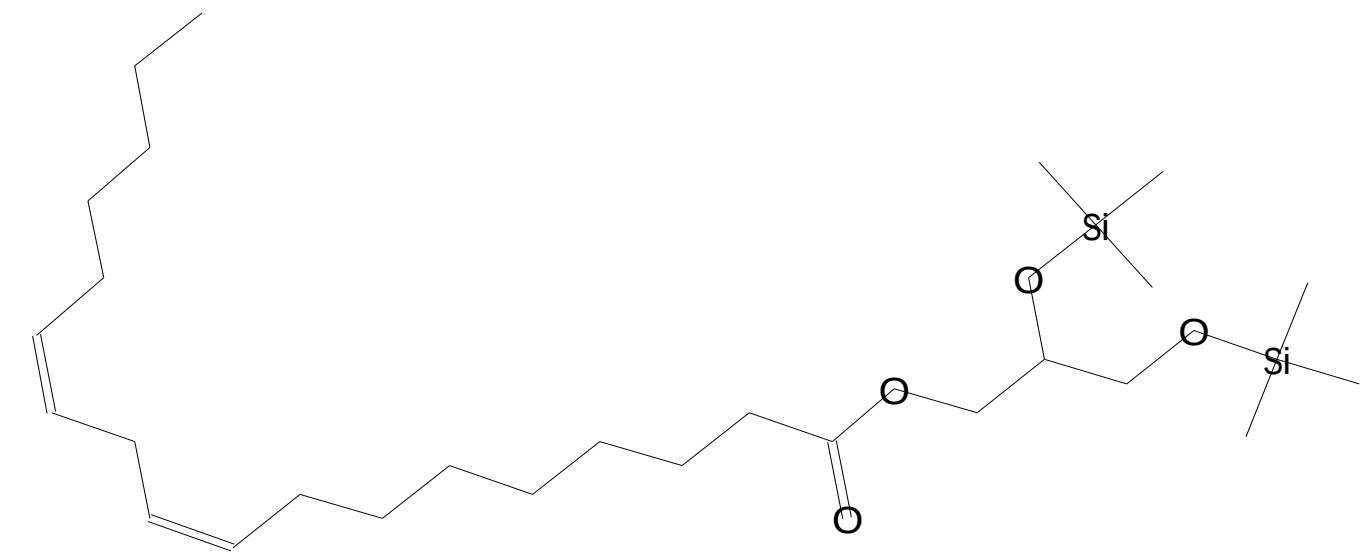

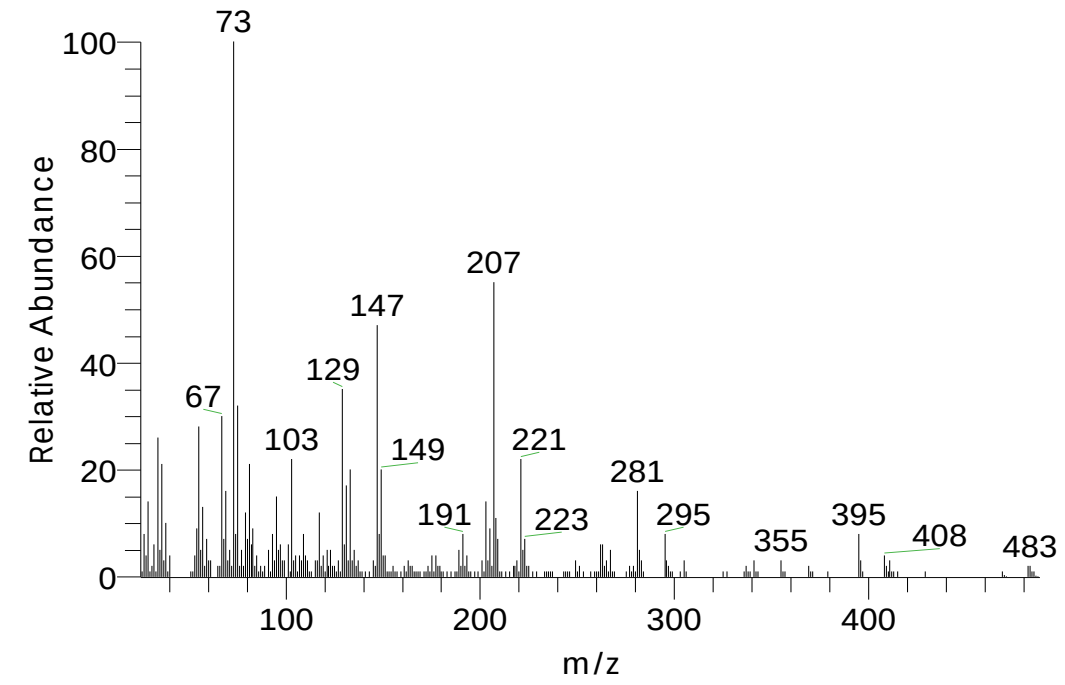

.psi.,.psi.-Carotene, 1,1',2,2'-tetrahydro-1,1'-dimethoxy-
Formula C42H64O2, MW 600, CAS# 13833-01-7, Entry# 41205
Lycopene, 1,1',2,2'-tetrahydro-1,1'-dimethoxy-, all-trans-

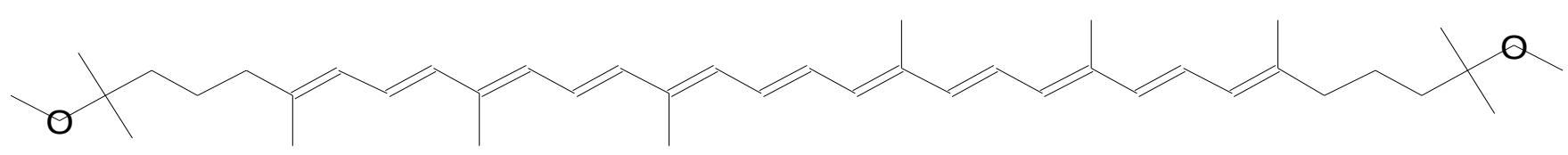

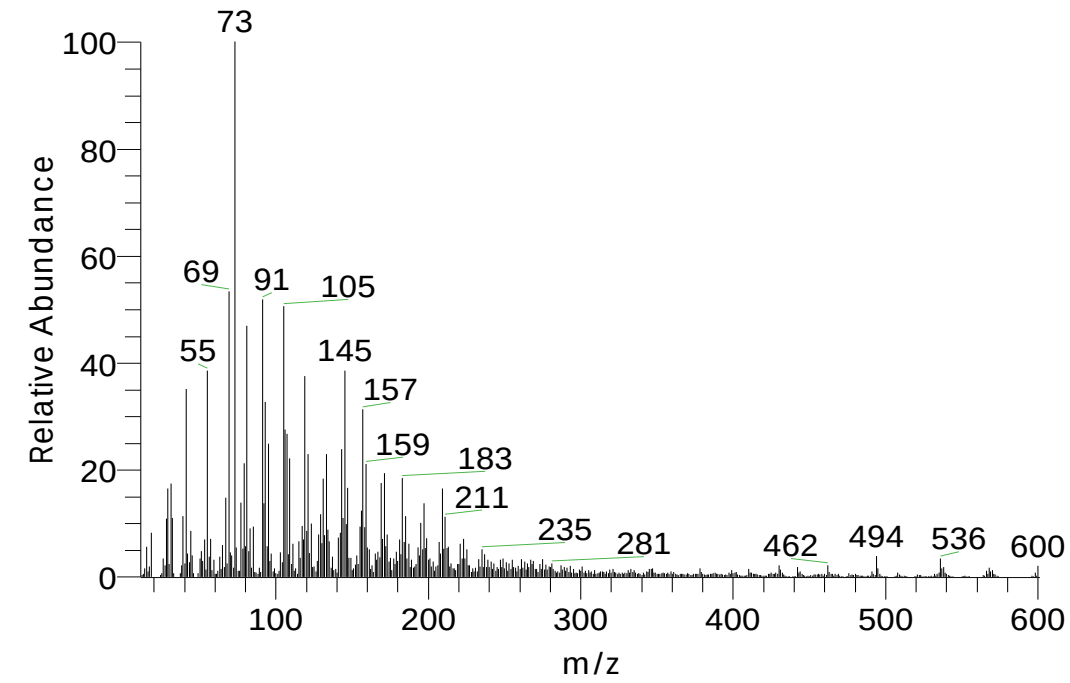

.PSI.,.PSI.-CAROTENE, 1,1',2,2'-TETRAHYDRO-1,1'-DIMETHOXY-
Formula C42H64O2, MW 600, CAS# 13833-01-7, Entry# 296796
1,1',2,2'-TETRAHYDRO-PSI,PSI-CAROTENE #

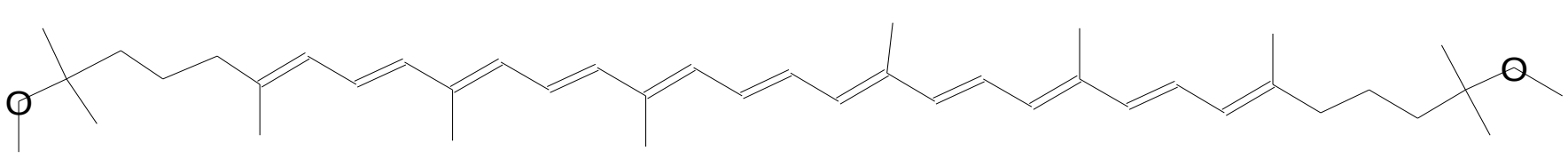

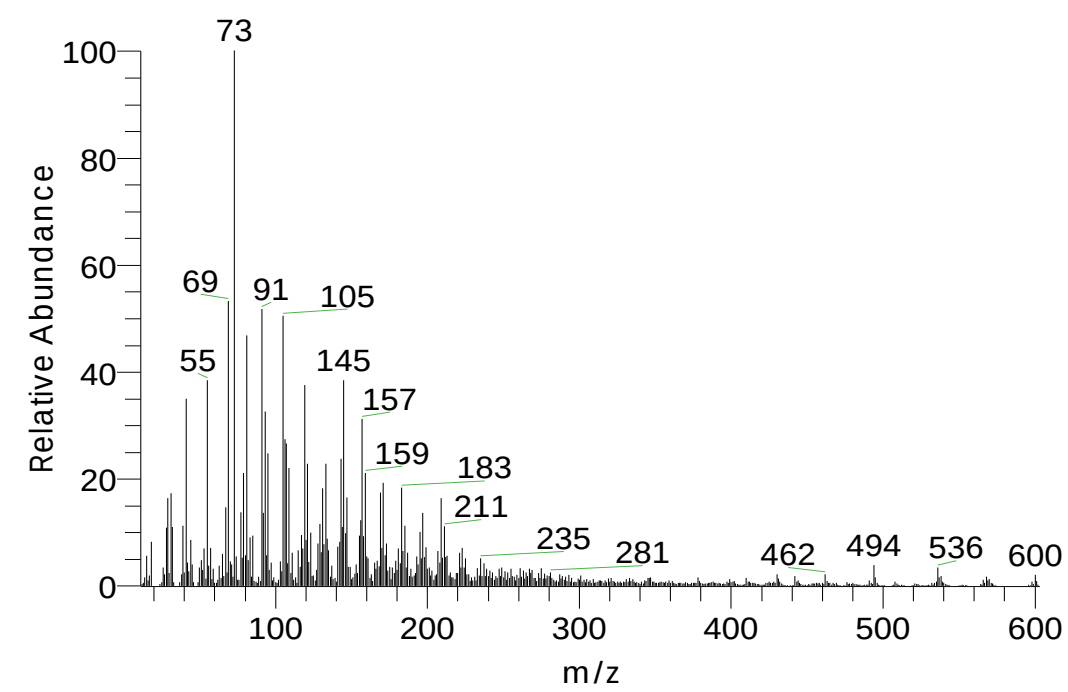

à-D-GALACTOPYRANOSIDE, METHYL 2,3-BIS-O-(TRIMETHYLSILYL)-, CYCLIC BUTYLBORONATE
Formula C17H37BO6Si2, MW 404, CAS# 56211-11-1, Entry# 257663

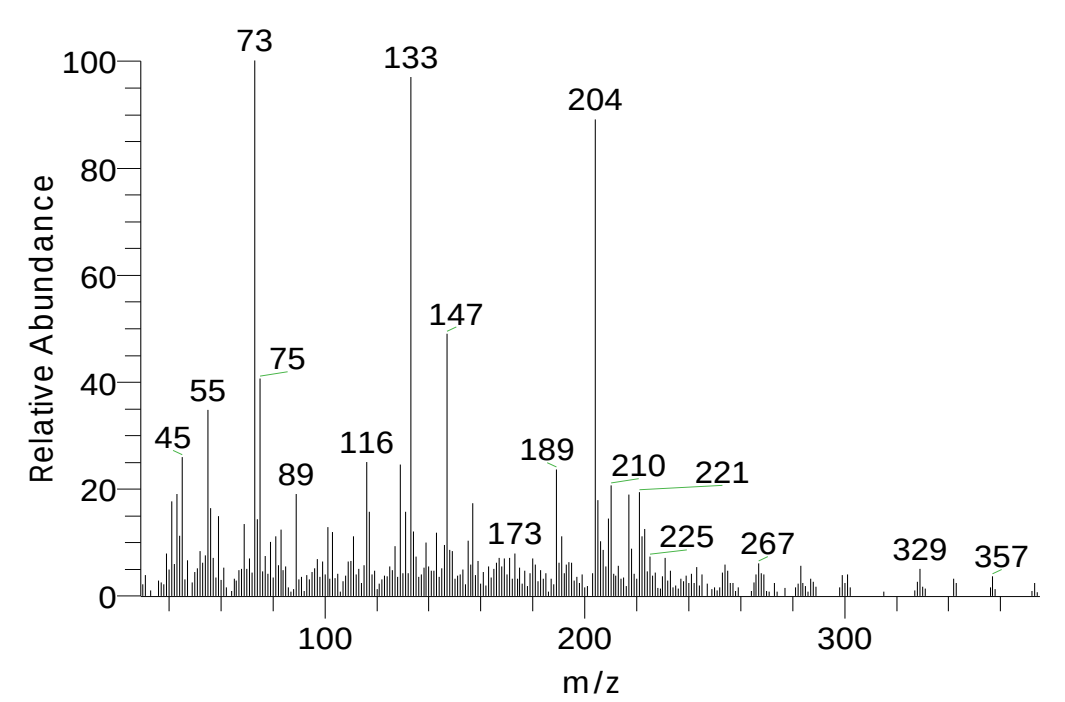

My GC-MS Report

Compound Structure | Hit Spectrum

SILANE, TRIMETHYL[[(3á)-STIGMAST-5-EN-3-YL]OXY]-
Formula C32H58OSi, MW 486, CAS# 2625-46-9, Entry# 283210
3-[(TRIMETHYLSILYL)OXY]STIGMAST-5-ENE #

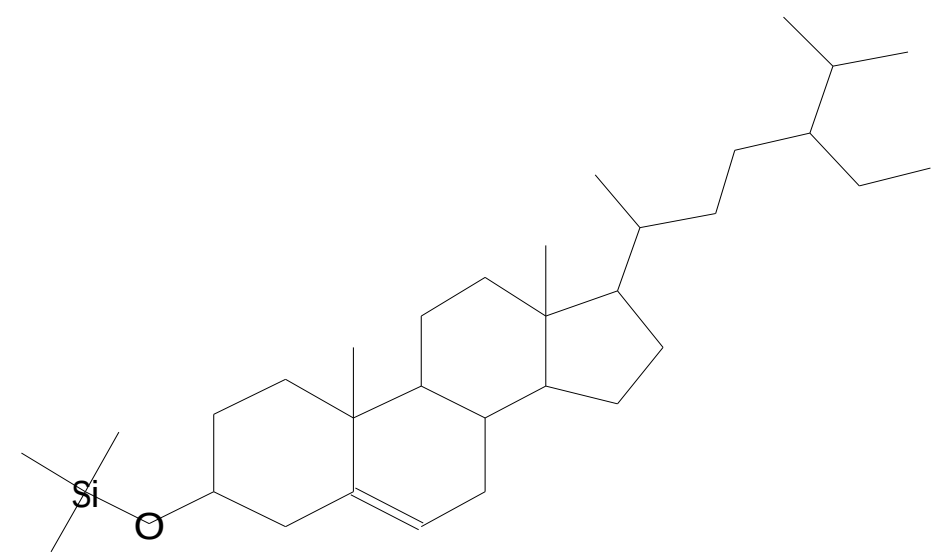

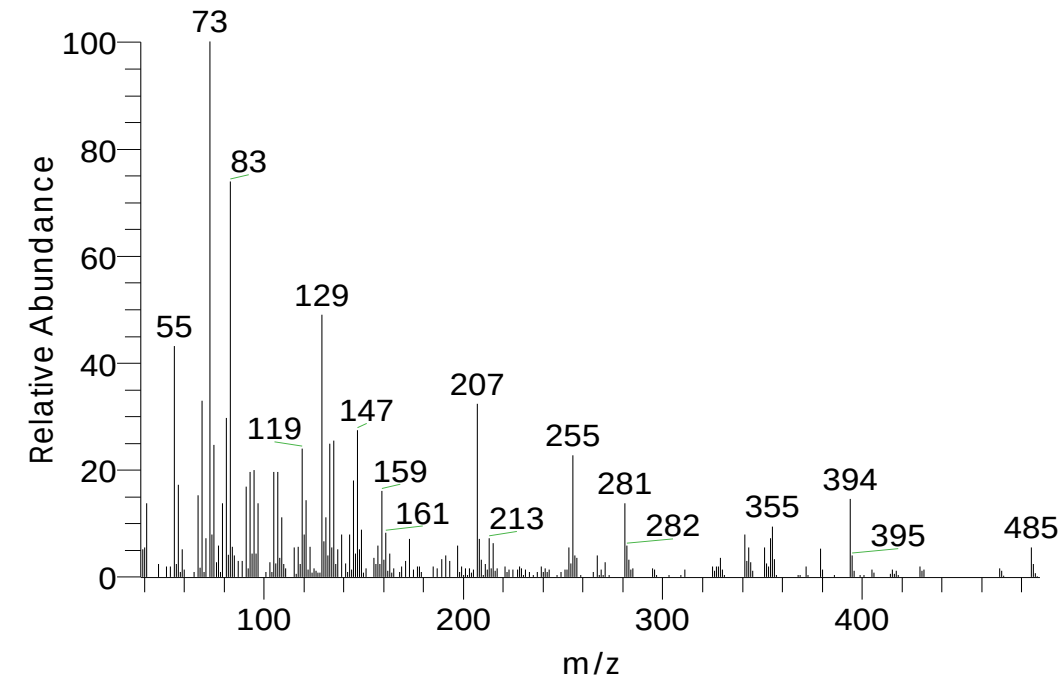

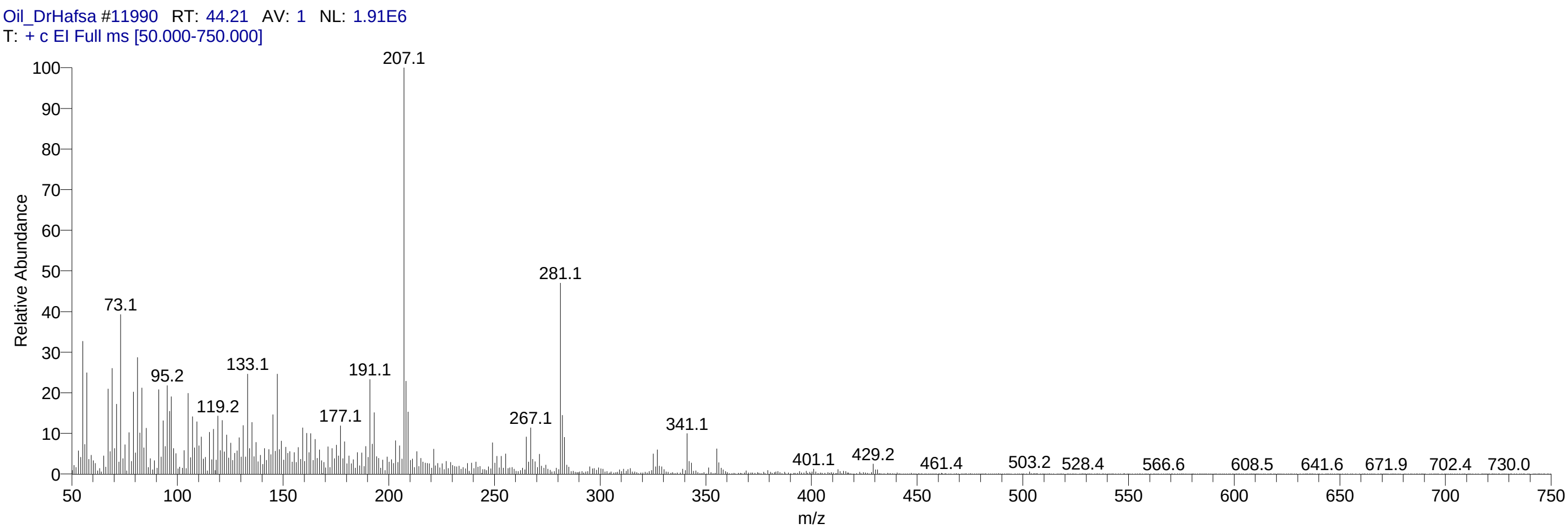

RT	Compound Name	Area %	MF	Molecular Formula	Molecular Weight	Cas #	Library
44.21	4H-1-BENZOPYRAN-4-ONE, 2-(3,4-DIMETHOXYPHENYL)-3,5-DIHYDROXY-7-METHOXY-	0.92	750	C18H16O7	344	6068-80-0	WileyRegistry8e
44.21	ETHYL ISO-ALLOCHOLATE	0.92	739	C26H44O5	436	NA	WileyRegistry8e
44.21	Ethyl iso-allocholate	0.92	738	C26H44O5	436	NA	mainlib
44.21	9,12-OCTADECADIENOIC ACID (Z,Z)-, 2,3-BIS[(TRIMETHYLSILYL)OXY]PROPYL ESTER	0.92	781	C27H54O4Si2	498	54284-45-6	WileyRegistry8e
44.21	03027205002 FLAVONE 4'-OH,5-OH,7-DI-O-GLUCOSIDE	0.92	702	C27H30O15	594	NA	WileyRegistry8e

Compound Structure | Hit Spectrum

4H-1-BENZOPYRAN-4-ONE, 2-(3,4-DIMETHOXYPHENYL)-3,5-DIHYDROXY-7-METHOXY-
Formula C18H16O7, MW 344, CAS# 6068-80-0, Entry# 224392
3',4',7-TRIMETHYLQUERCETIN

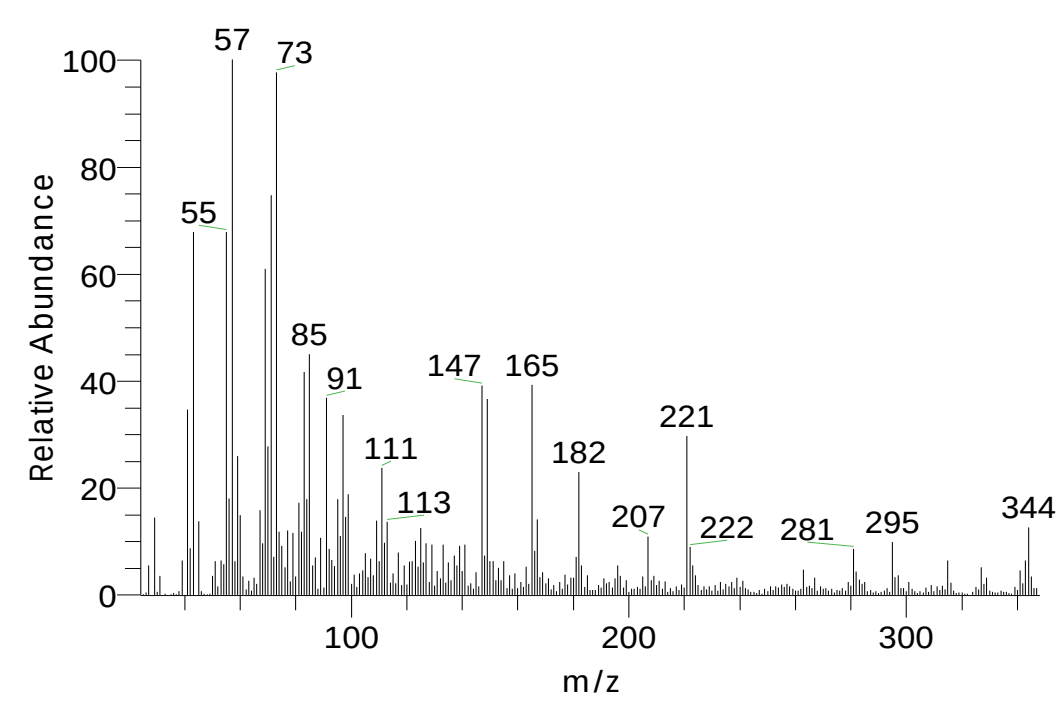

My GC-MS Report

Compound Structure | Hit Spectrum

ETHYL ISO-ALLOCHOLATE
Formula C26H44O5, MW 436, CAS# NA, Entry# 270212

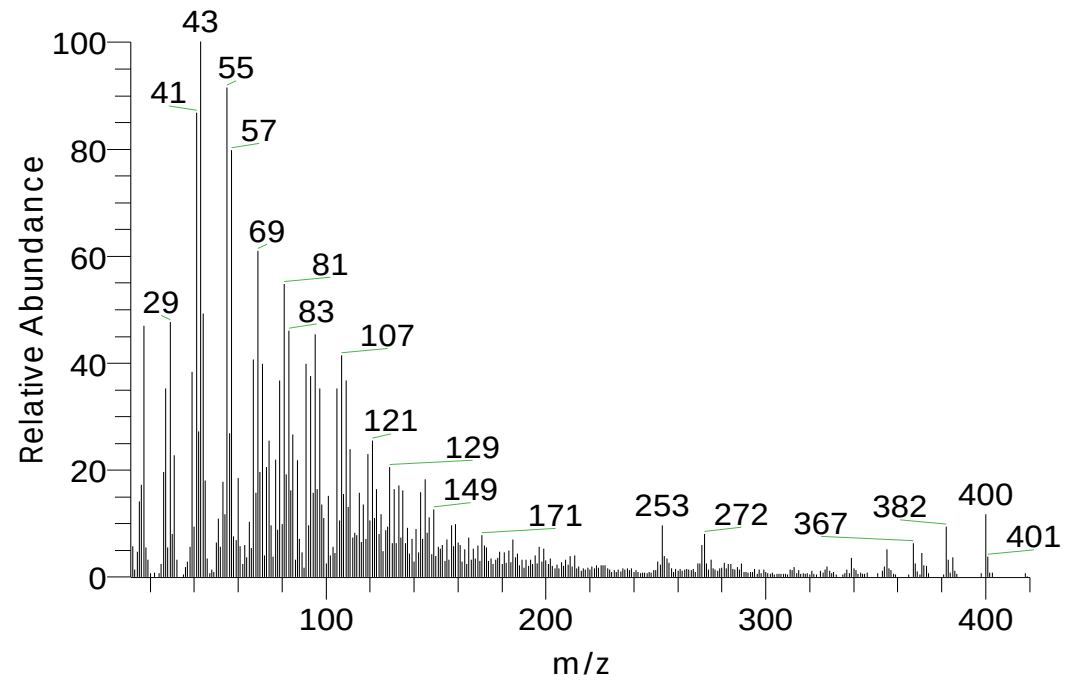

SI 728, RSI 738, mainlib, Entry# 7020, CAS# NA, Ethyl iso-allocholate

Ethyl iso-allocholate
Formula C26H44O5, MW 436, CAS# NA, Entry# 7020
Ethyl 3,7,12-trihydroxycholan-24-oate #

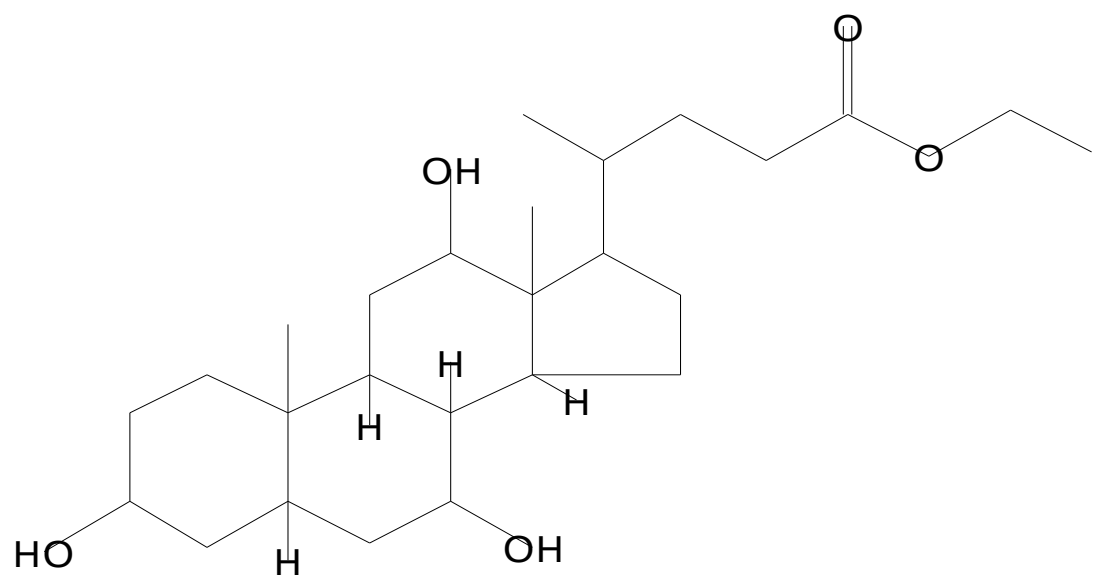

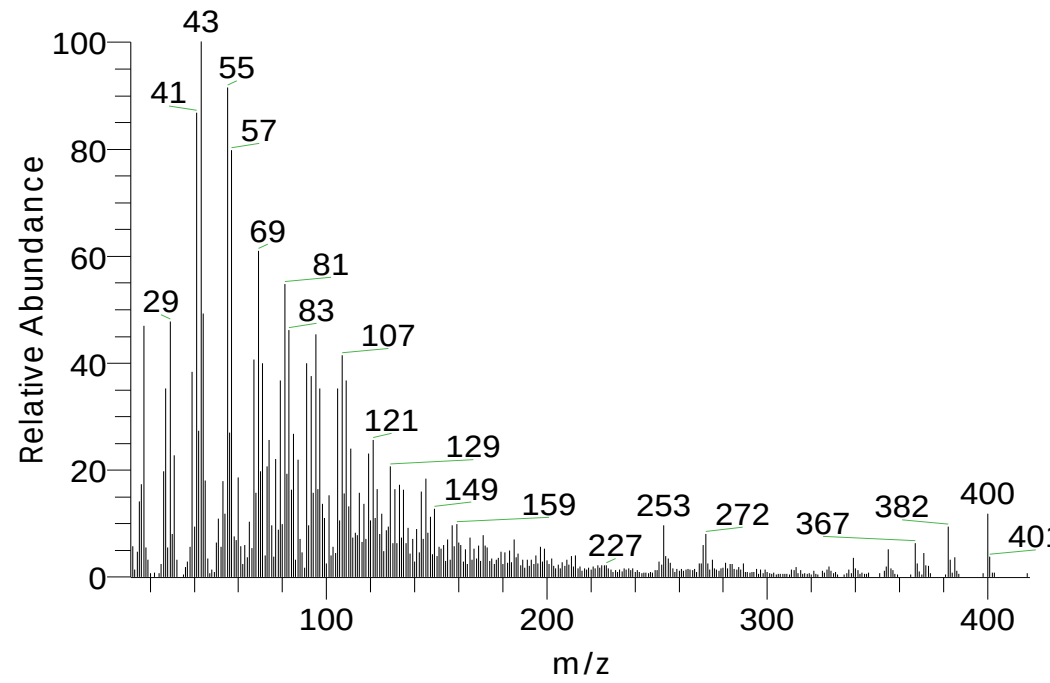

9,12-OCTADECADIENOIC ACID (Z,Z)-, 2,3-BIS[(TRIMETHYLSILYL)OXY]PROPYL ESTER
Formula C27H54O4Si2, MW 498, CAS# 54284-45-6, Entry# 285148
2,3-BIS[(TRIMETHYLSILYL)OXY]PROPYL (9Z,12Z)-9,12-OCTADECADIENOATE #

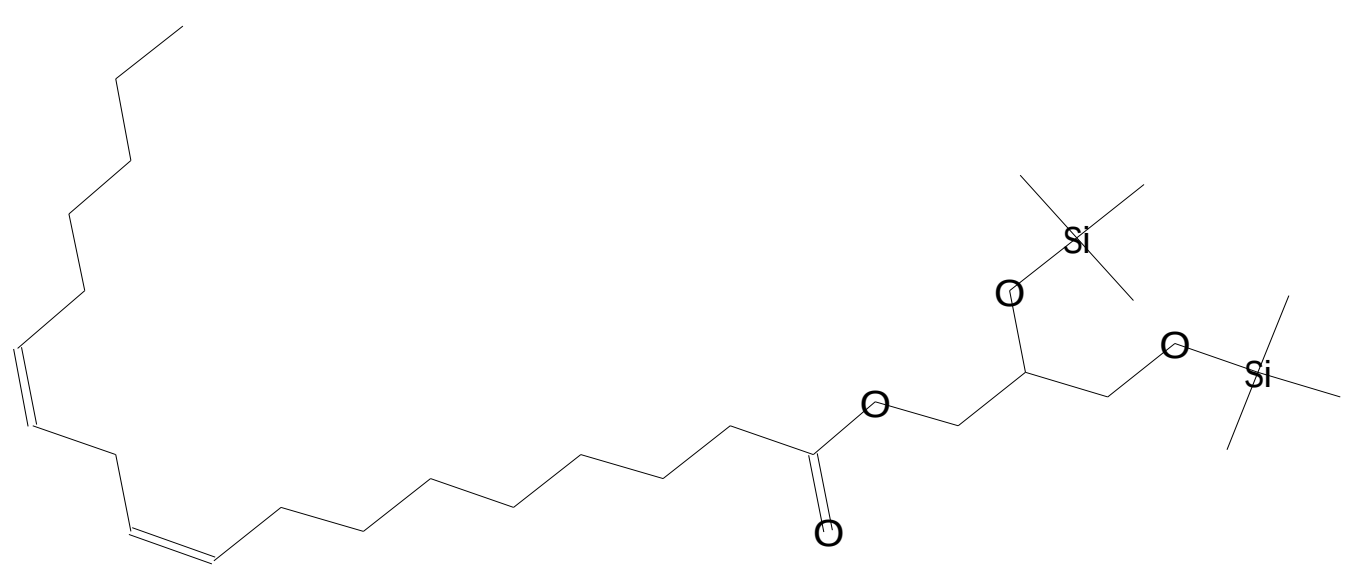

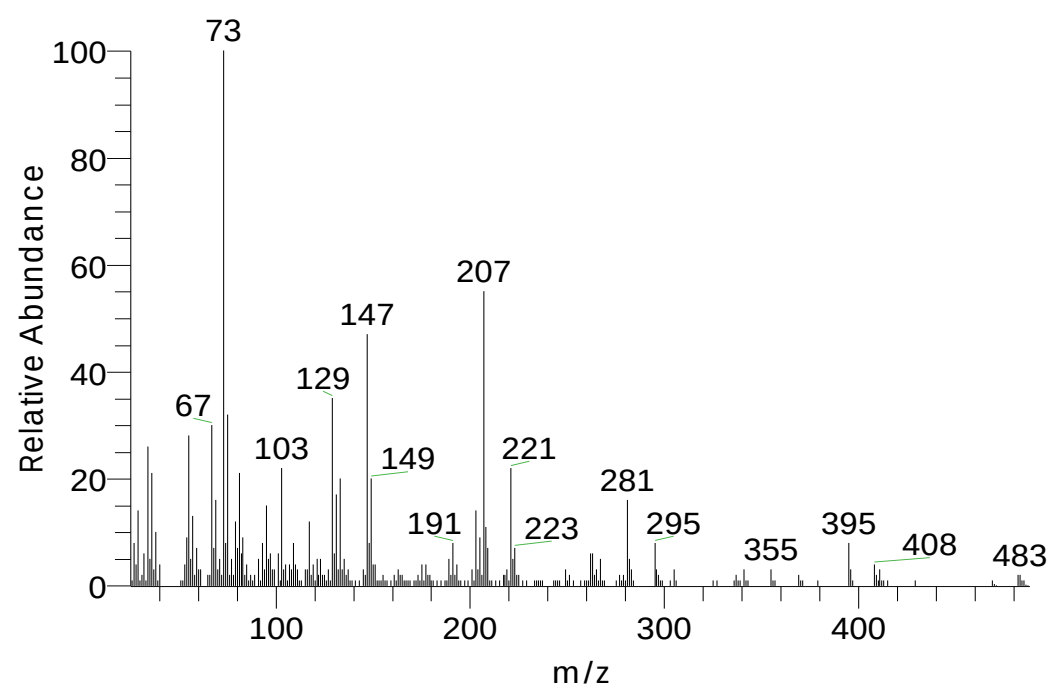

03027205002 FLAVONE 4'-OH,5-OH,7-DI-O-GLUCOSIDE
Formula C27H30O15, MW 594, CAS# NA, Entry# 296184

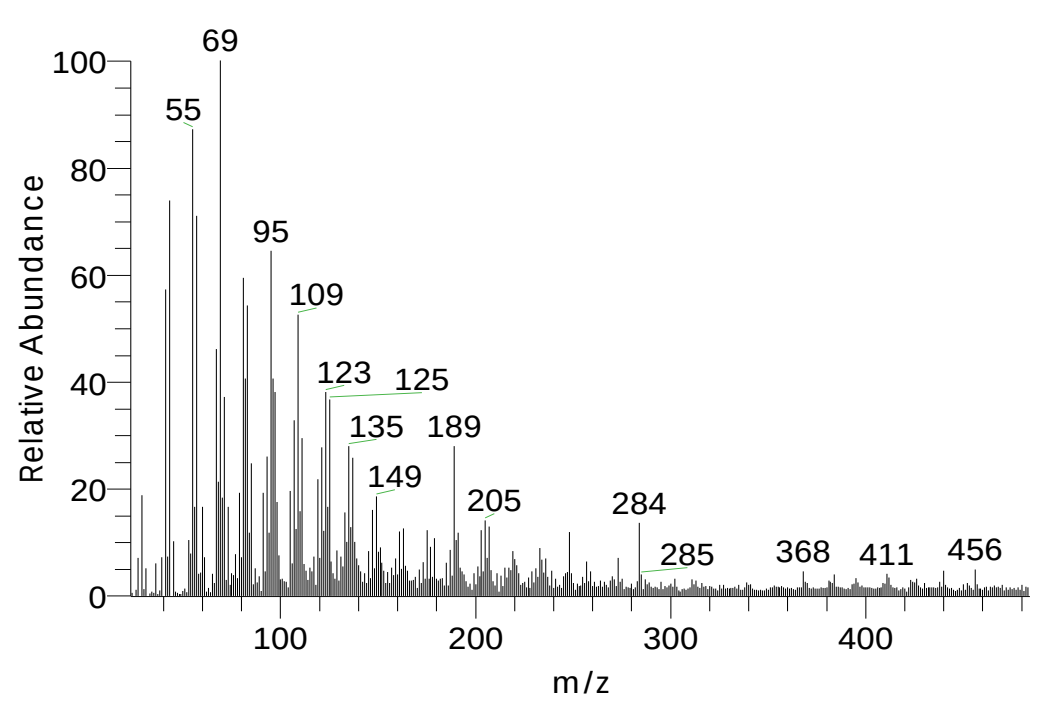

My GC-MS Report

Oil_DrHafsa #12077 RT: 44.50 AV: 1 NL: 1.88E6
T: + c EI Full ms [50.000-750.000]

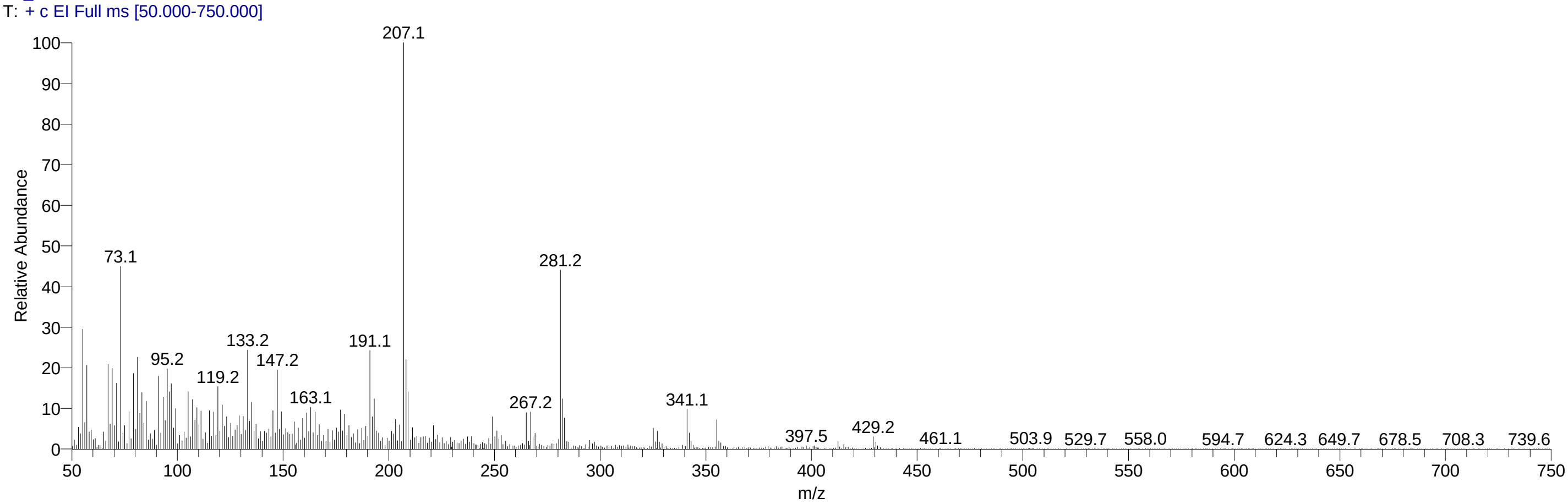

RT	Compound Name	Area %	MF	Molecular Formula	Molecular Weight	Cas #	Library
44.50	9,12-OCTADECADIENOIC ACID (Z,Z)-, 2,3-BIS[(TRIMETHYLSILYL)OXY]PROPYL ESTER	0.42	785	C27H54O4Si2	498	54284-45-6	WileyRegistry8e
44.50	Loperamide	0.42	676	C29H33ClN2O2	476	34552-83-5	CaymanSpectralLibrary-NIST.HP
44.50	W-18	0.42	672	C19H20ClN3O4S	421	93101-02-1	CaymanSpectralLibrary-NIST.HP
44.50	à-D-GALACTOPYRANOSIDE, METHYL 2,3-BIS-O-(TRIMETHYLSILYL)-, CYCLIC BUTYLBORONATE	0.42	717	C17H37BO6Si2	404	56211-11-1	WileyRegistry8e
44.50	SILANE, TRIMETHYL[[(3á)-STIGMAST-5-EN-3-YL]OXY]-	0.42	721	C32H58OSi	486	2625-46-9	WileyRegistry8e

Compound Structure

Hit Spectrum

9,12-OCTADECADIENOIC ACID (Z,Z)-, 2,3-BIS[(TRIMETHYLSILYL)OXY]PROPYL ESTER
Formula C27H54O4Si2, MW 498, CAS# 54284-45-6, Entry# 285148
2,3-BIS[(TRIMETHYLSILYL)OXY]PROPYL (9Z,12Z)-9,12-OCTADECADIENOATE #

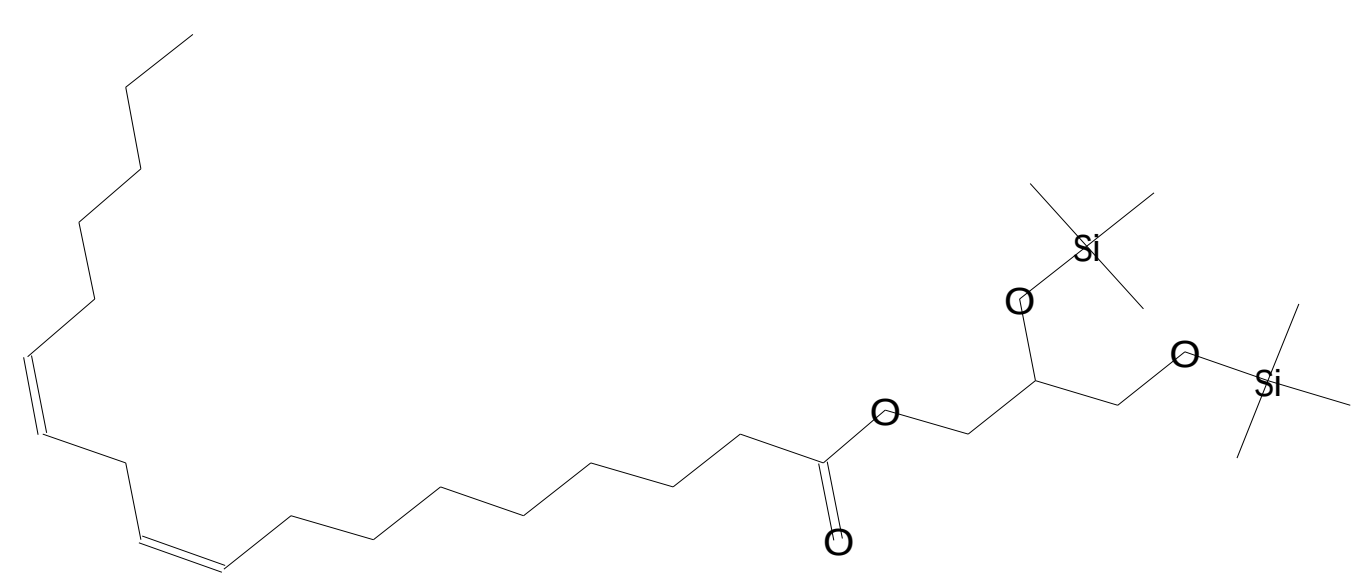

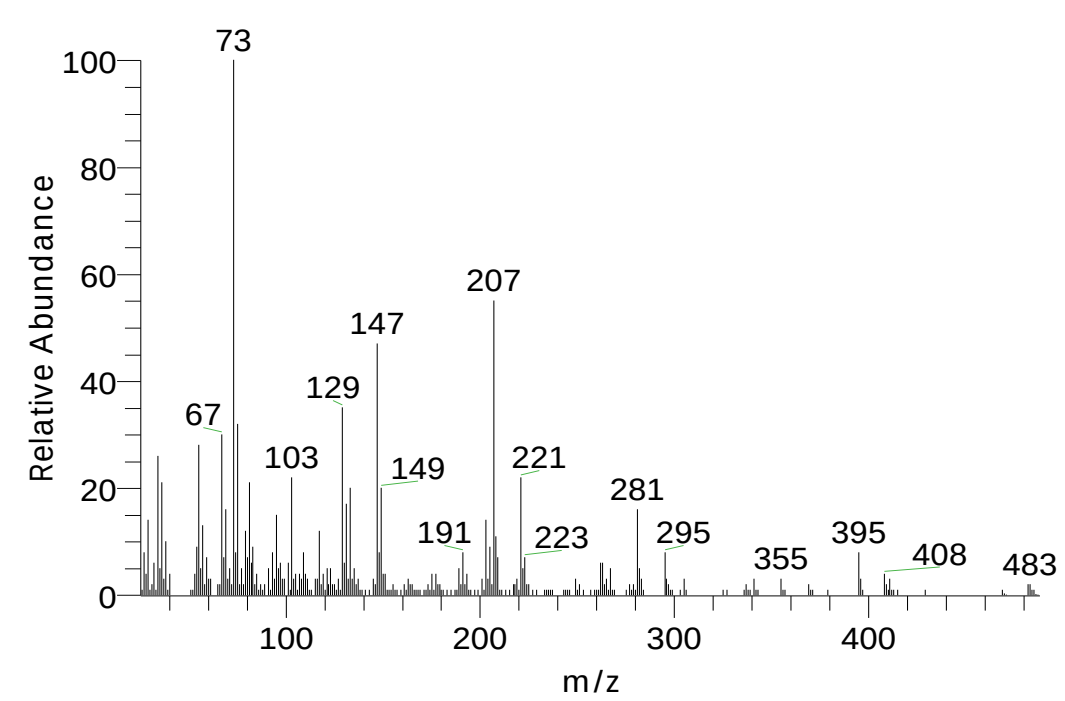

Loperamide
Formula C29H33ClN2O2, MW 476, CAS# 34552-83-5, Entry# 1181

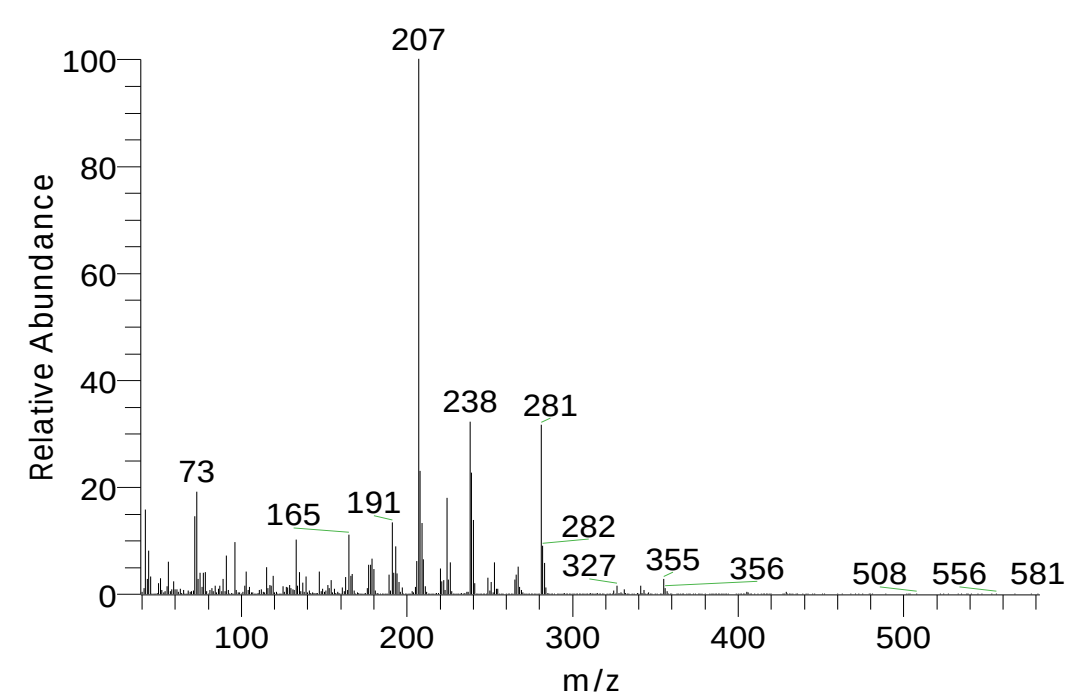

My GC-MS Report

Compound Structure | Hit Spectrum

W-18
Formula C19H20ClN3O4S, MW 421, CAS# 93101-02-1, Entry# 949

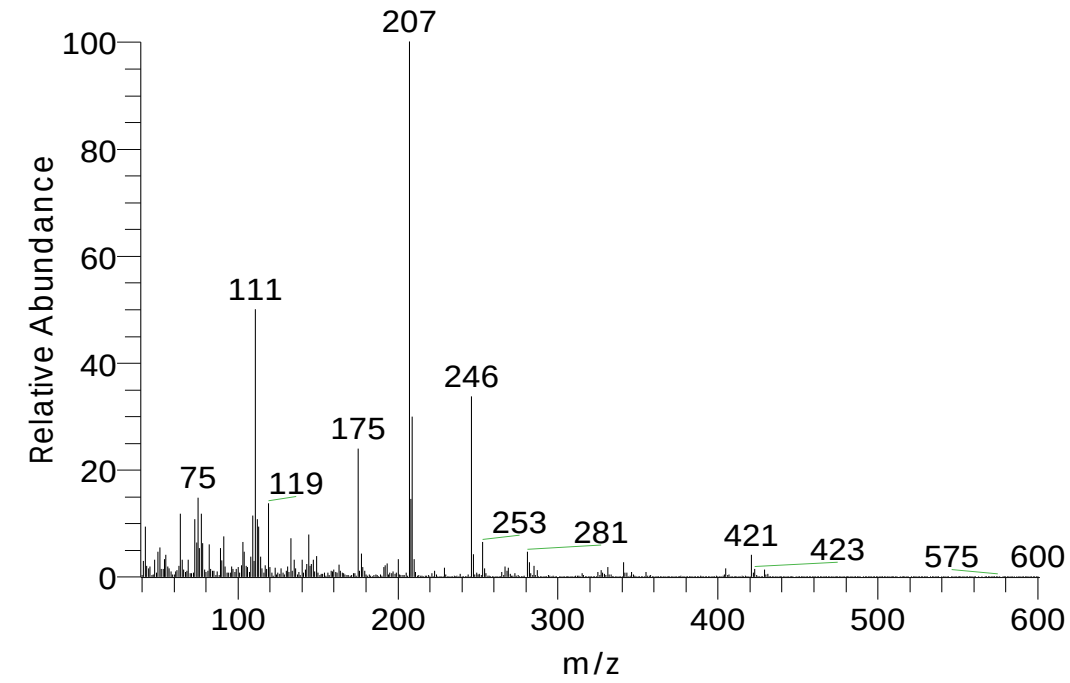

à-D-GALACTOPYRANOSIDE, METHYL 2,3-BIS-O-(TRIMETHYLSILYL)-, CYCLIC BUTYLBORONATE
Formula C17H37BO6Si2, MW 404, CAS# 56211-11-1, Entry# 257663

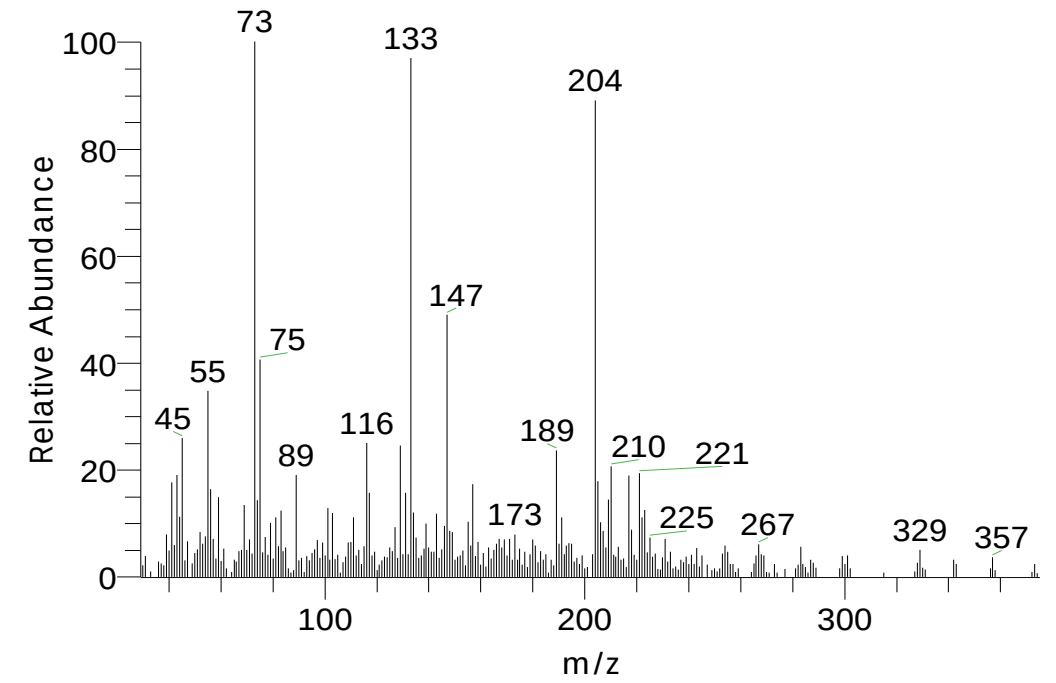

SILANE, TRIMETHYL[[(3á)-STIGMAST-5-EN-3-YL]OXY]-
Formula C32H58OSi, MW 486, CAS# 2625-46-9, Entry# 283210
3-[(TRIMETHYLSILYL)OXY]STIGMAST-5-ENE #

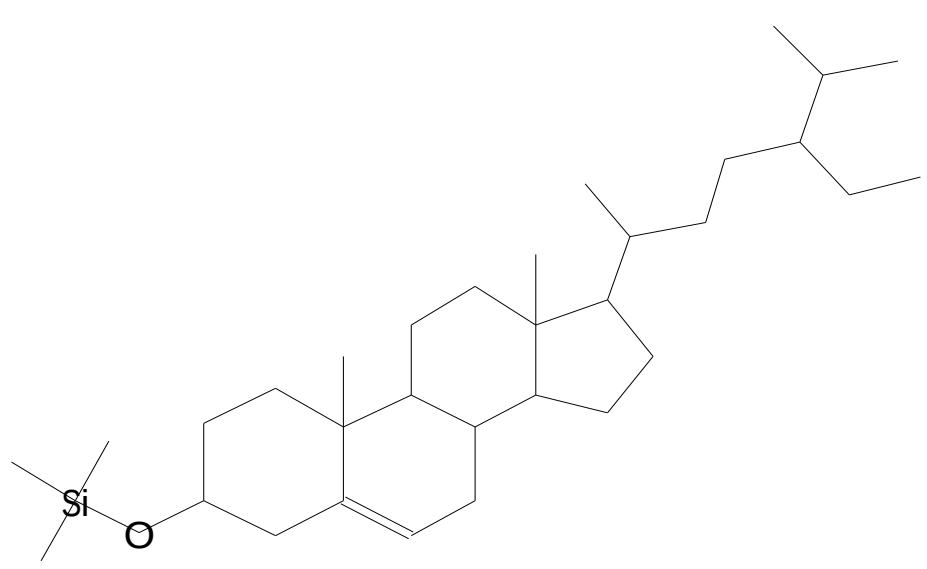

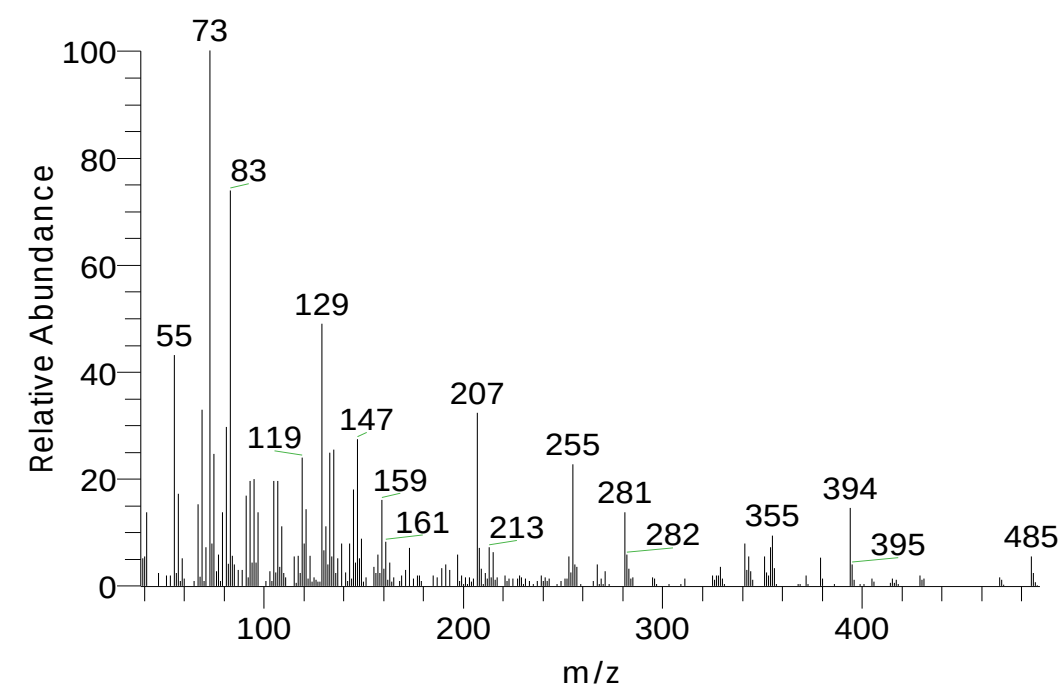

Oil_DrHafsa #12160 RT: 44.78 AV: 1 NL: 1.93E6
T: + c EI Full ms [50.000-750.000]

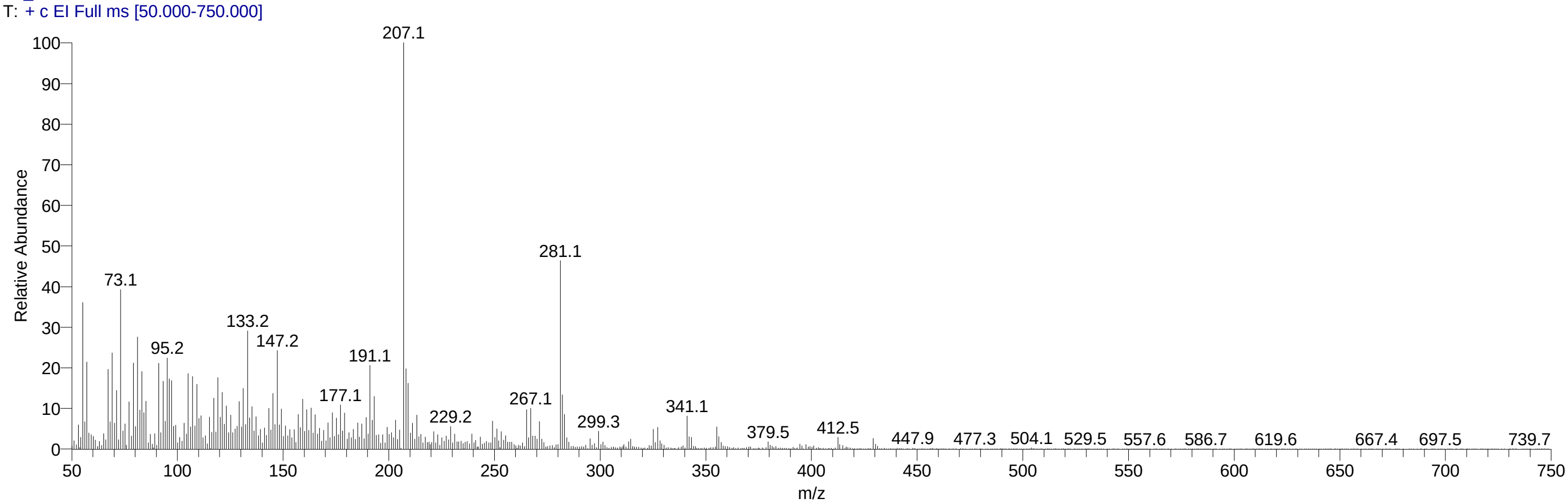

RT	Compound Name	Area %	MF	Molecular Formula	Molecular Weight	Cas #	Library
44.78	.psi.,.psi.-Carotene, 1,1',2,2'-tetrahydro-1,1'-dimethoxy-	1.21	714	C42H64O2	600	13833-01-7	mainlib
44.78	.PSI.,.PSI.-CAROTENE, 1,1',2,2'-TETRAHYDRO-1,1'-DIMETHOXY-	1.21	713	C42H64O2	600	13833-01-7	WileyRegistry8e

My GC-MS Report

RT	Compound Name	Area %	MF	Molecular Formula	Molecular Weight	Cas #	Library
44.78	9,12-OCTADECADIENOIC ACID (Z,Z)-, 2,3-BIS[(TRIMETHYLSILYL)OXY]PROPYL ESTER	1.21	771	C27H54O4Si2	498	54284-45-6	WileyRegistry8e
44.78	à-D-GALACTOPYRANOSIDE, METHYL 2,3-BIS-O-(TRIMETHYLSILYL)-, CYCLIC BUTYLBORONATE	1.21	717	C17H37BO6Si2	404	56211-11-1	WileyRegistry8e
44.78	SILANE, TRIMETHYL[[(3á)-STIGMAST-5-EN-3-YL]OXY]-	1.21	741	C32H58OSi	486	2625-46-9	WileyRegistry8e

Compound Structure | Hit Spectrum

.psi.,.psi.-Carotene, 1,1',2,2'-tetrahydro-1,1'-dimethoxy-
Formula C42H64O2, MW 600, CAS# 13833-01-7, Entry# 41205
Lycopene, 1,1',2,2'-tetrahydro-1,1'-dimethoxy-, all-trans-

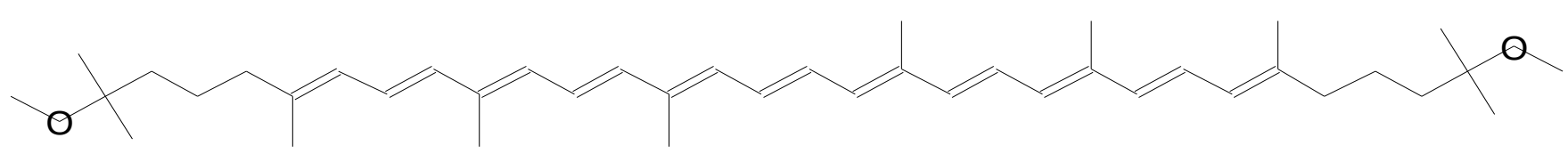

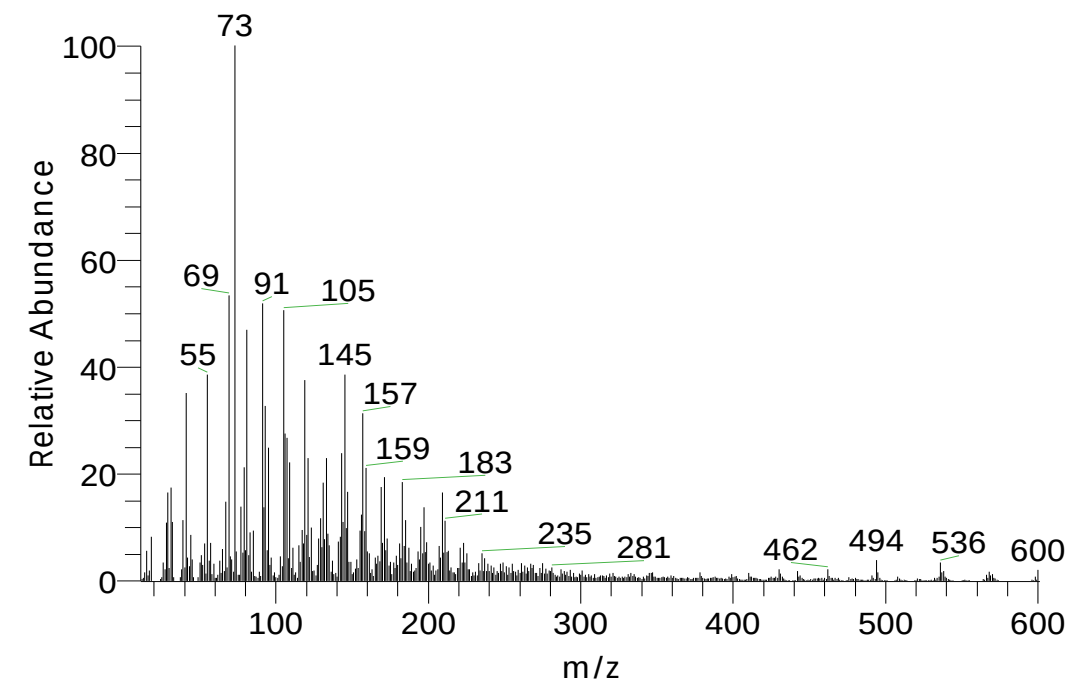

.PSI.,.PSI.-CAROTENE, 1,1',2,2'-TETRAHYDRO-1,1'-DIMETHOXY-
Formula C42H64O2, MW 600, CAS# 13833-01-7, Entry# 296796
1,1',2,2'-TETRAHYDRO-PSI,PSI-CAROTENE #

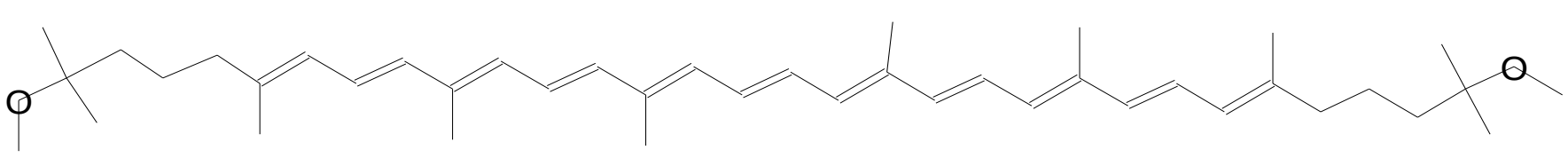

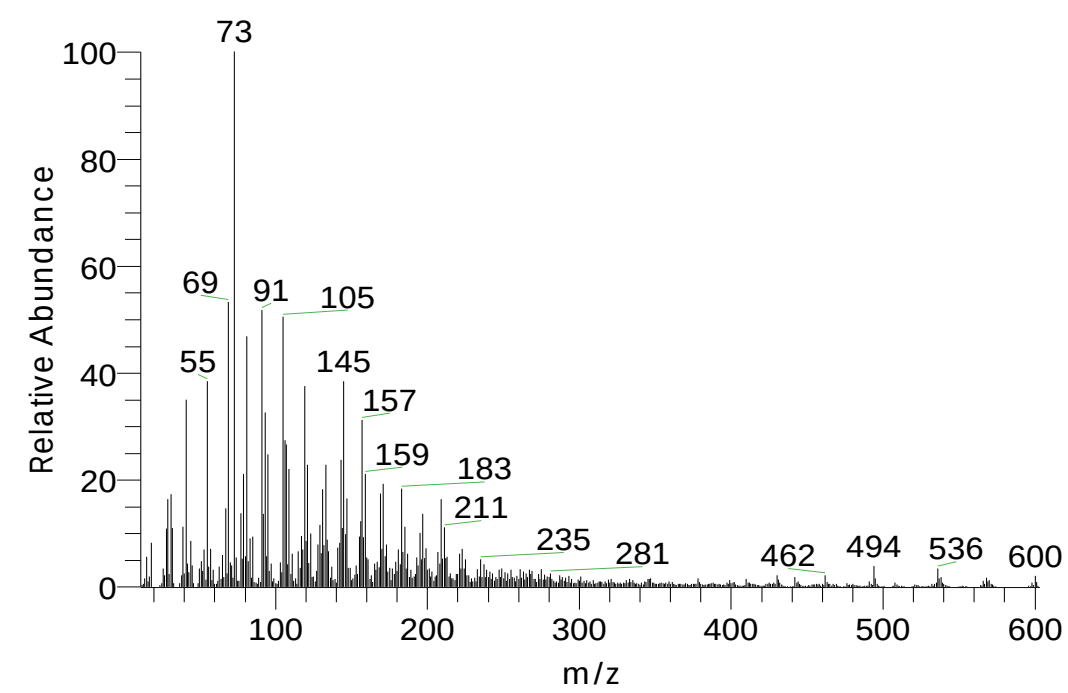

9,12-OCTADECADIENOIC ACID (Z,Z)-, 2,3-BIS[(TRIMETHYLSILYL)OXY]PROPYL ESTER
Formula C27H54O4Si2, MW 498, CAS# 54284-45-6, Entry# 285148
2,3-BIS[(TRIMETHYLSILYL)OXY]PROPYL (9Z,12Z)-9,12-OCTADECADIENOATE #

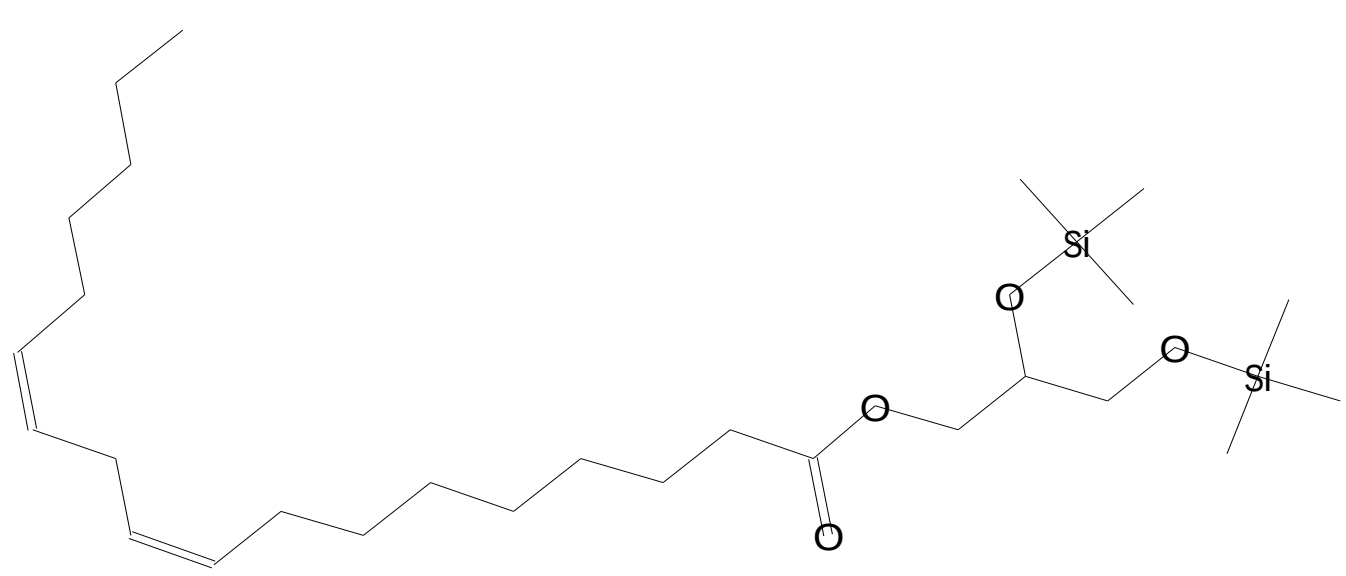

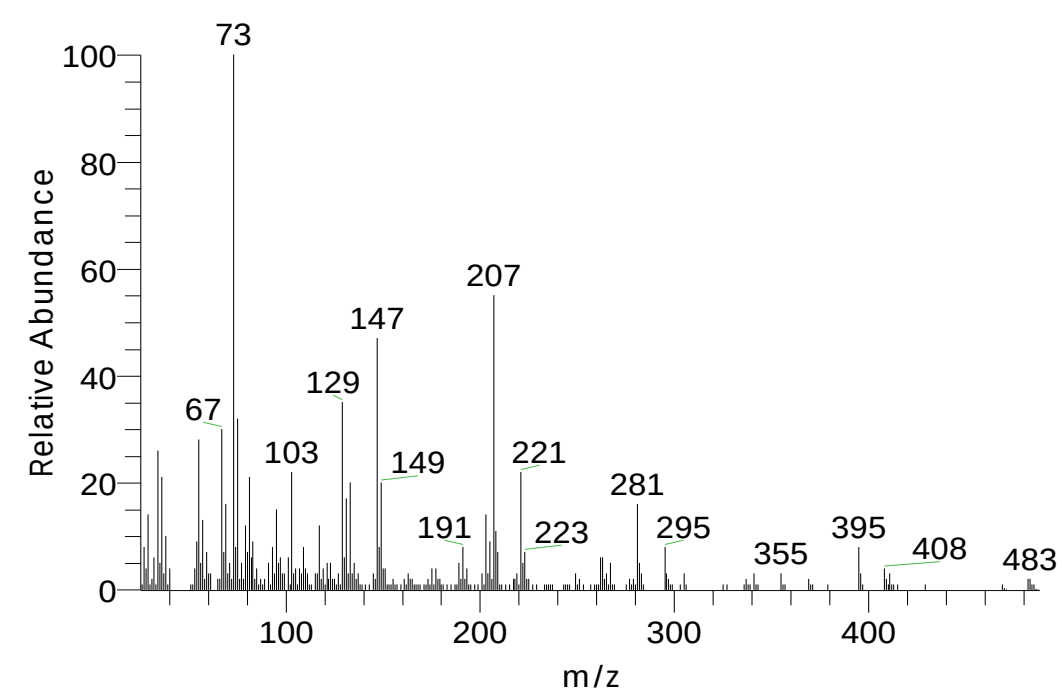

My GC-MS Report

Compound Structure | Hit Spectrum

à-D-GALACTOPYRANOSIDE, METHYL 2,3-BIS-O-(TRIMETHYLSILYL)-, CYCLIC BUTYLBORONATE
Formula C17H37BO6Si2, MW 404, CAS# 56211-11-1, Entry# 257663

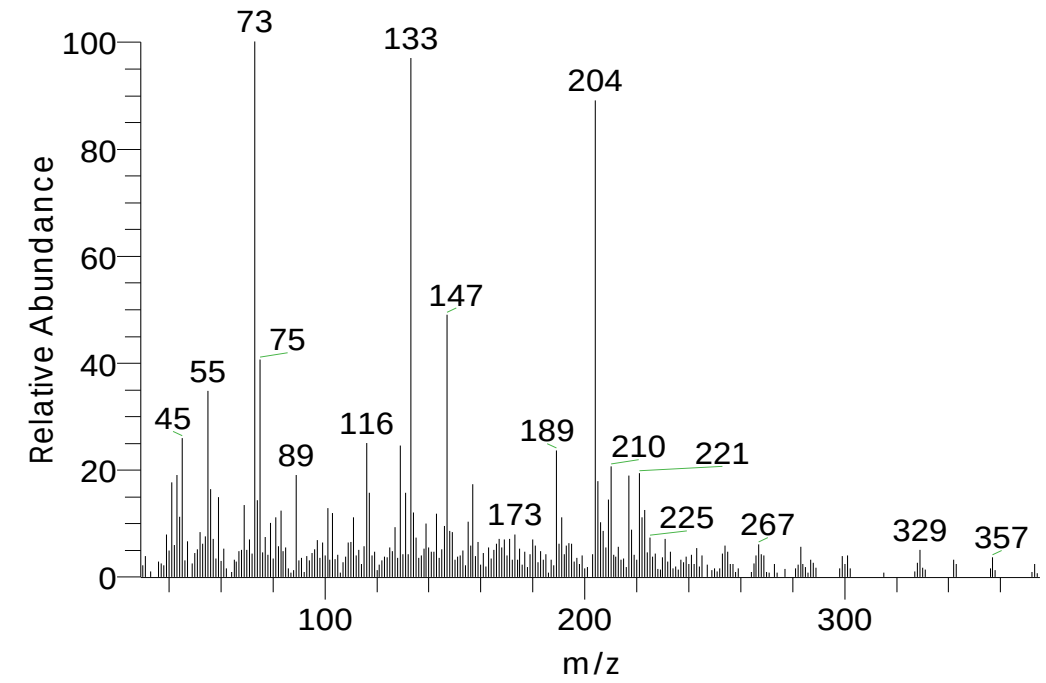

SILANE, TRIMETHYL[[(3á)-STIGMAST-5-EN-3-YL]OXY]-
Formula C32H58OSi, MW 486, CAS# 2625-46-9, Entry# 283210
3-[(TRIMETHYLSILYL)OXY]STIGMAST-5-ENE #

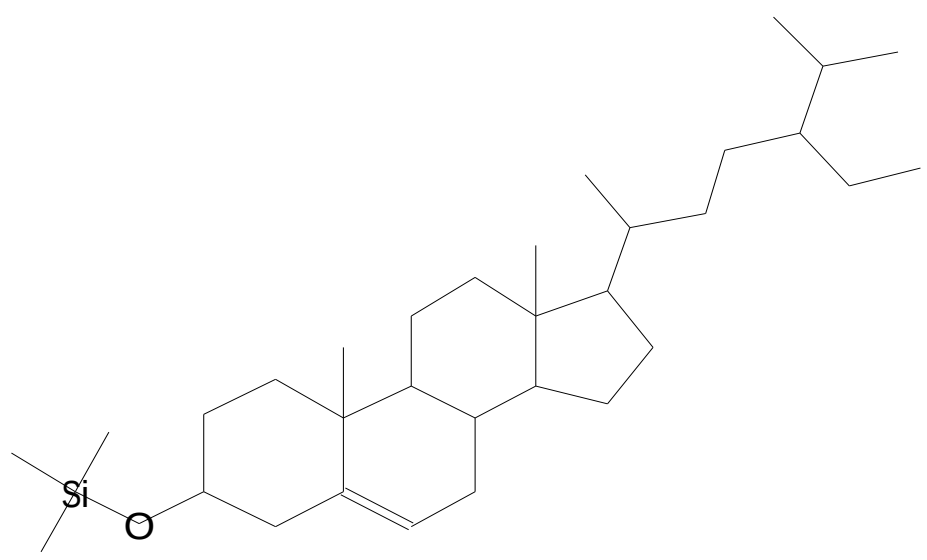

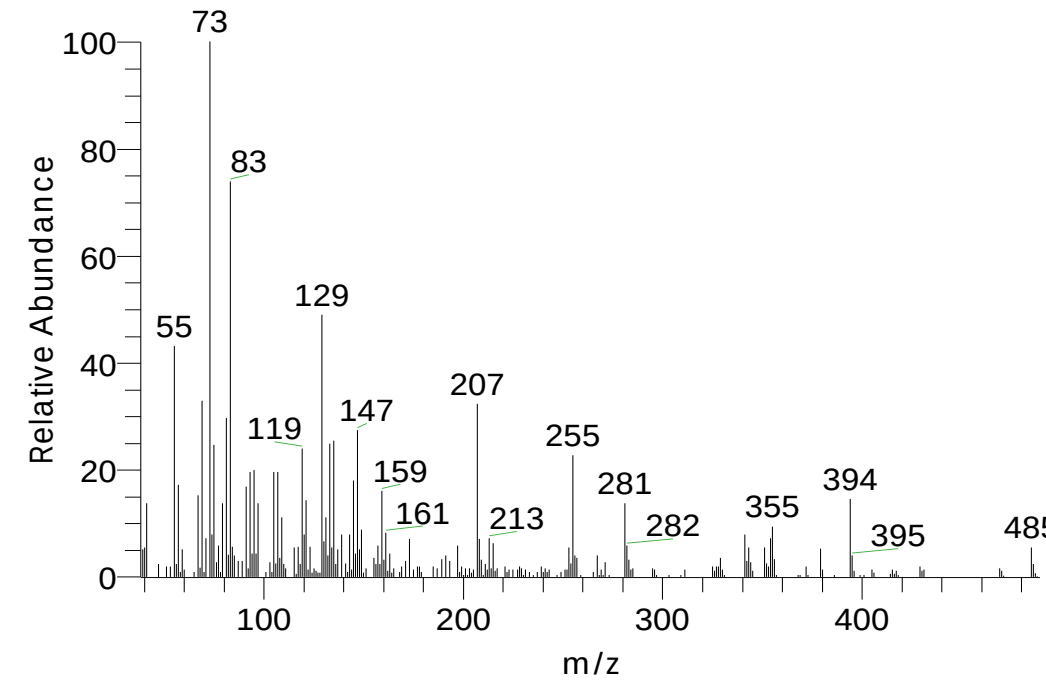

Oil_DrHafsa #12239 RT: 45.04 AV: 1 NL: 2.12E6
T: + c EI Full ms [50.000-750.000]

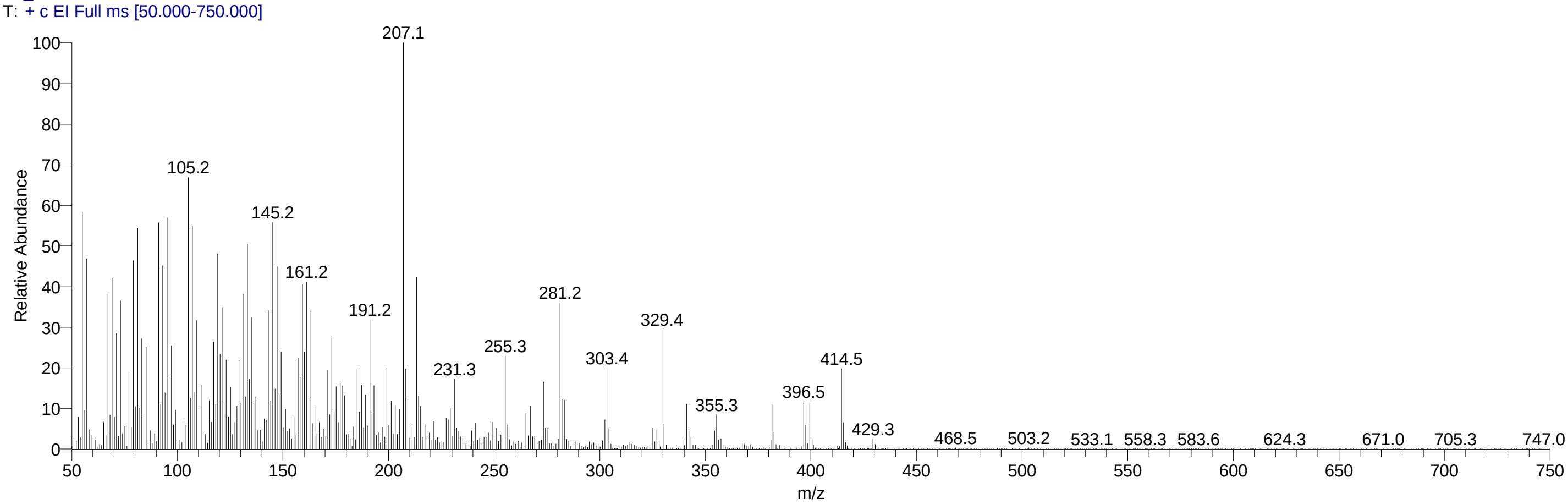

RT	Compound Name	Area %	MF	Molecular Formula	Molecular Weight	Cas #	Library
45.04	á-Sitosterol	6.94	848	C29H50O	414	83-46-5	replib
45.04	STIGMAST-5-EN-3-OL, (3á,24S)-	6.94	804	C29H50O	414	83-47-6	WileyRegistry8e
45.04	ç-Sitosterol	6.94	804	C29H50O	414	83-47-6	mainlib
45.04	STIGMAST-5-EN-3-OL, (3á,24S)-	6.94	804	C29H50O	414	83-47-6	WileyRegistry8e
45.04	ç-Sitosterol	6.94	829	C29H50O	414	83-47-6	replib

My GC-MS Report

Compound Structure | Hit Spectrum

á-Sitosterol
Formula C29H50O, MW 414, CAS# 83-46-5, Entry# 2073
Stigmast-5-en-3-ol, (3á)-

SI 799, RSI 848, replib, Entry# 2073, CAS# 83-46-5, á-Sitosterol

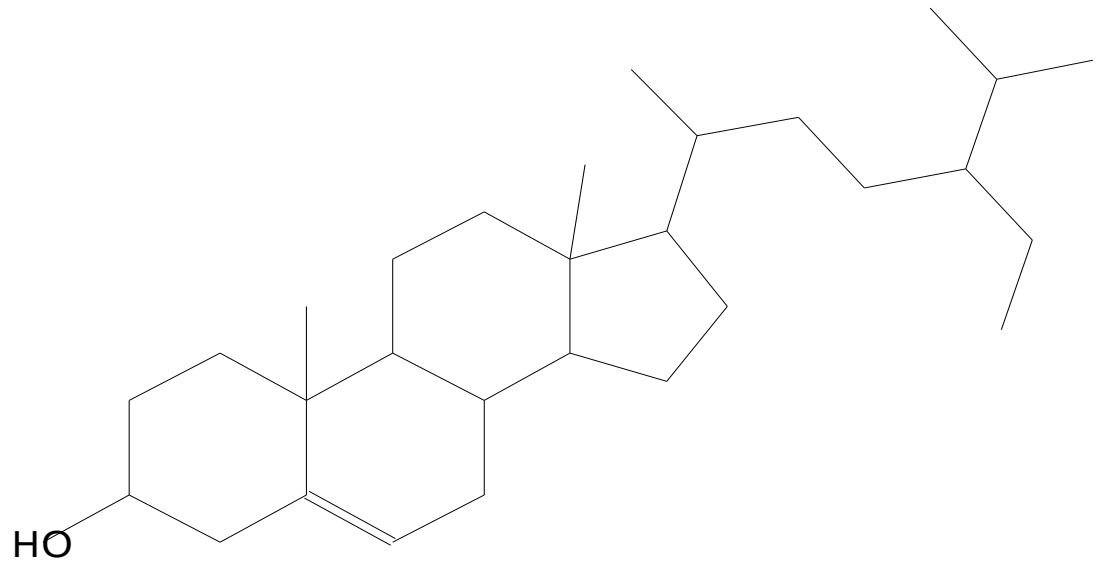

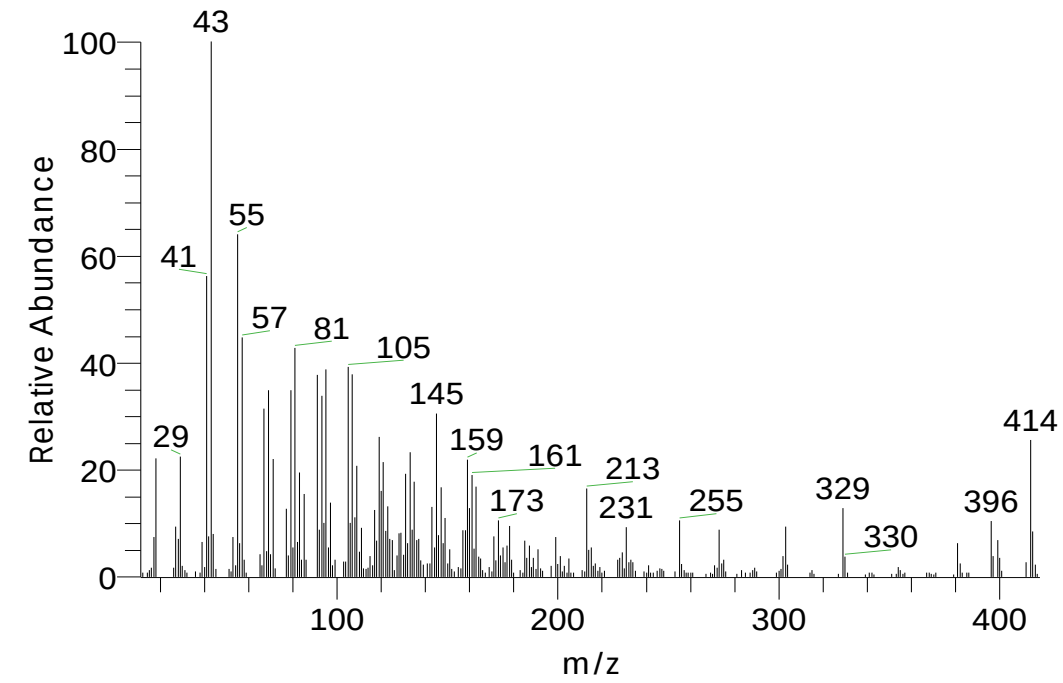

STIGMAST-5-EN-3-OL, (3á,24S)-
Formula C29H50O, MW 414, CAS# 83-47-6, Entry# 262357
STIGMAST-5-EN-3-OL #

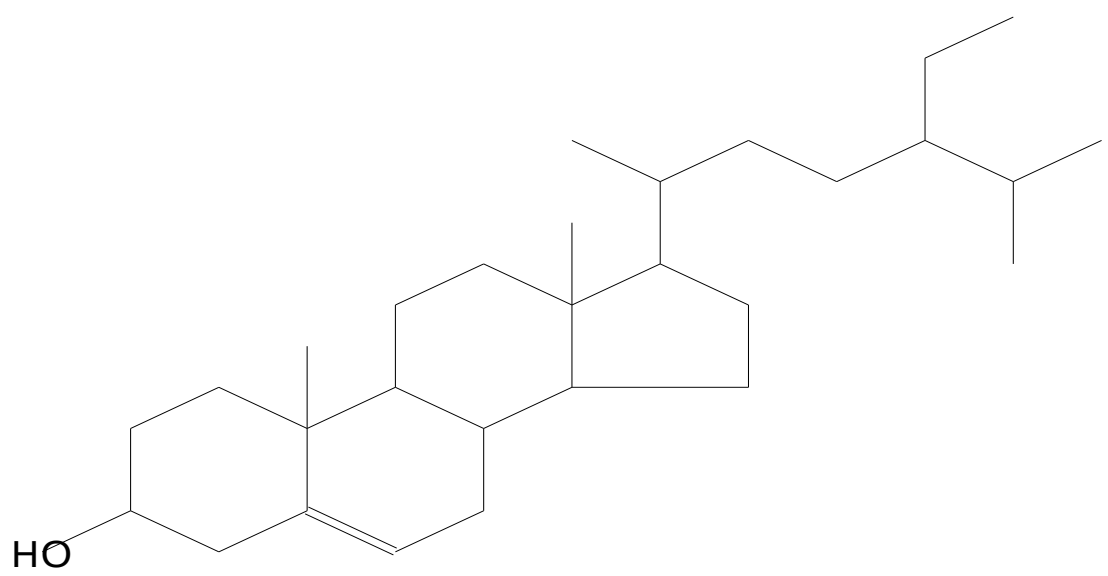

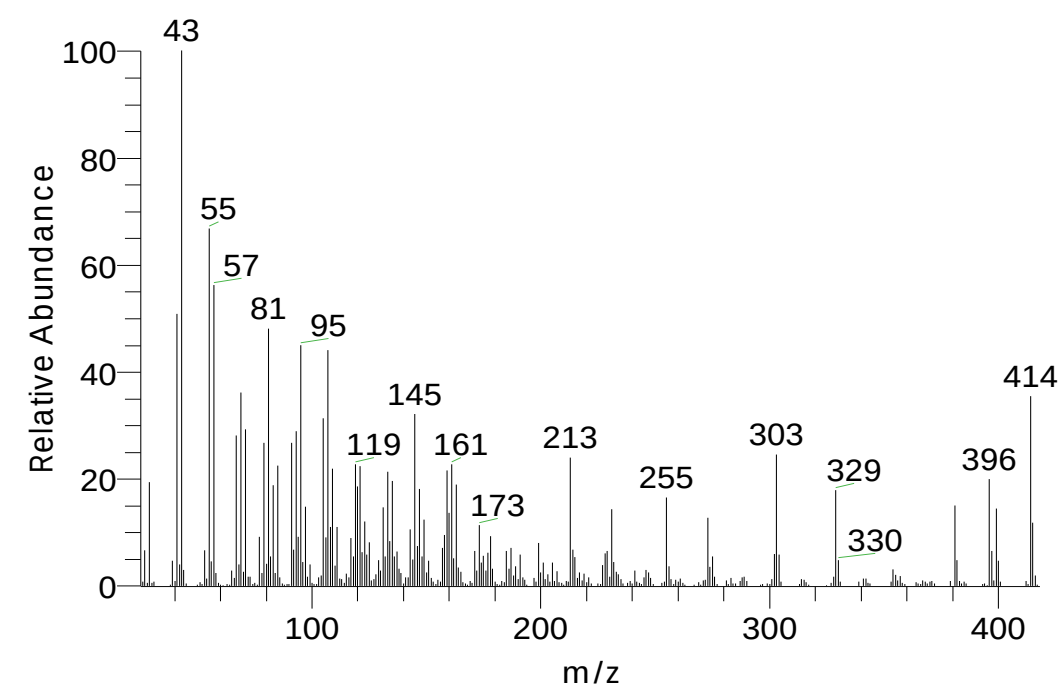

ç-Sitosterol
Formula C29H50O, MW 414, CAS# 83-47-6, Entry# 7212
Stigmast-5-en-3-ol, (3á,24S)-

SI 786, RSI 804, mainlib, Entry# 7212, CAS# 83-47-6, ç-Sitosterol

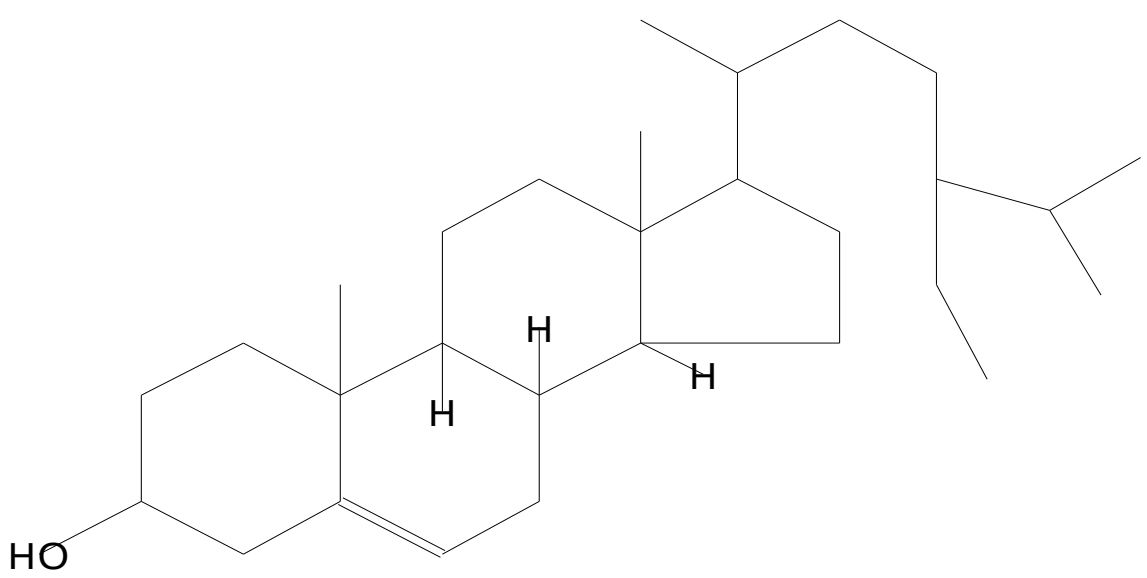

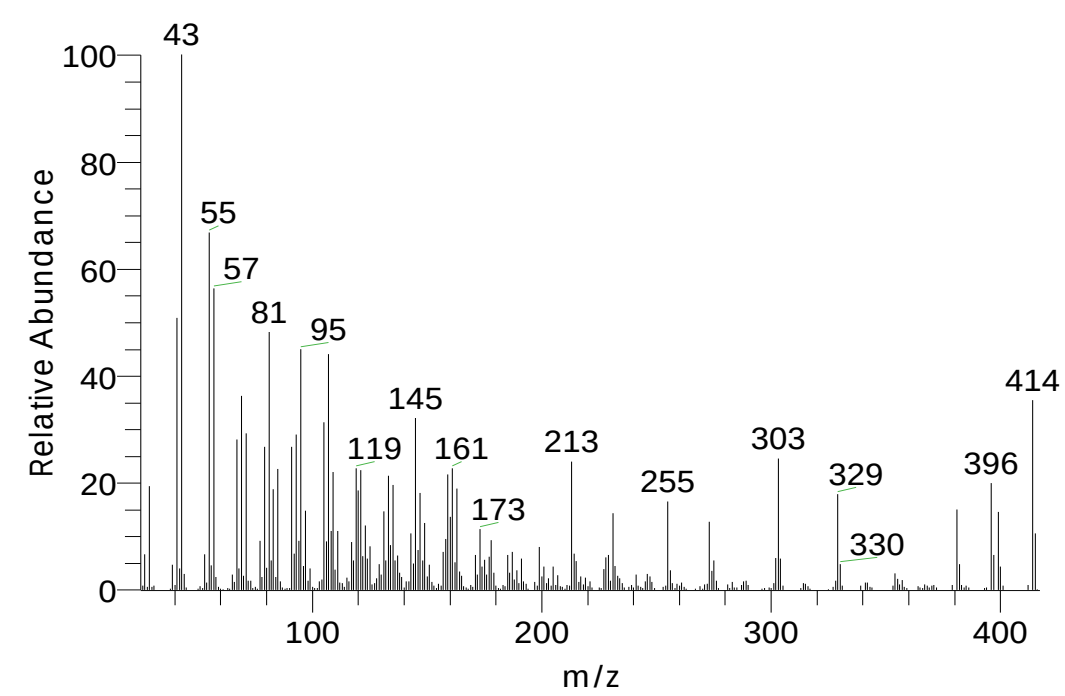

STIGMAST-5-EN-3-OL, (3á,24S)-
Formula C29H50O, MW 414, CAS# 83-47-6, Entry# 387446
STIGMAST-5-EN-3-OL #

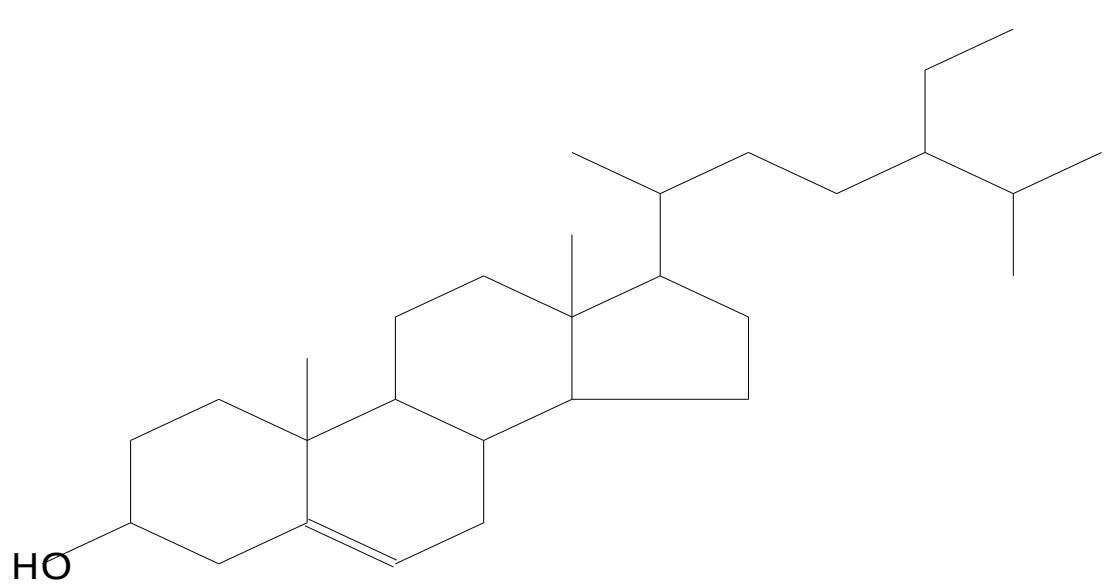

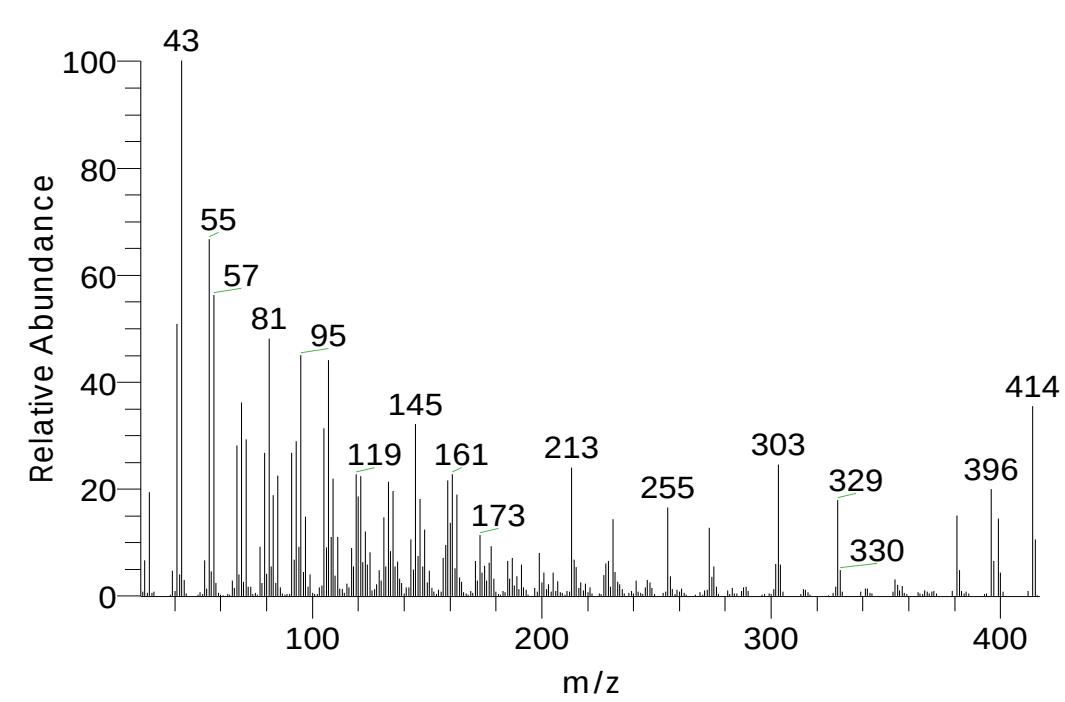

My GC-MS Report

Compound Structure | Hit Spectrum

ç-Sitosterol
Formula C29H50O, MW 414, CAS# 83-47-6, Entry# 33634
Stigmast-5-en-3-ol, (3á,24S)-

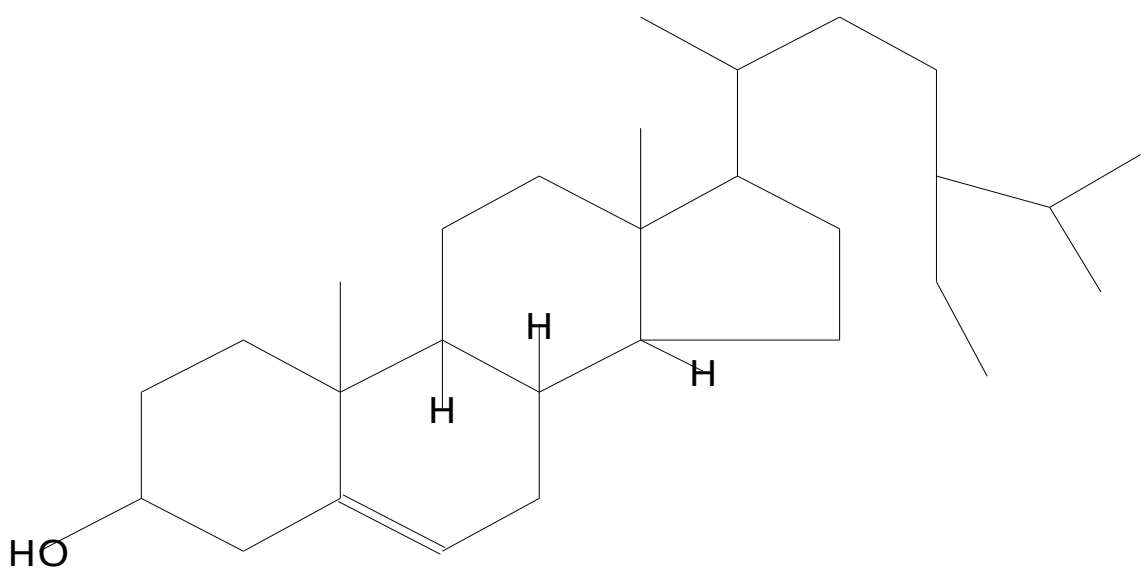

SI 782, RSI 829, replib, Entry# 33634, CAS# 83-47-6, ç-Sitosterol

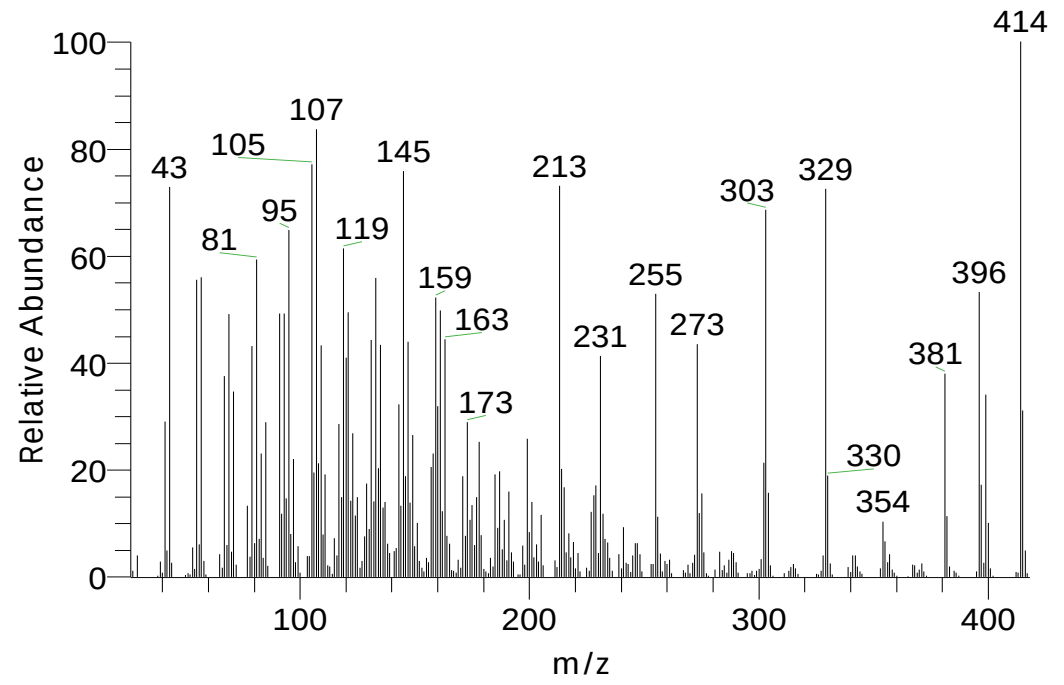

Oil_DrHafsa #12296 RT: 45.23 AV: 1 NL: 1.97E6
T: + c EI Full ms [50.000-750.000]

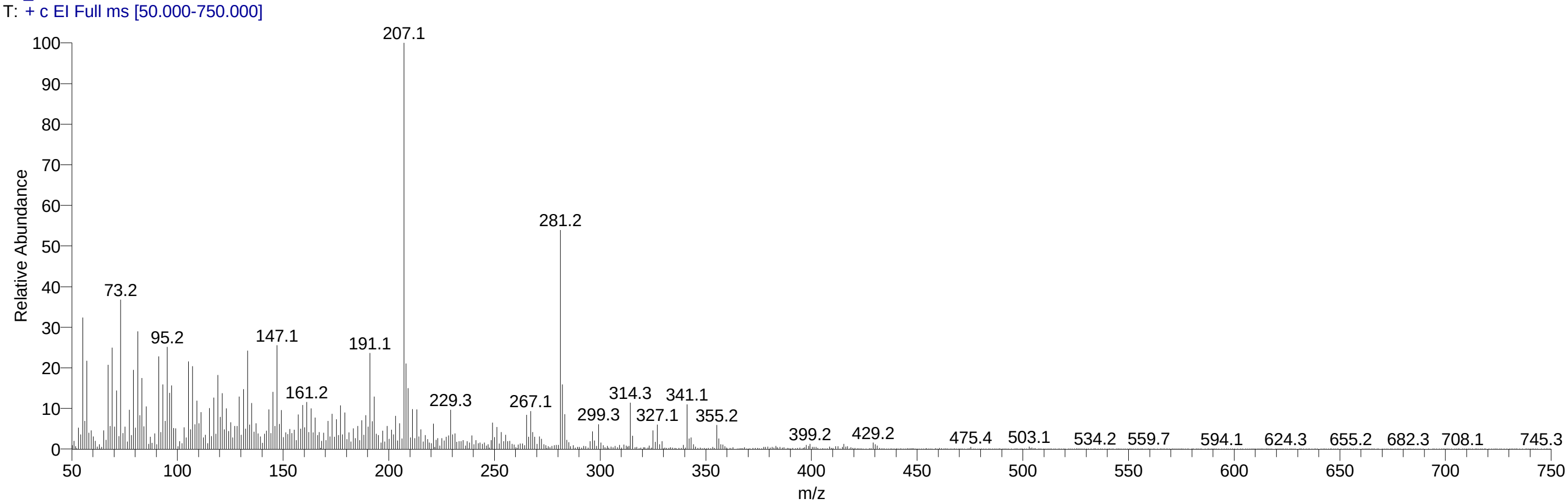

RT	Compound Name	Area %	MF	Molecular Formula	Molecular Weight	Cas #	Library
45.23	9,12-OCTADECADIENOIC ACID (Z,Z)-, 2,3-BIS[(TRIMETHYLSILYL)OXY]PROPYL ESTER	0.81	760	C27H54O4Si2	498	54284-45-6	WileyRegistry8e
45.23	.psi.,.psi.-Carotene, 1,1',2,2'-tetrahydro-1,1'-dimethoxy-	0.81	697	C42H64O2	600	13833-01-7	mainlib
45.23	.PSI.,.PSI.-CAROTENE, 1,1',2,2'-TETRAHYDRO-1,1'-DIMETHOXY-	0.81	696	C42H64O2	600	13833-01-7	WileyRegistry8e
45.23	9-OCTADECENOIC ACID (Z)-, 2-[(TRIMETHYLSILYL)OXY]-1-[[(TRIMETHYLSILYL)OXY]METHYL]ETHYL ESTER	0.81	713	C27H56O4Si2	500	54284-48-9	WileyRegistry8e
45.23	à-D-GALACTOPYRANOSIDE, METHYL 2,3-BIS-O-(TRIMETHYLSILYL)-, CYCLIC BUTYLBORONATE	0.81	713	C17H37BO6Si2	404	56211-11-1	WileyRegistry8e

Compound Structure | Hit Spectrum

9,12-OCTADECADIENOIC ACID (Z,Z)-, 2,3-BIS[(TRIMETHYLSILYL)OXY]PROPYL ESTER
Formula C27H54O4Si2, MW 498, CAS# 54284-45-6, Entry# 285148
2,3-BIS[(TRIMETHYLSILYL)OXY]PROPYL (9Z,12Z)-9,12-OCTADECADIENOATE #

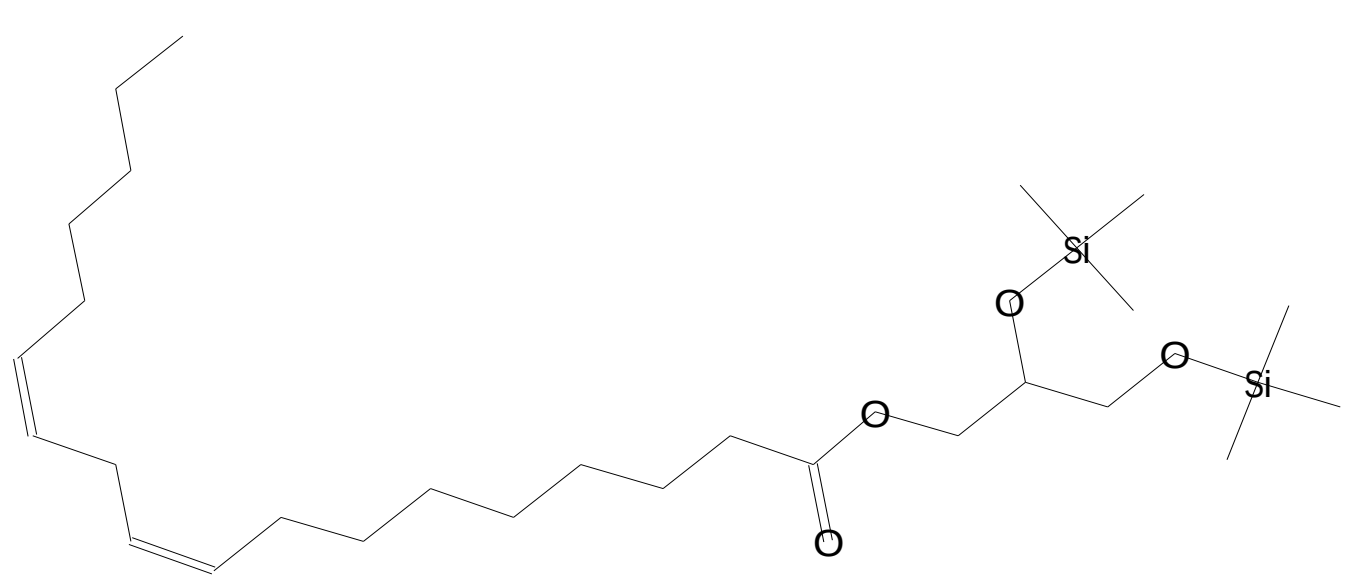

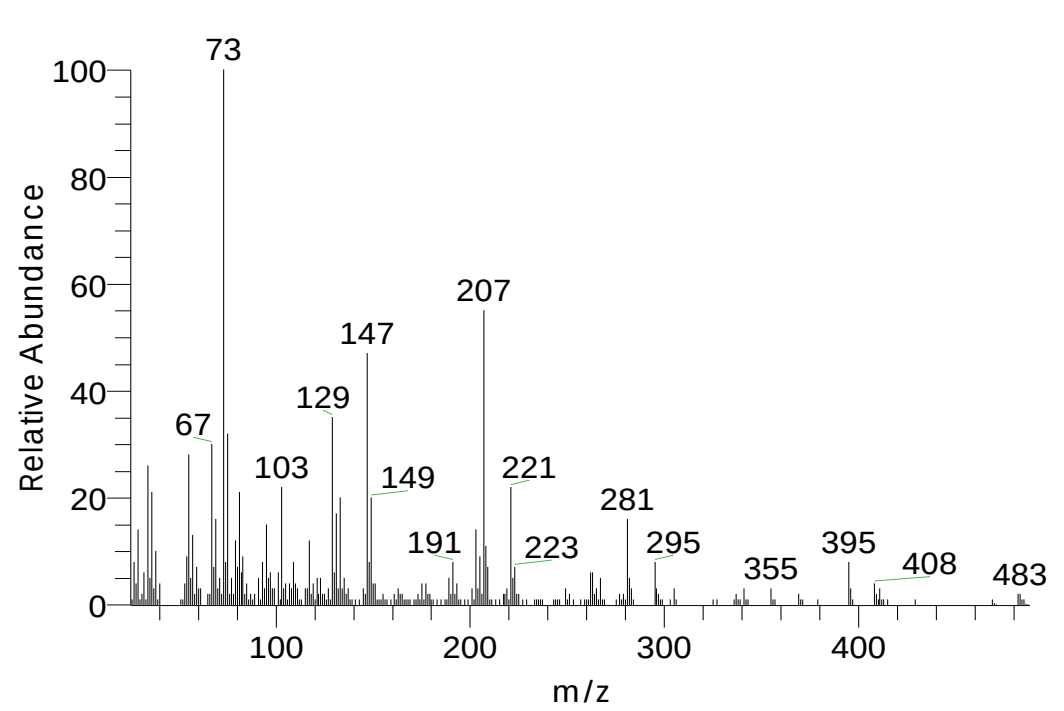

My GC-MS Report

Compound Structure | Hit Spectrum

.psi.,.psi.-Carotene, 1,1',2,2'-tetrahydro-1,1'-dimethoxy-
Formula C42H64O2, MW 600, CAS# 13833-01-7, Entry# 41205
Lycopene, 1,1',2,2'-tetrahydro-1,1'-dimethoxy-, all-trans-

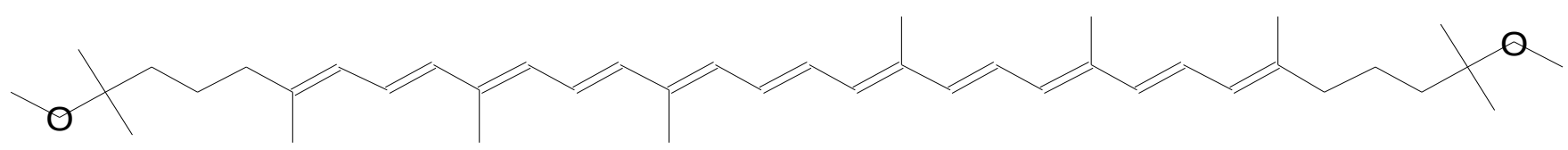

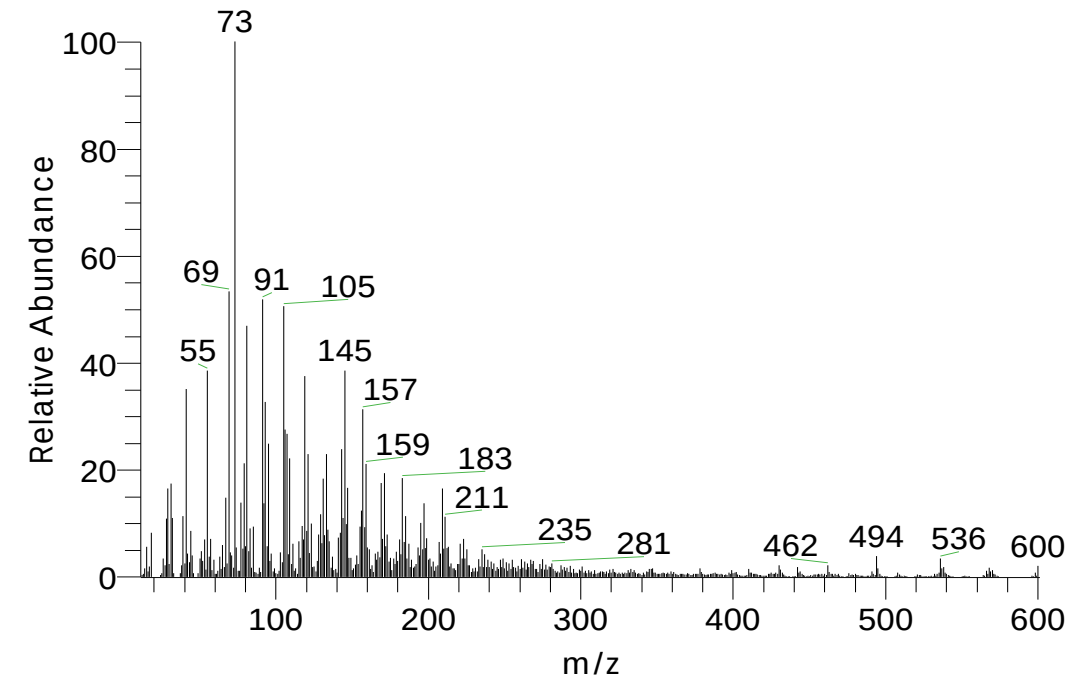

.PSI.,.PSI.-CAROTENE, 1,1',2,2'-TETRAHYDRO-1,1'-DIMETHOXY-
Formula C42H64O2, MW 600, CAS# 13833-01-7, Entry# 296796
1,1',2,2'-TETRAHYDRO-PSI,PSI-CAROTENE #

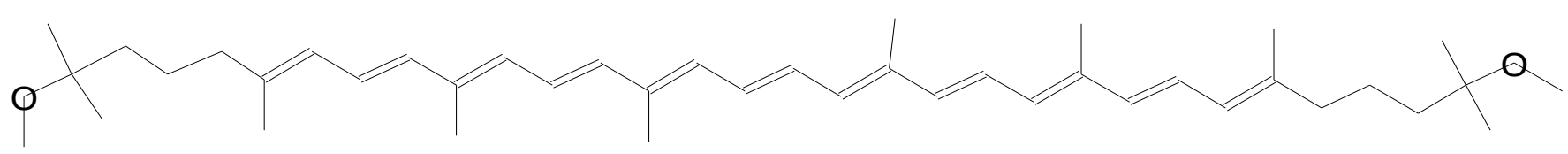

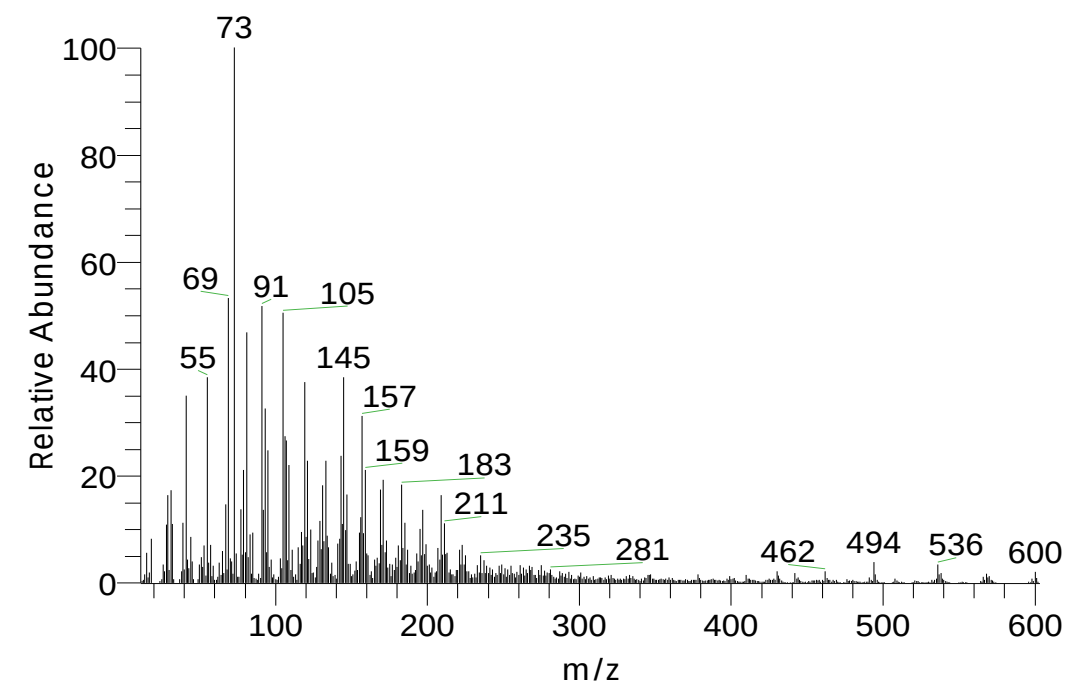

Formula C27H56O4Si2, MW 500, CAS# 54284-48-9, Entry# 285456
2-MONOOLEOYLGLYCEROL TRIMETHYLSILYL ETHER

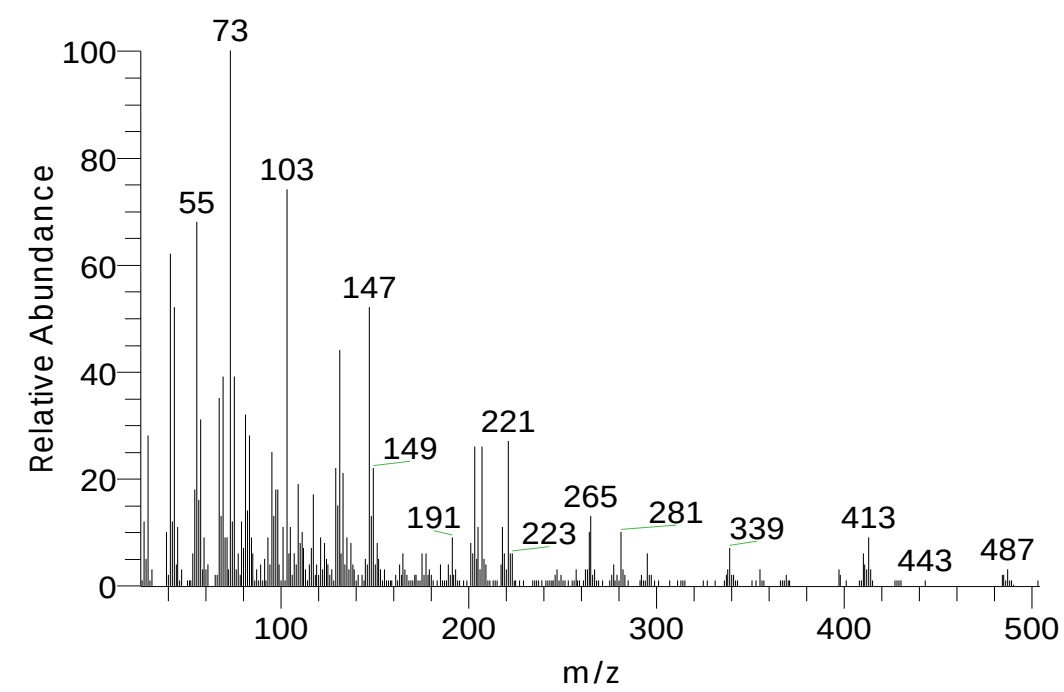

à-D-GALACTOPYRANOSIDE, METHYL 2,3-BIS-O-(TRIMETHYLSILYL)-, CYCLIC BUTYLBORONATE
Formula C17H37BO6Si2, MW 404, CAS# 56211-11-1, Entry# 257663

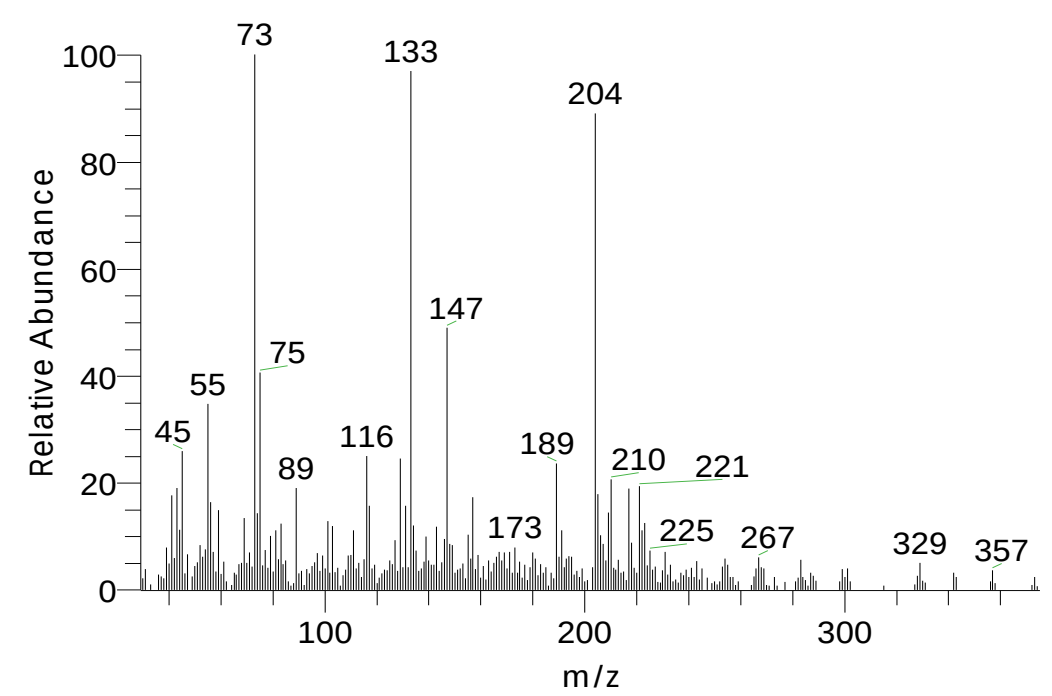

Supplement: Supplementary file 1 [file 41598_2026_55359_MOESM1_ESM.pdf]
